# Supplementary material for: Nature-inspired remodeling of (aza)indoles to meta-aminoaryl nicotinates for late-stage conjugation of vitamin B3 to (hetero)arylamines
Source: Nat Commun. 2020 Dec 9;11:6308. doi: 10.1038/s41467-020-19610-2 (PMC7726565; doi:10.1038/s41467-020-19610-2)
Supplement: Supplementary file 1 — Supplementary Information [file 41467_2020_19610_MOESM1_ESM.pdf]

< Supplementary Information >

Nature-inspired remodeling of (aza)indoles to *meta*-aminoaryl nicotinates for late-stage conjugation of vitamin B_3 to (hetero)arylamines

Begur Vasanthkumar Varun,† Kannan Vaithegi,† Sihyeong Yi, and Seung Bum Park*

CRI Center for Chemical Proteomics, Department of Chemistry

Seoul National University, Seoul 08826 (Korea)

*Corresponding author. E-mail: sbpark@snu.ac.kr

† These authors contributed equally.

Contents

I. Supplementary Figures and Tables ------------------------------------ 02

II. General Information --- 16

III. General Synthetic Procedures and
Characterization of All Starting Materials -------------------------- 17

IV. Experimental Synthetic Procedures and Characterization
of *meta*-Aminoaryl Nicotinate Final Products --------------------- 48

V. Late-stage Skeletal Transformation for the Synthesis
of Bioactive Nicotinates and Anilinic Drugs ---------------------- 73

VI. ^{1}H, ^{13}C, and ^{19}F NMR Spectra ------------------------------------- 86

VII. Supplementary References --- 248

I. Supplementary Figures and Tables

(1) Investigation of hypotheses and optimizing the reaction conditions

Initial reactions were performed on the basis of the hypothesis to simulate the biosynthetic formation of kynurenine/*N′*-formylkynurenine via cleavage of indole rings. Note that all screening reactions were performed in a 4.0 mL sealed vial equipped with a magnetic bar and a Teflon-lined screwed cap. As shown in Supplementary Figure 1, however, there is no exact method to cleave C2=C3 bond of the indole ring for the formation of *N′*-formylkynurenine. Therefore, we modified the biosynthetic route of vitamin B_3 and hypothesized that 3-formyl(aza)indoles can be transformed to *meta*-aminoaryl nicotinates by Aldol-type addition and dehydration followed by intramolecular cyclization, C–N bond cleavage and subsequent re-aromatization.

Supplementary Figure 1. Working hypothesis for simulating the biosynthetic formation of kynurenine via the cleavage of indole ring

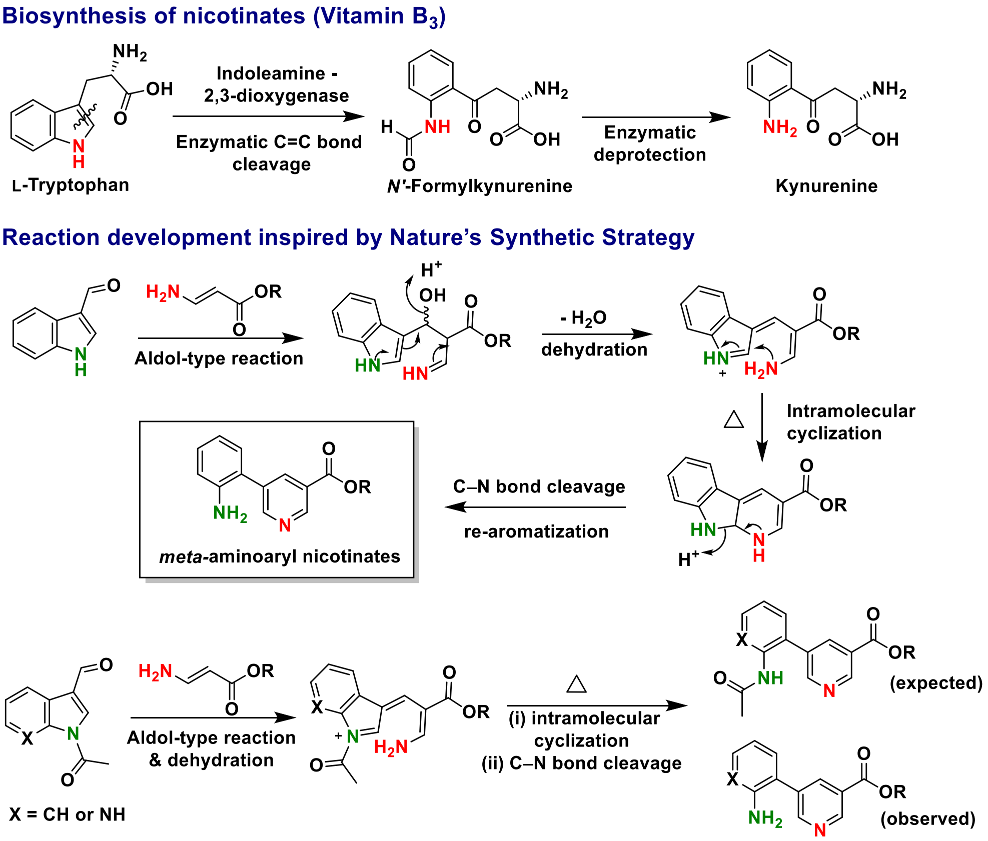

(2) Reaction using *N*-acetyl-protected (aza)indole-3-carboxaldehyde

Supplementary Figure 2. Unexpected deacylation using *N*-acyl 7-(aza)indole-3-carboxaldehydes with ethyl propiolate (**2a**) and NH_4OAc mixture under the reaction condition

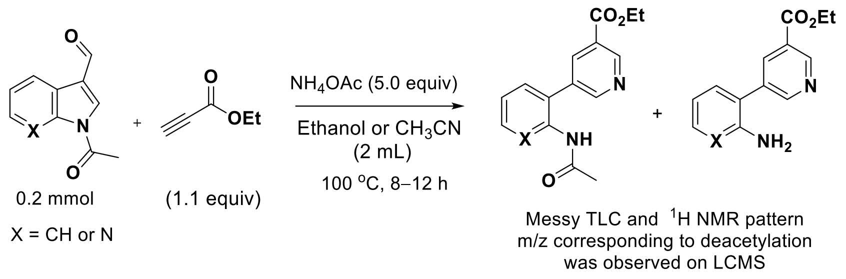

Reaction details and inference

We observed that *N*-acetyl-protected staring materials itself were not stable and susceptible to undergo deacetylation (based on TLC pattern). Reaction using commercially available *N*-acetyl-3-formylindole and *N*-acetyl-3-formylazaindole (prepared using the procedure reported in the literature[1]) with our optimized conditions showed messy TLC and ^1H NMR pattern. LC/MS also showed the presence of deacetylated product. However, we observed the formation of desired products using above reaction conditions, which supports our hypothesis of synthesis of vitamin B_3 scaffold via the cleavage of (aza)indole rings.

Substrate for optimization: Arylsulfonyl scaffold is a well-known profound moiety in a number of pharmaceutically active compounds. We observed that 7-azaindole scaffold is more reactive (with minimal byproduct formation) than the corresponding indole substrates. Hence, we choose *N*-phenylsulfonyl-7-azaindole-3-carboxaldehyde (**1a**) as a suitable substrate for the optimization study and further substrate scope study.

(3) Reaction optimization

All screening reactions were performed in a 4.0 mL sealed vial equipped with a magnetic bar and a Teflon-lined screwed cap. The yield of each reaction condition was confirmed by crude ^{1}H NMR analysis. All ^{1}H NMR yields were taken using 1,3,5-trimethoxybenzene as an internal standard. Values given in the parenthesis are isolated yields

Supplementary Table 1. Reaction optimization by solvent and reagent screening

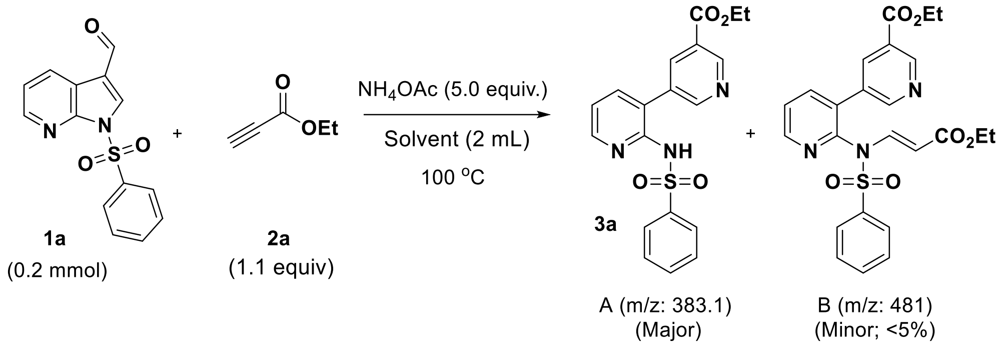

Entry	Solvent	Time (h)	% Yield (A)[a]
**1**	EtOH	12	88% (84%)
**2**	CH_3CN	12	81%
**3**	DCE	12	20%
**4**	DMSO	24	48%
**5**	DMF	24	60%
**6**	Dioxane	24	64%
**7**	THF	24	80%
**8**	Toluene	24	n.d.
	Deviation from Entry 1		
**10**	NH_4 (OAc) (3.0 equiv.)		80%
**11**	**2a** (1.2 equiv.), NH_4 (OAc) (4.0 equiv.), 8 h		98% (96%)

Notes: [a]Isolated yield given in the parenthesis is the average yield from at least 3 independent runs.

Supplementary Table 2. Reaction optimization by solvent screening for *N*-sulfonyl/benzyl-protected 3-formyl(aza)indoles.

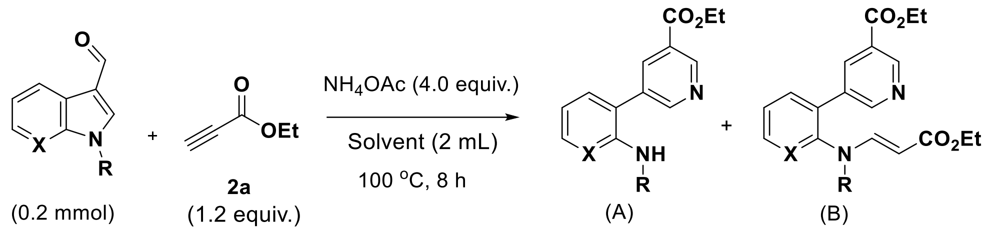

Entry	Solvent	X	N-Substitution	Yield (A)[a]	A:B (LCMS)
**1**	EtOH	-N	-SO_2Ph	98% (96%)	- [c]
**2**	CH_3CN	-N	-SO_2Ph	87% (76%)	93:7
**3**	EtOH	-N	-Bn	99% (96%)	- [c]
**4**	EtOH	-CH	-SO_2Ph	~90%	84:16
**5**	CH_3CN	-CH	-SO_2Ph	83% (90%)	91:9
**6**	EtOH	-CH	-Bn	97% (94%)	- [c]
**7 [b]**	CH_3CN	-CH	-Bn	83%	70:30

Notes: [a]Isolated yield given in the parenthesis is the average yield from at least 3 independent runs. [b]The reaction was not complete. [c]Byproduct B was not detected.

Under the standard reaction condition developed in Supplementary Table 1 (Entry 11), we observed the formation of undesired byproduct B in relatively high amount (~10–15%) and inseparable due to their similar polarity. Therefore, we performed solvent screening study for CH_3CN vs. EtOH. We generally observed that EtOH was generally the best solvent for complete conversion of starting material and the minimal formation of undesired byproducts, compared to CH_3CN, except the case of *N*-sulfonyl protected indole substrates. CH_3CN was selected as a good solvent for all *N*-sulfonyl-protected indole-3-carboxaldehydes. To avoid transesterification, MeOH was used as a solvent in the case of methyl propiolate and EtOH was used as a solvent in the case of ethyl propiolate. CH_3CN is used as solvent for all other propiolates.

General procedure for the reaction of *N*-substituted (aza)indole-3-carboxaldehydes. A 4-mL vial equipped with a magnetic stir bar and a Teflon-lined screwed cap was charged with **1** (0.2 mmol), ethyl propiolate (1.2 equiv.), and NH_4OAc (61.66 mg, 4 equiv.) in the appropriate solvent (2.0 mL, EtOH or CH_3CN). The vial was then sealed and heated at 100 ℃ for 8 h. Upon reaction completion checked by LC/MS and TLC analysis, the reaction mixture was concentrated under the reduced pressure, added with saturated aqueous $NaHCO_3$, and extracted with dichloromethane (DCM, 3 × 10 mL). The combined organic fractions were dried over anhydrous Na_2SO_4(s), filtered, and concentrated under reduced pressure. The crude compound was purified by silica-gel flash column chromatography to obtain the desired product bearing the nicotinate (**3**) or the nicotinamide (**4**) scaffolds.

Supplementary Table 3. Reaction optimization via catalyst screening for 3-formylazaindole (**5a**) without *N*-substituents

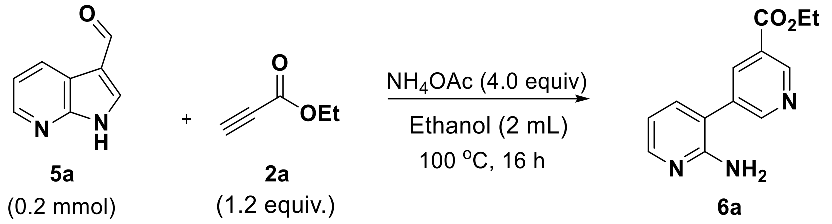

Entry	Catalyst (10 mol%)	Yield	Entry	Catalyst (10 mol%)	%Yield[b]
1[a]	-	56%	9	Ag_2CO_3	64%
2	CuI	63%	10	$AgNO_2$	66%
3	$CuCl_2.2H_2O$	62%	11	$AgNO_3$	64%
4	$Cu(OTf)_2$	60%	12	$AlCl_3$	35%
5	$ZnCl_2$	71%	13	$FeCl_3$	65%
7	$Zn(OTf)_2$	66%	15	$BF_3{\cdot}Et_2O$	63%
8	AgOTf	62%	16	CAN	71%
	Deviation from Entry 1				
9	no catalyst, 80 °C				41%
10	no catalyst, NH_4OAc (5.0 equiv.)				65−70% (65%)
11	$ZnCl_2$ (10 mol%), NH_4OAc (3.0 equiv.)				59%
12	$ZnCl_2$ (5 mol%, 20 mol%)				70%
13	Zn $(OTf)_2$ (5 mol%, 15 mol%), NH_4OAc (5.0 equiv.)				70%
14	BF_3Et_2O (30 mol%)				74% (82%)
15	BF_3Et_2O (30 mol%), NH_4OAc (3.0 equiv.)				68%
16	BF_3Et_2O (30 mol%), NH_4OAc (5.0 equiv.)				74% (78%)
17[a]	no catalyst, 120 °C				66%
18	$ZnCl_2$ (10 mol%), 120 °C				72%
19[b]	Zn $(OTf)_2$ (10 mol%), 120 °C				72% (85%)
20	Zn $(OTf)_2$ (10 mol%), NH_4OAc (5.0 equiv.), 120 °C				76%
21[b]	Zn $(OTf)_2$ (10 mol%), NH_4OAc (5.0 equiv.), 6 h				76% (90%)

Notes: [a] ^{1}H NMR yields obtained without catalyst were not consistent. [b]Isolated yield given in the parenthesis is the average yield from at least 3 independent runs.

Supplementary Table 4. Reaction optimization via catalyst screening for 3-formylindole (**5f**) without *N*-substituents

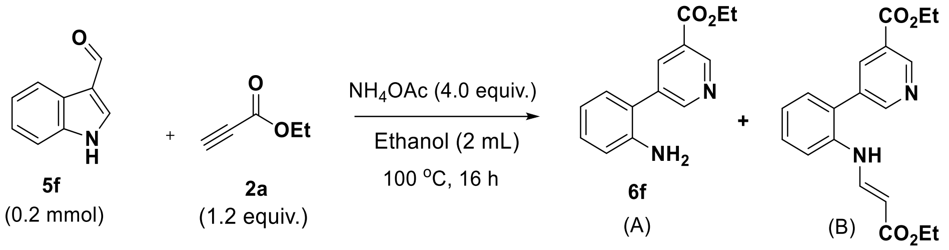

Entry	Conditions	LC/MS SM:A:B	Yield[d]
1[b]	No deviation		~79%
2	$BF_3 \cdot Et_2O$ (30 mol%)		~66%
3[c]	1.5 equiv. of propiolate		(88%)
5[c]	1.5 equiv. of propiolate, $Zn(OTf)_2$ (10 mol%)		(88%)
6	CH_3CN (2.0 mL) as solvent	33:60:7	
7	CH_3CN (2.0 mL), $BF_3 \cdot Et_2O$ (30 mol%)	6:92:2	
8	CH_3CN (2.0 mL), $Zn(OTf)_2$ (10 mol%)	11:87:2	75%
9[b]	CH_3CN (2.0 mL), 120 °C	33:63:4	~82%
10	CH_3CN (2.0 mL), $BF_3 \cdot Et_2O$ (30 mol%), 120 °C	0:97:3	76%
11	CH_3CN (2.0 mL), $Zn(OTf)_2$ (10 mol%), 120 °C	0:97:3	99% (90%)
12	CH_3CN (2.0 mL), $Zn(OTf)_2$ (5 mol%), 120 °C		78%
13	CH_3CN (2.0 mL), $Zn(OTf)_2$ (10 mol%), 120 °C, 8−12h		75−80%

Notes: [a]Generally all the reactions using ethanol produced undesired byproducts. [b] ^{1}H NMR yields obtained without catalyst were not consistent and messy. [c]Isolated products were not clean and contains inseparable byproducts. [d]Isolated yield given in the parenthesis is the average yield from at least 3 independent runs.

General procedure for the reaction of (aza)indole-3-carboxaldehydes. A 4-mL vial equipped with a magnetic stir bar and a Teflon-lined screwed cap was charged with **5** (0.2 mmol), **2** (1.2 equiv.), NH_4OAc (77.08 mg, 5 equiv.), and $Zn(OTf)_2$ (7.27 mg, 0.1 equiv.) in the appropriate solvent (2.0 mL, EtOH or CH_3CN). The vial was then sealed and heated at 120 °C for the desired times (6–16 h). Upon

reaction completion checked by TLC analysis, the reaction mixture was concentrated under the reduced pressure, added with saturated aqueous $NaHCO_3$, and extracted with DCM (3 × 10 mL). The combined organic fractions were dried over anhydrous Na_2SO_4(s), filtered, and concentrated under reduced pressure. The crude compound was purified by silica-gel flash column chromatography to obtain the desired products bearing the nicotinate (**6**) or the nicotinamide (**7**) scaffolds.

Supplementary Figure 3. Reaction using a commercially available E/Z *β*-amino acrylate

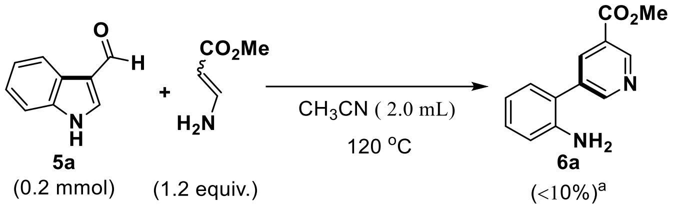

Deviation from standard condition

Entry	Activator	5a:6a (LCMS)[a]
1[b]	AcOH (1.2 equiv.)	80:20
2	AcOH (1.2 equiv.)	86:09
3[c]	$Zn(OTf)_2$ (10 mol%)	90:06

Notes: [a]Conversion of **5a** to **6a** (based on ^{1}H NMR and LC/MS) in all the reactions. [b]Ethanol was used as a solvent. [c]These reactions were performed after optimization of the reaction conditions.

(4) Investigation of hypothesis to generate *β*-aminoacrylate equivalents from propiolates

As the reaction using *β*-aminoacrylate was not successful, we hypothesized the *in situ* generation of *β*-aminoacrylate from propiolate (Supplementary Figure 4) based on the literature evidence where NH_4OAc is known to form cation complexes by activating the alkyne triple bond *via* a cation-π interaction[2] and on the other hand, NH_4OAc is also used as an ammonium source for enamine synthesis[3].This hypothesis was encouraged by ^1H NMR analysis of the reaction mixture obtained by heating methyl propiolate at 100 °C in CD_3CN for 10–20 min in the presence of NH_4OAc. ^1H NMR analysis of crude reaction mixture showed the formation of two doublet peaks (δ 4.82, J = 13.2 Hz and δ 4.44, J = 8.0 Hz), which indicate olefinic protons from the E/Z mixture (0.34:1) of methyl *β*-aminoacrylate *in situ* generated by the reaction of methyl propiolate with NH_4OAc.

Supplementary Figure 4. Use of methyl propiolate and NH_4OAc mixture to confirm the *in situ* generation of methyl *β*-aminoacrylate.

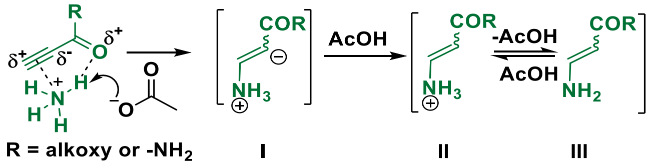

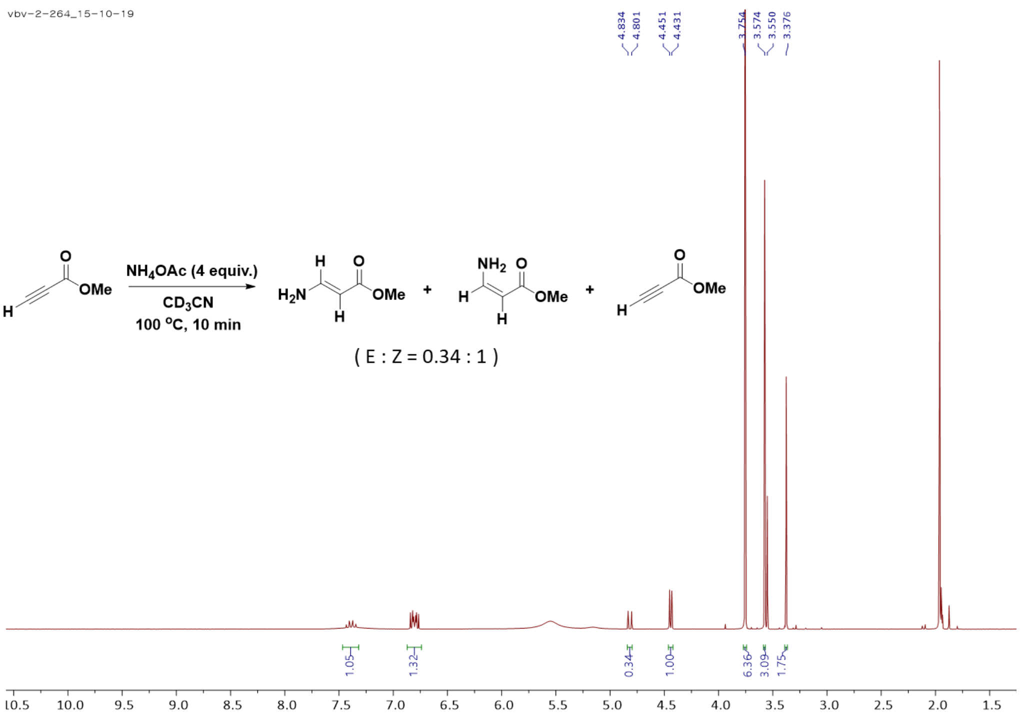

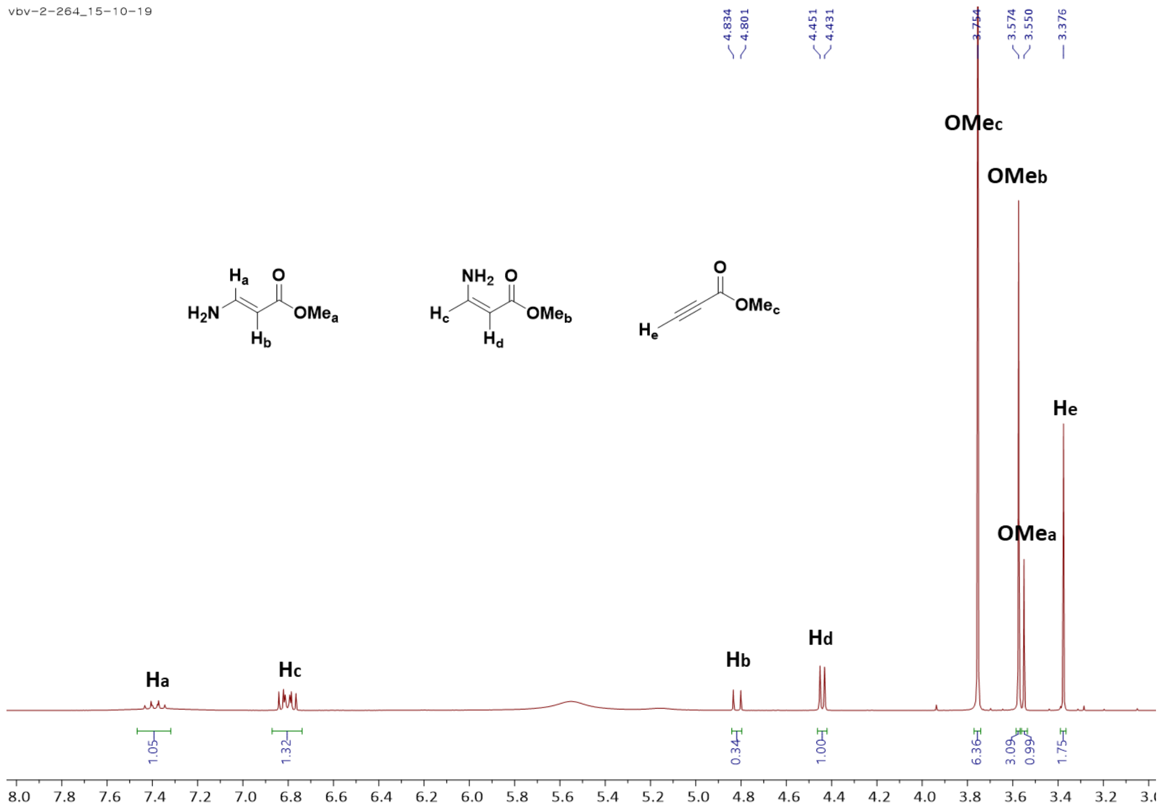
vbv-2-264_15-10-19
4.834
4.801
4.451
4.431
3.754
3.574
3.550
3.376
OMec
OMeb
He
OMea
Hd
Hb
Hc
Ha
Ha
O
H2N
OMea
Hb
NH2
O
Hc
OMeb
Hd
O
OMec
He
1.05
1.32
0.34
1.00
6.36
3.09
0.99
1.75
8.0 7.8 7.6 7.4 7.2 7.0 6.8 6.6 6.4 6.2 6.0 5.8 5.6 5.4 5.2 5.0 4.8 4.6 4.4 4.2 4.0 3.8 3.6 3.4 3.2

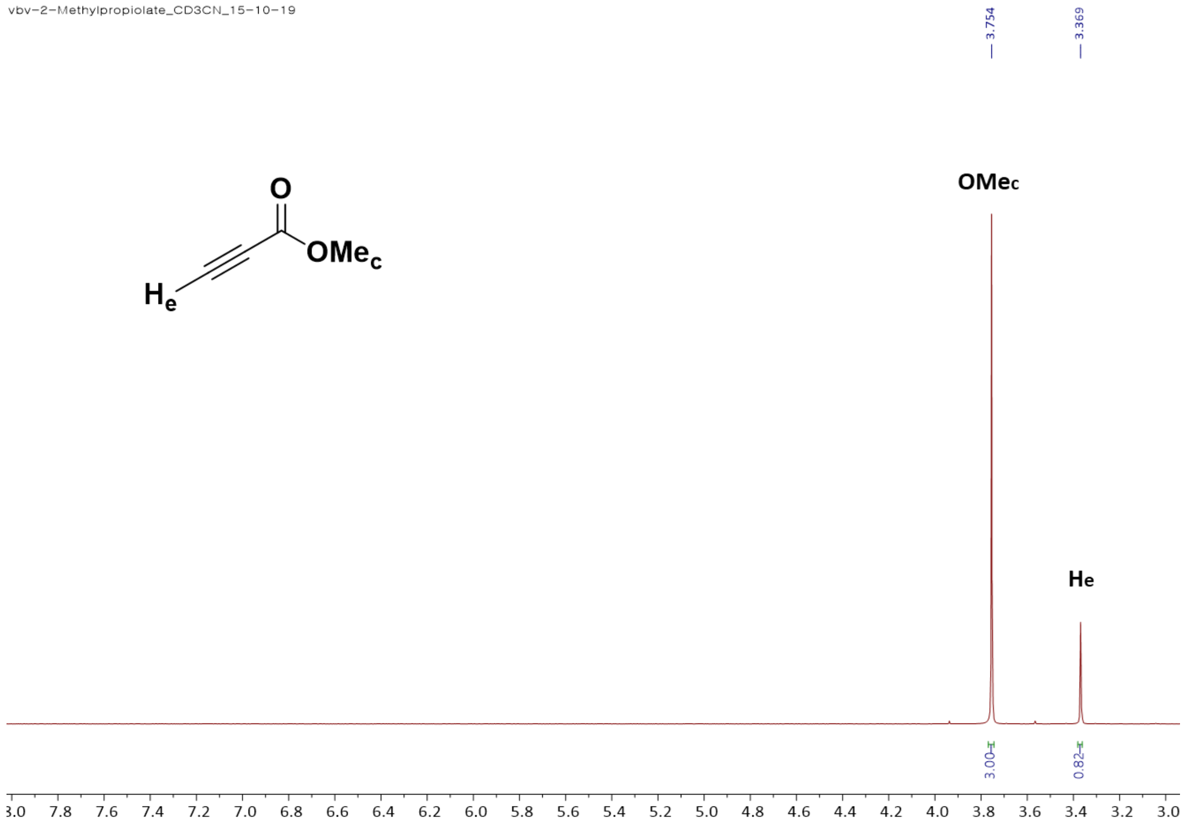
vbv-2-Methylpropiolate_CD3CN_15-10-19
3.754
3.369
O
OMec
He
OMec
He
3.00
0.82
7.8 7.6 7.4 7.2 7.0 6.8 6.6 6.4 6.2 6.0 5.8 5.6 5.4 5.2 5.0 4.8 4.6 4.4 4.2 4.0 3.8 3.6 3.4 3.2 3.0

(5) Mechanistic investigations

Supplementary Figure 5. *Plausible reaction pathways*

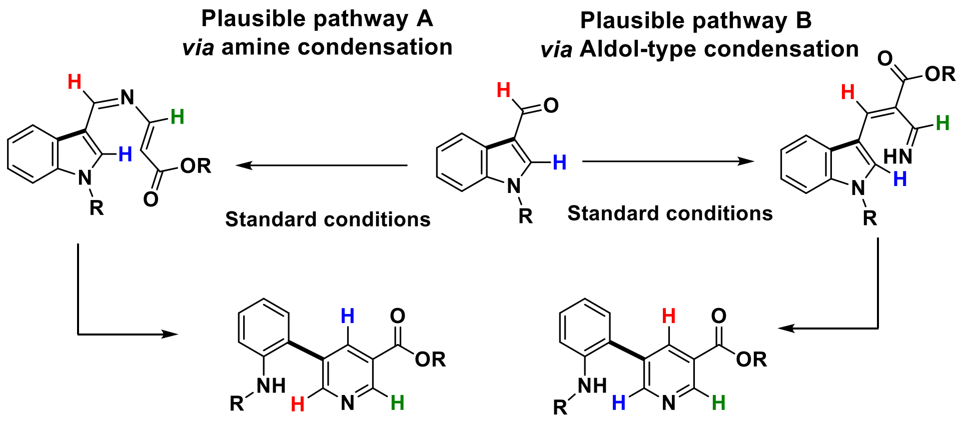

Explanations: As shown in Supplementary Figure 5, there are two possible reaction pathways which lead to the same product, *meta*-amino(hetero)aryl nicotinates. From the deuterium-labeling experiments, we confirmed that this biomimetic re-modeling of 3-formyl(aza)indole is proceeded via Plausible Pathway B via Aldol-type condensation followed by intramolecular cyclization and C–N bond cleavage. In addition, as described in Supplementary Figure 3, we observed very low reactivity when *β*-aminoacrylates were used as a reactant and the addition of acetic acid or $Zn(OTf)_2$ did not improve the reaction yields. Interestingly, we observed a slight improvement in yields (^{1}H NMR yield; ~35%) when reaction was performed in the presence of NH_4OAc (5.0 equiv.), as shown in Supplementary Figure 6. Therefore, NH_4OAc plays an important role in driving the reaction equilibrium as a catalyst for Aldol-type addition. However, the reaction with *β*-aminoacrylates is still not efficient, compared to our optimized protocol using propiolates and NH_4OAc via *in situ* generation of structural equivalents *β*-aminoacrylate.

Supplementary Figure 6. Reaction using E/Z mixture of β-aminoacrylate in the presence of NH_4OAc

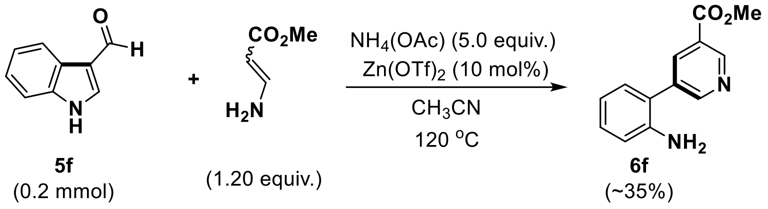

The above observation in Supplementary Figure 6 provides some clues for the overall mechanism of this transformation. As shown in Supplementary Figure 7, this transformation was initiated via two possible routes of Aldol-type addition, one by the addition of activated propiolates to 3-formyl(aza)indoles (route 1) and the other by *α*-carbanion addition of *β*-aminoacrylate (**I**) to 3-formyl(aza)indoles (route 2). The resulting Aldol addition intermediate **II** then undergoes the dehydration to yield the intermediate **III**, followed by intramolecular cyclization to generate relatively strained 6/5/6-fused tricyclic intermediate **VI**. This tryicyclic intermediate **VI** can undergo re-aromatization as a consequence of C_2–N bond cleavage that relieves the ring strain to form the desired *meta*-amino(hetero)aryl nicotinate scaffolds **VII**.

Supplementary Figure 7. Reaction pathway initiated by Aldol-type addition reaction

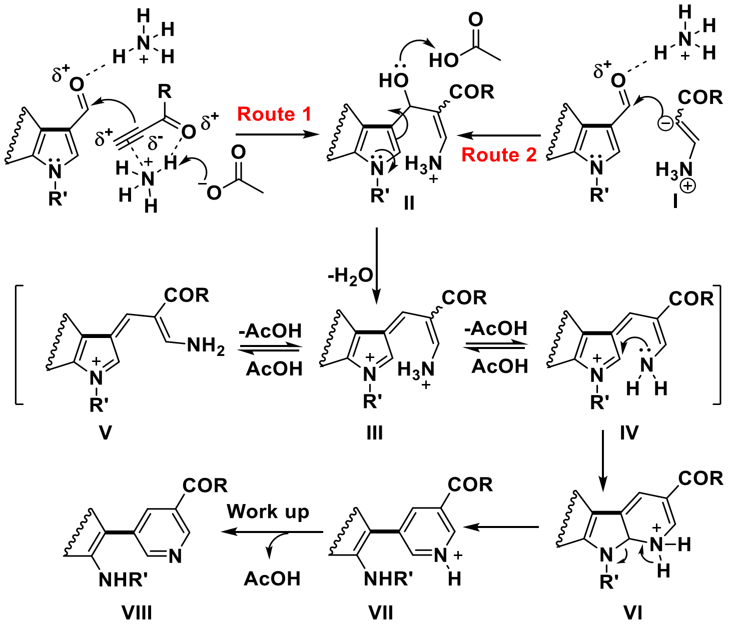

(6) Influence of electronic nature of substituent on the overall reactivity

Explanation: Under the optimized conditions (featuring a long reaction time in a sealed vial), no considerable differences was observed in the reactivity among various substrates containing electron-withdrawing (EWG) and electron-donating (EDG) substituents. In fact, electron-donating and electron-withdrawing substituents in 3-formyl(aza)indoles can influence the reaction mechanism in opposite manners at the different steps of cascade reactions, whereby these electronic effects possibly become mutually compensated under the long reaction time. Therefore, we performed a series of experiments in parallel under identical reaction conditions featuring a short reaction time of 30 min to examine the initial rate of product formation, which may disclose the electronic effects of substituents on the reactivity of substrates. As shown in Supplementary Table 5, azaindole-3-carboxaldehyde **5a** is more reactive than the corresponding indole-3-carboxaldehyde **5f** (Entry 1), and C_5-nitro-substituted indole-3-carboxaldehyde is more reactive than the corresponding methoxy-substituted one (Entry 2). These results indicate that the presence of EWG on the indole ring increases the reactivity of the substrates which is possibly due to the increased rate of C_2–N bond cleavage. Similarly, the low reactivity of **1a** might be due to the decreased rate of intramolecular cyclization which can be influenced by the density of lone pair electrons on indole nitrogen. Surprisingly, the C-4 substituted indole-3-caroboxaldehydes showed exceptionally high reactivity irrespective of electronic natures of substituent (Entry 4, Supplementary Table 5). This exceptional variation might be due to the influence of their torsional strains on the reaction rate as shown in the schematic representation**.**

Supplementary Table 5. Exploring the electronic effects of substituents on the reaction rate

General information: In order to eliminate the solvent influence, all the reactions were performed in CH_3CN. After 30 minutes of the reaction, the reaction mixture was cooled to room temperature, evaporated and the ^1H NMR of the crude compound was taken in DMSO-d_6, ^1H NMR yields were taken using Mesitylene (0.1 mmol, 13.8 µL) as an internal standard.

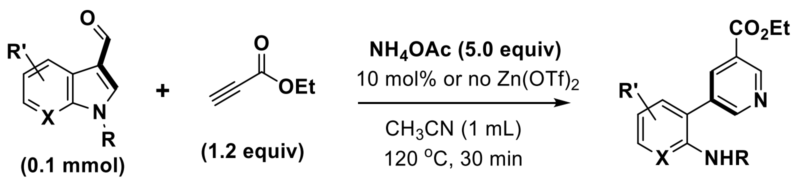

Entry	Starting compound	X	R	R'	Yield (^1H NMR Yield)
1	**5a**	N	H	H	53%
	**5f**	CH	H	H	27%
2	**5h**	CH	H	5-MeO	22%
	**5l**	CH	H	5-NO_2	67%
	**5i**	CH	H	6-MeO	30%
	**5m**	CH	H	6-NO_2	62%
3	**1a**	CH	SO_2Ph	H	26%
4	**5g**	CH	H	4-MeO	85%
	**5k**	CH	H	4-NO_2	70%

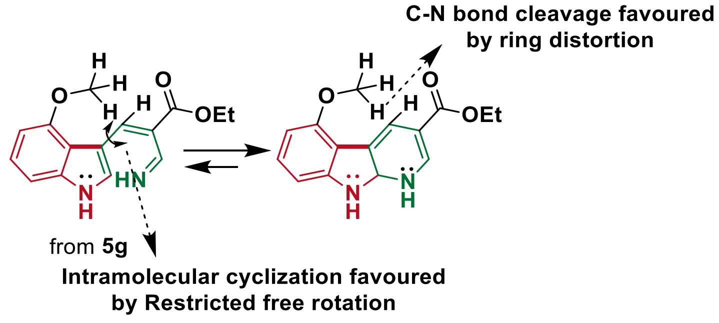

II. General Information

NMR spectra were obtained on an Agilent 400-MR DD2 Nuclear Magnetic Resonance System (400 MHz, Agilent, USA), JEOL JNM-LA400 with LFG (400 MHz, Jeol, Japan) or Varian/Oxford Unity Inova 500 MHz (Varian Assoc., Palo Alto, USA). Chemical shifts values were recorded as parts per million (δ), referenced to tetramethylsilane (TMS) as the internal standard or to the residual solvent peak ($CDCl_3$, ^{1}H: 0.00, ^{13}C: 77.16, CD_2Cl_2, ^{1}H: 0.00, ^{13}C: 54.00, CD_3OD, ^{1}H: 0.00, ^{13}C: 49.00, DMSO-d_6, ^{1}H: 0.00, ^{13}C: 39.51). Multiplicities were indicated as follows: s (singlet), d (doublet), t (triplet), q (quartet); m (multiplet); dd (doublet of doublet); dt (doublet of triplet); td (triplet of doublets); br s (broad singlet) and so on. Coupling constants were reported in hertz (Hz). Low resolution mass spectrometry (LRMS) was conducted by LCMS-2020 [Shimadzu]. High resolution mass spectrometry (HRMS) of final compounds was confirmed by Ultra High Resolution ESI Q-TOF mass spectrometer [Bruker] from Organic Chemistry Research Center at Sogang University. All commercially available reagents were used without further purification unless noted otherwise. Commercially available reagents were obtained from Sigma-Aldrich, TCI, Acros, or Alfa Aesar. All solvents were obtained by passing them through activated alumina columns of solvent purification systems from Glass Contour. Analytical thin-layer chromatography (TLC) was performed using Merck Kiselgel 60 F_{254} plates, and the components were visualized by observation under UV light (254 and 365 nm) or by treating the plates with ninhydrin followed by thermal visualization. Flash column chromatography was performed on Merck Kieselgel 60 (230-400 mesh) Biotage® Selekt and Isolera One (ZIP® KP-Sil and Sfär columns were used). Microwave reactions were performed using the Biotage® Robot Eight [Biotage] and microwave reaction conditions were as indicated in the Experimental Section. Infrared (IR) spectra were recorded on a PerkinElmer Spectrum IR Version 10.6.0. as neat sample and are reported in frequency of absorption (cm^{-1}). HPLC purification was performed on an Agilent 1260 Infinity system [Agilent] with an YMC-Pack silica column (SL12S05-2520WTX, 250 mm × 20 mm, 5 μm).

III. General Synthetic Procedures and Characterization of All Starting Materials

Supplementary Figure 8. Synthesis of starting materials azaindole-3-carboxaldehydes

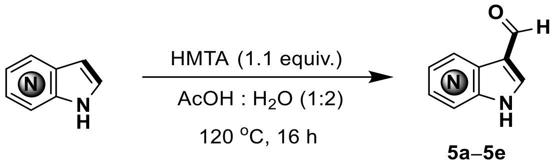

***General Procedure*:** Substituted 3-formylazaindoles were synthesized using the general procedure reported in the literature[4]. The mixture of azaindole derivative, hexamethylenetetramine (HMTA, 1.1 equiv.), AcOH and water was heated at 120 ℃ for 12 h in a round bottom flask equipped with a magnetic bar and a reflux condenser. X mL of water and X/2 mL of AcOH used for X mmol of azaindole. Precipitation was observed upon cooling the reaction mixture to room temperature (r.t.) and diluted with cold water. The precipitate was filtered, washed with cold water and hexane, and dried under the reduced pressure to afford the expected product. When precipitation was not observed especially with the small-scale reaction, the crude compound was extracted into ethyl acetate (EtOAc) or 10–20% DCM/MeOH and purified by silica-gel flash column chromatography. Acetal formation and product degradation were observed when MeOH/DCM mixture was used as an eluent for silica-gel flash column chromatography.

Compound 5a: 1*H*-Pyrrolo[2,3-b]pyridine-3-carbaldehyde

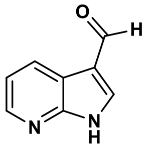

Following the general procedure described above with 7-azaindole (5 g, 42.32 mmol) provided **5a** in 85% yield (5.26 g) as off-white solid. ^{1}H NMR (500 MHz, DMSO-d_6) δ 12.73 (s, 1H), 9.97 (s, 1H), 8.50 (s, 1H), 8.44 (dd, J = 8.0, 1.5 Hz, 1H), 8.40 (dd, J = 4.5, 1.5 Hz, 1H), 7.31 (dd, J = 8.0, 4.5 Hz, 1H); ^{13}C NMR (125 MHz, DMSO-d_6): δ 185.28, 149.32, 144.74, 138.54, 129.13, 118.32, 116.59, 116.42; LRMS (ESI): m/z calcd for $C_8H_7N_2O$ $[M+H]^+$: 147.06; Found: 147.10.

Compound 5b: 4-Chloro-1*H*-pyrrolo[2,3-b]pyridine-3-carbaldehyde

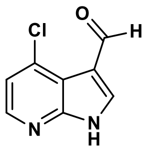

Following the general procedure described above with 4-chloro-1*H*-pyrrolo[2,3-b]pyridine (2.88 g, 18.84 mmol) provided **5b** in 62% yield (2.1 g) as orange colour solid. ^{1}H NMR (400 MHz, DMSO-d_6) δ 13.15 (s, 1H), 10.29 (s, 1H), 8.50 (s, 1H), 8.31 (d, *J* = 5.2 Hz, 1H), 7.42 (d, *J* = 5.2 Hz, 1H); ^{13}C NMR (125 MHz, DMSO-d_6): δ 184.04, 150.24, 144.71, 135.73, 135.05, 118.77, 116.29, 115.73; LRMS (ESI): *m/z* calcd for $C_8H_6ClN_2O$ [M+H]$^+$: 181.02; Found: 181.00.

Compound 5c: 1*H*-Pyrrolo[2,3-c]pyridine-3-carbaldehyde

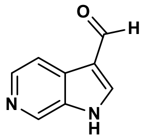

Following the general procedure described above with 6-azaindole (0.9 g, 7.26 mmol) provided **5c** in 78% yield (0.87 g) as off-white solid. ^{1}H NMR (400 MHz, DMSO- d_6): δ 12.57 (s, 1H), 10.02 (s, 1H), 8.89 (d, *J* = 1.2 Hz, 1H), 8.51 (s, 1H), 8.34 (d, *J* = 5.2 Hz, 1H), 8.00 (dd, *J* = 5.6, 1.2 Hz, 1H); ^{13}C NMR (100 MHz, DMSO-d_6): δ 185.39, 141.11, 140.61, 135.38, 134.04, 128.92, 117.35, 115.16; LRMS (ESI): *m/z* calcd for $C_8H_7N_2O$ [M+H]$^+$: 147.06; Found: 147.10.

Compound 5d: 1*H*-Pyrrolo[3,2-c]pyridine-3-carbaldehyde

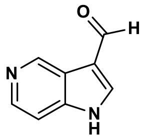

Following the general procedure described above with 5-azaindole (2 g, 16.93 mmol) provided **5d** in 55% yield (1.36 g) as white solid. ^{1}H NMR (400 MHz, DMSO- d_6) δ 12.43 (s, 1H), 9.99 (s, 1H), 9.28 (d, *J* = 1.2 Hz, 1H), 8.43 (s, 1H), 8.35 (d, *J* = 5.6 Hz, 1H), 7.53 (dd, *J* = 5.6, 1.2 Hz, 1H); ^{13}C NMR (100 MHz, DMSO-d_6): δ 185.19, 143.39, 142.51, 140.86, 139.36, 120.88, 117.61, 107.85; LRMS (ESI): *m/z* calcd for $C_8H_7N_2O$ [M+H]$^+$: 147.06; Found: 147.10.

Compound 5e: 1*H*-Pyrrolo[3,2-b]pyridine-3-carbaldehyde

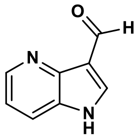

Following the general procedure described above with 4-Azaindole (0.9 g, 7.26 mmol) provided **5e** in 74% yield (0.82 g) as off-white solid. ^{1}H NMR (400 MHz, DMSO-d_6): δ 12.35 (brs, 1H), 10.19 (s, 1H), 8.51 (dd, *J* = 4.8, 1.2 Hz, 1H), 8.44 (s, 1H), 7.92 (dd, *J* = 8.4, 1.6 Hz, 1H), 7.28 (dd, *J* = 8.4, 4.4 Hz, 1H); ^{13}C NMR (100 MHz, DMSO-d_6): δ 184.02, 144.86, 143.53, 136.66, 129.34, 120.01, 118.21, 116.83; LRMS (ESI): *m/z* calcd for $C_8H_7N_2O$ [M+H]$^+$: 147.06; Found: 147.10.

Supplementary Figure 9. Formylation of indole substrates

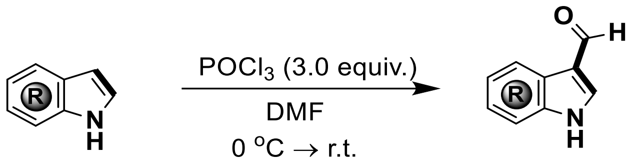

General Procedure: Substituted 3-formylindoles were synthesized using the general procedure reported in the literature[5]. To a stirred solution of an appropriate indole derivative in anhydrous dimethylformamide (DMF, 3.0–4.0 mL/g of indole) under argon atmosphere was slowly added phosphorous(V) oxychloride at 0 °C. The reaction mixture was brought to r.t. and stirring was continued for 45 min to 2 h. After completion of the reaction monitored by TLC and LC/MS, the reaction mixture was slowly poured into the cold aqueous saturated $NaHCO_3$ solution and stirred for 15–30 min. The solid precipitated upon neutralization was filtered and the residue was washed with water followed by hot hexane to afford the desired product. When precipitation was not observed especially with the small scale reaction, the crude compound was extracted into EtOAc and purified by silica-gel flash column chromatography. Acetal formation and product degradation was observed when MeOH/DCM mixture was used as an eluent for silica-gel flash column chromatography.

Compound 5j: 7-Methoxy-1*H*-indole-3-carbaldehyde

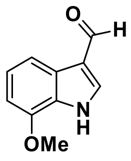

Following the general procedure described above with 7-methoxyindole (0.5 g, 3.40 mmol) provided **5j** in 99% yield (0.595 g) as pale yellow solid. ^1H NMR (400 MHz, $CDCl_3$): δ 10.04 (s, 1H), 9.27 (brs, 1H), 7.88 (dd, *J* = 8.0, 0.8 Hz, 1H), 7.78 (d, *J* = 2.0 Hz, 1H), 7.26–7.20 (m, 1H), 6.76 (d, *J* = 8.0 Hz, 1H), 3.95 (s, 3H); ^{13}C NMR (100 MHz, $CDCl_3$): δ 185.51, 146.13, 134.77, 127.32, 125.90, 123.77, 120.14, 114.33, 104.36, 55.57; LRMS (ESI): *m/z* calcd for $C_{10}H_{10}NO_2$ $[M+H]^+$: 176.07; Found: 176.10.

Compound 5l: 5-Nitro-1*H*-indole-3-carbaldehyde

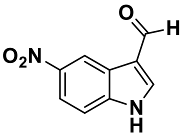

Following the general procedure described above with 5-nitroindole (1 g, 6.17 mmol) provided **5l** in 90% yield (1.05 g) as yellow colour solid. ^{1}H NMR (500 MHz, DMSO-*d$_6$*): δ 12.68 (s, 1H), 10.03 (s, 1H), 9.02–8.88 (m, 1H), 8.58 (s, 1H), 8.16 (dd, *J* = 9.0, 2.5 Hz, 1H), 7.72 (dd, *J* = 9.0, 0.5 Hz, 1H); ^{13}C NMR (125 MHz, DMSO-*d$_6$*): δ 185.58, 142.87, 141.67, 140.28, 123.58, 119.07, 118.82, 117.08, 113.34; LRMS (ESI): *m/z* calcd for $C_9H_5N_2O_3$ $[M+H]^-$: 189.03; Found: 189.05.

Compound 5o: 4-Fluoro-1*H*-indole-3-carbaldehyde

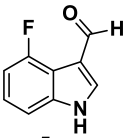

5o

Following the general procedure described above with 4-fluoroindole (0.33 g, 2.44 mmol) provided **5o** in 93% yield (0.37 g) as off-white solid. ^{1}H NMR (400 MHz, DMSO-*d$_6$*): δ 12.48 (s, 1H), 10.02 (d, *J* = 3.2 Hz, 1H), 8.31 (s, 1H), 7.37 (d, *J* = 8.4 Hz, 1H), 7.25 (td, *J* = 8.0, 4.8 Hz, 1H), 7.06–6.97 (m, 1H); ^{13}C NMR (100 MHz, DMSO-*d$_6$*): δ 183.64 (t, *J* = 1.8 Hz), 156.00 (d, *J* = 246.4 Hz), 139.74 (d, *J* =11.7 Hz), 136.13, 123.96 (d, *J* = 7.3 Hz), 116.94 (d, *J* = 5.7 Hz), 112.87 (d, *J* = 22.2 Hz), 109.10 (d, *J* = 3.6 Hz), 107.28 (d, *J* = 19.5 Hz); ^{19}F NMR (376 MHz, DMSO-*d$_6$*): δ –113.11; LRMS (ESI): *m/z* calcd for C_9H_7FNO $[M+H]^+$: 164.05; Found: 164.10.

Compound 5r: 7-Fluoro-1*H*-indole-3-carbaldehyde

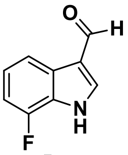

5r

Following the general procedure described above with 7-fluoroindole (0.25 g, 1.85 mmol) provided **5r** in 96% yield (0.29 g) as off-white solid. ^{1}H NMR (500 MHz, DMSO-*d$_6$*): δ 12.70 (s, 1H), 9.98 (s, 1H), 8.38 (s, 1H), 7.92 (d, *J* = 8.0 Hz, 1H), 7.21 (td, *J* = 8.0, 5.0 Hz, 1H), 7.12 (dd, *J* = 11.5, 8.0 Hz, 1H); ^{13}C NMR (100 MHz, DMSO-*d$_6$*): δ 185.28, 149.16 (d, *J* = 244.1 Hz), 139.01, 127.86 (d, *J* = 4.8 Hz), 124.92 (d, *J* = 13.0 Hz), 122.91, (d, *J* = 6.1 Hz), 118.88 (d, *J* = 1.7 Hz), 117.06 (d, *J* = 3.6 Hz), 108.56 (d, *J* = 15.8 Hz); ^{19}F NMR (376 MHz, DMSO-*d$_6$*): δ –131.91 (dd, *J* = 10.9, 5.3 Hz); LRMS (ESI): *m/z* calcd for C_9H_7FNO $[M+H]^+$: 164.05; Found: 164.10.

Compound 5w: 4-(Trifluoromethyl)-1*H*-indole-3-carbaldehyde

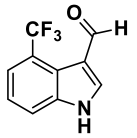

5w

Following the general procedure described above with 4-trifluoroindole (0.185 g, 1 mmol) provided **5w** in 84% yield (0.18 g) as light green solid. ^{1}H NMR (400 MHz, $CDCl_3$): δ 10.35 (s, 1H), 10.20 (s, 1H), 8.22 (d, *J* = 3.2 Hz, 1H), 7.72 (d, *J* = 8.0 Hz, 1H), 7.65 (d, *J* = 7.6 Hz, 1H), 7.37 (t, *J* = 8.0 Hz, 1H); ^{13}C NMR (100 MHz, $CDCl_3$): δ 187.96, (m), 137.90, 133.05, 128.94 (q, *J* = 269.8 Hz), 122.78, 122.65 (q, *J* = 32.7 Hz), 121.04 (q, *J* = 2.2 Hz), 120.67 (q, *J* = 5.8 Hz), 118.18, 116.75; ^{19}F NMR (376 MHz, $CDCl_3$): δ –61.73; LRMS (ESI): *m/z* calcd for $C_{10}H_7F_3NO$ $[M+H]^+$: 214.05; Found: 214.05.

Compound 5x: 5-(Trifluoromethyl)-1*H*-indole-3-carbaldehyde

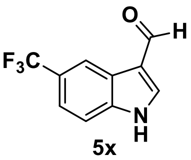

5x

Following the general procedure described above with 5-trifluoroindole (0.1 g, 0.54 mmol) provided **5x** in quantitative yield (0.134 g) as brown solid. ^{1}H NMR (500 MHz, DMSO-d_6): δ 12.51 (s, 1H), 10.01 (s, 1H), 8.51 (s, 1H), 8.41 (dd, *J* = 2.0, 1.0 Hz, 1H), 7.78–7.70 (m, 1H), 7.59 (dd, *J* = 8.5, 2.0 Hz, 1H); ^{13}C NMR (100 MHz, DMSO-d_6): δ 185.49, 140.25, 138.71, 125.16 (q, *J* = 270.3 Hz), 123.65, 122.91 (q, *J* = 31.1 Hz), 120.01 (q, *J* = 3.6 Hz), 118.33, 117.99 (q, *J* = 4.4 Hz), 113.48; ^{19}F NMR (376 MHz, DMSO-d_6): δ –59.22; LRMS (ESI): *m/z* calcd for $C_{10}H_7F_3NO$ $[M+H]^+$: 214.05; Found: 214.10.

Compound 5y: 6-(Trifluoromethyl)-1*H*-indole-3-carbaldehyde

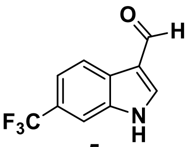

5y

Following the general procedure described above with 6-trifluoroindole (1 g, 5.40 mmol) provided **5y** in 98% yield (1.13 g) as orange solid. ^{1}H NMR (500 MHz, DMSO-d_6): 12.48 (s, 1H), 10.01 (s, 1H), 8.53 (d, *J* = 3.0 Hz, 1H), 8.29 (d, *J* = 8.5 Hz, 1H), 7.93–7.82 (m, 1H), 7.54 (dd, *J* = 8.5, 1.5 Hz, 1H); ^{13}C NMR (100 MHz, DMSO-d_6): δ 185.32, 140.64, 136.13, 126.90, 124.98 (q, *J* = 270.2 Hz), 124.02 (q, *J* = 31.3 Hz), 121.65, 118.46 (q, *J* = 3.4 Hz), 118.06, 109.92 (q, *J* = 4.4 Hz); ^{19}F NMR (376 MHz, DMSO-d_6) δ –59.62 (d, *J* = 3.0 Hz); LRMS (ESI): *m/z* calcd for $C_{10}H_7F_3NO$ $[M+H]^+$: 214.05; Found: 214.10.

Compound 5ad: 5-Hydroxy-1*H*-indole-3-carbaldehyde

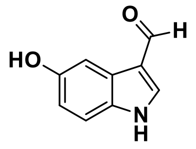

Following the general procedure described above with 5-hydroxyindole (0.3 g, 2.25 mmol) provided **5ad** in 83% yield (0.3 g) as pale brown colour solid. ^{1}H NMR (500 MHz, DMSO-d_6) δ 11.91 (s, 1H), 9.86 (s, 1H), 9.11 (s, 1H), 8.15 (s, 1H), 7.51 (d, *J* = 2.5 Hz, 1H), 7.32 (dd, *J* = 8.5, 0.5 Hz, 1H), 6.76 (dd, *J* = 9.0, 2.5 Hz, 1H); ^{13}C NMR (125 MHz, DMSO-d_6): δ 184.60, 153.39, 138.31, 131.16, 125.21, 117.81, 113.29, 112.88, 105.31; LRMS (ESI): *m/z* calcd for $C_9H_8NO_2$ $[M+H]^+$: 162.06; Found: 162.05.

Compound 5ae: 5-Ethoxy-1*H*-indole-3-carbaldehyde

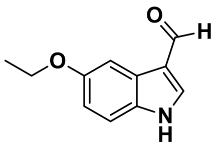

Following the general procedure described above with 5-ethoxy-1*H*-indole (0.3 g, 1.86 mmol) provided **5ae** in 63% yield (0.22 g) as off-white solid. ^{1}H NMR (500 MHz, DMSO-d_6): δ 12.02 (s, 1H), 9.90 (s, 1H), 8.21 (s, 1H), 7.58 (d, *J* = 2.5 Hz, 1H), 7.40 (dd, *J* = 8.5, 0.5 Hz, 1H), 6.88 (dd, *J* = 9.0, 3.0 Hz, 1H), 4.04 (q, *J* = 7.0 Hz, 2H), 1.36 (t, *J* = 7.0 Hz, 3H); ^{13}C NMR (125 MHz, DMSO-d_6): δ 184.82, 154.86, 138.41, 131.77, 124.93, 118.03, 113.70, 113.18, 103.28, 63.29, 14.83; LRMS (ESI): *m/z* calcd for $C_{11}H_{12}NO_2$ $[M+H]^+$: 190.09; Found: 190.10.

Compound 5af: Ethyl 3-formyl-1*H*-indole-5-carboxylate

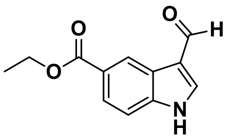

Ethyl 1*H*-indole-5-carboxylate (0.32 g, 1.69 mmol) was prepared by the following procedure reported in the literature[6] and used in the above described procedure to provide **5af** in 88% yield (0.324 g) as light yellow colour solid. ^{1}H NMR (400 MHz, DMSO-d_6) δ 12.43 (s, 1H), 9.98 (s, 1H), 8.89–8.65 (m, 1H), 8.43 (s, 1H), 7.88 (dd, *J* = 8.8, 1.6 Hz, 1H), 7.61 (dd, *J* = 8.8, 0.8 Hz, 1H), 4.33 (q, *J* = 7.2 Hz, 2H), 1.34 (t, *J* = 7.2 Hz, 3H); ^{13}C NMR (100 MHz, DMSO-d_6): δ 185.31, 166.30, 140.17, 139.62, 124.41, 123.77, 123.68, 122.93, 118.66, 112.50, 60.45, 14.28; LRMS (ESI): *m/z* calcd for $C_{12}H_{12}NO_3$ $[M+H]^+$: 218.08; Found: 218.10.

Compound 5ag′: Butyl 1*H*-indole-5-carboxylate

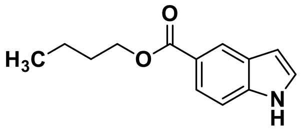

Butyl 1*H*-indole-5-carboxylate **5ag′** (1g, 74% yield, off-white solid) was prepared by the following procedure reported in the literature[6], from indole-5-carboxylicacid (1g, 6.21 mmol). ^{1}H NMR (400 MHz, DMSO-d_6): δ 11.48 (s, 1H), 8.28 (d, *J* = 1.6 Hz, 1H), 7.74 (dd, *J* = 8.4, 1.6 Hz, 1H), 7.54–7.43 (m, 2H), 6.61 (t, *J* = 2.4 Hz, 1H), 4.26 (t, *J* = 6.4 Hz, 2H), 1.76–1.64 (m, 2H), 1.51–1.38 (m, 2H), 0.95 (t, *J* = 7.2 Hz, 3H); ^{13}C NMR (100 MHz, DMSO-d_6): δ 166.84, 138.45, 127.24, 127.13, 122.63, 121.90, 120.63, 111.31, 102.57, 63.74, 30.42, 18.84, 13.64; LRMS (ESI): *m/z* calcd for $C_{13}H_{16}NO_2$ $[M+H]^+$: 218.12; Found: 218.10.

Compound 5ag: Butyl 3-formyl-1*H*-indole-5-carboxylate

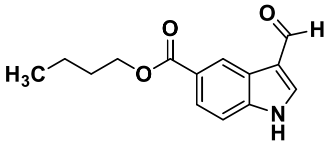

Butyl 1*H*-indole-5-carboxylate **5ag′** (0.5 g, 2.3 mmol) was used in the above described procedure to provide **5ag** in 80% yield (0.45 g) as off-white solid. ^{1}H NMR (500 MHz, DMSO-d_6): δ 12.45 (s, 1H), 9.99 (s, 1H), 8.77 (dd, *J* = 1.5, 0.5 Hz, 1H), 8.45 (s, 1H), 7.89 (dd, *J* = 8.5, 1.5 Hz, 1H), 7.62 (dd, *J* = 8.5, 0.5 Hz, 1H), 4.30 (t, *J* = 6.5 Hz, 2H), 1.76–1.69 (m, 2H), 1.49–1.40 (m, 2H), 0.96 (t, *J* = 7.5 Hz, 3H); ^{13}C NMR (125 MHz, DMSO-d_6): δ 185.36, 166.37, 140.26, 139.66, 124.42, 123.77, 123.73, 122.95, 118.67, 112.56, 64.16, 30.38, 18.82, 13.66; LRMS (ESI): *m/z* calcd for $C_{14}H_{16}NO_3$ $[M+H]^+$: 246.11; Found: 246.15.

Supplementary Figure 10. Synthesis of arylsulfonyl-protected (aza)indole-3-carboxaldehydes

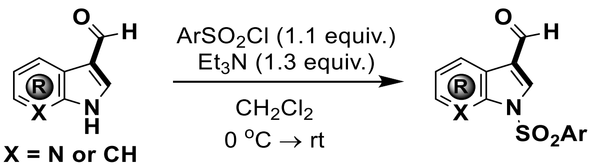

General Procedure: A round bottom flask placed under the ice bath, equipped with a magnetic bar, was charged with (aza)indole-3-carbaldehyde derivative, Et_3N (1.3 equiv.) and DCM. To this cold

suspension was slowly added appropriate arylsulfonyl chloride (1.1 equiv.), and the reaction mixture was stirred at r.t. for 3–4 h. The progress of reaction was monitored by TLC and LC/MS. After completion of the reaction, the solvent was evaporated to provide a light brown solid residue which was washed with hexane and filtered through a sintered funnel. The solid residue was then given warm water wash followed by hexane, and dried under the reduced pressure to obtain the desired *N*-sulfonyl protected product. About 10 mL of CH_2Cl_2 was used for 1.0 g of (aza)indole-3- carboxaldehydes and about 100 mL of hexane and water used for washing the residue. When a gummy solid was obtained after evaporation of CH_2Cl_2 *in vacuo*, the crude compound was purified by silica-gel flash column chromatography using EtOAc/hexane mixture as an eluent.

Compound 1a: 1-(Phenylsulfonyl)-1*H*-pyrrolo[2,3-*b*]pyridine-3-carbaldehyde

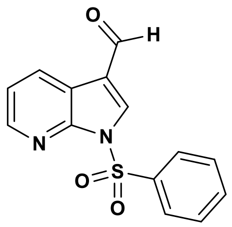

Following the general procedure described above with 1*H*-pyrrolo[2,3-*b*]pyridine-3-carbaldehyde **5a** (0.4 g, 2.74 mmol) provided **1a** in 91% yield (0.71 g) as off-white solid. ^{1}H NMR (400 MHz, $CDCl_3$): δ 10.05 (s, 1H), 8.54–8.46 (m, 2H), 8.41 (s, 1H), 8.32–8.26 (m, 2H), 7.65 (t, *J* = 7.6 Hz, 1H), 7.55 (t, *J* = 7.6 Hz, 2H), 7.32 (dd, *J* = 7.6, 5.2 Hz, 1H); ^{13}C NMR (100 MHz, $CDCl_3$): δ 185.27, 147.47, 146.80, 137.29, 135.82, 135.00, 131.44, 129.40 (2C), 128.68 (2C), 120.83, 119.57, 119.07; LRMS (ESI): *m/z* calcd for $C_{14}H_{11}N_2O_3S$ $[M+H]^+$: 287.05; Found: 287.10.

Compound 1b: 1-((4-Methoxyphenyl)sulfonyl)-1*H*-pyrrolo[2,3-*b*]pyridine-3-carbaldehyde

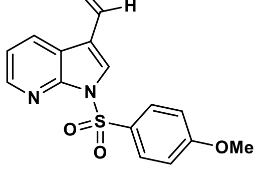

Following the general procedure described above with 1*H*-pyrrolo[2,3-*b*]pyridine-3-carbaldehyde **5a** (0.146 g, 1 mmol) provided **1b** in 61% yield (0.192 g) as off-white solid. ^{1}H NMR (500 MHz, DMSO-*d*$_6$): δ 10.09 (s, 1H), 8.99 (s, 1H), 8.51 (dd, *J* = 5.0, 1.5 Hz, 1H), 8.46 (dd, *J* = 8.0, 1.5 Hz, 1H), 8.26–8.12 (m, 2H), 7.46 (dd, *J* = 8.0, 4.5 Hz, 1H), 7.26–7.13 (m, 2H), 3.85 (s, 3H); ^{13}C NMR (125 MHz, DMSO-*d*$_6$): δ 186.79, 164.46, 146.56, 146.26, 137.83, 130.77 (2C), 130.63, 127.49, 120.94, 118.45, 118.26, 114.98 (2C), 55.97; LRMS (ESI) *m/z* calcd for $C_{15}H_{13}N_2O_4S$ $[M+H]^+$: 317.06; Found: 317.05.

Compound 1c: 1-((4-Nitrophenyl)sulfonyl)-1*H*-pyrrolo[2,3-*b*]pyridine-3-carbaldehyde

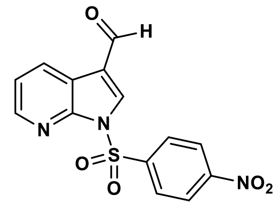

Following the general procedure described above with 1*H*-pyrrolo[2,3-*b*]pyridine-3-carbaldehyde **5a** (0.5 g, 3.42 mmol) provided **1c** in 86% yield (0.978 g) as yellow colour solid. ^{1}H NMR (400 MHz, $CDCl_3$): δ 10.07 (s, 1H), 8.58–8.48 (m, 4H), 8.42–8.32 (m, 3H), 7.37 (dd, *J* = 8.0, 4.8 Hz, 1H); ^{13}C NMR (100 MHz, $CDCl_3$): δ 185.06, 151.41, 147.36, 147.06, 142.56, 135.13, 131.90, 130.37 (2C), 124.58 (2C), 121.35, 120.33, 119.22; LRMS (ESI): *m/z* calcd for $C_{14}H_{10}N_3O_5S$ $[M+H]^+$: 332.03; Found: 332.00.

Compound 1n: 1-(Phenylsulfonyl)-1*H*-indole-3-carbaldehyde

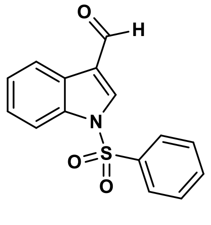

Following the general procedure described above with 1*H*-indole-3-carbaldehyde **5f** (1.2 g, 8.27 mmol) provided **1n** in 98% yield (2.3 g) as pale brown colour solid. ^{1}H NMR (500 MHz, $CDCl_3$): δ 10.10 (s, 1H), 8.25 (d, *J* = 9.0 Hz, 1H), 8.24 (s, 1H), 8.00–7.93 (m, 3H), 7.61 (t, *J* = 8.0 Hz, 1H), 7.51 (t, *J* = 8.0 Hz, 2H), 7.41 (t, *J* = 8.0 Hz, 1H), 7.36 (t, *J* = 7.5 Hz, 1H); ^{13}C NMR (125MHz, $CDCl_3$): δ 185.46, 137.45, 136.28, 135.34, 134.86, 129.83 (2C), 127.26 (2C), 126.51, 126.39, 125.25, 122.74, 122.61, 113.33; LRMS (ESI): *m/z* calcd for $C_{15}H_{12}NO_3S$ $[M+H]^+$: 286.05; Found: 286.05.

Compound 1q: 4-Methoxy-1-(phenylsulfonyl)-1*H*-indole-3-carbaldehyde

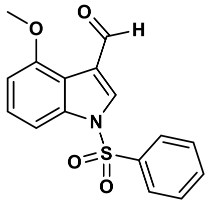

Following the general procedure described above with 4-methoxy-1*H*-indole-3-carbaldehyde **5g** (0.175 g, 1 mmol) provided **1q** in 79% yield (0.25 g) as grey colour solid. ^{1}H NMR (500 MHz, DMSO-d_6): δ 10.38 (s, 1H), 8.41 (s, 1H), 8.22–8.11 (m, 2H), 7.81–7.74 (m, 1H), 7.71–7.62 (m, 2H), 7.60 (d, *J* = 8.5 Hz, 1H), 7.40 (t, *J* = 8.0 Hz, 1H), 6.98 (d, *J* = 8.0 Hz, 1H), 3.93 (s, 3H); ^{13}C NMR (125 MHz, DMSO-d_6): δ 187.09, 153.98, 136.16, 135.44, 135.40, 130.15 (2C), 129.26, 127.37 (2C), 127.08, 121.84, 116.51, 106.01, 105.72, 55.80; LRMS (ESI): *m/z* calcd for $C_{16}H_{14}NO_4S$ $[M+H]^+$: 316.06; Found: 316.05.

Compound 1r: 5-Methoxy-1-(phenylsulfonyl)-1*H*-indole-3-carbaldehyde

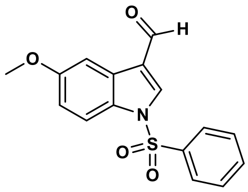

Following the general procedure described above with 5-methoxy-1*H*-indole-3-carbaldehyde **5h** (0.5 g, 2.85 mmol) provided **1r** in 94% yield (0.85 g) as light yellow colour solid. ^{1}H NMR (400 MHz, $CDCl_3$): δ 10.06 (s, 1H), 8.18 (s, 1H), 8.00–7.90 (m, 2H), 7.83 (d, *J* = 9.2 Hz, 1H), 7.71 (d, *J* = 2.4 Hz, 1H), 7.64–7.57 (m, 1H), 7.50 (t, *J* = 8.0 Hz, 2H), 7.01 (dd, *J* = 9.2, 2.4 Hz, 1H), 3.84 (s, 3H); ^{13}C NMR (100 MHz, $CDCl_3$): δ 185.58, 157.91, 137.50, 136.69, 134.79, 129.83, 129.79 (2C), 127.46, 127.17 (2C), 122.54, 116.33, 114.21, 104.21, 55.85; LRMS (ESI): *m/z* calcd for $C_{16}H_{14}NO_4S$ $[M+H]^+$: 316.06; Found: 316.05.

Compound 1s: 6-Methoxy-1-(phenylsulfonyl)-1*H*-indole-3-carbaldehyde

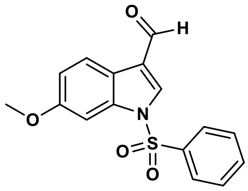

Following the general procedure described above with 6-methoxy-1*H*-indole-3-carbaldehyde **5i** (0.47 g, 2.68 mmol) provided **1s** in 96% yield (0.817 g) as brown colour solid. ^{1}H NMR (400 MHz, $CDCl_3$): δ 10.04 (s, 1H), 8.12 (s, 1H) 8.11 (d, *J* = 8.0 Hz, 2H), 8.00–7.92 (m, 2H), 7.62 (t, *J* = 7.6 Hz, 1H), 7.52 (t, *J* = 7.6 Hz, 2H), 7.46 (d, *J* = 2.4 Hz, 1H), 6.98 (dd, *J* = 8.8, 2.4 Hz, 1H), 3.88 (s, 3H); ^{13}C NMR (100 MHz, $CDCl_3$): δ 185.45, 159.16, 137.56, 136.58, 135.31, 134.85, 129.85 (2C), 127.20 (2C), 123.32, 122.85, 120.01, 113.97, 97.80, 55.92; LRMS (ESI): *m/z* calcd for $C_{16}H_{14}NO_4S$ $[M+H]^+$: 316.06; Found: 316.00.

Compound 1t: 7-Methoxy-1-(phenylsulfonyl)-1*H*-indole-3-carbaldehyde

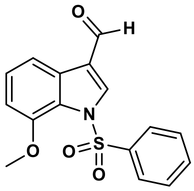

Following the general procedure described above with 7-methoxy-1*H*-indole-3-carbaldehyde **5j** (0.175 g, 1 mmol) provided **1t** in 30% yield (0.095 g) as white solid. ^{1}H NMR (500 MHz, DMSO-d_6): δ 10.14 (s, 1H), 8.93 (s, 1H), 8.03–7.93 (m, 2H), 7.82–7.72 (m, 2H), 7.67 (t, *J* = 8.0 Hz, 2H), 7.30 (t, *J* = 8.0 Hz, 1H), 6.94 (d, *J* = 8.0 Hz, 1H), 3.64 (s, 3H), 3.57 (brs, 1H); ^{13}C NMR (125 MHz, DMSO-d_6): δ 187.09, 153.98, 136.16, 135.45, 135.41, 130.15 (2C), 129.27, 127.37 (2C), 127.08, 121.84, 116.51, 106.02, 105.73, 55.81; LRMS (ESI): *m/z* calcd for $C_{16}H_{14}NO_4S$ $[M+H]^+$: 316.06; Found: 316.00.

Compound 1u: 4-Nitro-1-(phenylsulfonyl)-1*H*-indole-3-carbaldehyde

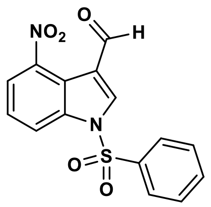

Following the general procedure described above with 4-nitro-1*H*-indole-3-carbaldehyde **5k** (0.190 g, 1 mmol) provided **1u** in 89% yield (0.295 g) as yellow solid. ^{1}H NMR (500 MHz, DMSO-d_6) δ 10.08 (s, 1H), 9.03 (s, 1H), 8.41 (dd, *J* = 8.5, 1.0 Hz, 1H), 8.27–8.21 (m, 2H), 8.02 (dd, *J* = 8.0, 1.0 Hz, 1H), 7.86–7.79 (m, 1H), 7.74–7.66 (m, 3H); ^{13}C NMR (125 MHz, DMSO-d_6) δ 185.63, 143.38, 138.58, 135.90, 135.78, 135.72, 130.35 (2C), 127.56 (2C), 126.40, 120.43, 120.17, 118.37, 117.67; LRMS (ESI): *m/z* calcd for $C_{15}H_{11}N_2O_5S$ [M+H]$^+$: 331.04; Found: 331.00.

Compound 1v: 5-Nitro-1-(phenylsulfonyl)-1*H*-indole-3-carbaldehyde

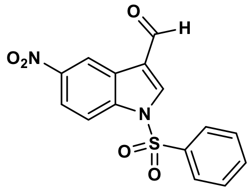

Following the general procedure described above with 5-nitro-1*H*-indole-3-carbaldehyde **5l** (0.5 g, 2.63 mmol) provided **1v** in 98% yield (0.85 g) as off-white solid. ^{1}H NMR (500 MHz, DMSO-d_6): δ 10.13 (s, 1H), 9.17 (s, 1H), 8.88 (d, *J* = 2.5 Hz, 1H), 8.31 (dd, *J* = 9.5, 2.5 Hz, 1H), 8.25–8.19 (m, 3H), 7.83 (t, *J* = 7.5 Hz, 1H), 7.71 (t, *J* = 7.5 Hz, 2H); ^{13}C NMR (125 MHz, DMSO-d_6): δ 186.65, 144.84, 140.82, 137.12, 135.93, 135.79, 130.40 (2C), 127.41 (2C), 125.80, 121.41, 121.38, 117.51, 114.17; LRMS (ESI): *m/z* calcd for $C_{15}H_{11}N_2O_5S$ [M+H]$^+$: 331.04; Found: 330.95.

Compound 1w: 6-Nitro-1-(phenylsulfonyl)-1*H*-indole-3-carbaldehyde

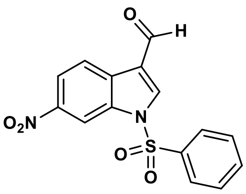

Following the general procedure described above with 6-nitro-1*H*-indole-3-carbaldehyde **5m** (0.5 g, 2.63 mmol) provided **1w** in 81% yield (0.702 g) as yellow solid. ^{1}H NMR (500 MHz, DMSO-d_6): δ 10.12 (s, 1H), 9.27 (s, 1H), 8.74 (d, *J* = 2.0 Hz, 1H), 8.32 (dd, *J* = 9.0. 0.5 Hz, 1H), 8.27 (dd, *J* = 9.0, 2.0 Hz, 1H), 8.21–8.17 (m, 2H), 7.85–7.81 (m, 1H), 7.74–7.69 (m, 2H); ^{13}C NMR (125 MHz, DMSO-d_6): δ 186.51, 145.45, 142.39, 135.92, 133.36, 130.56, 130.49 (2C), 127.30 (2C), 122.59, 121.06, 120.37, 109.02; LRMS (ESI): *m/z* calcd for $C_{15}H_{10}N_2O_5S$ [M+H]$^+$: 331.04; Found: 330.95.

Compound 1x: 4-Bromo-1-(phenylsulfonyl)-1*H*-indole-3-carbaldehyde

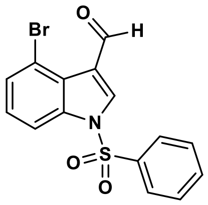

Following the general procedure described above with 4-bromo-1*H*-indole-3-carbaldehyde **5s** (0.2 g, 0.89 mmol) provided **1x** in 88% yield (0.286 g) as reddish brown colour solid. ^{1}H NMR (500 MHz, $CDCl_3$): δ 10.92 (s, 1H), 8.42 (s, 1H), 8.06–7.88 (m, 3H), 7.64 (t, *J* = 7.5 Hz, 1H), 7.57–7.43 (m, 3H), 7.29–7.20 (m, 1H); ^{13}C NMR (125 MHz, $CDCl_3$): δ 186.32, 137.16, 136.27, 135.11, 132.10, 129.93 (2C), 129.09, 127.38 (2C), 127.13, 126.40, 122.21, 114.06, 112.96; LRMS (ESI): *m/z* calcd for $C_{15}H_{11}BrNO_3S$ $[M+H]^+$: 363.96; Found: 363.85.

Compound 1y: 5-Bromo-1-(phenylsulfonyl)-1*H*-indole-3-carbaldehyde

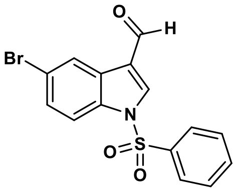

Following the general procedure described above with 5-bromo-1*H*-indole-3-carbaldehyde **5t** (0.5 g, 2.23 mmol) provided **1y** in 90% yield (0.733 g) as off-white solid. ^{1}H NMR (500 MHz, DMSO-d_6): δ 10.06 (s, 1H), 8.97 (s, 1H), 8.24 (d, *J* = 2.0 Hz, 1H), 8.16–8.10 (m, 2H), 7.96 (d, *J* = 9.0 Hz, 1H), 7.83–7.76 (m, 1H), 7.68 (t, *J* = 8.0 Hz, 2H), 7.63 (dd, *J* = 9.0, 2.0 Hz, 1H); ^{13}C NMR (125 MHz, DMSO-d_6): δ 186.69, 139.29, 136.01, 135.66, 133.19, 130.30 (2C), 129.00, 127.56, 127.24 (2C), 124.03, 120.63, 117.96, 115.26; LRMS (ESI): *m/z* calcd for $C_{15}H_{11}BrNO_3S$ $[M+H]^+$: 363.96; Found: 363.95.

Compound 1z: 6-Bromo-1-(phenylsulfonyl)-1*H*-indole-3-carbaldehyde

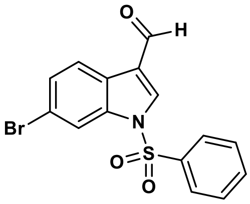

Following the general procedure described above with 6-bromo-1*H*-indole-3-carbaldehyde **5u** (0.3 g, 1.34 mmol) provided **1z** in 90% yield (0.44 g) as light yellow colour solid. ^{1}H NMR (500 MHz, DMSO-d_6) δ 10.08 (s, 1H), 8.97 (s, 1H), 8.21–8.17 (m, 2H), 8.12 (d, *J* = 2.0 Hz, 1H), 8.07 (d, *J* = 8.5 Hz, 1H), 7.84–7.79 (m, 1H), 7.71 (t, *J* = 8.0 Hz, 2H), 7.60 (dd, *J* = 8.5, 2.0 Hz, 1H); ^{13}C NMR (125 MHz, DMSO-d_6): δ 186.60, 138.90, 136.07, 135.67, 135.03, 130.35 (2C), 128.37, 127.25 (2C), 124.83, 123.53, 121.28, 118.93, 115.65; LRMS (ESI) *m/z* calcd for $C_{15}H_{11}BrNO_3S$ $[M+H]^+$: 363.96; Found: 363.95.

Compound 1aj: *N*-(4-((3-Formyl-1*H*-pyrrolo[2,3-*b*]pyridin-1-yl)sulfonyl)phenyl)acetamide

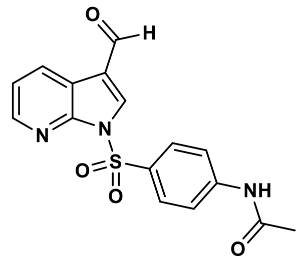

Following the general procedure described above with 1*H*-pyrrolo[2,3-*b*]pyridine-3-carbaldehyde **5a** (1 g, 6.84 mmol) provided **1aj** in 41% yield (0.964 g) as yellow colour solid. ^{1}H NMR (500 MHz, DMSO-d_6): δ 10.52 (s, 1H), 10.10 (s, 1H), 8.99 (s, 1H), 8.55–8.43 (m, 2H), 8.19 (d, *J* = 8.5 Hz, 2H), 7.86 (d, *J* = 9.0 Hz, 2H), 7.46 (dd, *J* = 7.5, 4.5 Hz, 1H), 2.10 (s, 3H); ^{13}C NMR (100 MHz, DMSO-d_6): δ 186.80, 169.33, 146.59, 146.26, 145.34, 137.77, 130.61, 129.81 (2C), 129.21, 120.93, 118.67 (2C), 118.52, 118.25, 24.16; LRMS (ESI): m/z calcd for $C_{16}H_{14}N_3O_4S$ [M+H]$^+$: 344.07; Found: 344.10.

Compound 1ak: *N*-(4-((3-Formyl-5-nitro-1*H*-indol-1-yl)sulfonyl)phenyl)acetamide

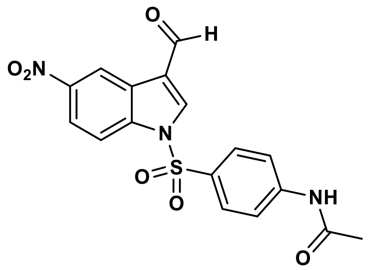

A round bottom flask placed under the ice bath, equipped with a magnetic bar, charged with 7-azaindole-3-carboxaldehyde in anhydrous DMF (4 mL), were added DMAP (19.2 mg, 0.16 mmol), and DIPEA (304.3 mg, 2.35 mmol) followed by 4-acetamidobenzenesulfonyl chloride (403.4 mg, 1.73 mmol). Then the reaction mixture was allowed to attain r.t. and stirred for 4 h. The progress of the reaction was monitored by TLC and LC/MS. After completion of the reaction, it was quenched by ice cold water (20 mL), the precipitate obtained was filtered, washed with warm water followed by hot hexane, and dried under the reduced pressure to afford the desired product **1ak** in 42% yield (0.26 g) as off-white solid. ^{1}H NMR (500 MHz, DMSO-d_6) δ 10.52 (s, 1H), 10.12 (s, 1H), 9.10 (s, 1H), 8.90 (d, *J* = 2.5 Hz, 1H), 8.31 (dd, *J* = 9.0, 2.5 Hz, 1H), 8.20 (d, *J* = 9.0 Hz, 1H), 8.17–8.11 (m, 2H), 7.86–7.79 (m, 2H), 2.06 (s, 3H); ^{13}C NMR (125 MHz, DMSO-d_6): δ 186.77, 169.48, 145.75, 144.80, 140.83, 137.09, 129.20 (2C), 128.37, 125.79, 121.33, 121.18, 119.16 (2C), 117.51, 114.20, 24.21; LRMS (ESI): m/z calcd for $C_{17}H_{14}N_3O_6S$ [M+H]$^+$: 388.06; Found: 388.10.

Supplementary Figure 11. Synthesis of benzyl-protected (aza)indole-3-carboxaldehydes

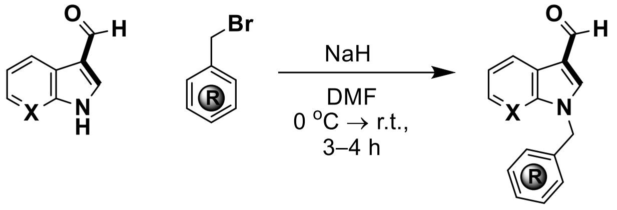

General procedure: A round bottom flask placed under the ice bath, equipped with a magnetic bar, charged with (aza)indole-3-carboxaldehyde in anhydrous DMF was added NaH (2 equiv., 60% suspension in paraffin oil) in a portion-wise under argon atmosphere. After complete addition of NaH, the reaction mixture was warmed to r.t. and stirred for 30–45 min. The reaction mixture was then cooled to 0 °C, an appropriate benzyl bromide derivative (1.1 equiv.) was added dropwise (in case of liquid), the reaction mixture was allowed to attain r.t. and stirred for 3–4 h. The progress of the reaction was monitored by TLC and LC/MS. After completion of the reaction, it was quenched by ice cold water (20 mL), the precipitate obtained was filtered, washed with warm water followed by hot hexane, and dried under the reduced pressure to afford the desired product. About 5.0 mL of anhydrous DMF was used for a 1 g of (aza)indole-3-carboxaldehyde. When benzyl bromide derivative is solid, it was added by dissolving in a minimum amount of dry DMF and the crude product obtained after filtration was purified by silica-gel flash column chromatography using EtOAc/hexane mixture as an eluent.

Compound 1d: 1-Benzyl-1*H*-pyrrolo[2,3-*b*]pyridine-3-carbaldehyde

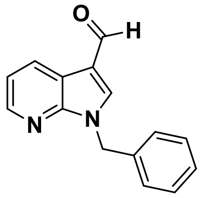

Following the general procedure described above with 1*H*-pyrrolo[2,3-*b*]pyridine-3-carbaldehyde **5a** (0.3 g, 2.05 mmol) provided **1d** in 70% yield (0.34 g) as off-white solid. ^{1}H NMR (400 MHz, $CDCl_3$): δ 9.93 (s, 1H), 8.57 (dd, J = 8.0, 2.0 Hz, 1H), 8.46 (dd, J = 4.8, 1.6 Hz, 1H), 7.79 (s, 1H), 7.39–7.25 (m, 6H), 5.54 (s, 2H); ^{13}C NMR (125 MHz, $CDCl_3$): δ 184.81, 148.69, 145.38, 137.95, 136.01, 130.71, 129.15 (2C), 128.46, 128.09 (2C), 119.19, 117.68, 116.88, 48.66; LRMS (ESI): *m/z* calcd for $C_{15}H_{13}N_2O$ [M+H]$^+$: 237.10; Found: 237.10.

Compound 1e: 1-(2-Bromobenzyl)-1*H*-pyrrolo[2,3-*b*]pyridine-3-carbaldehyde

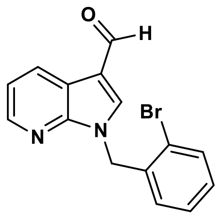

Following the general procedure described above with 1*H*-pyrrolo[2,3-*b*]pyridine-3-carbaldehyde **5a** (2 g, 13.68 mmol) provided **1e** in 73% yield (3.15 g) as white solid. ^{1}H NMR (400 MHz, $CDCl_3$) δ 9.96 (s, 1H), 8.59 (dd, *J* = 8.0, 1.6 Hz, 1H), 8.46 (dd, *J* = 4.8, 1.6 Hz, 1H), 7.87 (s, 1H), 7.64 (dd, *J* = 8.0, 1.6 Hz, 1H), 7.32–7.18 (m, 3H), 7.13 (dd, *J* = 8.0, 2.0 Hz, 1H), 5.65 (s, 2H); ^{13}C NMR (100 MHz, $CDCl_3$): δ 184.87, 148.76, 145.50, 138.02, 135.39, 133.38, 130.85, 130.25, 130.15, 128.22, 123.69, 119.33, 117.68, 117.09, 48.72; LRMS (ESI): *m/z* calcd for $C_{15}H_{12}BrN_2O$ $[M+H]^+$: 315.01; Found: 314.95.

Compound 1f: 1-(2-Iodobenzyl)-1*H*-pyrrolo[2,3-*b*]pyridine-3-carbaldehyde

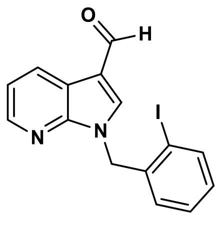

Following the general procedure described above with 1*H*-pyrrolo[2,3-*b*]pyridine-3-carbaldehyde **5a** (0.5 g, 3.42 mmol) provided **1f** in 86% yield (1.06 g) as light yellow colour solid. ^{1}H NMR (500 MHz, DMSO-d_6): δ 9.96 (s, 1H), 8.56–8.34 (m, 3H), 7.96 (d, *J* = 7.5 Hz, 1H), 7.44–7.27 (m, 2H), 7.09 (t, *J* = 7.5 Hz, 1H), 6.77 (d, *J* = 7.5 Hz, 1H), 5.57 (s, 2H); ^{13}C NMR (125 MHz, DMSO-d_6): δ 185.40, 148.01, 144.94, 140.45, 139.38, 138.57, 129.77, 129.73, 128.73, 128.16, 119.09, 116.84, 115.91, 98.36, 52.82; LRMS (ESI): *m/z* calcd for $C_{15}H_{12}IN_2O$ $[M+H]^+$: 363.00; Found: 362.95.

Compound 1g: 1-(4-Methoxybenzyl)-1*H*-pyrrolo[2,3-*b*]pyridine-3-carbaldehyde

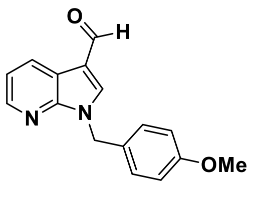

Following the general procedure described above with 1*H*-pyrrolo[2,3-*b*]pyridine-3-carbaldehyde **5a** (0.5 g, 3.42 mmol) provided **1g** in 77% yield (0.7 g) as off-white solid. ^{1}H NMR (400 MHz, $CDCl_3$): δ 9.90 (s, 1H), 8.55 (d, *J* = 7.6 Hz, 1H), 8.49–8.42 (m, 1H), 7.76 (s, 1H), 7.29–7.23 (m, 3H), 6.91–6.86 (m, 2H), 5.45 (s, 2H), 3.79 (s, 3H); ^{13}C NMR (125 MHz, $CDCl_3$): δ 184.65, 159.81, 148.72, 145.29, 137.71, 130.62, 129.73 (2C), 127.99, 119.11, 117.82, 116.82, 114.57 (2C), 55.42, 48.22; LRMS (ESI): *m/z* calcd for $C_{16}H_{15}N_2O_2$ $[M+H]^+$: 267.11; Found: 267.15.

Compound 1h: 1-(4-Chlorobenzyl)-1*H*-pyrrolo[2,3-*b*]pyridine-3-carbaldehyde

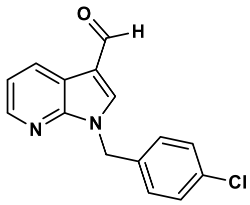

Following the general procedure described above with 1*H*-pyrrolo[2,3-*b*]pyridine-3-carbaldehyde (0.35 g, 2.39 mmol) provided **1h** in 65% yield (0.42 g) as off-white solid. ^{1}H NMR (500 MHz, $CDCl_3$): δ 9.94 (s, 1H), 8.57 (dd, *J* = 7.5, 1.5 Hz, 1H), 8.45 (dd, *J* = 4.5, 1.5 Hz, 1H), 7.80 (s, 1H), 7.33–7.26 (m, 3H), 7.23 (d, *J* = 8.0 Hz, 2H), 5.50 (s, 2H); ^{13}C NMR (125 MHz, $CDCl_3$): δ 184.76, 148.57, 145.50, 137.64, 134.62, 134.40, 130.80, 129.38 (2C), 129.33 (2C), 119.31, 117.67, 117.08, 48.05; LRMS (ESI): *m/z* calcd for $C_{15}H_{12}ClN_2O$ $[M+H]^+$: 271.06; Found: 271.10.

Compound 1o: 1-Benzyl-1*H*-indole-3-carbaldehyde

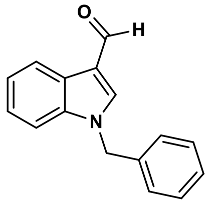

Following the general procedure described above with 1*H*-indole-3-carbaldehyde **5f** (2 g, 13.8 mmol) provided **1o** in 47% yield (1.51 g) as pale brown solid. ^{1}H NMR (500 MHz, $CDCl_3$): δ 9.97 (s, 1H), 8.32 (d, *J* = 7.5 Hz, 1H), 7.68 (s, 1H), 7.40–7.25 (m, 6H), 7.21–7.08 (m, 2H), 5.33 (s, 2H); ^{13}C NMR (125 MHz, $CDCl_3$): δ 184.72, 138.63, 137.57, 135.42, 129.22 (2C), 128.49, 127.32 (2C), 125.60, 124.26, 123.16, 122.26, 118.59, 110.49, 51.00; LRMS (ESI): *m/z* calcd for $C_{16}H_{14}NO$ $[M+H]^+$: 236.11; Found: 236.10.

Compound 1m: 1-Methyl-1*H*-pyrrolo[2,3-*b*]pyridine-3-carbaldehyde

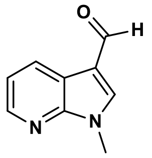

Following the same procedure using methyl iodide (1.1 equiv.) and 410 mg of 7-azaindole-3-carboxaldehyde **5a**, provided **1m** in 80% yield (360 mg) as off-white solid. ^{1}H NMR (500 MHz, $CDCl_3$): δ 9.96 (s, 1H), 8.54 (d, *J* = 8.0 Hz, 1H), 8.43 (d, *J* = 4.5 Hz, 1H), 7.84 (d, *J* = 2.5 Hz, 1H), 7.26 (dt, *J* = 7.5, 3.5 Hz, 1H), 3.96 (s, 3H); ^{13}C NMR (125 MHz, $CDCl_3$): δ 184.59, 148.78, 145.18, 139.09, 130.53, 118.89, 117.64, 116.32, 32.13; LRMS (ESI): *m/z* calcd for $C_9H_9N_2O$ $[M+H]^+$: 161.07; Found: 161.10.

Compound 1a′: 1-acetyl-1*H*-pyrrolo[2,3-*b*]pyridine-3-carbaldehyde

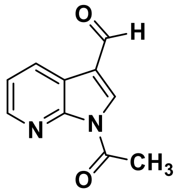

Following the same procedure using Acetyl chloride (1.2 equiv.) and 7-azaindole-3-carboxaldehyde **5a** (146 mg, 1 mmol) in THF provided **1a′** in 69% yield (130 mg) as white solid. ^{1}H NMR (400 MHz, $CDCl_3$): δ 10.09 (s, 1H), 8.61 (s, 1H), 8.59 (dd, J = 8.0, 1.6 Hz, 1H), 8.47 (dd, J = 4.8, 1.6 Hz, 1H), 7.35 (dd, J = 8.0, 4.8 Hz, 1H), 3.13 (s, 3H); ^{13}C NMR (100 MHz, $CDCl_3$): δ 186.17, 169.17, 148.24, 145.96, 135.12, 131.39, 120.63, 119.81, 119.78, 26.21; LRMS (ESI): *m/z* calcd for $C_{10}H_9N_2O_2$ $[M+H]^+$: 189.07; Found: 189.10.

Supplementary Figure 12. Synthesis of *N*-phenyl-protected azaindole-3-carboxaldehyde (**1i**)

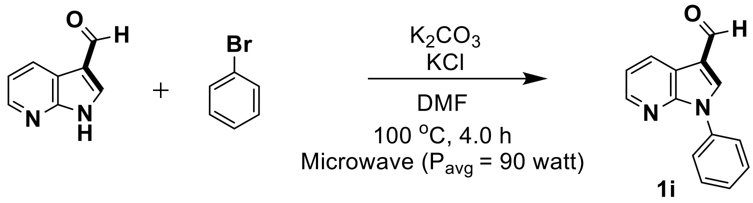

Experimental procedure: The desired product was prepared by following the procedure reported in the literature[7]. In a 10 mL microwave vial equipped with magnetic stir bar and a Teflon-lined screw cap was added azaindole-3-carboxaldehyde (**5a**, 300 mg), KCl (1 equiv.), K_2CO_3 (3 equiv.), bromobenzene (1.1 equiv.), CuI (0.1 equiv.) and DMF (6 mL). The reaction mixture was continuously stirred under microwave irradiation for 4 h while temperature was maintaining at 100 °C. After completion of the reaction monitored by TLC and LC/MS, the reaction mixture was diluted with saturated aq. NH_4Cl solution (10 mL) and the crude compound was extracted using EtOAc (2 × 10 mL). The combined organic extracts were washed with brine (10 mL), dried over anhydrous Na_2SO_4(s), filtered, and concentrated under the reduced pressure. The crude product obtained was then purified by silica-gel flash column chromatography using EtOAc/hexane (1:3) as an eluent to afford the desired starting material **1i** in 65% yield (300 mg) as off-white solid.

Compound 1i: 1-Phenyl-1*H*-pyrrolo[2,3-*b*]pyridine-3-carbaldehyde

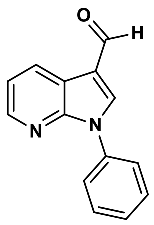

^{1}H NMR (500 MHz, $CDCl_3$): δ 10.09 (s, 1H), 8.65 (d, *J* = 8.0 Hz, 1H), 8.47 (d, *J* = 4.5 Hz, 1H), 8.12 (s, 1H), 7.74 (d, *J* = 7.5 Hz, 2H), 7.59 (t, *J* = 8.0 Hz, 2H), 7.47 (t, *J* = 7.5 Hz, 1H), 7.33 (dd, *J* = 7.5, 4.0 Hz, 1H); ^{13}C NMR (125 MHz, $CDCl_3$): δ 185.06, 148.62, 145.87, 137.80, 137.11, 130.99, 129.80 (2C), 128.13, 124.69 (2C), 119.65, 118.34, 117.70; LRMS (ESI): *m/z* calcd for $C_{14}H_{11}N_2O$ $[M+H]^+$: 223.09; Found: 223.05.

Supplementary Figure 13. Synthesis of *N*-pyridyl-protected azaindole-3-carboxaldehyde **1j**.

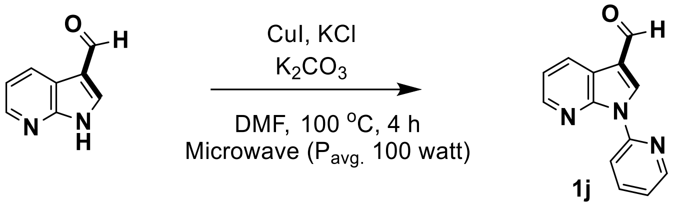

Experimental procedure: The desired product was prepared by following the procedure reported in the literature[7]. 1*H*-Pyrrolo[2,3-*b*]pyridine-3-carbaldehyde (**5a**, 100 mg, 0.68 mmol), potassium chloride (51.01 mg, 1.0 equiv.) , K_2CO_3 (283.71 mg, 3.0 equiv.), 2-chloropyridine (93.23 mg, 1.2 equiv., 77.69 μL), CuI (13.03 mg, 1.2 equiv.) and anhydrous DMF (4 mL) were added to a 10 mL microwave vial and the reaction mixture was irradiated with microwave ($P_{(avg.)}$ = 90 watt) while stirring at 100 °C for 4 h. The reaction was monitored by TLC and LC/MS. Upon completion of the reaction, the reaction mixture was diluted with saturated aqueous NH_4Cl solution (10 mL) and extracted with EtOAc (2 × 10 mL). The combined organic extracts were washed with brine (10 mL), dried over anhydrous Na_2SO_4(s), filtered and evaporated to provide the crude product. The crude product was then purified by silica-gel flash column chromatography (EtOAc:hexane=1:3). The desired starting material 1-(2-pyridyl)pyrrolo[2,3-b]pyridine-3-carbaldehyde (**1j**) obtained in 38% yield (58.0 mg) as off-whit solid.

Compound 1j: 1-(Pyridin-2-yl)-1*H*-pyrrolo[2,3-*b*]pyridine-3-carbaldehyde

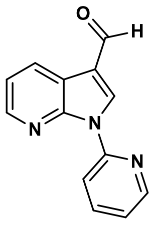

^{1}H NMR (400 MHz, $CDCl_3$): δ 10.11 (s, 1H), 9.03 (s, 1H), 8.95 (d, *J* = 8.0 Hz, 1H), 8.65 (dd, *J* = 8.0, 1.6 Hz, 1H), 8.53 (dd, *J* = 4.8, 1.6 Hz, 1H), 8.49 (dd, *J* = 4.8, 1.6 Hz, 1H), 7.97–7.90 (m, 1H), 7.34 (dd, *J* = 8.0, 4.8 Hz, 1H), 7.29 (m, 1H); ^{13}C NMR (100 MHz, $CDCl_3$): δ 185.76, 149.67, 148.65, 148.37, 145.30, 138.81, 136.71, 131.14, 122.13, 119.77, 119.68, 117.97, 116.70; LRMS (ESI): *m/z* calcd for $C_{13}H_{10}N_3O$ $[M+H]^+$: 224.08; Found: 224.10.

Supplementary Figure 14. Synthesis of *N*-pyrimidyl-protected azaindole-3-carboxaldehyde **1k**

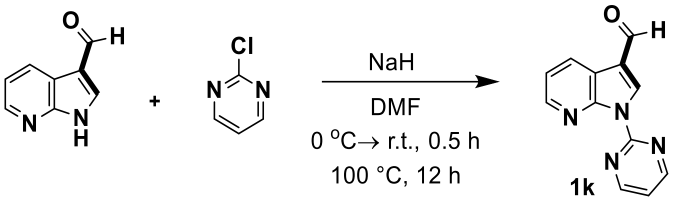

Typical experimental procedure: A round bottom flask placed under the ice bath, equipped with a magnetic bar, charged with 7-azaindole-3-carboxaldehyde (**5a**, 400 mg, 2.74 mmol) in dry DMF (6 mL) was added NaH (2 equiv., 60% suspension in paraffin oil) in a portion wise under argon atmosphere. After complete addition of NaH, the reaction mixture was warmed to r.t. and stirred for 30 min. The reaction mixture was cooled to 0 °C, 2-chloropyrimidine (1.1 equiv.) was added dropwise, the reaction mixture was allowed to attain r.t. and then heated at 100 °C for 12 h. The progress of the reaction was monitored TLC and LC/MS. After completion of the reaction, it was quenched by ice cold water (20 mL), the precipitate obtained was filtered, washed with warm water (50 mL) followed by hot hexane (30 mL), and dried under the reduced pressure to afforded the desired product (**1k**) in 350 mg with 57% yield as off-white solid.

Compound 1k: 1-(Pyrimidin-2-yl)-1*H*-pyrrolo[2,3-*b*]pyridine-3-carbaldehyde

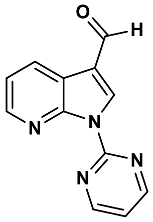

^{1}H NMR (500 MHz, $CDCl_3$): δ 10.13 (s, 1H), 9.01–8.87 (m, 3H), 8.73–8.59 (m, 2H), 7.41–7.29 (m, 2H); ^{13}C NMR (100 MHz, $CDCl_3$): δ 185.66, 159.08 (2C), 156.22, 148.41, 146.67, 137.52, 131.16, 120.19, 119.92, 118.94, 118.92; LRMS (ESI): *m/z* calcd for $C_{12}H_9N_4O$ [M+H]$^+$: 225.08; Found: 225.05.

Supplementary Figure 15. Synthesis of 2-thienylmethyl-protected (aza)indole-3-carboxaldehyde **1l** and **1p**

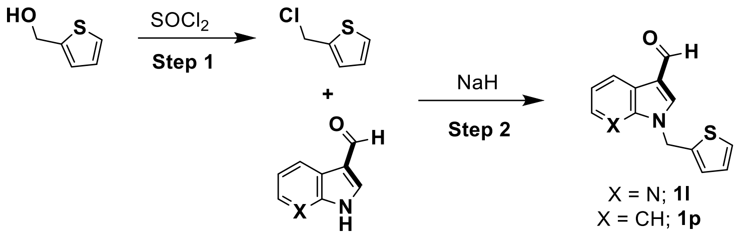

Experimental procedure.

Step1: To a stirred solution of 2-thienyl methanol (500 mg) and Et_3N (1.2 equiv.) in DCM (6 mL) at 0 °C, was slowly added thionyl chloride and the reaction mixture was warmed to 30 ℃. After being stirred for 2 h, the reaction mixture was neutralised with saturated aqueous $NaHCO_3$ solution (10 mL) and crude compound was extracted with DCM (2 × 10 mL). The combined organic extracts were washed with brine (10 mL), dried over anhydrous Na_2SO_4(s), and filtered and concentrated *in vacuo* to afford crude (2-chloromethyl)thiophene. The product was a lachrymator agent in nature and used in the next step without further purification and characterization[8].

Step 2: To a well stirred, ice-cold solution of (aza)indole (400 mg) in dry DMF (5 mL) was added of NaH (2 equiv., 60% suspension in paraffin oil) under argon atmosphere. The reaction mixture was then allowed to attain r.t. and stirred for 30 min. The reaction mixture was then cooled to 0 ℃, added dropwise with the solution of crude (2-chloromethyl)thiophene (1.1 equiv.) in 1 mL of DMF obtained from the step 1, and allowed to attain r.t. After being stirred for 2 h, the reaction mixture was quenched with ice-cold water (50 mL) and the crude compound was extracted into EtOAc (2 × 20 mL). The combined organic extract was washed with brine (20 mL), dried over anhydrous Na_2SO_4(s), concentrated *in vacuo,* and purified by silica-gel flash column chromatography using EtOAc:hexane mixture (3:7) as an eluent to furnish the desired product.

Compound 1l: 1-(Thiophen-2-ylmethyl)-1*H*-pyrrolo[2,3-*b*]pyridine-3-carbaldehyde

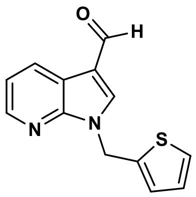

Following the general procedure described above with 1*H*-pyrrolo[2,3-b]pyridine-3-carbaldehyde **5a** (0.4 g, 2.74 mmol) provided **1l** in 75% yield (0.5 g) as brown solid. ^{1}H NMR (500 MHz, $CDCl_3$) δ 9.93 (s, 1H), 8.54 (dd, *J* = 8.0, 2.0 Hz, 1H), 8.46 (dd, *J* = 4.5, 1.5 Hz, 1H), 7.85 (s, 1H), 7.30–7.24 (m, 2H), 7.13 (d, *J* = 3.5 Hz, 1H), 6.98 (dd, *J* = 5.0, 3.5 Hz, 1H), 5.69 (s, 2H); ^{13}C NMR (125 MHz, $CDCl_3$) δ 184.71, 148.29, 145.31, 137.75, 137.33, 130.69, 127.86, 127.34, 126.60, 119.23, 117.75, 117.04, 43.09; LRMS (ESI): *m/z* calcd for $C_{13}H_{11}N_2OS$ $[M+H]^+$: 243.06; Found: 243.05.

Compound 1p: 1-(Thiophen-2-ylmethyl)-1*H*-indole-3-carbaldehyde

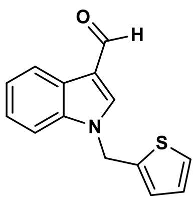

Following the general procedure described above with 1*H*-indole-3-carbaldehyde **5f** (0.25 g, 1.72 mmol) provided **1p** in 63% yield (0.26 g) as brown solid. ^{1}H NMR (400 MHz, $CDCl_3$) δ 9.98 (s, 1H), 8.35–8.28 (m, 1H), 7.71 (s, 1H), 7.45–7.40 (m, 1H), 7.36–7.26 (m, 3H), 7.03 (d, *J* = 3.6 Hz, 1H), 6.98 (dd, *J* = 5.2, 3.2 Hz, 1H), 5.49 (s, 2H); ^{13}C NMR (100 MHz, $CDCl_3$): δ 184.75, 137.90, 137.48, 137.22, 127.41, 127.37, 126.55, 125.64, 124.32, 123.28, 122.36, 118.79, 110.22, 45.72; LRMS (ESI): *m/z* calcd for $C_{14}H_{12}NOS$ $[M+H]^+$: 242.06; Found: 242.10.

Supplementary Figure 16. Synthesis of boronic ester-substituted indole-3-carboaldehydes

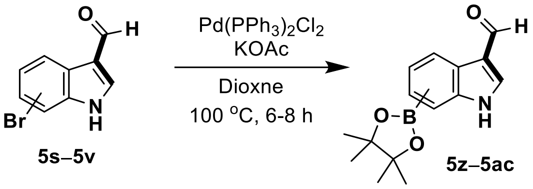

General procedure: The suspension of bromo-substituted indole-3-carboxaldehyde and 4,4,5,5-tetramethyl-2-(4,4,5,5-tetramethyl-1,3,2-dioxaborolan-2-yl)-1,3,2-dioxaborolane (1.2 equiv.) $Pd(PPh_3)_2Cl_2$ (5.0 mol%) and potassium acetate (3.0 equiv.) in dioxane was degassed with argon for 20 min and then stirred at 100 °C for 6–8 h. The progress of the reaction was monitored by TLC and LC/MS. After completion of the reaction, the reaction mixture was cooled to r.t., diluted with saturated aqueous $NaHCO_3$, filtered through a short pad of celite, and washed with EtOAc (10 mL). The crude compound was then extracted from filtrate using EtOAc (3 × 20 mL), the combined organic extract was given a brine wash (3 × 10 mL), dried over anhydrous Na_2SO_4(s), and filtered and concentrated under the reduced pressure. The crude compound was purified by silica-gel flash column chromatography (EtOAc:hexane = 3:7) to provide the desired product (**5z**–**5ac**) as an off-white solid.

Compound 5z: 4-(4,4,5,5-Tetramethyl-1,3,2-dioxaborolan-2-yl)-1*H*-indole-3-carbaldehyde

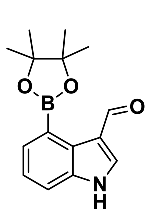

Following the general procedure described above with 4-bromo-1*H*-indole-3-carbaldehyde **5s** (0.224 g, 1 mmol) provided **5z** in 52% yield (0.141 g) as white solid. ^{1}H NMR (500 MHz, DMSO-d_6): δ 12.21 (s, 1H), 10.25 (s, 1H), 8.23 (s, 1H), 7.58 (dd, J = 8.0, 1.0 Hz, 1H), 7.41 (dd, J = 7.0, 1.0 Hz, 1H), 7.23 (dd, J = 8.0, 7.0 Hz, 1H), 1.36 (s, 12H); ^{13}C NMR (125 MHz, DMSO-d_6): δ 186.17, 136.36, 135.04, 128.44, 126.72, 122.31, 119.11, 114.50, 83.46 (2C), 24.97 (4C) (boron attached carbon not observed); LRMS (ESI): *m/z* calcd for $C_{15}H_{19}BNO_3$ $[M+H]^+$: 272.15; Found: 272.20.

Compound 5aa: 5-(4,4,5,5-Tetramethyl-1,3,2-dioxaborolan-2-yl)-1*H*-indole-3-carbaldehyde

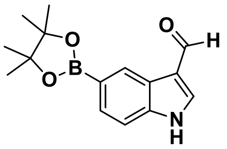

Following the general procedure described above with 5-bromo-1*H*-indole-3-carbaldehyde **5t** (0.224 g, 1 mmol) provided **5aa** in 94% yield (0.255 g) as white solid. ^{1}H NMR (500 MHz, DMSO-d_6): δ 12.23 (s, 1H), 9.96 (s, 1H), 8.53 (s, 1H), 8.33 (s, 1H), 7.57 (dd, *J* = 9.0, 1.0 Hz, 1H), 7.51 (d, *J* = 8.0, 0.5 Hz, 1H), 1.32 (s, 12H); ^{13}C NMR (125 MHz, DMSO-d_6): δ 185.17, 139.06, 138.99, 129.24, 128.36, 123.72, 118.30, 111.89, 83.41 (2C), 24.72 (4C) (boron attached carbon not observed); LRMS (ESI): *m/z* calcd for $C_{15}H_{19}BNO_3$ [M+H]$^+$: 272.15; Found: 272.15.

Compound 5ab: 6-(4,4,5,5-Tetramethyl-1,3,2-dioxaborolan-2-yl)-1*H*-indole-3-carbaldehyde

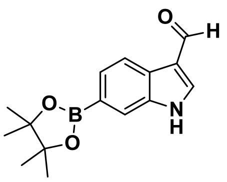

Following the general procedure described above with 6-bromo-1*H*-indole-3-carbaldehyde **5u** (0.4 g, 1.79 mmol) provided **5ab** in 76% yield (0.366 g) as white solid. ^{1}H NMR (400 MHz, DMSO-d_6): δ 12.21 (s, 1H), 9.95 (s, 1H), 8.38 (s, 1H), 8.10 (dd, *J* = 7.6, 0.8 Hz, 1H), 7.84 (d, *J* = 0.8 Hz, 1H), 7.53 (dd, *J* = 8.0, 0.8 Hz, 1H), 1.32 (s, 12H); ^{13}C NMR (100 MHz, DMSO-d_6): δ 185.02, 139.45, 136.73, 127.72, 126.57, 120.24, 118.89, 118.12, 83.50 (2C), 24.71 (4C) (boron attached carbon not observed); LRMS (ESI): *m/z* calcd for $C_{15}H_{19}BNO_3$ [M+H]$^+$: 272.15; Found: 272.15.

Compound 5ac: 7-(4,4,5,5-Tetramethyl-1,3,2-dioxaborolan-2-yl)-1*H*-indole-3-carbaldehyde

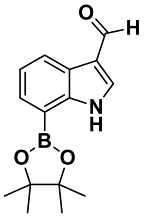

Following the general procedure described above with 7-bromo-1*H*-indole-3-carbaldehyde **5v** (0.1 g, 0.45 mmol) provided **5ac** in 91% yield (0.11 g) as colourless liquid. ^{1}H NMR (400 MHz, $CDCl_3$): δ 10.06 (s, 1H), 9.79 (s, 1H), 8.43 (dt, *J* = 8.0, 0.8 Hz, 1H), 7.88 (d, *J* = 2.8 Hz, 1H), 7.76 (dd, *J* = 7.2, 1.2 Hz, 1H), 7.33 (t, *J* = 8.0, 1H), 1.41 (s, 12H); ^{13}C NMR (100 MHz, $CDCl_3$): δ 185.32, 142.16, 135.44, 131.53, 125.58, 123.59, 122.67, 119.50, 84.39 92C), 25.13 (4C) (boron attached carbon not observed); LRMS (ESI): *m/z* calcd for $C_{15}H_{19}BNO_3$ [M+H]$^+$: 272.15; Found: 272.15.

Supplementary Figure 17. Synthesis of starting material **5ah** (a precursor of *Nico*-procain scaffold)

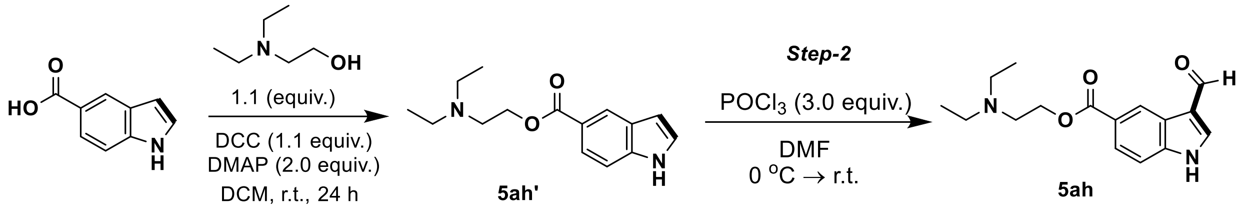

Experimental procedure

Step 1: To a stirred solution of 1*H*-indole-5-carboxylic acid (250 mg) in DCM (16 mL), were added 2-(diethylamino)ethanol (1.1 equiv.), 1,3-dicyclohexylcarbodiimide (DCC, 1.1 equiv.) and 4-dimethylaminpyridine (DMAP, 2.0 equiv.) at r.t. The reaction mixture was then stirred at r.t. for 24 h. The progress of the reaction was monitored by TLC and LC/MS. After completion of the reaction, it was diluted with water (20 mL) and extracted with DCM (2 × 20 mL). The combined organic fractions were washed with brine (20 mL), dried over anhydrous Na_2SO_4(s), filtered, and concentrated under the reduced pressure to provide a crude product. The crude product was then purified by silica-gel flash column chromatography (MeOH/DCM = 5:95 to 10:90) to furnish the desired product (404 mg, 37%) which was used in the next step for the synthesis of starting material **5ah′** as a colourless gummy liquid.

Compound 5ah′: 2-(Diethylamino)ethyl 1*H*-indole-5-carboxylate

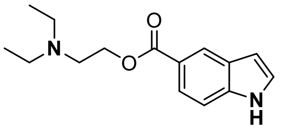

^{1}H NMR (400 MHz, DMSO-d_6): δ 11.49 (s, 1H), 8.26 (d, *J* = 1.6 Hz, 1H), 7.73 (dd, *J* = 8.4, 1.6 Hz, 1H), 7.51–7.44 (m, 2H), 6.60 (t, *J* = 2.4 Hz, 1H), 4.31 (t, *J* = 6.0 Hz, 2H), 2.88–2.76 (m, 2H), 2.59 (q, *J* = 7.2 Hz, 4H), 1.00 (t, *J* = 7.2 Hz, 6H); ^{13}C NMR (125 MHz, DMSO-d_6): δ 166.78, 138.47, 127.22, 127.19, 122.72, 121.93, 120.53, 111.33, 102.58, 50.76, 47.07 (3C), 11.93, 11.89; LRMS (ESI): *m/z* calcd for $C_{15}H_{21}N_2O_2$ $[M+H]^+$: 261.16; Found: 261.20.

Step 2: The starting material **5ah** (45% as white solid) was synthesized according to the experimental procedure described in Supplementary Figure 9.

Compound 5ah: 2-(Diethylamino)ethyl 3-formyl-1*H*-indole-5-carboxylate

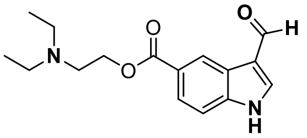

^{1}H NMR (400 MHz, DMSO-*d*$_6$): δ 10.02 (s, 1H), 8.82 (d, *J* = 2.0 Hz, 1H), 8.46 (s, 1H), 7.91 (dd, *J* = 8.8, 2.0 Hz, 1H), 7.64 (d, *J* = 8.4 Hz, 1H), 4.34 (t, *J* = 6.0 Hz, 2H), 2.79 (t, *J* = 6.0 Hz, 2H), 2.56 (q, *J* = 7.2 Hz, 4H), 1.00 (t, *J* = 7.2 Hz, 6H); ^{13}C NMR (100 MHz, DMSO-*d*$_6$): δ 185.24, 166.33, 140.11, 139.66, 124.39, 123.74, 123.09, 118.72, 112.48, 62.93, 50.74, 47.08 (2C), 12.03 (2C); LRMS (ESI): *m/z* calcd for $C_{16}H_{21}N_2O_3$ [M+H]$^+$: 289.16; Found: 289.15.

Supplementary Figure 18. Synthesis of propiolate from propiolic acid

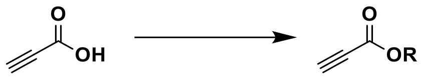

General experimental procedure.

Method A: The mixture of propiolic acid, p-toluenesulfonic acid (*p*TsOH, 5.0 mol%), and appropriate alcohols or compounds bearing hydroxyl group (1.0 equiv.) in toluene (2 mL per 100 mg propiolic acid) was refluxed for 12 h in a 50 mL round bottom flask equipped with a magnetic bar and a Dean-stark condenser. After being stirred for 12 h, the reaction was brought to r.t., toluene was evaporated and the crude compound was purified by silica-gel flash column chromatography (EtOAc:hexane = 5:95) to obtain the desired product.

Method B: In a dried round bottom flask equipped with a magnetic bar, the equimolar mixture of prop-2-ynoic acid, appropriate compound bearing hydroxyl group, and DMAP were dissolved in dry DCM. To this reaction mixture was added DCC (1.1 equiv.) under argon atmosphere at r.t., and the reaction mixture was stirred for 2 h. The progress of the reaction was monitored by TLC via observing the complete consumption of alcohol. After completion of the reaction, the reaction mixture was passed through a short pad of celite, washed with DCM, and subjected to silica-gel flash column chromatography to obtain the desired propiolate.

Compound 2c: Benzyl propiolate

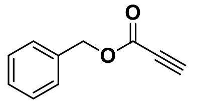

Compound **2c** (1.01 g, 89% yield, as yellow liquid) was prepared by the synthetic procedure described in **Method A** using propiolic acid (0.5 g, 7.14 mmol) and benzyl alcohol. ^{1}H NMR (400 MHz, $CDCl_3$): δ 7.45–7.31 (m, 5H), 5.22 (s, 2H), 2.89 (s, 1H); ^{13}C NMR (100 MHz, $CDCl_3$): δ 152.64, 134.64, 128.83, 128.79 (2C), 128.68 (2C), 75.21, 74.65, 68.01; LRMS (ESI): *m/z* calcd for $C_{10}H_9O_2$ $[M+H]^+$: 161.06; Found: 161.20.

Compound 2d: Hexyl propiolate

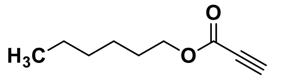

Compound **2d** (0.76 g, 69% yield, as light yellow liquid) was prepared by the synthetic procedure described in **Method A** using propiolic acid (0.5 g, 7.14 mmol) and 1-hexanol. ^{1}H NMR (400 MHz, $CDCl_3$) δ 4.19 (t, *J* = 6.4 Hz, 2H), 2.88 (s, 1H), 1.73–1.63 (m, 2H), 1.42–1.26 (m, 6H), 0.90 (t, *J* = 6.4 Hz, 3H); ^{13}C NMR (100 MHz, $CDCl_3$): δ 152.95, 74.92, 74.53, 66.60, 31.45, 28.39, 25.53, 22.60, 14.08; LRMS (ESI): *m/z* calcd for $C_9H_{15}O_2$ $[M+H]^+$: 155.11; Found: 155.05.

Compound 2e: Tetradecyl propiolate

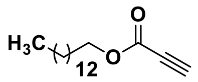

Compound **2e** (1.9 g, 99.9% yield, as white solid) was prepared by the synthetic procedure described in **Method A** using propiolic acid (0.5 g, 7.14 mmol) and Myristyl alcohol. ^{1}H NMR (400 MHz, $CDCl_3$): δ 4.19 (t, *J* = 6.8 Hz, 2H), 2.87 (s, 1H), 1.68 (p, *J* = 6.8 Hz, 2H), 1.40–1.18 (m, 22H), 0.88 (t, *J* = 6.8 Hz, 3H); ^{13}C NMR (100 MHz, $CDCl_3$): δ 152.98, 74.96, 74.50, 66.66, 32.07, 29.83, 29.80 (2C), 29.77, 29.69, 29.61, 29.50, 29.31, 28.46, 25.89, 22.84, 14.27; LRMS (ESI): *m/z* calcd for $C_{17}H_{31}O_2$ $[M+H]^+$: 267.23; Found: 267.10.

Compound 2f: 2-(1,3-Dimethyl-2,6-dioxo-1,2,3,6-tetrahydro-7*H*-purin-7-yl)ethyl propiolate

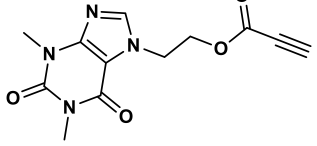

Compound **2f** (1 g, 41% yield, as yellow solid) was prepared by the synthetic procedure described in **Method B** using propiolic acid (0.625 g, 8.92 mmol) and Etofylline (7-(2-hydroxyethyl)-1,3-dimethyl-purine-2,6-dione). ^{1}H NMR (400 MHz, $CDCl_3$): δ 7.61 (s, 1H), 4.66–4.54 (m, 4H), 3.60 (s, 3H), 3.41 (s, 3H), 2.96 (s, 1H); ^{13}C NMR (100 MHz, $CDCl_3$): δ 155.38, 151.98, 151.72, 149.25,

141.79, 106.72, 76.23, 73.93, 64.28, 45.72, 29.96, 28.13; LRMS (ESI): *m/z* calcd for $C_{12}H_{13}N_4O_4$ $[M+H]^+$: 277.09; Found: 277.10.

Compound 2g: (1*R*,5*S*)-3,3,5-Trimethylcyclohexyl propiolate

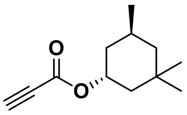

Compound **2g** (0.3 g, 22% yield, as colourless liquid) was prepared by the synthetic procedure described in **Method A** using propiolic acid (0.5 g, 7.14 mmol) and (1*R*,5*S*)-3,3,5-trimethylcyclohexanol. ^{1}H NMR (400 MHz, $CDCl_3$) δ 5.20 (t, *J* = 3.2 Hz, 1H), 2.83 (s, 1H), 1.98–1.83 (m, 2H), 1.75 (dq, *J* = 15.2, 2.4 Hz, 1H), 1.46 (dq, *J* = 13.2, 2.4 Hz, 1H), 1.28 (dd, *J* = 14.8, 3.6 Hz, 1H), 1.10–1.01 (m, 4H), 0.93–0.82 (m, 7H); ^{13}C NMR (100 MHz, $CDCl_3$): δ 152.51, 75.42, 73.99, 73.86, 48.04, 41.13, 38.20, 33.98, 30.71, 27.23, 23.42, 22.48; LRMS (ESI): *m/z* calcd for $C_{12}H_{19}O_2$ $[M+H]^+$: 195.14; Found: 195.30.

Compound 2h: (8*R*,9*S*,10*R*,13*S*,14*S*,17*S*)-10,13-Dimethyl-3-oxo-2,3,6,7,8,9,10,11,12,13,14,15,16,17-tetradecahydro-1*H*-cyclopenta[a]phenanthren-17-yl propiolate

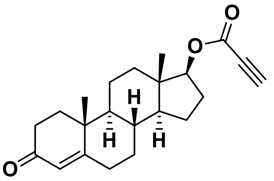

Compound **2h** (1.58 g, 64% yield, as yellow solid) was prepared by the synthetic procedure described in **Method A** using propiolic acid (0.5 g, 7.14 mmol) and Testosterone (17*β*-Hydroxyandrost-4-en-3-one). ^{1}H NMR (400 MHz, $CDCl_3$): δ 5.73 (s, 1H), 4.71 (t, *J* = 8.4 Hz, 1H), 2.91 (s, 1H), 2.47–2.17 (m, 5H), 2.07–2.00 (m, 1H), 1.90–1.81 (m, 2H), 1.76–1.55 (m, 5H), 1.48–1.32 (m, 2H), 1.20 (s, 4H), 1.13–1.01 (m, 2H), 1.00–0.85 (m, 4H); ^{13}C NMR (100 MHz, $CDCl_3$): δ 199.46, 170.84, 152.84, 124.05, 84.68, 75.00, 74.60, 53.68, 50.20, 42.80, 38.66, 36.59, 35.76, 35.43, 33.99, 32.75, 31.50, 27.35, 23.51, 20.56, 17.47, 12.12; LRMS (ESI): *m/z* calcd for $C_{22}H_{29}O_3$ $[M+H]^+$: 341.21; Found: 341.30.

Compound 2i: (8*R*,9*S*,13*S*,14*S*,17*S*)-3-Hydroxy-13-methyl-7,8,9,11,12,13,14,15,16,17-decahydro-6*H*-cyclopenta[a]phenanthren-17-yl propiolate

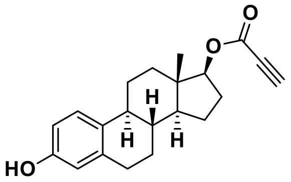

Compound **2i** (1 g, 89% yield, as yellow solid) was prepared by the synthetic procedure described in **Method A** using propiolic acid (0.243 g, 3.47 mmol) and estradiol. ^{1}H NMR (400 MHz, DMSO-d_6) δ 9.00 (s, 1H), 7.03 (d, *J* = 8.4 Hz, 1H), 6.50 (dd, *J* = 8.4, 2.8 Hz, 1H), 6.43 (d, *J* = 2.8 Hz, 1H), 4.72 (t, *J* = 8.4 Hz, 1H), 4.56 (s, 1H), 2.81–2.63 (m, 2H), 2.27–2.05 (m, 3H), 1.82–1.64 (m, 3H), 1.61–1.49 (m,

1H), 1.42–1.21 (m, 6H), 0.78 (s, 3H); ^{13}C NMR (100 MHz, DMSO-d_6): δ 154.97, 152.30, 137.04, 130.00, 126.05, 114.93, 112.75, 84.30, 78.89, 74.88, 48.79, 43.13, 42.73, 38.21, 36.30, 29.06, 26.92, 26.78, 25.81, 22.74, 11.94; LRMS (ESI): *m/z* calcd for $C_{21}H_{25}O_3$ [M+H]$^+$: 325.18; Found: 325.20.

Supplementary Figure 19. Synthesis of compound **5f′** (1*H*-Indole-3-carbaldehyde-*d*)

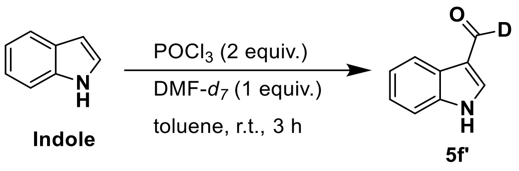

Compound 5f′:

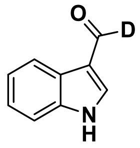

5f′

Compound **5f′** (44% yield, as yellow solid) was prepared using the procedure reported in the literature[9]. ^{1}H NMR (400 MHz, DMSO-d_6): δ 12.13 (s, 1H), 8.29 (s, 1H), 8.18–8.04 (m, 1H), 7.52 (d, *J* = 7.6 Hz, 1H), 7.31–7.17 (m, 2H); ^{13}C NMR (125 MHz, DMSO-d_6): δ 184.70 (t, J_{DC} = 25.5 Hz, 1C), 138.38, 137.04, 124.12, 123.43, 122.09, 120.81, 118.07, 112.39; LRMS (ESI): *m/z* calcd for C_9H_7DNO [M+H]$^+$: 147.07; Found: 147.15.

Supplementary Figure 20. Synthesis of compound **5f″** (1*H*-Indole-2-*d*-3-carbaldehyde)

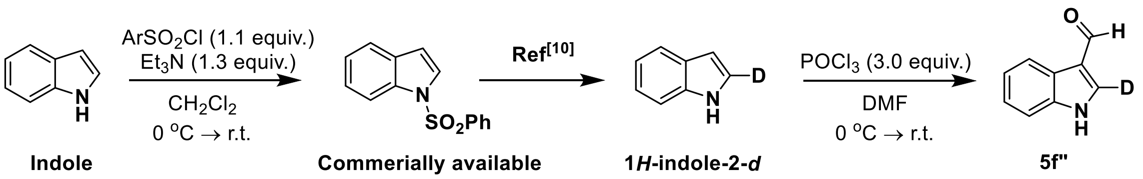

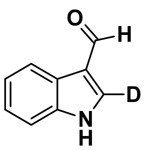

5f″

Following the general procedure described in Supplementary Figure 9 with 1*H*-indole-2-*d* (0.236 g, 2 mmol) provided **5f″** in 72% yield (0.211 g) as pale orange solid. ^{1}H NMR (500 MHz, DMSO-d_6) δ 12.15 (s, 1H), 9.94 (s, 1H), 8.14–8.08 (m, 1H), 7.52 (dt, *J* = 8.0, 1.0 Hz, 1H), 7.30–7.20 (m, 2H); ^{13}C NMR (125 MHz, DMSO-d_6): δ 184.96, 137.02, 124.12, 123.47, 122.14, 120.84, 118.03, 112.44; LRMS (ESI): *m/z* calcd for C_9H_6DNO [M+H]$^+$: 147.07; Found: 147.15.

Supplementary Figure 21. Synthesis of compound **9a** (7*H*-[1,3]Dioxolo[4,5-*J*]pyrrolo[3,2,1-de]phenanthridine-4-carbaldehyde)

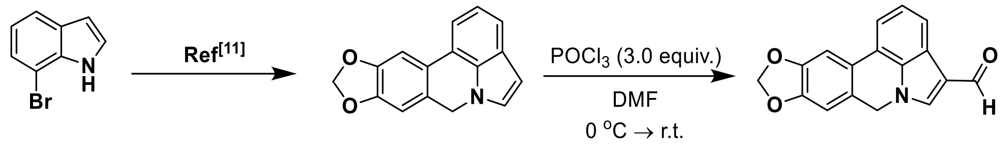

7-Bromoindole dehydroanhydrolycorine **8a** **9a**

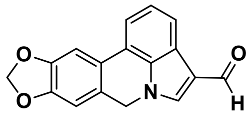

Compound **8a** was prepared using the procedure reported in the literature[11]. Following the general procedure described in **Supplementary Figure 9** with **8a** (0.104 g, 0.4 mmol) provided **9a** in 74% yield (0.085 g) as off-white solid. ^{1}H NMR (400 MHz, DMSO-d_6): δ 9.93 (s, 1H), 8.31 (s, 1H), 7.82 (d, *J* = 8.0 Hz, 1H), 7.66 (s, 1H), 7.65 (d, *J* = 7.2 Hz, 1H), 7.20 (t, *J* = 7.6 Hz, 1H), 6.95 (s, 1H), 6.09 (s, 2H), 5.60 (s, 2H); ^{13}C NMR (100 MHz, DMSO-d_6): δ 184.86, 147.72, 147.55, 138.47, 133.00, 123.76, 123.57, 122.10, 122.03, 119.93, 119.19, 118.53, 115.37, 107.46, 103.02, 101.52, 47.57; LRMS (ESI): *m/z* calcd for $C_{17}H_{12}NO_3$ $[M+H]^+$: 278.08; Found: 278.10.

Supplementary Figure 22. Synthesis of compound **12a**

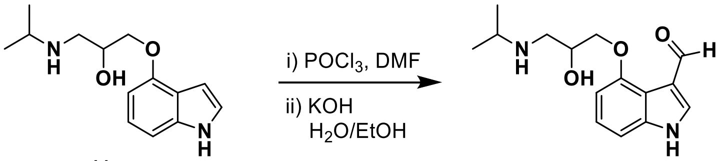

11a
Pindolol
(Non Selective β-blocker)

12a (35%)

Compound 12a: 4-(2-Hydroxy-3-(isopropylamino)propoxy)-1*H*-indole-3-carbaldehyde

To an ice-cooled stirred solution of **11a** (288 mg, 1.0 mmol) in dry DMF (5 mL) was slowly added $POCl_3$ (561 μL, 3.0 mmol) at 0 °C. The reaction was monitored by TLC and LC/MS. After completion of the reaction, the reaction mixture was poured into a solution of KOH (280 mg, 5.0 mmol) in EtOH (50 mL) and water (5 mL) at 0 °C. The solution was heated to 40 °C and stirred for 24 h. The reaction of hydrolysis was monitored by LC/MS. After the completion of the reaction, organic layer was extracted with 10 % MeOH in DCM solution (3 × 20 mL), dried over anhydrous Na_2SO_4(s), filtered,

concentrated under the reduced pressure, and purified by silica-gel flash column chromatography using DCM/MeOH mixture as an eluent to furnish **12a** (35%) as yellow gummy liquid.

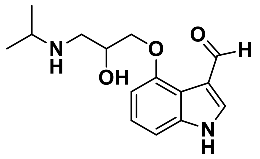

^{1}H NMR (500 MHz, DMSO-d_6): δ 12.32 (s, 1H), 10.28 (s, 1H), 8.50 (s, 2H), 8.11 (d, J = 3.5 Hz, 1H), 7.22–7.10 (m, 2H), 6.76 (dd, J = 7.0, 1.0 Hz, 1H), 4.31–4.23 (m, 1H), 4.22–4.08 (m, 2H), 3.43–3.22 (m, 2H), 3.15–3.04 (m, 1H), 2.50 (p, J = 2.0 Hz, 2H), 1.26 (dd, J = 6.5, 5.0 Hz, 6H); ^{13}C NMR (125 MHz, DMSO-d_6): δ 186.15, 152.67, 138.33, 131.93, 123.76, 118.23, 115.25, 106.20, 103.23, 69.81, 65.35, 49.83, 46.70, 18.74, 18.12; LRMS (ESI): *m/z* calcd for $C_{15}H_{21}N_2O_3$ [M+H]$^+$: 277.16; Found: 277.20.

Supplementary Figure 23. Synthesis of compound **15a**

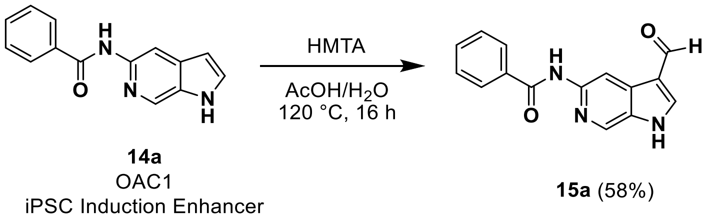

Compound 15a: *N*-(3-Formyl-1*H*-pyrrolo[2,3-c]pyridin-5-yl)benzamide

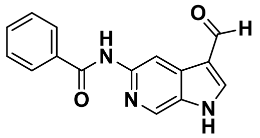

Following the general procedure described in **Supplementary Figure 8** with **14a** (0.2 g, 0.84 mmol) provided **5a″** in 58% yield (0.129 g) as brown solid. ^{1}H NMR (500 MHz, DMSO-d_6) δ 12.46 (s, 1H), 10.62 (s, 1H), 10.01 (s, 1H), 8.87 (d, J = 1.5 Hz, 1H), 8.68 (d, J = 1.5 Hz, 1H), 8.51 (s, 1H), 8.14–8.01 (m, 2H), 7.64–7.43 (m, 3H); ^{13}C NMR (125 MHz, DMSO-d_6): δ 185.07, 165.26, 145.78, 141.81, 134.49, 132.84, 131.72, 131.59, 131.13, 128.31, 128.25, 127.81, 127.68, 117.58, 105.72; LRMS (ESI): *m/z* calcd for $C_{15}H_{12}N_3O_2$ [M+H]$^+$: 266.09; Found: 266.10.

IV. Experimental Synthetic Procedures and Characterization of *meta*-Aminoaryl Nicotinate Final Products

Supplementary Figure 24. Skeletal transformation of *N*-substituted (aza)indole-3-carboxaldehydes to meta-aminoaryl nicotinates (**3a–3z**) using ethyl propiolate

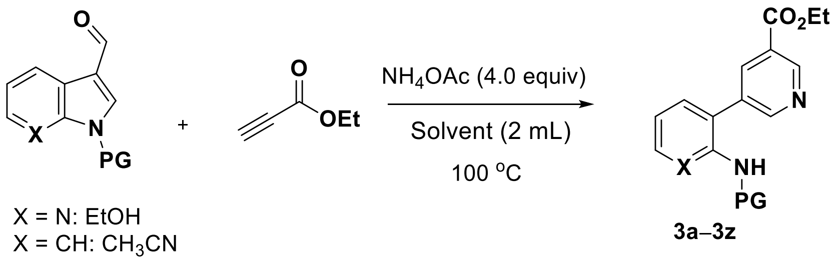

General procedure. A 4-mL vial equipped with a magnetic stir bar and a Teflon-lined screwed cap was charged with **1** (0.2 mmol), ethyl propiolate (1.2 equiv.), and NH_4OAc (61.66 mg, 4 equiv.) in the appropriate solvent (2.0 mL, EtOH or CH_3CN). The vial was then sealed and heated at 100 °C for 8 h. Upon reaction completion monitored by TLC and LC/MS analysis, the reaction mixture was concentrated under the reduced pressure, added with saturated aqueous $NaHCO_3$, and extracted with DCM (3 × 10 mL). The combined organic fractions were dried over anhydrous Na_2SO_4(s), filtered, and concentrated under the reduced pressure. The crude compound was purified by silica-gel flash column chromatography to obtain the desired product bearing the nicotinate scaffolds (**3**). For products **3b, 3d**, **3h**, **3i**, and **3l**, the reaction was performed in 0.5 mmol scale in a 4 mL vial equipped with a Teflon-lined screwed cap and ~2.0 mL of ethanol was used as solvent. For product **3f**, the reaction was performed in 500 mg scale in a 20 mL vial equipped with a Teflon-lined screwed cap and ~8.0 mL of ethanol was used as solvent.

Compound 3a: Ethyl 2'-(phenylsulfonamido)-[3,3'-bipyridine]-5-carboxylate

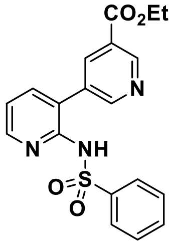

3a

Brown solid; Yield: 95% (avg); ^{1}H NMR (500 MHz, $CDCl_3$): δ 9.14 (d, *J* = 2.5 Hz, 1H), 8.82 (d, *J* = 2.5 Hz, 1H), 8.42 (t, *J* = 2.5 Hz, 1H), 8.01–7.95 (m, 2H), 7.87–7.80 (m, 1H), 7.66 (dd, *J* = 7.0, 1.5 Hz, 1H), 7.55–7.44 (m, 3H), 6.77 (t, *J* = 6.5 Hz, 1H), 4.41 (q, *J* = 7.0 Hz, 2H), 1.40 (t, *J* = 7.0 Hz, 3H); ^{13}C NMR (125 MHz, $CDCl_3$): δ 165.00, 153.15, 151.69, 150.23, 142.85, 141.54, 137.77, 136.61, 132.16, 131.65, 128.93 (2C), 128.14, 126.21 (2C), 125.93, 112.57, 61.66, 14.36; IR: 3210, 1720, 1623, 1589, 1257, 741 cm^{-1}; HRMS (ESI+): *m/z* calcd for $C_{19}H_{17}N_3NaO_4S^+$ $[M+Na]^+$: 406.08320; Found: 406.0833.

Compound 3b: Ethyl 2'-((4-methoxyphenyl)sulfonamido)-[3,3'-bipyridine]-5-carboxylate

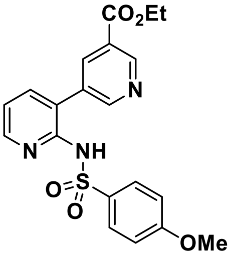

3b

Off-white solid; Yield: 67% (122 mg); ^{1}H NMR (500 MHz, $CDCl_3$): δ 9.14 (s, 1H), 8.83 (s, 1H), 8.40 (t, *J* = 2.0 Hz, 1H), 7.92 (d, *J* = 9.0 Hz, 2H), 7.84 (s, 1H), 7.62 (d, *J* = 7.0 Hz, 1H), 6.99–6.90 (m, 2H), 6.77 (s, 1H), 4.42 (q, *J* = 7.0 Hz, 2H), 3.84 (s, 3H), 1.40 (t, *J* = 7.0 Hz, 3H); ^{13}C NMR (125 MHz, $CDCl_3$); δ 165.00, 162.59, 153.23, 151.41, 150.26, 141.14 (2C), 137.65 (2C), 134.71, 131.77, 128.51 (2C), 128.29, 125.99, 114.04 (2C), 61.64, 55.62, 14.35. IR: 2988, 1726, 1595. 1335, 1260, 1155, 577 cm^{-1}; LRMS (ESI): *m/z* calcd for $C_{20}H_{20}N_3O_5S$ $[M+H]^+$: 414.11; Found: 414.05.

Compound 3d: Ethyl 2'-(benzylamino)-[3,3'-bipyridine]-5-carboxylate

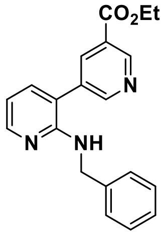

3d

Gummy yellow liquid; Yield: 96% (avg); ^{1}H NMR (500 MHz, $CDCl_3$): δ 9.19 (d, *J* = 2.0 Hz, 1H), 8.84 (d, *J* = 2.5 Hz, 1H), 8.38 (t, *J* = 2.0 Hz, 1H), 8.23 (dd, *J* = 5.0, 2.0 Hz, 1H), 7.34–7.27 (m, 5H), 7.26–7.20 (m, 1H), 6.74 (dd, *J* = 7.0, 5.0 Hz, 1H), 4.70–4.59 (m, 3H), 4.42 (q, *J* = 7.0 Hz, 2H), 1.41 (t, *J* = 7.0 Hz, 3H); ^{13}C NMR (125 MHz, $CDCl_3$): δ 164.99, 155.41, 153.55, 150.17, 148.66, 139.57, 137.94, 137.51, 133.82, 128.73 (2C), 127.73 (2C), 127.33, 126.79, 117.59, 113.45, 61.80, 45.77, 14.40; IR: 3314, 1719, 1575, 1500, 1261, 766 cm^{-1}; HRMS (ESI+): *m/z* calcd for $C_{20}H_{20}N_3O_2^+$ $[M+H]^+$: 334.15500; Found: 334.1564.

Compound 3e: Ethyl 2'-((2-bromobenzyl)amino)-[3,3'-bipyridine]-5-carboxylate

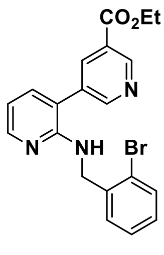

3e

White solid; Yield: 87% (100 mg); ^{1}H NMR (400 MHz, $CDCl_3$): δ 9.21 (d, J = 2.0 Hz, 1H), 8.84 (d, J = 2.4 Hz, 1H), 8.38 (t, J = 2.4 Hz, 1H), 8.21 (dd, J = 4.8, 1.6 Hz, 1H), 7.52 (dd, J = 8.0, 1.6 Hz, 1H), 7.42 (dd, J = 7.6, 1.6 Hz, 1H), 7.31 (dd, J = 7.2, 2.0 Hz, 1H), 7.27–7.22 (m, 1H), 7.11 (td, J = 8.0, 2.0 Hz, 1H), 6.72 (dd, J = 7.6, 5.2 Hz, 1H), 4.88 (t, J = 6.0 Hz, 1H), 4.70 (d, J = 6.0 Hz, 2H), 4.43 (q, J = 7.2 Hz, 2H), 1.42 (t, J = 7.2 Hz, 3H); ^{13}C NMR (100 MHz, $CDCl_3$): δ 165.00, 155.12, 153.59, 150.22, 148.62, 138.50, 137.92, 137.51, 133.72, 132.92, 130.41, 128.93, 127.54, 126.74, 123.93, 117.68, 113.53, 61.80, 45.98, 14.42.; IR: 2978, 1715, 1637, 1438, 1260, 747 cm^{-1}; LRMS (ESI): m/z calcd for $C_{20}H_{19}BrN_3O_2$ $[M+H]^+$: 412.07; Found: 412.10.

Compound 3f: Ethyl 2'-((2-iodobenzyl)amino)-[3,3'-bipyridine]-5-carboxylate

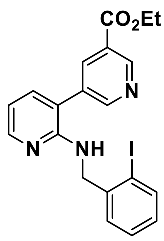

3f

White solid; Yield: 69% (452 mg); ^{1}H NMR (400 MHz, $CDCl_3$): δ 9.21 (d, J = 2.0 Hz, 1H), 8.85 (d, J = 2.0 Hz, 1H), 8.39 (t, J = 2.0 Hz, 1H), 8.21 (dd, J = 5.2, 2.0 Hz, 1H), 7.80 (d, J = 8.0 Hz, 1H), 7.40 (dd, J = 7.6, 1.6 Hz, 1H), 7.34–7.24 (m, 2H), 6.94 (td, J = 7.6, 1.6 Hz, 1H), 6.73 (dd, J = 7.2, 5.2 Hz, 1H), 4.85 (t, J = 6.0 Hz, 1H), 4.64 (d, J = 5.6 Hz, 2H), 4.43 (q, J = 7.2 Hz, 2H), 1.41 (t, J = 7.2 Hz, 3H); ^{13}C NMR (100 MHz, $CDCl_3$): δ 165.02, 155.03, 153.66, 150.24, 148.64, 141.50, 139.59, 137.94, 137.59, 133.70, 129.98, 129.14, 128.40, 126.75, 117.71, 113.57, 99.28, 61.81, 50.32, 14.45; IR: 3414, 1720, 1585, 1499, 1399, 1262, 1108, 766 cm^{-1}; LRMS (ESI): m/z calcd for $C_{20}H_{19}IN_3O_2$ $[M+H]^+$: 460.05; Found: 459.85.

Compound 3g: Ethyl 2'-((4-methoxybenzyl)amino)-[3,3'-bipyridine]-5-carboxylate

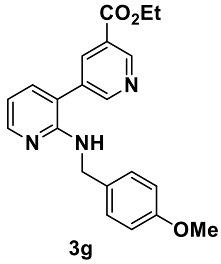

3g

Pale-yellow solid; Yield: 69% (57.2 mg); ^{1}H NMR (500 MHz, $CDCl_3$): δ 9.16 (d, J = 2.0 Hz, 1H), 8.81 (d, J = 2.5 Hz, 1H), 8.36 (t, J = 2.0 Hz, 1H), 8.23 (dd, J = 5.0, 1.5 Hz, 1H), 7.31 (dd, J = 7.0, 2.0 Hz, 1H), 7.25–7.21 (m, 2H), 6.85–6.81 (m, 2H), 6.73 (dd, J = 7.5, 5.0 Hz, 1H), 4.63–4.55 (m, 3H), 4.41 (q, J = 7.0 Hz, 2H), 3.77 (s, 3H), 1.41 (t, J = 7.0 Hz, 3H); ^{13}C NMR (125 MHz, $CDCl_3$): δ 164.96, 158.99, 155.44, 153.51, 150.08, 148.63, 137.86, 137.47, 133.86, 131.62, 129.05 (2C), 126.77, 117.56, 114.14, 113.31 (2C), 61.74, 55.37, 45.30, 14.37; IR: 3128, 1740, 1610, 1511, 1245, 1173, 735 cm^{-1}; LRMS (ESI): m/z calcd for $C_{21}H_{22}N_3O_3$ $[M+H]^+$: 364.17; Found: 364.10.

Compound 3h: Ethyl 2'-((4-chlorobenzyl)amino)-[3,3'-bipyridine]-5-carboxylate

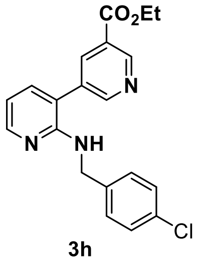

3h

White solid; Yield: 87% (160 mg); ^{1}H NMR (500 MHz, $CDCl_3$): δ 9.17 (d, *J* = 2.0 Hz, 1H), 8.82 (d, *J* = 2.5 Hz, 1H), 8.36 (t, *J* = 2.0 Hz, 1H), 8.20 (dd, *J* = 5.0, 2.0 Hz, 1H), 7.32 (dd, *J* = 7.5, 2.0 Hz, 1H), 7.21–7.28 (m, 4H), 6.74 (dd, *J* = 7.5, 5.0 Hz, 1H), 4.72 (t, *J* = 5.5 Hz, 1H), 4.62 (d, *J* = 5.5Hz, 2H), 4.42 (q, *J* = 7.0 Hz, 2H), 1.41 (t, *J* = 7.0 Hz, 3H); ^{13}C NMR (125 MHz, $CDCl_3$): δ 164.95, 155.21, 153.52, 153.50, 150.18, 150.15, 148.63, 138.31, 137.97, 137.48, 137.46, 133.74, 132.98, 129.03 (2C), 128.78 (2C), 126.82, 113.66, 61.82, 44.97, 14.39; IR: 2923, 1732, 1576, 1488, 1396, 1262, 1091, 765 cm^{-1}; HRMS (ESI+): *m/z* calcd for $C_{20}H_{19}N_3ClO_2^+$ $[M+H]^+$: 368.11603; Found: 368.1162

Compound 3i: Ethyl 2'-(phenylamino)-[3,3'-bipyridine]-5-carboxylate

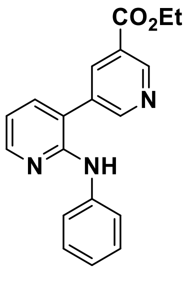

3i

Gummy brown solid; Yield: 94% (60 mg); ^{1}H NMR (500 MHz, $CDCl_3$): δ 9.22 (d, *J* = 2.0 Hz, 1H), 8.87 (d, *J* = 2.0 Hz, 1H), 8.39 (t, *J* = 2.0 Hz, 1H), 8.30 (dd, *J* = 5.0, 2.0 Hz, 1H), 7.46–7.41 (m, 3H), 7.27 (dd, *J* = 9.0, 7.5 Hz, 2H), 7.03–6.96 (m, 1H), 6.90 (dd, *J* = 7.5, 5.0 Hz, 1H), 6.32 (s, 1H), 4.43 (q, *J* = 7.0 Hz, 2H), 1.42 (t, *J* = 7.0 Hz, 3H); ^{13}C NMR (125 MHz, $CDCl_3$): δ 164.90, 153.61, 152.94, 150.39, 148.46, 140.18, 138.67, 137.59, 133.57, 128.97 (2C), 126.85, 122.75, 120.20 (2C), 119.11, 115.63, 61.87, 14.39; IR: 3377, 1709, 1601, 1573, 1436, 1226, 747 cm^{-1}; LRMS (ESI): *m/z* calcd for $C_{19}H_{18}N_3O_2$ $[M+H]^+$: 320.14; Found: 320.15.

Compound 3j: Ethyl 2'-(pyridin-2-ylamino)-[3,3'-bipyridine]-5-carboxylate

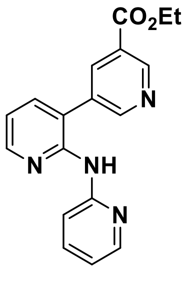

3j

Pale yellow solid; Yield: 97% (62 mg); ^{1}H NMR (500 MHz, $CDCl_3$); δ 9.25 (d, *J* = 5.5 Hz, 1H), 8.91–8.84 (m, 1H), 8.45–8.32 (m, 3H), 8.15–8.05 (m, 1H), 7.65 (d, *J* = 7.0 Hz, 1H), 7.48 (d, *J* = 7.5 Hz, 1H), 7.22 (d, *J* = 10.0 Hz, 1H), 7.03–6.92 (m, 1H), 6.89–6.80 (m, 1H), 4.42 (q, *J* = 7.2Hz, 2H), 1.45–1.35 (m, 3H); ^{13}C NMR (125 MHz, $CDCl_3$) δ 164.79, 153.54, 153.21, 151.45, 150.52, 148.00, 147.74, 138.75, 137.81, 137.59, 133.03, 126.76, 120.07, 117.40, 116.44, 112.73, 61.74, 14.30; IR: 3164, 1745, 1694, 1514, 1431, 1251, 1149 cm^{-1} ; LRMS (ESI): *m/z* calcd for $C_{18}H_{17}N_4O_2$ $[M+H]^+$: 321.14; Found: 321.15.

Compound 3k: Ethyl 2'-(pyrimidin-2-ylamino)-[3,3'-bipyridine]-5-carboxylate

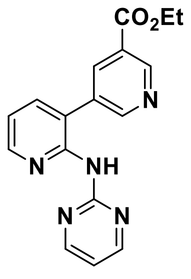

3k

Brown solid; Yield: 96% (62 mg); ^{1}H NMR (400 MHz, $CDCl_3$): δ 9.08 (d, *J* = 1.6 Hz, 1H), 8.86 (d, *J* = 2.0 Hz, 1H), 8.59–8.52 (m, 1H), 8.47 (s, 1H), 8.39 (t, *J* = 2.4 Hz, 1H), 8.25 (d, *J* = 4.8 Hz, 2H), 7.70 (dd, *J* = 7.6, 1.6 Hz, 1H), 7.25 (dd, *J* = 7.6, 4.8 Hz, 1H), 6.63 (t, *J* = 4.8 Hz, 1H), 4.39 (q, *J* = 7.2 Hz, 2H), 1.38 (t, *J* = 7.2 Hz, 3H); ^{13}C NMR (125 MHz, $CDCl_3$): δ 165.06, 159.14, 157.89, 152.86, 150.16, 149.63, 148.78, 139.44, 136.50, 136.49, 134.75, 126.14, 126.00, 120.33, 113.70, 61.61, 14.32; IR: 3211, 1715, 1564, 1520, 1395, 1252, 797 cm^{-1}; HRMS (ESI+): *m/z* calcd for $C_{17}H_{16}N_5O_2^+$ $[M+H]^+$: 322.12985; Found: 322.1298.

Compound 3l: Ethyl 2'-((thiophen-2-ylmethyl)amino)-[3,3'-bipyridine]-5-carboxylate

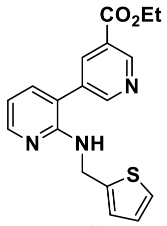

3l

Pale yellow solid; Yield: 95% (161 mg); ^{1}H NMR (400 MHz, $CDCl_3$): δ 9.19 (d, *J* = 3.2 Hz, 1H), 8.82 (s, 1H), 8.37 (s, 1H), 8.25 (d, *J* = 4.8 Hz, 1H), 7.33 (d, *J* = 7.2 Hz, 1H), 7.17 (d, *J* = 4.8 Hz, 1H), 6.99–6.94 (m, 1H), 6.91 (t, *J* = 4.4 Hz, 1H), 6.76 (t, *J* = 6.0 Hz, 1H), 4.83 (d, *J* = 5.2 Hz, 2H), 4.76–4.60 (m, 1H), 4.43 (q, *J* = 7.2 Hz, 2H), 1.42 (t, *J* = 7.2 Hz, 3H); ^{13}C NMR (100 MHz, CDCl3): δ 165.01, 154.87, 153.57, 150.21, 148.53, 142.78, 138.00, 137.50, 133.64, 126.84, 126.76, 125.48, 124.82, 117.81, 113.84, 61.81, 40.67, 14.42; IR: 374, 1713, 1577, 1367, 1222, 765 cm^{-1}; HRMS (ESI+): *m/z* calcd for $C_{18}H_{18}N_3O_2S^+$ $[M+H]^+$: 340.11142; Found: 340.1117.

Compound 3m: Ethyl 2'-(methylamino)-[3,3'-bipyridine]-5-carboxylate

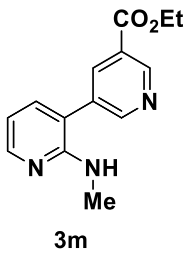

3m

White solid; Yield: 60% (31 mg); ^{1}H NMR (400 MHz, $CDCl_3$): δ 9.19 (s, 1H), 8.80 (s, 1H), 8.34 (t, *J* = 2.0 Hz, 1H), 8.23 (dd, *J* = 5.2, 2.0 Hz, 1H), 7.28 (dd, *J* = 7.2, 1.6 Hz, 1H), 6.69 (dd, *J* = 7.2, 5.2 Hz, 1H), 4.54–4.47 (m, 1H), 4.44 (q, *J* = 7.2 Hz, 2H), 2.97 (d, *J* = 4.8 Hz, 3H), 1.43 (t, *J* = 7.2 Hz, 3H); ^{13}C NMR (100 MHz, $CDCl_3$): δ 165.01, 156.29, 153.63, 150.02, 148.57, 137.55, 137.51, 133.92, 126.67, 117.66, 112.75, 61.78, 28.78, 14.36; IR: 3326, 1717, 1590, 1390, 1261, 763 cm^{-1}; HRMS (ESI+): *m/z* calcd for $C_{14}H_{16}N_3O_2^+$ $[M+H]^+$: 258.12370; Found: 258.1234.

Compound 3n: Ethyl 5-(2-(phenylsulfonamido)phenyl)nicotinate

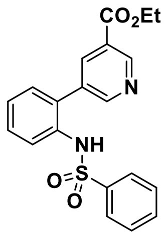

3n

Brown solid; Yield: 95% (avg); ^{1}H NMR (500 MHz, $CDCl_3$): δ 9.12 (s, 1H), 8.29 (s, 1H), 7.89 (s, 1H), 7.68 (d, *J* = 8.0 Hz, 1H), 7.58–7.48 (m, 3H), 7.43 (td, *J* = 7.5, 1.5 Hz, 1H), 7.38 (t, *J* = 7.5 Hz, 2H), 7.31–7.25 (m, 1H), 7.13 (dd, *J* = 7.5, 1.5 Hz, 1H), 7.01 (s, 1H), 4.43 (q, *J* = 7.0 Hz, 2H), 1.43 (t, *J* = 7.0 Hz, 3H); ^{13}C NMR (125 MHz, $CDCl_3$): δ 164.81, 152.98, 149.93, 139.20, 137.43, 133.70, 133.21, 131.67, 130.85, 130.00, 129.22 (2C), 127.06 (2C), 126.57, 124.97, 61.85, 14.41; IR: 3232, 1752, 1640, 1583, 1284, 784 cm^{-1}: HRMS (ESI+): *m/z* calcd for $C_{20}H_{18}N_2NaO_4S^+$ [M+Na]$^+$: 405.08795; Found: 405.0881

Compound 3o: Ethyl 5-(2-(benzylamino)phenyl)nicotinate

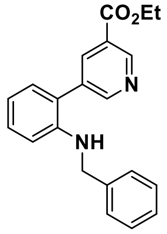

3o

Orange solid; Yield: 96% (avg); ^{1}H NMR (500 MHz, $CDCl_3$): δ 9.18 (d, *J* = 2.0 Hz, 1H), 8.88 (d, *J* = 2.0 Hz, 1H), 8.43 (s, 1H), 7.33–7.29 (m, 4H), 7.28–7.23 (m, 2H), 7.09 (dd, *J* = 7.5, 1.5 Hz, 1H), 6.83 (td, *J* = 7.5, 1.0 Hz, 1H), 6.72 (dd, *J* = 8.5, 1.0 Hz, 1H), 4.43 (q, *J* = 7.0 Hz, 2H), 4.33 (s, 2H), 1.42 (t, *J* = 7.0 Hz, 3H); ^{13}C NMR (125 MHz, $CDCl_3$): δ 165.26, 154.03, 149.67, 145.14, 138.99, 137.94, 135.21, 130.65, 130.13, 128.83 (2C), 127.43, 127.30 (2C), 126.60, 122.95, 117.91, 111.52, 61.67, 48.31, 14.41; IR: 3022, 1717, 1604, 1512, 1249, 746 cm^{-1}; HRMS (ESI+): *m/z* calcd for $C_{22}H_{21}N_2O_2^+$ [M+H]$^+$: 333.15975; Found: 333.1599

Compound 3p: Ethyl 5-(2-((thiophen-2-ylmethyl)amino)phenyl)nicotinate

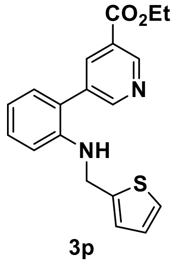

3p

Yellow solid; Yield: 77% (52 mg); ^{1}H NMR (400 MHz, $CDCl_3$): δ 9.17 (d, *J* = 2.0 Hz, 1H), 8.85 (d, *J* = 2.0 Hz, 1H), 8.41 (t, *J* = 2.0 Hz, 1H), 7.32–7.26 (m, 1H), 7.18 (d, *J* = 5.2 Hz, 1H), 7.10 (d, *J* = 6.0 Hz, 1H), 6.98–6.90 (m, 2H), 6.88–6.79 (m, 2H), 4.50 (s, 2H), 4.42 (q, *J* = 7.2 Hz, 2H), 4.15 (s, 1H), 1.41 (t, *J* = 7.2 Hz, 3H); ^{13}C NMR (100 MHz, $CDCl_3$): δ 165.20, 153.99, 149.67, 144.56, 142.71, 137.90, 134.99, 130.67, 130.05, 126.97, 126.50, 124.97, 124.71, 123.30, 118.35, 111.66, 61.63, 43.45, 14.39; IR: 3216, 1744, 1617, 1528, 1250, 7489 cm^{-1}; LRMS (ESI): m/z calcd for $C_{19}H_{19}N_2O_2S$ [M+H]$^+$: 339.12; Found: 339.10.

Compound 3q: Ethyl 5-(2-methoxy-6-(phenylsulfonamido)phenyl)nicotinate

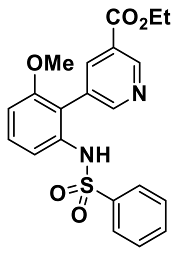

3q

Yellow solid; Yield: 68% (56 mg); ^{1}H NMR (400 MHz, $CDCl_3$): δ 9.12 (d, *J* = 2.0 Hz, 1H), 8.07 (d, *J* = 2.0 Hz, 1H), 7.79 (t, *J* = 2.0 Hz, 1H), 7.63–7.52 (m, 3H), 7.47–7.40 (m, 2H), 7.39–7.32 (m, 2H), 6.78 (dd, *J* = 6.4, 3.2 Hz, 1H), 6.57 (brs, 1H), 4.43 (q, *J* = 7.2 Hz, 2H), 3.64 (s, 3H), 1.43 (t, *J* = 7.2 Hz, 3H); ^{13}C NMR (100 MHz, $CDCl_3$): δ 164.90, 157.42, 154.47, 150.13, 139.09, 139.00, 135.32, 133.33, 130.44, 129.29 (2C), 129.23, 127.11 (2C), 126.40, 119.05, 115.16, 108.03, 61.74, 55.86, 14.43; IR: 3248, 1740, 1607, 1529, 1240, 721 cm^{-1}; LRMS (ESI): *m/z* calcd for $C_{21}H_{21}N_2O_5S$ $[M+H]^+$: 413.12; Found: 413.05.

Compound 3r: Ethyl 5-(5-methoxy-2-(phenylsulfonamido)phenyl)nicotinate

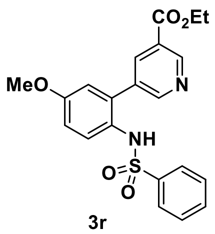

3r

Yellow solid; Yield: 95% (78 mg); ^{1}H NMR (400 MHz, $CDCl_3$): δ 9.07 (s, 1H), 8.34 (d, *J* = 1.2 Hz,1H), 7.84 (t, *J* = 2.0 Hz, 1H), 7.50 (d, *J* = 8.8 Hz, 1H), 7.48–7.42 (m, 2H), 7.42–7.37 (m, 2H), 7.33–7.25 (m, 2H), 6.96 (dd, *J* = 9.2, 3.2 Hz, 1H), 6.68 (d, *J* = 2.8 Hz, 1H), 4.42 (q, *J* = 7.2 Hz, 2H), 3.81 (s, 3H), 1.43 (t, *J* = 7.2 Hz, 3H); ^{13}C NMR (100 MHz, $CDCl_3$): δ 164.81, 158.64, 152.68, 149.39, 139.32, 137.30, 135.58, 134.03, 132.74, 129.96, 128.96 (2C), 126.92 (2C), 126.11, 125.71, 115.95, 114.94, 61.77, 55.67, 14.37; IR: 3258, 1719, 1604, 1563, 1327, 1214, 1153, 730 cm^{-1}; LRMS (ESI): *m/z* calcd for $C_{21}H_{21}N_2O_5S$ $[M+H]^+$: 413.12; Found: 413.05.

Compound 3s: Ethyl 5-(4-methoxy-2-(phenylsulfonamido)phenyl)nicotinate

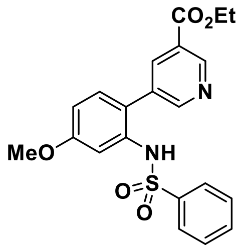

3s

Off-white solid; Yield: 70% (58 mg); ^{1}H NMR (400 MHz, $CDCl_3$): δ 9.08 (d, *J* = 2.4 Hz, 1H), 8.24 (d, *J* = 2.0 Hz, 1H), 7.86 (t, *J* = 2.4 Hz, 1H), 7.65–7.57 (m, 2H), 7.54 (t, *J* = 7.6 Hz, 1H), 7.40 (t, *J* = 7.6 Hz, 2H), 7.29–7.23 (m, 1H), 7.03 (d, *J* = 8.4 Hz, 1H), 6.95 (bs, 1H), 6.80 (dd, *J* = 8.4, 2.4 Hz, 1H), 4.43 (q, *J* = 7.2 Hz, 2H), 3.85 (s, 3H), 1.43 (t, *J* = 7.2 Hz, 3H); ^{13}C NMR (100 MHz, $CDCl_3$): δ 164.84, 160.72, 153.31, 149.70, 139.12, 137.60, 134.79, 133.42, 133.30, 131.60, 129.27 (2C), 127.12 (2C), 126.33, 123.31, 112.50, 109.43, 61.83, 55.70, 14.42; IR: 3228, 1740, 1611, 1517, 1331, 1158, 725 cm^{-1}; LRMS (ESI): *m/z* calcd for $C_{21}H_{21}N_2O_5S$ $[M+H]^+$: 413.12; Found: 413.05.

Compound 3t: Ethyl 5-(3-methoxy-2-(phenylsulfonamido)phenyl)nicotinate

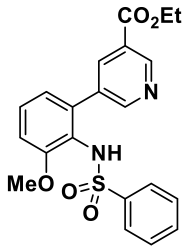

3t

Yellow solid; Yield: 85% (70 mg); ^{1}H NMR (400 MHz, $CDCl_3$): δ 9.08 (d, *J* = 2.0 Hz, 1H), 8.87 (d, *J* = 2.4 Hz, 1H), 8.34 (t, *J* = 2.0 Hz, 1H), 7.50–7.39 (m, 3H), 7.36–7.25 (m, 3H), 6.97 (dd, *J* = 7.6, 1.2 Hz, 1H), 6.79 (d, *J* = 8.4, 1.2 Hz, 1H), 6.74 (s, 1H), 4.42 (q, *J* = 7.2 Hz, 2H), 3.44 (s, 3H), 1.43 (t, *J* = 7.2 Hz, 3H); ^{13}C NMR (100 MHz, $CDCl_3$): δ 165.38, 155.09, 153.67, 149.21, 139.76, 137.70, 137.37, 135.26, 132.50, 128.96, 128.41 (2C), 127.24 (2C), 125.80, 123.07, 122.63, 110.97, 61.49, 55.60, 14.44; IR: 3229, 1744, 1627, 1535, 1251, 721 cm^{-1}; HRMS (ESI+): *m/z* calcd for $C_{21}H_{21}N_2O_5S^+$ $[M+H]^+$: 413.11657; Found: 413.1166.

Compound 3u: Ethyl 5-(2-nitro-6-(phenylsulfonamido)phenyl)nicotinate

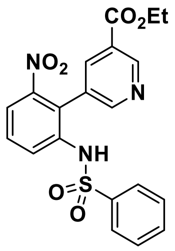

3u

Yellow solid; Yield: 94% (80 mg); ^{1}H NMR (500 MHz, $CDCl_3$): δ 9.24 (d, *J* = 2.5 Hz, 1H), 8.10 (dd, *J* = 8.5, 1.5 Hz, 1H), 8.03 (d, *J* = 2.5 Hz, 1H), 7.83 (dd, *J* = 8.0, 1.0 Hz, 1H), 7.79 (t, *J* = 2.0 Hz, 1H), 7.66–7.61 (m, 2H), 7.57 (dd, *J* = 8.5, 1.5 Hz, 2H), 7.51–7.47 (m, 2H), 6.36 (s, 1H), 4.45 (q, *J* = 7.0 Hz, 2H), 1.44 (t, *J* = 7.0 Hz, 3H); ^{13}C NMR (125 MHz, $CDCl_3$): δ 164.30, 152.55, 151.46, 149.76, 138.50, 137.49, 136.67, 134.10, 130.56, 129.73 (2C), 128.05, 127.06 (2C), 126.88, 126.73, 124.06, 121.26, 62.11, 14.43; IR: 3074, 1740, 1629, 1531, 1246, 808 cm^{-1}; LRMS (ESI): *m/z* calcd for $C_{20}H_{18}N_3O_6S$ $[M+H]^+$: 428.09; Found: 428.05.

Compound 3v: Ethyl 5-(5-nitro-2-(phenylsulfonamido)phenyl)nicotinate

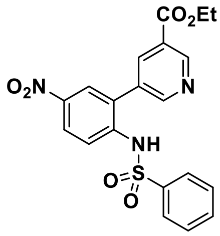

3v

Yellow solid; Yield: 92% (79 mg); ^{1}H NMR (400 MHz, DMSO-d_6): δ 10.44 (brs, 1H), 9.14–9.06 (m, 1H), 8.71–8.62 (m, 1H), 8.25 (dd, *J* = 9.2, 2.8 Hz, 1H), 8.15 (d, *J* = 2.4 Hz, 1H), 8.13 (t, *J* = 2.0 Hz, 1H), 7.74–7.69 (m, 2H), 7.69–7.63 (m, 1H), 7.60–7.54 (m, 2H), 7.51 (d, *J* = 8.8 Hz, 1H), 4.41 (q, *J* = 7.2Hz, 2H), 1.37 (t, *J* = 7.2 Hz, 3H); ^{13}C NMR (100 MHz, DMSO-d_6): δ 164.51, 153.36, 149.21, 144.14, 139.66, 137.35, 133.26, 132.57, 132.05, 129.48 (2C), 126.51, 126.45 (2C), 125.56, 124.70, 123.88, 61.34, 14.12; IR: 3119, 1756, 1661, 1533, 1286, 808 cm^{-1}; HRMS (ESI+): *m/z* calcd for $C_{20}H_{17}N_3NaO_6S^+$ $[M+Na]^+$: 450.07303; Found: 450.0731.

Compound 3w: Ethyl 5-(4-nitro-2-(phenylsulfonamido)phenyl)nicotinate

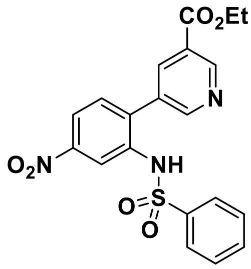

3w

Yellow solid; Yield: 70% (60 mg); ^{1}H NMR (400 MHz, CDCl$_3$) δ 9.11 (d, *J* = 2.0 Hz, 1H), 8.50 (d, *J* = 2.0 Hz, 1H), 8.43 (d, *J* = 2.4 Hz, 1H), 8.07 (dd, *J* = 8.4, 2.4 Hz, 1H), 8.04 (t, *J* = 2.0 Hz, 1H), 7.75–7.62 (m, 2H), 7.65–7.52 (m, 2H), 7.49 (t, *J* = 7.6 Hz, 2H), 7.34 (d, *J* = 8.4 Hz, 1H), 4.44 (q, *J* = 7.2 Hz, 2H), 1.44 (t, *J* = 7.2 Hz, 3H); ^{13}C NMR (100 MHz, CDCl$_3$) δ 164.39, 152.59, 150.89, 148.71, 138.76, 137.46, 136.28, 135.62, 133.86, 131.90, 131.85, 129.65 (2C), 127.23 (2C), 120.31, 117.92, 62.15, 14.38; IR: 3086, 1717, 1626, 1527, 1351, 1173, 587 cm^{-1}; LRMS (ESI): *m/z* calcd for $C_{20}H_{18}N_3O_6S$ [M+H]$^+$: 428.09; Found: 428.10.

Compound 3w′: Ethyl (E)-5-(2-(*N*-(3-ethoxy-3-oxoprop-1-en-1-yl)phenylsulfonamido)-4-nitrophenyl)nicotinate

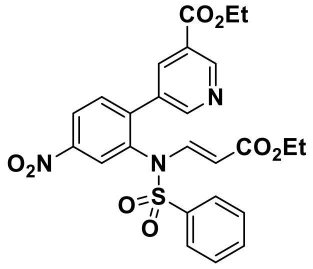

3w'

Gummy brown solid; Yield: 8% (8 mg); ^{1}H NMR (400 MHz, CDCl$_3$): δ 9.26 (d, *J* = 2.4 Hz, 1H), 8.74 (d, *J* = 2.4 Hz, 1H), 8.43 (dd, *J* = 8.4, 2.4 Hz, 1H), 8.33 (t, *J* = 2.0 Hz, 1H), 8.23 (d, *J* = 14.0 Hz, 1H), 7.74 (d, *J* = 8.4 Hz, 1H), 7.69 (t, *J* = 7.2 Hz, 1H), 7.64–7.60 (m, 2H), 7.57–7.49 (m, 3H), 4.74 (d, *J* = 14.0 Hz, 1H), 4.43 (q, *J* = 7.2 Hz, 2H), 4.24–4.11 (m, 2H), 1.42 (t, *J* = 7.2 Hz, 3H), 1.27 (t, *J* = 7.2 Hz, 3H); ^{13}C NMR (100 MHz, CDCl$_3$): δ 166.03, 164.63, 152.13, 151.32, 148.42, 145.80, 143.89, 137.15, 137.02, 134.82, 134.56, 133.11, 131.96, 129.97 (2C), 127.80 (2C), 126.29, 125.57, 125.50, 102.62, 61.94, 60.78, 14.40, 14.33; IR: 3218, 1735, 1624, 1524, 1346, 1249, 746 cm^{-1}; LRMS (ESI): *m/z* calcd for $C_{25}H_{24}N_3O_8S$ [M+H]$^+$: 526.13; Found: 526.05.

Compound 3x: Ethyl 5-(2-bromo-6-(phenylsulfonamido)phenyl)nicotinate

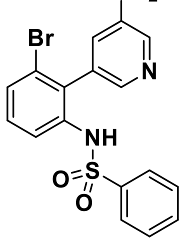

3x

Brown solid; Yield: 65% (60 mg); ^{1}H NMR (400 MHz, DMSO-*d$_6$*): δ 9.82 (s, 1H), 9.06 (s, 1H), 8.32 (s, 1H), 7.76 (s, 1H), 7.70–7.58 (m, 2H), 7.55–7.48 (m, 3H), 7.36 (t, *J* = 8.0 Hz, 1H), 7.23 (d, *J* = 8.0 Hz, 1H), 4.39 (q, *J* = 7.2 Hz, 2H), 1.36 (t, *J* = 7.2 Hz, 3H); ^{13}C NMR (100 MHz, DMSO-*d$_6$*): δ 164.46, 154.00, 149.05, 140.06, 138.11, 136.09, 134.74, 132.86, 130.81, 130.74, 129.29 (2C), 126.27 (2C), 125.96, 124.01, 61.32, 14.10; IR: 3120, 1751, 1634, 1537, 1335, 1286, 784 cm^{-1}; HRMS (ESI+): *m/z* calcd for $C_{20}H_{18}BrN_2O_4S^+$ [M+H]$^+$: 461.01652; Found: 461.0165

Compound 3y: Ethyl 5-(5-bromo-2-(phenylsulfonamido)phenyl)nicotinate

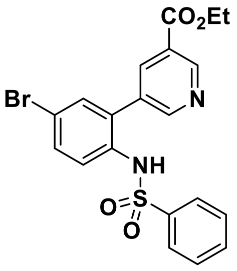

3y

Pale yellow solid; Yield: 70% (65 mg), ^{1}H NMR (400 MHz, $CDCl_3$): δ 9.15 (d, *J* = 2.0 Hz, 1H), 8.22 (d, *J* = 2.0 Hz, 1H), 7.85 (t, *J* = 2.0 Hz, 1H), 7.61–7.51 (m, 5H), 7.45–7.39 (m, 2H), 7.28 (d, *J* = 2.4 Hz, 1H), 6.70 (s, 1H), 4.45 (q, *J* = 7.2 Hz, 2H), 1.44 (t, *J* = 7.2 Hz, 3H); ^{13}C NMR (100 MHz, $CDCl_3$): δ 164.57, 152.77, 150.43, 138.93, 137.38, 133.49, 133.46, 133.34, 132.99, 132.95, 132.28, 129.39 (2C), 127.05 (2C), 126.45, 126.43, 119.79, 61.99, 14.43; IR: 2924, 1739, 1624, 1536, 1378, 1248, 1163, 737 cm^{-1}; LRMS (ESI): *m/z* calcd for $C_{20}H_{18}BrN_2O_4S$ $[M+H]^+$: 461.02; Found: 461.00.

Compound 3z: Ethyl 5-(4-bromo-2-(phenylsulfonamido)phenyl)nicotinate

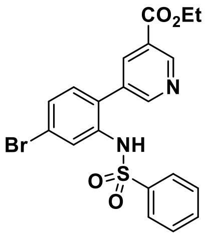

3z

Colourless liquid; Yield: 82% (76 mg), ^{1}H NMR (500 MHz, $CDCl_3$): δ 9.16 (d, *J* = 2.0 Hz, 1H), 8.28 (d, *J* = 2.5 Hz, 1H), 7.97 (t, *J* = 2.0 Hz, 1H), 7.83 (d, *J* = 2.0 Hz, 1H), 7.62–7.55 (m, 3H), 7.46–7.42 (m, 2H), 7.42–7.40 (m, 1H), 7.01 (d, *J* = 8.0 Hz, 1H), 6.77 (s, 1H), 4.45 (q, *J* = 7.0 Hz, 2H), 1.44 (t, *J* = 7.0 Hz, 3H); ^{13}C NMR (125 MHz, $CDCl_3$): δ 164.36, 152.14, 149.62, 138.76, 138.11, 135.00, 133.66, 133.08, 131.94, 129.83, 129.66, 129.48 (2C), 127.34, 127.15 (2C), 126.90, 123.79, 62.15, 14.43; IR: 3068, 1722, 1679, 1563, 1310, 1159, 546 cm^{-1}; LRMS (ESI): *m/z* calcd for $C_{20}H_{18}BrN_2O_4S$ $[M+H]^+$: 461.02; Found: 461.00.

Supplementary Figure 25 Skeletal transformation of *N*-substituted (aza)indole-3-carboxaldehydes to *meta*-aminoaryl nicotinates (**4a–4d**) using propinolamide

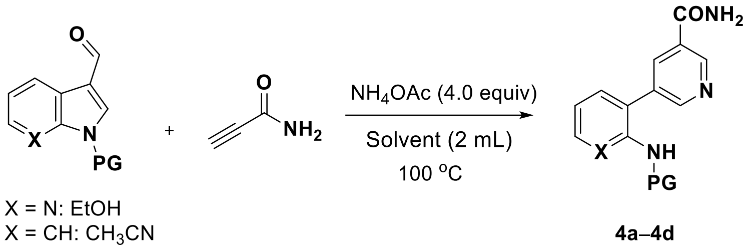

General procedure. A 4-mL vial equipped with a magnetic stir bar and a Teflon-lined screwed cap was charged with **1** (0.2 mmol), propiolamide (1.2 equiv.), and NH_4OAc (61.66 mg, 4 equiv.) in the appropriate solvent (2.0 mL, EtOH or CH_3CN). The vial was then sealed and heated at 100 ℃ for 8 h. Upon reaction completion checked by TLC analysis, the reaction mixture was concentrated under the reduced pressure, added with saturated aqueous $NaHCO_3$, and extracted with DCM (3 × 10 mL). The combined organic fractions were dried over anhydrous Na_2SO_4(s), filtered, and concentrated under the reduced pressure. The crude compound was purified by silica-gel flash column chromatography to obtain the desired product bearing the nicotinamide (**4**) scaffolds.

Compound 4a: 2'-(Phenylsulfonamido)-[3,3'-bipyridine]-5-carboxamide

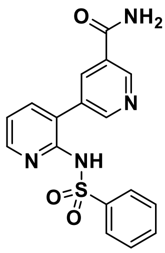

4a

Off-white solid; Yield: 59% (42 mg); ^{1}H NMR (500 MHz, DMSO-d_6) δ 9.01 (d, J = 2.0 Hz, 1H), 8.78 (d, J = 2.0 Hz, 1H), 8.31 (t, J = 2.0 Hz, 1H), 8.21 (s, 1H), 8.04 (s, 1H), 7.91–7.81 (m, 3H), 7.66 (s, 1H), 7.60–7.45 (m, 3H), 7.02 (s, 1H); ^{13}C NMR (125 MHz, DMSO-d_6) δ 166.37, 154.06, 151.84, 147.65, 142.88, 141.34, 136.02, 135.81, 132.19, 131.94, 129.32, 128.82 (2C), 128.67, 126.29 (2C), 112.65; IR: 3206, 1717, 1635, 1582, 1253, 1107, 756 cm^{-1}; HRMS (ESI+): m/z calcd for $C_{17}H_{14}N_4NaO_3S^+$ [M+Na]$^+$: 377.06788; Found: 377.0680.

Compound 4b: 2'-(Benzylamino)-[3,3'-bipyridine]-5-carboxamide

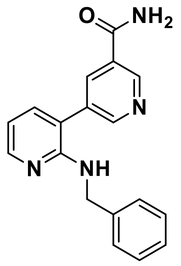

4b

Off-white solid; Yield: 59% (36 mg); ^{1}H NMR (400 MHz, DMSO-d_6): δ 9.03 (d, J = 2.0 Hz, 1H), 8.75 (d, J = 2.0 Hz, 1H), 8.29 (t, J = 2.0 Hz, 1H), 8.19 (s, 1H), 8.03 (dd, J = 4.8, 1.6 Hz, 1H), 7.64 (s, 1H), 7.38 (dd, J = 7.2, 2.0 Hz, 1H), 7.33–7.24 (m, 4H), 7.20–7.14 (m, 1H), 6.69–6.63 (m, 2H), 4.53 (d, J = 6.0 Hz, 2H); ^{13}C NMR (100 MHz, DMSO-d_6): δ 166.34, 155.38, 151.73, 151.70, 147.68, 141.11, 137.89, 135.49, 133.28, 129.66, 128.00 (2C), 126.94 (2C), 126.16, 117.54, 112.26, 43.94; IR: 3171, 1755, 1677, 1529, 1372, 1183, 808 cm^{-1}; LRMS (ESI): *m/z* calcd for $C_{18}H_{17}N_4O$ [M+H]$^+$: 305.14; Found: 305.10.

Compound 4c: 5-(2-(Phenylsulfonamido)phenyl)nicotinamide

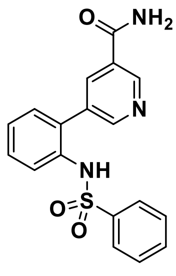

4c

White solid; Yield: 69% (49 mg); ^{1}H NMR (400 MHz, DMSO-d_6): δ 9.82 (s, 1H), 8.95 (d, J = 2.0 Hz, 1H), 8.52 (d, J = 2.4 Hz, 1H), 8.15 (s, 1H), 8.06 (t, J = 2.4 Hz, 1H), 7.63 (s, 1H), 7.59–7.54 (m, 1H), 7.54–7.49 (m, 2H), 7.48–7.41 (m, 2H), 7.39–7.30 (m, 3H), 7.11–7.02 (m, 1H); ^{13}C NMR (100 MHz, DMSO-d_6): δ 166.33, 151.59, 147.12, 147.09, 140.24, 135.55, 135.27, 133.98, 133.54, 132.57, 131.12, 129.08, 129.06 (2C), 127.46, 127.28, 126.32 (2C); IR: 3171, 1766, 1672, 1537, 1157, 746 cm^{-1}; LRMS (ESI): *m/z* calcd for $C_{18}H_{16}N_3O_3S$ [M+H]$^+$: 354.09; Found: 354.10.

Compound 4d: 5-(2-(Benzylamino)phenyl)nicotinamide

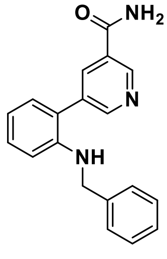

4d

Off-white solid; Yield: 62% (37 mg); ^{1}H NMR (400 MHz, DMSO-d_6) δ 9.01 (d, J = 2.4 Hz, 1H), 8.75 (d, J = 2.0 Hz, 1H), 8.30 (t, J = 2.0 Hz, 1H), 8.20 (s, 1H), 7.62 (s, 1H), 7.39–7.26 (m, 4H), 7.24–7.17 (m, 1H), 7.14–7.07 (m, 1H), 7.04 (dd, J = 7.6, 1.6 Hz, 1H), 6.66 (t, J = 7.6 Hz, 1H), 6.52 (d, J = 8.0 Hz, 1H), 5.73 (t, J = 6.0 Hz, 1H), 4.29 (d, J = 5.6 Hz, 2H); ^{13}C NMR (100 MHz, DMSO-d_6) δ 166.46, 151.94, 147.30, 145.27, 140.17, 135.63, 134.79, 130.52, 129.59, 129.29, 128.29 (2C), 126.77 (2C), 126.51, 122.80, 116.33, 111.00, 46.36; IR: 3179, 1761, 1677, 1527, 1216, 1183, 807 cm^{-1}; HRMS (ESI+): *m/z* calcd for $C_{19}H_{18}N_3O^+$ [M+H]$^+$: 304.14444; Found: 304.1443.

Supplementary Figure 26 Skeletal transformation of (aza)indole-3-carboxaldehydes to *meta*-aminoaryl nicotinates (**6a–6v**) using propiolate

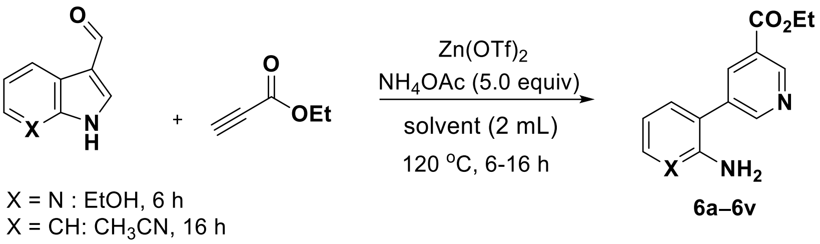

General procedure. A 4-mL vial equipped with a magnetic stir bar and a Teflon-lined screwed cap was charged with **5** (0.2 mmol), ethyl propiolate **2** (1.2 equiv.), NH_4OAc (77.08 mg, 5 equiv.), and $Zn(OTf)_2$ (7.27 mg, 0.1 equiv.) in the appropriate solvent (2.0 mL, EtOH or CH_3CN). The vial was then sealed and heated at 120 ℃ for the desired times (6–16 h). Upon reaction completion checked by TLC analysis, the reaction mixture was concentrated under the reduced pressure, added with saturated aqueous $NaHCO_3$, and extracted with DCM (3 × 10 mL). The combined organic fractions were dried over anhydrous Na_2SO_4(s), filtered, and concentrated under the reduced pressure. The crude compound was purified by silica-gel flash column chromatography to obtain the desired products bearing the nicotinate scaffolds (**6**).

Compound 6a: Ethyl 2'-amino-[3,3'-bipyridine]-5-carboxylate

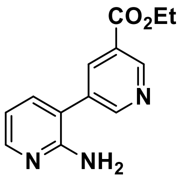

6a

Off-white solid; Yield: 90% (avg); ^{1}H NMR (400 MHz, $CDCl_3$): δ 9.20 (d, *J* = 2.0 Hz, 1H), 8.87 (d, *J* = 2.2 Hz, 1H), 8.41 (t, *J* = 2.2 Hz, 1H), 8.13 (dd, *J* = 5.2, 2.0 Hz, 1H), 7.38 (dd, *J* = 7.6, 1.6 Hz, 1H), 6.80 (dd, *J* = 7.4, 5.2 Hz, 1H), 4.73 (s, 2H), 4.44 (q, *J* = 7.2 Hz, 2H), 1.43 (t, *J* = 7.2 Hz, 3H); ^{13}C NMR (100 MHz, $CDCl_3$): δ 165.02, 155.98, 153.33, 150.02, 148.78, 138.43, 137.13, 133.95, 126.61, 117.15, 114.81, 61.78, 14.36; IR: 3195, 1716, 1624, 1571, 1258, 765 cm^{-1}; HRMS (ESI+): *m/z* calcd for $C_{13}H_{14}N_3O_2^+$ [M+H]$^+$: 244.10805; Found: 244.1080.

Compound 6b: Ethyl 2'-amino-4'-chloro-[3,3'-bipyridine]-5-carboxylate

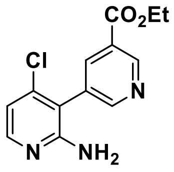
6b

Yellow gummy liquid; Yield: 63% (35 mg); ^{1}H NMR (400 MHz, $CDCl_3$) δ 9.27 (d, *J* = 2.0 Hz, 1H), 8.74 (d, *J* = 2.4 Hz, 1H), 8.31 (t, *J* = 2.0 Hz, 1H), 8.01 (d, *J* = 5.6 Hz, 1H), 6.85 (d, *J* = 5.6 Hz, 1H), 4.64 (brs, 2H), 4.44 (q, *J* = 7.2 Hz, 2H), 1.43 (t, *J* = 7.2 Hz, 3H); ^{13}C NMR (100 MHz, $CDCl_3$) δ 164.85, 157.44, 154.51, 150.89, 148.71, 144.42, 138.82, 130.43, 126.93, 115.76, 115.49, 61.92, 14.40; IR: 3179, 1719, 1619, 1557, 1228, 764 cm^{-1}; HRMS (ESI+): *m/z* calcd for $C_{13}H_{13}ClN_3O_2^+$ $[M+H]^+$: 278.06908; Found: 278.0693.

Compound 6c: Ethyl 3'-amino-[3,4'-bipyridine]-5-carboxylate

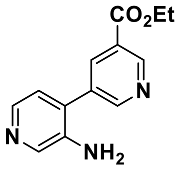
6c

White solid; Yield: 82% (40 mg); ^{1}H NMR (400 MHz, DMSO-d_6): δ 9.09 (d, *J* = 2.0 Hz, 1H), 8.88 (d, *J* = 2.0 Hz, 1H), 8.34 (t, *J* = 2.0 Hz, 1H), 8.15 (s, 1H), 7.87 (d, *J* = 4.8 Hz, 1H), 7.07 (d, *J* = 4.8 Hz, 1H), 5.36 (s, 2H), 4.38 (q, *J* = 7.2 Hz, 2H), 1.35 (t, *J* = 7.2 Hz, 3H); ^{13}C NMR (100 MHz, DMSO-d_6): δ 164.60, 152.91, 148.93, 141.94, 138.28, 137.77, 136.34, 133.11, 126.53, 125.76, 123.96, 61.28, 14.07; IR: 3279, 1691, 1378, 1233, 1032, 755 cm^{-1}; HRMS (ESI+): *m/z* calcd for $C_{13}H_{14}N_3O_2^+$ $[M+H]^+$: 244.10805; Found: 244.1082.

Compound 6d: Ethyl 4'-amino-[3,3'-bipyridine]-5-carboxylate

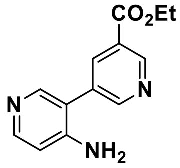
6d

Off-white solid; Yield: quantitative (49 mg); ^{1}H NMR (400 MHz, $CDCl_3$) δ 9.23 (d, *J* = 2.0 Hz, 1H), 8.85 (d, *J* = 2.0 Hz, 1H), 8.39 (t, *J* = 2.0 Hz, 1H), 8.25 (d, *J* = 5.6 Hz, 1H), 8.17 (s, 1H), 6.67 (d, *J* = 5.6 Hz, 1H), 4.44 (q, *J* = 7.2 Hz, 2H), 4.39 (brs, 2H), 1.43 (t, *J* = 7.2 Hz, 3H); ^{13}C NMR (100 MHz, $CDCl_3$) δ 165.02, 153.55, 150.44, 150.36, 150.33, 150.24, 137.55, 131.77, 126.72, 118.26, 110.05, 61.86, 14.38; IR: 3338, 1719, 1644, 1592, 1244, 770 cm^{-1}; HRMS (ESI+): *m/z* calcd for $C_{13}H_{14}N_3O_2^+$ $[M+H]^+$: 244.10805; Found: 244.1080.

Compound 6e: Ethyl 3-amino-[2,3'-bipyridine]-5'-carboxylate

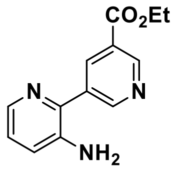

6e

Off-white solid; Yield: 98% (48 mg); ^{1}H NMR (400 MHz, $CDCl_3$): δ 9.21 (d, *J* = 2.0 Hz, 1H), 9.12 (d, *J* = 2.0 Hz, 1H), 8.64 (t, *J* = 2.0 Hz, 1H), 8.16 (dd, *J* = 4.0, 1.6 Hz, 1H), 7.17–7.07 (m, 2H), 4.42 (q, *J* = 7.2 Hz, 2H), 3.96 (s, 2H), 1.41 (t, *J* = 7.2 Hz, 3H); ^{13}C NMR (100 MHz, $CDCl_3$): δ 165.15, 153.23, 150.19, 140.76, 140.65, 140.56, 137.03, 134.45, 126.36, 124.21, 123.48, 61.65, 14.35; IR: 3337, 1717, 1635, 1435, 1253, 756 cm^{-1}; HRMS (ESI+): *m/z* calcd for $C_{13}H_{14}N_3O_2^+$ $[M+H]^+$: 244.10805; Found: 244.1081.

Compound 6f: Ethyl 5-(2-aminophenyl) nicotinate

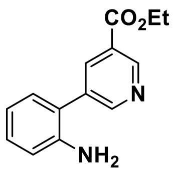

6f

Yellow sticky liquid; Yield: 90% (avg); ^{1}H NMR (400 MHz, $CDCl_3$) δ 9.20 (d, *J* = 2.0 Hz, 1H), 8.88 (d, *J* = 2.4 Hz, 1H), 8.42 (t, *J* = 2.0 Hz, 1H), 7.25–7.20 (m, 1H), 7.12 (dd, *J* = 7.6, 1.6 Hz, 1H), 6.87 (td, *J* = 7.6, 1.2 Hz, 1H), 6.80 (d, *J* = 8.0, 1H), 4.43 (q, *J* = 7.2 Hz, 2H), 3.70 (s, 2H) 1.42 (t, *J* = 7.2 Hz, 3H); ^{13}C NMR (100 MHz, $CDCl_3$) δ 165.36, 153.80, 149.60, 143.85, 137.54, 135.25, 130.75, 129.94, 126.49, 122.79, 119.27, 116.20, 61.72, 14.44; IR: 3364, 1715, 1621, 1575, 1303, 1239, 748 cm^{-1}; HRMS (ESI+): *m/z* calcd for $C_{14}H_{15}N_2O_2^+$ $[M+H]^+$: 243.11280; Found: 243.1128.

Compound 6g: Ethyl 5-(2-amino-6-methoxyphenyl)nicotinate

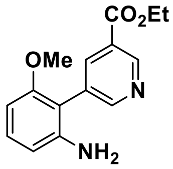

6g

Yellow gummy liquid; Yield: 96% (52 mg); ^{1}H NMR (400 MHz, $CDCl_3$): δ 9.16 (d, *J* = 2.0 Hz, 1H), 8.72 (d, *J* = 2.0 Hz, 1H), 8.30 (t, *J* = 2.0 Hz, 1H), 7.15 (t, *J* = 8.0 Hz, 1H), 6.42 (dd, *J* = 13.6, 8.4 Hz, 2H), 4.41 (q, *J* = 7.2 Hz, 2H), 3.68 (s, 3H), 3.61 (brs, 2H), 1.40 (t, *J* = 7.2 Hz, 3H); ^{13}C NMR (100 MHz, $CDCl_3$): δ 165.37, 157.70, 155.46, 149.35, 145.36, 139.44, 131.08, 130.08, 126.42, 111.28, 108.80, 100.92, 61.48, 55.60, 14.32; IR: 3215, 1716, 1618, 1584, 1239, 766 cm^{-1}; HRMS (ESI+): *m/z* calcd for $C_{15}H_{17}N_2O_3^+$ $[M+H]^+$: 273.12337; Found: 273.1234.

Compound 6h: Ethyl 5-(2-amino-5-methoxyphenyl)nicotinate

Yellow gummy liquid; Yield: 70% (38 mg); ^{1}H NMR (400 MHz, $CDCl_3$) δ 9.20 (d, *J* = 2.0 Hz, 1H), 8.89 (d, *J* = 2.0 Hz, 1H), 8.42 (t, *J* = 2.0 Hz, 1H), 6.83 (dd, *J* = 8.4, 2.8 Hz, 1H), 6.79–6.67 (m, 2H), 4.43 (q, *J* = 7.2 Hz, 2H), 3.78 (s, 3H), 3.48 (brs, 2H), 1.42 (t, *J* = 7.2 Hz, 3H); ^{13}C NMR (100 MHz, $CDCl_3$) δ 165.31, 153.71, 153.18, 149.63, 137.51, 137.49, 135.25, 126.46, 123.89, 117.67, 115.88, 115.85, 61.72, 55.99, 14.42. IR: 3222, 1717, 1613, 1504, 1211, 734 cm^{-1}; LRMS (ESI): *m/z* calcd for $C_{15}H_{17}N_2O_3$ [M+H]$^+$: 273.12; Found: 273.10.

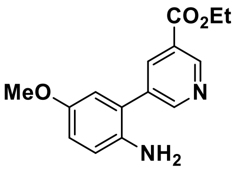

6h

Compound 6i: Ethyl 5-(2-amino-4-methoxyphenyl)nicotinate

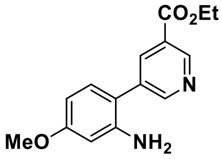

6i

Yellow gummy liquid; Yield: 77% (42 mg); ^{1}H NMR (400 MHz, $CDCl_3$): δ 9.14 (d, *J* = 1.6 Hz, 1H), 8.84 (d, *J* = 2.0 Hz, 1H), 8.38 (t, *J* = 2.0 Hz, 1H), 7.03 (d, *J* = 8.4 Hz, 1H), 6.44 (dd, *J* = 8.4, 2.4 Hz, 1H), 6.34 (d, *J* = 2.4 Hz, 1H), 4.42 (q, *J* = 7.2 Hz, 2H), 3.80 (s, 5H), 1.41 (t, *J* = 7.2 Hz, 3H); ^{13}C NMR (100 MHz, $CDCl_3$): δ 165.36, 161.11, 153.75, 149.00, 145.09, 137.45, 135.12, 131.69, 126.37, 115.76, 105.01, 101.46, 61.62, 55.33, 14.35; IR: 3220, 1717, 1611, 1578, 1240, 734 cm^{-1}; LRMS (ESI): *m/z* calcd for $C_{15}H_{17}N_2O_3$ [M+H]$^+$: 273.12; Found: 273.10.

Compound 6j: Ethyl 5-(2-amino-3-methoxyphenyl)nicotinate

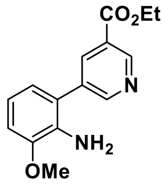

6j

Brown solid; Yield: 79% (43 mg); ^{1}H NMR (400 MHz, $CDCl_3$): δ 9.19 (d, *J* = 2.1 Hz, 1H), 8.90 (d, *J* = 2.4 Hz, 1H), 8.44 (t, *J* = 2.0 Hz, 1H), 6.88–6.80 (m, 2H), 6.77 (dd, *J* = 7.2, 1.6 Hz, 1H), 4.43 (q, *J* = 7.2 Hz, 2H), 3.91 (s, 5H), 1.41 (t, *J* = 7.2 Hz, 3H); ^{13}C NMR (100 MHz, $CDCl_3$): δ 165.34, 153.74, 149.50, 147.48, 137.47, 135.16, 134.03, 126.42, 122.58, 122.48, 118.26, 110.36, 61.65, 55.84, 14.39; IR: 3194, 1718, 1614, 1577, 1250, 732 cm^{-1}; LRMS (ESI): *m/z* calcd for $C_{15}H_{17}N_2O_3$ [M+H]$^+$: 273.12; Found: 273.10.

Compound 6k: Ethyl 5-(2-amino-6-nitrophenyl)nicotinate

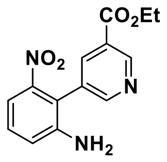

6k

Yellow solid; Yield: 89% (51 mg); ^{1}H NMR (400 MHz, $CDCl_3$): δ 9.24 (d, *J* = 2.0 Hz, 1H), 8.67 (d, *J* = 2.0 Hz, 1H), 8.24 (t, *J* = 2.0 Hz, 1H), 7.43–7.29 (m, 2H), 7.02 (dd, *J* = 8.0, 1.2 Hz, 1H), 4.42 (q, *J* = 7.2 Hz, 2H), 3.84 (s, 2H), 1.41 (t, *J* = 7.2 Hz, 3H); ^{13}C NMR (100 MHz, $CDCl_3$): δ 164.87, 153.58, 150.61, 150.15, 146.34, 138.12, 130.32, 129.97, 126.85, 119.74, 115.87, 113.98, 61.85, 14.35; IR: 3379, 1717, 1627, 1522, 1241, 732 cm^{-1}; LRMS (ESI): *m/z* calcd for $C_{14}H_{14}N_3O_4$ $[M+H]^+$: 288.10; Found: 288.10.

Compound 6l: Ethyl 5-(2-amino-5-nitrophenyl)nicotinate

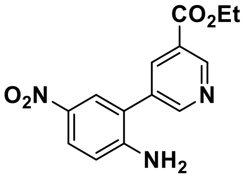

6l

Yellow solid; Yield: 89% (51 mg); ^{1}H NMR (400 MHz, DMSO-d_6): δ 8.31 (s, 1H), 8.04 (dd, *J* = 9.2, 2.4 Hz, 1H), 7.91 (d, *J* = 2.8 Hz, 1H), 6.85 (d, *J* = 9.2 Hz, 1H), 6.62 (s, 2H), 4.39 (q, *J* = 7.2 Hz, 2H), 1.36 (t, *J* = 7.2 Hz, 3H); ^{13}C NMR (100 MHz, DMSO-d_6): δ 165.00, 153.45, 152.98, 148.93, 136.78, 136.28, 127.21, 126.02, 120.00, 114.20 (3C), 61.26, 14.05; IR: 3346, 1723, 1650, 1578, 1302, 1243, 1107 cm^{-1}; LRMS (ESI): *m/z* calcd for $C_{14}H_{14}N_3O_4$ $[M+H]^+$: 288.10; Found: 288.10.

Compound 6m: Ethyl 5-(2-amino-4-nitrophenyl)nicotinate

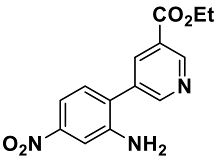

6m

Yellow solid; Yield: 57% (33 mg); ^{1}H NMR (400 MHz, DMSO-d_6) δ 9.10 (d, *J* = 2.0 Hz, 1H), 8.85 (d, *J* = 2.4 Hz, 1H), 8.31 (t, *J* = 2.0 Hz, 1H), 7.65 (d, *J* = 2.4 Hz, 1H), 7.45 (dd, *J* = 8.0, 2.0 Hz, 1H), 7.31 (d, *J* = 8.4 Hz, 1H), 4.38 (q, *J* = 7.2 Hz, 2H), 1.35 (t, *J* = 7.2 Hz, 3H); ^{13}C NMR (100 MHz, DMSO-d_6) δ 164.60, 153.12, 149.03, 148.39, 147.36, 136.66, 133.58, 131.61, 127.01, 125.79, 110.51, 108.94, 61.31, 14.08; IR: 3346, 1723, 1650, 1578, 1302, 1243, 1107 cm^{-1}; HRMS (ESI+): *m/z* calcd for $C_{14}H_{14}N_3O_4^+$ $[M+H]^+$: 288.09788; Found: 288.0982

Compound 6n: Ethyl 5-(2-amino-3-nitrophenyl)nicotinate

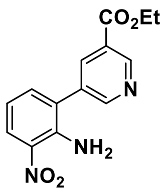

6n

Yellow solid; Yield: 96% (55 mg); ^{1}H NMR (400 MHz, $CDCl_3$): δ 9.25 (d, *J* = 2.0 Hz, 1H), 8.82 (d, *J* = 2.4 Hz, 1H), 8.38 (t, *J* = 2.0 Hz, 1H), 8.23 (dd, *J* = 8.8, 1.6 Hz, 1H), 7.38–7.26 (m, 1H), 6.83 (dd, *J* = 8.4, 7.2 Hz, 1H), 6.22 (s, 2H), 4.45 (q, *J* = 7.2 Hz, 2H), 1.43 (t, *J* = 7.2 Hz, 3H); ^{13}C NMR (100 MHz, $CDCl_3$): δ 164.78, 153.73, 150.75, 142.64, 137.89, 136.98, 133.15, 132.94, 127.10, 126.85, 126.33, 116.65, 61.95, 14.37; IR: 3472, 1723, 1618, 1518, 1261, 744 cm^{-1}; LRMS (ESI): *m/z* calcd for $C_{14}H_{14}N_3O_4$ $[M+H]^+$: 288.10; Found: 288.10.

Compound 6o: Ethyl 5-(2-amino-6-fluorophenyl)nicotinate

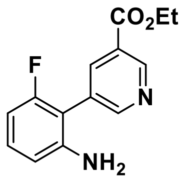

6o

Off-white solid; Yield: 83% (43 mg); ^{1}H NMR (400 MHz, $CDCl_3$): δ 9.23 (d, *J* = 2.0 Hz, 1H), 8.81 (d, *J* = 2.4 Hz, 1H), 8.37 (t, *J* = 2.4 Hz, 1H), 7.21–7.12 (m, 1H), 6.63–6.52 (m, 2H), 4.43 (q, *J* = 7.2 Hz, 2H), 3.72 (s, 2H), 1.42 (t, *J* = 7.2 Hz, 3H); ^{13}C NMR (100 MHz, $CDCl_3$): δ 165.11, 160.76 (d, *J* = 242.9 Hz), 154.77, 150.12, 145.95 (d, *J* = 5.3 Hz), 139.02, 130.48 (d, *J* = 10.6 Hz), 128.66, 126.62, 111.26 (d, *J* = 2.7 Hz), 110.57 (d, *J* = 19.0 Hz), 105.18 (d, *J* = 22.6 Hz), 61.71, 14.35; ^{19}F NMR (376 MHz, $CDCl_3$): δ –115.50 (dd, *J* = 9.4, 6.4 Hz); IR: 3219, 1717, 1626, 1575, 1468, 1240, 1107, 781 cm^{-1}; LRMS (ESI): *m/z* calcd for $C_{14}H_{14}FN_2O_2$ $[M+H]^+$: 261.10; Found: 261.10.

Compound 6p: Ethyl 5-(2-amino-5-fluorophenyl)nicotinate

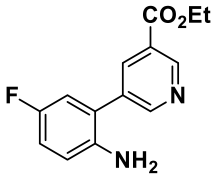

6p

Yellow solid; Yield: 71% (36.8 mg); ^{1}H NMR (400 MHz, $CDCl_3$) δ 9.20 (d, *J* = 2.0 Hz, 1H), 8.86 (d, *J* = 2.0 Hz, 1H), 8.40 (t, *J* = 2.0 Hz, 1H), 6.94 (td, *J* = 8.8, 3.2 Hz, 1H), 6.86 (dd, *J* = 8.8, 2.8 Hz, 1H), 6.74 (dd, *J* = 8.8, 4.8 Hz, 1H), 4.43 (q, *J* = 7.2 Hz, 2H), 3.63 (brs, 2H), 1.42 (t, *J* = 7.2 Hz, 3H); ^{13}C NMR (100 MHz, $CDCl_3$): δ 165.10, 156.44 (d, *J* = 236.1 Hz), 153.46, 149.80, 140.05 (d, *J* = 2.2 Hz), 137.42, 134.36, 126.51, 123.56 (d, *J* = 7.2 Hz), 117.22 (d, *J* = 7.6 Hz), 116.80 (d, *J* = 22.8 Hz), 116.43 (d, *J* = 22.1 Hz), 61.76, 14.35; ^{19}F NMR (376 MHz, $CDCl_3$): δ -126.25 – -126.31 (m) ; IR: 3373, 1719, 1625, 1508, 1243, 1109, 768 cm^{-1}; LRMS (ESI): *m/z* calcd for $C_{14}H_{14}FN_2O_2$ $[M+H]^+$: 261.10; Found: 261.10.

Compound 6q: Ethyl 5-(2-amino-4-fluorophenyl)nicotinate

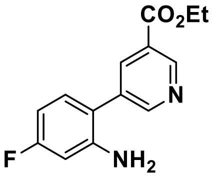

6q

Pale yellow solid; Yield: 93% (48.3 mg); ^{1}H NMR (400 MHz, $CDCl_3$) δ 9.17 (d, *J* = 2.0 Hz, 1H), 8.82 (d, *J* = 2.0 Hz, 1H), 8.37 (t, *J* = 2.0 Hz, 1H), 7.04 (dd, *J* = 8.4, 6.4 Hz, 1H), 6.63–6.44 (m, 2H), 4.43 (q, *J* = 7.2 Hz, 2H), 3.92 (s, 2H), 1.42 (t, *J* = 7.2 Hz, 3H); ^{13}C NMR (100 MHz, $CDCl_3$): δ 165.17, 163.95 (d, *J* = 245.0 Hz), 153.64, 149.45, 145.65 (d, *J* = 11.1 Hz), 137.61, 134.52, 132.04 (d, *J* = 10.0 Hz), 126.50, 118.59 (d, *J* = 2.7 Hz), 105.87 (d, *J* = 21.8 Hz), 102.62 (d, *J* = 24.8 Hz), 61.73, 14.33; ^{19}F NMR (376 MHz, $CDCl_3$): δ -112.50 – -112.62 (m); IR: 3362, 1719, 1631, 1502, 1244, 1108, 767 cm^{-1}; LRMS (ESI): *m/z* calcd for $C_{14}H_{14}FN_2O_2$ $[M+H]^+$: 261.10; Found: 261.10.

Compound 6r: Ethyl 5-(2-amino-3-fluorophenyl)nicotinate

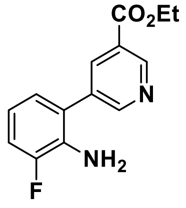

6r

Off-white solid; Yield: 90% (47.06 mg); ^{1}H NMR (500 MHz, $CDCl_3$) δ 9.20 (d, *J* = 2.0 Hz, 1H), 8.88 (d, *J* = 2.0 Hz, 1H), 8.42 (t, *J* = 2.0 Hz, 1H), 7.06 (ddd, *J* = 10.5, 8.0, 1.5 Hz, 1H), 6.92 (dt, *J* = 7.5, 1.5 Hz, 1H), 6.79 (td, *J* = 8.0, 5.0 Hz, 1H), 4.44 (q, *J* = 7.0 Hz, 2H), 3.83 (s, 2H), 1.42 (t, *J* = 7.0 Hz, 3H); ^{13}C NMR (125 MHz, $CDCl_3$): δ 165.16, 153.48, 151.90 (d, *J* = 238.0 Hz), 149.85, 137.36, 134.15, 132.73 (d, *J* = 10.6 Hz), 126.53, 125.78, 124.65, 118.36 (d, *J* = 6.3 Hz), 115.39 (d, *J* = 15.4 Hz), 61.76, 14.38; ^{19}F NMR (376 MHz, $CDCl_3$): δ –131.91 (dd, *J* = 10.9, 5.3 Hz); IR: 3368, 1719, 1630, 1595, 1255, 1107, 765 cm^{-1}; LRMS (ESI): *m/z* calcd for $C_{14}H_{14}FN_2O_2$ $[M+H]^+$: 261.10; Found: 261.10.

Compound 6s: Ethyl 5-(2-amino-6-bromophenyl)nicotinate

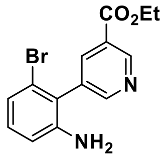

6s

Yellow solid; Yield: 83% (53 mg); ^{1}H NMR (400 MHz, $CDCl_3$): δ 9.24 (d, *J* = 2.4 Hz, 1H), 8.70 (d, *J* = 2.8 Hz, 1H), 8.26 (d, *J* = 2.4 Hz, 1H), 7.12–7.01 (m, 2H), 6.74 (dd, *J* = 7.2, 2.4 Hz, 1H), 4.43 (q, *J* = 7.2 Hz, 2H), 3.64 (s, 2H), 1.42 (t, *J* = 7.2 Hz, 3H); ^{13}C NMR (100 MHz, $CDCl_3$) δ 165.10, 154.85, 150.33, 145.88, 139.05, 133.84, 130.69, 126.65, 124.61, 123.02, 122.53, 114.53, 61.73, 14.37; IR: 3216, 1717, 1626, 1561, 1241, 873 cm^{-1}; LRMS (ESI): *m/z* calcd for $C_{14}H_{14}BrN_2O_2$ $[M+H]^+$: 321.02; Found: 321.00.

Compound 6t: Ethyl 5-(2-amino-5-bromophenyl)nicotinate

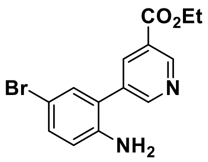

6t

Yellow solid; Yield: 90% (58 mg); ^{1}H NMR (400 MHz, $CDCl_3$) δ 9.18 (d, *J* = 2.0 Hz, 1H), 8.83 (d, *J* = 2.0 Hz, 1H), 8.37 (t, *J* = 2.0 Hz, 1H), 7.29 (dd, *J* = 8.8, 2.4 Hz, 1H), 7.22 (d, *J* = 2.4 Hz, 1H), 6.68 (d, *J* = 8.8 Hz, 1H), 4.43 (q, *J* = 7.2 Hz, 2H), 3.82 (brs, 2H), 1.42 (t, *J* = 7.2 Hz, 3H); ^{13}C NMR (100 MHz, $CDCl_3$) δ 165.04, 153.40, 149.85, 143.03, 137.40, 133.95, 132.86, 132.45, 126.51, 124.33, 117.63, 110.49, 61.76, 14.34; IR: 3366, 1716, 1624, 1489, 1240, 767 cm^{-1}; LRMS (ESI): *m/z* calcd for $C_{14}H_{14}BrN_2O_2$ $[M+H]^+$: 321.02; Found: 321.00.

Compound 6u: Ethyl 5-(2-amino-4-bromophenyl)nicotinate

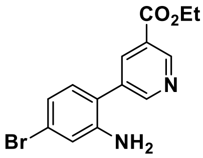

6u

Yellow solid; Yield: 83% (53 mg); ^{1}H NMR (400 MHz, $CDCl_3$): δ 9.18 (d, *J* = 2.0 Hz, 1H), 8.82 (d, *J* = 2.4 Hz, 1H), 8.37 (t, *J* = 2.0 Hz, 1H), 7.00–6.92 (m, 3H), 4.43 (q, *J* = 7.2 Hz, 2H), 3.84 (s, 2H), 1.42 (t, *J* = 7.2 Hz, 3H); ^{13}C NMR (100 MHz, $CDCl_3$): δ 165.14, 153.48, 149.77, 145.19, 137.37, 134.29, 131.91, 126.53, 123.55, 121.99, 121.49, 118.63, 61.77, 14.36; IR: 3216, 1716, 1624, 1489, 1240, 734 cm^{-1}; LRMS (ESI): *m/z* calcd for $C_{14}H_{14}BrN_2O_2$ $[M+H]^+$: 321.02; Found: 321.00.

Compound 6v: Ethyl 5-(2-amino-3-bromophenyl)nicotinate

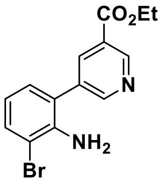

6v

White solid; Yield: 85% (55 mg); ^{1}H NMR (400 MHz, $CDCl_3$) δ 9.20 (d, *J* = 2.0 Hz, 1H), 8.84 (d, *J* = 2.4 Hz, 1H), 8.39 (t, *J* = 2.0 Hz, 1H), 7.48 (dd, *J* = 8.0, 1.6 Hz, 1H), 7.04 (dd, *J* = 7.6, 1.6 Hz, 1H), 6.72 (t, *J* = 8.0 Hz, 1H), 4.43 (q, *J* = 7.2 Hz, 2H), 4.20 (s, 2H), 1.42 (t, *J* = 7.2 Hz, 3H); ^{13}C NMR (100 MHz, $CDCl_3$) δ 165.06, 153.57, 149.95, 141.74, 137.49, 134.80, 133.16, 129.85, 126.53, 123.73, 119.36, 110.31, 61.75, 14.36; IR: 3366, 1720, 1616, 1455, 1252, 733 cm^{-1}; HRMS (ESI+): *m/z* calcd for $C_{14}H_{14}BrN_2O_2^+$ $[M+H]^+$: 321.02332; Found: 321.0235.

Compound 6w: Ethyl 5-(2-amino-6-(trifluoromethyl)phenyl)nicotinate

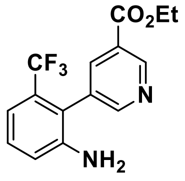

6w

Off-white solid; Yield: 84% (52 mg); ^{1}H NMR (400 MHz, $CDCl_3$): δ 9.28 (d, *J* = 2.0 Hz, 1H), 8.69 (d, *J* = 2.0 Hz, 1H), 8.25 (d, *J* = 2.0 Hz, 1H), 7.32 (t, *J* = 7.6 Hz, 1H), 7.18 (d, *J* = 7.6 Hz, 1H), 6.96 (d, *J* = 8.0 Hz, 1H), 4.43 (q, *J* = 7.2 Hz, 2H), 3.55 (s, 2H), 1.42 (t, *J* = 7.2 Hz, 3H); ^{13}C NMR (125 MHz, $CDCl_3$): δ 165.08, 154.59, 150.65, 145.77, 139.11, 131.30, 130.30 (q, *J* = 29.5 Hz), 129.62, 126.54, 123.94, (q, *J* = 272.4 Hz), 120.11, 118.94, 115.95 (q, *J* = 5.5 Hz),, 61.81, 14.39; ^{19}F NMR (376 MHz, $CDCl_3$): δ –57.71; IR: 3377, 1721, 1626, 1472, 1244, 1091, 768 cm^{-1}; LRMS (ESI): *m/z* calcd for $C_{15}H_{14}F_3N_2O_2$ $[M+H]^+$: 311.10; Found: 311.05.

Compound 6x: Ethyl 5-(2-amino-5-(trifluoromethyl)phenyl)nicotinate

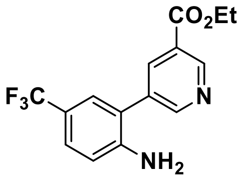

6x

Pale yellow solid; Yield: 83% (51.8 mg); ^{1}H NMR (500 MHz, $CDCl_3$): δ 9.22 (s, 1H), 8.86 (s, 1H), 8.39 (t, *J* = 2.0 Hz, 1H), 7.50–7.42 (m, 1H), 7.35 (d, *J* = 2.0 Hz, 1H), 6.84 (d, *J* = 8.5 Hz, 1H), 4.44 (q, *J* = 7.0 Hz, 2H), 4.10 (s, 2H), 1.43 (t, *J* = 7.0 Hz, 3H); ^{13}C NMR (100 MHz, $CDCl_3$): δ 165.06, 153.51, 150.09, 146.94, 137.57, 133.99, 127.91 (q, *J* = 3.8 Hz), 127.00 (q, *J* = 3.7 Hz), 126.70, 124.62 (q, *J* = 269.4 Hz), 122.02, 120.82 (q, *J* = 32.7 Hz), 115.51, 61.86, 14.35; ^{19}F NMR (376 MHz, $CDCl_3$): δ –61.34; IR: 3375, 1718, 1630, 1515, 1291, 1106, 767 cm^{-1}; LRMS (ESI): *m/z* calcd for $C_{15}H_{14}F_3N_2O_2$ $[M+H]^+$: 311.10; Found: 311.05.

Compound 6y: Ethyl 5-(2-amino-4-(trifluoromethyl)phenyl)nicotinate

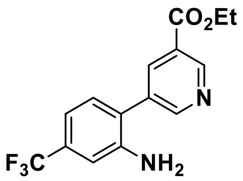

6y

Yellow solid; Yield: 86% (53.1 mg); ^{1}H NMR (400 MHz, $CDCl_3$): δ δ 9.23 (s, 1H), 8.86 (s, 1H), 8.40 (t, *J* = 2.0 Hz, 1H), 7.21 (d, *J* = 8.0 Hz, 1H), 7.09 (dd, *J* = 8.0, 1.6 Hz, 1H), 7.03 (d, *J* = 1.6 Hz, 1H), 4.44 (q, *J* = 7.2 Hz, 2H), 3.95 (s, 2H), 1.43 (t, *J* = 7.2 Hz, 3H); ^{13}C NMR (100 MHz, $CDCl_3$): δ 165.08, 153.42, 150.15, 144.35, 137.43, 134.07, 132.05 (q, *J* = 32.2 Hz), 131.20, 126.66, 125.64, 124.04 (q, *J* = 271.0 Hz), 115.42 (q, *J* = 3.8 Hz), 112.53 (q, *J* = 3.9 Hz), 61.86, 14.37; ^{19}F NMR (376 MHz, $CDCl_3$): δ –63.08; IR: 3374, 1718, 1630, 1579, 1240, 1119, 769 cm^{-1}; LRMS (ESI): *m/z* calcd for $C_{15}H_{14}F_3N_2O_2$ $[M+H]^+$: 311.10; Found: 311.05.

Compound 6z: Ethyl 5-(2-amino-6-(4,4,5,5-tetramethyl-1,3,2-dioxaborolan-2-yl)phenyl)nicotinate

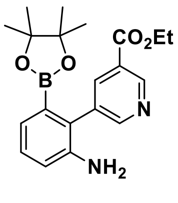
6z

Off-white solid; Yield: 89% (65 mg); ^{1}H NMR (500 MHz, DMSO-d_6) δ 9.04 (d, *J* = 2.0 Hz, 1H), 8.58 (d, *J* = 2.0 Hz, 1H), 8.01 (t, *J* = 2.0 Hz, 1H), 7.15–7.09 (m, 1H), 6.93 (dd, *J* = 7.5, 1.0 Hz, 1H), 6.87 (dd, *J* = 8.0, 1.5 Hz, 1H), 4.76 (s, 2H), 4.36 (q, *J* = 7.0 Hz, 2H), 1.33 (t, *J* = 7.0 Hz, 3H), 1.01 (s, 12H); ^{13}C NMR (125 MHz, DMSO-d_6) δ 164.96, 154.37, 148.03, 145.54, 138.05, 135.48, 128.40, 126.14, 125.10, 122.88, 117.52, 83.04 (2C), 61.07, 24.24 (4C), 14.09 (boron-attached carbon not observed) ; IR: 3436, 1792, 1661, 1306, 1212, 1024, 758 cm^{-1}; LRMS (ESI): *m/z* calcd for $C_{20}H_{26}BN_2O_4$ [M+H]$^+$: 369.20; Found: 369.20.

Compound 6aa: Ethyl 5-(2-amino-5-(4,4,5,5-tetramethyl-1,3,2-dioxaborolan-2-yl)phenyl)nicotinate

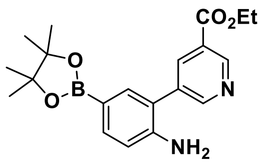
6aa

Off-white solid; Yield: 94% (70 mg); ^{1}H NMR (500 MHz, $CDCl_3$): δ 9.18 (d, *J* = 2.0 Hz, 1H), 8.87 (d, *J* = 2.5 Hz, 1H), 8.40 (t, *J* = 2.0 Hz, 1H), 7.66 (dd, *J* = 8.0, 1.5 Hz, 1H), 7.56 (d, *J* = 1.5 Hz, 1H), 6.78 (d, *J* = 8.0 Hz, 1H), 4.43 (q, *J* = 7.0 Hz, 2H), 3.96 (s, 2H), 1.42 (t, *J* = 7.0 Hz, 3H), 1.33 (s, 12H); ^{13}C NMR (125MHz, $CDCl_3$) δ 165.30, 153.81, 149.50, 146.67, 137.68, 137.67, 136.71, 135.06, 126.44, 121.90, 115.16, 83.64 (2C), 61.66, 24.94 (2C), 14.39 (boron-attached carbon not observed). IR: 3368, 1721, 1624, 1354, 1299, 1141, 769 cm^{-1}; HRMS (ESI+): *m/z* calcd for $C_{20}H_{26}BN_2O_4^+$ [M+H]$^+$: 369.19801; Found: 369.1984.

Compound 6ab: Ethyl 5-(2-amino-4-(4,4,5,5-tetramethyl-1,3,2-dioxaborolan-2-yl)phenyl)nicotinate

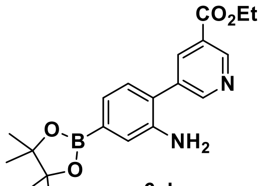
6ab

Compound **6ab** was obtained in a reasonably high yield (>90%) as a crude product, but we observed a lower yield (60%) after silica gel-based purification, probably due to degradation of boronic ester. Pale yellow gummy liquid; Yield: 60% (44 mg); ^{1}H NMR (500 MHz, $CDCl_3$): δ 9.20 (d, *J* = 2.5 Hz, 1H), 8.88 (d, *J* = 2.5 Hz, 1H), 8.42 (t, *J* = 2.0 Hz, 1H), 7.31 (dd, *J* = 7.5, 1.0 Hz, 1H), 7.25 (d, *J* = 1.0 Hz, 1H), 7.14 (d, *J* = 7.5 Hz, 1H), 4.43 (q, *J* = 7.0 Hz, 2H), 3.69 (s, 2H), 1.42 (t, *J* = 7.0 Hz, 3H), 1.36 (s, 12H); ^{13}C NMR (125 MHz, $CDCl_3$): δ 165.32, 153.64, 149.69, 143.26, 137.40, 135.17, 130.12, 126.47, 125.46, 125.44, 122.44, 84.06 (2C), 61.72, 25.00 (4C), 14.42 (boron-attached carbon not observed); IR: 3228, 1721, 1623, 1595, 1357, 1237, 735 cm^{-1}; LRMS (ESI): *m/z* calcd for $C_{20}H_{26}BN_2O_4$ [M+H]$^+$: 369.20; Found: 369.20.

Compound 6ac: Ethyl 5-(2-amino-3-(4,4,5,5-tetramethyl-1,3,2-dioxaborolan-2-yl)phenyl)nicotinate

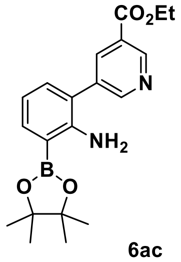

6ac

Compound **6ac** was obtained in a reasonably high yield (>90%) as a crude product, but we observed a lower yield (56%) after silica gel-based purification, probably due to degradation of boronic ester. Pale yellow liquid; Yield: 56% (41 mg); ^{1}H NMR (400 MHz, $CDCl_3$) δ 9.20 (d, *J* = 2.0 Hz, 1H), 8.85 (d, *J* = 2.4 Hz, 1H), 8.40 (t, *J* = 2.0 Hz, 1H), 7.71 (dd, *J* = 7.2, 1.6 Hz, 1H), 7.15 (dd, *J* = 7.2, 1.6 Hz, 1H), 6.79 (t, *J* = 7.6 Hz, 1H), 4.86 (s, 2H), 4.42 (q, *J* = 7.2 Hz, 2H), 1.41 (t, *J* = 7.2 Hz, 3H), 1.36 (s, 12H); ^{13}C NMR (100 MHz, $CDCl_3$) δ 165.34, 154.04, 150.72, 149.61, 137.84, 137.78, 135.53, 134.17, 126.48, 122.20, 117.39, 83.97 (2C), 61.66, 25.06 (4C), 14.43 (boron-attached carbon not observed); IR: 2978, 1722, 1618, 1591, 1360, 1295, 754 cm^{-1}; LRMS (ESI): *m/z* calcd for $C_{20}H_{26}BN_2O_4$ $[M+H]^+$: 369.20; Found: 369.25.

Supplementary Figure 27 Skeletal transformation of (aza)indole-3-carboxaldehydes to *meta*-aminoaryl nicotinates (**7a–7b**) using propiolamide

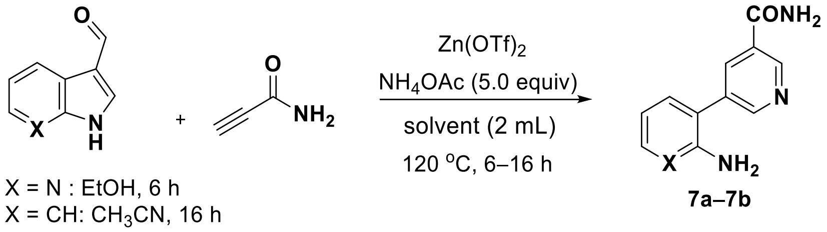

Compound 7a: 2'-Amino-[3,3'-bipyridine]-5-carboxamide

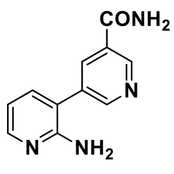

7a

Off-white solid; Yield: 94% (40 mg); ^{1}H NMR (500 MHz, DMSO-*d*$_6$) δ 9.00 (d, *J* = 2.5 Hz, 1H), 8.73 (d, *J* = 2.5 Hz, 1H), 8.28 (t, *J* = 2.5 Hz, 1H), 8.22 (s, 1H), 8.02 (dd, *J* = 5.0, 2.0 Hz, 1H), 7.64 (s, 1H), 7.42 (dd, *J* = 7.5, 2.0 Hz, 1H), 6.70 (dd, *J* = 7.0, 4.5 Hz, 1H), 5.86 (s, 2H); ^{13}C NMR (125 MHz, DMSO-*d*$_6$) δ 166.47, 156.88, 151.47, 148.09, 147.50, 138.22, 134.99, 133.65, 129.59, 116.46, 113.01; IR: 3367, 1737, 1629, 1371, 1231, 704 cm^{-1}; LRMS (ESI): *m/z* calcd for $C_{11}H_{11}N_4O$ $[M+H]^+$: 215.09; Found: 215.10.

Compound 7b: 5-(2-Aminophenyl)nicotinamide

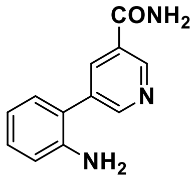

7b

Off-white solid; Yield: 73% (31 mg); ^{1}H NMR (400 MHz, DMSO-d_6): δ 8.96 (d, J = 2.0 Hz, 1H), 8.71 (d, J = 2.0 Hz, 1H), 8.24 (t, J = 2.0 Hz, 1H), 8.18 (s, 1H), 7.60 (s, 1H), 7.11 (td, J = 7.6, 1.6 Hz, 1H), 7.04 (dd, J = 7.6, 1.6 Hz, 1H), 6.79 (d, J = 8.0 Hz, 1H), 6.67 (t, J = 7.6 Hz, 1H), 4.99 (s, 2H); ^{13}C NMR (100 MHz, DMSO-d_6): δ 166.56, 151.57, 146.97, 145.72, 135.02, 134.99, 130.41, 129.51, 129.14, 121.55, 116.80, 115.56; IR: 3016, 1738, 1435, 1365, 1217, 752 cm^{-1}; HRMS (ESI+): m/z calcd for $C_{12}H_{11}N_3NaO^+$ $[M+Na]^+$: 236.07943; Found: 236.0795.

Supplementary Figure 28 Ester hydrolysis of nicotinates to nicotinic acids

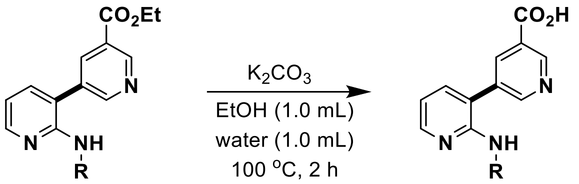

Experimental procedure: To a mixture of appropriate ethyl nicotinate derivative and K_2CO_3 (2.0 equiv.) was added a 1:1 mixture of EtOH/water and then the reaction mixture was heated at 100 °C for 2 h. The progress of the reaction was monitored by TLC and LC/MS. Upon complete consumption of starting material, the solvent was evaporated under the reduced pressure, the crude compound was diluted with water (10.0 mL) and washed with diethyl ether (2 × 10 mL). The desired product was precipitated out upon neutralizing the aqueous layer with saturated citric acid solution (pH 5–7). The precipitate was filtered, washed with water followed by hexane, and dried *in vacuo* to obtain the desired product.

Note: The sulfonyl-protected product **3a′** did not precipitate out upon neutralization. The crude compound was extracted using 10% MeOH in EtOAc (3 × 10 mL), and the combined organic layer was washed with brine (10 mL), dried over anhydrous Na_2SO_4(s) and solvent was evaporated *in vacuo* to obtain the desired product. For products **3a′** and **3d′**, the reaction was performed in 0.3 mmol scale and ~3.0 mL of solvent used. For products **6a′**, the reaction was performed in 0.255 mmol scale and ~2.6 mL of solvent used.

Compound 3a′: 2'-(Phenylsulfonamido)-[3,3'-bipyridine]-5-carboxylic acid

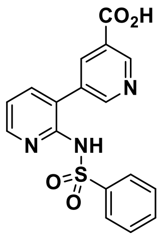

3a'

Off-white solid; Yield: 80% (85 mg), ^{1}H NMR (400 MHz, DMSO-d_6): δ 12.86 (brs, 1H), 9.07 (s, 1H), 8.85 (d, J = 1.6 Hz, 1H), 8.38 (t, J = 1.6 Hz, 1H), 8.07 (s, 1H), 7.97–7.85 (m, 3H), 7.63–7.50 (m, 3H), 7.04 (s, 1H); ^{13}C NMR (100 MHz, DMSO-d_6): δ 166.15, 153.00, 149.87, 149.26, 142.82, 141.32, 137.39, 136.38, 132.20, 132.00, 128.83 (3C), 126.16 (2C), 125.84, 113.00; IR: 3401, 1658, 1158, 1023, 996, 762 cm^{-1}; LRMS (ESI): m/z calcd for $C_{17}H_{14}N_3O_4S$ [M+H]$^+$: 356.07; Found: 356.05.

Compound 3d′: 2'-(Benzylamino)-[3,3'-bipyridine]-5-carboxylic acid

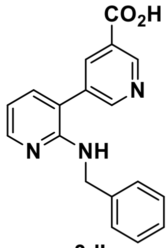

3d'

Yellow solid; Yield: 76% (70 mg); ^{1}H NMR (400 MHz, DMSO-d_6) δ 13.52 (brs, 1H), 9.08 (s, 1H), 8.83 (s, 1H), 8.29 (t, J = 2.0 Hz, 1H), 8.04 (dd, J = 4.8, 1.6 Hz, 1H), 7.39 (dd, J = 7.2, 1.6 Hz, 1H), 7.33–7.11 (m, 5H), 6.74–6.59 (m, 2H), 4.52 (d, J = 6.0 Hz, 2H); ^{13}C NMR (100 MHz, DMSO-d_6) δ 166.24, 155.38, 153.15, 148.98, 147.77, 141.08, 137.83, 137.17, 133.67, 128.00 (2C), 126.94 (2C), 126.79, 126.17, 117.22, 112.33, 43.90; IR: 3352, 1810, 1645, 1502, 1275, 748 cm^{-1}; LRMS (ESI): m/z calcd for $C_{18}H_{16}N_3O_2$ [M+H]$^+$: 306.12; Found: 306.10.

Compound 6a′: 2'-Amino-[3,3'-bipyridine]-5-carboxylic acid

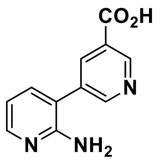

6a'

White solid; Yield: 91% (50 mg); ^{1}H NMR (500 MHz, DMSO-d_6): δ 9.04 (d, J = 2.0 Hz, 1H), 8.80 (d, J = 2.5 Hz, 1H), 8.28 (t, J = 2.0 Hz, 1H), 8.02 (dd, J = 5.0, 2.0 Hz, 1H), 7.43 (dd, J = 7.5, 2.0 Hz, 1H), 6.70 (dd, J = 7.5, 5.0 Hz, 1H), 5.90 (s, 2H); ^{13}C NMR (125 MHz, DMSO-d_6) δ 166.35, 156.84, 152.86, 148.82, 147.97, 138.24, 136.73, 133.92, 126.69, 116.23, 113.05.; LRMS (ESI): m/z calcd for $C_{11}H_{10}N_3O_2$ [M+H]$^+$: 216.08; Found: 216.10.

V. Late-stage Skeletal Transformation for the Synthesis of Bioactive Nicotinates and Anilinic Drugs

Following the general procedure described in **Supplementary Figure 24** with starting material **3a** (0.2 mmol) and alkynes (**2b–2i**). For the compound **3ah**, the reaction performed in 0.5 mmol scale with 5 mL of solvent. Compound **2b** is methyl propiolate (commercially available).

Compound 3ab: Methyl 2'-(phenylsulfonamido)-[3,3'-bipyridine]-5-carboxylate

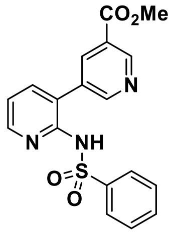

3ab

Yellow liquid; Yield: 85% (63 mg); ^{1}H NMR (500 MHz, $CDCl_3$): δ 9.15 (d, *J* = 2.0 Hz, 1H), 8.82 (d, *J* = 2.0 Hz, 1H), 8.42 (t, *J* = 2.0 Hz, 1H), 8.02–7.95 (m, 2H), 7.79 (s, 1H), 7.66 (dd, *J* = 7.5, 2.0 Hz, 1H), 7.57–7.45 (m, 3H), 6.77 (t, *J* = 7.0 Hz, 1H), 3.95 (s, 3H); ^{13}C NMR (125 MHz, $CDCl_3$) δ 165.51, 153.27, 151.74, 150.36, 142.89, 141.48, 137.82, 136.63, 132.19, 131.65, 128.96 (2C), 128.70, 126.27 (2C), 125.62, 112.46, 52.63; IR: 3210, 1724, 1623, 1539, 1258, 735 cm^{-1}; HRMS (ESI+): *m/z* calcd for $C_{18}H_{15}N_3NaO_4S^+$ $[M+Na]^+$: 392.06755; Found: 392.0675.

Compound 3ac: Benzyl 2'-(phenylsulfonamido)-[3,3'-bipyridine]-5-carboxylate

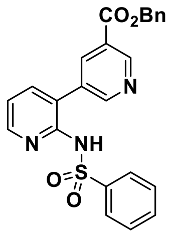

3ac

Off-white solid; Yield: 86% (77 mg); ^{1}H NMR (400 MHz, $CDCl_3$) δ 9.19 (d, *J* = 2.0 Hz, 1H), 8.82 (d, *J* = 2.4 Hz, 1H), 8.44 (t, *J* = 2.0 Hz, 1H), 8.01–7.91 (m, 2H), 7.76 (s, 1H), 7.63 (dd, *J* = 7.2, 1.6 Hz, 1H), 7.54–7.33 (m, 8H), 6.75 (t, *J* = 6.8 Hz, 1H), 5.40 (s, 2H); ^{13}C NMR (100 MHz, $CDCl_3$) δ 164.91, 153.46, 151.75, 150.49, 142.81, 141.48, 137.82, 136.94, 135.57, 132.19, 131.67, 128.95 (2C), 128.83 (2C), 128.62, 128.55, 128.40 (2C), 126.25 (2C), 125.67, 112.27, 67.32; IR: 3210, 1722, 1624, 1590, 1257, 741 cm^{-1}; HRMS (ESI+): *m/z* calcd for $C_{24}H_{19}N_3NaO_4S^+$ $[M+Na]^+$: 468.09885; Found: 468.0989.

Compound 3ad: Hexyl 2'-(phenylsulfonamido)-[3,3'-bipyridine]-5-carboxylate

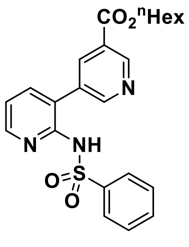

3ad

Brown solid; Yield: 80% (70 mg); ^{1}H NMR (400 MHz, $CDCl_3$): δ 12.81 (brs, 1H), 9.16 (d, *J* = 2.8 Hz, 1H), 8.82 (d, *J* = 2.4 Hz, 1H), 8.41 (s, 1H), 7.98 (d, *J* = 7.2 Hz, 2H), 7.79 (s, 1H), 7.66 (d, *J* = 7.2 Hz, 1H), 7.57–7.45 (m, 3H), 6.77 (t, *J* = 6.8 Hz, 1H), 4.36 (t, *J* = 6.8 Hz, 2H), 1.77 (p, *J* = 6.8 Hz, 2H), 1.49–1.39 (m, 2H), 1.38–1.30 (m, 4H), 0.98–0.85 (m, 3H); ^{13}C NMR (100 MHz, $CDCl_3$): δ 165.11, 153.29, 151.74, 150.36, 142.78, 141.47, 137.71, 136.66, 132.21, 131.64, 128.96 (2C), 128.70, 126.29 (2C), 126.04, 112.32, 65.85, 31.53, 28.72, 25.72, 22.63, 14.12; IR: 3211, 1720, 1623, 1589, 1255, 766 cm^{-1}; HRMS (ESI+): *m/z* calcd for $C_{23}H_{25}N_3NaO_4S^+$ $[M+Na]^+$: 462.14580; Found: 462.1460.

Compound 3ae: Tetradecyl 2'-(phenylsulfonamido)-[3,3'-bipyridine]-5-carboxylate

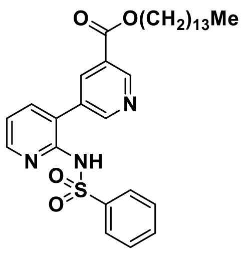

3ae

Brown solid; Yield: 75% (83 mg); ^{1}H NMR (400 MHz, $CDCl_3$): δ 9.29–8.99 (m, 1H), 8.83 (s, 1H), 8.41 (s, 1H), 8.03–7.93 (m, 2H), 7.84 (d, *J* = 6.4 Hz, 1H), 7.66 (dd, *J* = 7.2, 1.6 Hz, 1H), 7.56–7.42 (m, 3H), 6.76 (t, *J* = 6.4 Hz, 1H), 4.35 (t, *J* = 6.8 Hz, 2H), 1.77 (p, *J* = 6.8 Hz, 2H), 1.47–1.38 (m, 2H), 1.38–1.22 (m, 20H), 0.87 (t, *J* = 6.8 Hz, 3H); ^{13}C NMR (100 MHz, $CDCl_3$) δ 165.08, 153.19, 151.67, 150.14, 142.91, 141.54, 137.65, 136.48, 132.07, 131.79, 128.89 (2C), 128.62, 126.17 (2C), 125.94, 112.44, 65.77, 31.94, 29.71, 29.69, 29.67 (2C), 29.63, 29.55, 29.38, 29.31, 28.69, 25.98, 22.71, 14.15; IR: 2923, 1722, 1624, 1591, 1257, 967 cm^{-1}; HRMS (ESI+): *m/z* calcd for $C_{31}H_{41}N_3NaO_4S^+$ $[M+Na]^+$: 574.27100; Found: 574.2712.

Compound 3af: 2-(1,3-Dimethyl-2,6-dioxo-1,2,3,6-tetrahydro-7*H*-purin-7-yl)ethyl 2'-(phenylsulfonamido)-[3,3'-bipyridine]-5-carboxylate

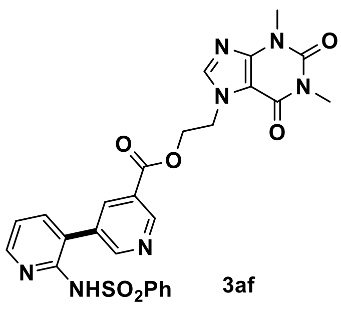

3af

Gummy yellow solid; Yield: 96% (108 mg); ^{1}H NMR (400 MHz, $CDCl_3$) δ 12.81 (brs, 1H), 9.03 (d, *J* = 2.0 Hz, 1H), 8.80 (d, *J* = 2.0 Hz, 1H), 8.46 (t, *J* = 2.0 Hz, 1H), 7.94 (d, *J* = 7.2 Hz, 2H), 7.83 (s, 1H), 7.74 (s, 1H), 7.68 (d, *J* = 7.6 Hz, 1H), 7.55–7.42 (m, 3H), 6.79 (t, *J* = 7.2 Hz, 1H), 4.79–4.67 (m, 4H), 3.56 (s, 3H), 3.39 (s, 3H); ^{13}C NMR (100 MHz, $CDCl_3$) δ 164.42, 155.29, 153.49, 151.64, 150.08, 149.17, 142.83, 142.03, 141.44, 137.86, 136.37, 132.18, 131.69, 128.91 (2C), 128.82, 126.13 (2C), 126.03, 124.75, 112.34, 106.76, 63.81, 46.05, 29.88, 28.03; IR: 3112, 1727, 1701, 1654,

1541, 1255, 730 cm^{-1}; HRMS (ESI+): *m/z* calcd for $C_{26}H_{23}N_7NaO_6S^+$ [M+Na]$^+$: 584.13227; Found: 584.1324.

Compound 3ag: (1*R*,5*S*)-3,3,5-Trimethylcyclohexyl 2'-(phenylsulfonamido)-[3,3'-bipyridine]-5-carboxylate

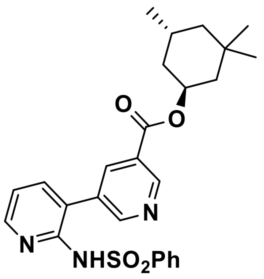

3ag

Pale yellow gummy liquid; Yield: 83% (80 mg); ^{1}H NMR (400 MHz, $CDCl_3$): δ 9.16 (d, *J* = 3.6 Hz, 1H), 8.83 (s, 1H), 8.41 (d, *J* = 2.4 Hz, 1H), 8.02–7.94 (m, 2H), 7.87–7.79 (m, 1H), 7.65 (dd, *J* = 7.2, 1.6 Hz, 1H), 7.57–7.43 (m, 3H), 6.78 (t, *J* = 6.0 Hz, 1H), 5.41 (t, *J* = 2.4 Hz, 1H), 1.98 (d, *J* = 12.4 Hz, 3H), 1.83 (dd, *J* = 15.2, 2.4 Hz, 1H), 1.51 (dd, *J* = 13.2, 2.8 Hz, 1H), 1.41 (dd, *J* = 15.2, 3.2 Hz, 1H), 1.22–1.08 (m, 4H), 0.98–0.90 (m, 6H); ^{13}C NMR (100 MHz, $CDCl_3$) δ 164.50, 153.22, 151.67, 150.13, 142.82, 141.48, 137.70, 136.47, 132.15, 131.70, 128.93 (2C), 128.36, 126.50 (2C), 126.23, 112.36, 72.89, 48.00, 41.58, 38.43, 33.92, 30.65, 27.68, 23.54, 22.52; IR: 2949, 1717, 1624, 1591, 1262, 1084, 965 cm^{-1}; HRMS (ESI+): *m/z* calcd for $C_{26}H_{29}N_3NaO_4S^+$ [M+Na]$^+$: 502.17710; Found: 502.1774.

Compound 3ah: (10*S*,13*R*,17*R*)-10,13-Dimethyl-3-oxo-2,3,6,7,8,9,10,11,12,13,14,15,16,17-tetradecahydro-1H-cyclopenta[a]phenanthren-17-yl 2'-(phenylsulfonamido)-[3,3'-bipyridine]-5-carboxylate

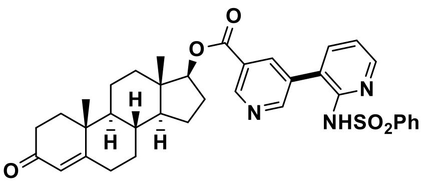

3ah

Yellow solid; Yield: 12% (40 mg); ^{1}H NMR (400 MHz, $CDCl_3$) δ 9.18 (d, *J* = 2.0 Hz, 1H), 8.82 (d, *J* = 2.4 Hz, 1H), 8.44 (t, *J* = 2.0 Hz, 1H), 8.03–7.86 (m, 2H), 7.80–7.59 (m, 2H), 7.60–7.41 (m, 3H), 6.79 (t, *J* = 6.8 Hz, 1H), 5.74 (s, 1H), 4.90 (t, *J* = 8.0 Hz, 1H), 2.50–2.36 (m, 2H), 2.34–2.26 (m, 1H), 2.09–1.99 (m, 1H), 1.92–1.83 (m, 1H), 1.80–1.56 (m, 6H), 1.50–1.38 (m, 2H), 1.34–1.09 (m, 7H), 1.09–0.93 (m, 4H), 0.92–0.83 (m, 1H); ^{13}C NMR (100 MHz, $CDCl_3$): δ 199.59, 170.95, 164.95, 153.27, 151.77, 150.45, 142.34, 141.21, 137.75, 136.83, 132.32, 131.60, 128.97 (2C), 128.83, 126.44 (2C), 126.25, 124.16, 113.78, 83.83, 53.86, 50.44, 43.11, 38.77, 36.85, 35.87, 35.57, 34.08, 32.87, 31.64, 27.78, 23.74, 20.70, 17.57, 12.48; IR: 3210, 1725, 1623, 1590, 1260, 1084, 744 cm^{-1}; HRMS (ESI+): *m/z* calcd for $C_{36}H_{39}N_3NaO_5S^+$ [M+Na]$^+$: 648.25026; Found: 648.2506.

Compound 3ai: (13*R*,17*R*)-3-Hydroxy-13-methyl-7,8,9,11,12,13,14,15,16,17-decahydro-6*H*-cyclopenta[a]phenanthren-17-yl 2'-(phenylsulfonamido)-[3,3'-bipyridine]-5-carboxylate

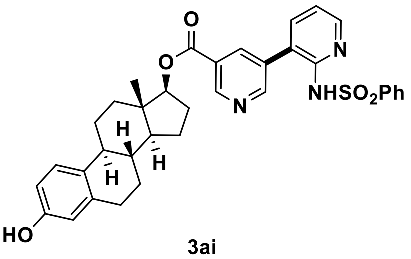

3ai

White solid; Yield: 66% (81 mg); ^{1}H NMR (400 MHz, $CDCl_3/CD_3OD$) δ 9.14 (d, *J* = 2.0 Hz, 1H), 8.80 (d, *J* = 2.0 Hz, 1H), 8.47 (t, *J* = 2.0 Hz, 1H), 7.97–7.90 (m, 2H), 7.86–7.74 (m, 1H), 7.71 (dd, *J* = 7.2, 2.0 Hz, 1H), 7.58–7.44 (m, 3H), 7.13 (d, *J* = 8.4 Hz, 1H), 6.90–6.79 (m, 1H), 6.64 (dd, *J* = 8.4, 2.8 Hz, 1H), 6.57 (d, *J* = 2.8 Hz, 1H), 5.05–4.92 (m, 1H), 2.93–2.74 (m, 2H), 2.42–2.27 (m, 2H), 2.27–2.18 (m, 1H), 1.98–1.79 (m, 3H), 1.76–1.66 (m, 1H), 1.56–1.44 (m, 4H), 1.44–1.27 (m, 4H), 0.96 (s, 3H); ^{13}C NMR (100 MHz, $CDCl_3/CD_3OD$) δ 164.86, 154.36, 152.77, 151.62, 149.62, 142.58, 141.53, 138.03, 137.83, 136.47, 132.12, 132.05, 131.33, 128.82 (2C), 128.67, 126.29 (2C), 126.06, 125.96, 115.20, 112.72, 112.64, 84.18, 49.75, 43.74, 43.38, 38.60, 36.93, 29.54, 27.64, 27.20, 26.16, 23.30, 12.31; IR: 2922, 1730, 1624, 1589, 1257, 749 cm^{-1}; HRMS (ESI+): *m/z* calcd for $C_{35}H_{35}N_3NaO_5S^+$ $[M+Na]^+$: 632.21896; Found: 632.2188.

Supplementary Figure 29. Propionation of **3ai** to **3ai'** (estropronicate embedded with arylamine scaffold)

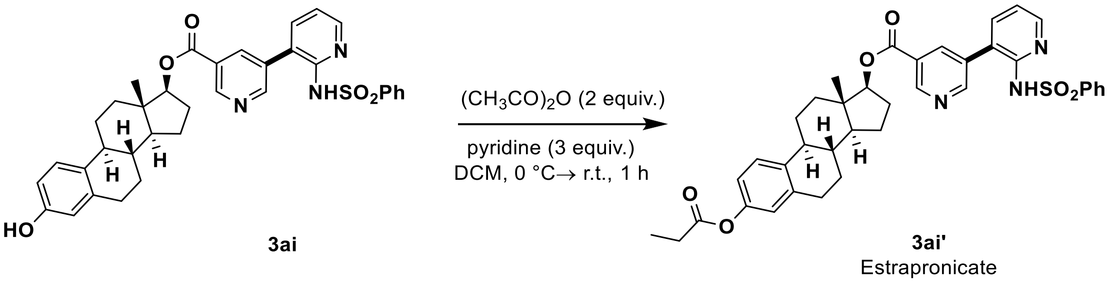

3ai

3ai'
Estrapronicate

Experimental procedure: To a stirred solution of [(13*R*,17*R*)-3-hydroxy-13-methyl-6,7,8,9,11,12,14,15,16,17-decahydrocyclopenta[a]phenanthren-17-yl] 5-[2-(benzenesulfonamido)-3-pyridyl]pyridine-3-carboxylate (58 mg, 0.095 mmol) in DCM (4 mL), were added pyridine (22.6 mg, 0.29 mmol, 23 μL) and propanoyl propanoate (24.8 mg, 0.19 mol, 24.2 μL) at 0 °C, then the reaction mixture was warmed to r.t. and then stirred for 1 h. After that, the reaction mixture was diluted with DCM, washed with saturated $NaHCO_3$ solution (2 × 10 mL) and brine (10 mL), dried over anhydrous

$Na2SO4(s)$, evaporated to give crude product which was purified by silica-gel flash column chromatography using EA and hexane (1:1) as an eluent to furnish, titled compound **3ai′** (23 mg, 36.32% yield) as off-white solid.

Compound 3ai′: (13*R*,17*R*)-13-Methyl-3-(propionyloxy)-7,8,9,11,12,13,14,15,16,17-decahydro-6*H*-cyclopenta[a]phenanthren-17-yl 2'-(phenylsulfonamido)-[3,3'-bipyridine]-5-carboxylate

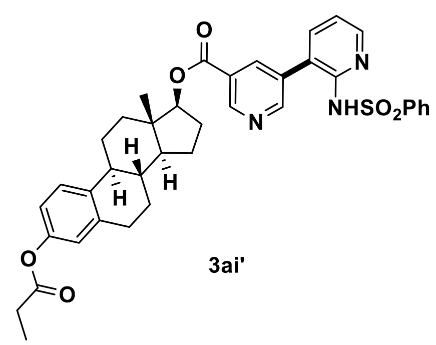

3ai'

^{1}H NMR (500 MHz, $CDCl_3$) δ 9.19 (d, *J* = 2.0 Hz, 1H), 8.83 (d, *J* = 2.0 Hz, 1H), 8.42 (t, *J* = 2.0 Hz, 1H), 8.00–7.94 (m, 2H), 7.74 (s, 1H), 7.65 (d, *J* = 7.0 Hz, 1H), 7.56–7.46 (m, 3H), 7.28 (dd, *J* = 8.5, 1.0 Hz, 1H), 6.84 (dd, *J* = 8.5, 2.5 Hz, 1H), 6.82–6.74 (m, 2H), 5.02–4.95 (m, 1H), 2.95–2.81 (m, 2H), 2.57 (q, *J* = 7.5 Hz, 2H), 2.41–2.25 (m, 3H), 2.00–1.89 (m, 2H), 1.87–1.79 (m, 1H), 1.77–1.61(m, 2H), 1.58–1.45 (m, 3H), 1.43–1.34 (m, 2H), 1.26 (t, *J* = 7.5 Hz, 3H), 0.96 (s, 3H); ^{13}C NMR (125 MHz, $CDCl_3$): δ 173.39, 165.00, 153.30, 150.43, 148.71, 142.77, 141.38, 138.20, 137.71, 136.44, 132.27, 131.65, 128.97 (2C), 128.59, 126.51 (2C), 126.29, 126.18, 121.65, 118.75, 115.47, 112.66, 84.01, 50.01, 44.11, 43.49, 38.37, 37.08, 29.61, 27.89, 27.85, 27.18, 26.17, 23.51, 12.52, 9.26; IR: 3211, 1719, 1756, 1624, 1592, 1258, 766 cm^{-1}; HRMS (ESI+): *m/z* calcd for $C_{38}H_{39}N_3NaO_6S^+$ $[M+Na]^+$: 688.24518; Found: 688.2453.

Compound 3aj: Ethyl 2'-((4-acetamidophenyl)sulfonamido)-[3,3'-bipyridine]-5-carboxylate

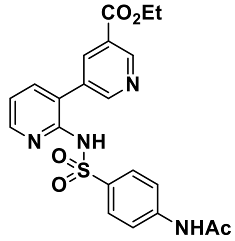

3aj

Using the synthetic procedure described in **Supplementary Figure 24** with starting material **1aj** and ethyl propiolate (**2a**). White solid; Yield: 65% (57 mg); ^{1}H NMR (500 MHz, CD_2Cl_2/CD_3OD) δ 9.12 (s, 1H), 8.78 (d, *J* = 2.5 Hz, 1H), 8.47 (s, 1H), 7.84 (d, *J* = 8.5 Hz, 2H), 7.81–7.59 (m, 4H), 6.84 (s, 1H), 4.42 (q, *J* = 7.0 Hz, 2H), 4.12 (s, 2H), 2.14 (s, 3H), 1.40 (t, *J* = 7.0 Hz, 3H); ^{13}C NMR (125 MHz, CD_2Cl_2/CD_3OD): δ171.02, 165.62, 153.37, 150.12, 144.48, 142.96, 138.75, 135.95, 132.95, 127.59 (2C), 126.87, 119.79 (2C), 112.31, 62.45, 24.20, 14.47 (3 carbons were not observed); IR: 3213, 1715, 1623, 1589, 1255, 767 cm^{-1}; HRMS (ESI+): *m/z* calcd for $C_{21}H_{20}N_4NaO_5S^+$ $[M+Na]^+$: 463.10466; Found: 463.1048.

Compound 3ak: Ethyl 2'-((4-acetamidophenyl)sulfonamido)-5'-nitro-[3,3'-bipyridine]-5-carboxylate

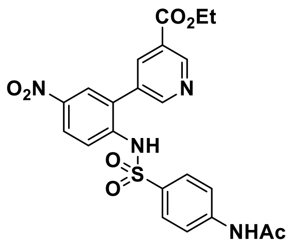

3ak

Using the general procedure described in **Supplementary Figure 25** with starting material **1ak** and ethyl propiolate (**2a**). Yellow solid; Yield: 67% (65 mg); ^{1}H NMR (400 MHz, $CDCl_3$): δ 8.99 (s, 1H), 8.51 (s, 1H), 8.39 (d, J = 10.0 Hz, 1H), 8.19 (dd, J = 9.2, 2.8 Hz, 1H), 8.13 (t, J = 2.0 Hz, 1H), 8.02 (d, J = 2.8 Hz, 1H), 7.76 (d, J = 8.8 Hz, 1H), 7.70–7.57 (m, 4H), 4.38 (q, J = 7.2 Hz, 2H), 2.16 (s, 3H), 1.40 (t, J = 7.2 Hz, 3H); ^{13}C NMR (100 MHz, $CDCl_3$): δ 169.43, 164.35, 152.87, 150.61, 144.06, 143.34, 140.60, 138.05, 132.89, 131.93, 129.31, 128.52 (2C), 126.93, 126.57 (2C), 125.32, 120.94, 119.66, 62.21, 24.71, 14.31; IR: 3108, 1717, 1698, 1623, 1585, 1370, 1255, 766 cm^{-1}; HRMS (ESI+): *m/z* calcd for $C_{22}H_{20}N_4NaO_7S^+$ $[M+Na]^+$: 507.09504; Found: 507.0978.

Supplementary Figure 30. Deacylation of **3aj** to **3aj'** (Sulfapyridine embedded with nicotinate scaffold)

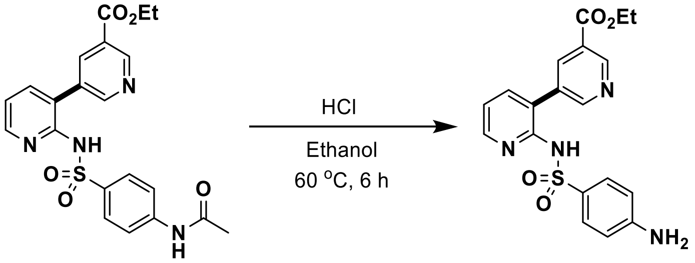

3aj

3aj'
Nico-Sulfa pyridine

Experimental procedure: To a stirred solution of ethyl 5-[2-[(4-acetamidophenyl)sulfonylamino]-3pyridyl]pyridine-3-carboxylate (**3aj**, 50 mg, 113.51 μmol) in ethanol (1.30 mL) was added HCl (11 M, 0.9 mL) (30–35% in water), and the reaction mixture was heated at 60 °C for 1 h. The progress of the reaction was monitored by TLC and LC/MS. Upon completion of the reaction, the reaction mixture was quenched with addition of solid K_2CO_3 in a portion wise and stirred for additional 1 h at r.t. The reaction mixture was then filtered through a short pad of celite, washed with the 20% MeOH in DCM (30 mL), and the filtrate was collected and condensed under the reduced pressure. The obtained crude product was purified by Biotage Isolera column chromatography using 4% methanol in DCM as an eluent to furnish the desired product (**3ak'**) as an off-white solid (39.6 mg, 88%) as off-white solid.

Compound 3aj′: Ethyl 2'-((4-aminophenyl)sulfonamido)-[3,3'-bipyridine]-5-carboxylate

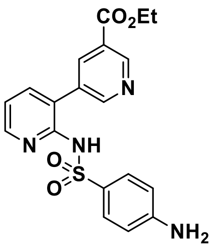

3aj'

^{1}H NMR (500 MHz, CD_2Cl_2): δ 9.04 (d, J = 2.0 Hz, 1H), 8.71 (d, J = 2.0 Hz, 1H), 8.31 (s, 1H), 7.73 (s, 1H), 7.64–7.55 (m, 2H), 7.55–7.48 (m, 1H), 6.71 (s, 1H), 6.59–6.52 (m, 2H), 4.31 (q, J = 7.0 Hz, 2H), 4.17 (s, 2H), 1.31 (t, J = 7.0, Hz, 3H); ^{13}C NMR (125 MHz, CD_2Cl_2): δ 165.46, 153.65, 151.25, 150.62, 141.21, 137.93 136.54, 132.37, 128.98 (2C), 126.63, 126.56, 126.53, 114.13 (2C), 62.12, 14.59; IR: 3364, 1717, 1624, 1586, 1256, 1111 cm^{-1}; HRMS (ESI+): m/z calcd for $C_{19}H_{18}N_4NaO_4S^+$ $[M+Na]^+$: 421.09410; Found: 421.0944.

Supplementary Figure 31. Synthesis of *Nico*-paracetamol/phenacetin

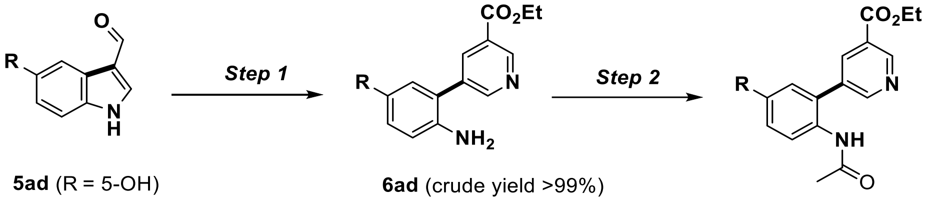

5ad (R = 5-OH)
5ae (R = 5-OEt)

6ad (crude yield >99%)
6ae (crude yield >99%)

6ad' (Isolatd; 60%)
Nico-Paracetamol
6ae' (Isolated; 59%)
Nico-Phenacetin

General procedure:

Step 1: Reactions were performed under the standard reaction conditions **(Supplementary Figure 26)**. After completion of the reaction, the reaction mixture was passed through a short pad of celite, washed with 5% MeOH in DCM, evaporated the solvent. Formation of desired product was confirmed by ^{1}H NMR and LCMS. The crude compound was taken into next step without further purification.

Step 2: To a stirred solution of crude compound obtained from Step 1 in 6.0 mL of 1:1 mixture of THF and H_2O was slowly added to a mixture of Ac_2O (0.4 mmol, 37.8 μL) at room temperature and heated at 120 °C for 20 min. The reaction was monitored by TLC and LC/MS. After completion of the reaction, it was quenched with saturated $NaHCO_3$ solution (10 mL) and extracted with EtOAc (2 × 10 mL). The combined organic layer was given a brine wash (10 mL), dried over anhydrous Na_2SO_4(s), filtered, concentrated under reduced pressure and purified by silica-gel flash column chromatography using EtOAc/Hexane mixture as an eluent to furnish the desired product.

Compound 6ad′: Ethyl 5-(2-acetamido-5-hydroxyphenyl)nicotinate

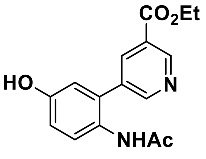

6ad'

Brown solid; Yield: 60% (36 mg); ^{1}H NMR (400 MHz, DMSO-d_6): δ 9.68 (s, 1H), 9.30 (s, 1H), 9.04 (d, J = 2.0 Hz, 1H), 8.75 (d, J = 2.0 Hz, 1H), 8.19 (t, J = 2.0 Hz, 1H), 7.16 (d, J = 8.4 Hz, 1H), 6.89–6.77 (m, 2H), 4.38 (q, J = 7.2 Hz, 2H), 1.79 (s, 3H), 1.35 (t, J = 7.2 Hz, 3H); ^{13}C NMR (100 MHz, DMSO-d_6): δ 168.80, 164.66, 155.88, 152.71, 148.35, 136.14, 135.04, 134.47, 129.44, 126.46, 125.36, 116.14, 115.75, 61.24, 22.55,

14.07; IR: 3233, 1722, 1642, 1529, 1254, 765 cm^{-1}; HRMS (ESI+): *m/z* calcd for $C_{16}H_{16}N_2NaO_4^+$ $[M+Na]^+$: 323.10023; Found: 323.1005.

Compound 6ae′: Ethyl 5-(2-acetamido-5-ethoxyphenyl)nicotinate

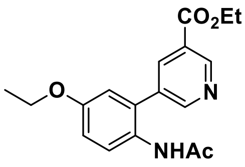

6ae'

Off-white solid; Yield: 59% (39 mg); ^1H NMR (400 MHz, $CDCl_3$): δ 9.18 (s, 1H), 8.75 (s, 1H), 8.30 (d, *J* = 2.4 Hz, 1H), 7.68 (d, *J* = 8.8 Hz, 1H), 7.11–7.01 (m, 1H), 6.96 (dd, *J* = 8.8, 2.8 Hz, 1H), 6.82 (d, *J* = 2.8 Hz, 1H), 4.42 (q, *J* = 7.2 Hz, 2H), 4.05 (q, *J* = 6.8 Hz, 2H), 2.01 (s, 3H), 1.45–1.38 (m, 6H); ^{13}C NMR (100 MHz, $CDCl_3$) δ 169.12, 165.09, 157.16, 153.20, 149.91, 137.40, 134.57, 132.53, 127.37, 127.30, 126.40, 116.33, 115.26, 64.02, 61.81, 23.82, 14.89, 14.39; IR: 3251, 1722, 1661, 1524, 1275, 1045 cm^{-1}; HRMS (ESI+): *m/z* calcd for $C_{18}H_{20}N_2NaO_4^+$ $[M+Na]^+$: 351.13153; Found: 351.1316.

Supplementary Figure 32: Preparation of bioactive *meta*-(hetero)aryl nicotinate analogues

These compounds were synthesized using the general procedure described in Supplementary Figure 26.

Compound 6af: Ethyl 5-(2-amino-5-(ethoxycarbonyl)phenyl)nicotinate

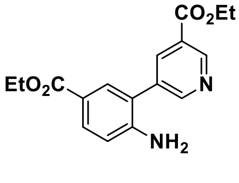

6af

Using the general procedure described in **Supplementary Figure 26** with starting material (**5af**) and ethyl propiolate (**2a**). Brown solid; Yield: 89% (56 mg); ^1H NMR (400 MHz, $CDCl_3$): δ 9.22 (d, *J* = 2.0 Hz, 1H), 8.87 (d, *J* = 2.4 Hz, 1H), 8.40 (t, *J* = 2.4 Hz, 1H), 7.91 (dd, *J* = 8.4, 2.0 Hz, 1H), 7.82 (d, *J* = 2.0 Hz, 1H), 6.78 (d, *J* = 8.4 Hz, 1H), 4.44 (q, *J* = 7.2 Hz, 2H), 4.34 (q, *J* = 7.2 Hz, 2H), 4.18 (brs, 2H), 1.43 (t, *J* = 7.2 Hz, 3H), 1.37 (t, *J* = 7.2 Hz, 3H); ^{13}C NMR (100 MHz, $CDCl_3$): δ 166.47, 165.16, 153.69, 149.98, 148.10, 137.61, 134.29, 132.72, 131.74, 126.64, 121.67, 120.86, 115.08, 61.80, 60.71, 14.54, 14.40; IR: 3365, 1707, 1608, 1572, 1306, 1256, 769 cm^{-1}; HRMS (ESI+): *m/z* calcd for $C_{17}H_{19}N_2O_4^+$ $[M+H]^+$: 315.13393; Found: 315.1342.

Compound 6ag: Ethyl 5-(2-amino-5-(butoxycarbonyl)phenyl)nicotinate

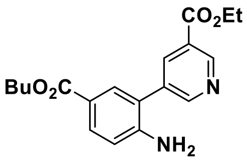

6ag

Using the general procedure described in **Supplementary Figure 26** with starting material (**5ag**) and ethyl propiolate (**2a**). Brown solid; Yield: 73% (50 mg); ^{1}H NMR (400 MHz, $CDCl_3$): δ 9.21 (d, *J* = 1.6 Hz, 1H), 8.87 (d, *J* = 2.0 Hz, 1H), 8.40 (t, *J* = 2.0 Hz, 1H), 7.90 (dd, *J* = 8.4, 2.0 Hz, 1H), 7.81 (d, *J* = 2.0 Hz, 1H), 6.79 (d, *J* = 8.4 Hz, 1H), 4.44 (q, *J* = 7.2 Hz, 2H), 4.29 (t, *J* = 6.8 Hz, 2H), 4.20 (brs, 2H), 1.73 (dq, *J* = 8.0, 6.8 Hz, 2H), 1.52–1.38 (m, 5H), 0.97 (t, *J* = 7.2 Hz, 3H); ^{13}C NMR (100 MHz, $CDCl_3$): δ 166.53, 165.13, 153.66, 149.94, 148.13, 137.60, 134.29, 132.68, 131.71, 126.62, 121.65, 120.82, 115.06, 64.60, 61.78, 30.96, 19.38, 14.37, 13.88; IR: 3365, 1707, 1607, 1573, 1305, 1254, 769 cm^{-1}; HRMS (ESI+): *m/z* calcd for $C_{19}H_{23}N_2O_4^+$ $[M+H]^+$: 343.16523; Found: 343.1654.

Compound 6ah: Ethyl 5-(2-amino-5-((2-(diethylamino)ethoxy)carbonyl)phenyl)nicotinate

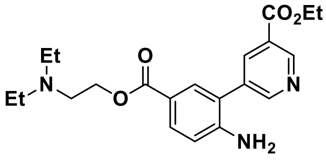

6ah

Following the general procedure described in **Supplementary Figure 26** with starting material (**5ah**) and ethyl propiolate (**2a**). Off-white solid; Yield: 23% (18 mg); ^{1}H NMR (400 MHz, $CDCl_3$): δ 9.22 (d, *J* = 2.0 Hz, 1H), 8.87 (d, *J* = 2.0 Hz, 1H), 8.39 (d, *J* = 2.0 Hz, 1H), 7.89 (dd, *J* = 8.4, 2.0 Hz, 1H), 7.81 (d, *J* = 2.0 Hz, 1H), 6.79 (d, *J* = 8.8 Hz, 1H), 4.43 (t, *J* = 6.4 Hz, 4H), 4.22 (s, 2H), 2.94 (t, *J* = 6.4 Hz, 2H), 2.73 (q, *J* = 7.2 Hz, 4H), 1.43 (t, *J* = 7.2 Hz, 3H), 1.12 (t, *J* = 7.2 Hz, 6H); ^{13}C NMR (100 MHz, $CDCl_3$): δ 166.30, 165.12, 153.61, 150.00, 148.34, 137.56, 134.19, 132.86, 131.79, 126.66, 121.67, 120.29, 115.12, 62.35, 61.81, 50.98, 47.87 (2C), 14.39, 11.60 (2C); IR: 3212, 1705, 1629, 1570, 1251, 768 cm^{-1}; HRMS (ESI+): *m/z* calcd for $C_{21}H_{28}N_3O_4^+$ $[M+H]^+$: 386.20743; Found: 386.2075.

Compound 6ai: 2-(1,3-Dimethyl-2,6-dioxo-1,2,3,6-tetrahydro-7H-purin-7-yl)ethyl 5-(2-amino-5-(ethoxycarbonyl)phenyl)nicotinate

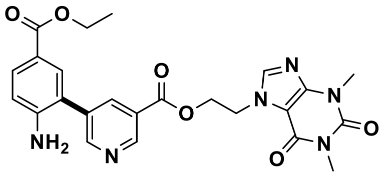

6ai

Following the general procedure described in **Supplementary Figure 26** with starting material **5af** and propiolate (**2f**). Pale yellow solid; Yield: 61% (60 mg); ^{1}H NMR (500 MHz, $CDCl_3$) δ 9.09 (s, 1H), 8.88 (d, *J* = 2.5 Hz, 1H), 8.32 (s, 1H), 7.90 (d, *J* = 8.5 Hz, 1H), 7.78 (d, *J* = 2.0 Hz, 1H), 7.64 (s, 1H), 6.80 (d, *J* = 8.5 Hz, 1H), 4.81–

4.71 (m, 4H), 4.33 (q, J = 7.0 Hz, 2H), 4.28 (s, 2H), 3.57 (s, 3H), 3.37 (s, 3H), 1.37 (t, J = 7.0 Hz, 3H); ^{13}C NMR (100 MHz, $CDCl_3$) δ 166.32, 164.53, 155.28, 154.19, 151.60, 149.65, 149.16, 148.15, 141.57, 137.49, 134.49, 132.58, 131.73, 125.42, 121.17, 120.72, 115.14, 106.89, 63.89, 60.64, 45.98, 29.86, 28.01, 14.46; IR: 3225, 1726, 1701, 1655, 1255, 1026, 762 cm^{-1}; HRMS (ESI+): *m/z* calcd for $C_{24}H_{24}N_6NaO_6^+$ $[M+Na]^+$: 515.16495; Found: 515.1658.

Compound 6aj: (1*R*,5*S*)-3,3,5-Trimethylcyclohexyl 5-(2-amino-5-(ethoxycarbonyl)phenyl)nicotinate

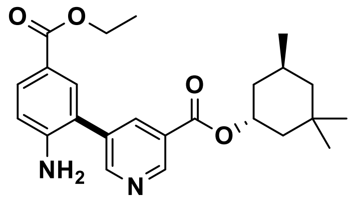

6aj

Following the general procedure described in **Supplementary Figure 26** with starting material **5af** and propiolate (**2g**). Light yellow solid; Yield: 74% (61 mg); ^{1}H NMR (500 MHz, $CDCl_3$): δ 9.22 (d, J = 2.0 Hz, 1H), 8.87 (d, J = 2.5 Hz, 1H), 8.41 (t, J = 2.0 Hz, 1H), 7.91 (dd, J = 8.5, 2.0 Hz, 1H), 7.82 (d, J = 2.0 Hz, 1H), 6.79 (d, J = 8.5 Hz, 1H), 5.42 (t, J = 3.0 Hz, 1H), 4.34 (q, J = 7.5 Hz, 2H), 4.20 (s, 2H), 2.06–1.91 (m, 3H), 1.84 (dd, J = 15.0, 2.5 Hz, 1H), 1.51 (dd, J = 13.0, 2.5 Hz, 1H), 1.46–1.34 (m, 4H), 1.23–1.10 (m, 4H), 0.98–0.91 (m, 6H); ^{13}C NMR (125 MHz, $CDCl_3$): δ 166.41, 164.63, 153.60, 149.89, 148.14, 137.51, 134.36, 132.70, 131.73, 127.12, 121.72, 120.98, 115.13, 73.03, 60.64, 48.11, 41.69, 38.52, 33.94, 30.71, 27.70, 23.65, 22.53, 14.52; IR: 3365, 1707, 1608, 1571, 1304, 1258, 1107, 770 cm^{-1}; HRMS (ESI+): *m/z* calcd for $C_{24}H_{31}N_2O_4^+$ $[M+H]^+$: 411.22783; Found: 411.2276.

Compound 6f′: Ethyl 5-(2-aminophenyl)nicotinate-4-*d*

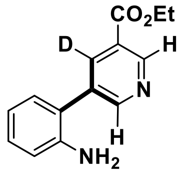

6f'

Following the general procedure described in **Supplementary Figure 26** with starting material **5f′** and ethyl propiolate (**2a**). Gummy yellow liquid; Yield: 77% (37 mg); ^{1}H NMR (400 MHz, $CDCl_3$): δ 9.19 (s, 1H), 8.88 (s, 1H), 7.21 (t, J = 7.6 Hz, 1H), 7.11 (d, J = 7.6 Hz, 1H), 6.86 (t, J = 7.6 Hz, 1H), 6.80 (d, J = 8.4 Hz, 1H), 4.43 (q, J = 7.2 Hz, 2H), 3.74 (s, 2H), 1.42 (t, J = 7.2 Hz, 3H); ^{13}C NMR (100 MHz, $CDCl_3$) δ 165.29, 153.74, 149.51, 143.87, 137.16 (t, J_{DC} = 25.5 Hz, 1C), 135.15, 130.69, 129.88, 126.36, 122.69, 119.17, 116.15, 61.65, 14.38; IR: 3401, 1658, 1158, 1023, 996, 762 cm^{-1}; HRMS (ESI+): *m/z* calcd for $C_{14}H_{13}DN_2O_2^+$ $[M+H]^+$: 244.11908; Found: 244.1193.

Compound 6f″: Ethyl 5-(2-aminophenyl)nicotinate-6-*d*

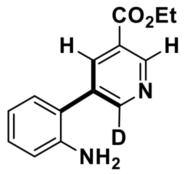

6f"

Following the general procedure described in **Supplementary Figure 26** with starting material **5f″** and ethyl propiolate **(2a)**. Yellow gum; Yield: 86% (42 mg); ^{1}H NMR (500 MHz, $CDCl_3$): δ 9.19 (d, *J* = 2.0 Hz, 1H), 8.42 (d, *J* = 2.0 Hz, 1H), 7.22 (ddd, *J* = 9.0, 7.0, 1.5 Hz, 1H), 7.11 (dd, *J* = 7.5, 1.5 Hz, 1H), 6.87 (td, *J* = 7.5, 1.5 Hz, 1H), 6.80 (dd, *J* = 8.0, 1.0 Hz, 1H), 4.43 (q, *J* = 7.0 Hz, 2H), 3.74 (s, 2H), 1.42 (t, *J* = 7.0 Hz, 3H); ^{13}C NMR (125 MHz, $CDCl_3$): δ 165.30, 153.35(t, J_{DC} = 26.3 Hz, 1C), 149.50, 143.86, 137.50, 135.14, 130.69, 129.89, 126.47, 122.71, 119.18, 116.17, 61.66, 14.38; IR: 3216, 1717, 1623, 1304, 1238, 1119, 747 cm^{-1}; HRMS (ESI+): *m/z* calcd for $C_{14}H_{13}DN_2O_2^+$ $[M+H]^+$: 244.11908; Found: 244.1194.

Compound 6f‴: Ethyl 5-(2-aminophenyl)nicotinate-2-*d*

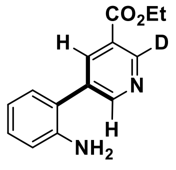

6f'''

Following the general procedure described in **Supplementary Figure 26** with starting material indole 3-carboxaldehyde **5f**, ethyl propiolate-*d* (**2a′**)[12] and EtOD as a solvent (2 mL). Yellow gum; Yield: 66% (32 mg); ^{1}H NMR (500 MHz, $CDCl_3$): δ 8.88 (d, *J* = 2.0 Hz, 1H), 8.42 (d, *J* = 2.5 Hz, 1H), 7.22 (td, *J* = 7.5, 1.5 Hz, 1H), 7.11 (dd, *J* = 7.5, 1.5 Hz, 1H), 6.89–6.85 (m, 1H), 6.84–6.77 (m, 1H), 4.43 (q, *J* = 7.0 Hz, 2H), 1.42 (t, *J* = 7.0 Hz, 3H); ^{13}C NMR (125 MHz, $CDCl_3$): δ 165.25, 153.60, 149.05(t, J_{DC}= 26.5 Hz, 1C), 143.84, 137.60, 135.33, 130.69, 129.90, 126.39, 122.68, 119.19, 116.17, 61.68, 14.38; IR: 3215, 1715, 1621, 1306, 1218, 1105, 747 cm^{-1}; LRMS (ESI): *m/z* calcd for $C_{14}H_{14}DN_2O_2$ $[M+H]^+$: 244.12; Found: 244.15.

Compound 10a′: Ethyl 5-([1,3]dioxolo[4,5-*J*]phenanthridin-4-yl)nicotinate

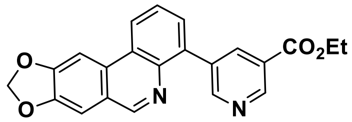

10a'

Following the general procedure described in **Supplementary Figure 26** with starting material **9a** and ethyl propiolate (**2a**). Off-white solid; Yield: 72% (53.5 mg); ^{1}H NMR (500 MHz, DMSO-d_6): δ 9.17–9.04 (m, 3H), 8.79 (d, *J* = 8.5 Hz, 1H), 8.55–8.50 (m, 1H), 8.37 (s, 1H), 7.86–7.81 (m, 1H), 7.77 (t, *J* = 7.5 Hz, 1H), 7.63 (s, 1H), 6.30 (s, 2H), 4.40 (q, *J* = 7.0 Hz, 2H), 1.36 (t, *J* = 7.0 Hz, 3H); ^{13}C NMR (125 MHz, DMSO-d_6): δ 164.87, 154.59, 151.84, 151.72, 148.37, 148.23, 140.76, 138.23, 135.72, 135.36, 129.71, 129.17, 126.57, 125.04, 124.49, 123.97, 122.80, 105.21, 102.33, 100.56, 61.23, 14.10; IR: 2922, 1717, 1470, 1251, 1036, 764 cm^{-1}; HRMS (ESI+): *m/z* calcd for $C_{22}H_{17}N_2O_4^+$ $[M+H]^+$: 373.11828; Found: 373.1184.

Compound 13a: Ethyl 5-(2-amino-6-(2-hydroxy-3-(isopropylamino)propoxy)phenyl)nicotinate

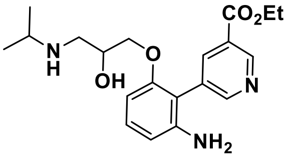

13a

Following the general procedure described in **Supplementary Figure 26** with starting material **12a** and ethyl propiolate (**2a**). Yellow gum; Yield: 34% (25 mg); ^{1}H NMR (500 MHz, $CDCl_3$): δ 9.08 (d, *J* = 2.0 Hz, 1H), 8.70 (d, *J* = 2.0 Hz, 1H), 8.30 (t, *J* = 2.0 Hz, 1H), 7.10 (t, *J* = 8.0 Hz, 1H), 6.44 (dd, *J* = 8.5, 1.0 Hz, 1H), 6.35 (dd, *J* = 8.5, 1.0 Hz, 1H), 4.39 (q, *J* = 7.0 Hz, 2H), 4.34–4.27 (m, 1H), 4.00–3.96 (m, 1H), 3.88 (dd, *J* = 10.0, 6.0 Hz, 1H), 3.24 (sept, *J* = 6.5 Hz, 1H), 2.83 (dd, *J* = 13.0, 2.5 Hz, 1H), 2.72 (dd, *J* = 12.5, 10.0 Hz, 1H), 2.05–2.00 (m, 1H), 1.39 (t, *J* = 7.0 Hz, 3H), 1.28 (d, *J* = 6.5 Hz, 3H), 1.25 (d, *J* = 6.5 Hz, 4H); ^{13}C NMR (125 MHz, $CDCl_3$): δ 165.26, 156.25, 155.42, 149.13, 145.36, 139.52, 131.13, 130.40, 126.64, 111.39, 109.54, 102.25, 70.02, 65.46, 61.76, 51.29, 47.91, 19.03, 18.80, 14.39; IR: 3336, 11717, 1625, 1583, 1306, 1242, 1107, 729 cm^{-1}; HRMS (ESI+): *m/z* calcd for $C_{20}H_{28}N_3O_4^+$ $[M+H]^+$: 374.20743; Found: 374.2077.

Compound 16a: Ethyl 5'-amino-2'-benzamido-[3,4'-bipyridine]-5-carboxylate

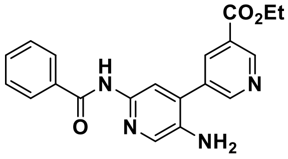

16a

Following the general procedure described in **Supplementary Figure 26** with starting material **15a** and ethyl propiolate (**2a**). Pale yellow solid; Yield: 67% (48 mg); ^{1}H NMR (500 MHz, DMSO-d_6): δ 10.52 (s, 1H), 9.13 (d, *J* = 2.0 Hz, 1H), 8.93 (d, *J* = 2.5 Hz, 1H), 8.37 (t, *J* = 2.0 Hz, 1H), 8.03–8.00 (m, 2H), 7.99 (d, *J* = 0.5 Hz, 1H), 7.91 (s, 1H), 7.59–7.55 (m, 1H), 7.52–7.47 (m, 2H), 5.21 (s, 2H), 4.40 (q, *J* = 7.0 Hz, 2H), 1.36 (t, *J* = 7.0 Hz, 3H); ^{13}C NMR (125 MHz, DMSO-d_6): δ 165.01, 164.57, 152.82, 149.14, 142.79, 139.15, 136.34, 135.51, 134.32, 133.38, 131.53, 129.05, 128.27 (2C), 127.70 (2C), 125.85, 115.80, 61.32, 40.01, 14.08; IR: 3240, 1727, 1663, 1549, 1392, 1271, 694 cm^{-1}; HRMS (ESI+): *m/z* calcd for $C_{20}H_{19}N_4O_3^+$ $[M+H]^+$: 363.14517; Found: 363.1451.

VI. ^{1}H, ^{13}C, and ^{19}F NMR Spectra

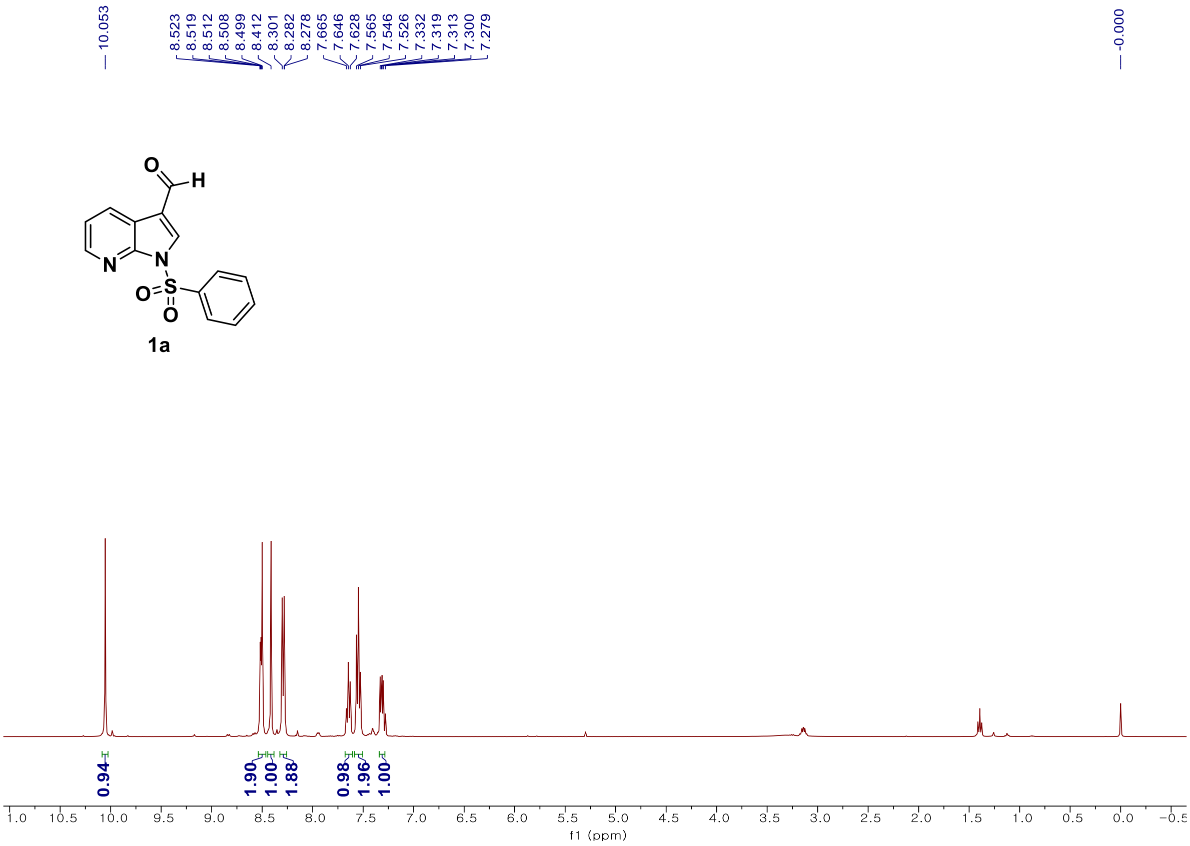

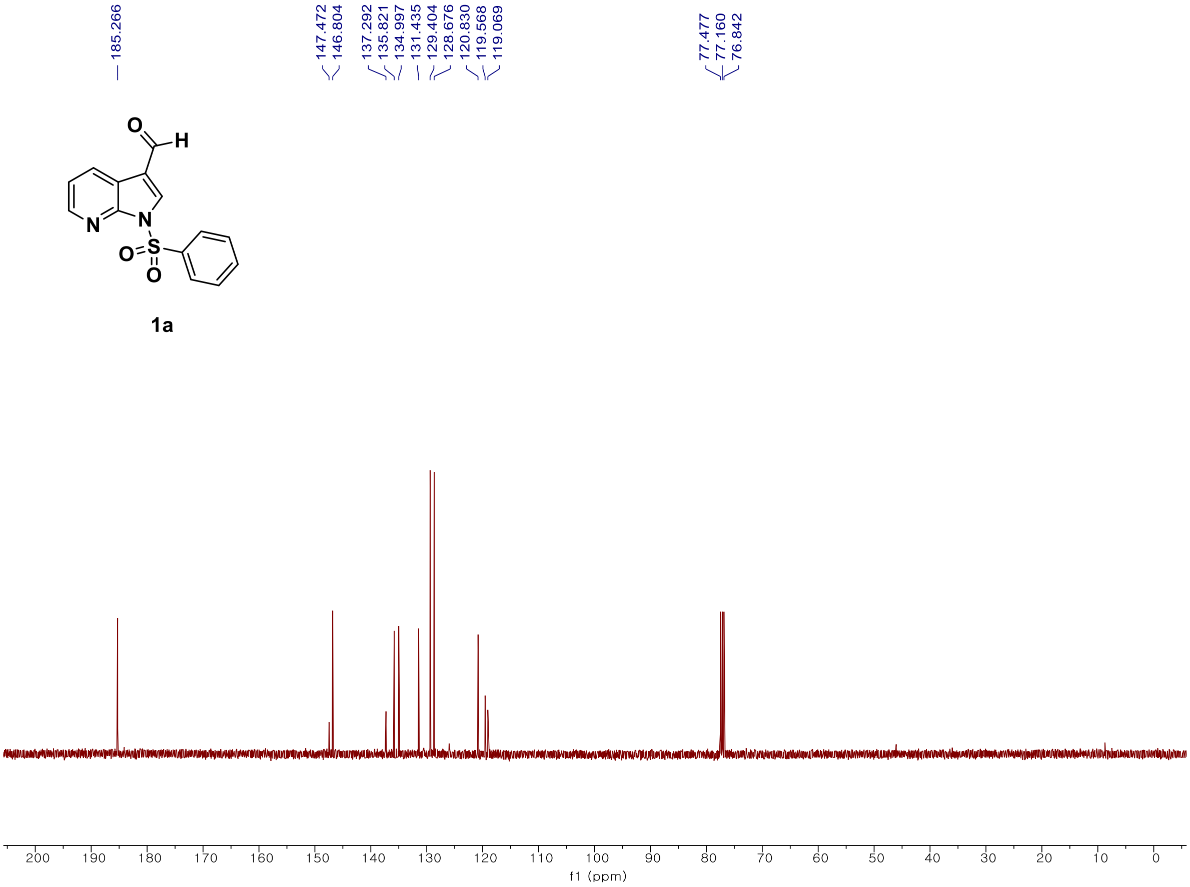

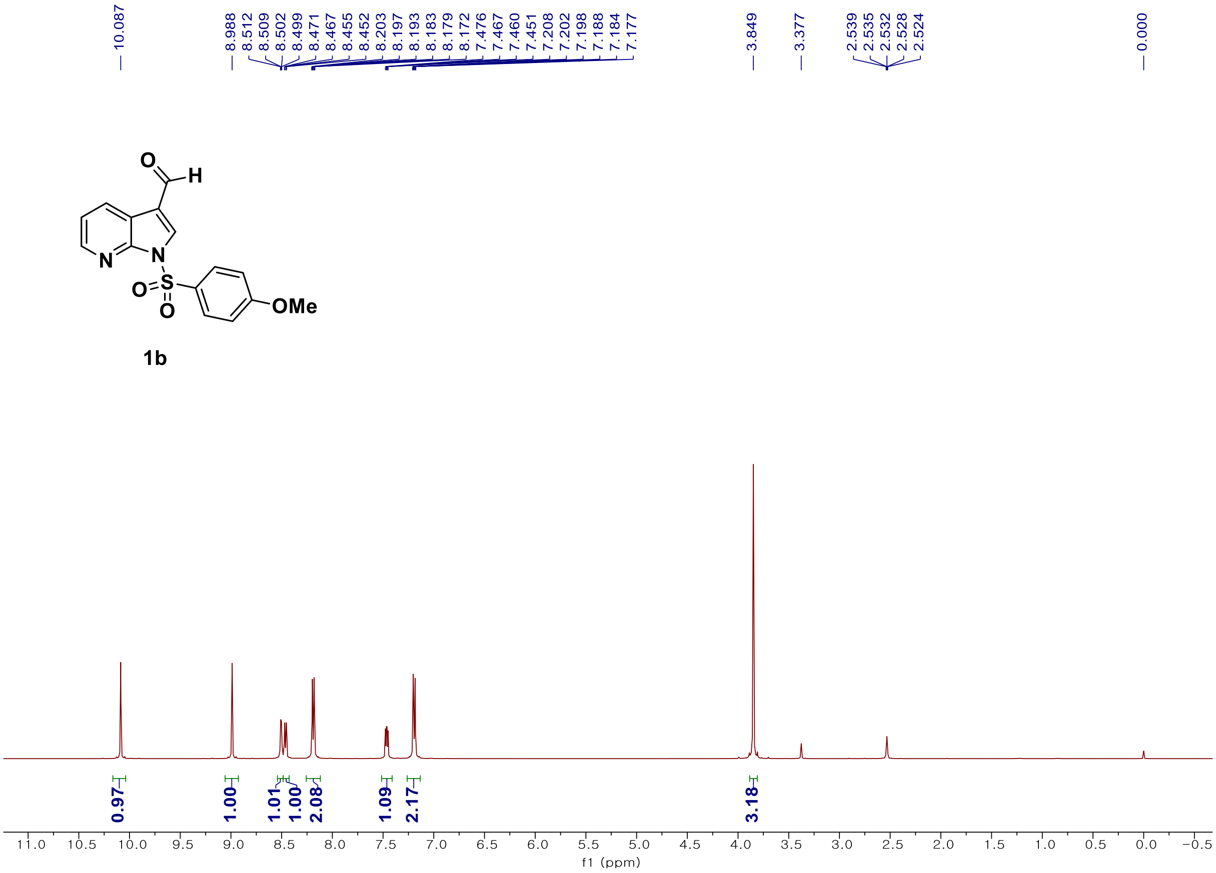
10.087
8.988
8.512
8.509
8.502
8.499
8.471
8.467
8.455
8.452
8.203
8.197
8.193
8.183
8.179
8.172
7.476
7.467
7.460
7.451
7.208
7.202
7.198
7.188
7.184
7.177
3.849
3.377
2.539
2.535
2.532
2.528
2.524
0.000
O
H
N
N
O
S
O
OMe
1b
0.97
1.00
1.01
1.00
2.08
1.09
2.17
3.18
11.0 10.5 10.0 9.5 9.0 8.5 8.0 7.5 7.0 6.5 6.0 5.5 5.0 4.5 4.0 3.5 3.0 2.5 2.0 1.5 1.0 0.5 0.0 −0.5
f1 (ppm)

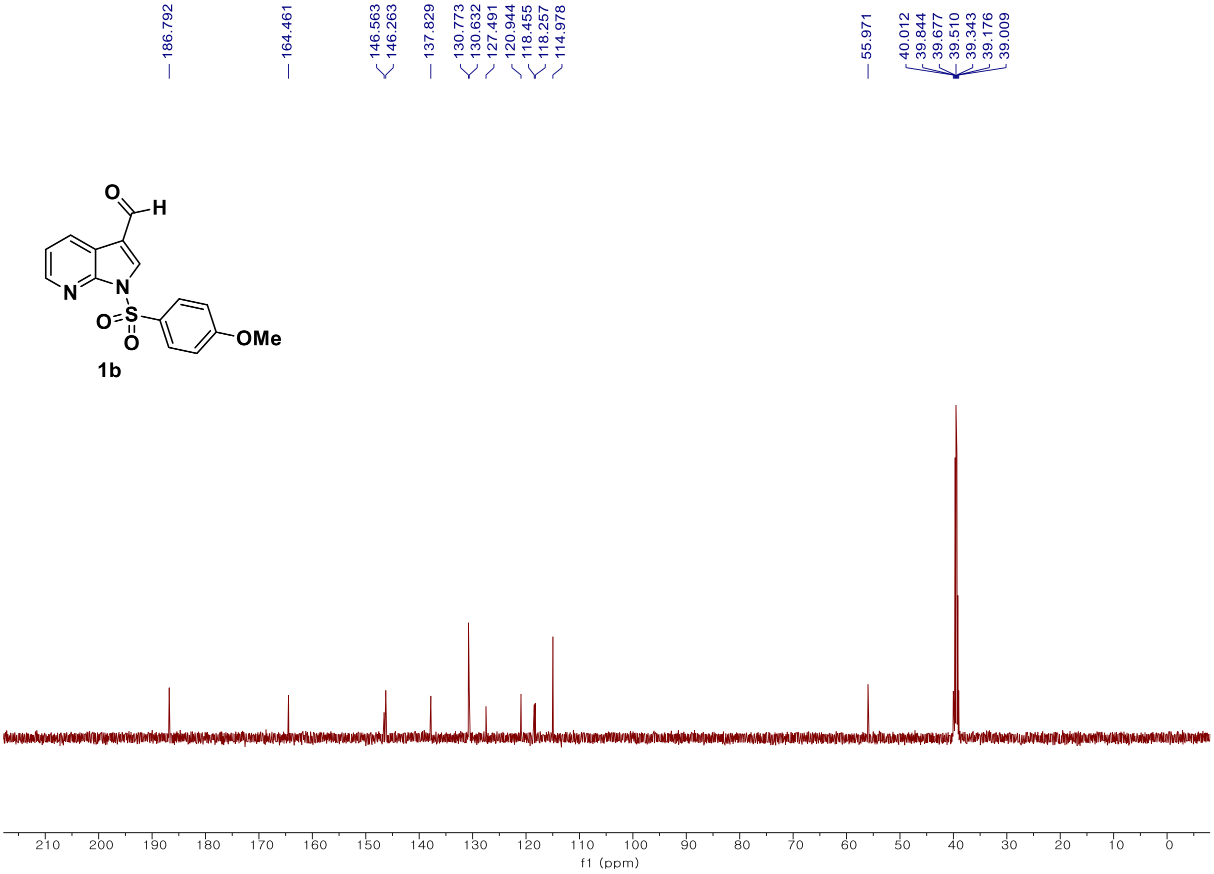
186.792
164.461
146.563
146.263
137.829
130.773
130.632
127.491
120.944
118.455
118.257
114.978
55.971
40.012
39.844
39.677
39.510
39.343
39.176
39.009
O
H
N
N
O
S
O
OMe
1b
210 200 190 180 170 160 150 140 130 120 110 100 90 80 70 60 50 40 30 20 10 0
f1 (ppm)

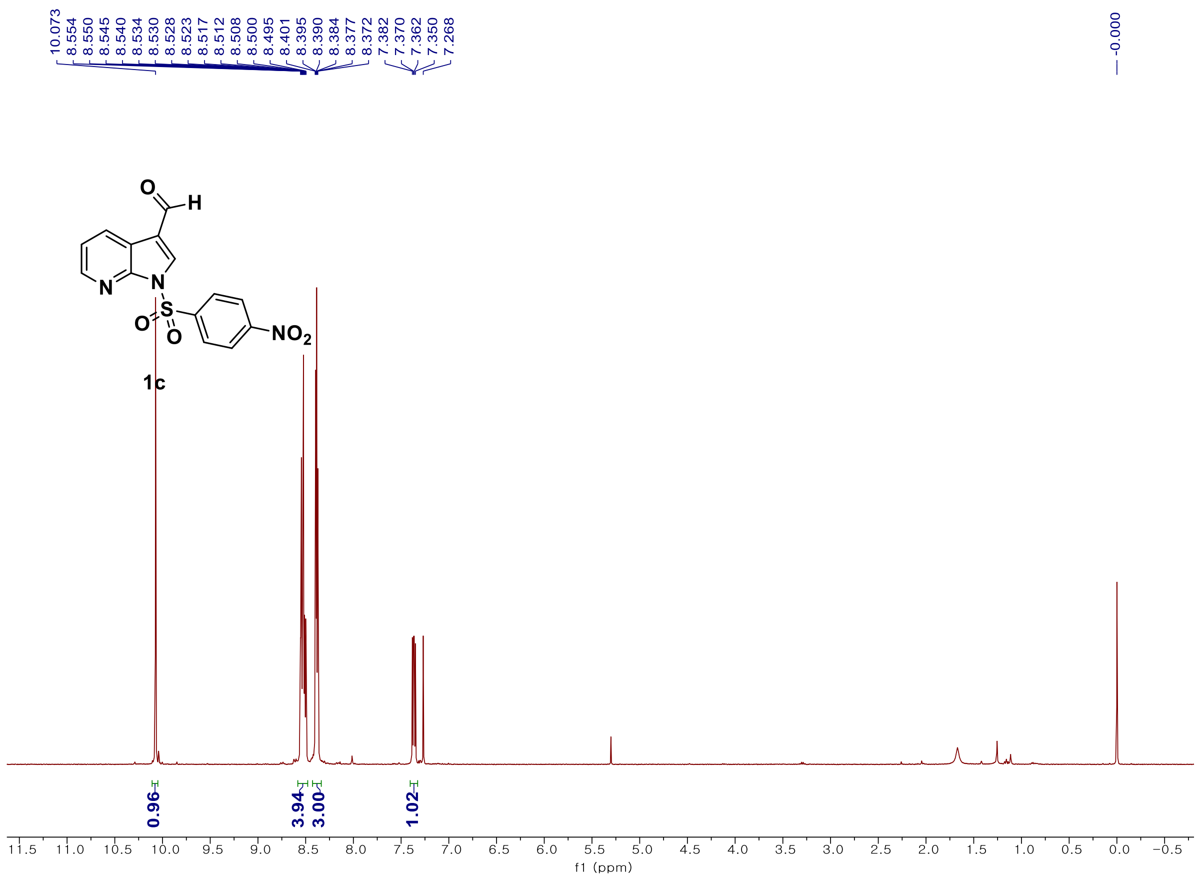
10.073
8.554
8.550
8.545
8.540
8.534
8.530
8.528
8.523
8.517
8.512
8.508
8.500
8.495
8.401
8.395
8.390
8.384
8.377
8.372
7.382
7.370
7.362
7.350
7.268
-0.000
1c
0.96
3.94
3.00
1.02
f1 (ppm)

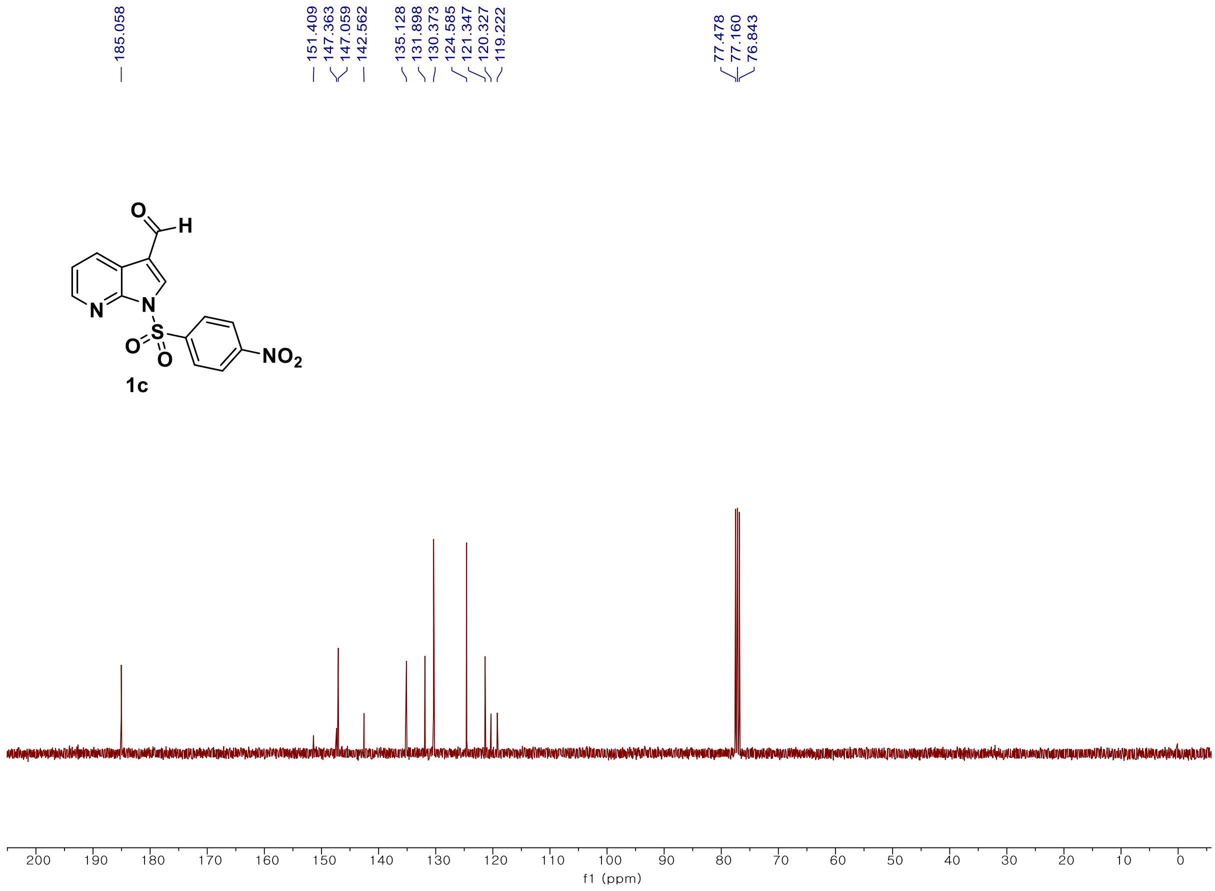
185.058
151.409
147.363
147.059
142.562
135.128
131.898
130.373
124.585
121.347
120.327
119.222
77.478
77.160
76.843
1c
f1 (ppm)

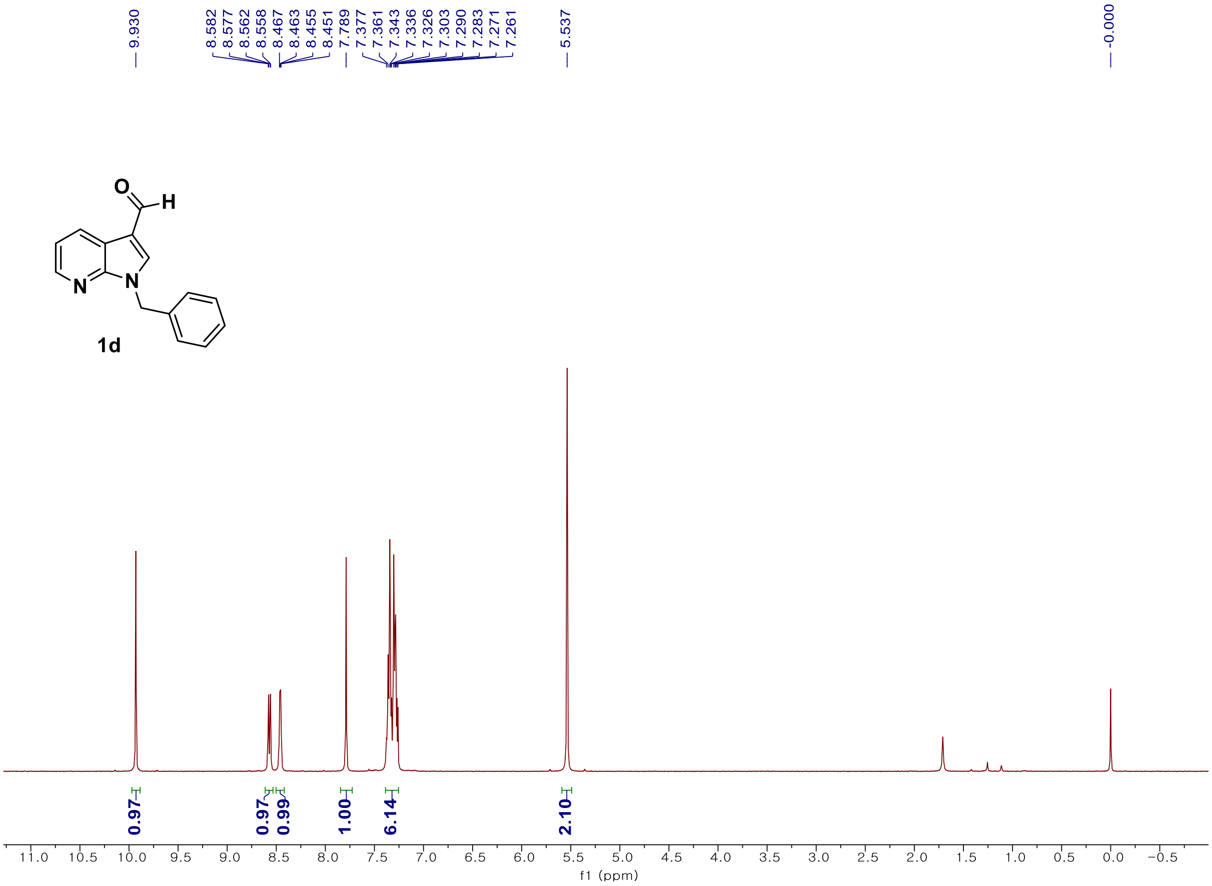
9.930
8.582
8.577
8.562
8.558
8.467
8.463
8.455
8.451
7.789
7.377
7.361
7.343
7.336
7.326
7.303
7.290
7.283
7.271
7.261
5.537
-0.000
1d
0.97
0.97
0.99
1.00
6.14
2.10
f1 (ppm)

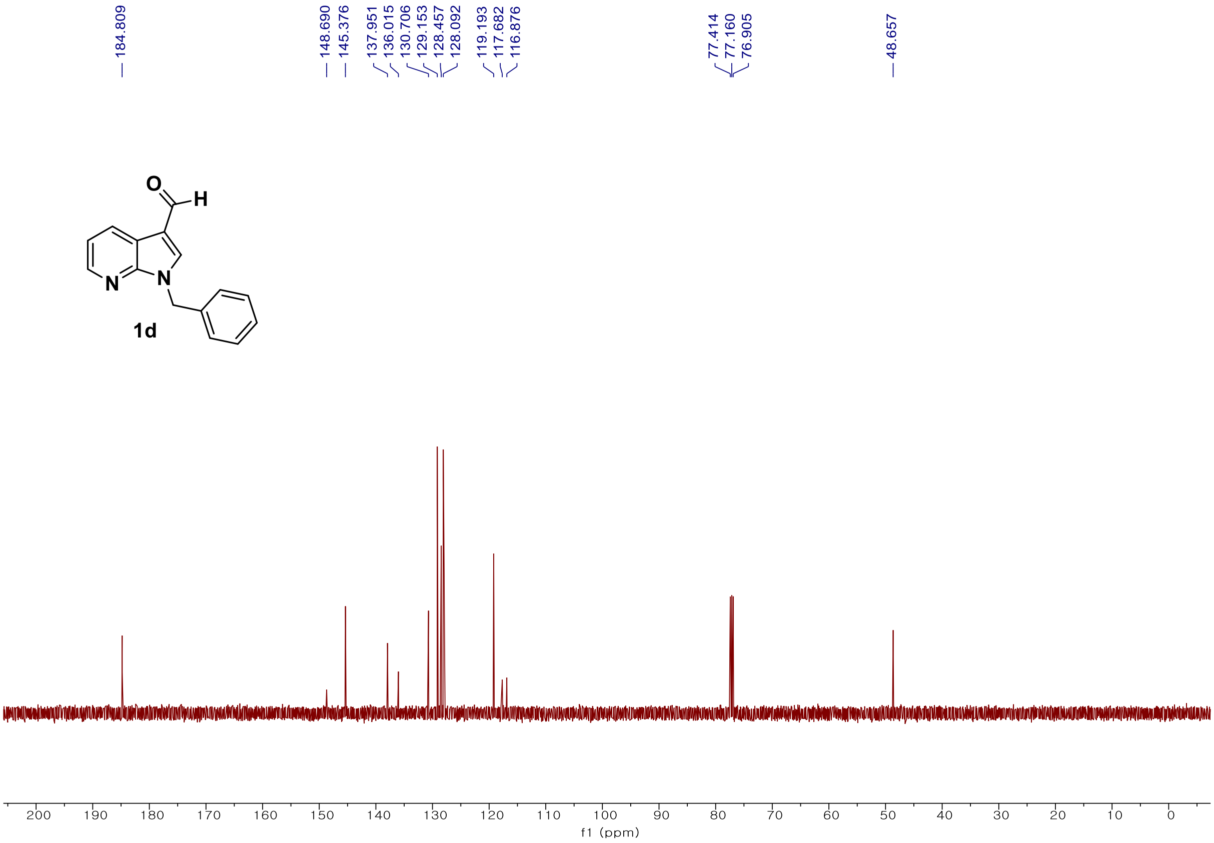
184.809
148.690
145.376
137.951
136.015
130.706
129.153
128.457
128.092
119.193
117.682
116.876
77.414
77.160
76.905
48.657
1d
f1 (ppm)

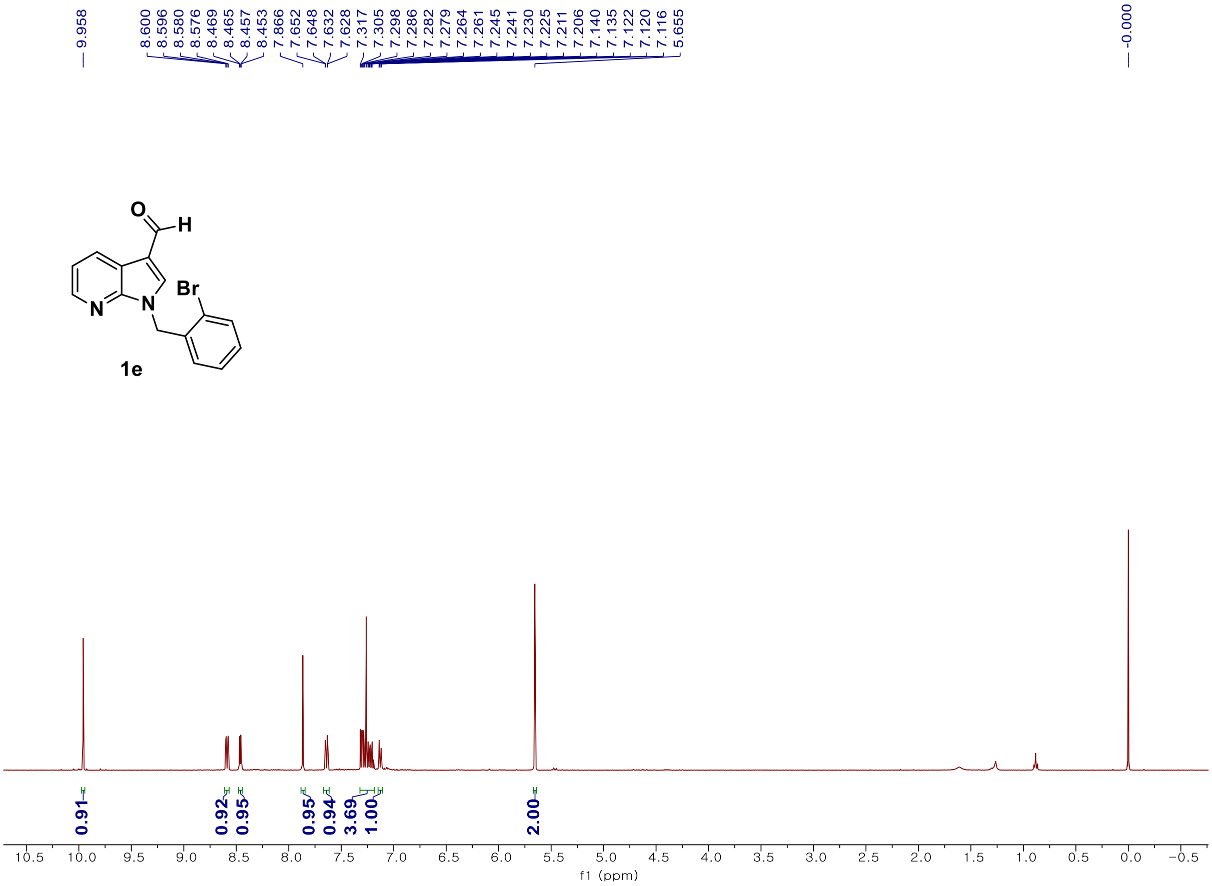
9.958
8.600 8.596 8.580 8.576 8.469 8.465 8.457 8.453 7.866 7.652 7.648 7.632 7.628 7.317 7.305 7.298 7.286 7.282 7.279 7.264 7.261 7.245 7.241 7.230 7.225 7.211 7.206 7.140 7.135 7.122 7.120 7.116 5.655
-0.000
O
H
Br
N
N
1e
0.91
0.92
0.95
0.95
0.94
3.69
1.00
2.00
f1 (ppm)

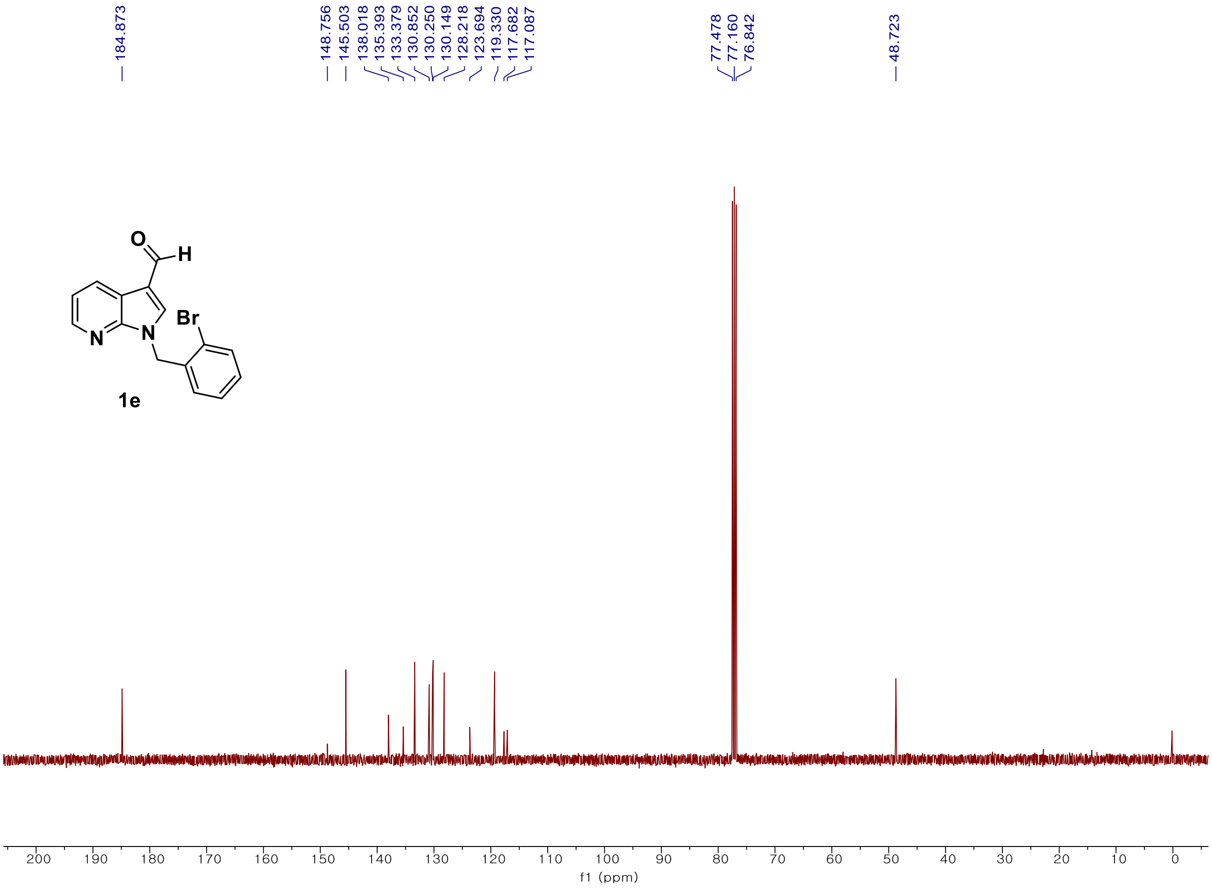
184.873
148.756 145.503 138.018 135.393 133.379 130.852 130.250 130.149 128.218 123.694 119.330 117.682 117.087
77.478 77.160 76.842
48.723
O
H
Br
N
N
1e
f1 (ppm)

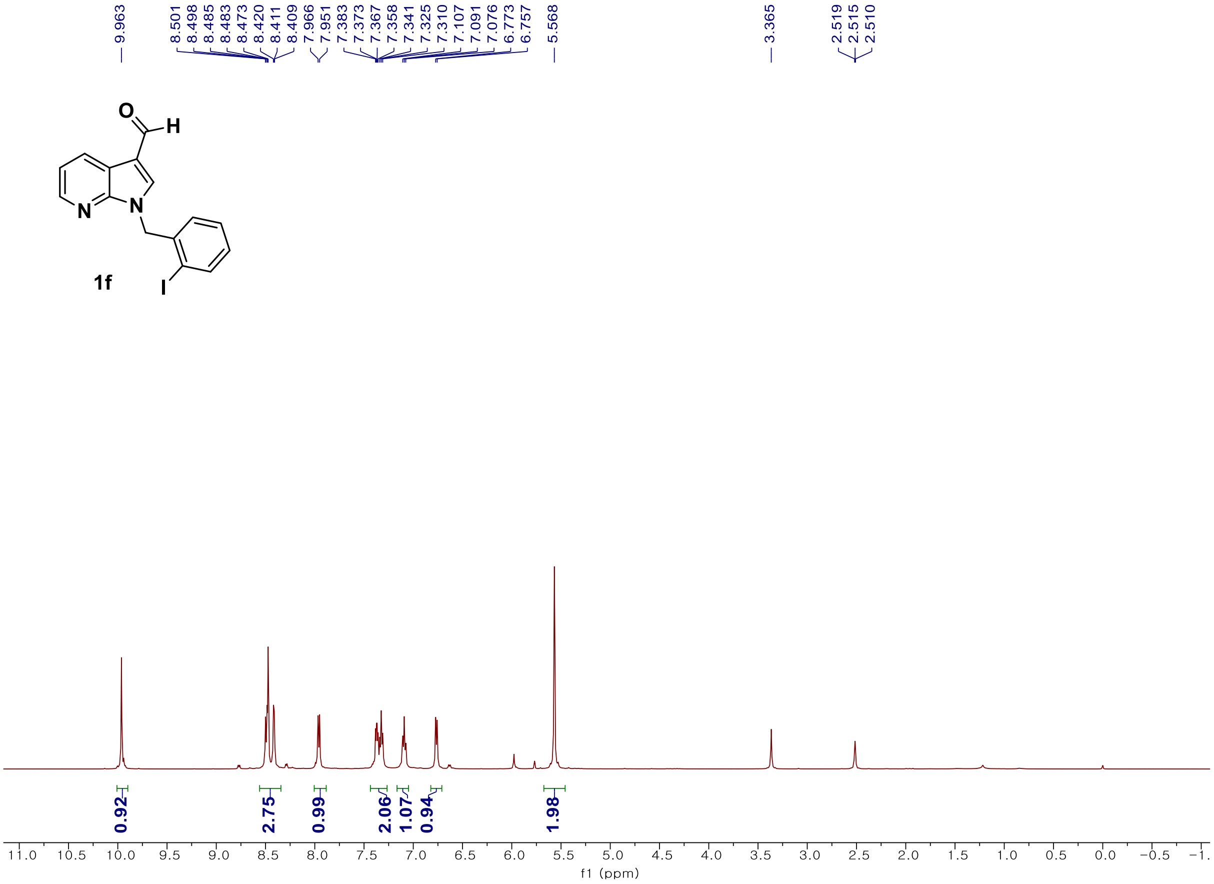
9.963
8.501 8.498 8.485 8.483 8.473 8.420 8.411 8.409 7.966 7.951 7.383 7.373 7.367 7.358 7.341 7.325 7.310 7.107 7.091 7.076 6.773 6.757
5.568
3.365
2.519 2.515 2.510
O
H
N
N
1f
I
0.92
2.75
0.99
2.06
1.07
0.94
1.98
f1 (ppm)

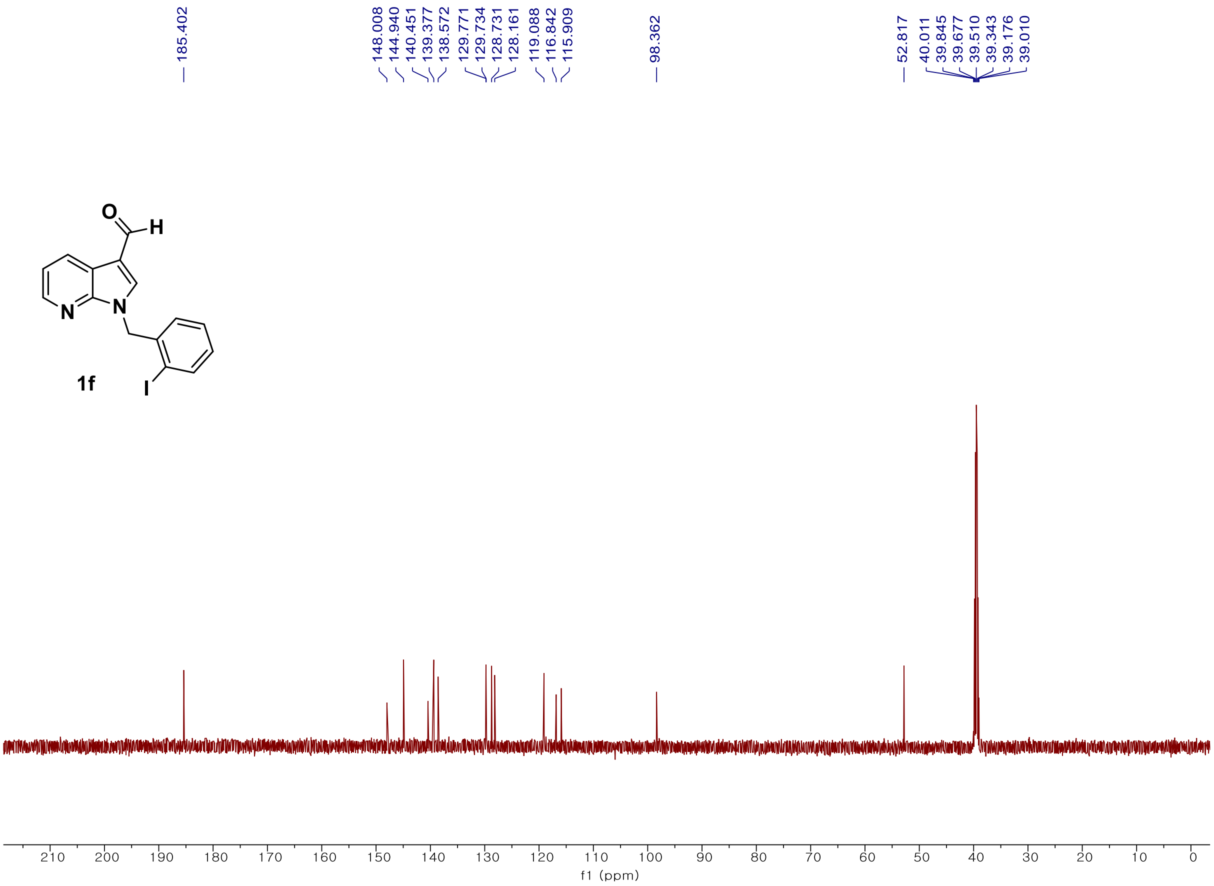
185.402
148.008 144.940 140.451 139.377 138.572 129.771 129.734 128.731 128.161 119.088 116.842 115.909
98.362
52.817
40.011 39.845 39.677 39.510 39.343 39.176 39.010
O
H
N
N
1f
I
f1 (ppm)

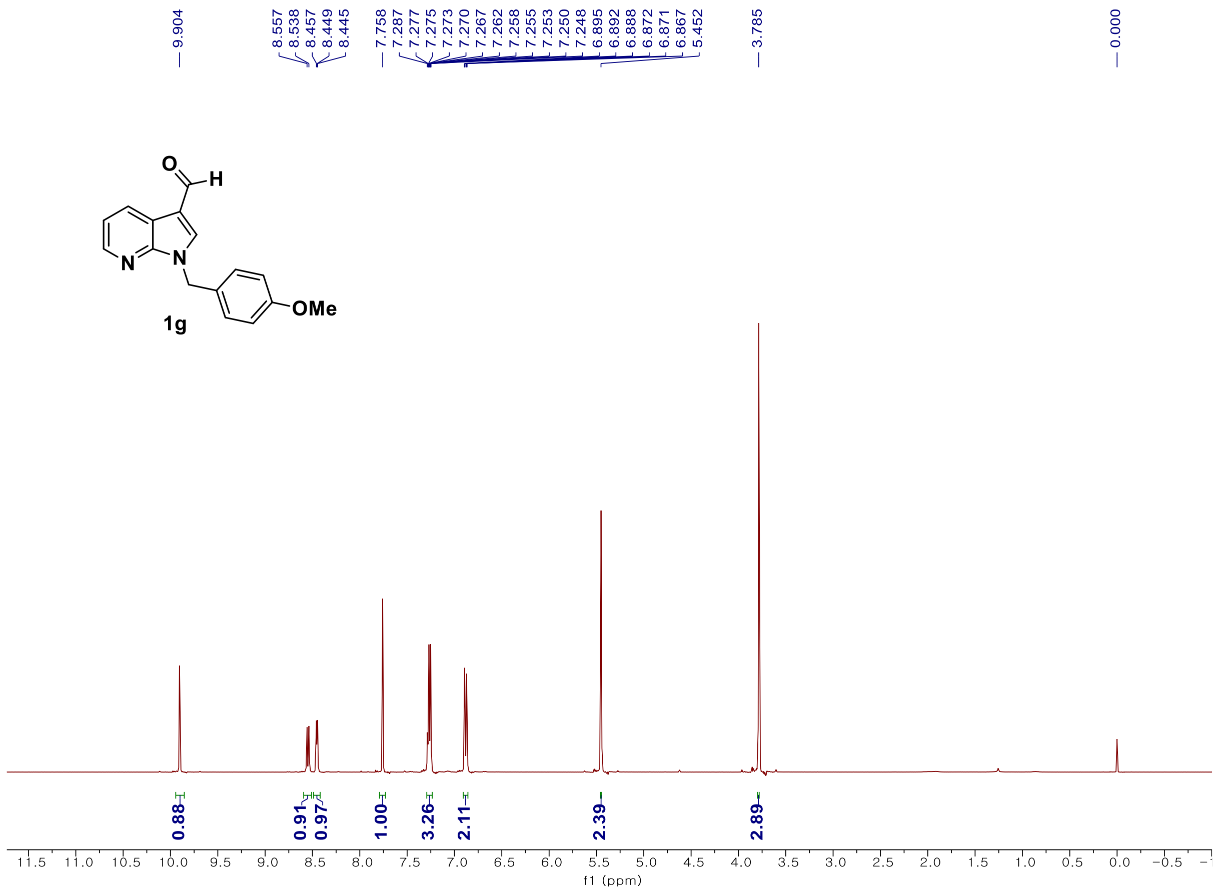
9.904
8.557
8.538
8.457
8.449
8.445
7.758
7.287
7.277
7.275
7.273
7.270
7.267
7.262
7.258
7.255
7.253
7.250
7.248
6.895
6.892
6.888
6.872
6.871
6.867
5.452
3.785
0.000
O
H
N
N
OMe
1g
0.88
0.91
0.97
1.00
3.26
2.11
2.39
2.89
f1 (ppm)

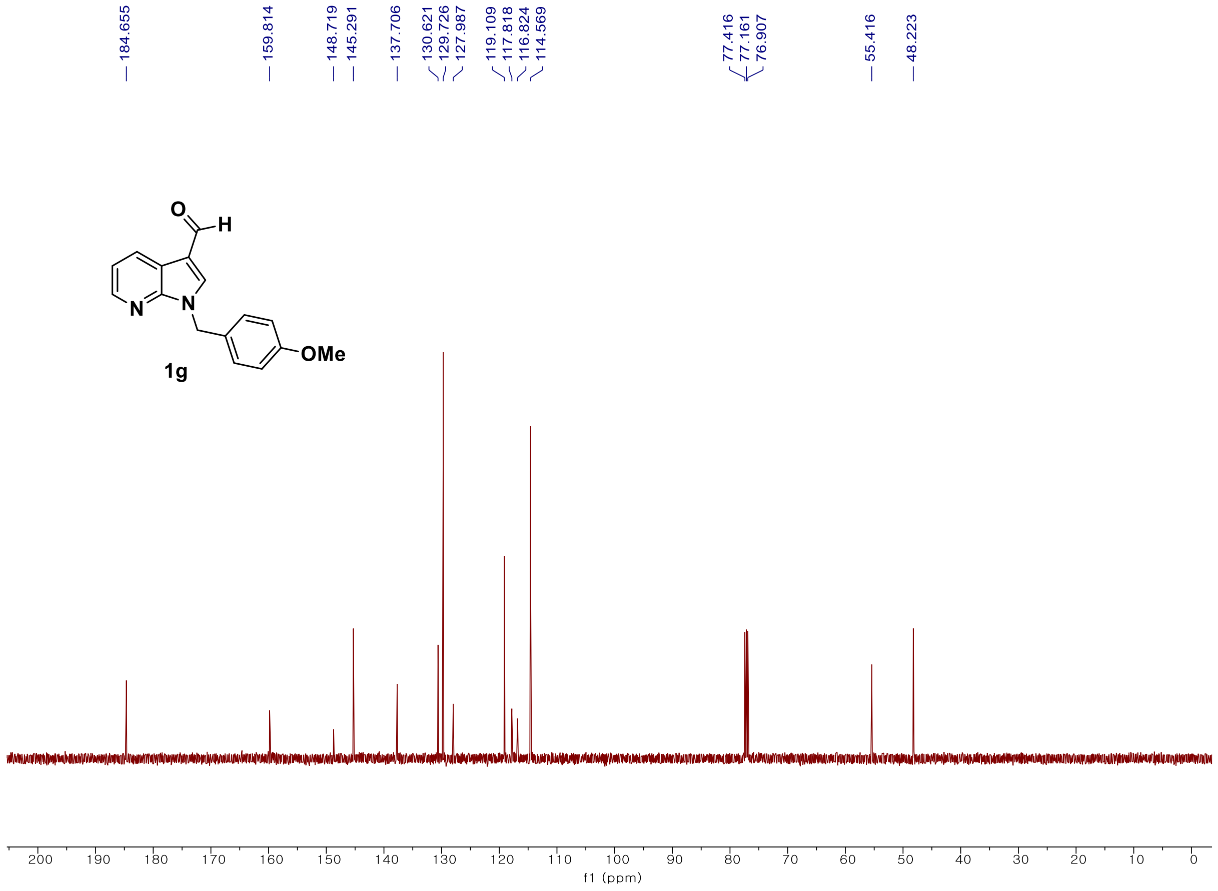
184.655
159.814
148.719
145.291
137.706
130.621
129.726
127.987
119.109
117.818
116.824
114.569
77.416
77.161
76.907
55.416
48.223
O
H
N
N
OMe
1g
f1 (ppm)

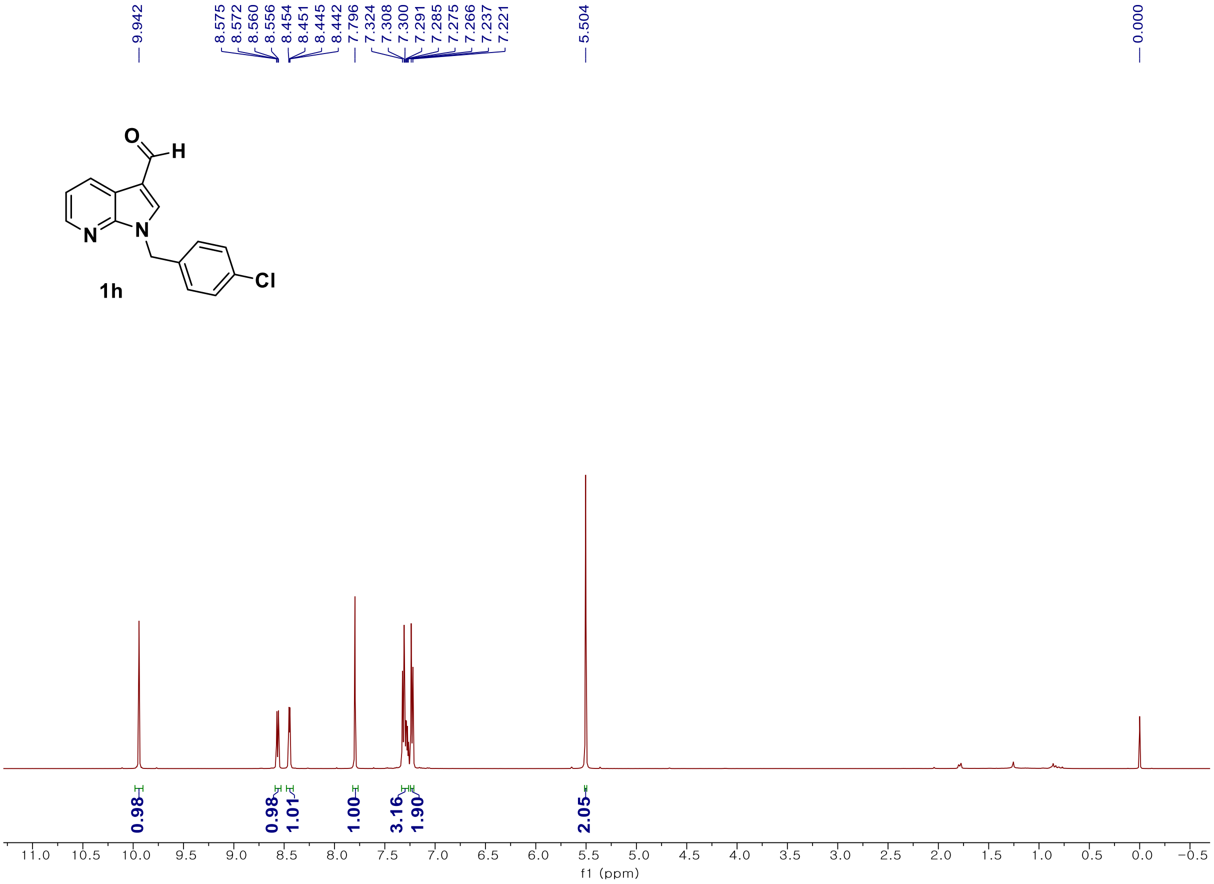
9.942
8.575
8.572
8.560
8.556
8.454
8.451
8.445
8.442
7.796
7.324
7.308
7.300
7.291
7.285
7.275
7.266
7.237
7.221
5.504
0.000
O
H
N
N
Cl
1h
0.98
0.98
1.01
1.00
3.16
1.90
2.05
11.0 10.5 10.0 9.5 9.0 8.5 8.0 7.5 7.0 6.5 6.0 5.5 5.0 4.5 4.0 3.5 3.0 2.5 2.0 1.5 1.0 0.5 0.0 -0.5
f1 (ppm)

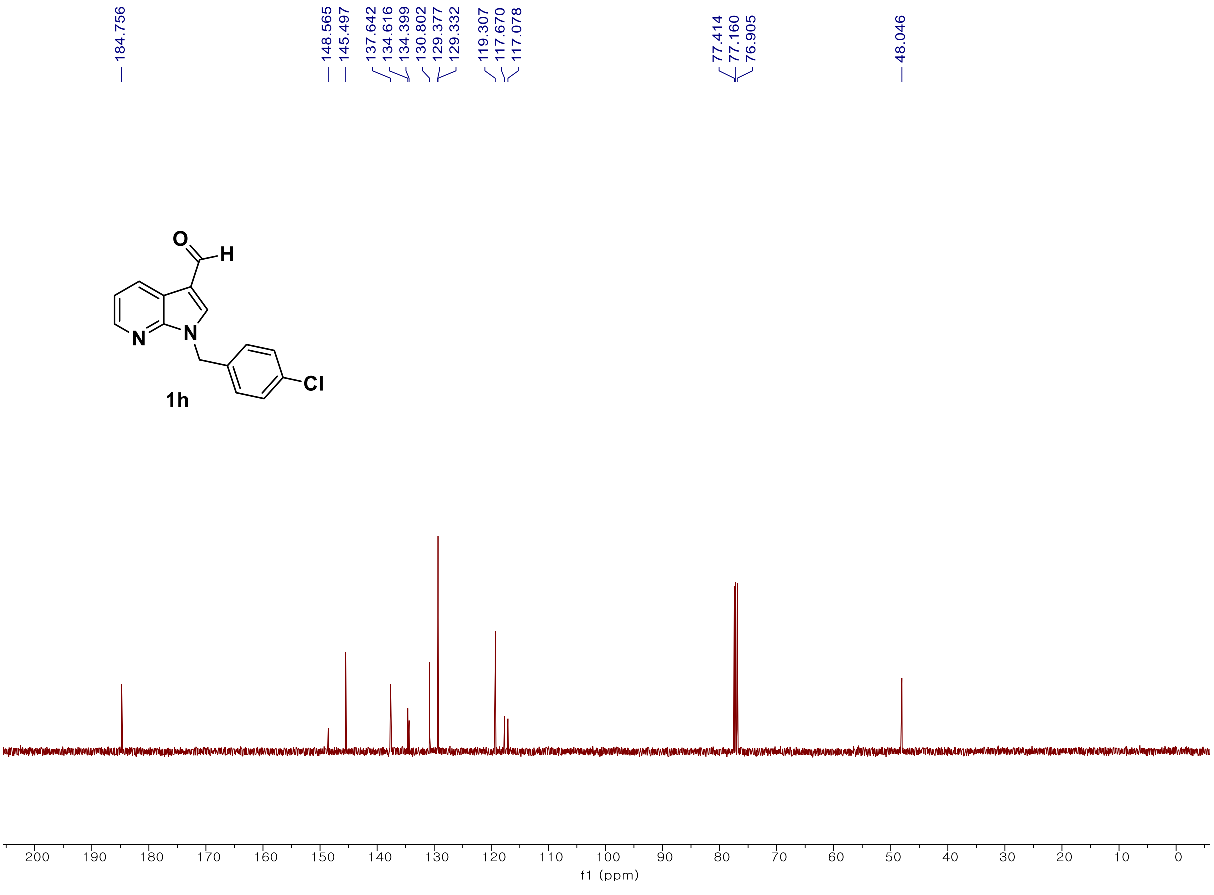
184.756
148.565
145.497
137.642
134.616
134.399
130.802
129.377
129.332
119.307
117.670
117.078
77.414
77.160
76.905
48.046
O
H
N
N
Cl
1h
200 190 180 170 160 150 140 130 120 110 100 90 80 70 60 50 40 30 20 10 0
f1 (ppm)

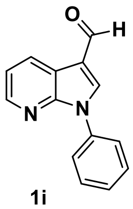

1i

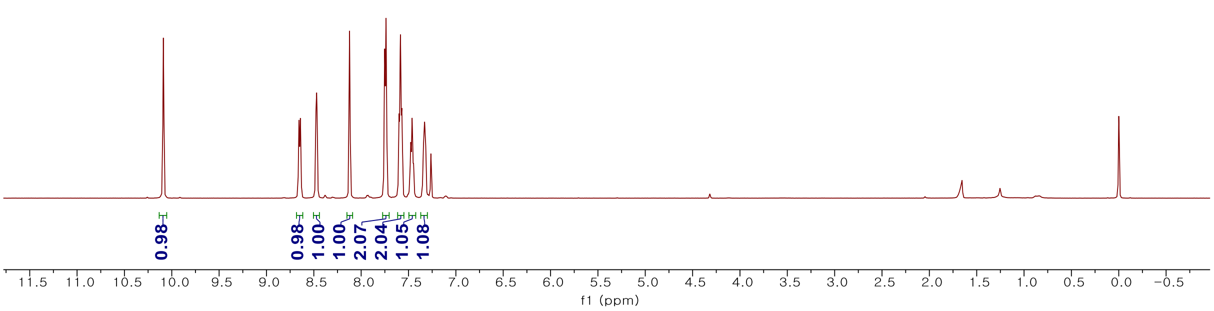

10.089
8.657
8.641
8.478
8.469
8.123
7.752
7.737
7.601
7.585
7.569
7.477
7.462
7.446
7.345
7.337
7.330
7.319
7.263
0.001
0.98
0.98
1.00
1.00
2.07
2.04
1.05
1.08
f1 (ppm)

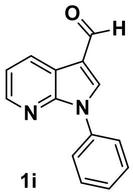

1i

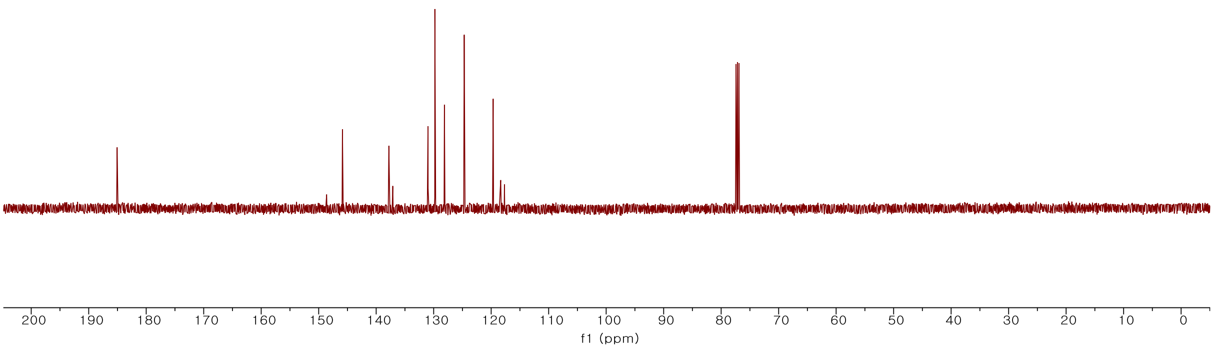

185.056
148.618
145.869
137.797
137.111
130.986
129.797
128.132
124.688
119.649
118.338
117.695
77.416
77.161
76.906
f1 (ppm)

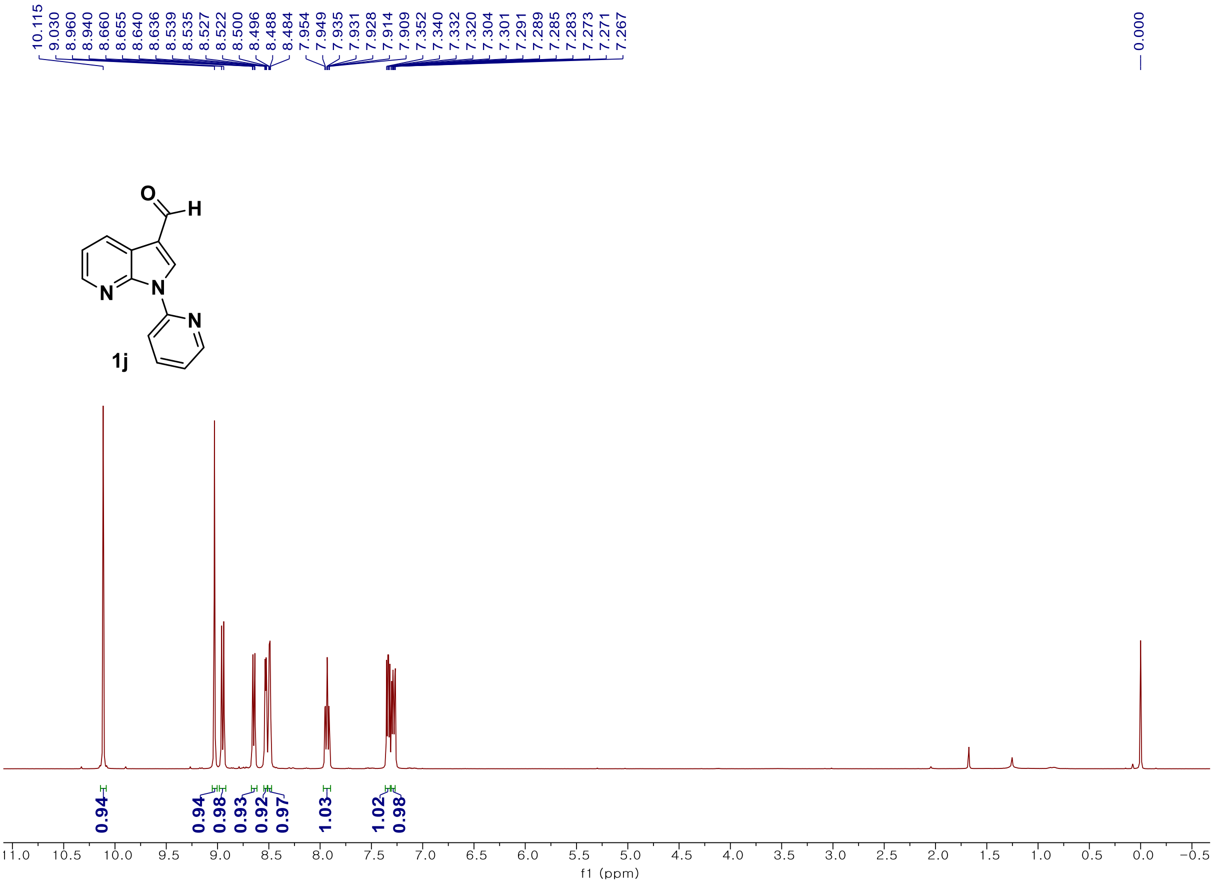
10.115
9.030
8.960
8.940
8.660
8.655
8.640
8.636
8.539
8.535
8.527
8.522
8.500
8.496
8.488
8.484
7.954
7.949
7.935
7.931
7.928
7.914
7.909
7.352
7.340
7.332
7.320
7.304
7.301
7.291
7.289
7.285
7.283
7.273
7.271
7.267
0.000
1j
0.94
0.94
0.98
0.93
0.92
0.97
1.03
1.02
0.98
f1 (ppm)

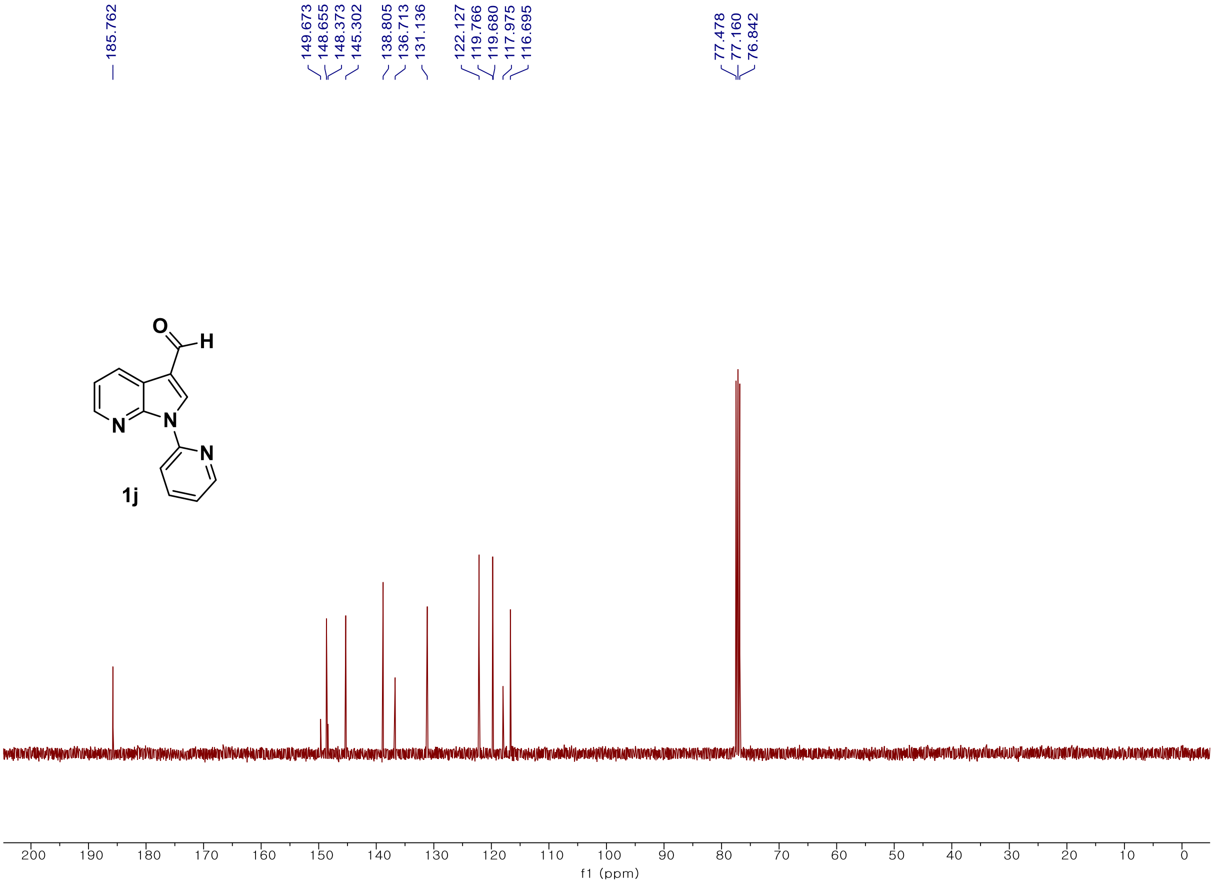
185.762
149.673
148.655
148.373
145.302
138.805
136.713
131.136
122.127
119.766
119.680
117.975
116.695
77.478
77.160
76.842
1j
f1 (ppm)

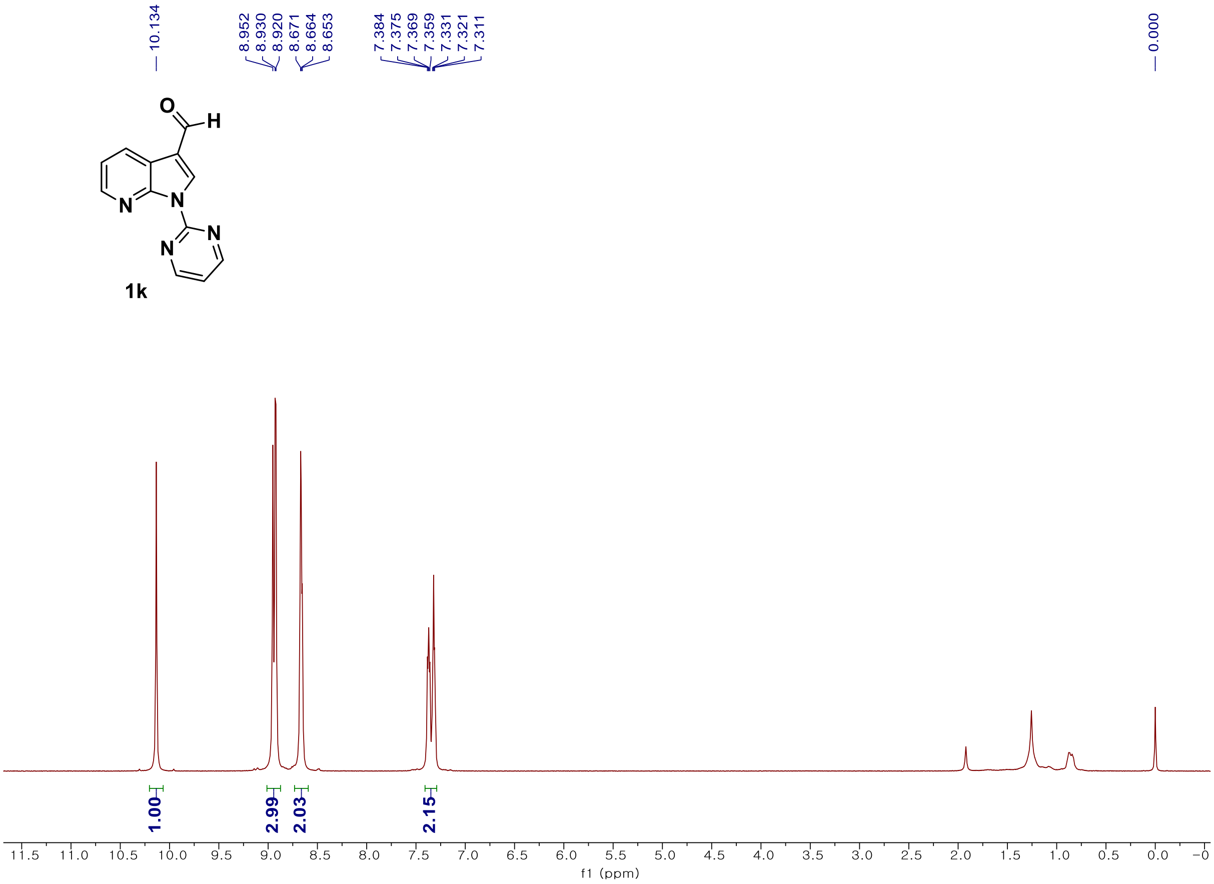
10.134
8.952
8.930
8.920
8.671
8.664
8.653
7.384
7.375
7.369
7.359
7.331
7.321
7.311
0.000
O
H
N
N
N
N
1k
1.00
2.99
2.03
2.15
f1 (ppm)

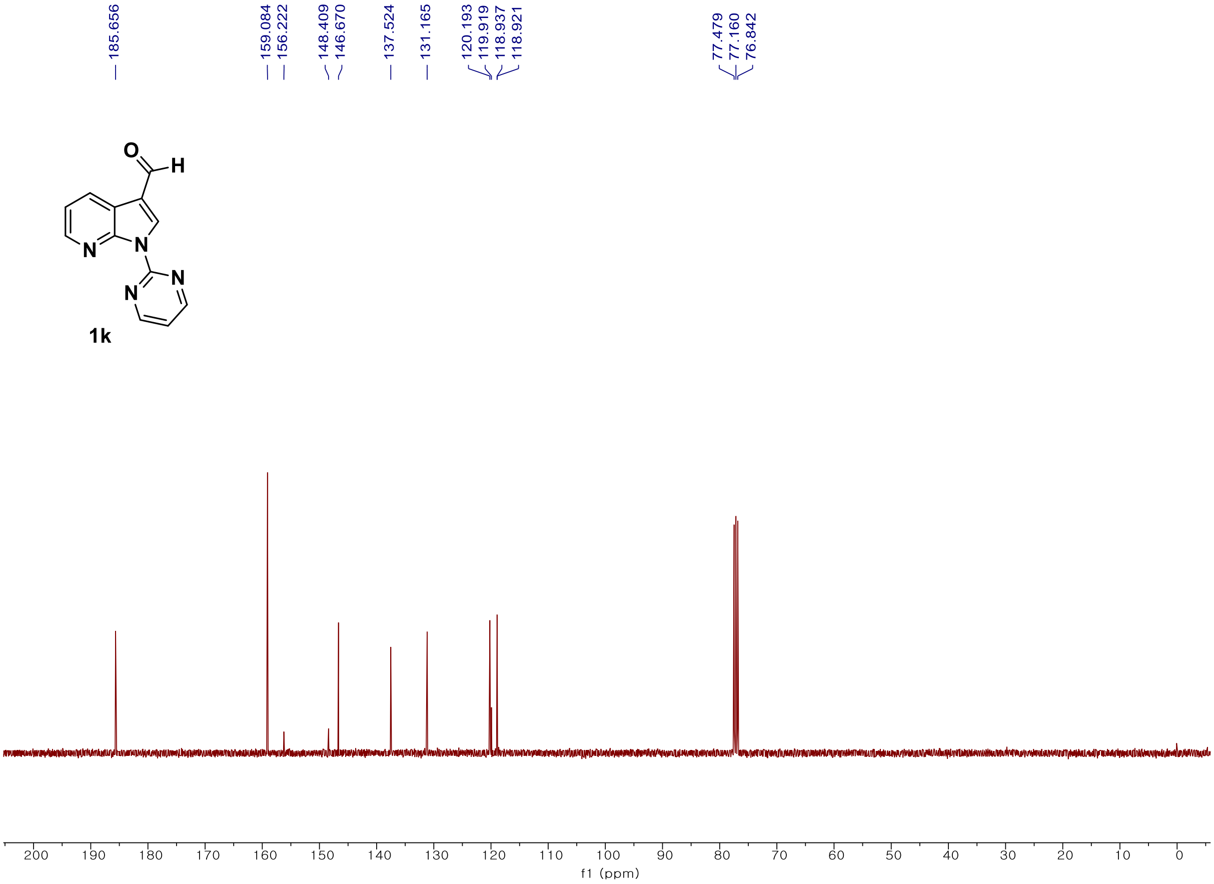
185.656
159.084
156.222
148.409
146.670
137.524
131.165
120.193
119.919
118.937
118.921
77.479
77.160
76.842
O
H
N
N
N
N
1k
f1 (ppm)

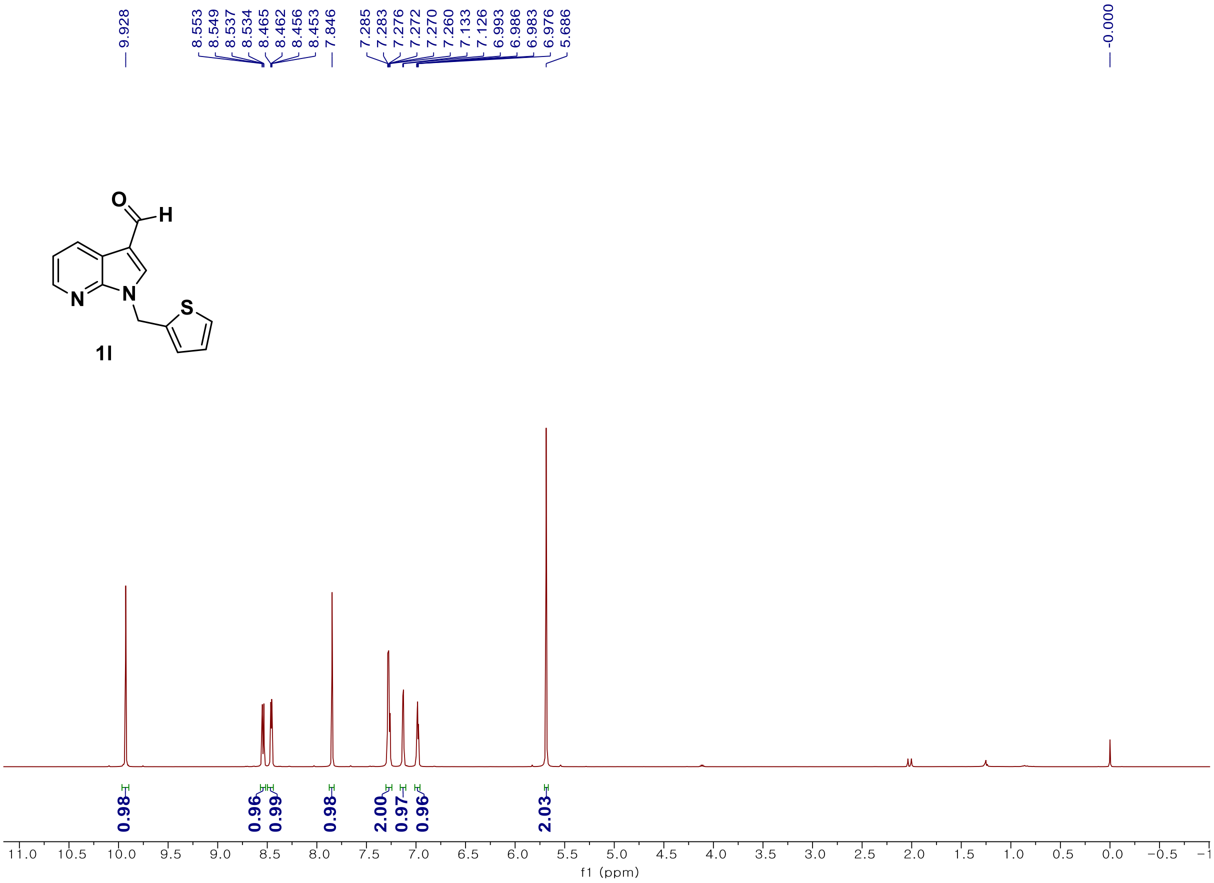
9.928
8.553
8.549
8.537
8.534
8.465
8.462
8.456
8.453
7.846
7.285
7.283
7.276
7.272
7.270
7.260
7.133
7.126
6.993
6.986
6.983
6.976
5.686
-0.000
O
H
N
N
S
1l
0.98
0.96
0.99
0.98
2.00
0.97
0.96
2.03
f1 (ppm)

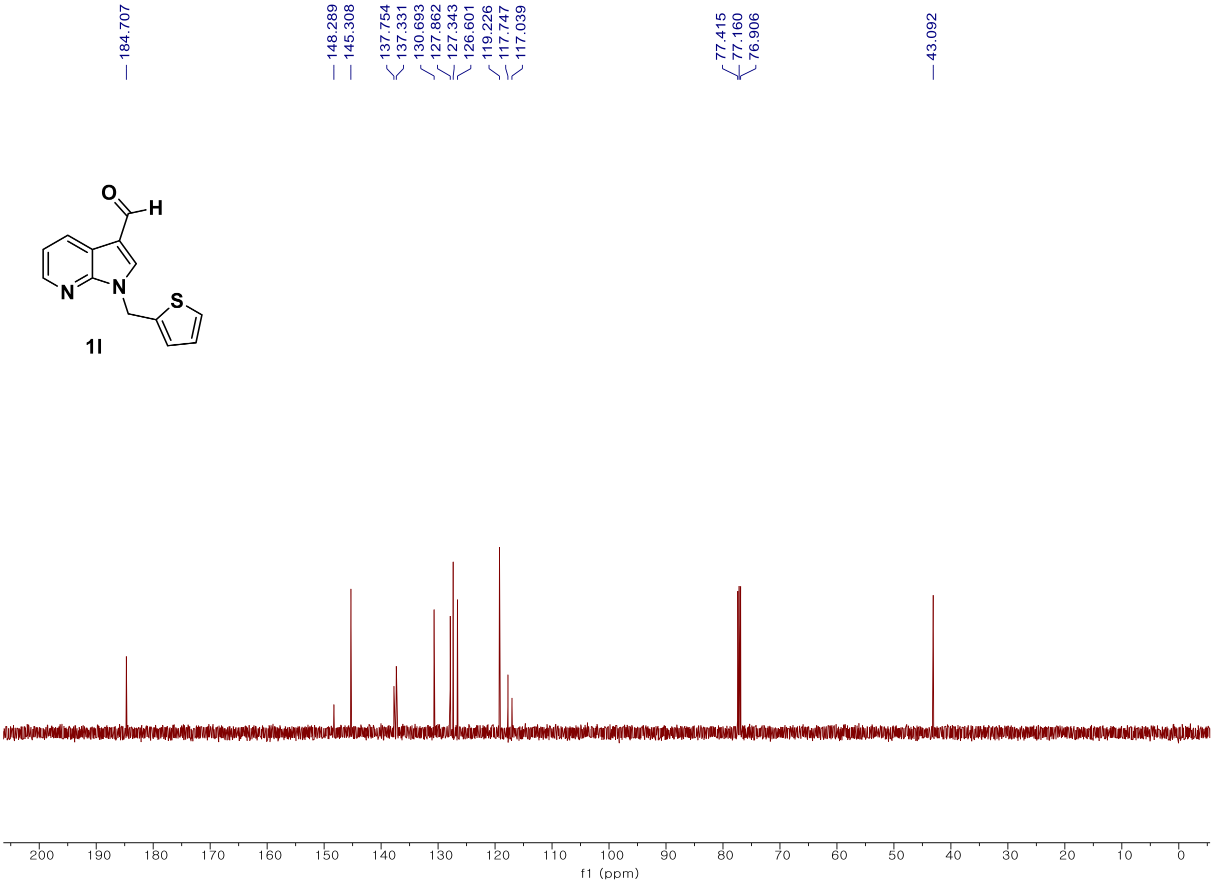
184.707
148.289
145.308
137.754
137.331
130.693
127.862
127.343
126.601
119.226
117.747
117.039
77.415
77.160
76.906
43.092
O
H
N
N
S
1l
f1 (ppm)

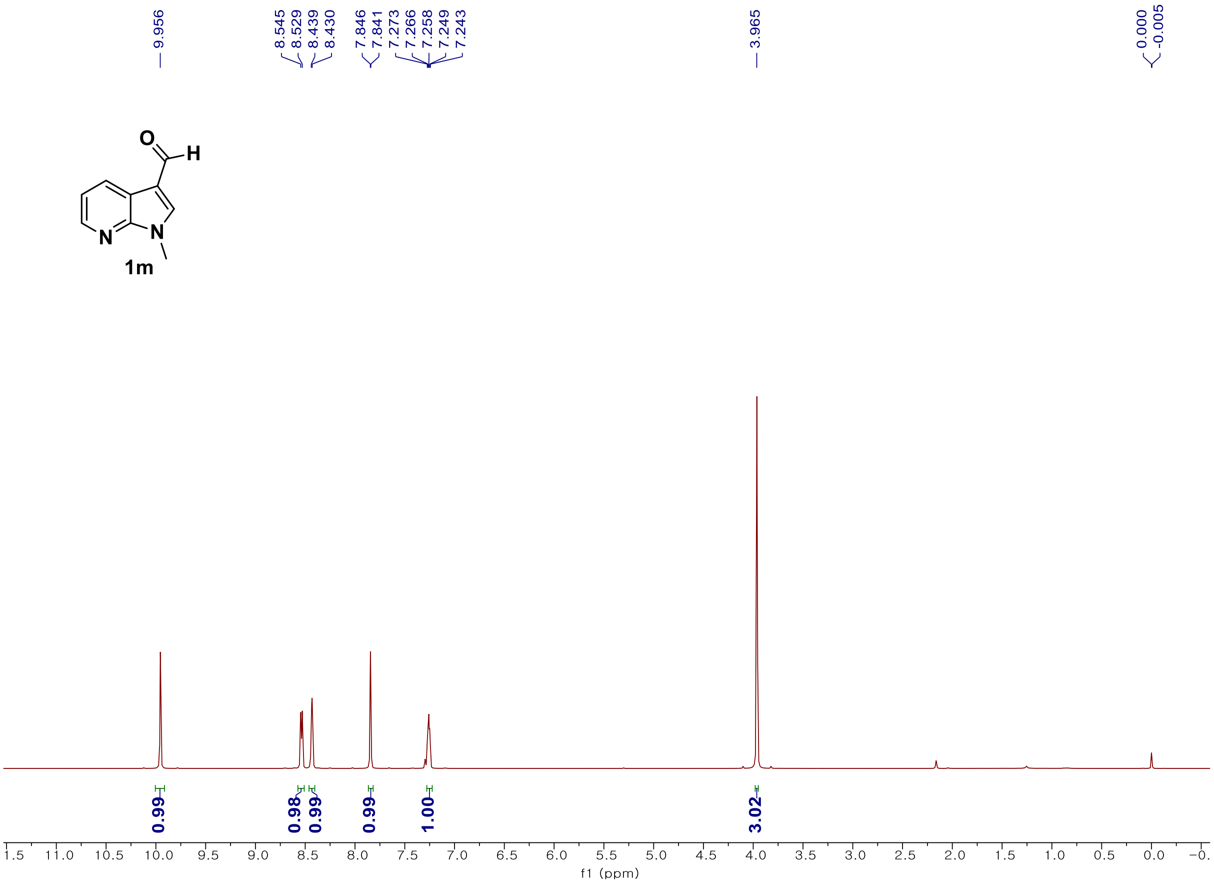
9.956
8.545
8.529
8.439
8.430
7.846
7.841
7.273
7.266
7.258
7.249
7.243
3.965
0.000
-0.005
1m
0.99
0.98
0.99
0.99
1.00
3.02
f1 (ppm)

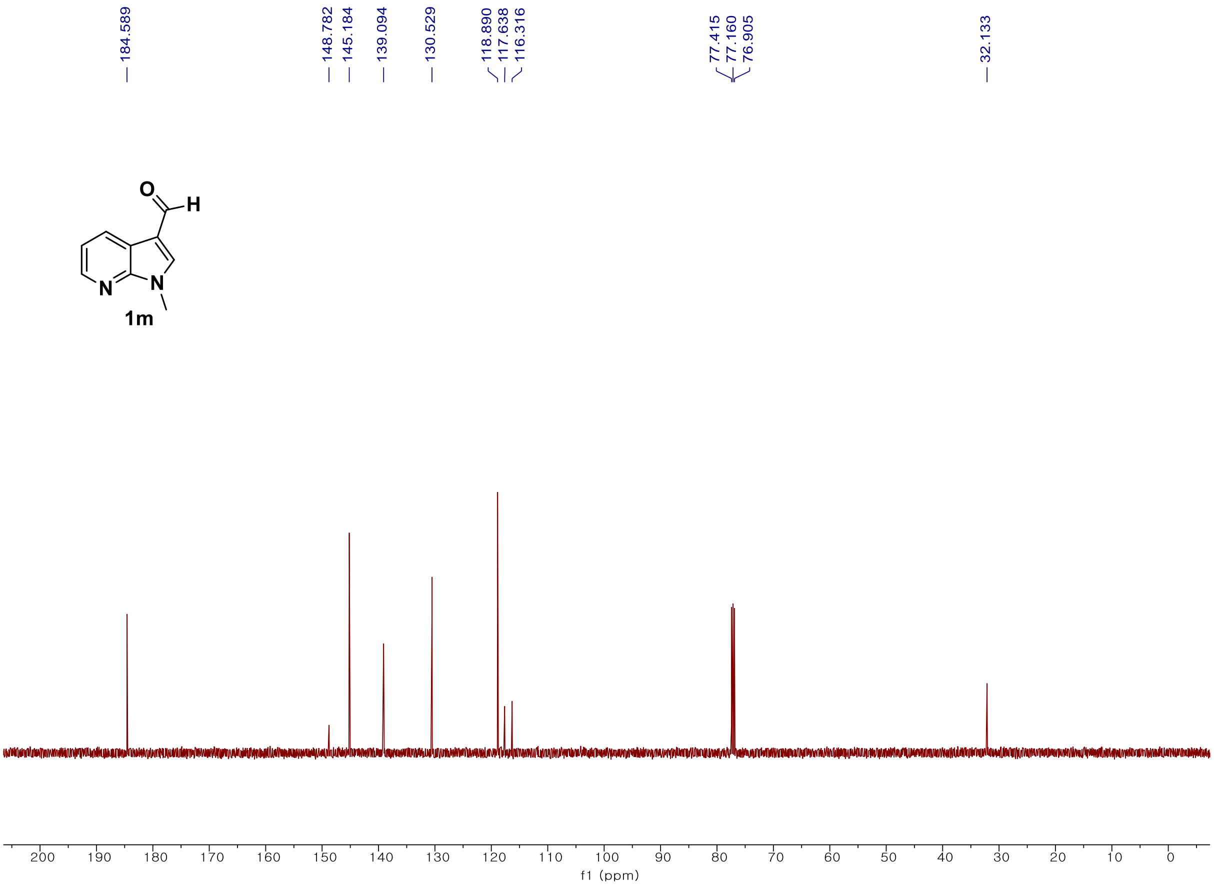
184.589
148.782
145.184
139.094
130.529
118.890
117.638
116.316
77.415
77.160
76.905
32.133
1m
f1 (ppm)

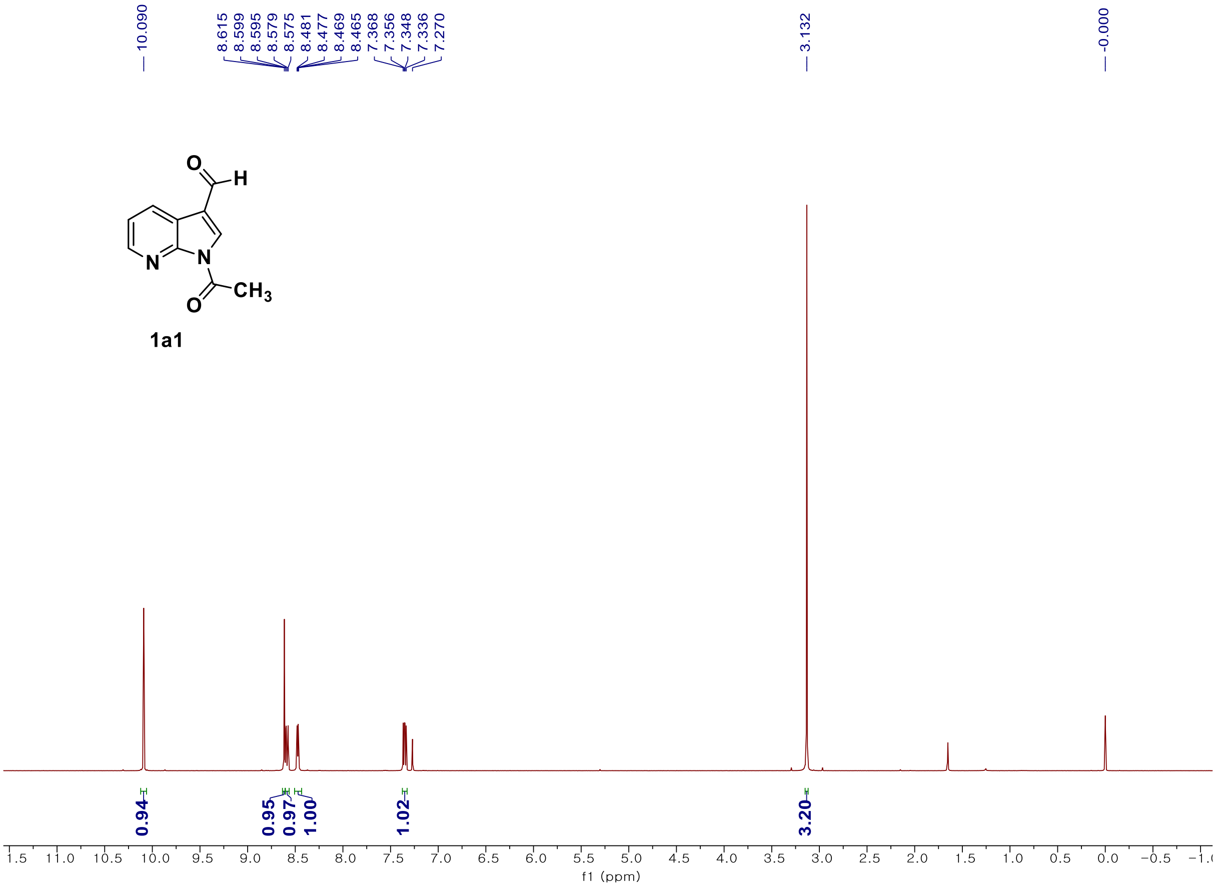
10.090
8.615
8.599
8.595
8.579
8.575
8.481
8.477
8.469
8.465
7.368
7.356
7.348
7.336
7.270
3.132
-0.000
O
H
N
N
O
CH3
1a1
0.94
0.95
0.97
1.00
1.02
3.20
f1 (ppm)

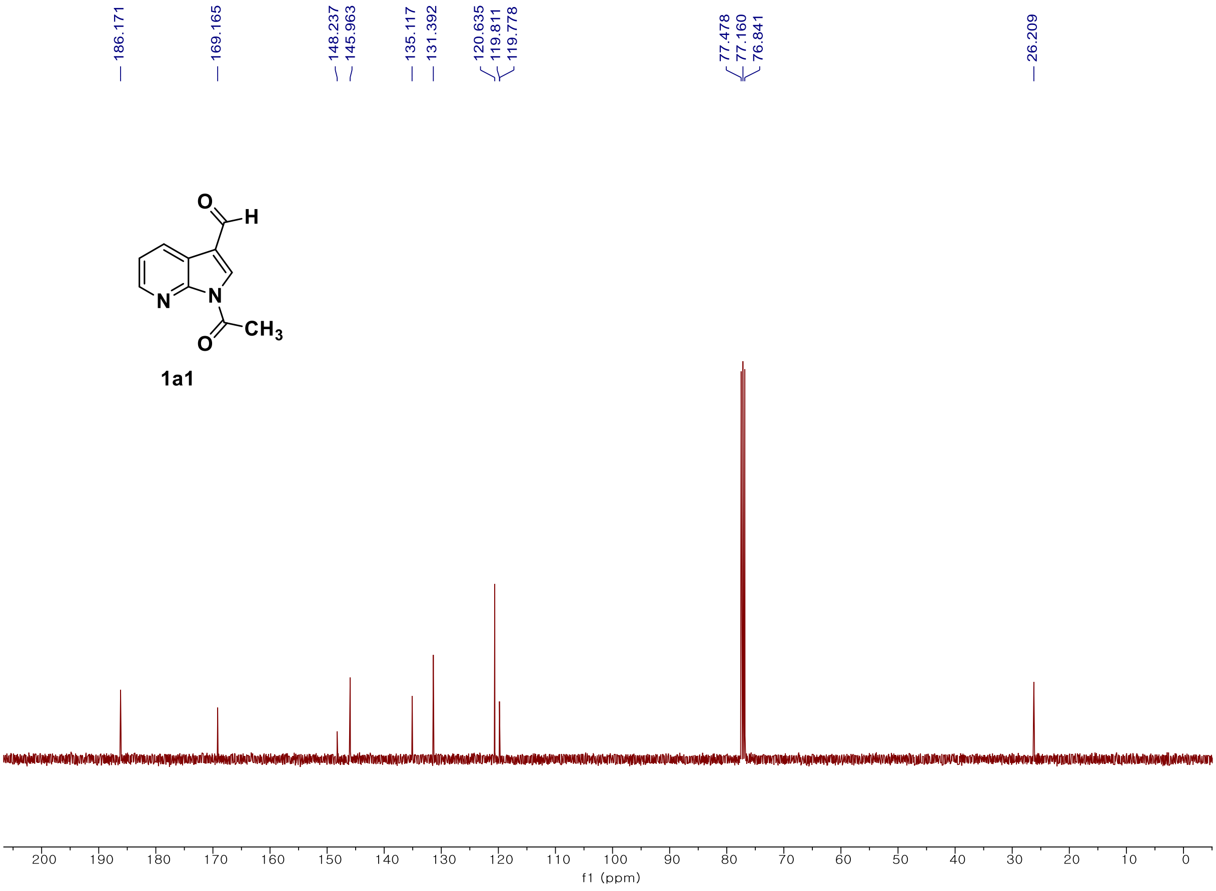
186.171
169.165
148.237
145.963
135.117
131.392
120.635
119.811
119.778
77.478
77.160
76.841
26.209
O
H
N
N
O
CH3
1a1
f1 (ppm)

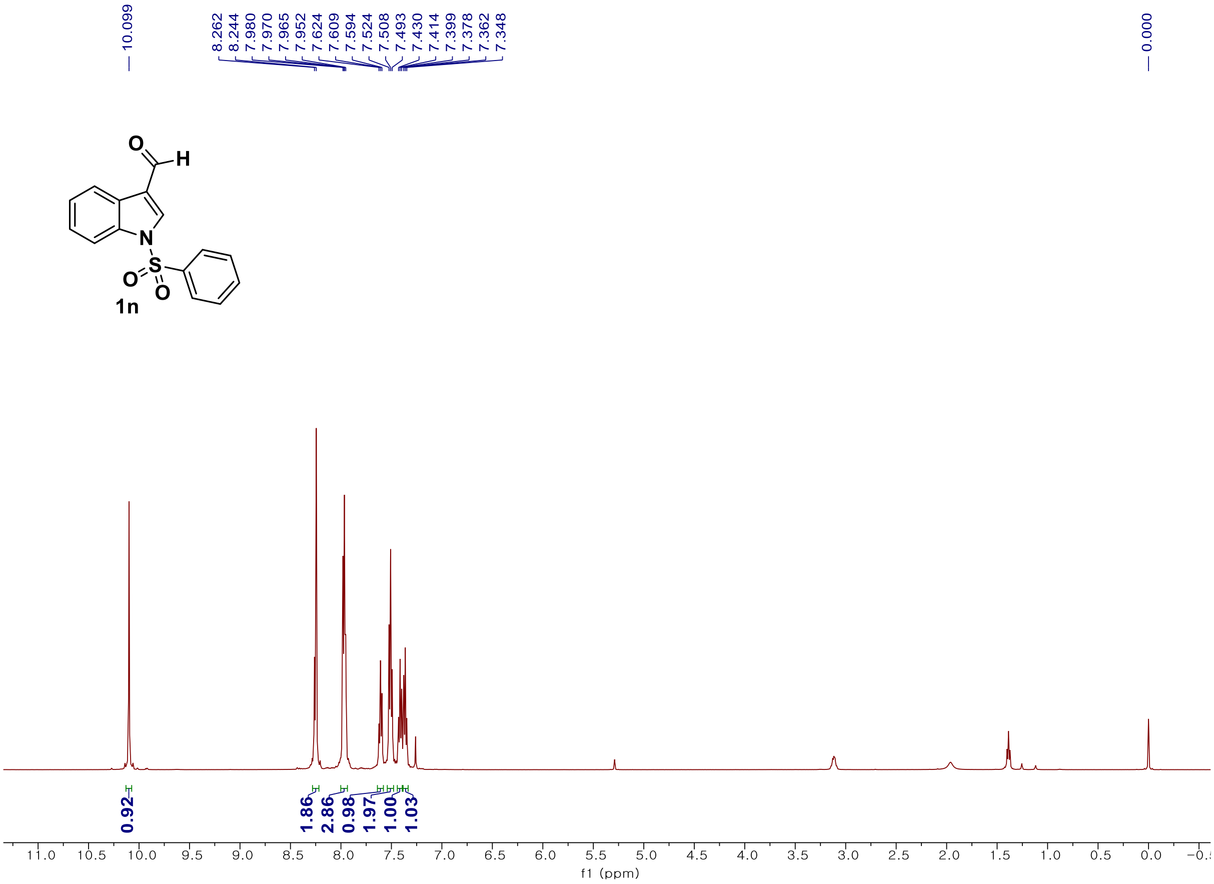
10.099
8.262
8.244
7.980
7.970
7.965
7.952
7.624
7.609
7.594
7.524
7.508
7.493
7.430
7.414
7.399
7.378
7.362
7.348
0.000
O
H
N
S
O
O
1n
0.92
1.86
2.86
0.98
1.97
1.00
1.03
11.0 10.5 10.0 9.5 9.0 8.5 8.0 7.5 7.0 6.5 6.0 5.5 5.0 4.5 4.0 3.5 3.0 2.5 2.0 1.5 1.0 0.5 0.0
f1 (ppm)

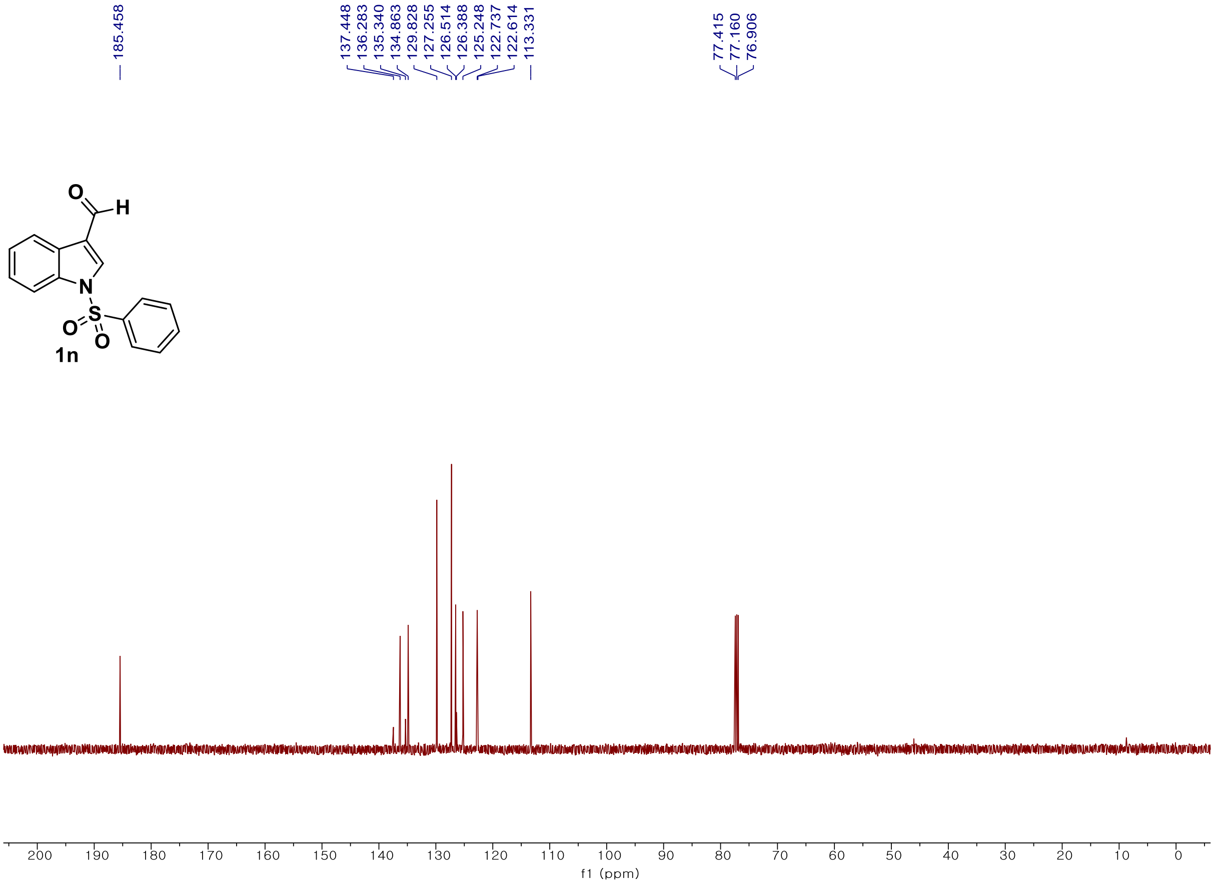
185.458
137.448
136.283
135.340
134.863
129.828
127.255
126.514
126.388
125.248
122.737
122.614
113.331
77.415
77.160
76.906
O
H
N
S
O
O
1n
200 190 180 170 160 150 140 130 120 110 100 90 80 70 60 50 40 30 20 10 0
f1 (ppm)

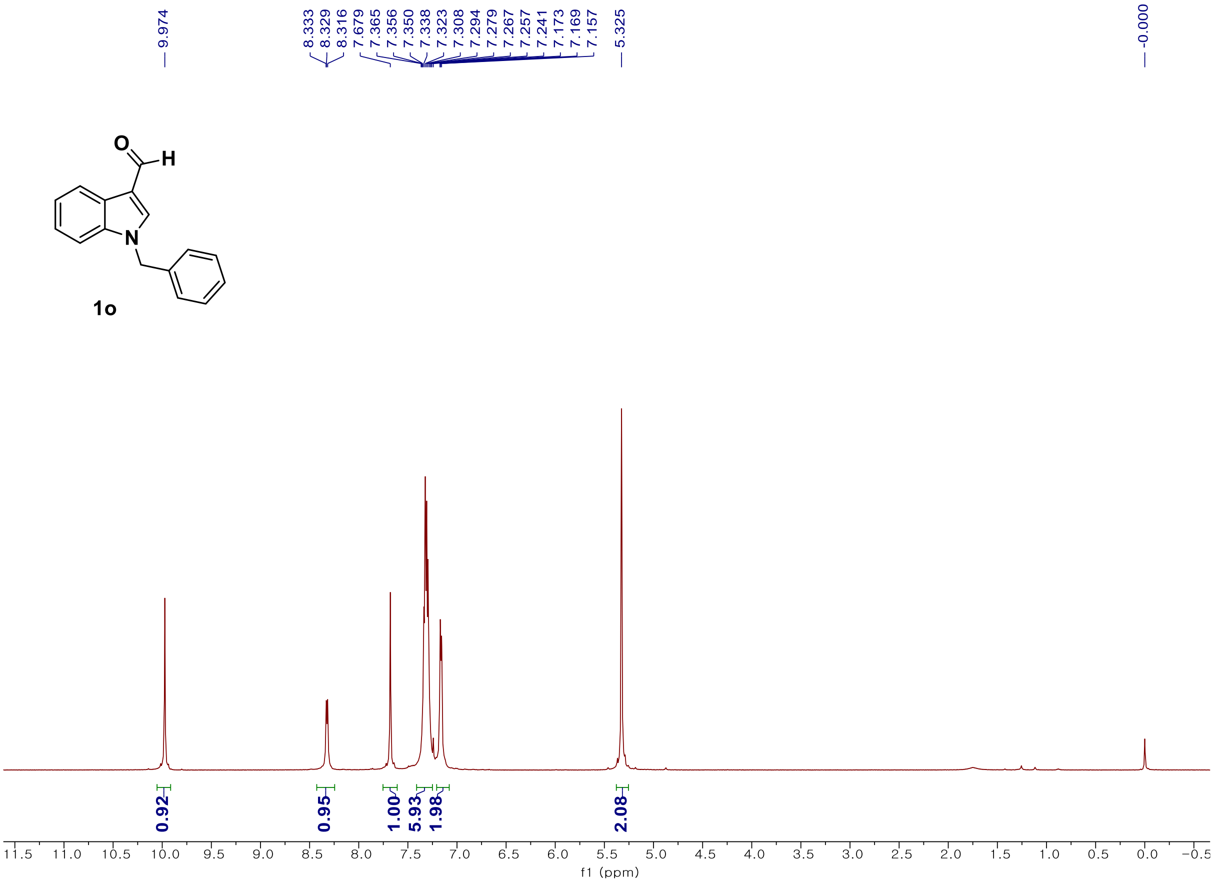
9.974
8.333
8.329
8.316
7.679
7.365
7.356
7.350
7.338
7.323
7.308
7.294
7.279
7.267
7.257
7.241
7.173
7.169
7.157
5.325
-0.000
1o
0.92
0.95
1.00
5.93
1.98
2.08
f1 (ppm)

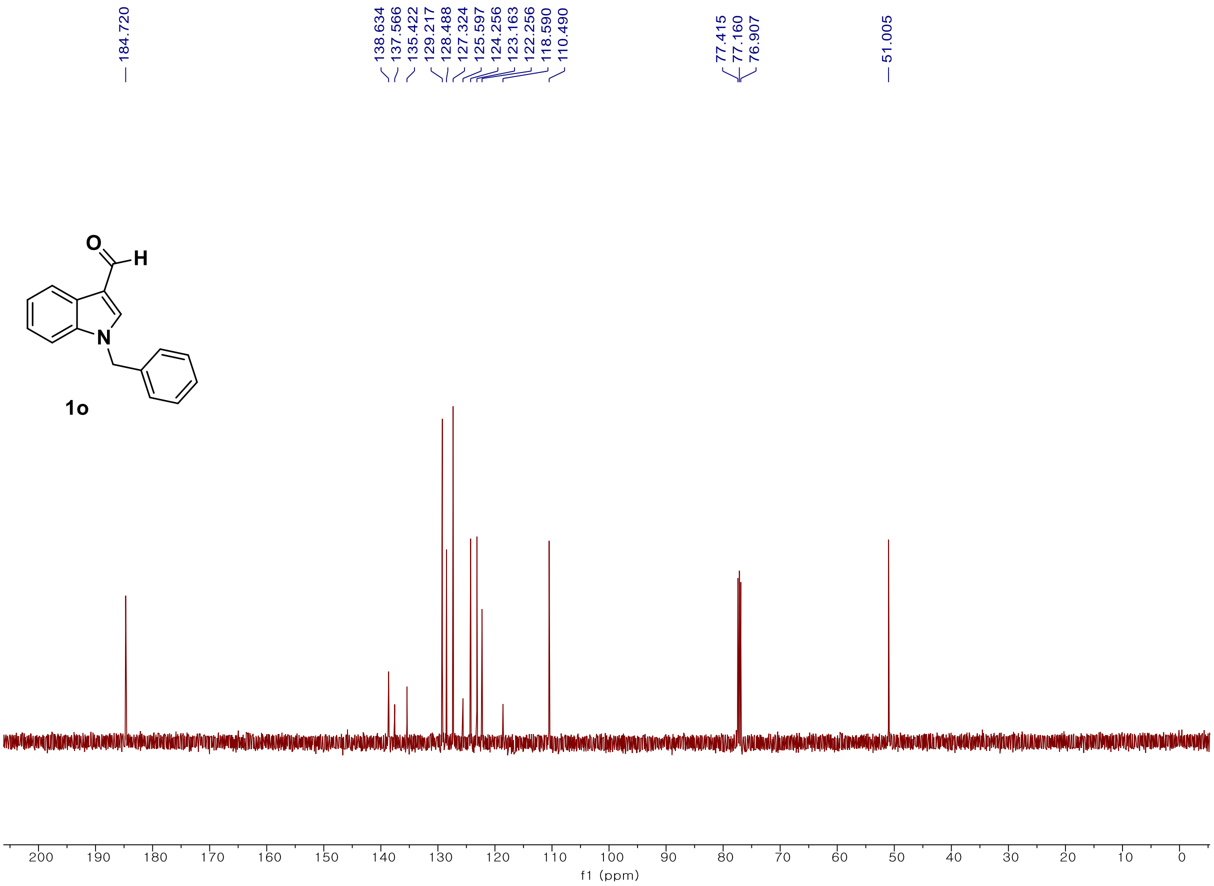
184.720
138.634
137.566
135.422
129.217
128.488
127.324
125.597
124.256
123.163
122.256
118.590
110.490
77.415
77.160
76.907
51.005
1o
f1 (ppm)

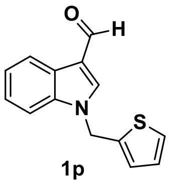

1p

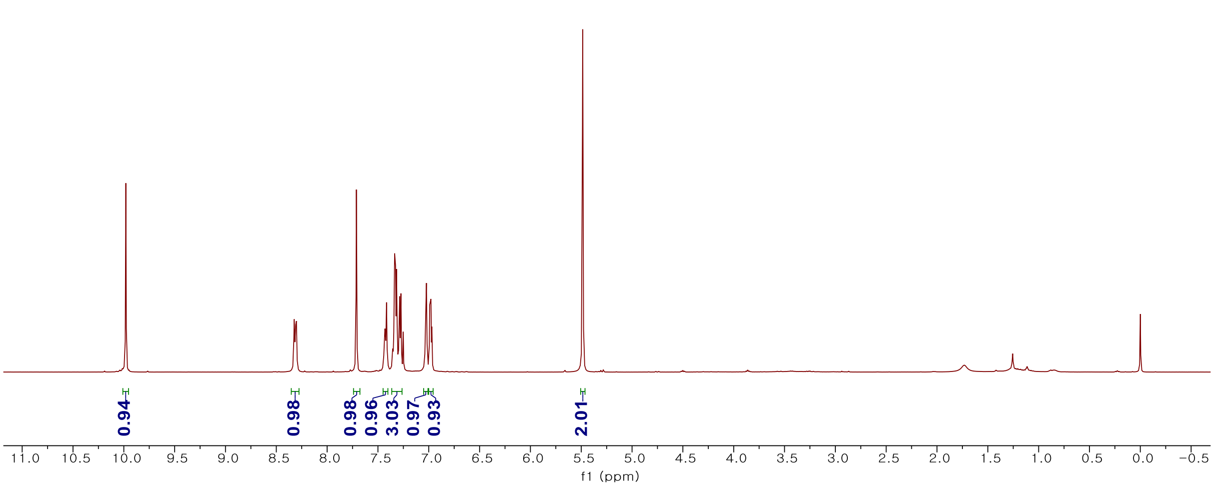
9.980
8.325 8.321 8.314 8.309 8.302 7.712 7.438 7.432 7.426 7.418 7.415 7.407 7.354 7.341 7.336 7.327 7.317 7.313 7.300 7.296 7.289 7.285 7.276 7.273 7.251 7.032 7.023 6.991 6.983 6.978 6.970 5.486
-0.000
0.94 0.98 0.98 0.96 3.03 0.97 0.93 2.01
11.0 10.5 10.0 9.5 9.0 8.5 8.0 7.5 7.0 6.5 6.0 5.5 5.0 4.5 4.0 3.5 3.0 2.5 2.0 1.5 1.0 0.5 0.0 -0.5
f1 (ppm)

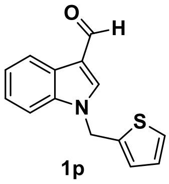

1p

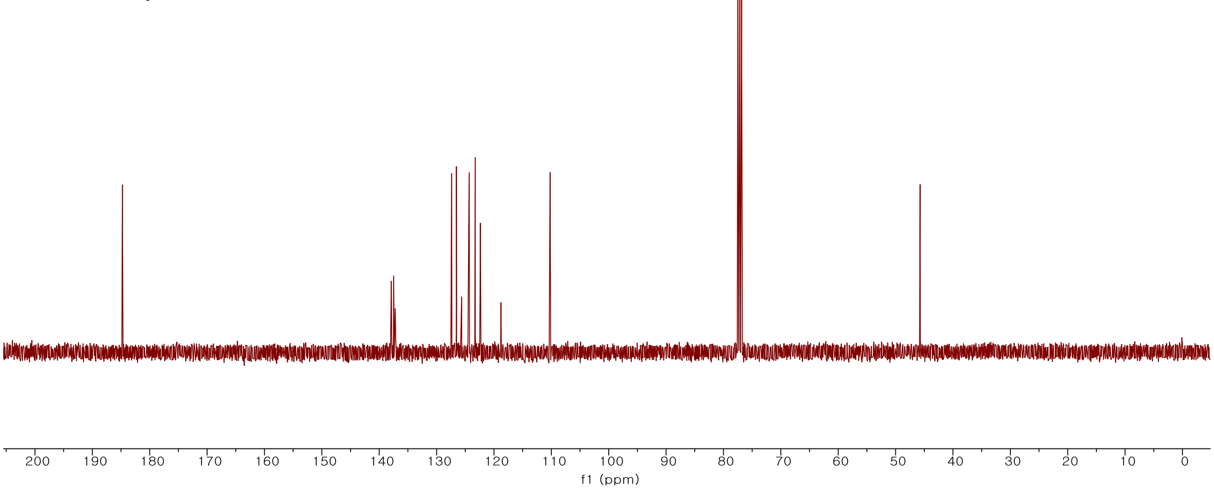
184.753
137.895 137.478 137.222 127.412 127.373 126.545 125.635 124.325 123.278 122.359 118.786 110.217
77.478 77.160 76.842
45.719
200 190 180 170 160 150 140 130 120 110 100 90 80 70 60 50 40 30 20 10 0
f1 (ppm)

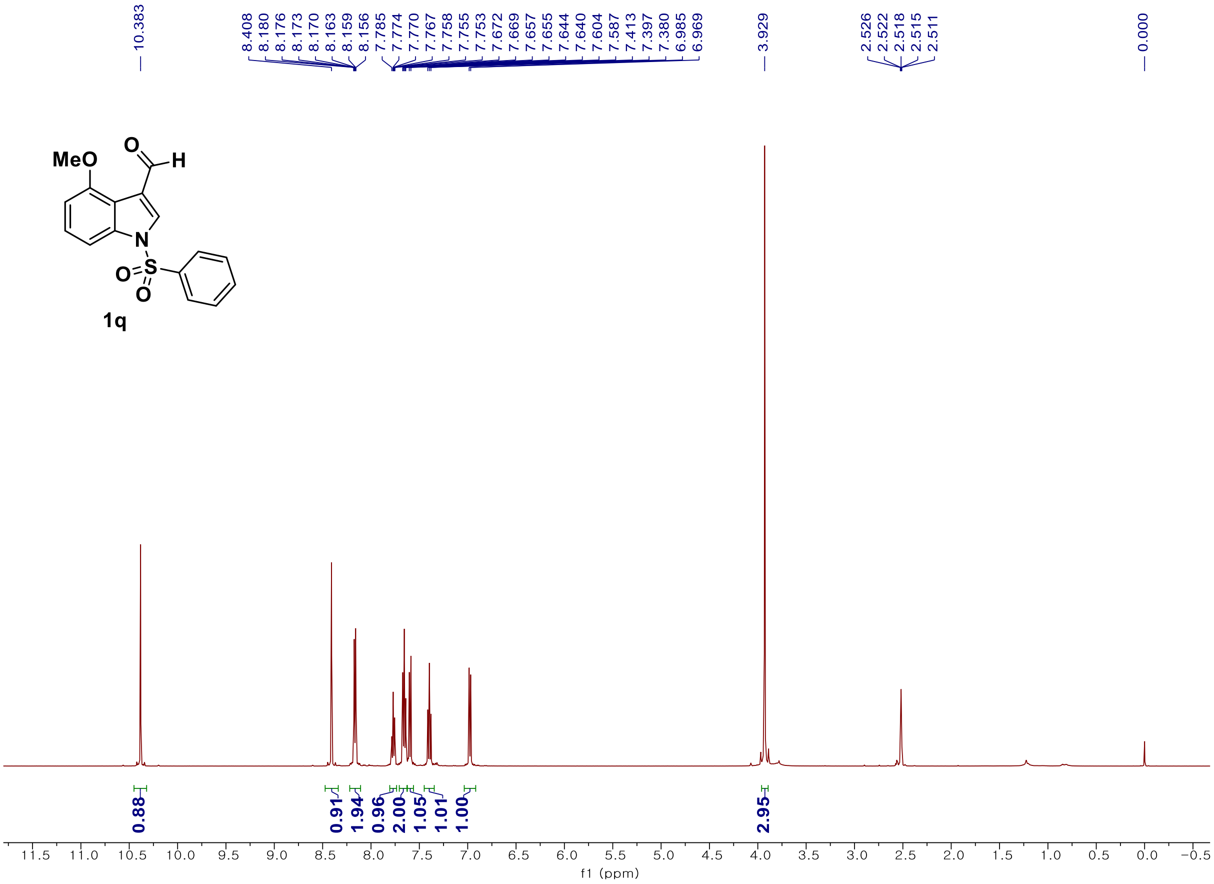
10.383
8.408
8.180
8.176
8.173
8.170
8.163
8.159
8.156
7.785
7.774
7.770
7.767
7.758
7.755
7.753
7.672
7.669
7.657
7.655
7.644
7.640
7.604
7.587
7.413
7.397
7.380
6.985
6.969
3.929
2.526
2.522
2.518
2.515
2.511
0.000
MeO
O
H
N
O=S
O
1q
0.88
0.91
1.94
0.96
2.00
1.05
1.01
1.00
2.95
f1 (ppm)

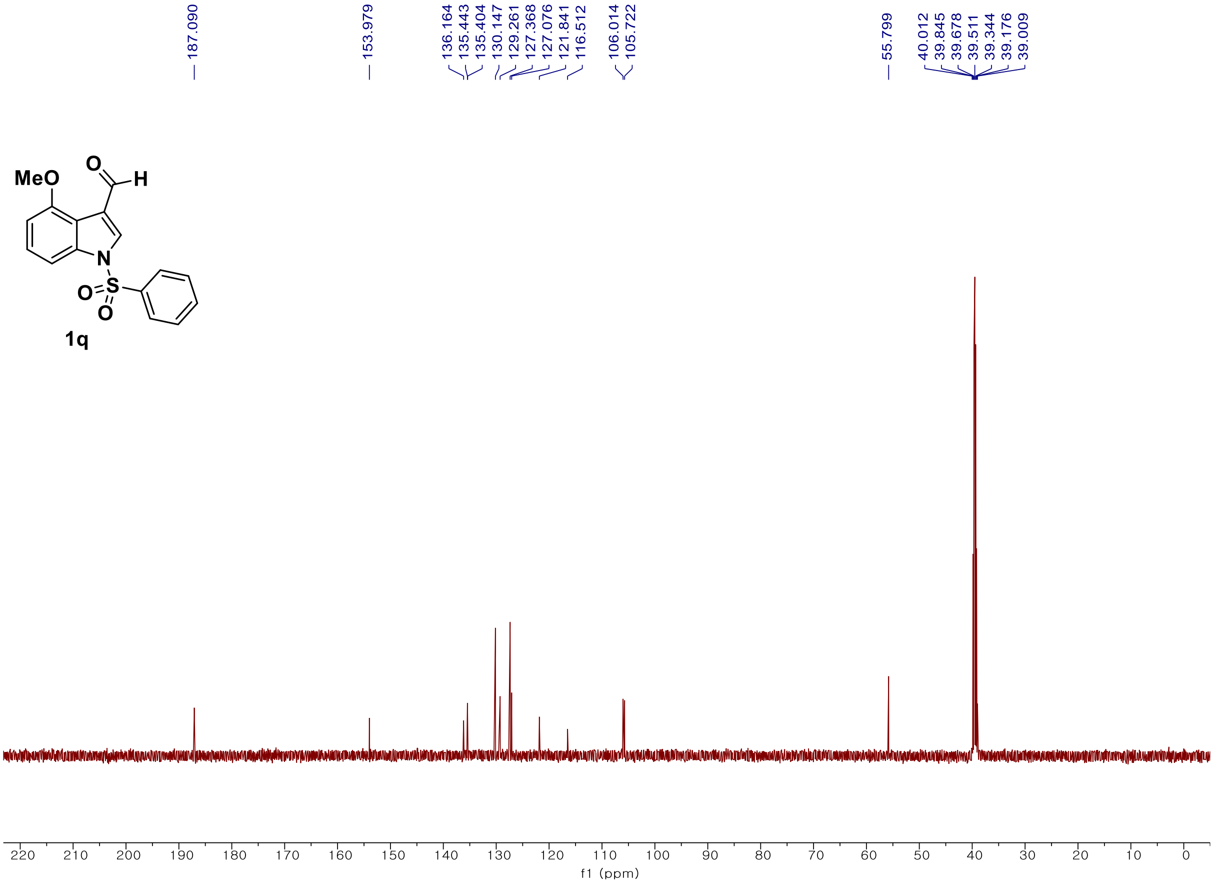
187.090
153.979
136.164
135.443
135.404
130.147
129.261
127.368
127.076
121.841
116.512
106.014
105.722
55.799
40.012
39.845
39.678
39.511
39.344
39.176
39.009
MeO
O
H
N
O=S
O
1q
f1 (ppm)

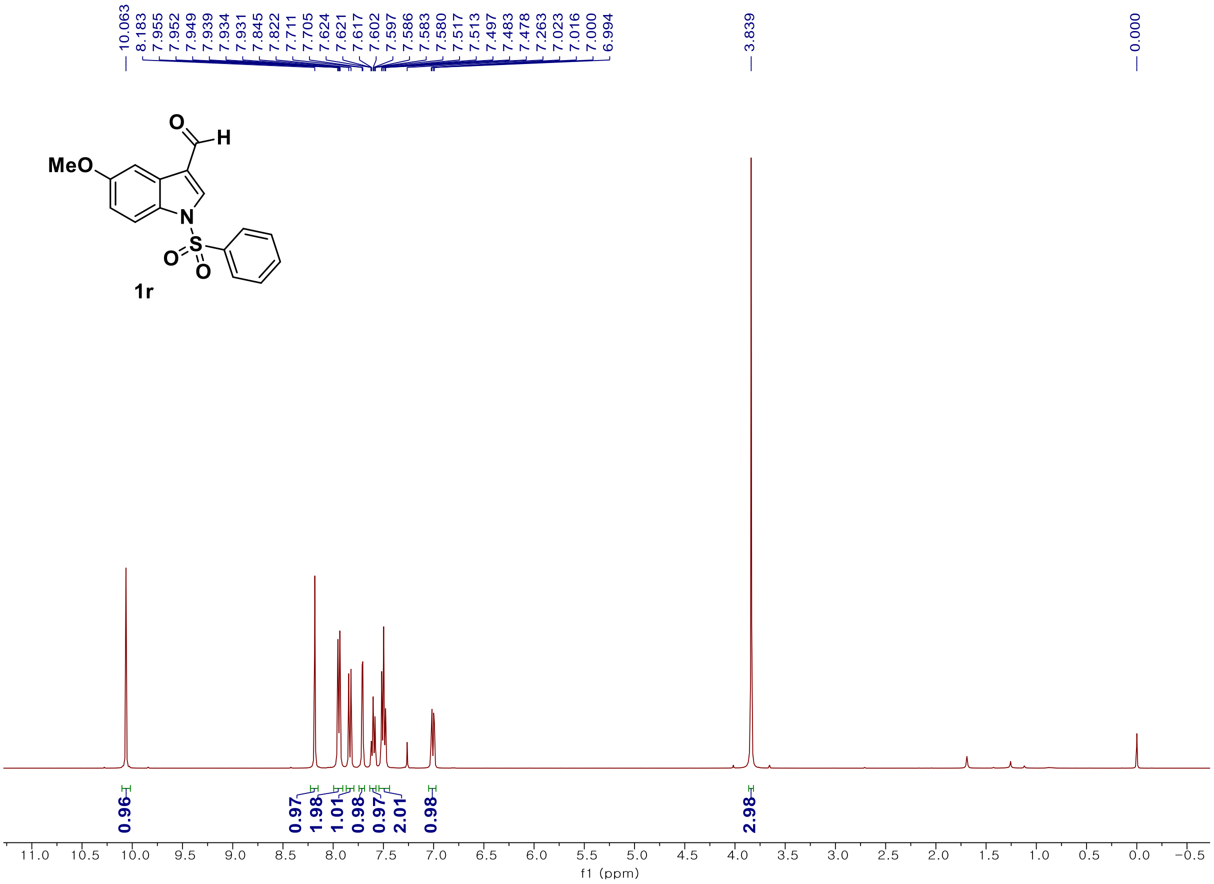
10.063
8.183
7.955
7.952
7.949
7.939
7.934
7.931
7.845
7.822
7.711
7.705
7.624
7.621
7.617
7.602
7.597
7.586
7.583
7.580
7.517
7.513
7.497
7.483
7.478
7.263
7.023
7.016
7.000
6.994
3.839
0.000
O
H
MeO
N
S
O
O
1r
0.96
0.97
1.98
1.01
0.98
0.97
2.01
0.98
2.98
f1 (ppm)

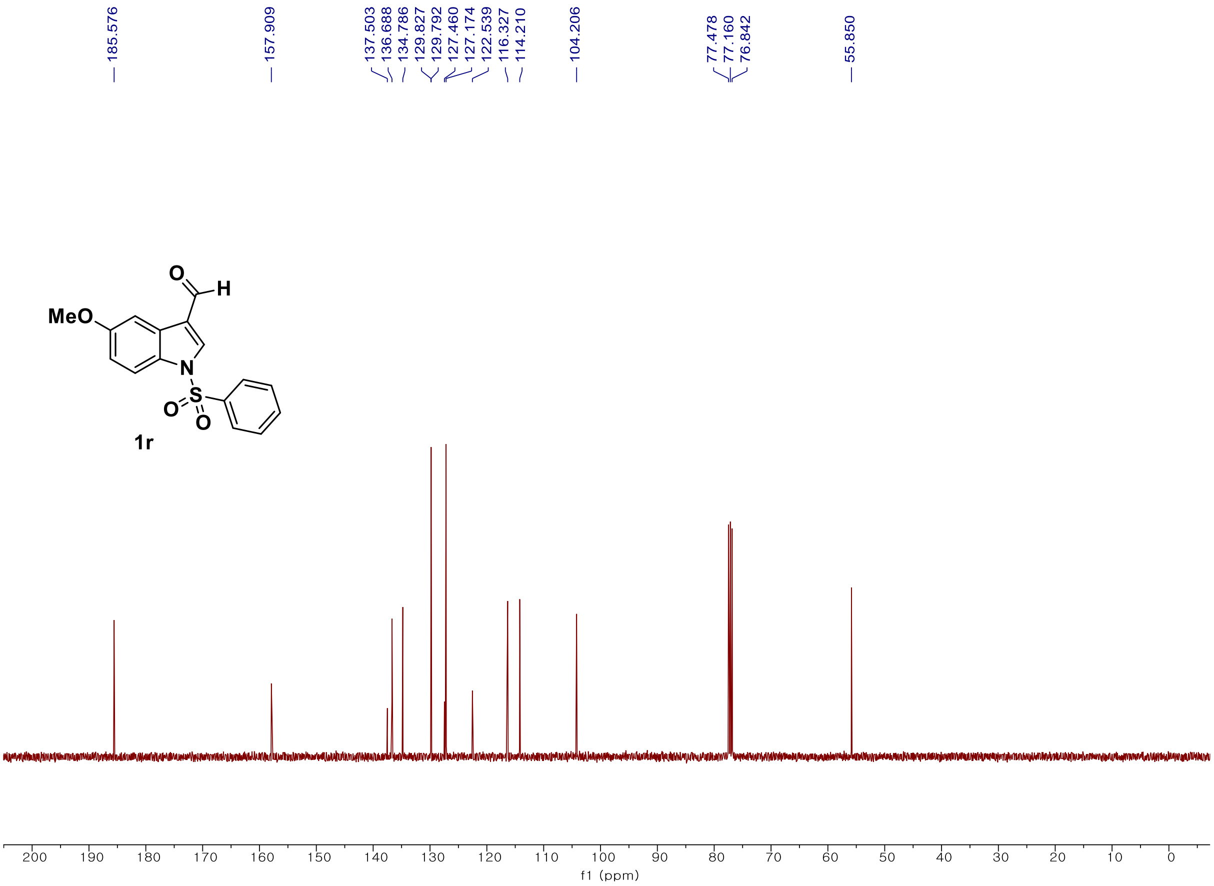
185.576
157.909
137.503
136.688
134.786
129.827
129.792
127.460
127.174
122.539
116.327
114.210
104.206
77.478
77.160
76.842
55.850
O
H
MeO
N
S
O
O
1r
f1 (ppm)

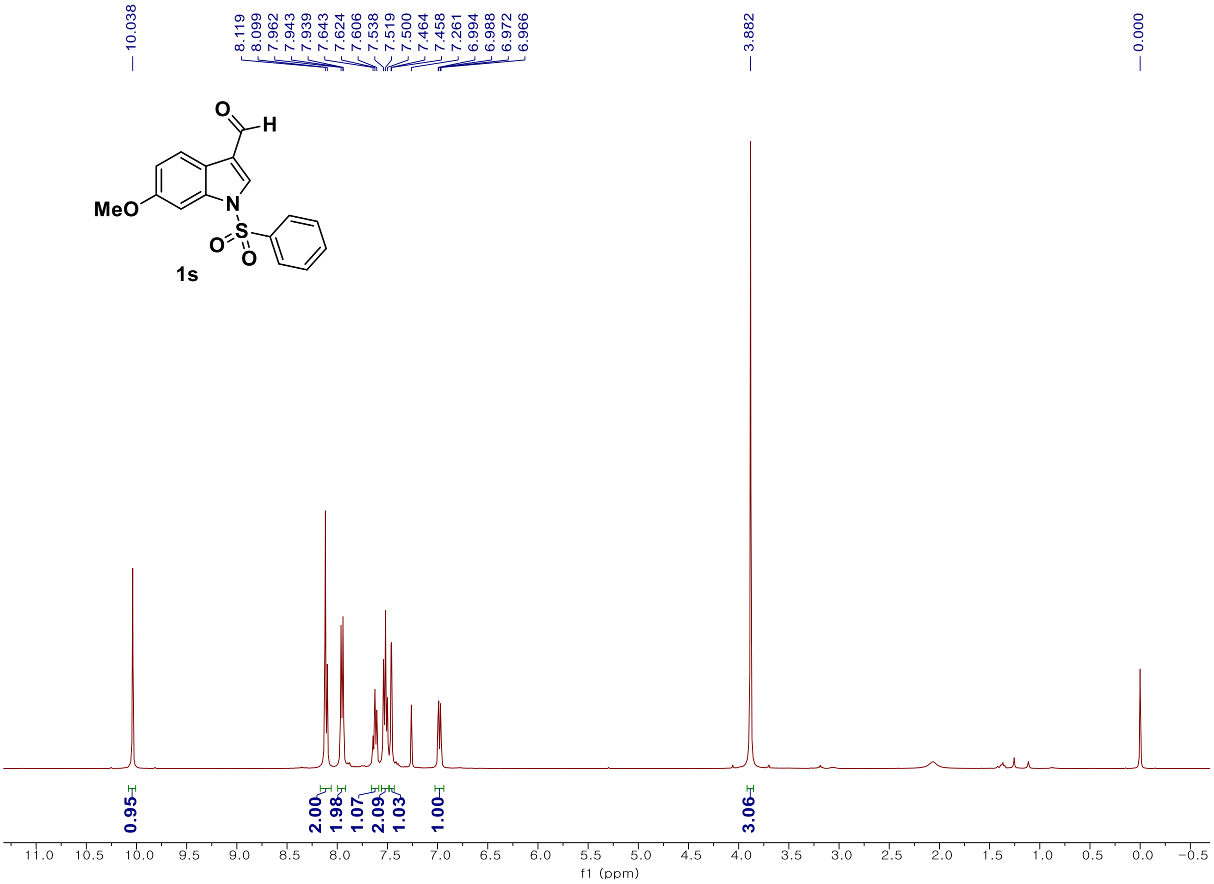
10.038
8.119
8.099
7.962
7.943
7.939
7.643
7.624
7.606
7.538
7.519
7.500
7.464
7.458
7.261
6.994
6.988
6.972
6.966
3.882
0.000
MeO
O
H
N
S
O
O
1s
0.95
2.00
1.98
1.07
2.09
1.03
1.00
3.06
f1 (ppm)

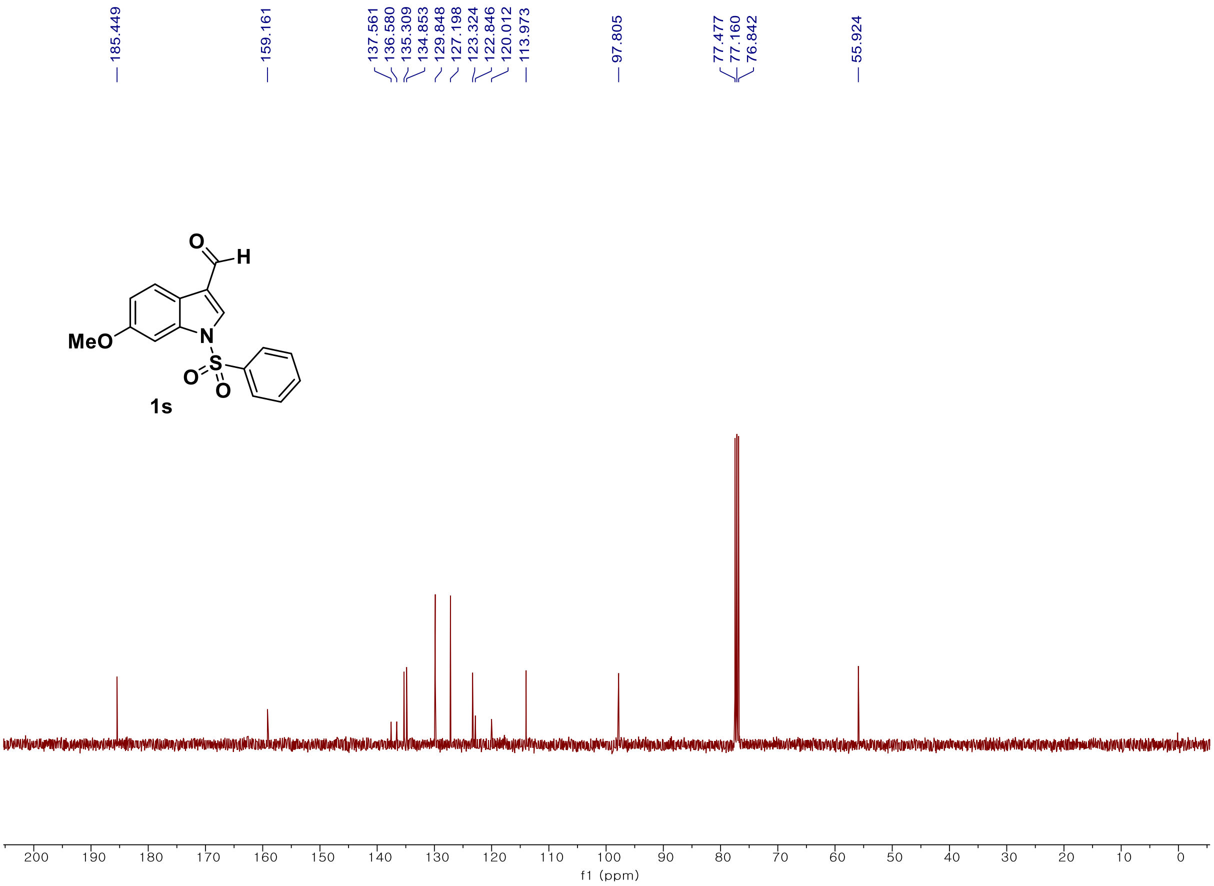
185.449
159.161
137.561
136.580
135.309
134.853
129.848
127.198
123.324
122.846
120.012
113.973
97.805
77.477
77.160
76.842
55.924
MeO
O
H
N
S
O
O
1s
f1 (ppm)

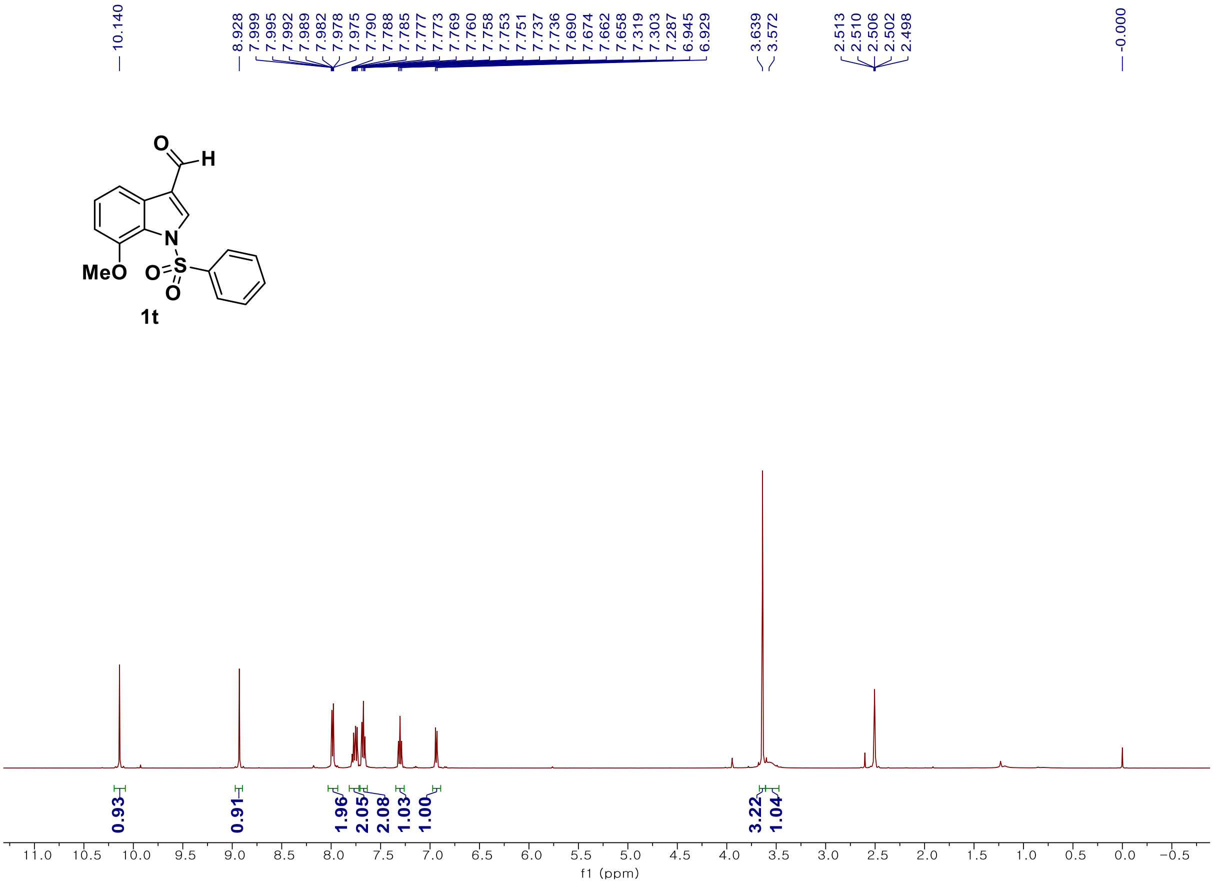
10.140
8.928
7.999
7.995
7.992
7.989
7.982
7.978
7.975
7.790
7.788
7.785
7.777
7.773
7.769
7.760
7.758
7.753
7.751
7.737
7.736
7.690
7.674
7.662
7.658
7.319
7.303
7.287
6.945
6.929
3.639
3.572
2.513
2.510
2.506
2.502
2.498
-0.000
MeO
1t
0.93
0.91
1.96
2.05
2.08
1.03
1.00
3.22
1.04
f1 (ppm)

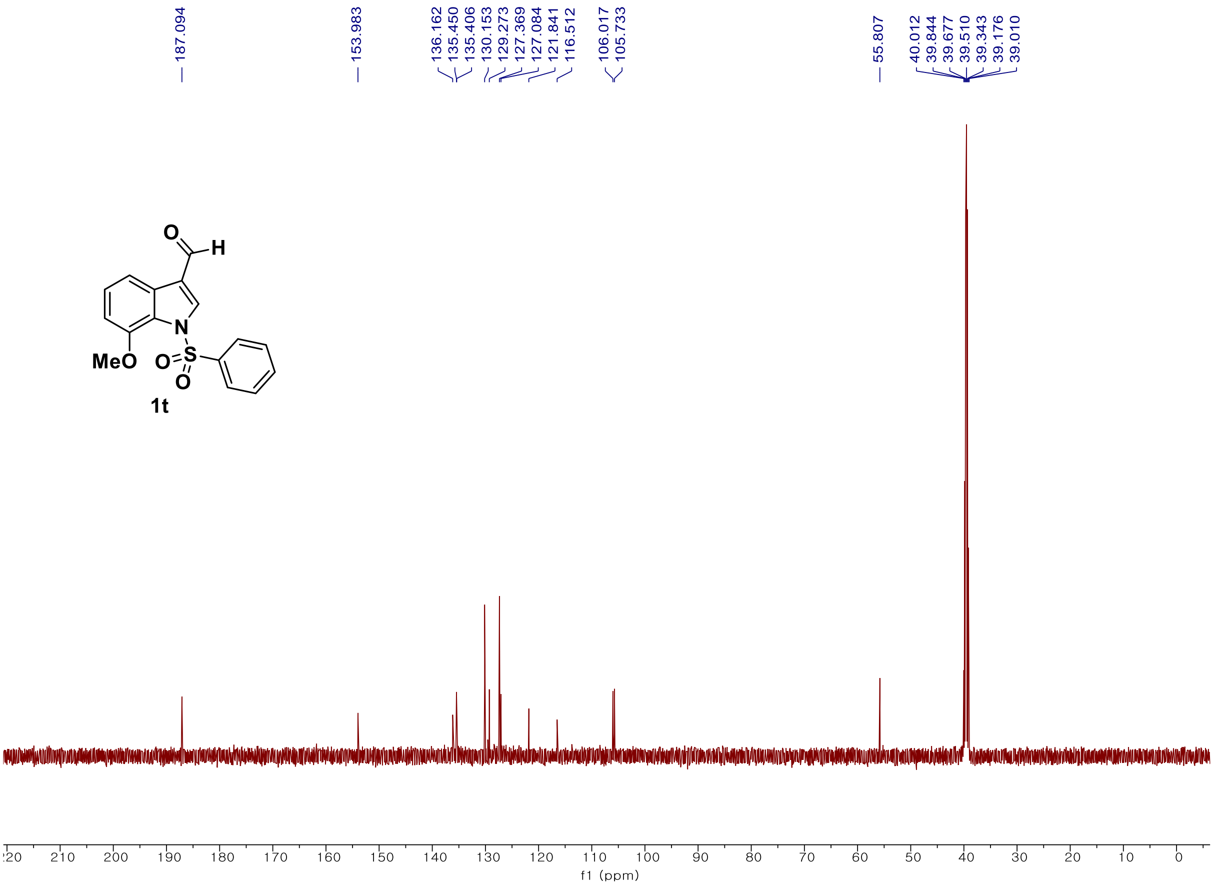
187.094
153.983
136.162
135.450
135.406
130.153
129.273
127.369
127.084
121.841
116.512
106.017
105.733
55.807
40.012
39.844
39.677
39.510
39.343
39.176
39.010
MeO
1t
f1 (ppm)

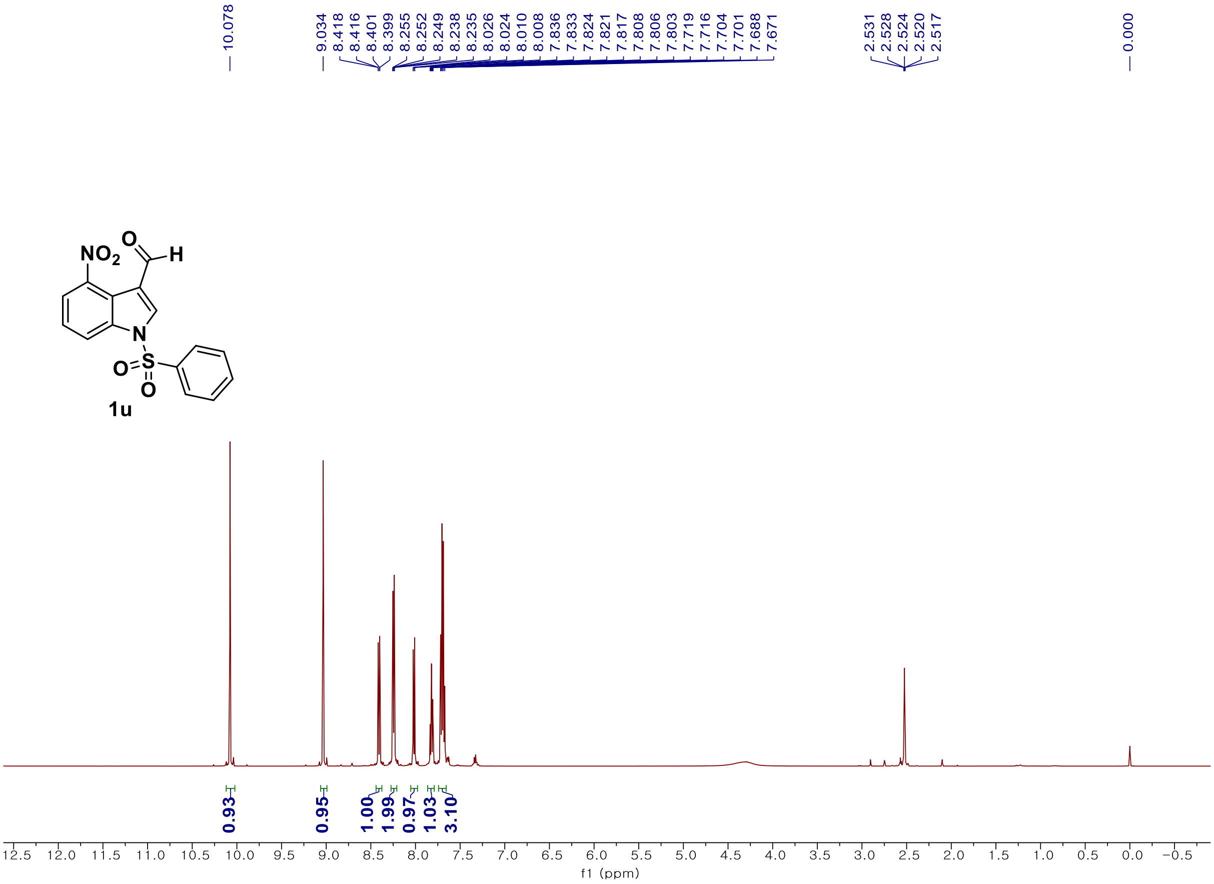
10.078
9.034
8.418
8.416
8.401
8.399
8.255
8.252
8.249
8.238
8.235
8.026
8.024
8.010
8.008
7.836
7.833
7.824
7.821
7.817
7.808
7.806
7.803
7.719
7.716
7.704
7.701
7.688
7.671
2.531
2.528
2.524
2.520
2.517
0.000
NO2
O
H
N
O=S
O
1u
0.93
0.95
1.00
1.99
0.97
1.03
3.10
f1 (ppm)

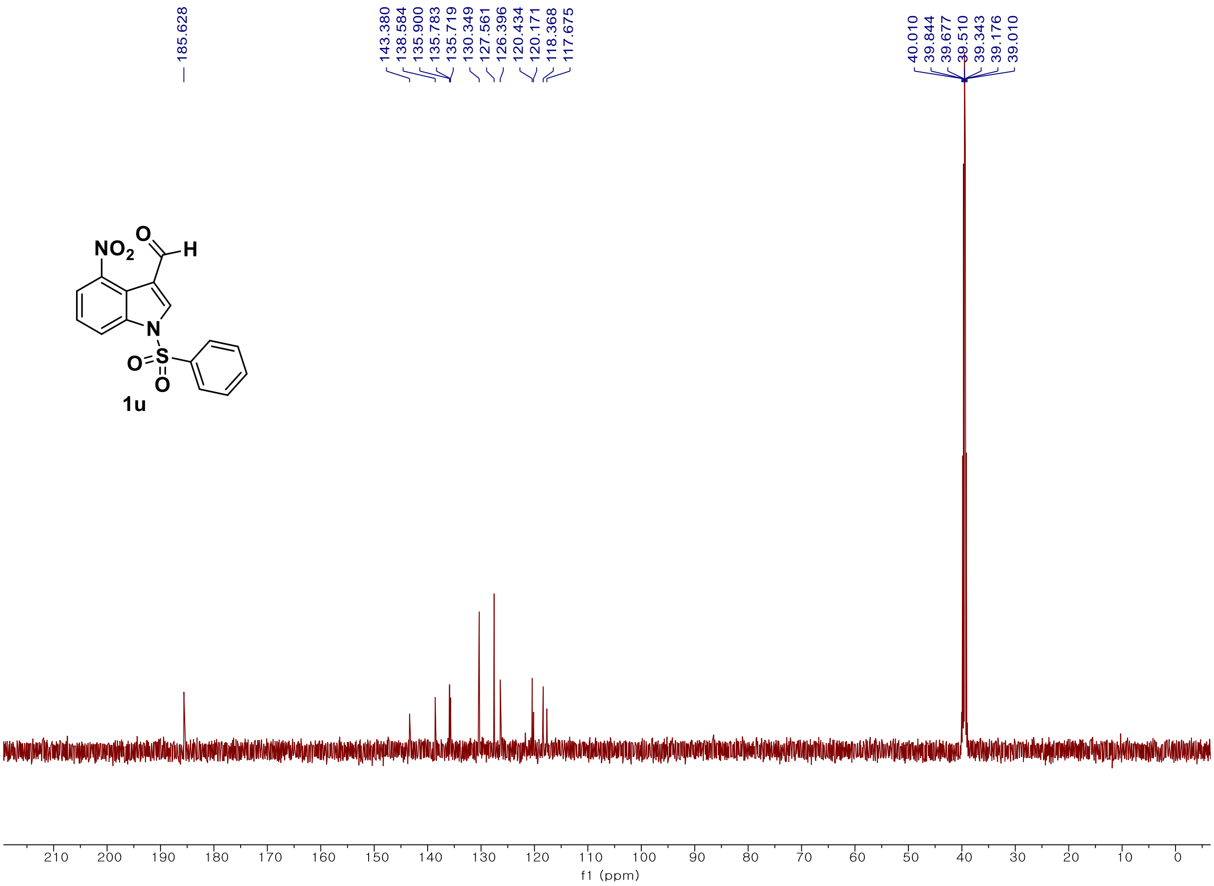
185.628
143.380
138.584
135.900
135.783
135.719
130.349
127.561
126.396
120.434
120.171
118.368
117.675
40.010
39.844
39.677
39.510
39.343
39.176
39.010
NO2
O
H
N
O=S
O
1u
f1 (ppm)

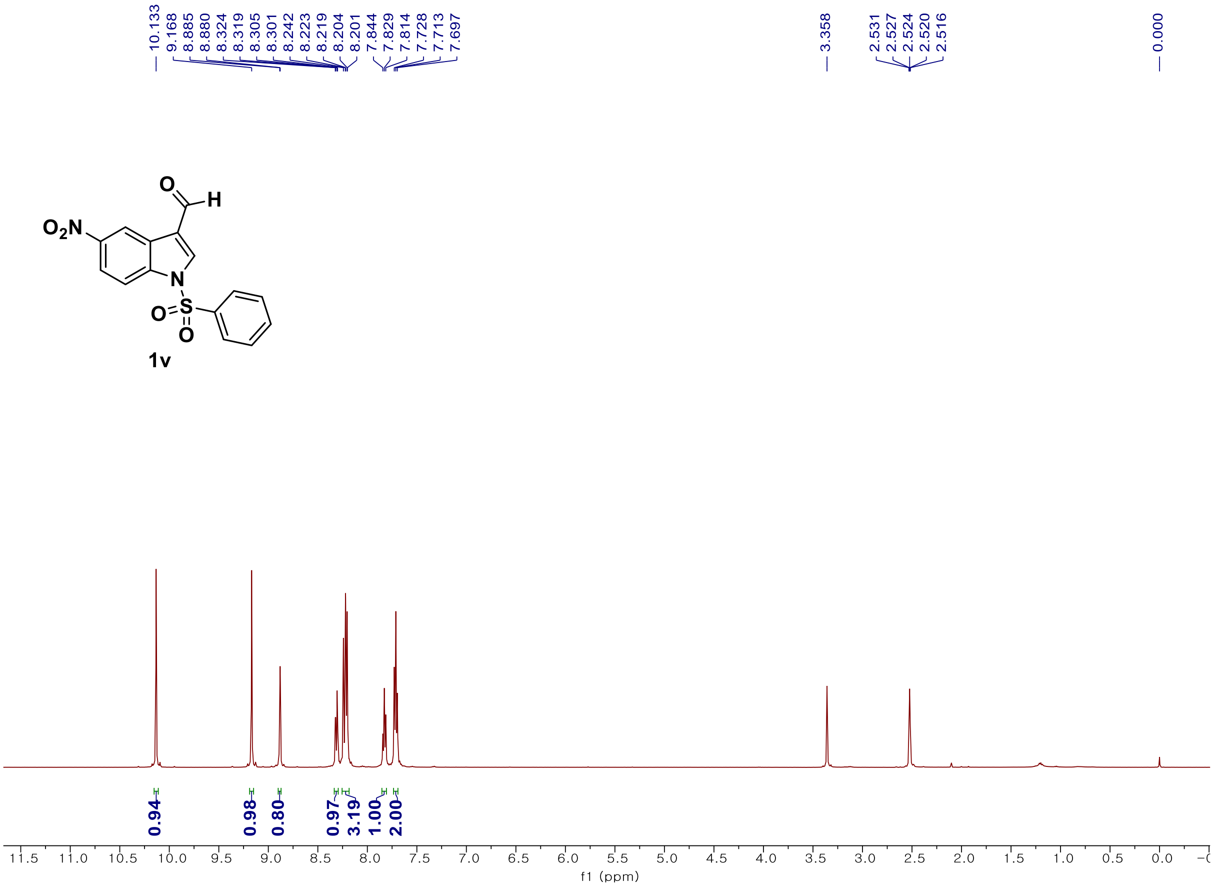
10.133
9.168
8.885
8.880
8.324
8.319
8.305
8.301
8.242
8.223
8.219
8.204
8.201
7.844
7.829
7.814
7.728
7.713
7.697
3.358
2.531
2.527
2.524
2.520
2.516
0.000
O_2N
O
H
N
S
O
O
1v
0.94
0.98
0.80
0.97
3.19
1.00
2.00
11.5 11.0 10.5 10.0 9.5 9.0 8.5 8.0 7.5 7.0 6.5 6.0 5.5 5.0 4.5 4.0 3.5 3.0 2.5 2.0 1.5 1.0 0.5 0.0
f1 (ppm)

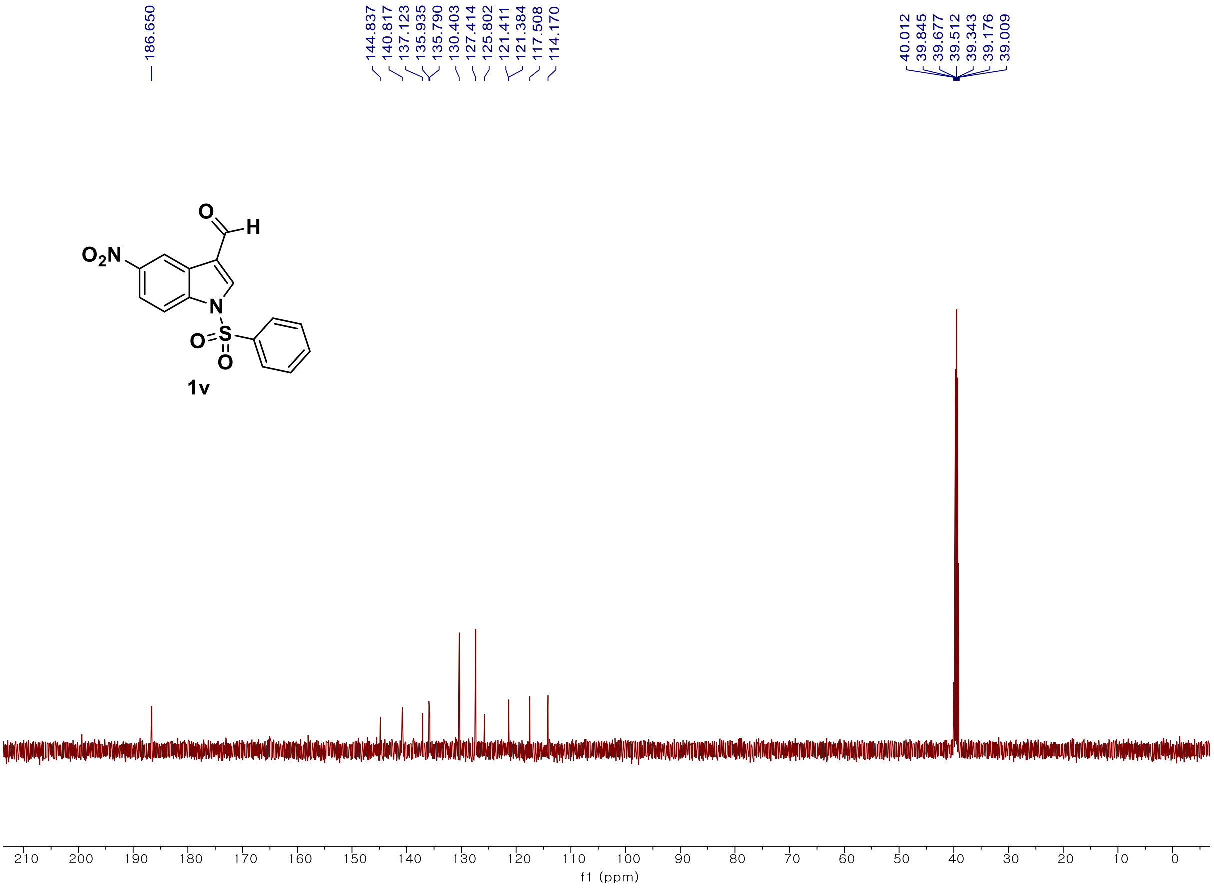
186.650
144.837
140.817
137.123
135.935
135.790
130.403
127.414
125.802
121.411
121.384
117.508
114.170
40.012
39.845
39.677
39.512
39.343
39.176
39.009
O_2N
O
H
N
S
O
O
1v
210 200 190 180 170 160 150 140 130 120 110 100 90 80 70 60 50 40 30 20 10 0
f1 (ppm)

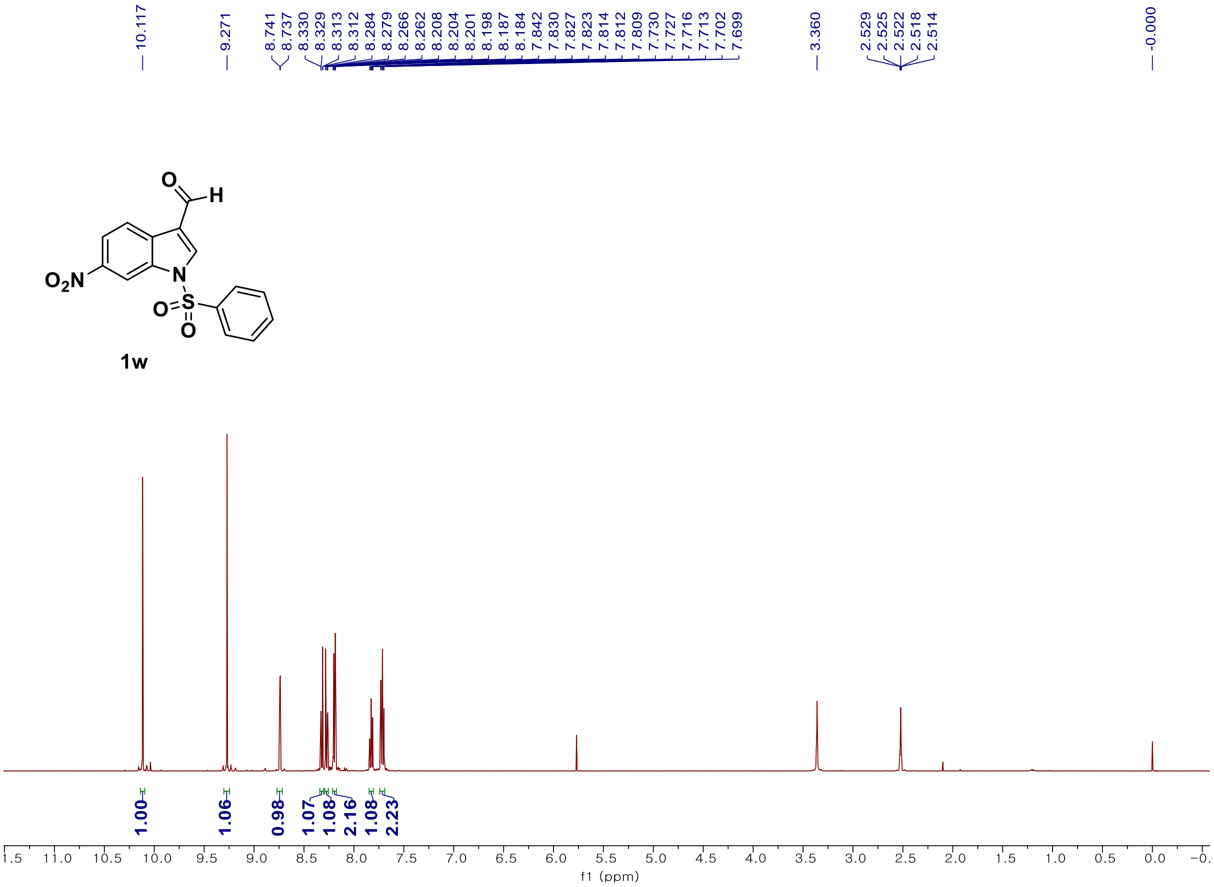
10.117
9.271
8.741
8.737
8.330
8.329
8.313
8.312
8.284
8.279
8.266
8.262
8.208
8.204
8.201
8.198
8.187
8.184
7.842
7.830
7.827
7.823
7.814
7.812
7.809
7.730
7.727
7.716
7.713
7.702
7.699
3.360
2.529
2.525
2.522
2.518
2.514
-0.000
O
H
O_2N
N
O=S
O
1w
1.00
1.06
0.98
1.07
1.08
2.16
1.08
2.23
f1 (ppm)

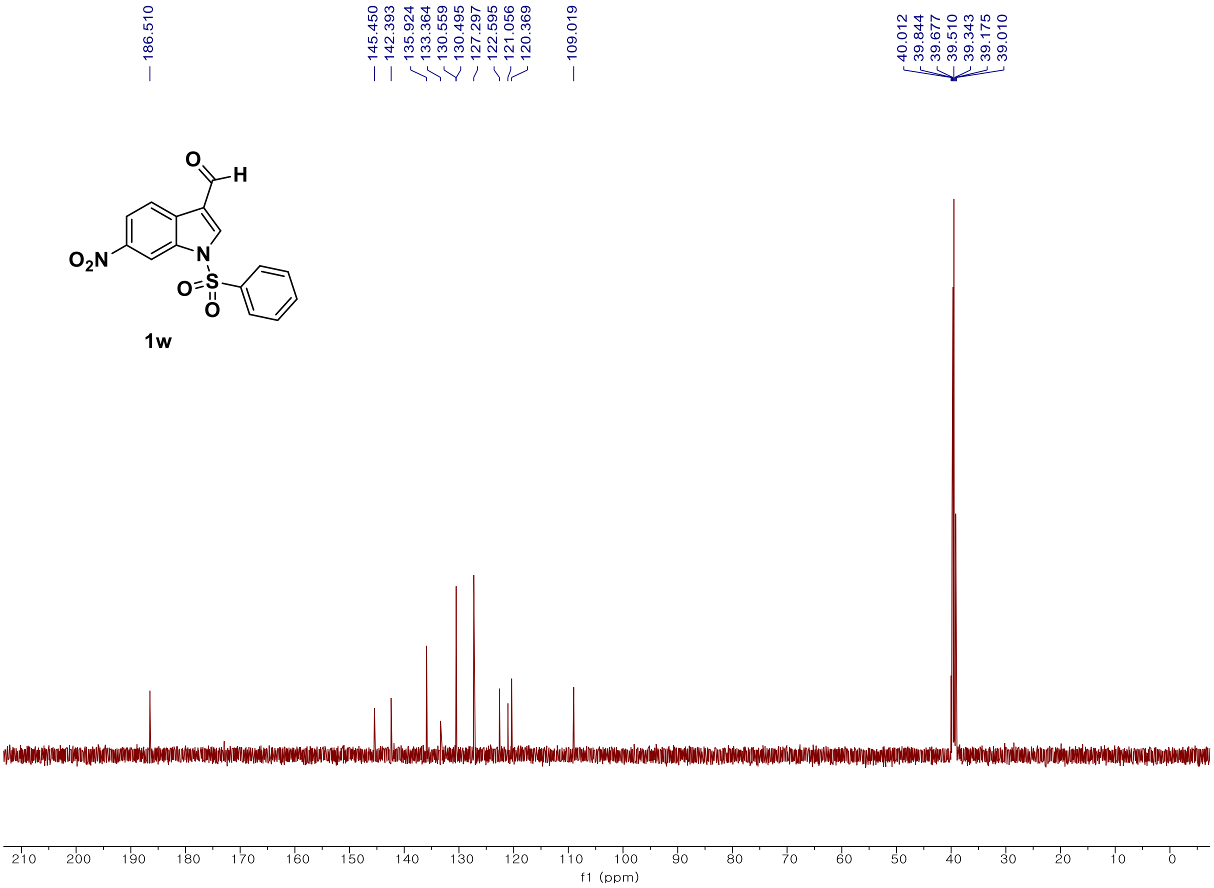
186.510
145.450
142.393
135.924
133.364
130.559
130.495
127.297
122.595
121.056
120.369
109.019
40.012
39.844
39.677
39.510
39.343
39.175
39.010
O
H
O_2N
N
O=S
O
1w
f1 (ppm)

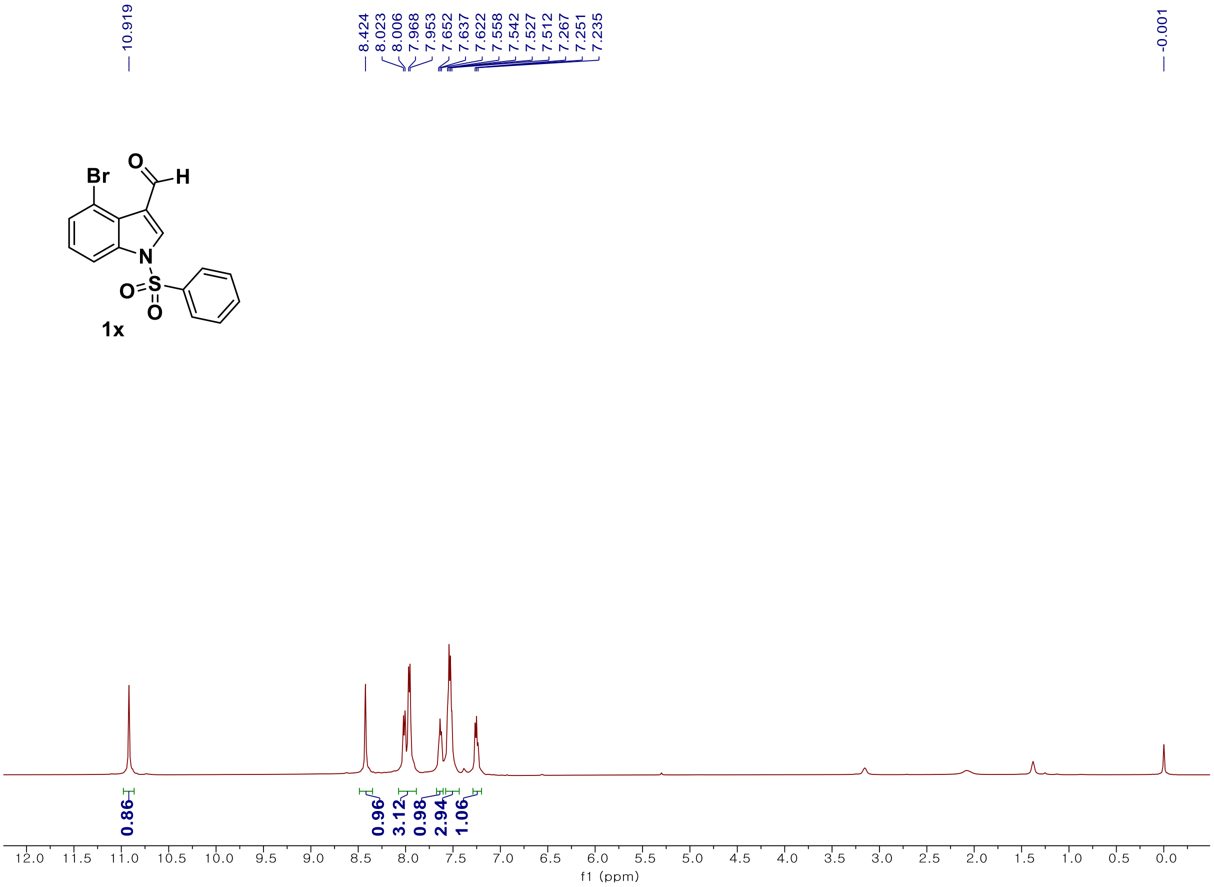
10.919
8.424
8.023
8.006
7.968
7.953
7.652
7.637
7.622
7.558
7.542
7.527
7.512
7.267
7.251
7.235
-0.001
Br
O
H
N
O=S
O
1x
0.86
0.96
3.12
0.98
2.94
1.06
12.0 11.5 11.0 10.5 10.0 9.5 9.0 8.5 8.0 7.5 7.0 6.5 6.0 5.5 5.0 4.5 4.0 3.5 3.0 2.5 2.0 1.5 1.0 0.5 0.0
f1 (ppm)

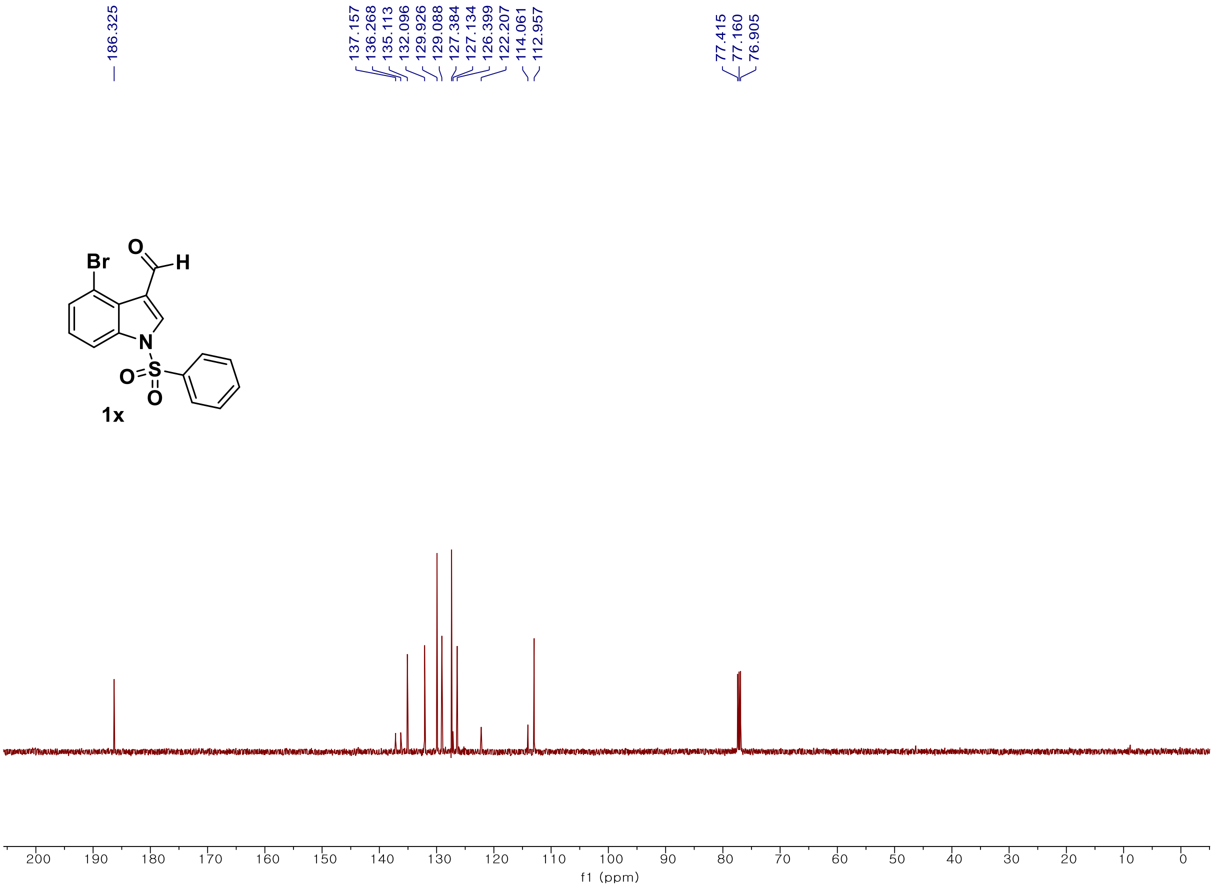
186.325
137.157
136.268
135.113
132.096
129.926
129.088
127.384
127.134
126.399
122.207
114.061
112.957
77.415
77.160
76.905
Br
O
H
N
O=S
O
1x
200 190 180 170 160 150 140 130 120 110 100 90 80 70 60 50 40 30 20 10 0
f1 (ppm)

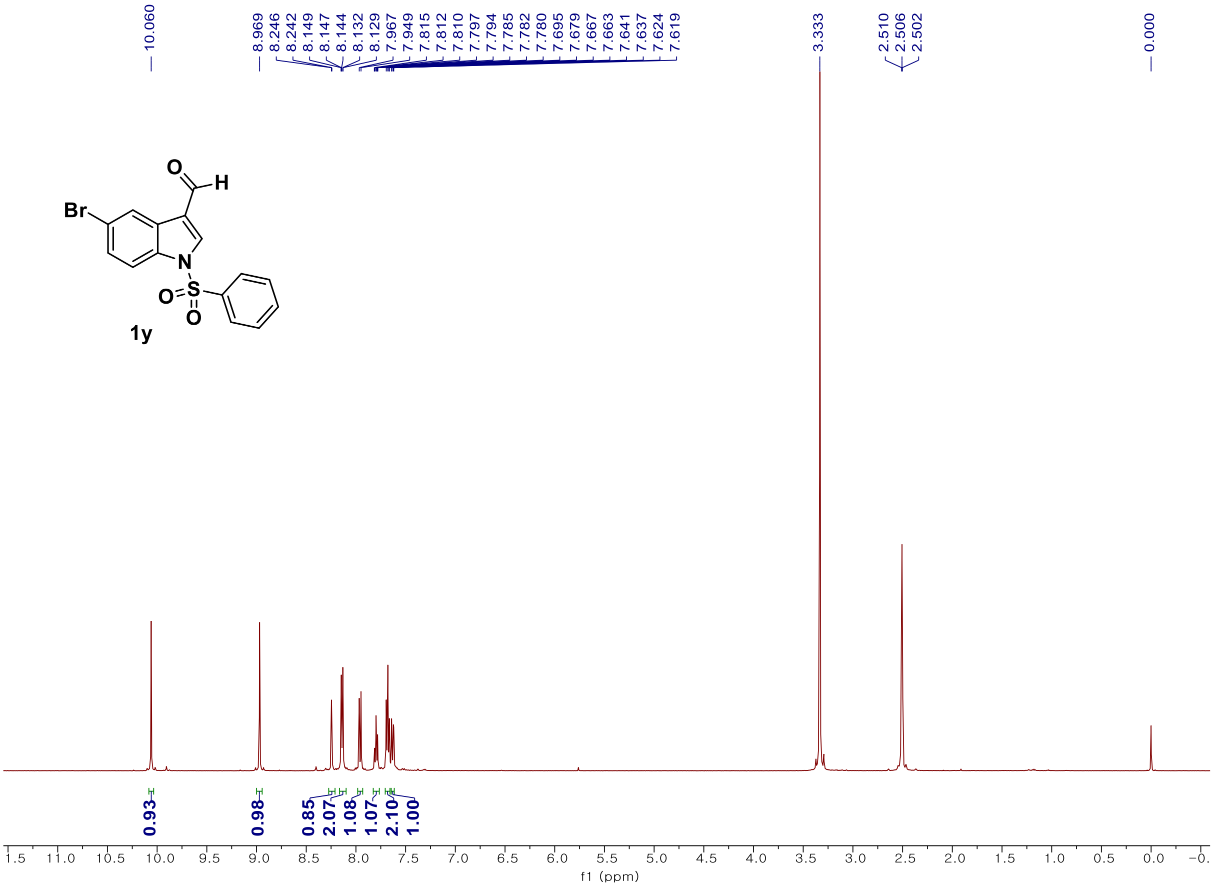
10.060
8.969
8.246
8.242
8.149
8.147
8.144
8.132
8.129
7.967
7.949
7.815
7.812
7.810
7.797
7.794
7.785
7.782
7.780
7.695
7.679
7.667
7.663
7.641
7.637
7.624
7.619
3.333
2.510
2.506
2.502
0.000
O
H
Br
N
S
O
O
1y
0.93
0.98
0.85
2.07
1.08
1.07
2.10
1.00
f1 (ppm)

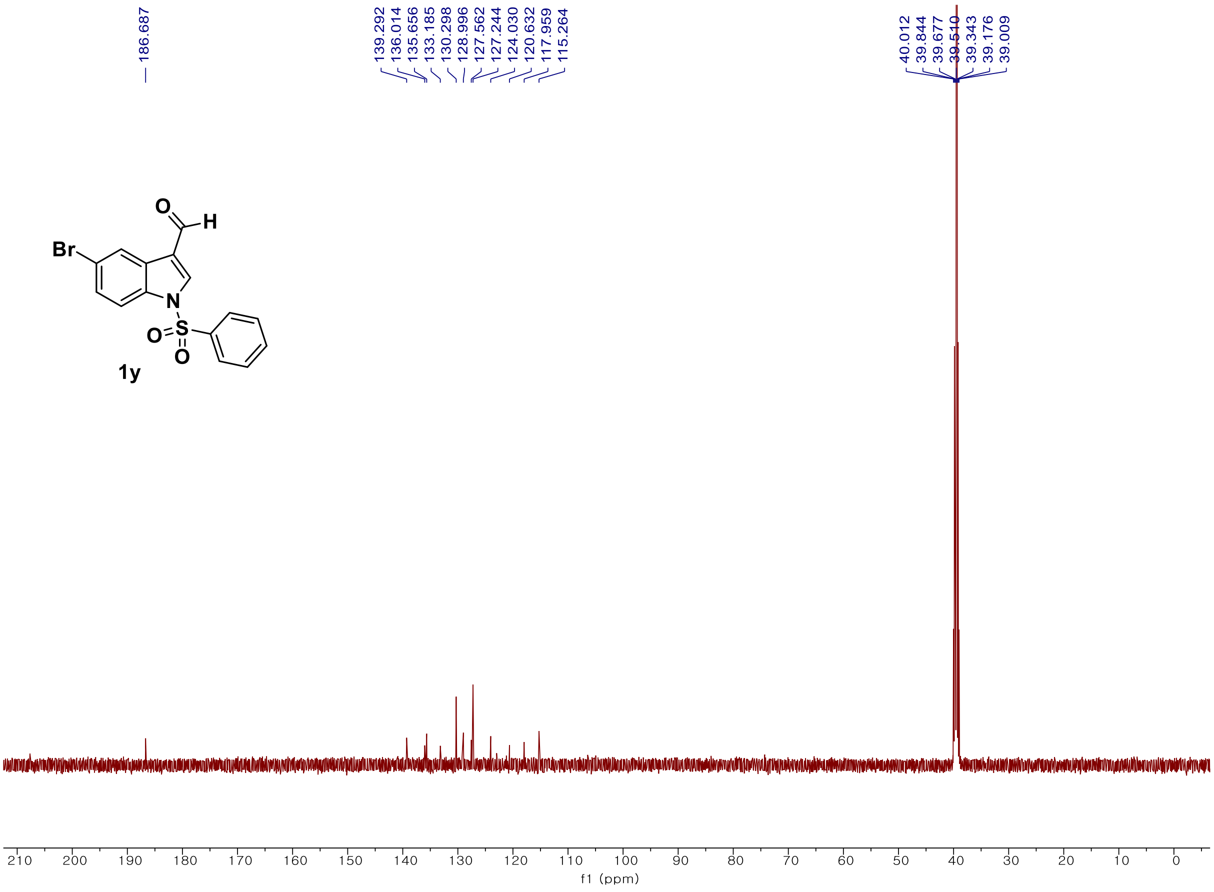
186.687
139.292
136.014
135.656
133.185
130.298
128.996
127.562
127.244
124.030
120.632
117.959
115.264
40.012
39.844
39.677
39.510
39.343
39.176
39.009
O
H
Br
N
S
O
O
1y
f1 (ppm)

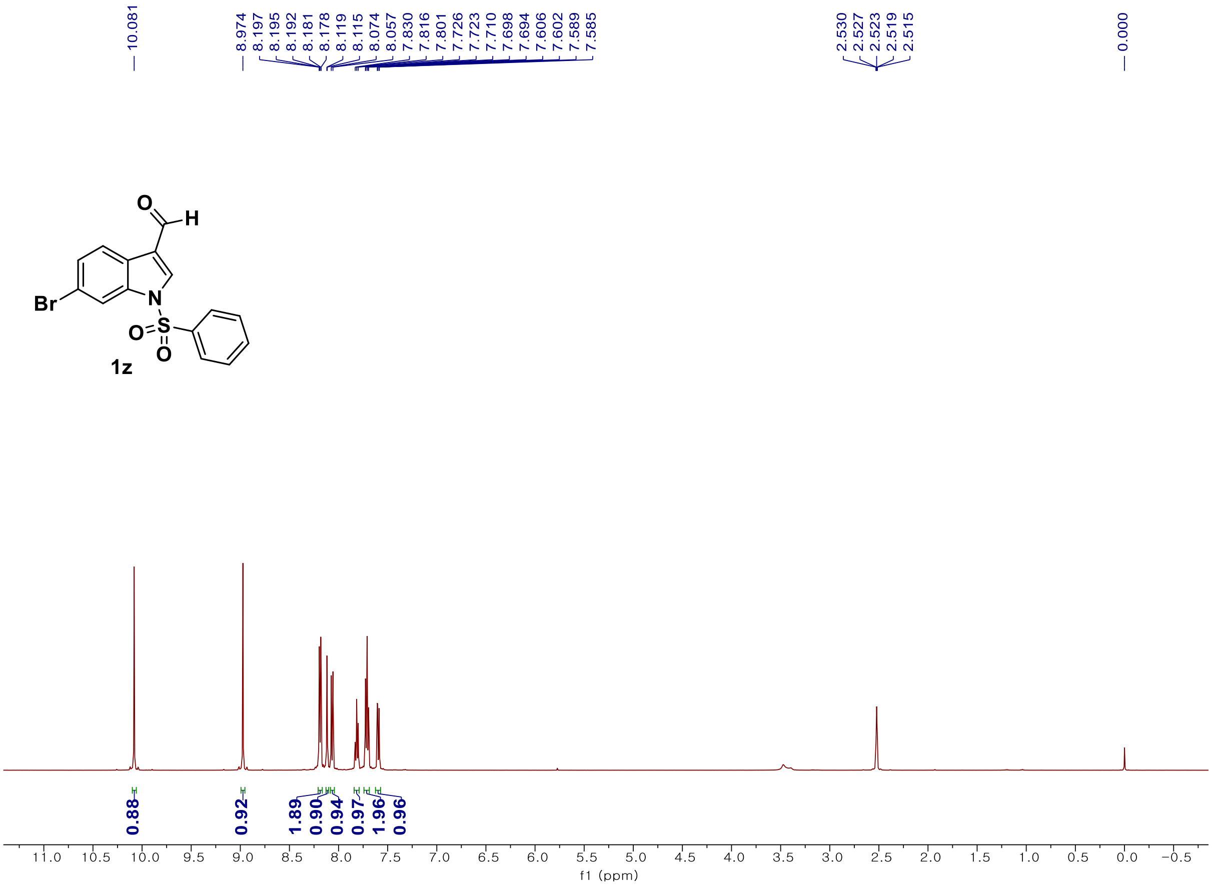
10.081
8.974
8.197
8.195
8.192
8.181
8.178
8.119
8.115
8.074
8.057
7.830
7.816
7.801
7.726
7.723
7.710
7.698
7.694
7.606
7.602
7.589
7.585
2.530
2.527
2.523
2.519
2.515
0.000
1z
0.88
0.92
1.89
0.90
0.94
0.97
1.96
0.96
f1 (ppm)

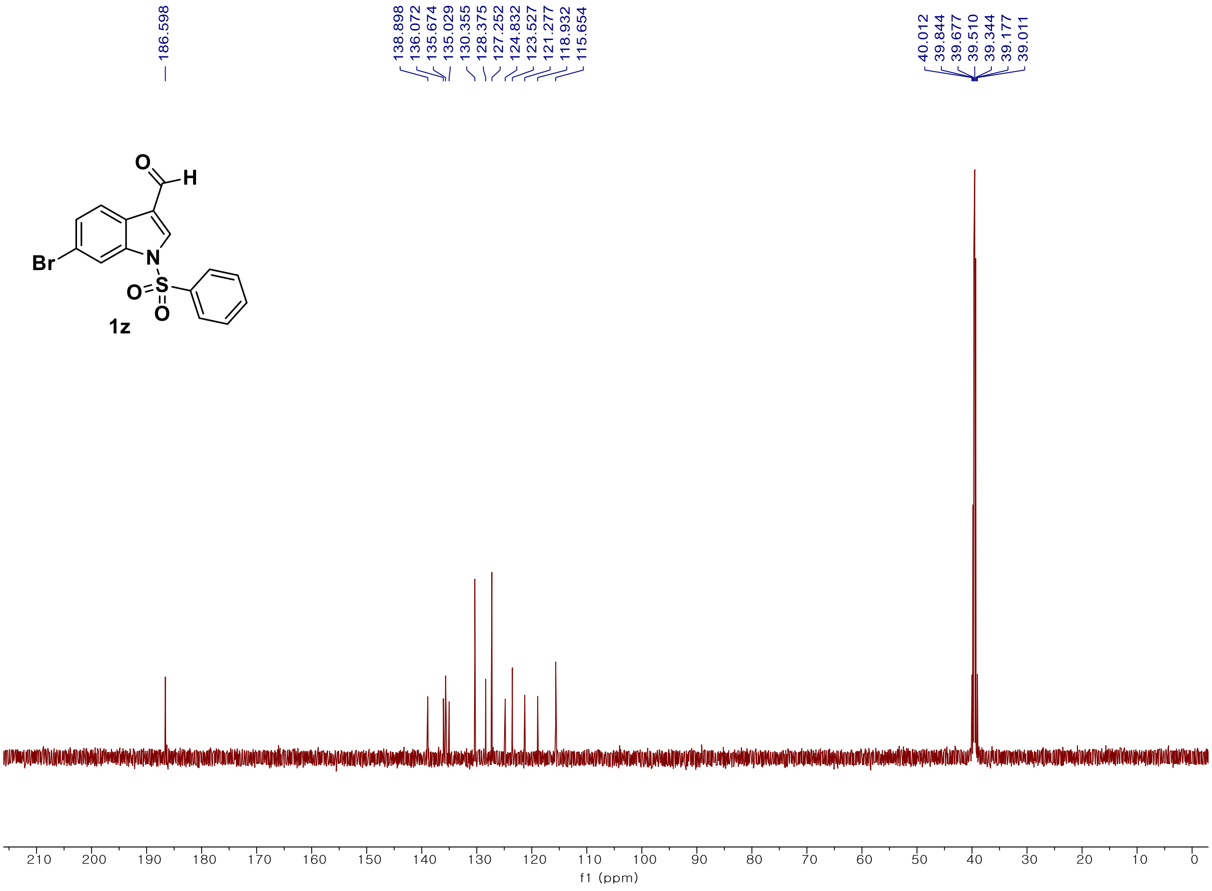
186.598
138.898
136.072
135.674
135.029
130.355
128.375
127.252
124.832
123.527
121.277
118.932
115.654
40.012
39.844
39.677
39.510
39.344
39.177
39.011
1z
f1 (ppm)

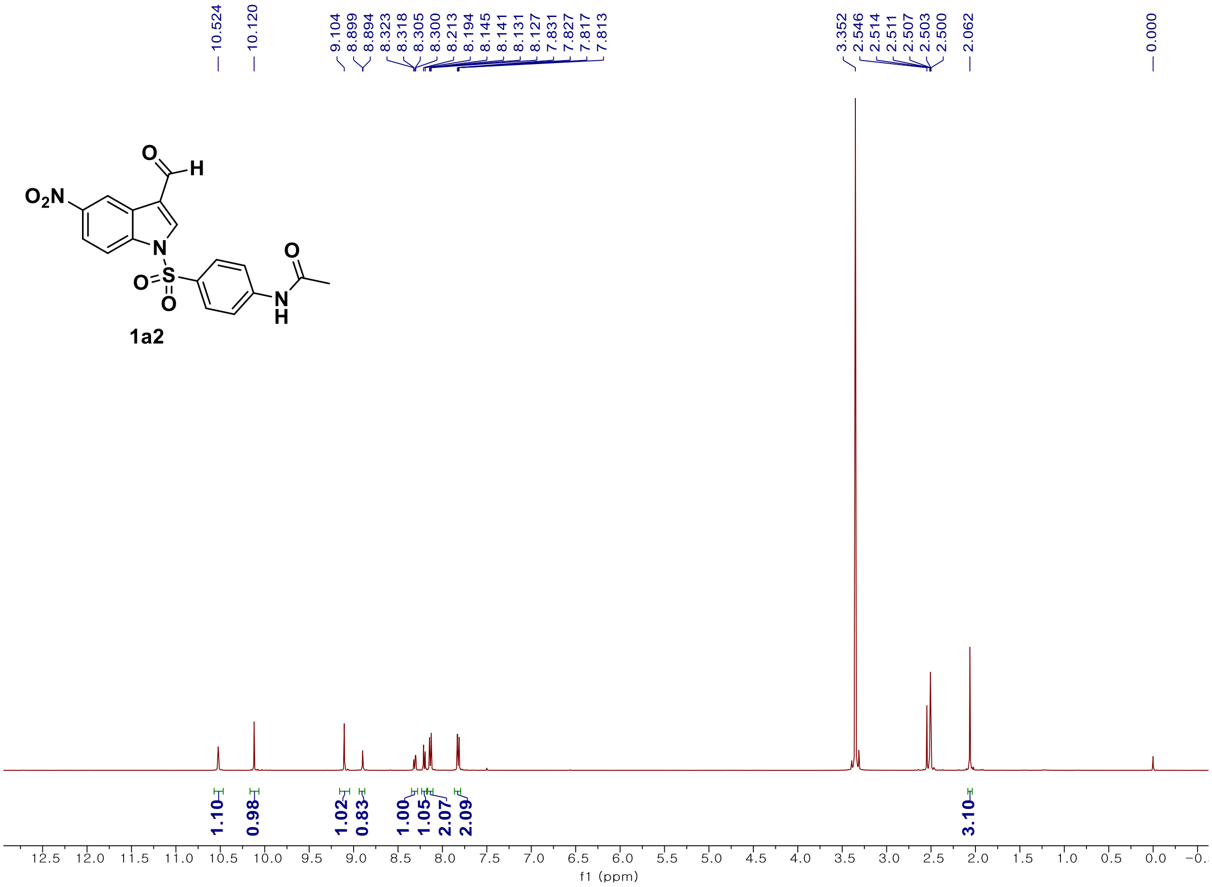
10.524
10.120
9.104
8.899
8.894
8.323
8.318
8.305
8.300
8.213
8.194
8.145
8.141
8.131
8.127
7.831
7.827
7.817
7.813
3.352
2.546
2.514
2.511
2.507
2.503
2.500
2.062
0.000
1a2
1.10
0.98
1.02
0.83
1.00
1.05
2.07
2.09
3.10
f1 (ppm)

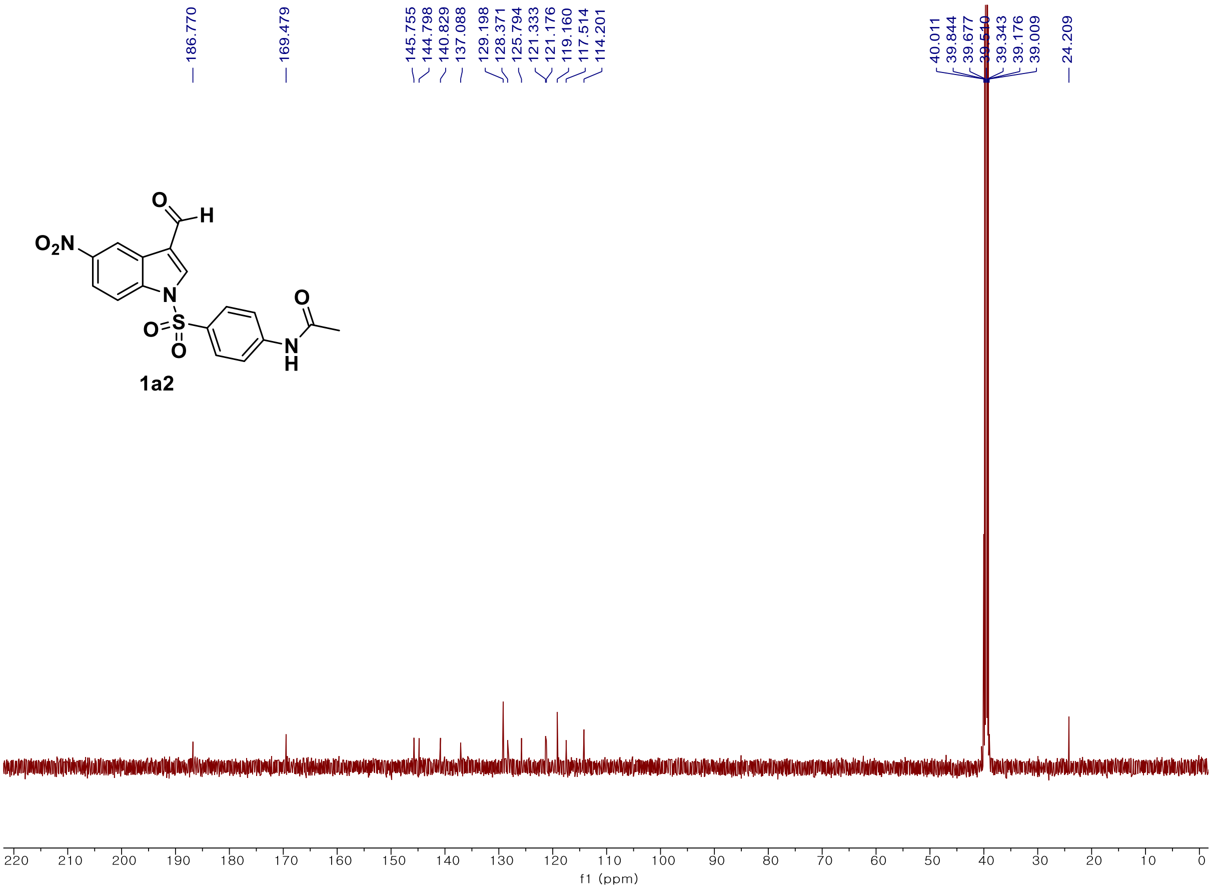
186.770
169.479
145.755
144.798
140.829
137.088
129.198
128.371
125.794
121.333
121.176
119.160
117.514
114.201
40.011
39.844
39.677
39.343
39.176
39.009
24.209
1a2
f1 (ppm)

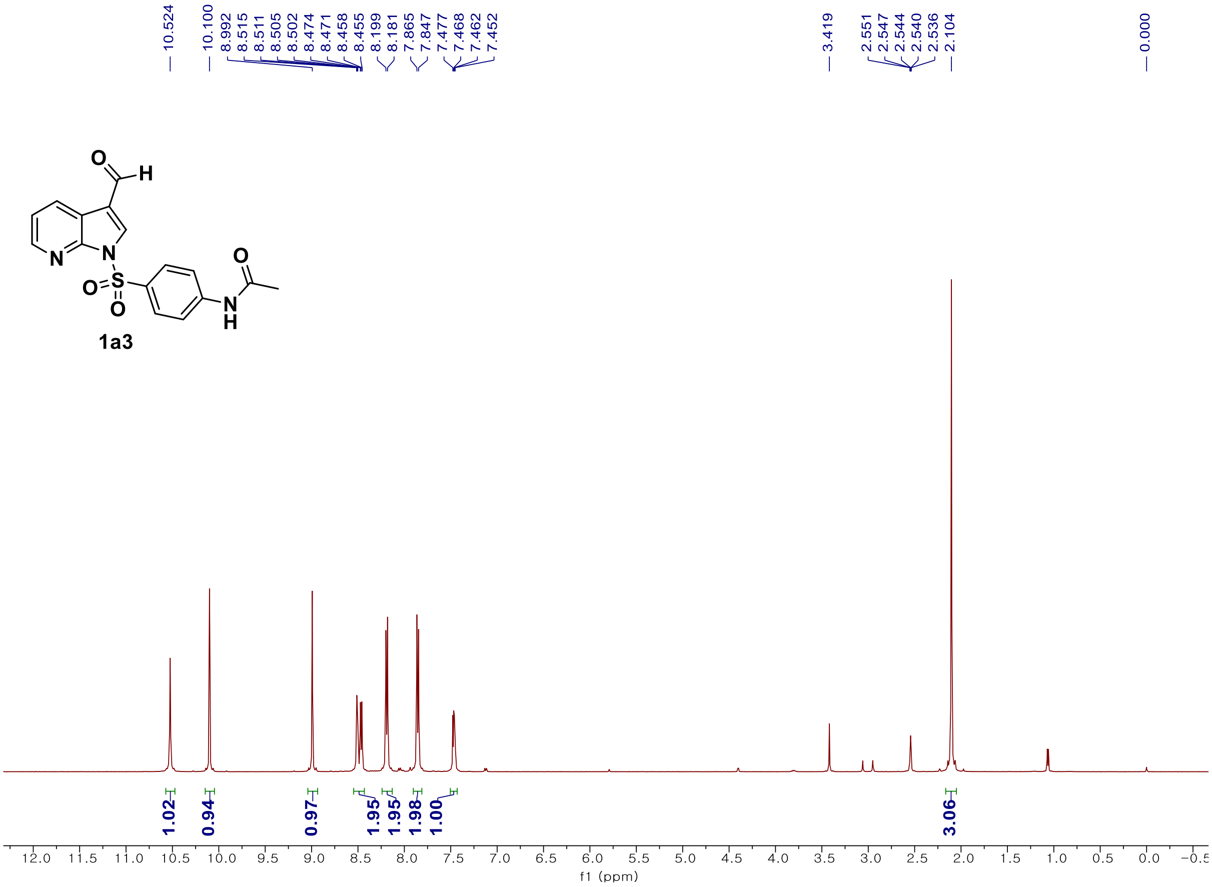
10.524
10.100
8.992
8.515
8.511
8.505
8.502
8.474
8.471
8.458
8.455
8.199
8.181
7.865
7.847
7.477
7.468
7.462
7.452
3.419
2.551
2.547
2.544
2.540
2.536
2.104
0.000
1a3
1.02
0.94
0.97
1.95
1.95
1.98
1.00
3.06
f1 (ppm)

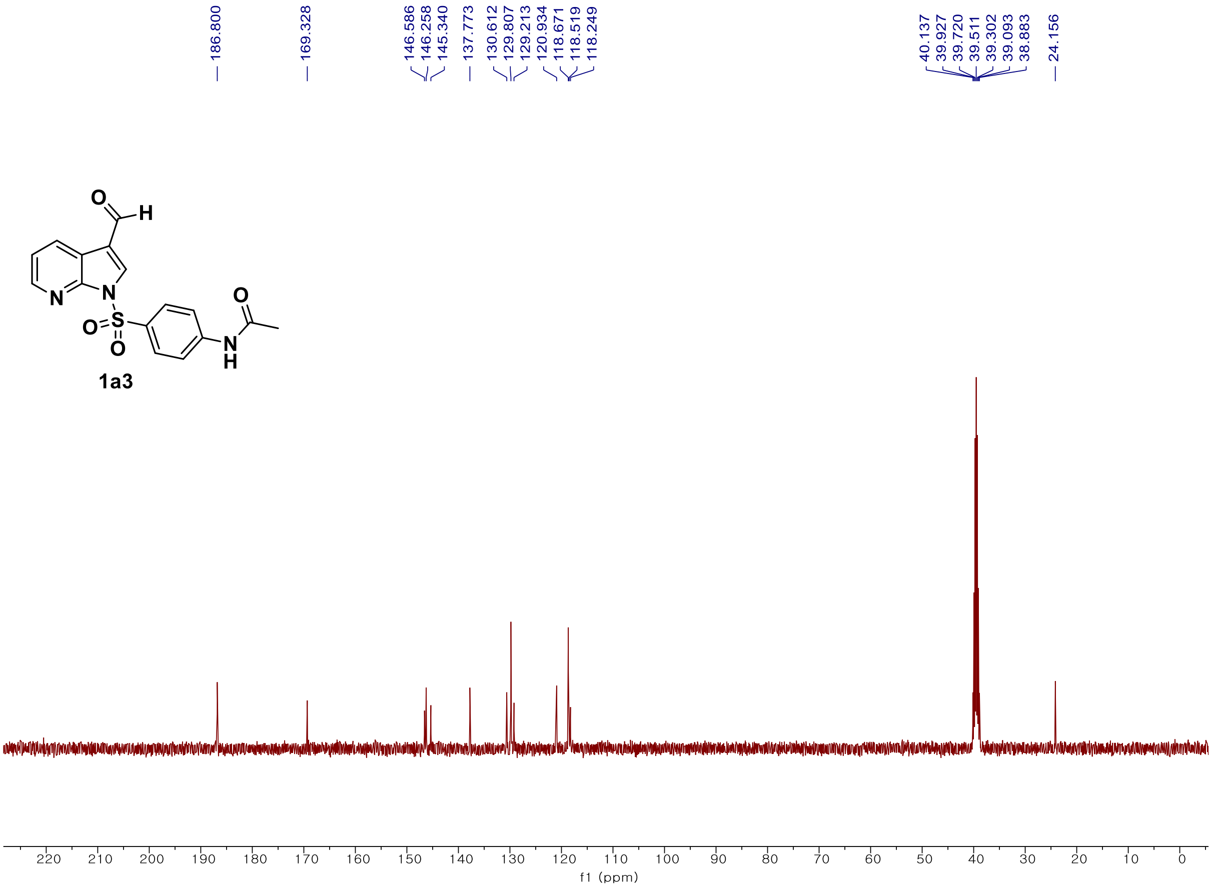
186.800
169.328
146.586
146.258
145.340
137.773
130.612
129.807
129.213
120.934
118.671
118.519
118.249
40.137
39.927
39.720
39.511
39.302
39.093
38.883
24.156
1a3
f1 (ppm)

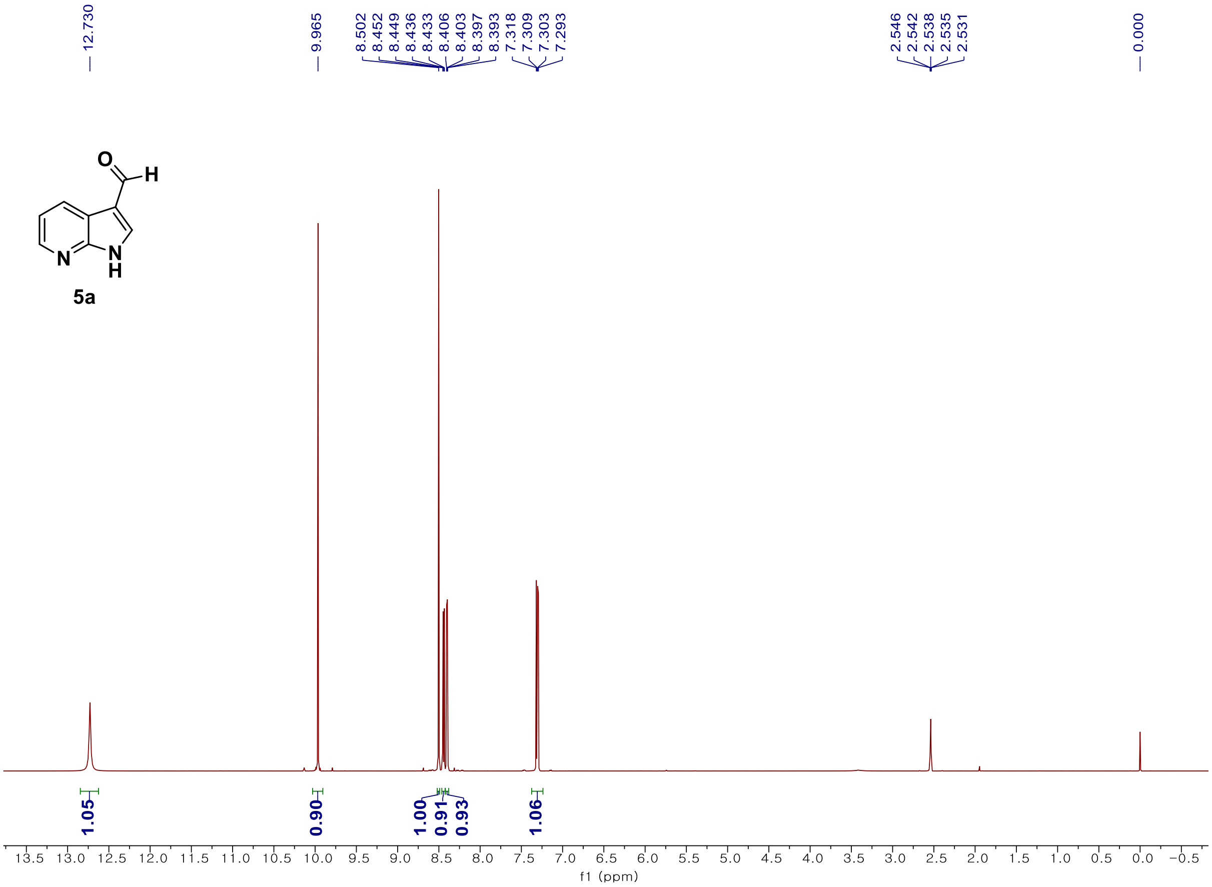

12.730
9.965
8.502
8.452
8.449
8.436
8.433
8.406
8.403
8.397
8.393
7.318
7.309
7.303
7.293
2.546
2.542
2.538
2.535
2.531
0.000
1.05
0.90
1.00
0.91
0.93
1.06
f1 (ppm)

5a

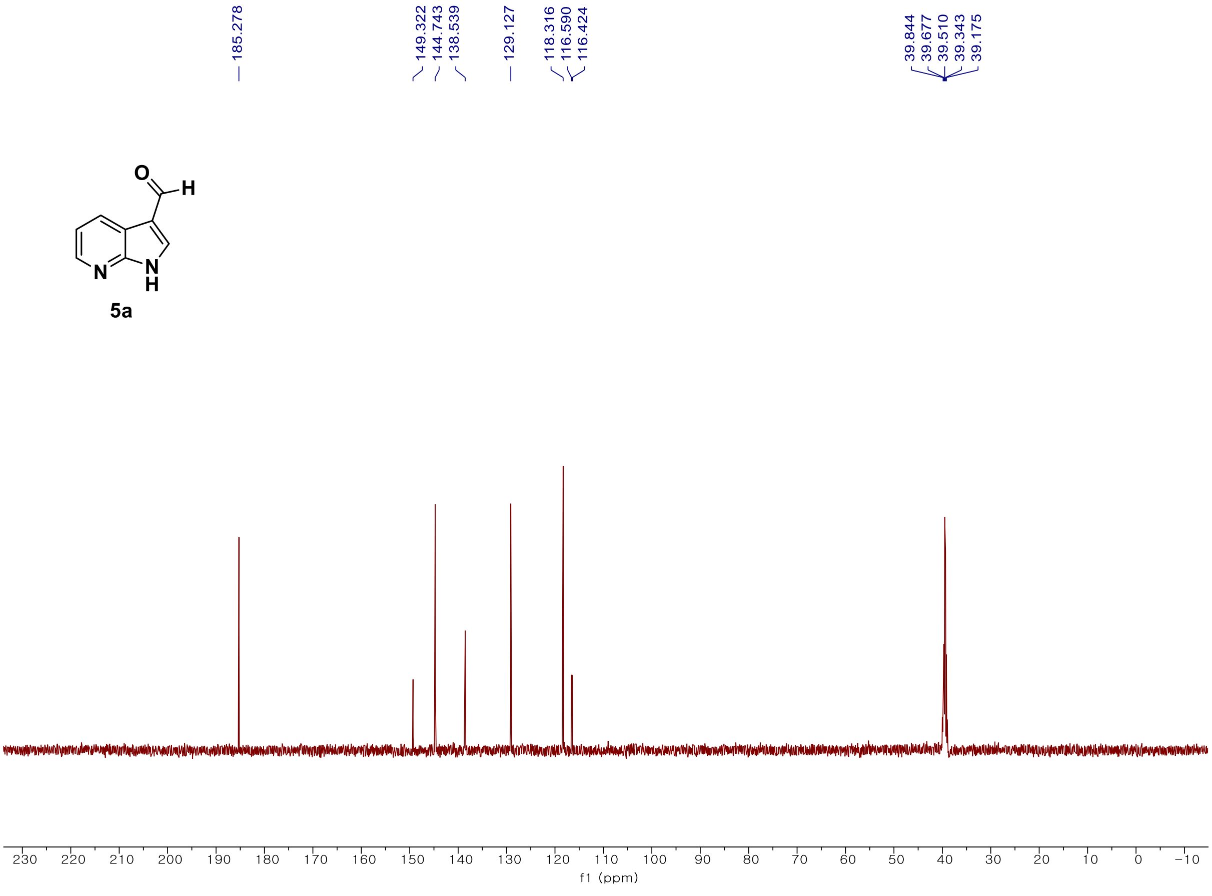

185.278
149.322
144.743
138.539
129.127
118.316
116.590
116.424
39.844
39.677
39.510
39.343
39.175
f1 (ppm)

5a

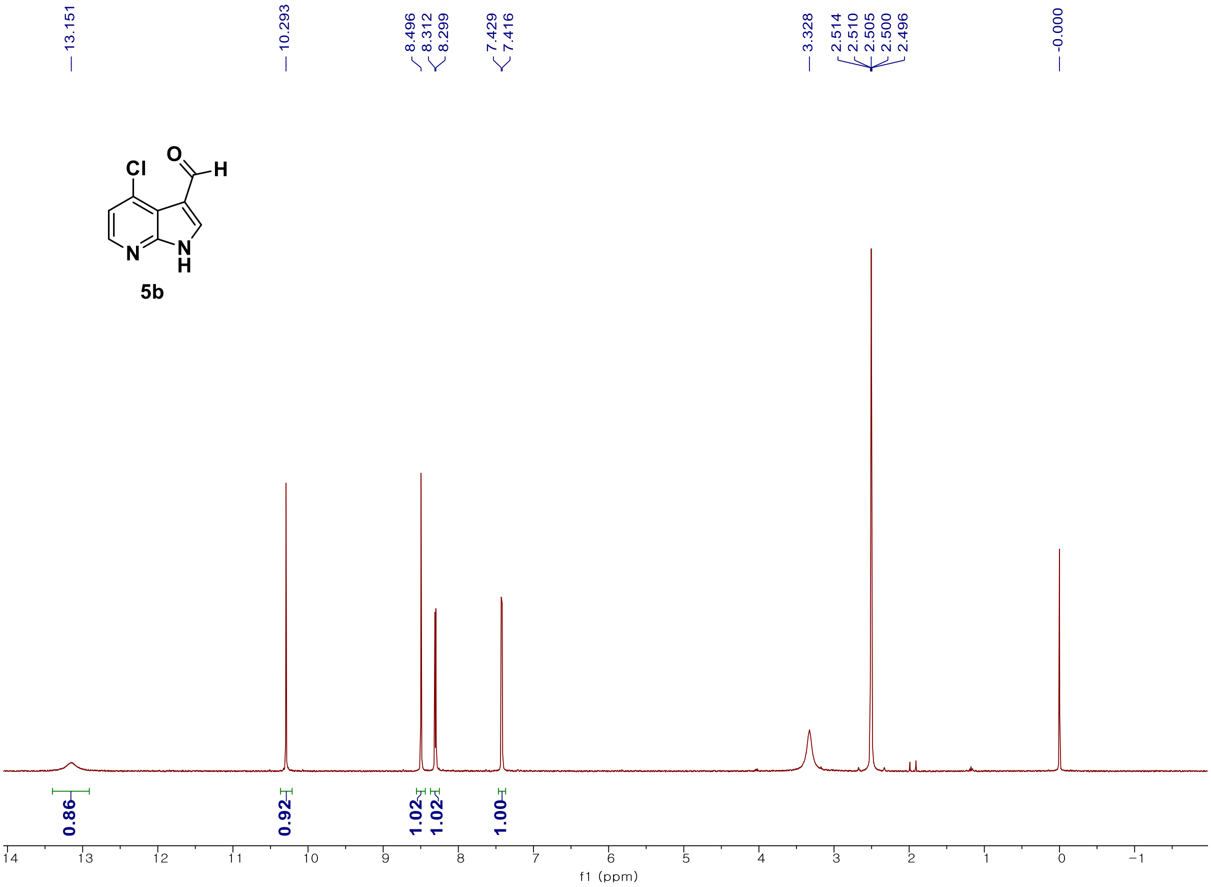
13.151
10.293
8.496
8.312
8.299
7.429
7.416
3.328
2.514
2.510
2.505
2.500
2.496
-0.000
Cl
O
H
N
N
H
5b
0.86
0.92
1.02
1.02
1.00
f1 (ppm)

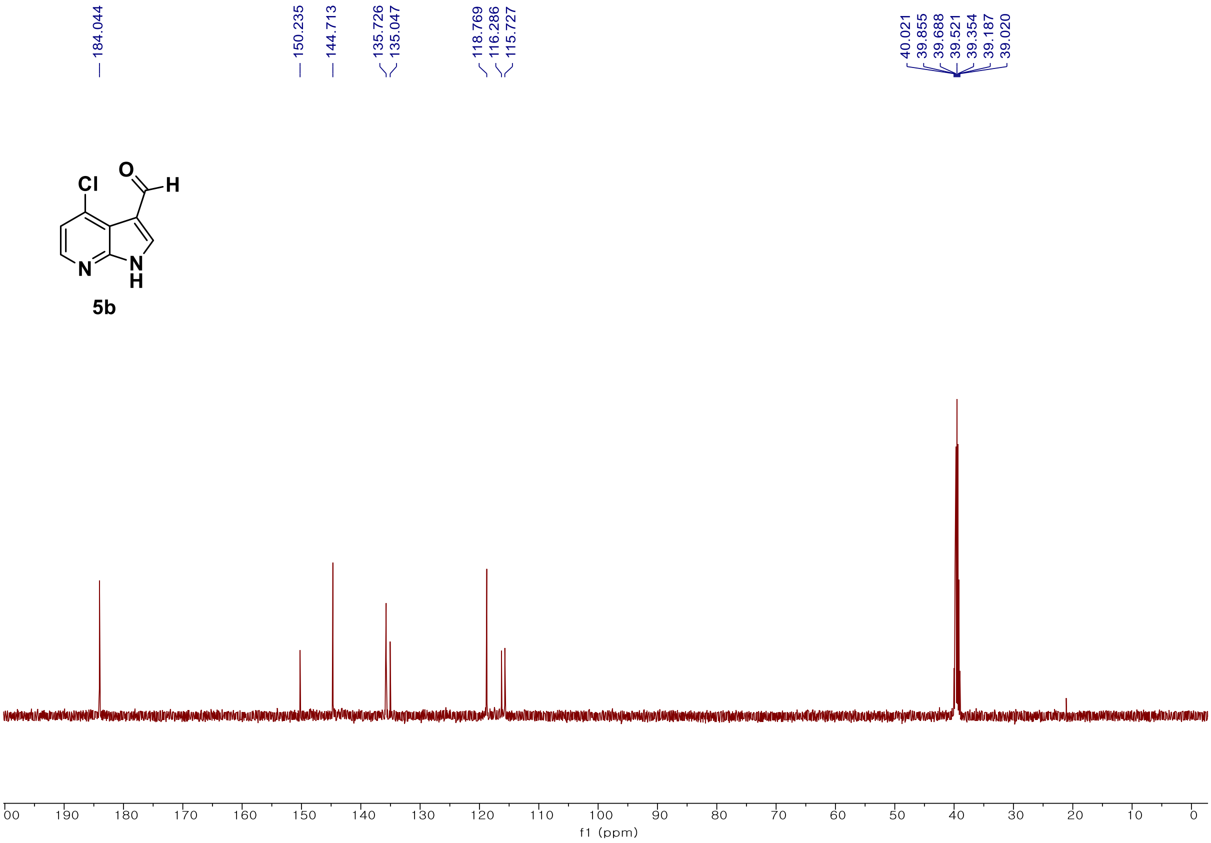
184.044
150.235
144.713
135.726
135.047
118.769
116.286
115.727
40.021
39.855
39.688
39.521
39.354
39.187
39.020
Cl
O
H
N
N
H
5b
f1 (ppm)

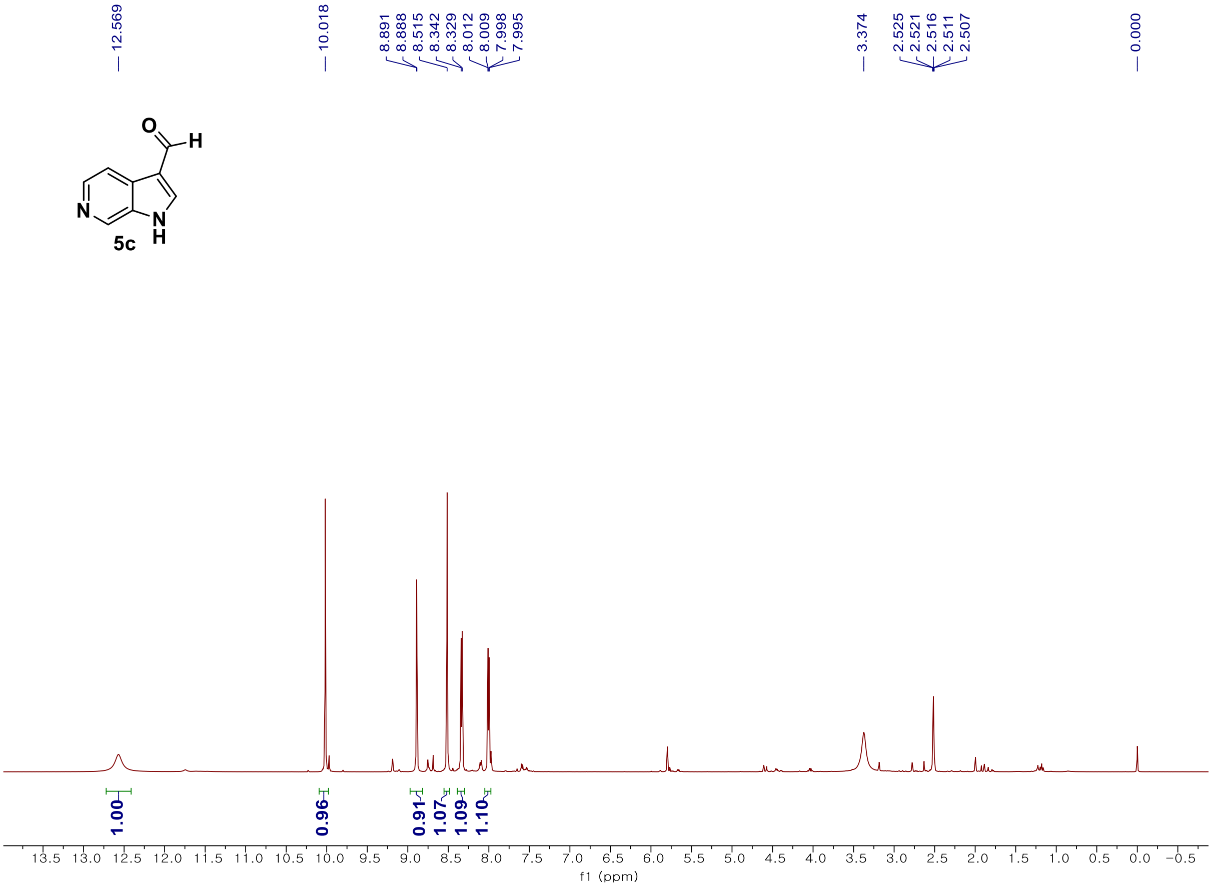
12.569
10.018
8.891
8.888
8.515
8.342
8.329
8.012
8.009
7.998
7.995
3.374
2.525
2.521
2.516
2.511
2.507
0.000
O
H
N
N
H
5c
1.00
0.96
0.91
1.07
1.09
1.10
f1 (ppm)

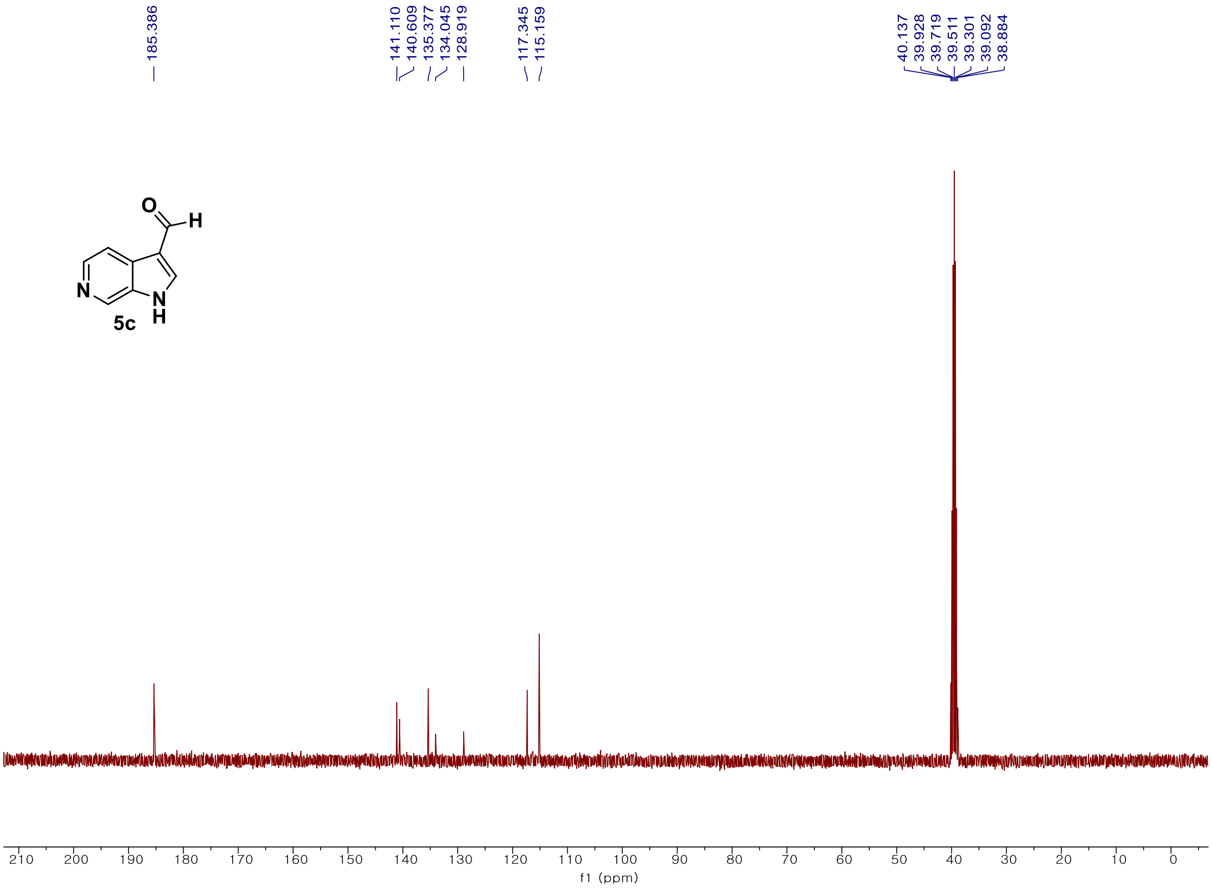
185.386
141.110
140.609
135.377
134.045
128.919
117.345
115.159
40.137
39.928
39.719
39.511
39.301
39.092
38.884
O
H
N
N
H
5c
f1 (ppm)

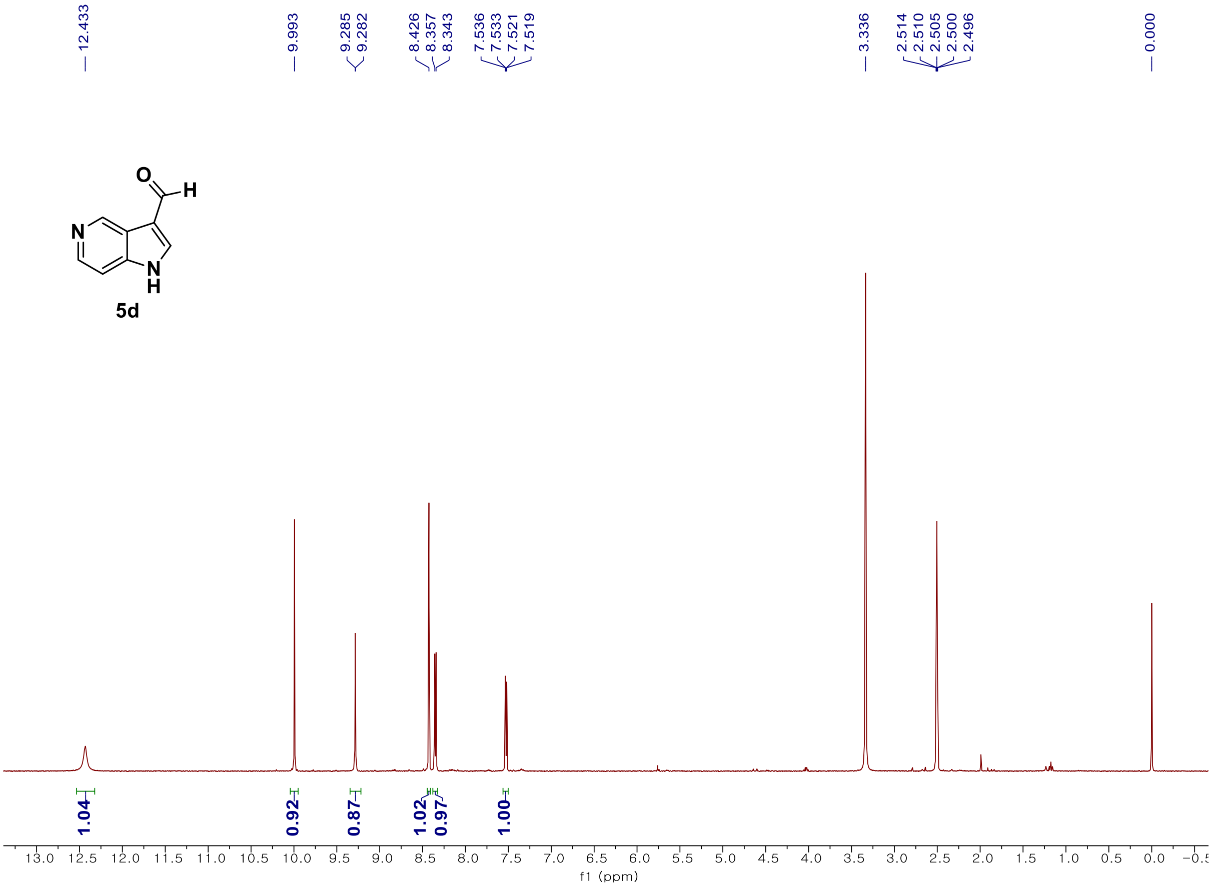
12.433
9.993
9.285
9.282
8.426
8.357
8.343
7.536
7.533
7.521
7.519
3.336
2.514
2.510
2.505
2.500
2.496
0.000
5d
1.04
0.92
0.87
1.02
0.97
1.00
f1 (ppm)

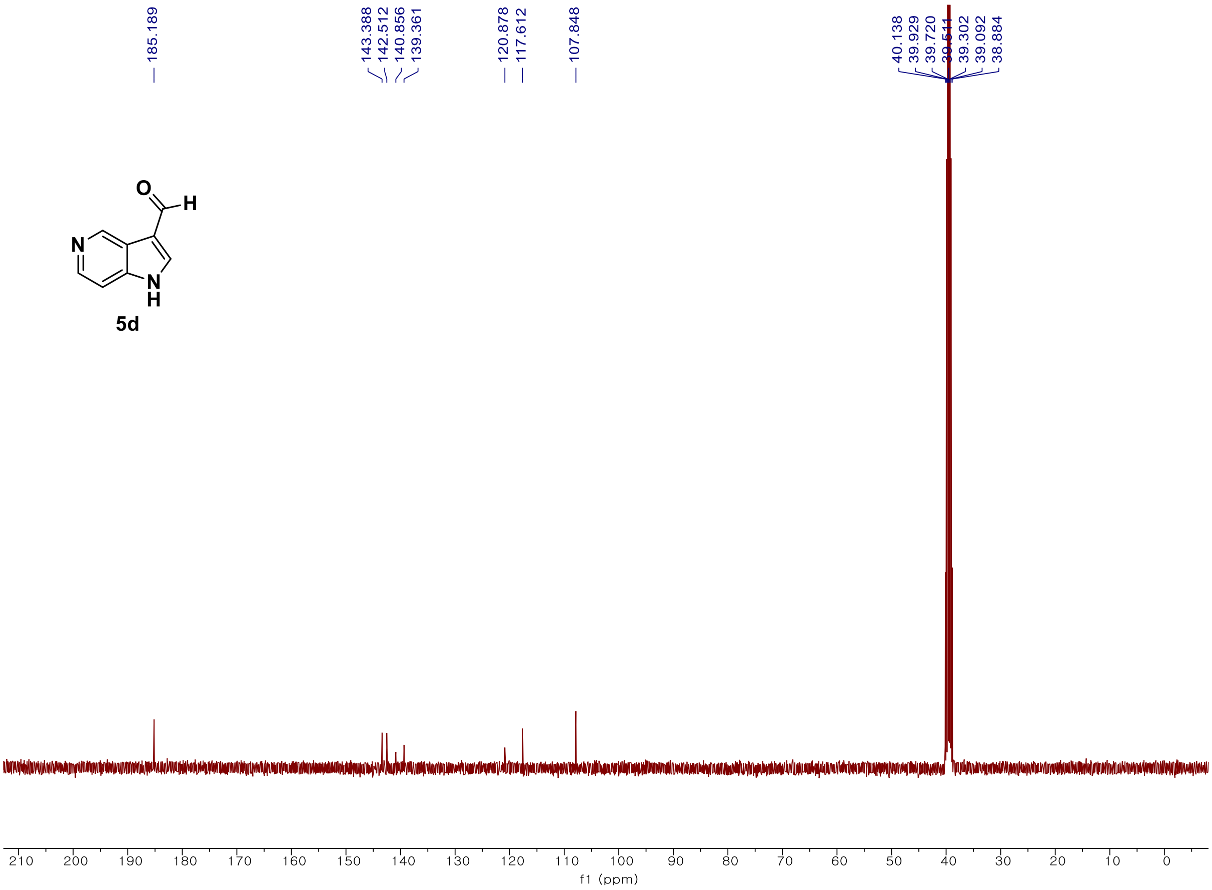
185.189
143.388
142.512
140.856
139.361
120.878
117.612
107.848
40.138
39.929
39.720
39.511
39.302
39.092
38.884
5d
f1 (ppm)

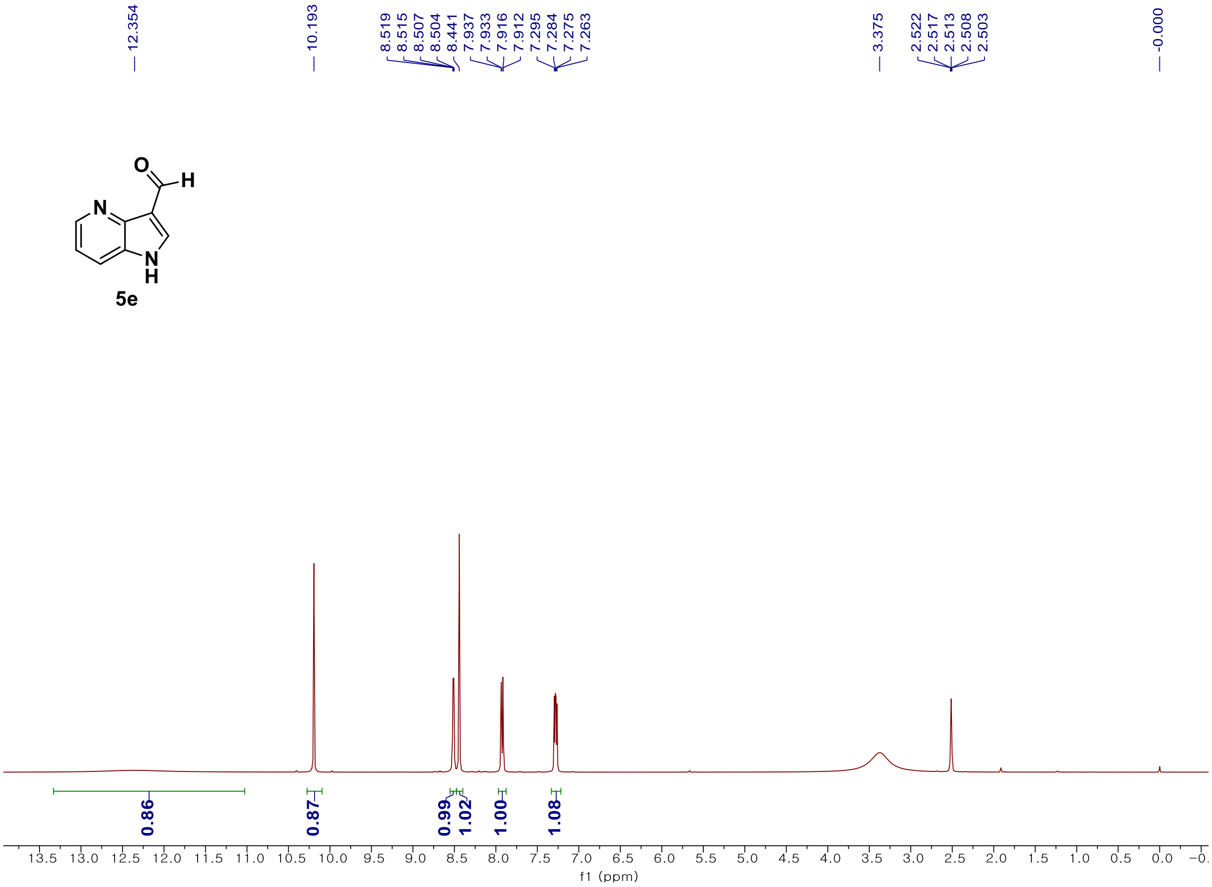
12.354
10.193
8.519
8.515
8.507
8.504
8.441
7.937
7.933
7.916
7.912
7.295
7.284
7.275
7.263
3.375
2.522
2.517
2.513
2.508
2.503
-0.000
O
H
N
N
H
5e
0.86
0.87
0.99
1.02
1.00
1.08
f1 (ppm)

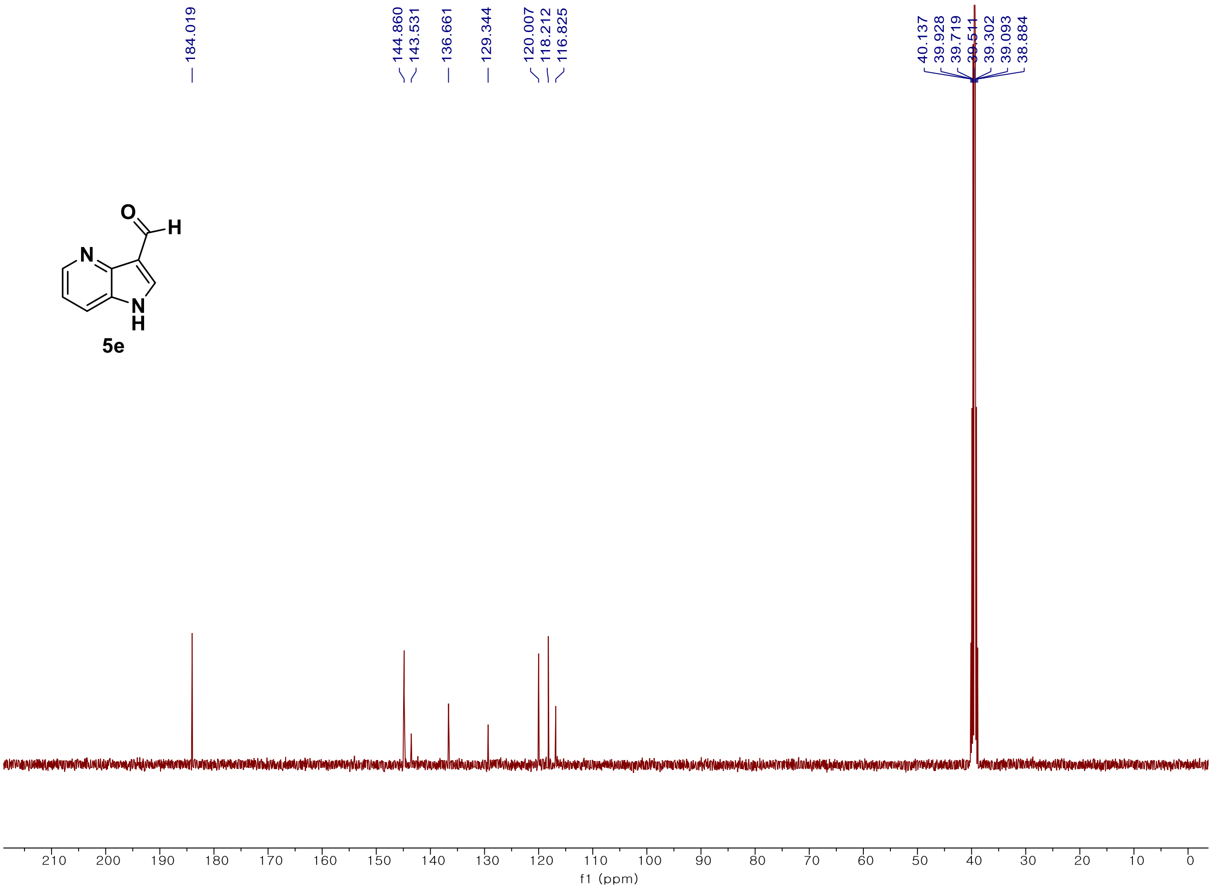
184.019
144.860
143.531
136.661
129.344
120.007
118.212
116.825
40.137
39.928
39.719
39.511
39.302
39.093
38.884
O
H
N
N
H
5e
f1 (ppm)

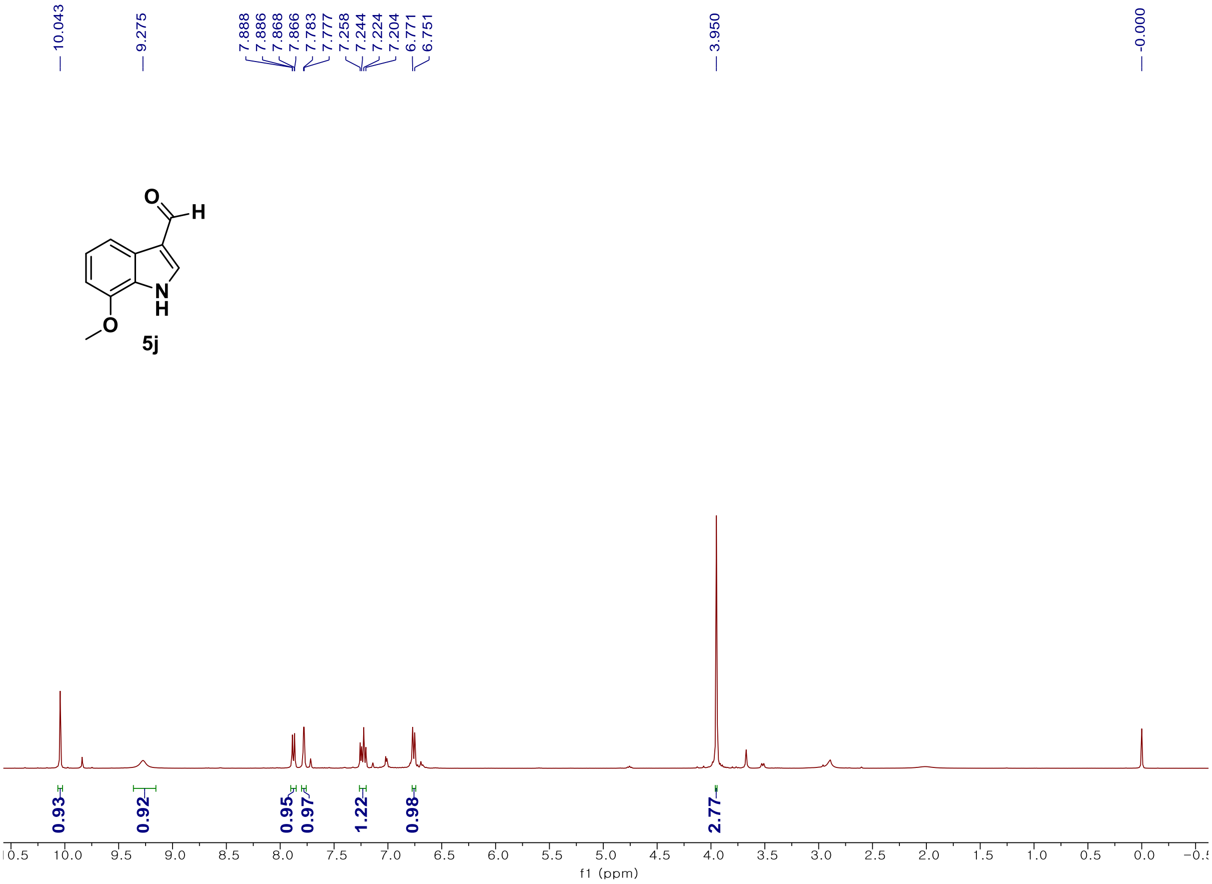
10.043
9.275
7.888
7.886
7.868
7.866
7.783
7.777
7.258
7.244
7.224
7.204
6.771
6.751
3.950
-0.000
5j
0.93
0.92
0.95
0.97
1.22
0.98
2.77
f1 (ppm)

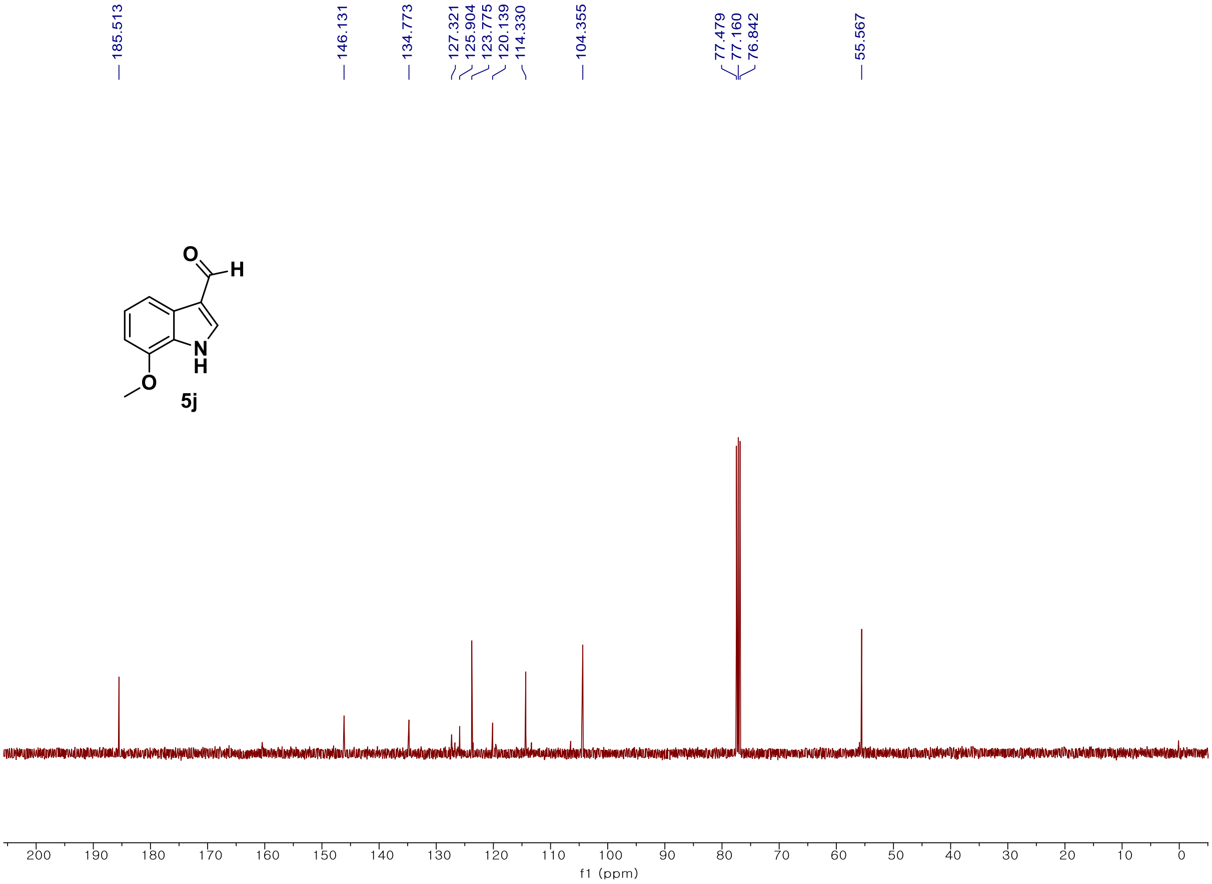
185.513
146.131
134.773
127.321
125.904
123.775
120.139
114.330
104.355
77.479
77.160
76.842
55.567
5j
f1 (ppm)

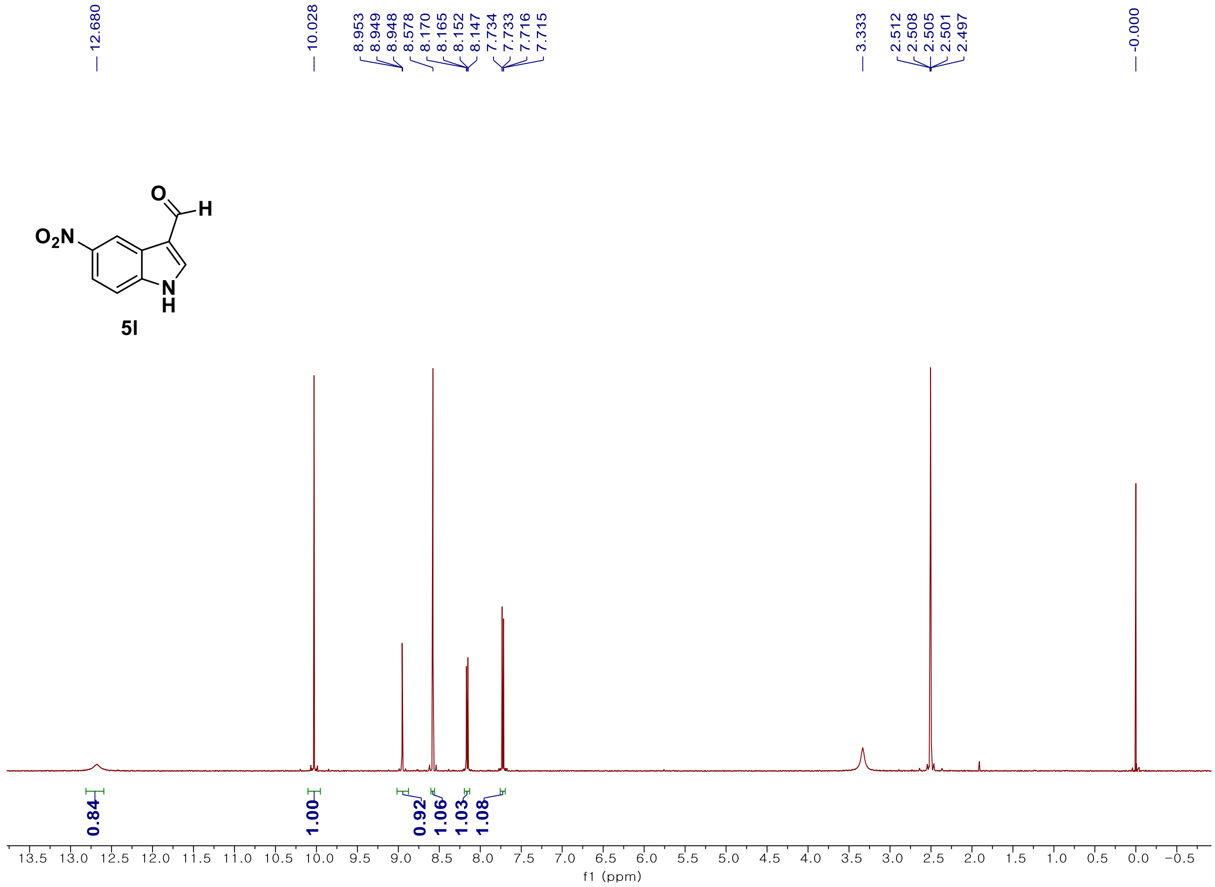
12.680
10.028
8.953
8.949
8.948
8.578
8.170
8.165
8.152
8.147
7.734
7.733
7.716
7.715
3.333
2.512
2.508
2.505
2.501
2.497
-0.000
O
H
O2N
N
H
5l
0.84
1.00
0.92
1.06
1.03
1.08
f1 (ppm)

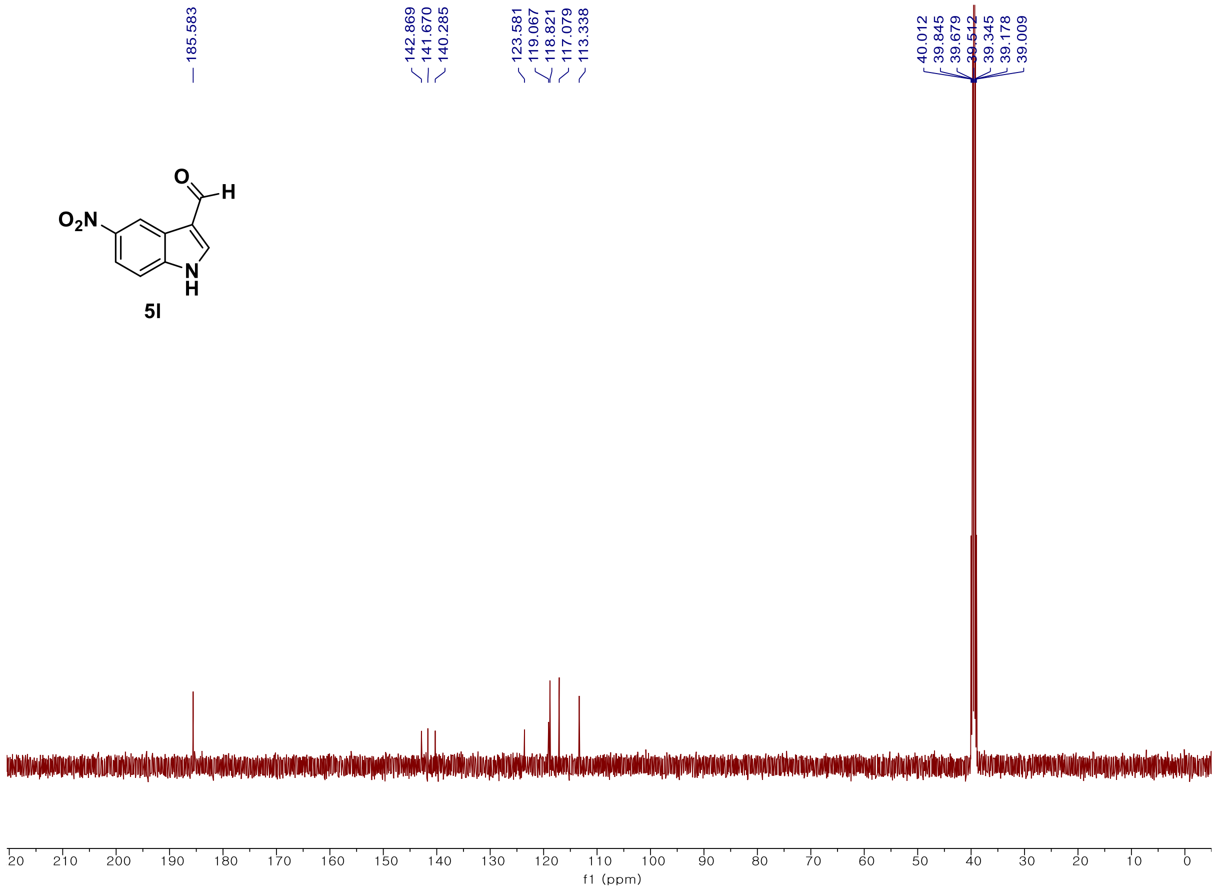
185.583
142.869
141.670
140.285
123.581
119.067
118.821
117.079
113.338
40.012
39.845
39.679
39.512
39.345
39.178
39.009
O
H
O2N
N
H
5l
f1 (ppm)

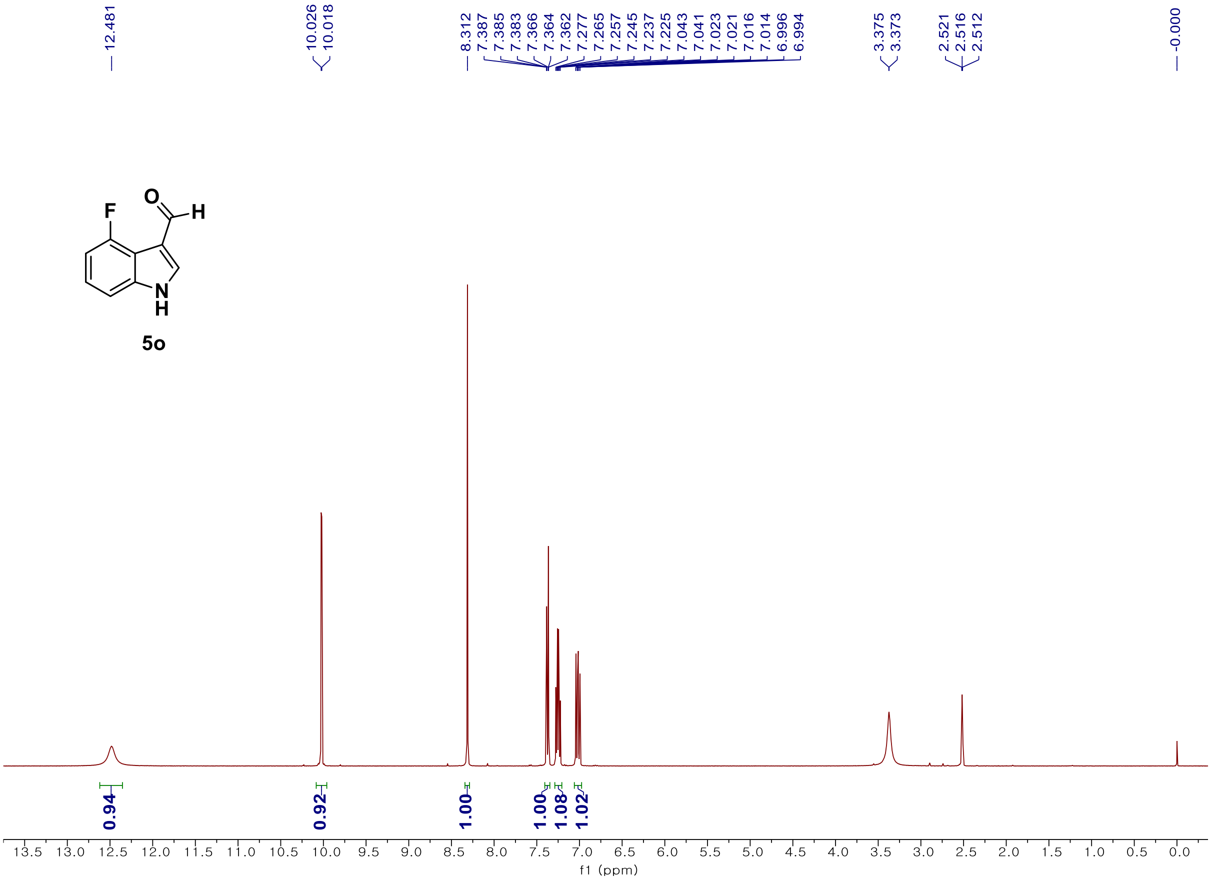
12.481
10.026
10.018
8.312
7.387
7.385
7.383
7.366
7.364
7.362
7.277
7.265
7.257
7.245
7.237
7.225
7.043
7.041
7.023
7.021
7.016
7.014
6.996
6.994
3.375
3.373
2.521
2.516
2.512
-0.000
F
O
H
N
H
5o
0.94
0.92
1.00
1.00
1.08
1.02
13.5 13.0 12.5 12.0 11.5 11.0 10.5 10.0 9.5 9.0 8.5 8.0 7.5 7.0 6.5 6.0 5.5 5.0 4.5 4.0 3.5 3.0 2.5 2.0 1.5 1.0 0.5 0.0
f1 (ppm)

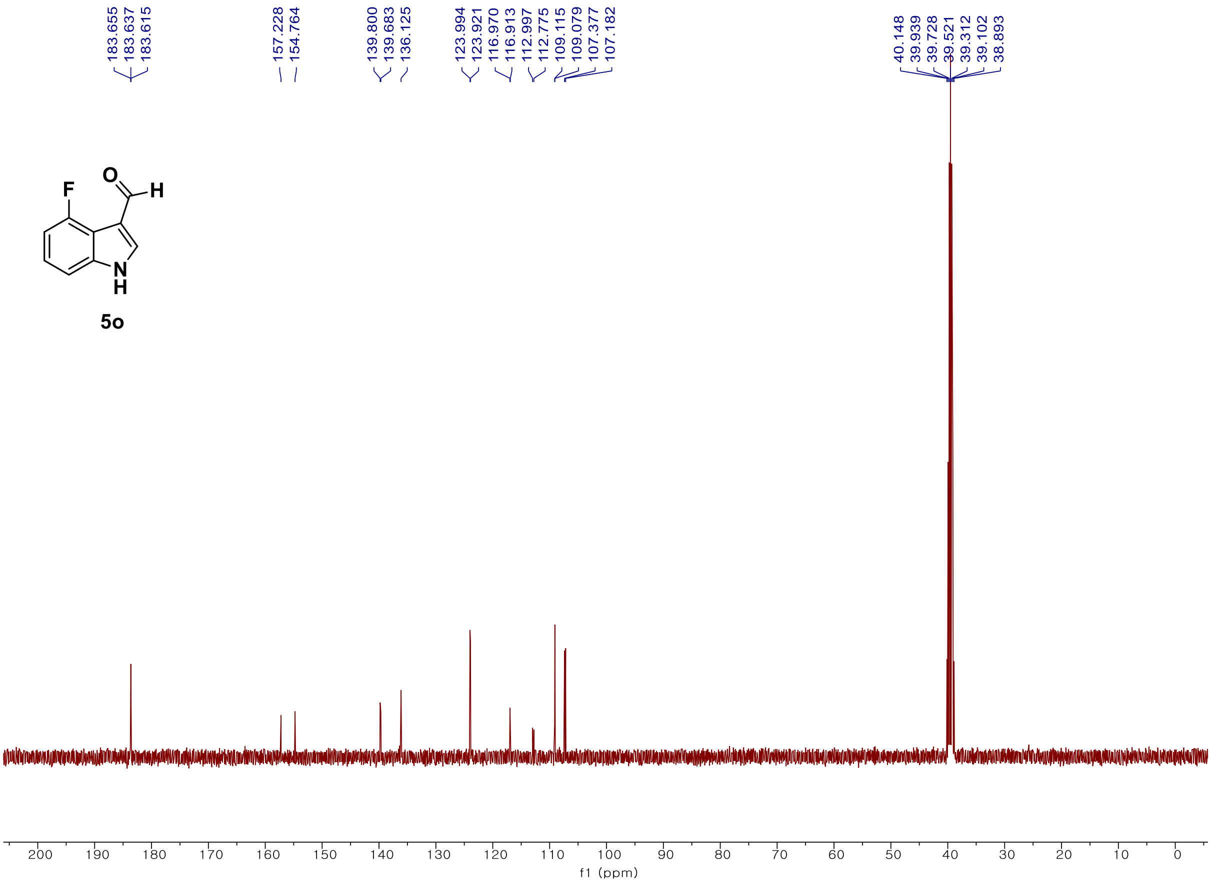
183.655
183.637
183.615
157.228
154.764
139.800
139.683
136.125
123.994
123.921
116.970
116.913
112.997
112.775
109.115
109.079
107.377
107.182
40.148
39.939
39.728
39.521
39.312
39.102
38.893
F
O
H
N
H
5o
200 190 180 170 160 150 140 130 120 110 100 90 80 70 60 50 40 30 20 10 0
f1 (ppm)

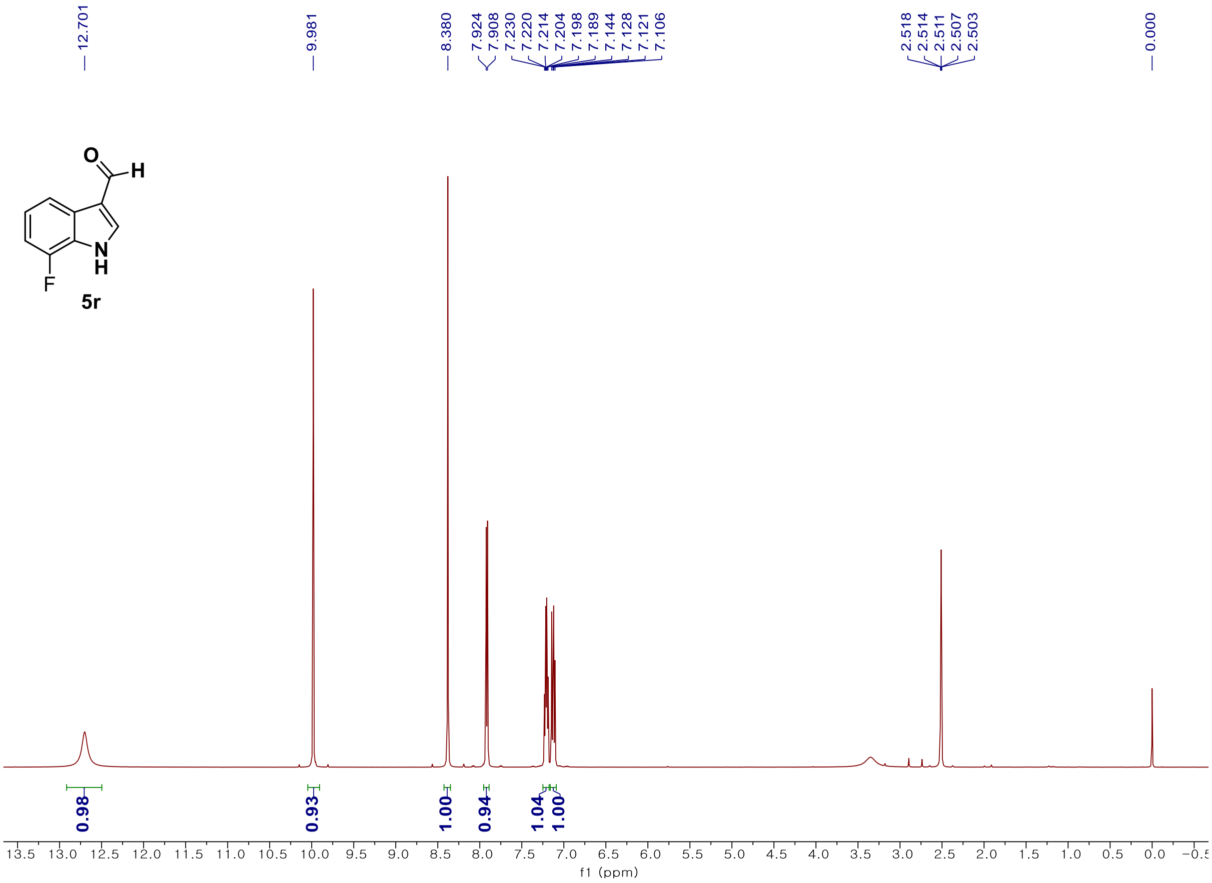
12.701
9.981
8.380
7.924
7.908
7.230
7.220
7.214
7.204
7.198
7.189
7.144
7.128
7.121
7.106
2.518
2.514
2.511
2.507
2.503
0.000
O
H
N
H
F
5r
0.98
0.93
1.00
0.94
1.04
1.00
f1 (ppm)

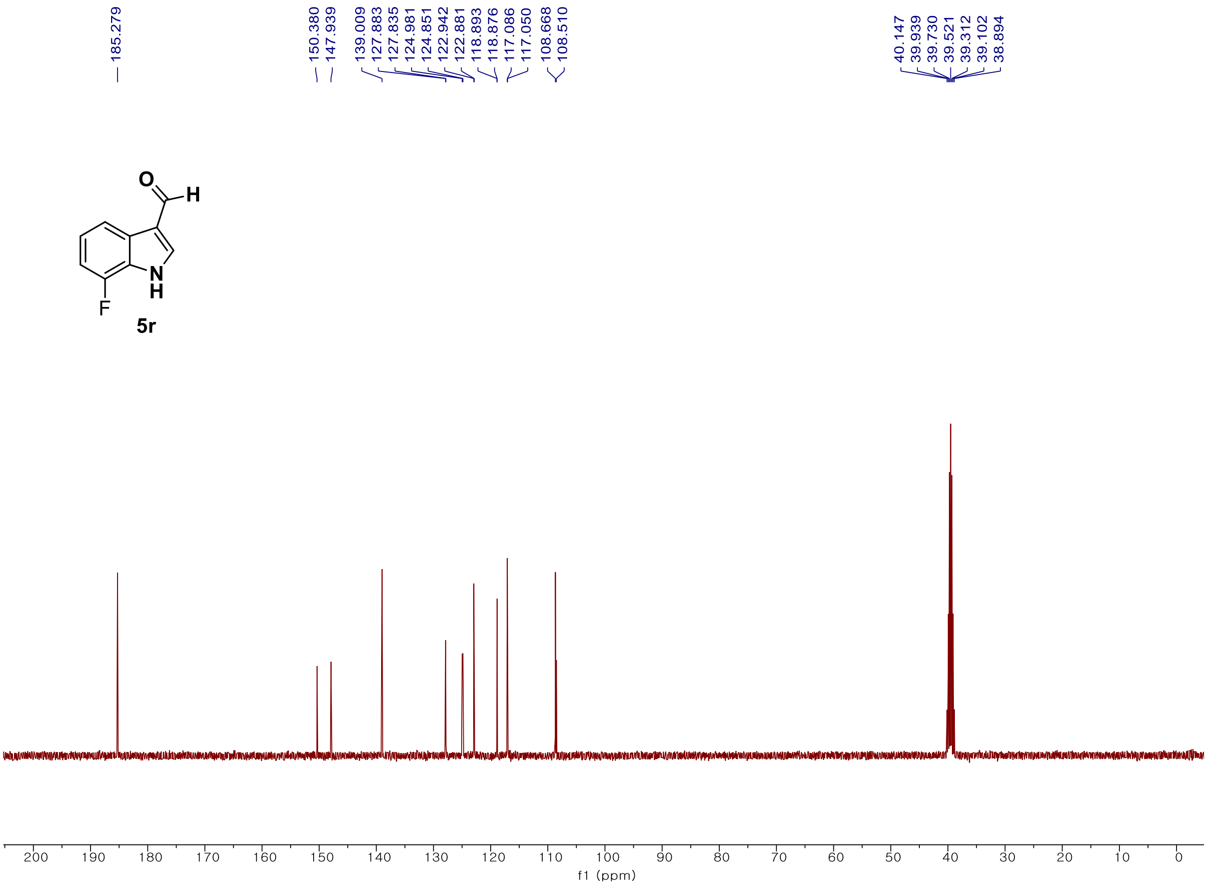
185.279
150.380
147.939
139.009
127.883
127.835
124.981
124.851
122.942
122.881
118.893
118.876
117.086
117.050
108.668
108.510
40.147
39.939
39.730
39.521
39.312
39.102
38.894
O
H
N
H
F
5r
f1 (ppm)

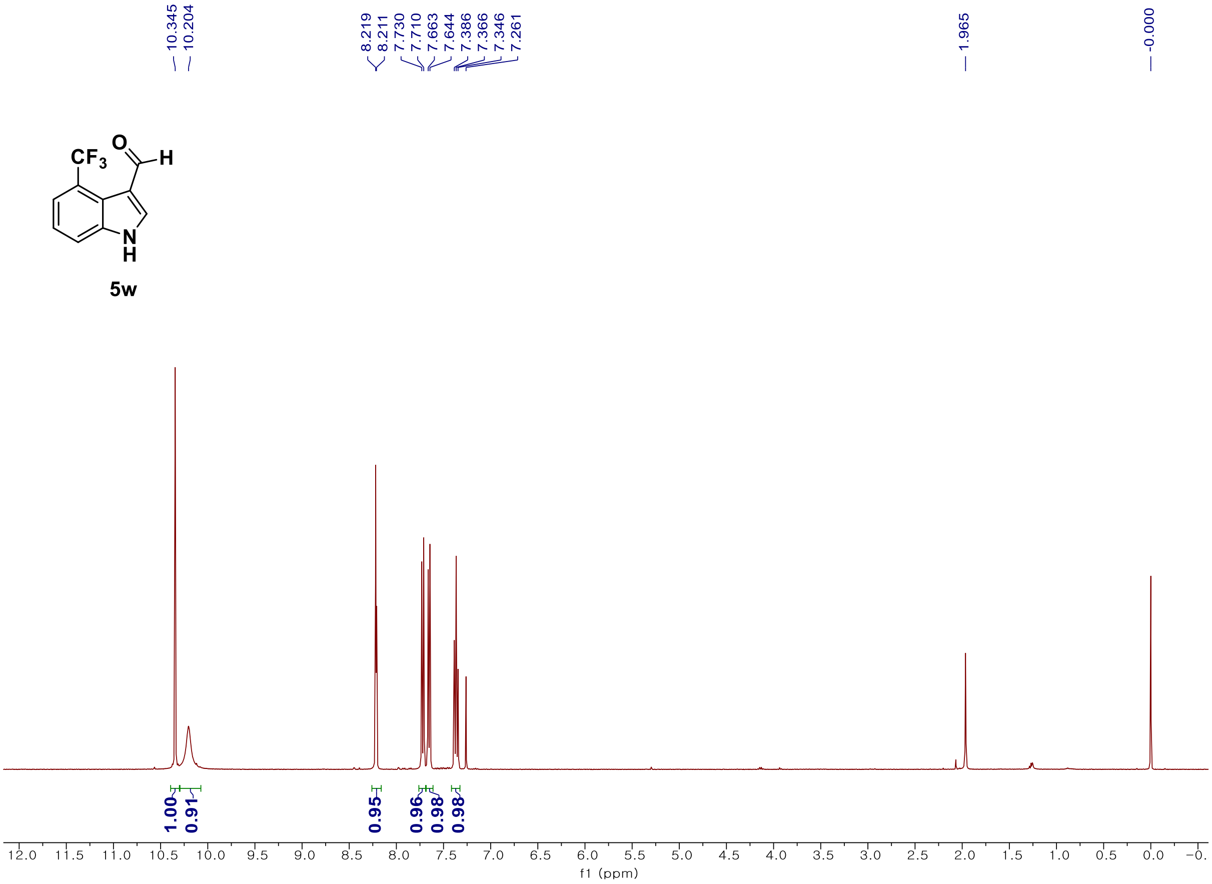
10.345
10.204
8.219
8.211
7.730
7.710
7.663
7.644
7.386
7.366
7.346
7.261
1.965
-0.000
CF3
O
H
N
H
5w
1.00
0.91
0.95
0.96
0.98
0.98
f1 (ppm)

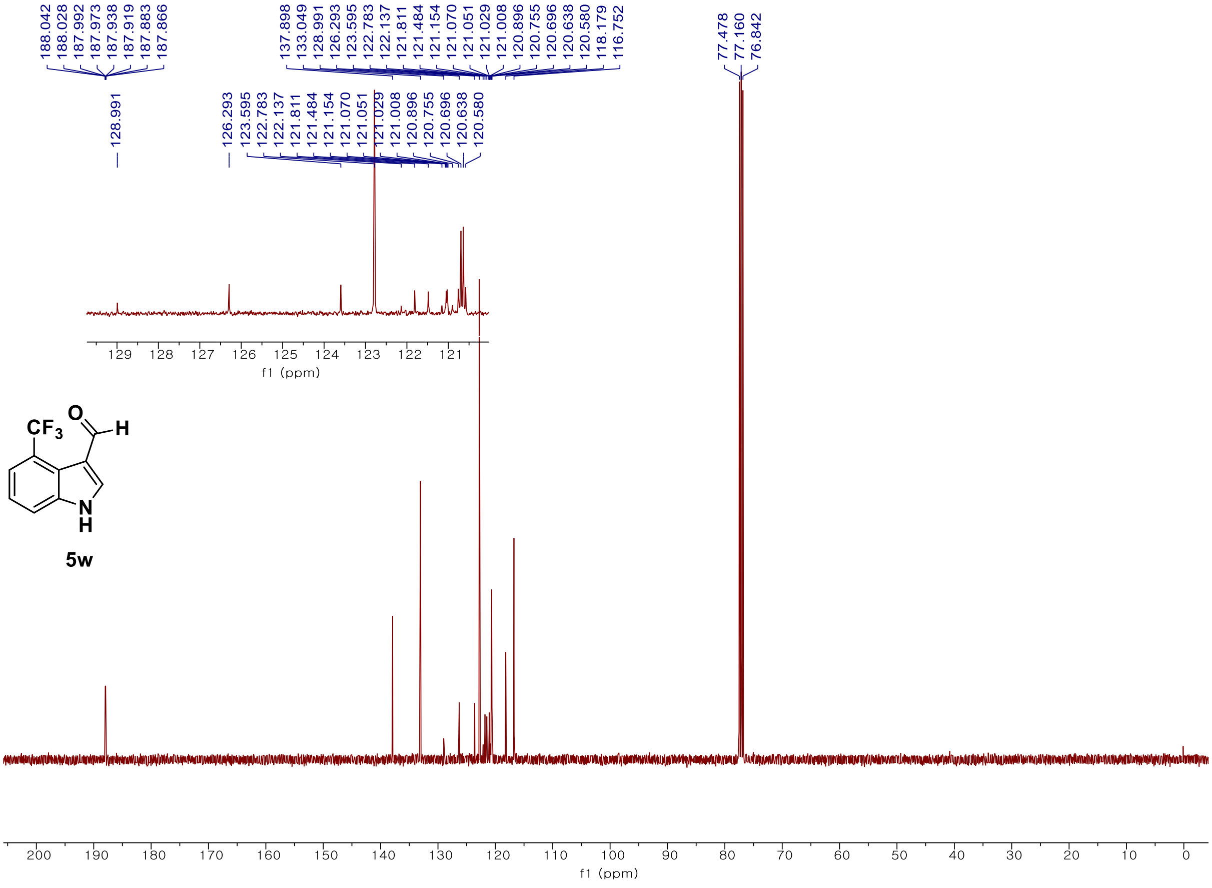
188.042
188.028
187.992
187.973
187.938
187.919
187.883
187.866
137.898
133.049
128.991
126.293
123.595
122.783
122.137
121.811
121.484
121.154
121.070
121.051
121.029
121.008
120.896
120.755
120.696
120.638
120.580
118.179
116.752
77.478
77.160
76.842
128.991
126.293
123.595
122.783
122.137
121.811
121.484
121.154
121.070
121.051
121.029
121.008
120.896
120.755
120.696
120.638
120.580
f1 (ppm)
CF3
O
H
N
H
5w
f1 (ppm)

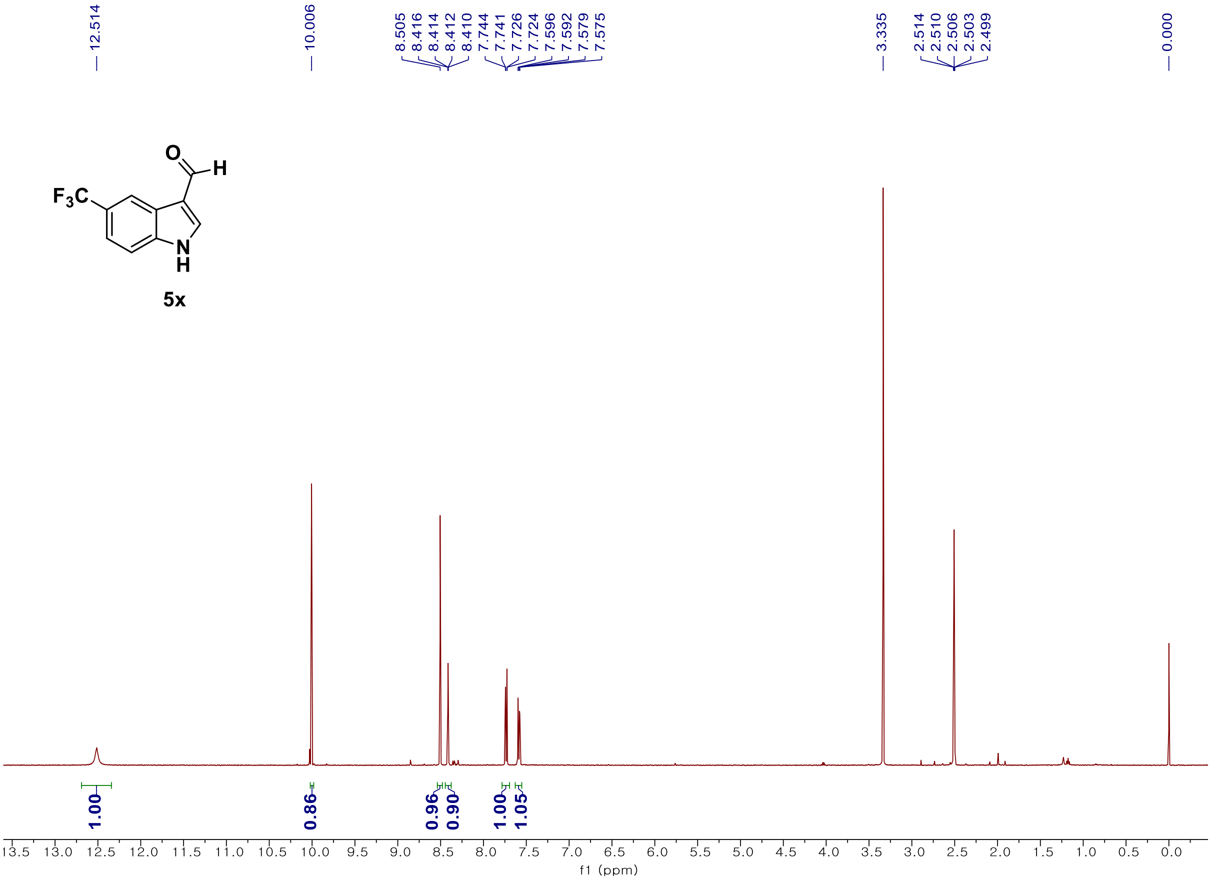
12.514
10.006
8.505 8.416 8.414 8.412 8.410 7.744 7.741 7.726 7.724 7.596 7.592 7.579 7.575
3.335
2.514 2.510 2.506 2.503 2.499
0.000
F3C
O
H
N
H
5x
1.00
0.86
0.96
0.90
1.00
1.05
f1 (ppm)

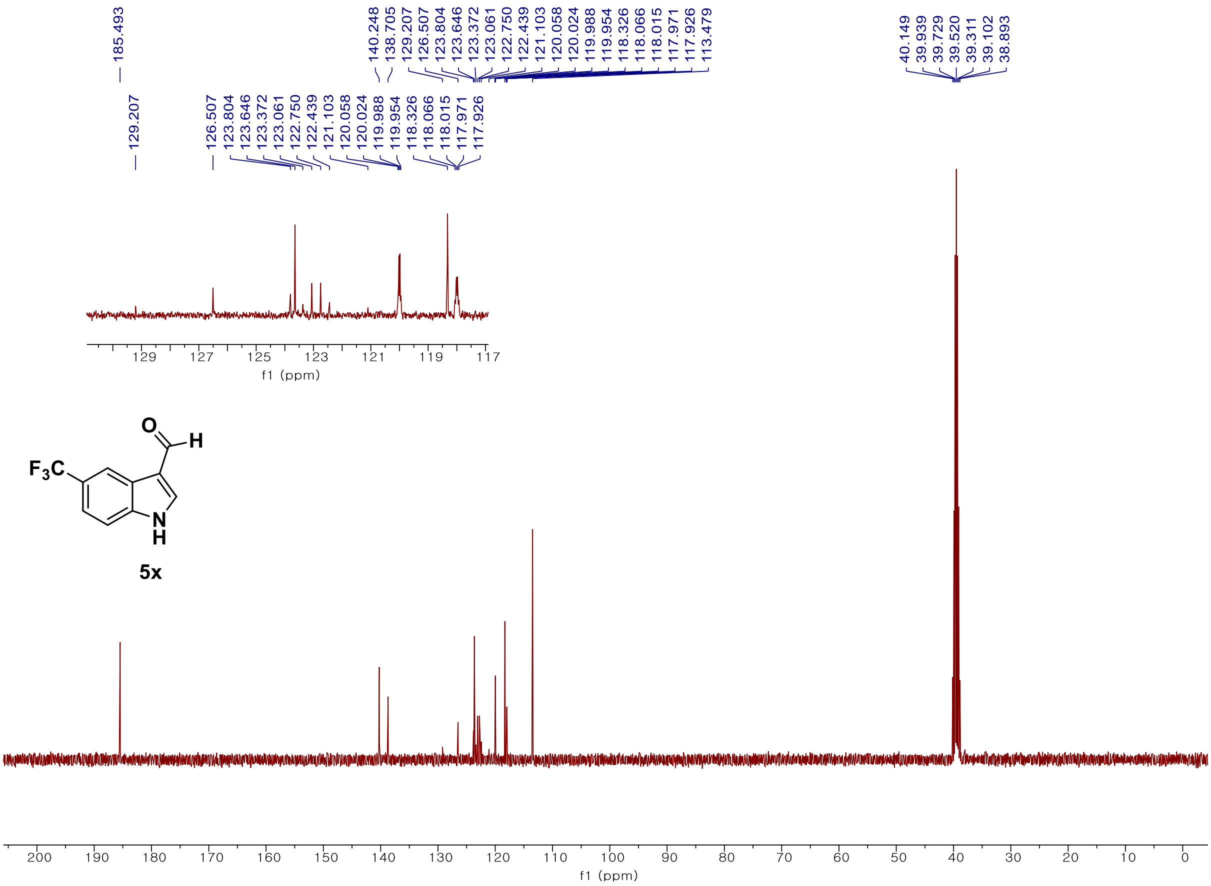
185.493
140.248 138.705 129.207 126.507 123.804 123.646 123.372 123.061 122.750 122.439 121.103 120.058 120.024 119.988 119.954 118.326 118.066 118.015 117.971 117.926 113.479
40.149 39.939 39.729 39.520 39.311 39.102 38.893
129.207
126.507 123.804 123.646 123.372 123.061 122.750 122.439 121.103 120.058 120.024 119.988 119.954 118.326 118.066 118.015 117.971 117.926
f1 (ppm)
F3C
O
H
N
H
5x
f1 (ppm)

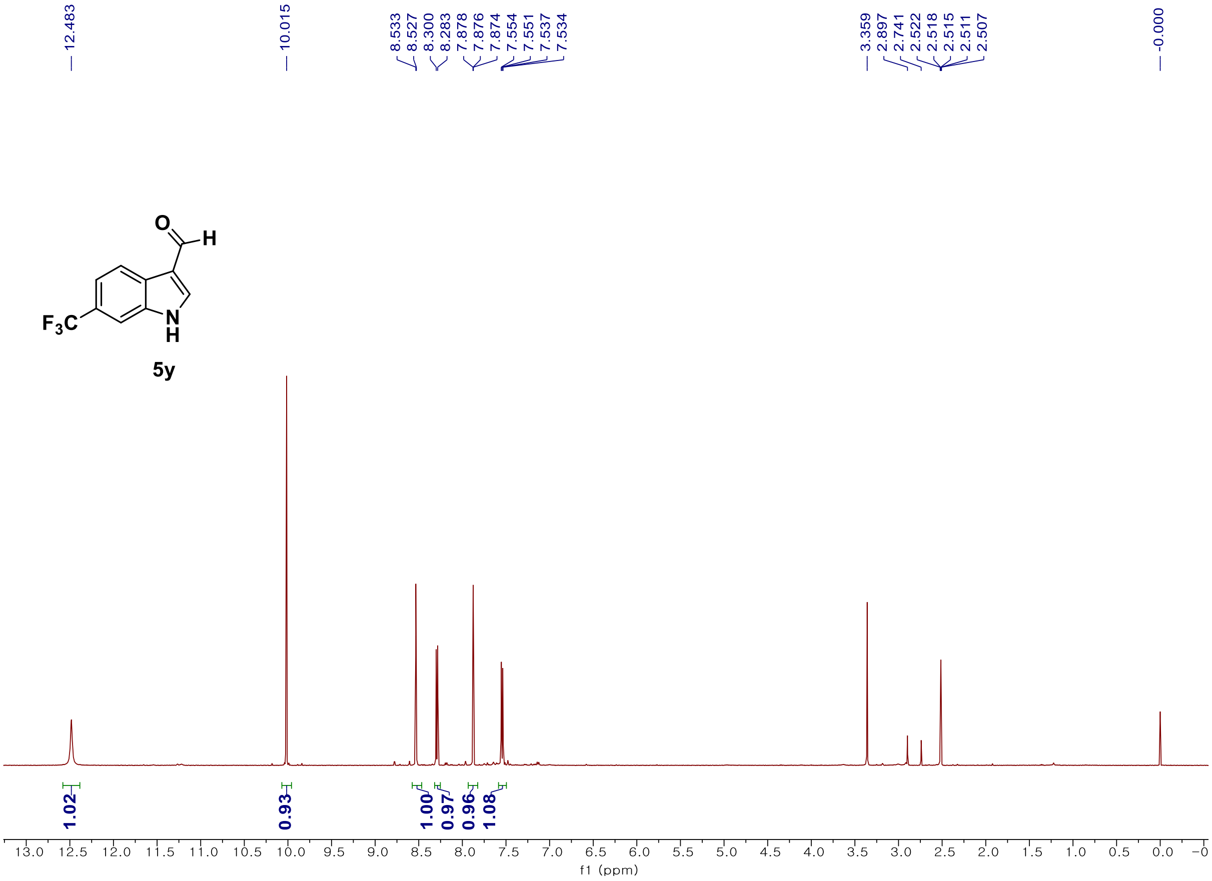
12.483
10.015
8.533
8.527
8.300
8.283
7.878
7.876
7.874
7.554
7.551
7.537
7.534
3.359
2.897
2.741
2.522
2.518
2.515
2.511
2.507
-0.000
O
H
F3C
N
H
5y
1.02
0.93
1.00
0.97
0.96
1.08
13.0 12.5 12.0 11.5 11.0 10.5 10.0 9.5 9.0 8.5 8.0 7.5 7.0 6.5 6.0 5.5 5.0 4.5 4.0 3.5 3.0 2.5 2.0 1.5 1.0 0.5 0.0
f1 (ppm)

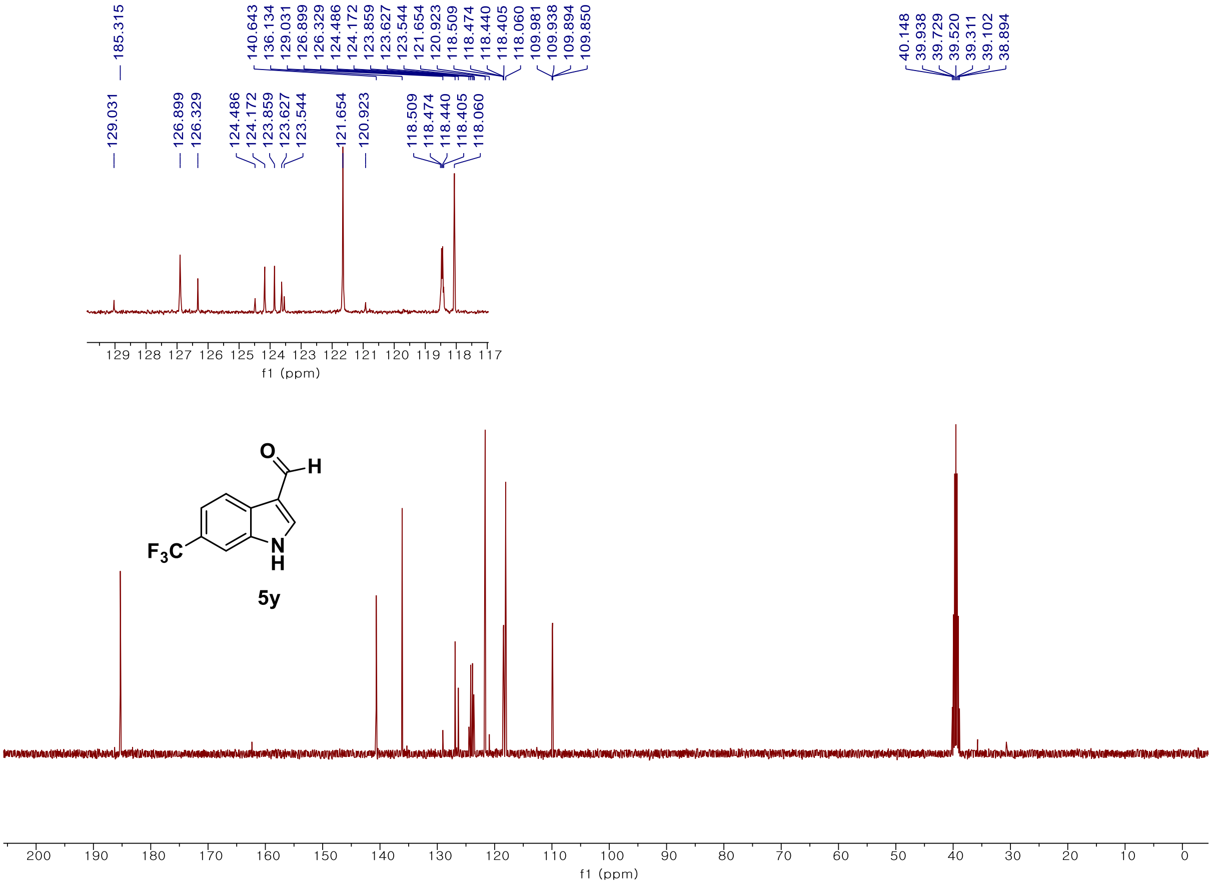
185.315
140.643
136.134
129.031
126.899
126.329
124.486
124.172
123.859
123.627
123.544
121.654
120.923
118.509
118.474
118.440
118.405
118.060
109.981
109.938
109.894
109.850
40.148
39.938
39.729
39.520
39.311
39.102
38.894
129.031
126.899
126.329
124.486
124.172
123.859
123.627
123.544
121.654
120.923
118.509
118.474
118.440
118.405
118.060
129 128 127 126 125 124 123 122 121 120 119 118 117
f1 (ppm)
O
H
F3C
N
H
5y
200 190 180 170 160 150 140 130 120 110 100 90 80 70 60 50 40 30 20 10 0
f1 (ppm)

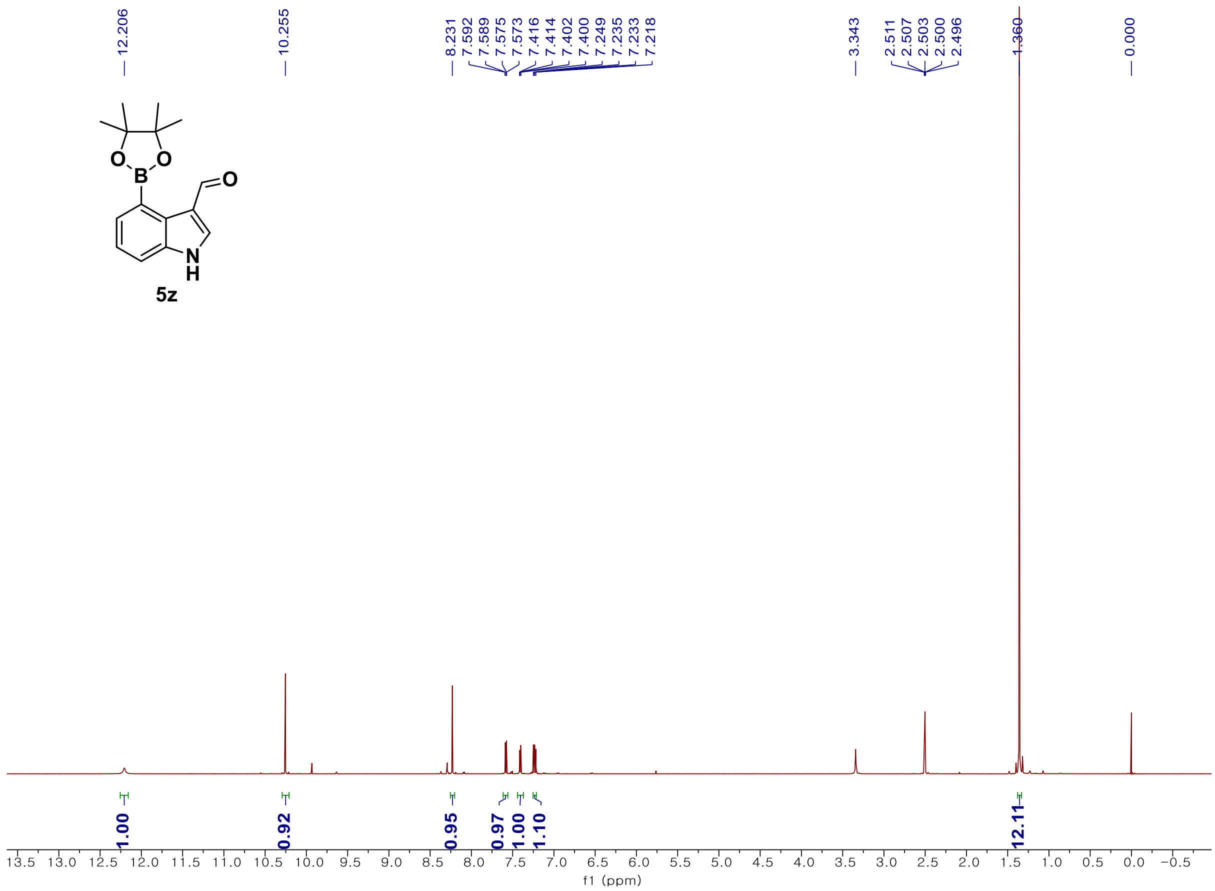
12.206
10.255
8.231
7.592
7.589
7.575
7.573
7.416
7.414
7.402
7.400
7.249
7.235
7.233
7.218
3.343
2.511
2.507
2.503
2.500
2.496
1.360
0.000
O
B
O
O
N
H
5z
1.00
0.92
0.95
0.97
1.00
1.10
12.11
13.5 13.0 12.5 12.0 11.5 11.0 10.5 10.0 9.5 9.0 8.5 8.0 7.5 7.0 6.5 6.0 5.5 5.0 4.5 4.0 3.5 3.0 2.5 2.0 1.5 1.0 0.5 0.0 -0.5
f1 (ppm)

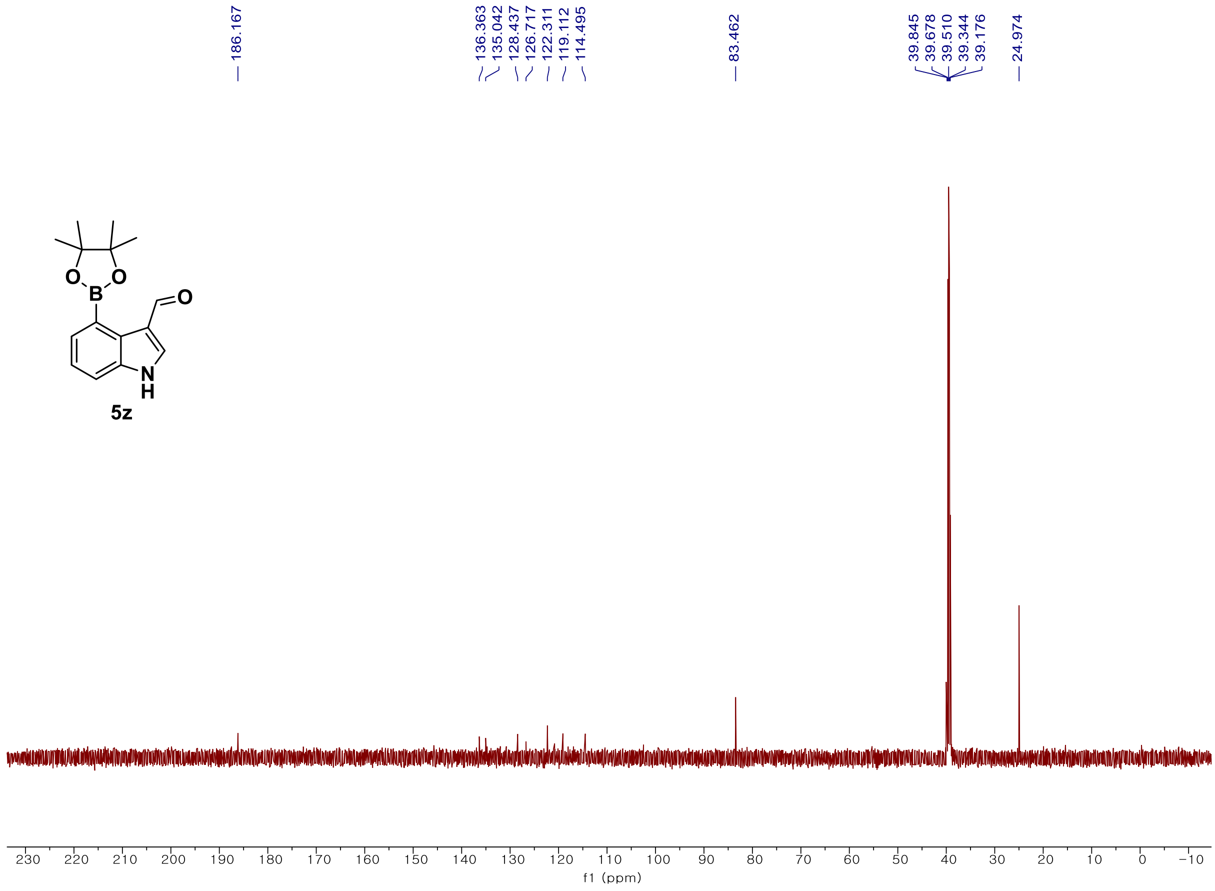
186.167
136.363
135.042
128.437
126.717
122.311
119.112
114.495
83.462
39.845
39.678
39.510
39.344
39.176
24.974
O
B
O
O
N
H
5z
230 220 210 200 190 180 170 160 150 140 130 120 110 100 90 80 70 60 50 40 30 20 10 0 -10
f1 (ppm)

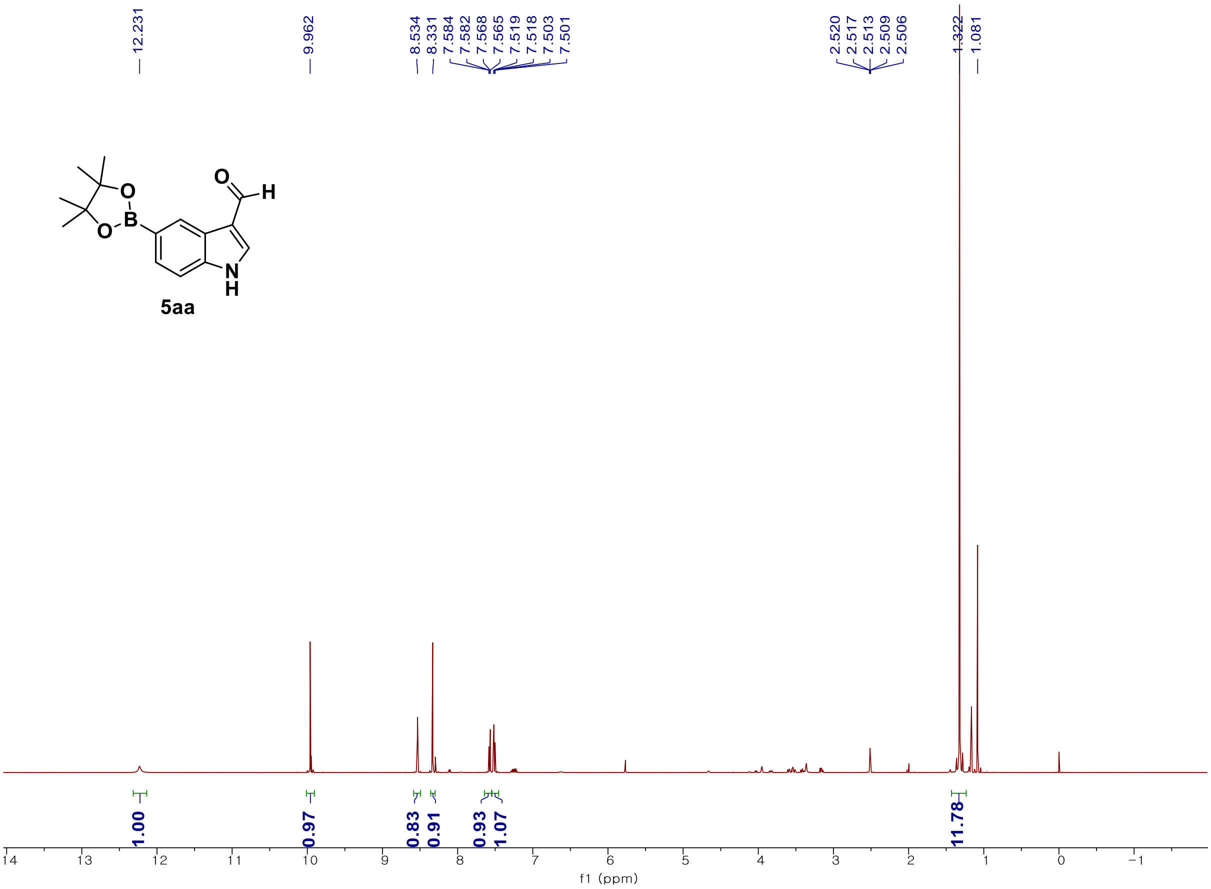

12.231
9.962
8.534
8.331
7.584
7.582
7.568
7.565
7.519
7.518
7.503
7.501
2.520
2.517
2.513
2.509
2.506
1.322
1.081
O
H
O
B
O
N
H
5aa
1.00
0.97
0.83
0.91
0.93
1.07
11.78
f1 (ppm)

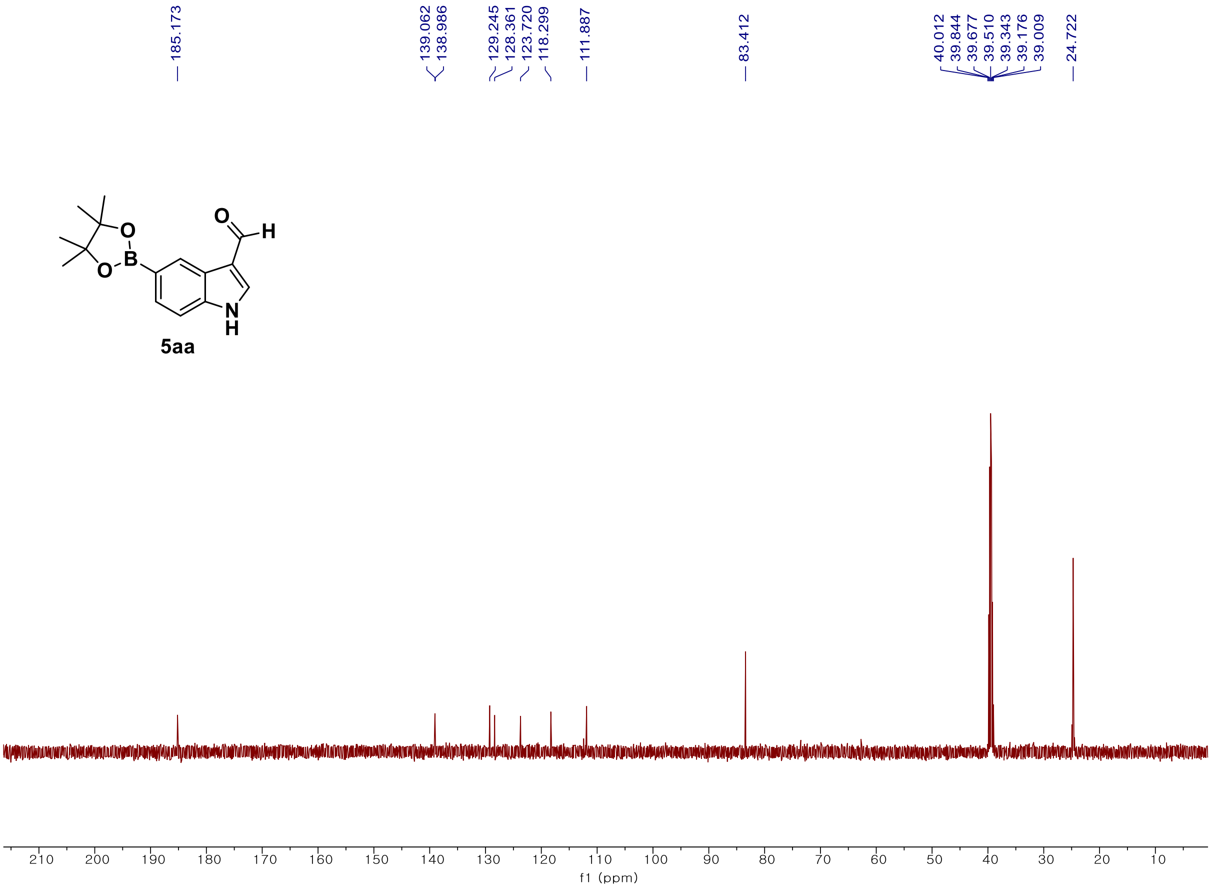

185.173
139.062
138.986
129.245
128.361
123.720
118.299
111.887
83.412
40.012
39.844
39.677
39.510
39.343
39.176
39.009
24.722
O
H
O
B
O
N
H
5aa
f1 (ppm)

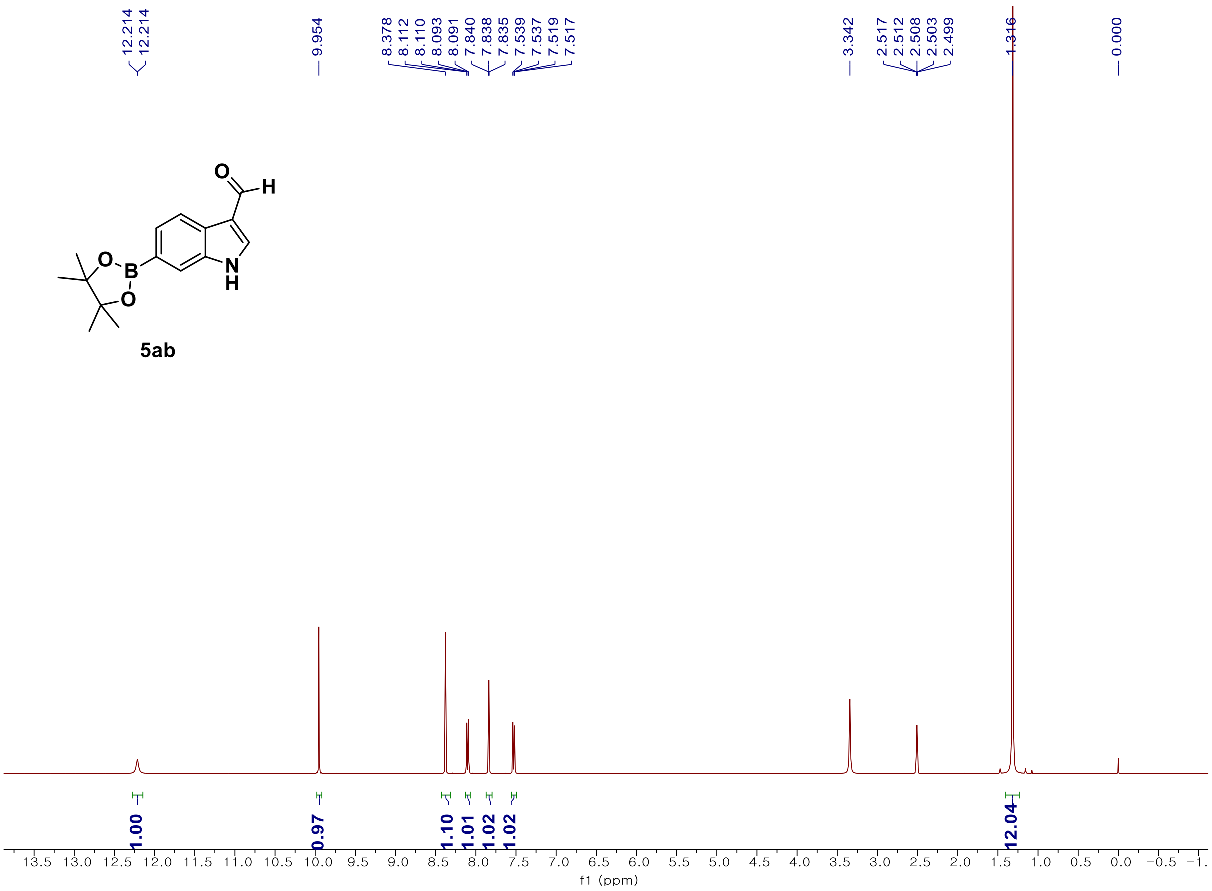
12.214
12.214
9.954
8.378
8.112
8.110
8.093
8.091
7.840
7.838
7.835
7.539
7.537
7.519
7.517
3.342
2.517
2.512
2.508
2.503
2.499
1.316
0.000
O
H
O
B
O
N
H
5ab
1.00
0.97
1.10
1.01
1.02
1.02
12.04
13.5 13.0 12.5 12.0 11.5 11.0 10.5 10.0 9.5 9.0 8.5 8.0 7.5 7.0 6.5 6.0 5.5 5.0 4.5 4.0 3.5 3.0 2.5 2.0 1.5 1.0 0.5 0.0 −0.5 −1.
f1 (ppm)

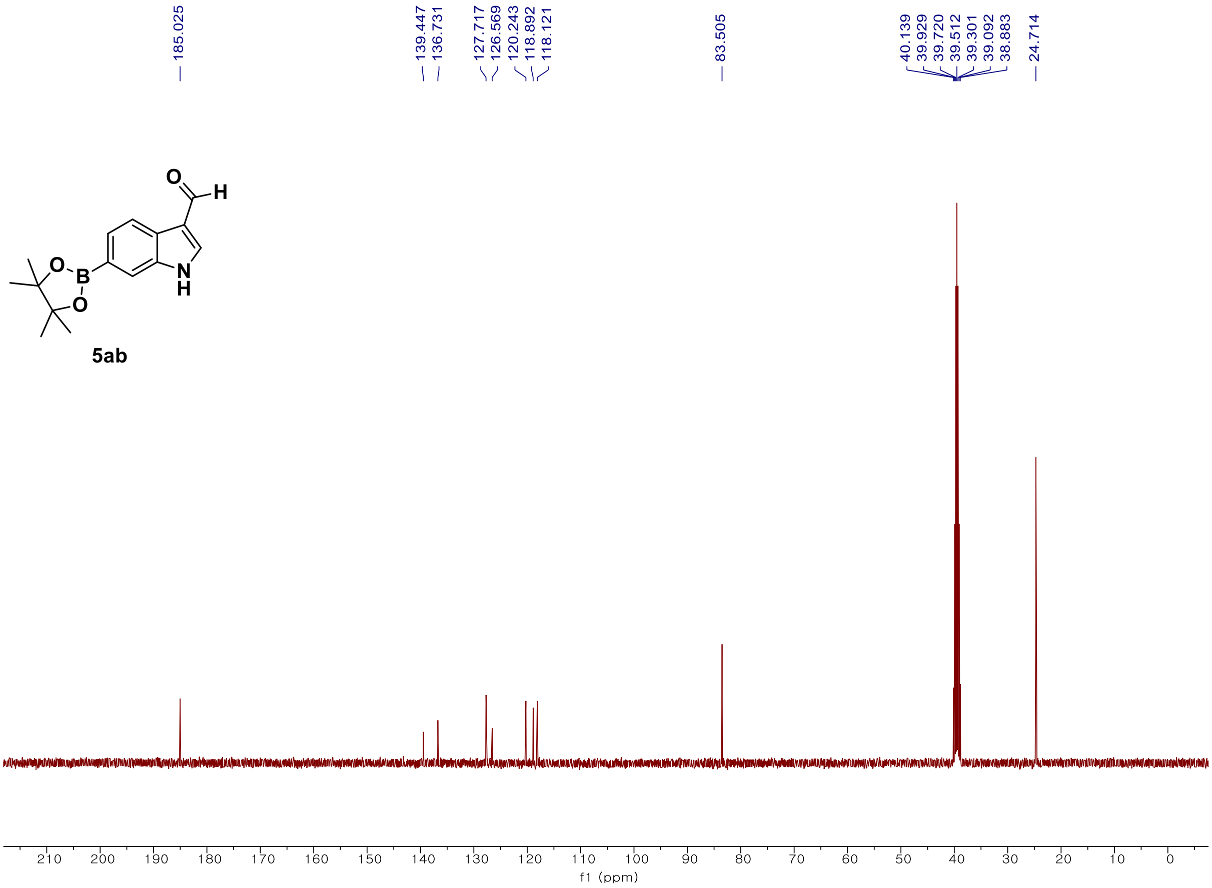
185.025
139.447
136.731
127.717
126.569
120.243
118.892
118.121
83.505
40.139
39.929
39.720
39.512
39.301
39.092
38.883
24.714
O
H
O
B
O
N
H
5ab
210 200 190 180 170 160 150 140 130 120 110 100 90 80 70 60 50 40 30 20 10 0
f1 (ppm)

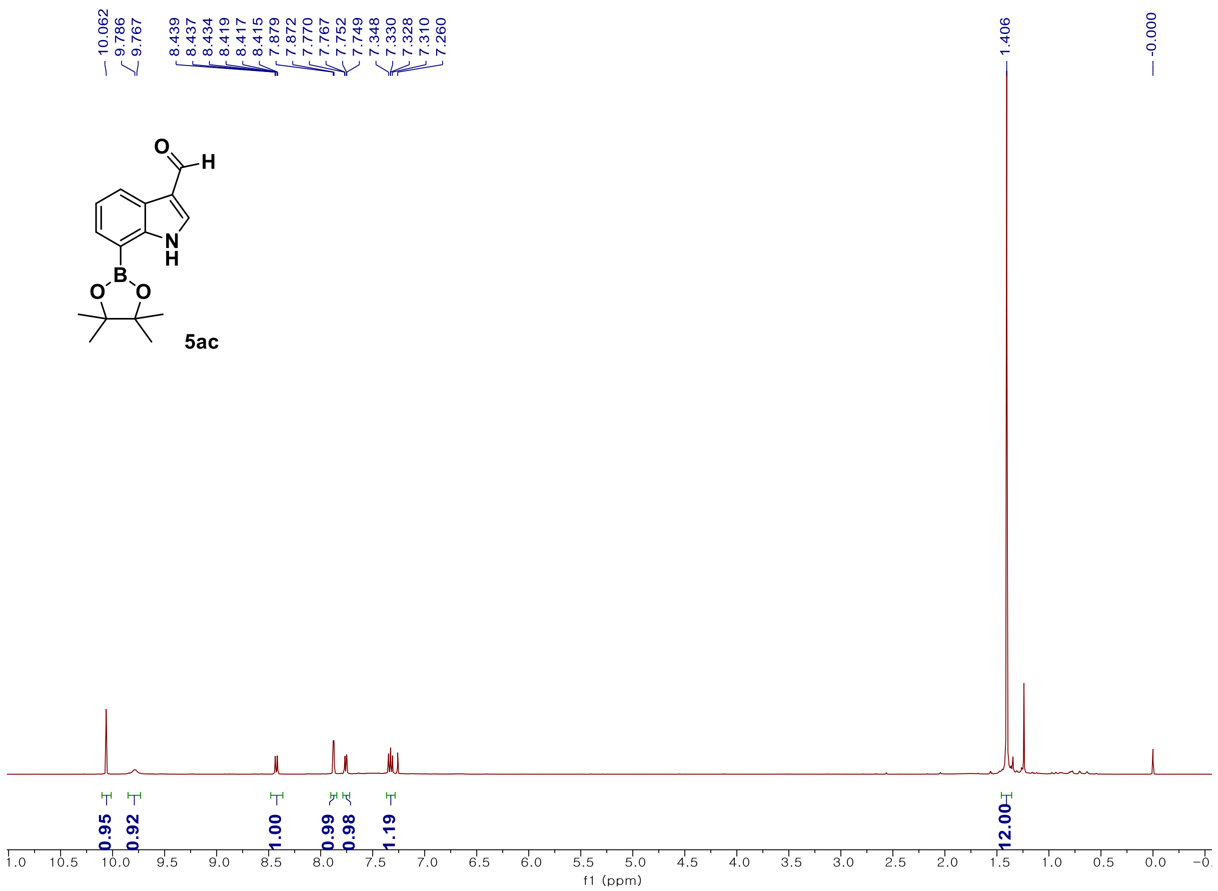
10.062
9.786
9.767
8.439
8.437
8.434
8.419
8.417
8.415
7.879
7.872
7.770
7.767
7.752
7.749
7.348
7.330
7.328
7.310
7.260
1.406
-0.000
5ac
0.95
0.92
1.00
0.99
0.98
1.19
12.00
f1 (ppm)

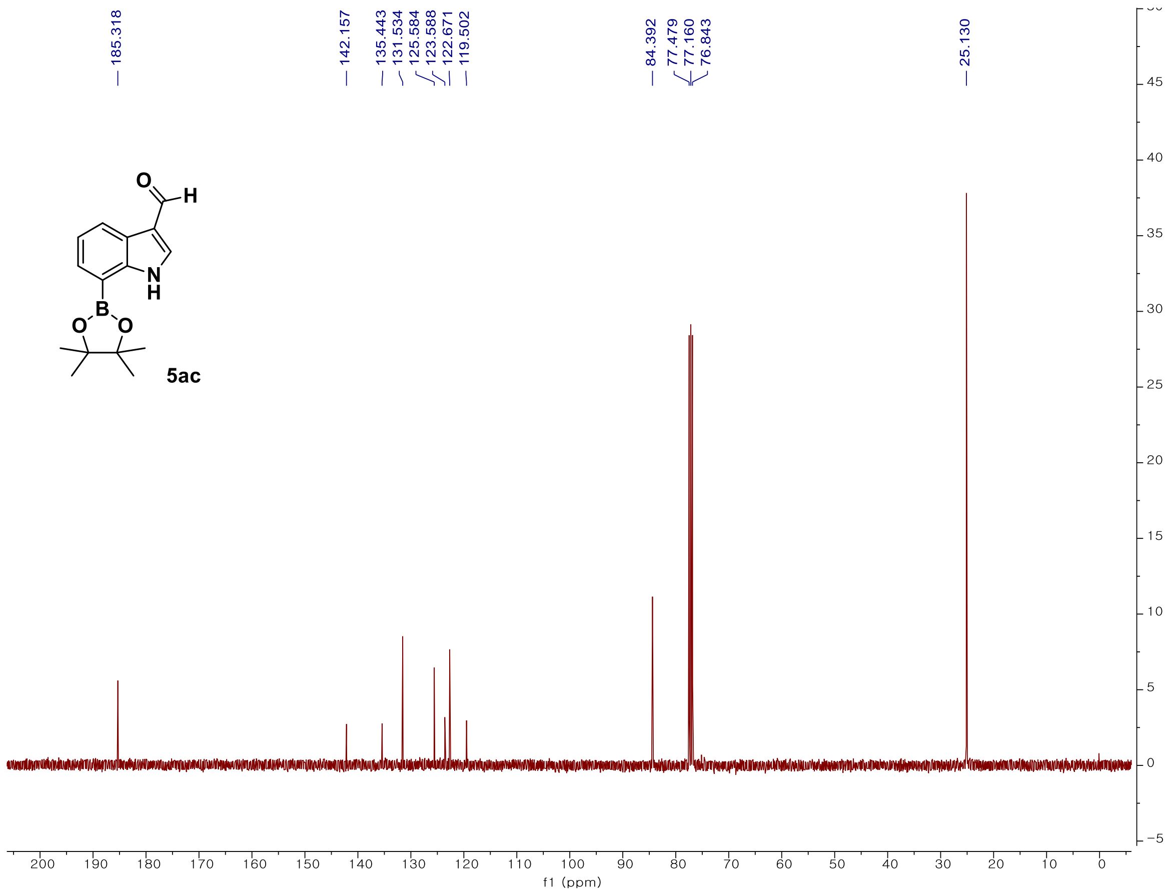
185.318
142.157
135.443
131.534
125.584
123.588
122.671
119.502
84.392
77.479
77.160
76.843
25.130
5ac
f1 (ppm)

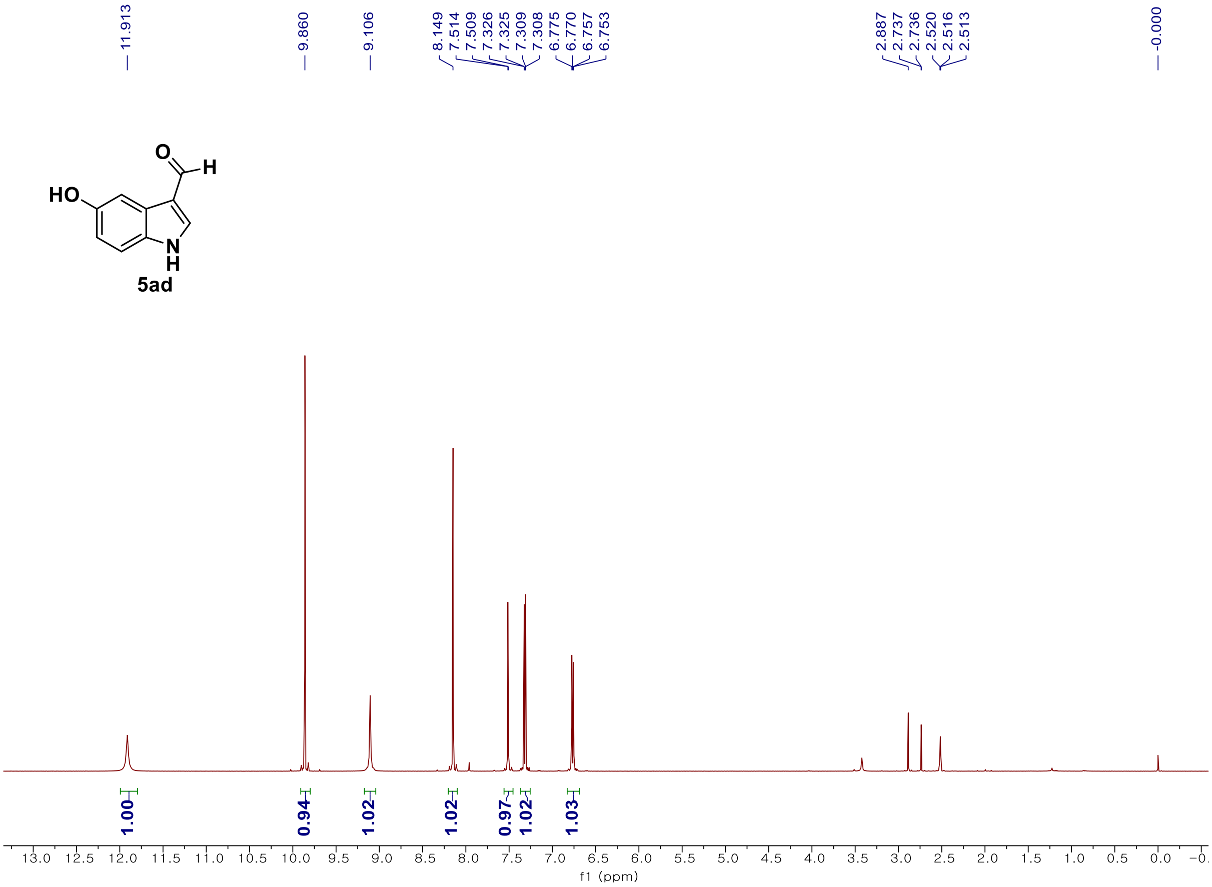
11.913
9.860
9.106
8.149
7.514
7.509
7.326
7.325
7.309
7.308
6.775
6.770
6.757
6.753
2.887
2.737
2.736
2.520
2.516
2.513
-0.000
HO
O
H
N
H
5ad
1.00
0.94
1.02
1.02
0.97
1.02
1.03
f1 (ppm)

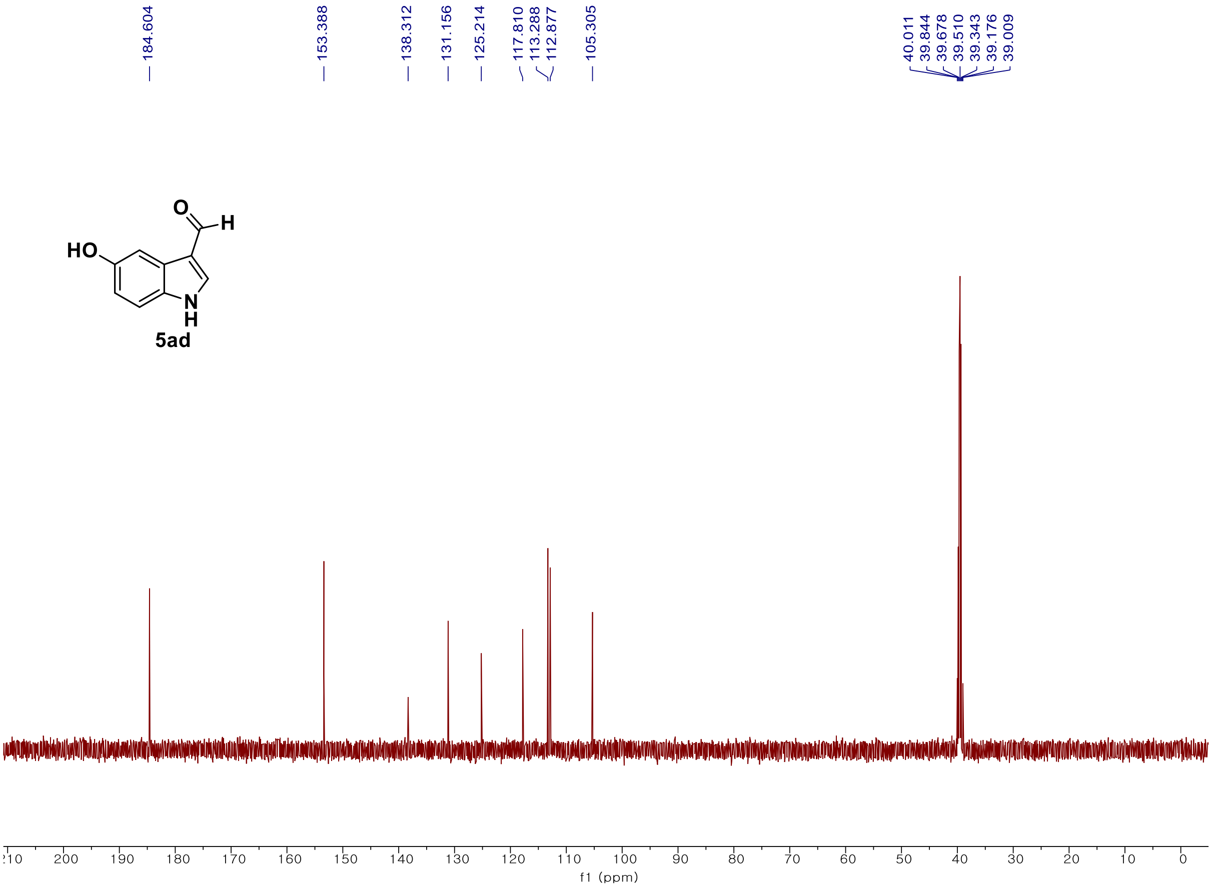
184.604
153.388
138.312
131.156
125.214
117.810
113.288
112.877
105.305
40.011
39.844
39.678
39.510
39.343
39.176
39.009
HO
O
H
N
H
5ad
f1 (ppm)

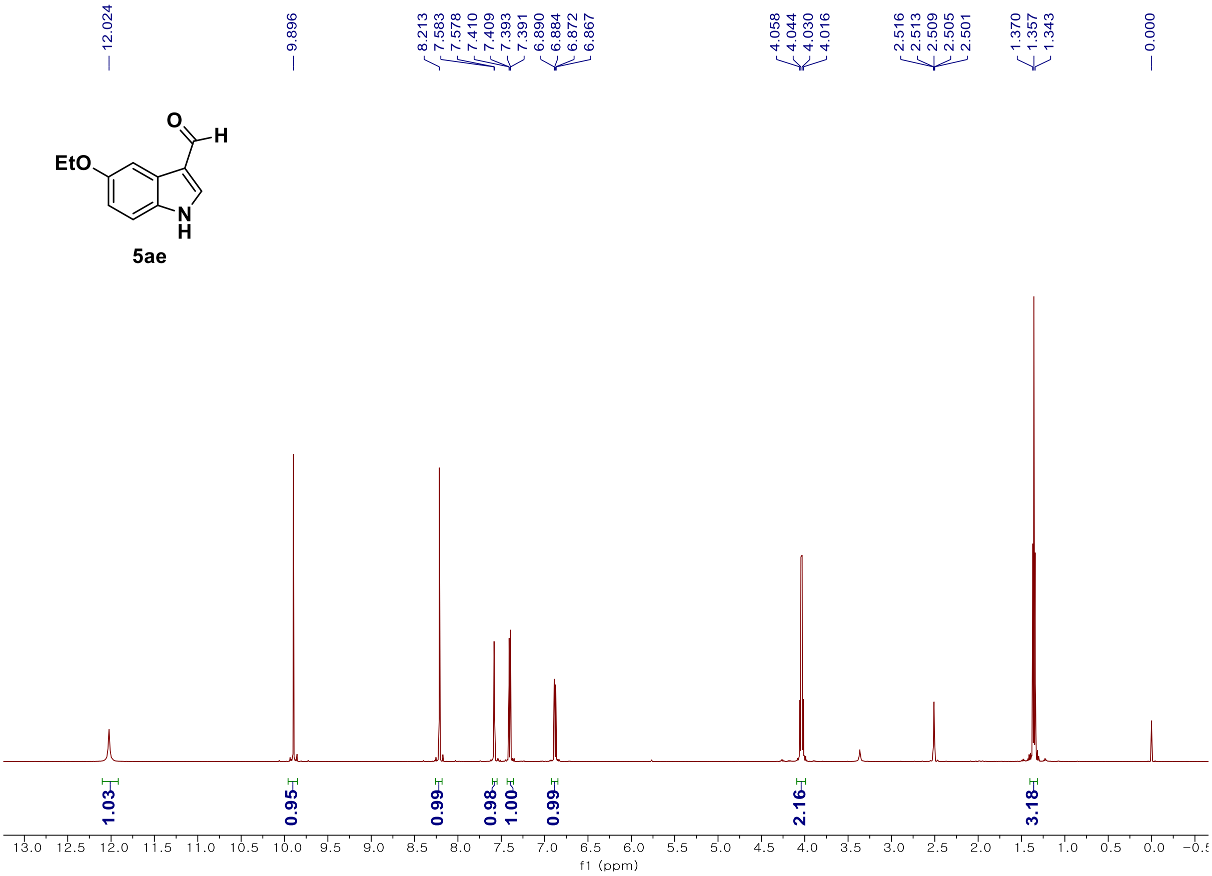
12.024
9.896
8.213
7.583
7.578
7.410
7.409
7.393
7.391
6.890
6.884
6.872
6.867
4.058
4.044
4.030
4.016
2.516
2.513
2.509
2.505
2.501
1.370
1.357
1.343
0.000
EtO
O
H
N
H
5ae
1.03
0.95
0.99
0.98
1.00
0.99
2.16
3.18
f1 (ppm)

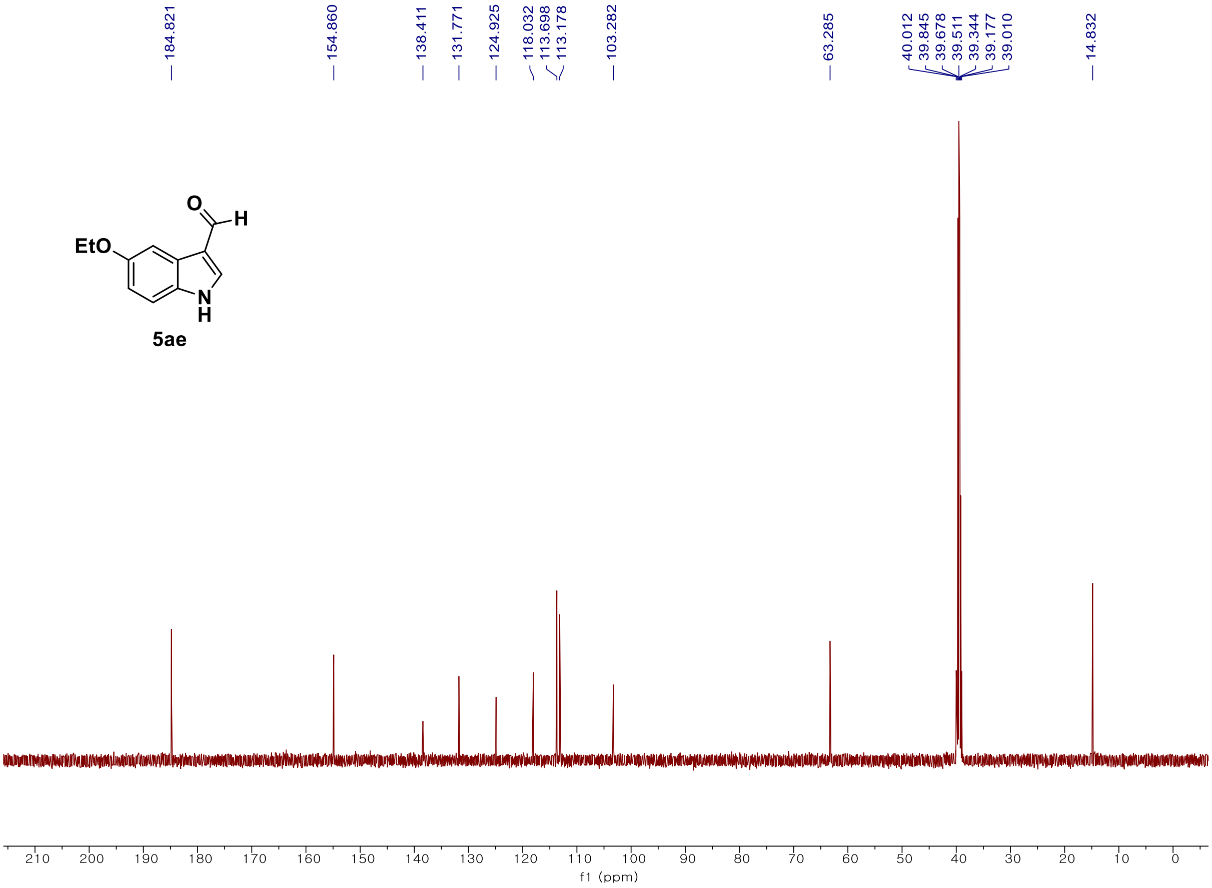
184.821
154.860
138.411
131.771
124.925
118.032
113.698
113.178
103.282
63.285
40.012
39.845
39.678
39.511
39.344
39.177
39.010
14.832
EtO
O
H
N
H
5ae
f1 (ppm)

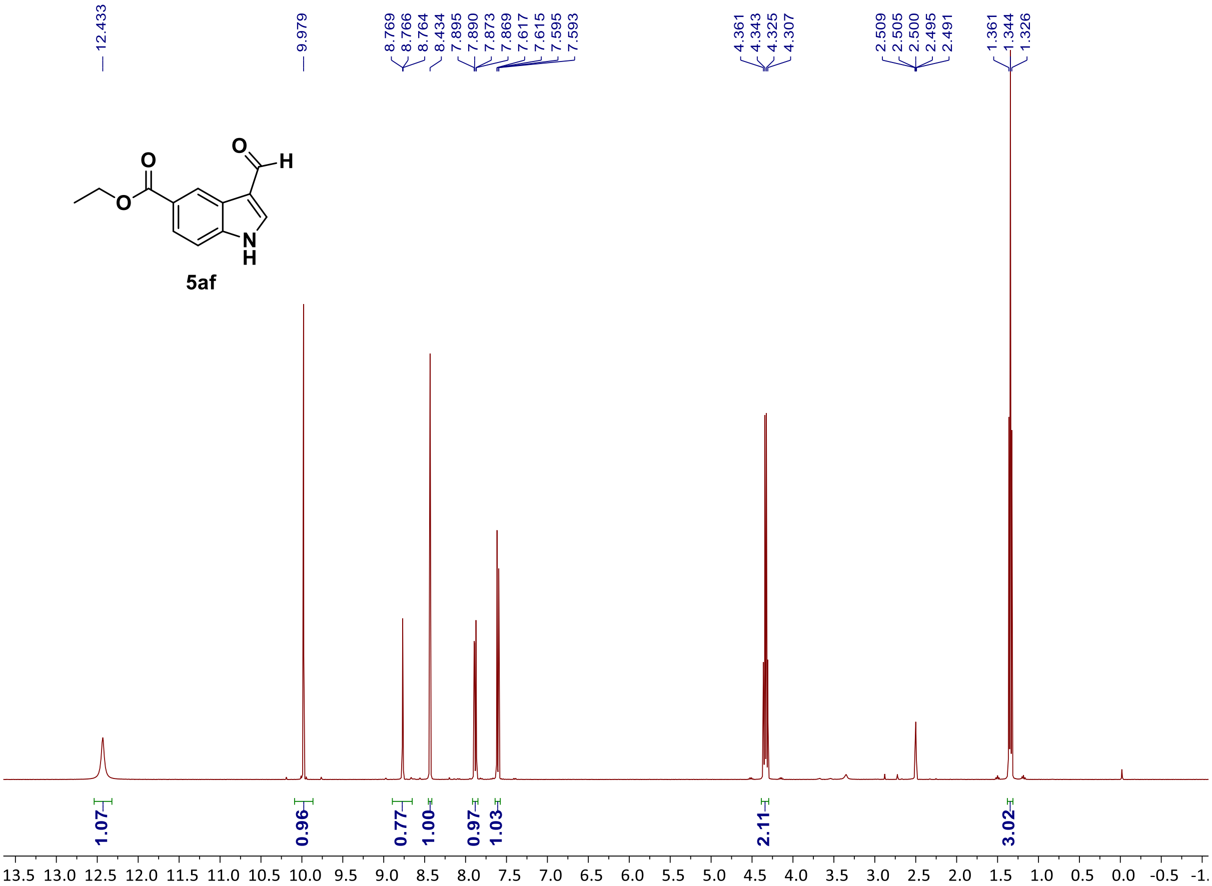
12.433
9.979
8.769
8.766
8.764
8.434
7.895
7.890
7.873
7.869
7.617
7.615
7.595
7.593
4.361
4.343
4.325
4.307
2.509
2.505
2.500
2.495
2.491
1.361
1.344
1.326
O
H
O
N
H
5af
1.07
0.96
0.77
1.00
0.97
1.03
2.11
3.02
13.5 13.0 12.5 12.0 11.5 11.0 10.5 10.0 9.5 9.0 8.5 8.0 7.5 7.0 6.5 6.0 5.5 5.0 4.5 4.0 3.5 3.0 2.5 2.0 1.5 1.0 0.5 0.0 -0.5 -1.

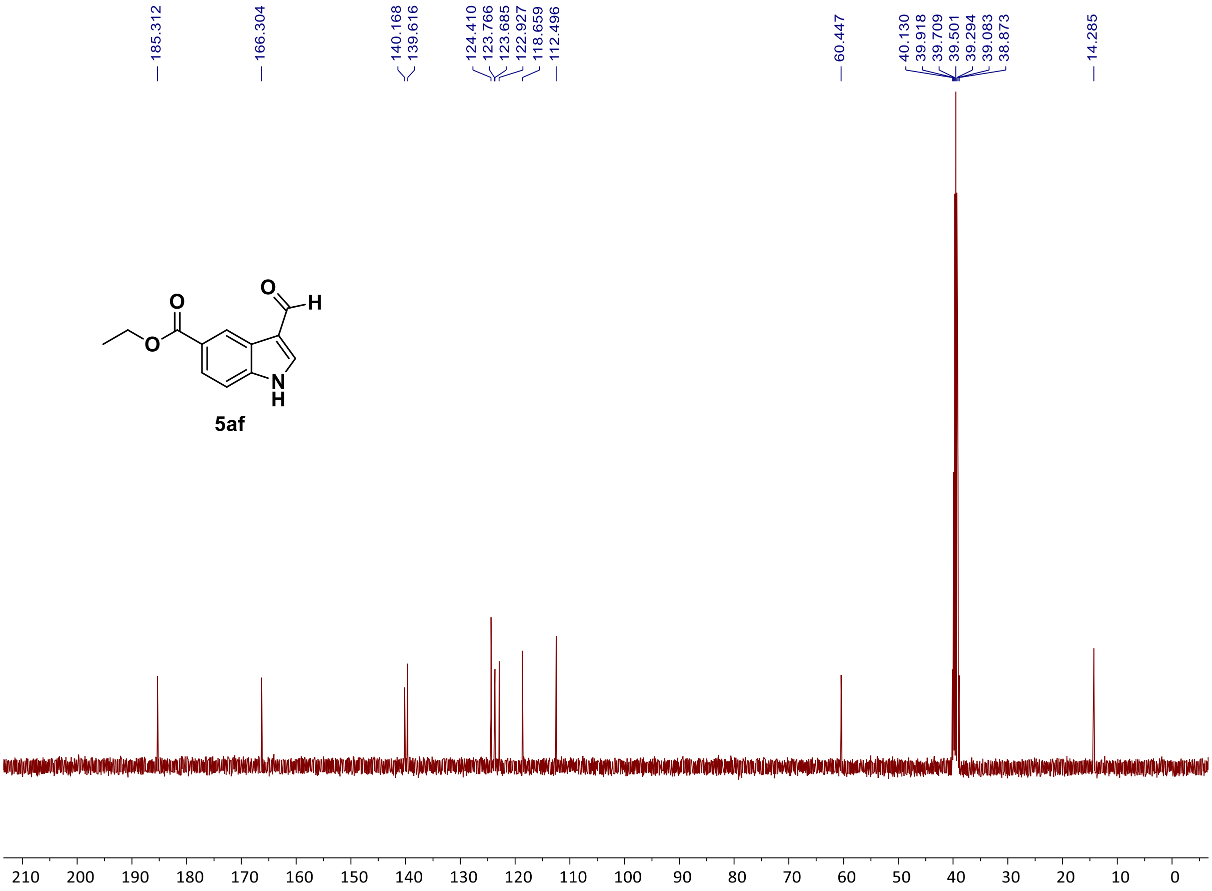
185.312
166.304
140.168
139.616
124.410
123.766
123.685
122.927
118.659
112.496
60.447
40.130
39.918
39.709
39.501
39.294
39.083
38.873
14.285
O
H
O
N
H
5af
210 200 190 180 170 160 150 140 130 120 110 100 90 80 70 60 50 40 30 20 10 0

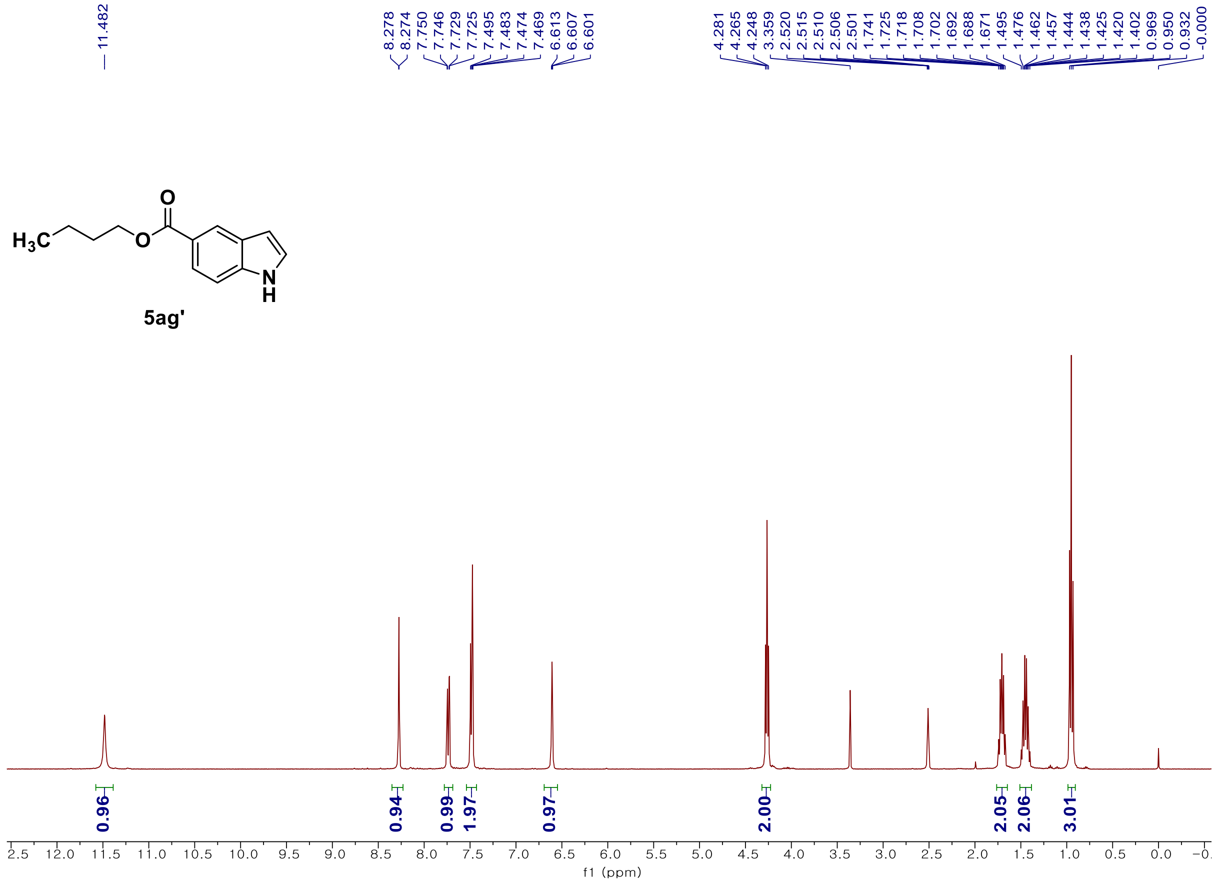
11.482
8.278
8.274
7.750
7.746
7.729
7.725
7.495
7.483
7.474
7.469
6.613
6.607
6.601
4.281
4.265
4.248
3.359
2.520
2.515
2.510
2.506
2.501
1.741
1.725
1.718
1.708
1.702
1.692
1.688
1.671
1.495
1.476
1.462
1.457
1.444
1.438
1.425
1.420
1.402
0.969
0.950
0.932
-0.000
H3C
O
O
N
H
5ag'
0.96
0.94
0.99
1.97
0.97
2.00
2.05
2.06
3.01
f1 (ppm)

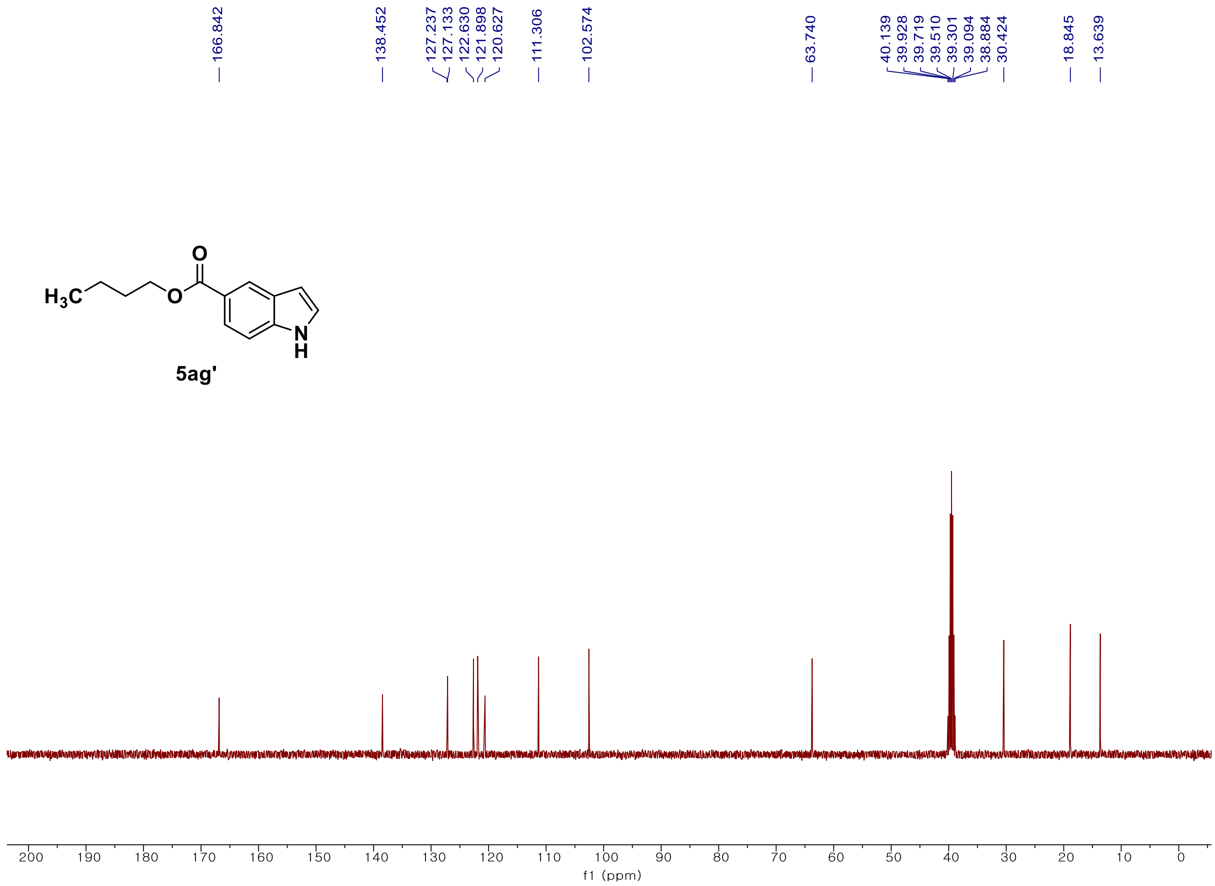
166.842
138.452
127.237
127.133
122.630
121.898
120.627
111.306
102.574
63.740
40.139
39.928
39.719
39.510
39.301
39.094
38.884
30.424
18.845
13.639
H3C
O
O
N
H
5ag'
f1 (ppm)

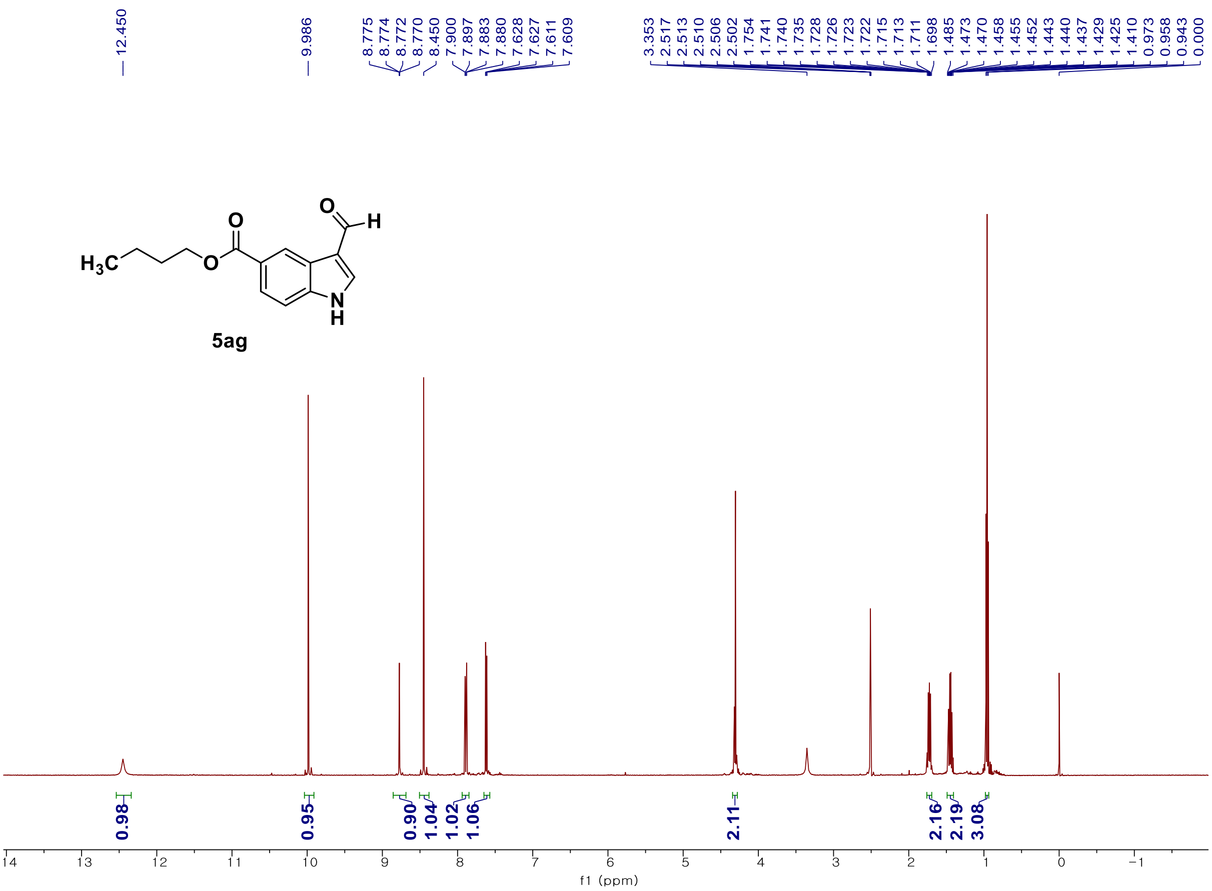
12.450
9.986
8.775
8.774
8.772
8.770
8.450
7.900
7.897
7.883
7.880
7.628
7.627
7.611
7.609
3.353
2.517
2.513
2.510
2.506
2.502
1.754
1.741
1.740
1.735
1.728
1.726
1.723
1.722
1.715
1.713
1.711
1.698
1.485
1.473
1.470
1.458
1.455
1.452
1.443
1.440
1.437
1.429
1.425
1.410
0.973
0.958
0.943
0.000
5ag
0.98
0.95
0.90
1.04
1.02
1.06
2.11
2.16
2.19
3.08
f1 (ppm)

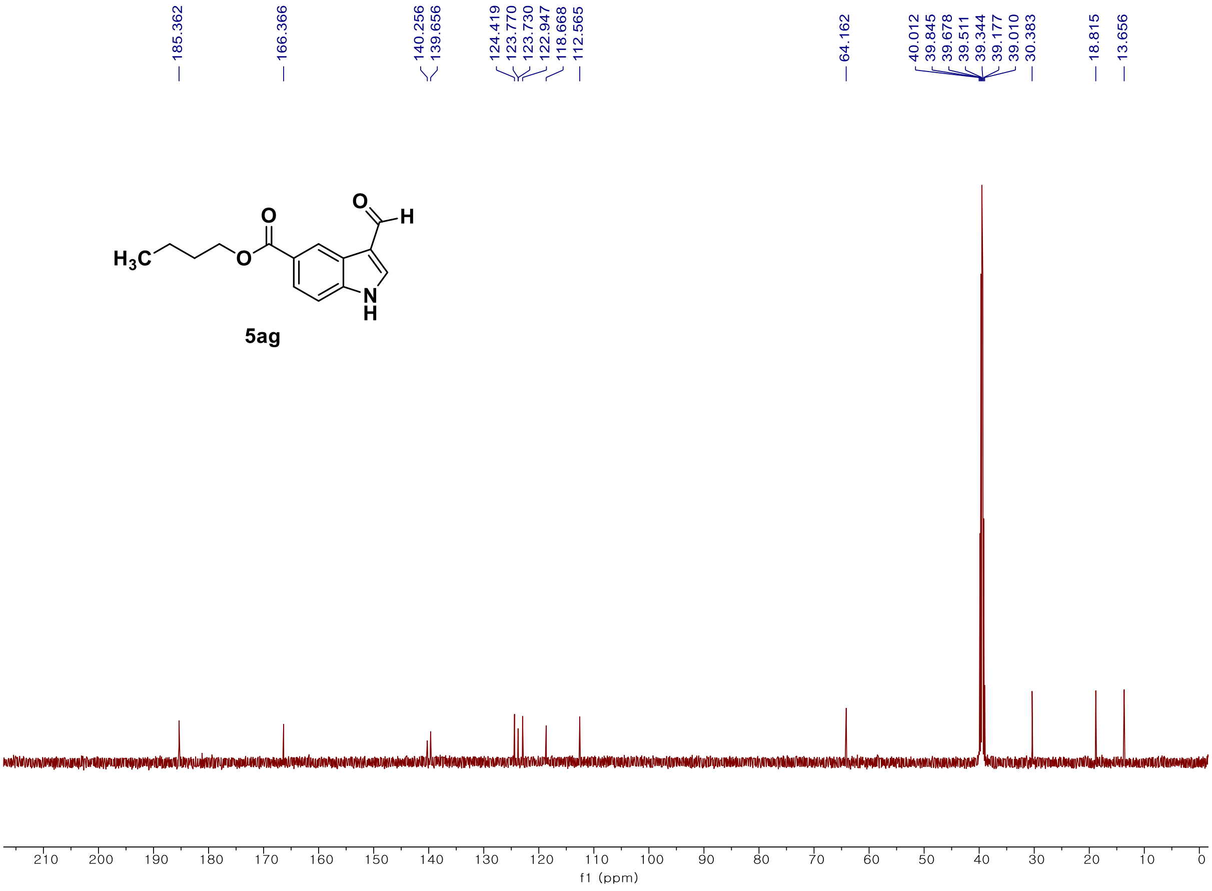
185.362
166.366
140.256
139.656
124.419
123.770
123.730
122.947
118.668
112.565
64.162
40.012
39.845
39.678
39.511
39.344
39.177
39.010
30.383
18.815
13.656
5ag
f1 (ppm)

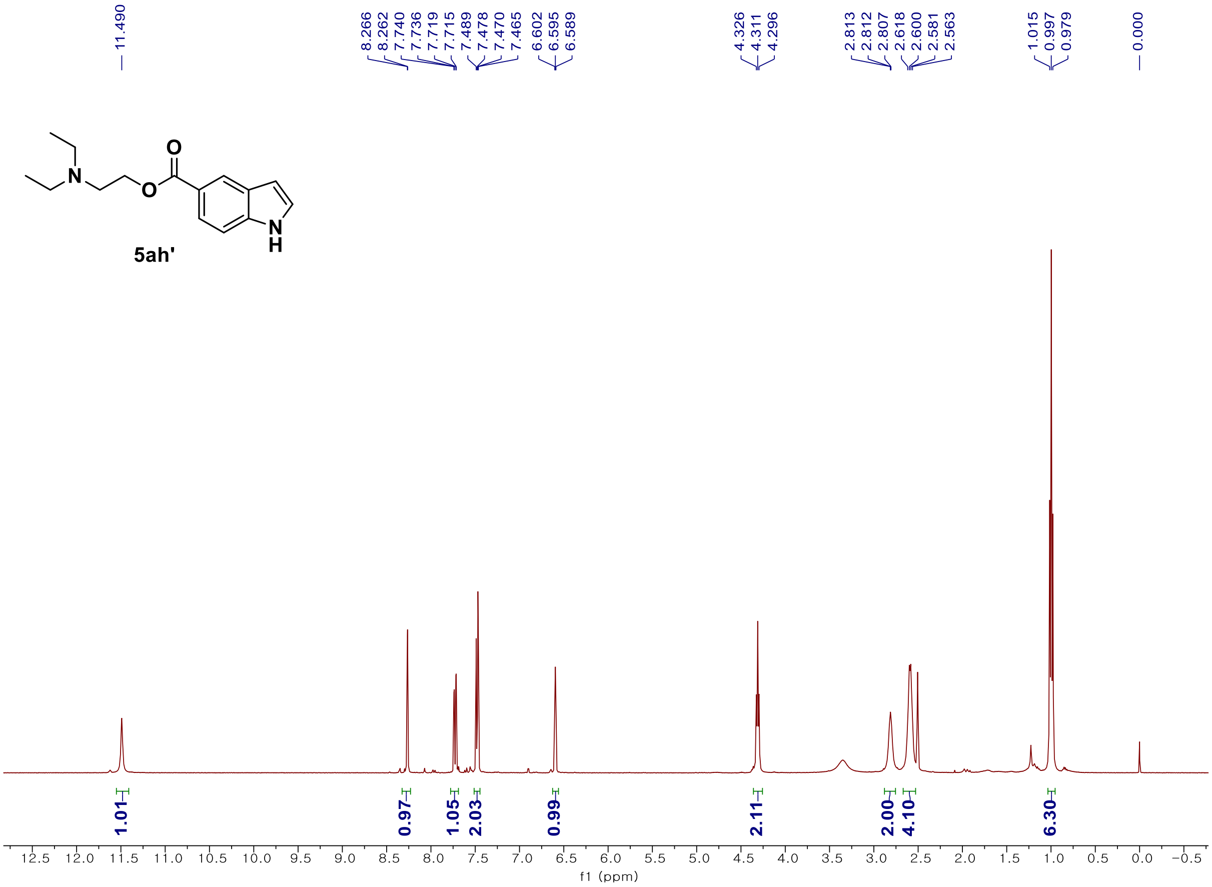
11.490
8.266
8.262
7.740
7.736
7.719
7.715
7.489
7.478
7.470
7.465
6.602
6.595
6.589
4.326
4.311
4.296
2.813
2.812
2.807
2.618
2.600
2.581
2.563
1.015
0.997
0.979
0.000
O
N
O
N
H
5ah'
1.01
0.97
1.05
2.03
0.99
2.11
2.00
4.10
6.30
f1 (ppm)

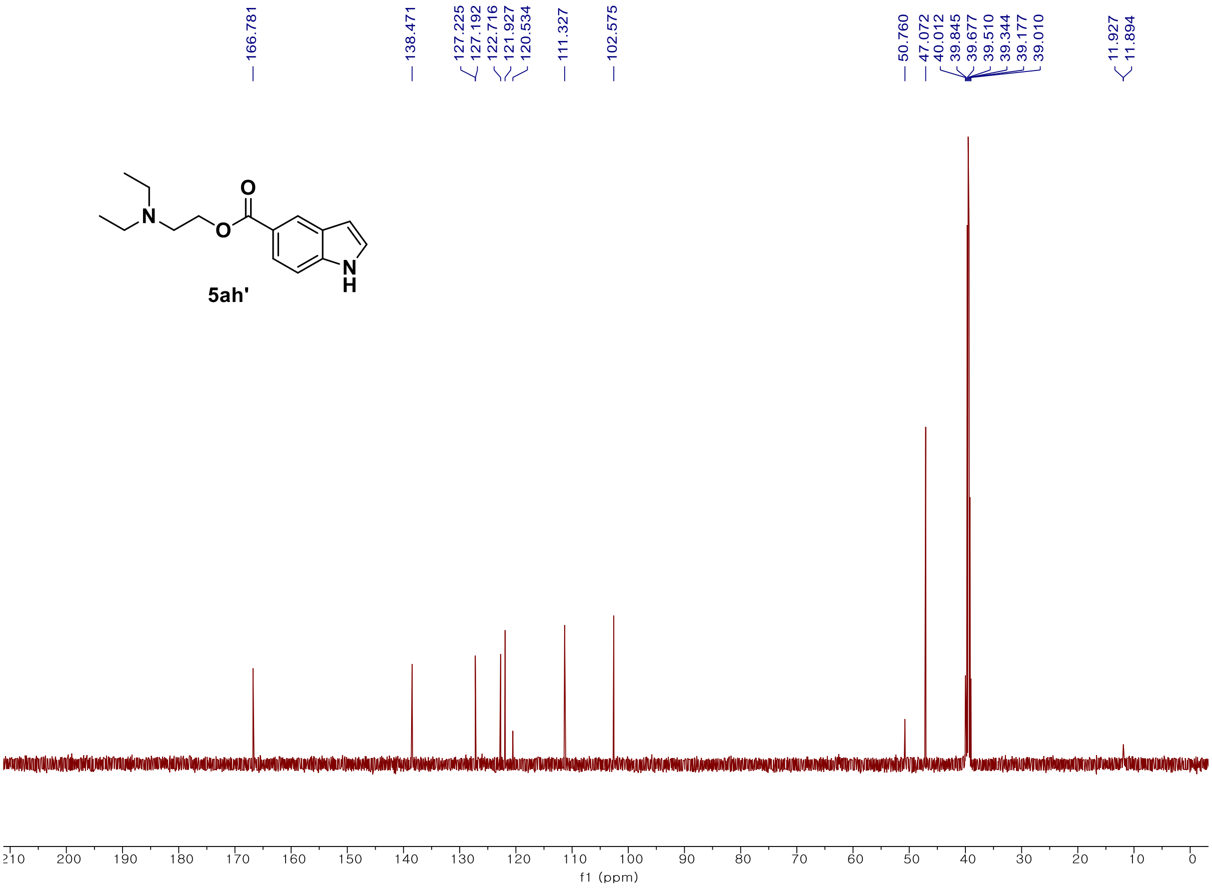
166.781
138.471
127.225
127.192
122.716
121.927
120.534
111.327
102.575
50.760
47.072
40.012
39.845
39.677
39.510
39.344
39.177
39.010
11.927
11.894
O
N
O
N
H
5ah'
f1 (ppm)

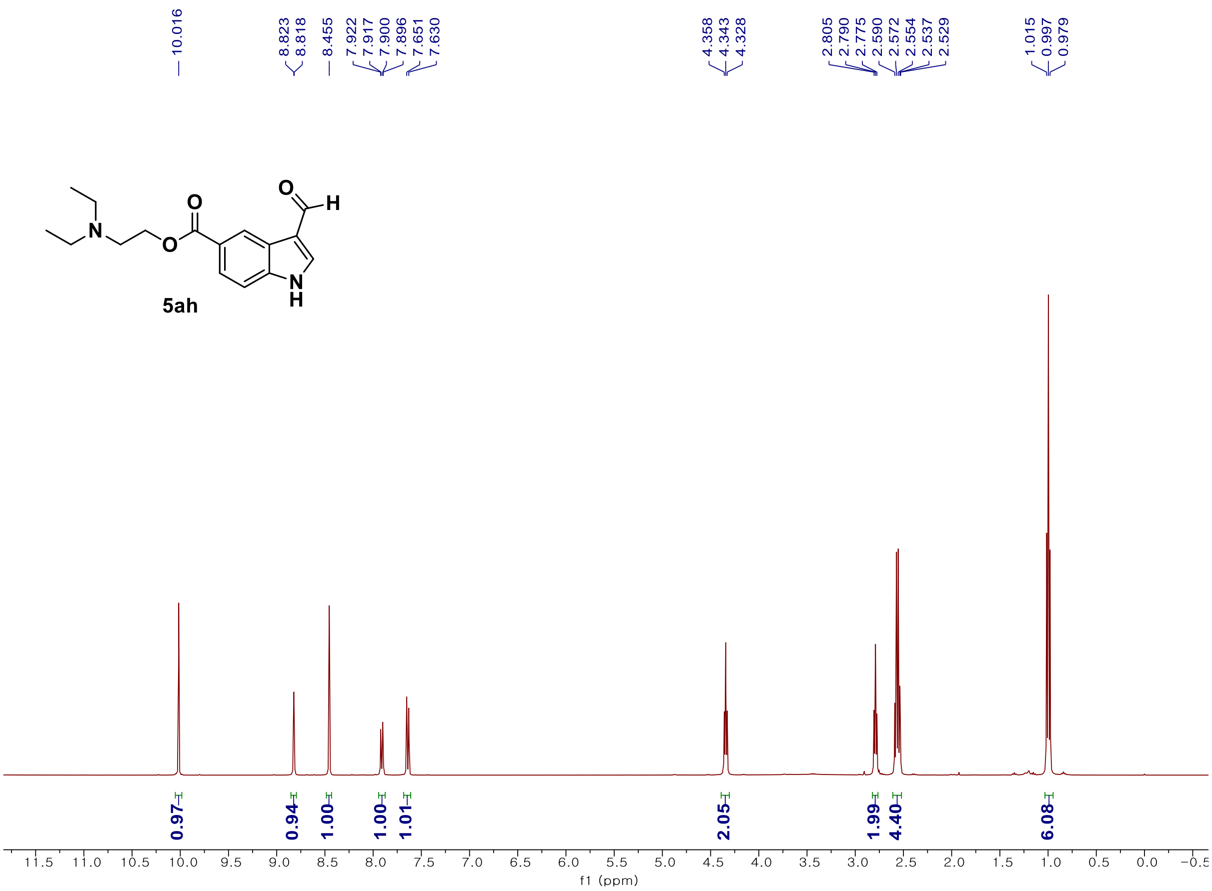
10.016
8.823
8.818
8.455
7.922
7.917
7.900
7.896
7.651
7.630
4.358
4.343
4.328
2.805
2.790
2.775
2.590
2.572
2.554
2.537
2.529
1.015
0.997
0.979
5ah
0.97
0.94
1.00
1.00
1.01
2.05
1.99
4.40
6.08
f1 (ppm)

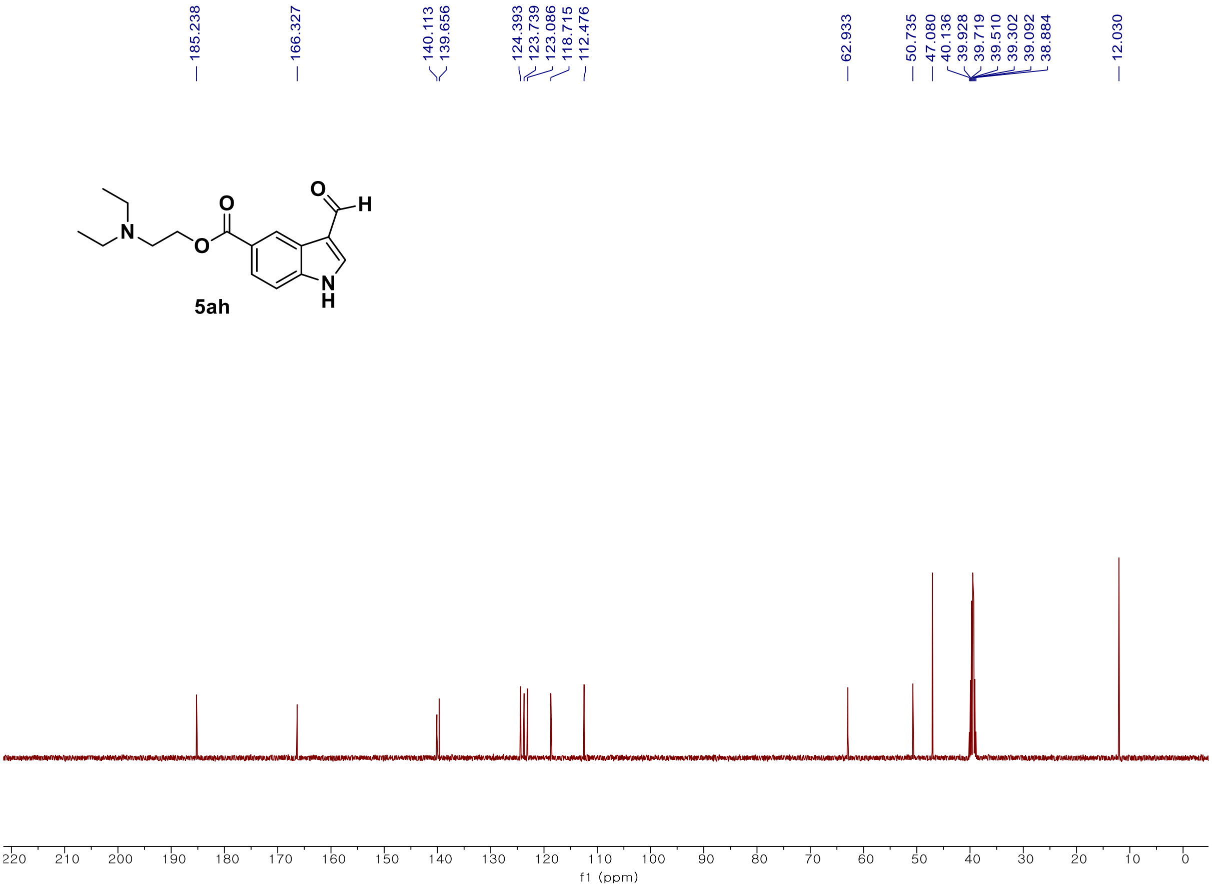
185.238
166.327
140.113
139.656
124.393
123.739
123.086
118.715
112.476
62.933
50.735
47.080
40.136
39.928
39.719
39.510
39.302
39.092
38.884
12.030
5ah
f1 (ppm)

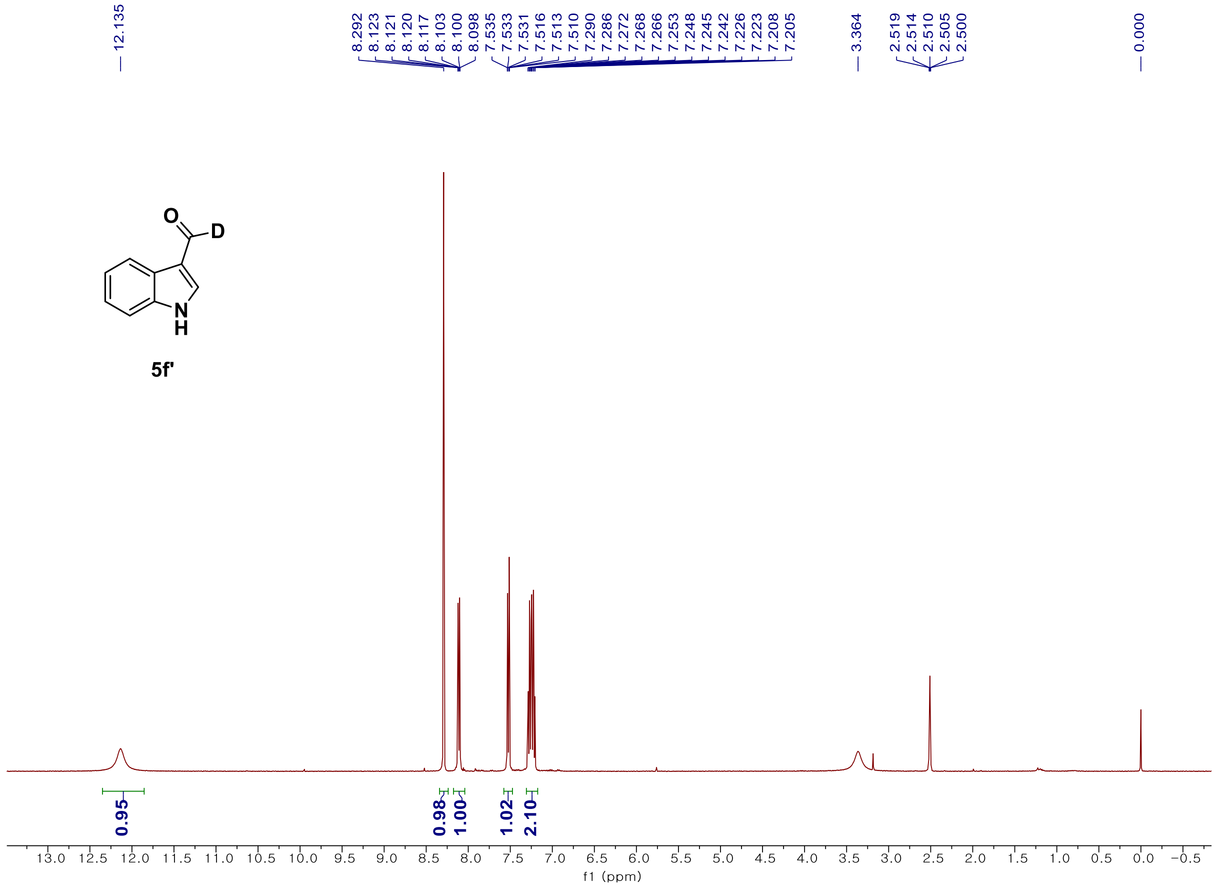
12.135
8.292
8.123
8.121
8.120
8.117
8.103
8.100
8.098
7.535
7.533
7.531
7.516
7.513
7.510
7.290
7.286
7.272
7.268
7.266
7.253
7.248
7.245
7.242
7.226
7.223
7.208
7.205
3.364
2.519
2.514
2.510
2.505
2.500
0.000
O
D
N
H
5f'
0.95
0.98
1.00
1.02
2.10
13.0 12.5 12.0 11.5 11.0 10.5 10.0 9.5 9.0 8.5 8.0 7.5 7.0 6.5 6.0 5.5 5.0 4.5 4.0 3.5 3.0 2.5 2.0 1.5 1.0 0.5 0.0 −0.5
f1 (ppm)

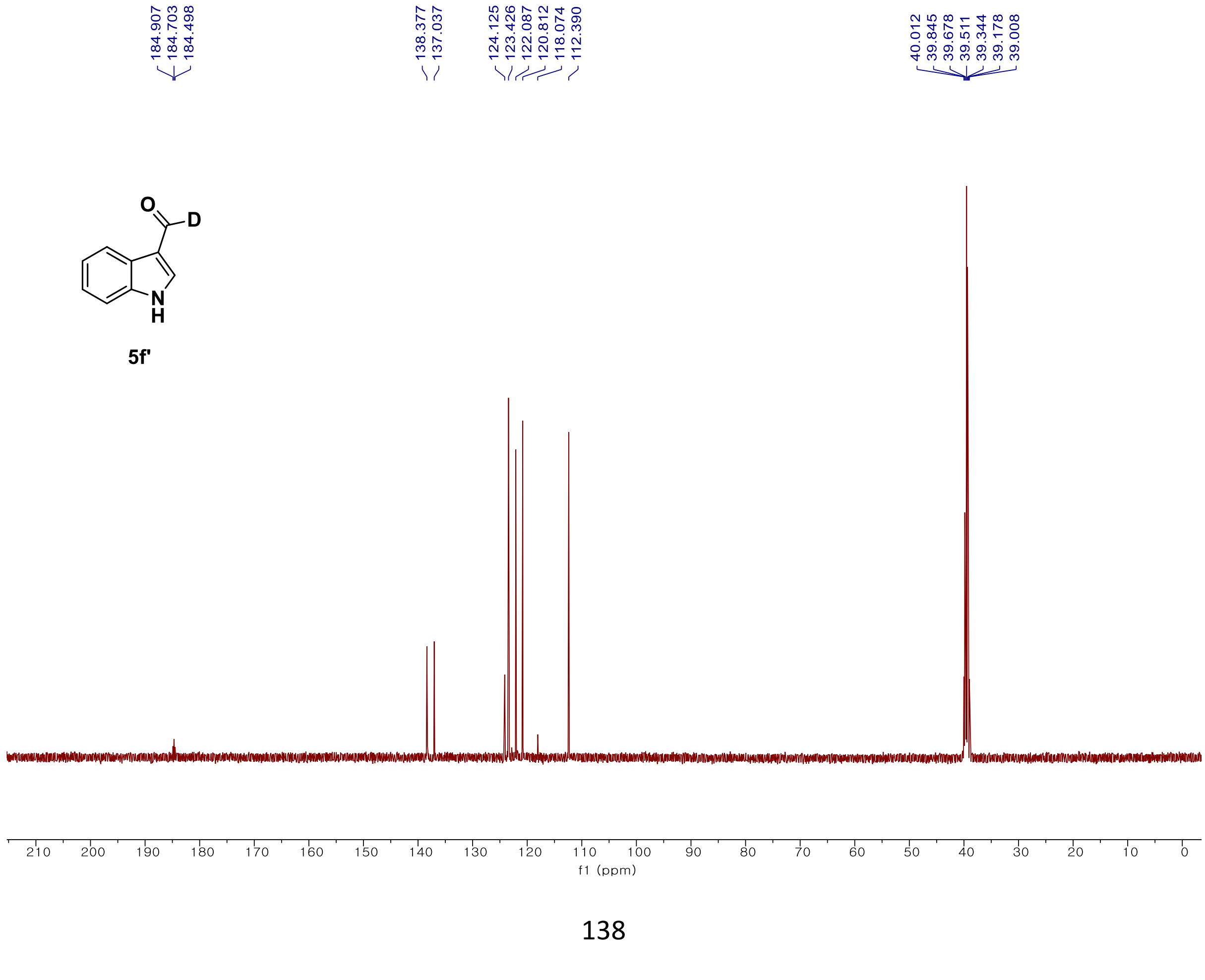
184.907
184.703
184.498
138.377
137.037
124.125
123.426
122.087
120.812
118.074
112.390
40.012
39.845
39.678
39.511
39.344
39.178
39.008
O
D
N
H
5f'
210 200 190 180 170 160 150 140 130 120 110 100 90 80 70 60 50 40 30 20 10 0
f1 (ppm)

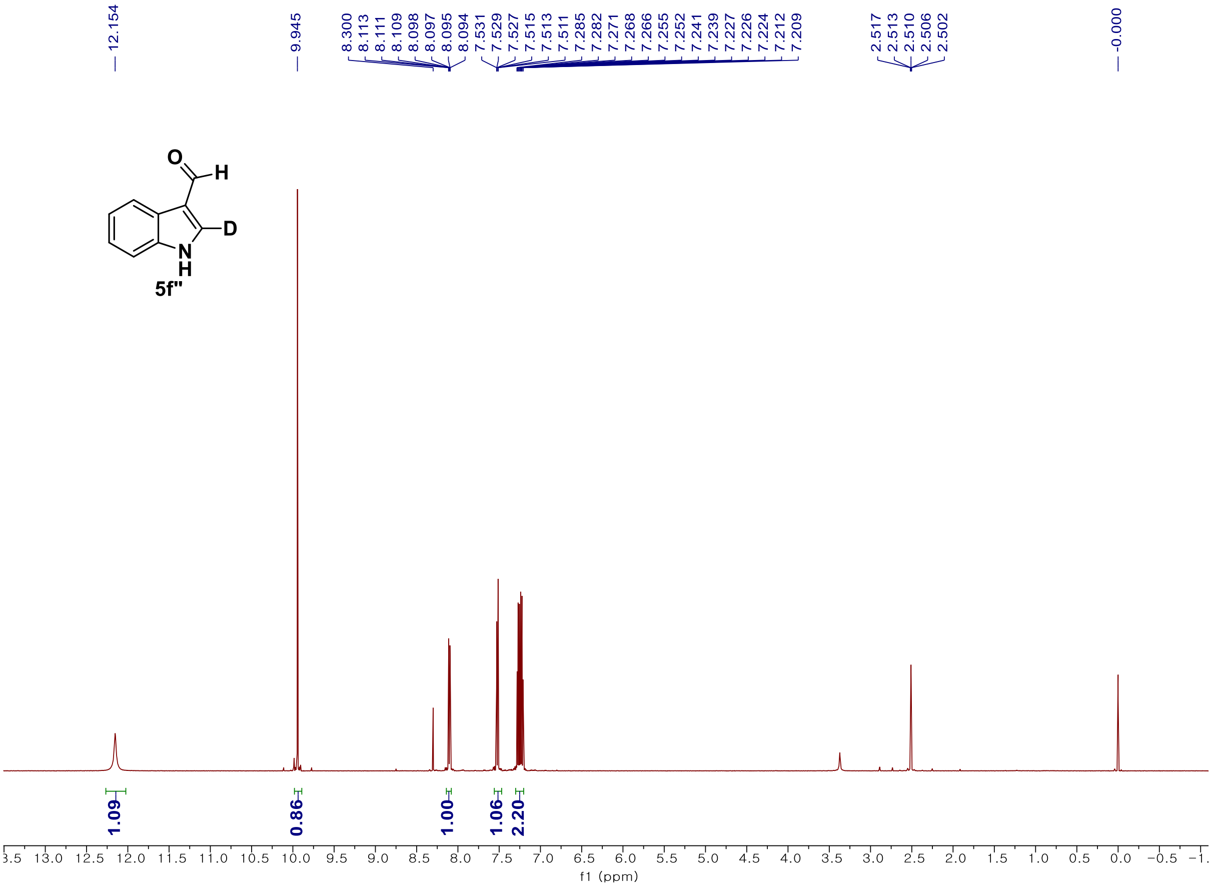
12.154
9.945
8.300
8.113
8.111
8.109
8.098
8.097
8.095
8.094
7.531
7.529
7.527
7.515
7.513
7.511
7.285
7.282
7.271
7.268
7.266
7.255
7.252
7.241
7.239
7.227
7.226
7.224
7.212
7.209
2.517
2.513
2.510
2.506
2.502
-0.000
O
H
D
N
H
5f''
1.09
0.86
1.00
1.06
2.20
f1 (ppm)

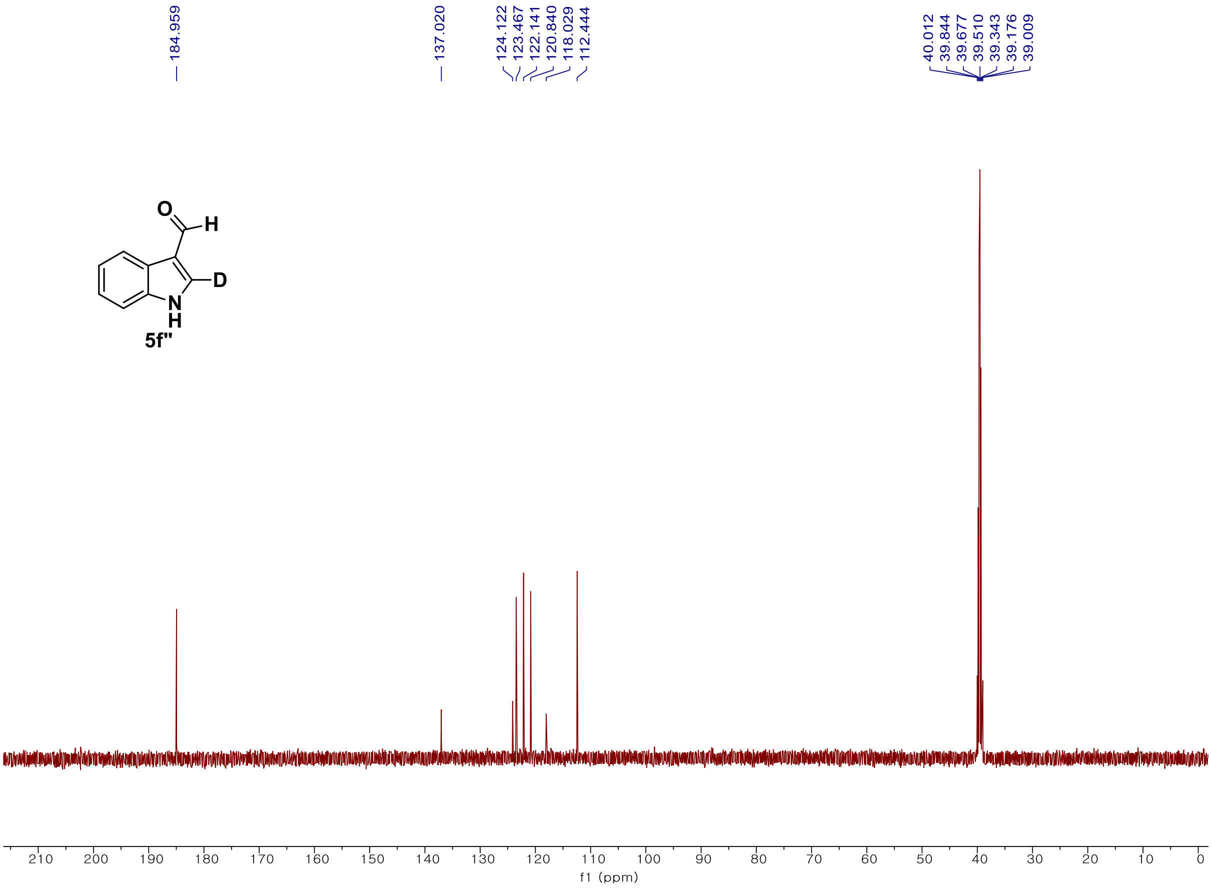
184.959
137.020
124.122
123.467
122.141
120.840
118.029
112.444
40.012
39.844
39.677
39.510
39.343
39.176
39.009
O
H
D
N
H
5f''
f1 (ppm)

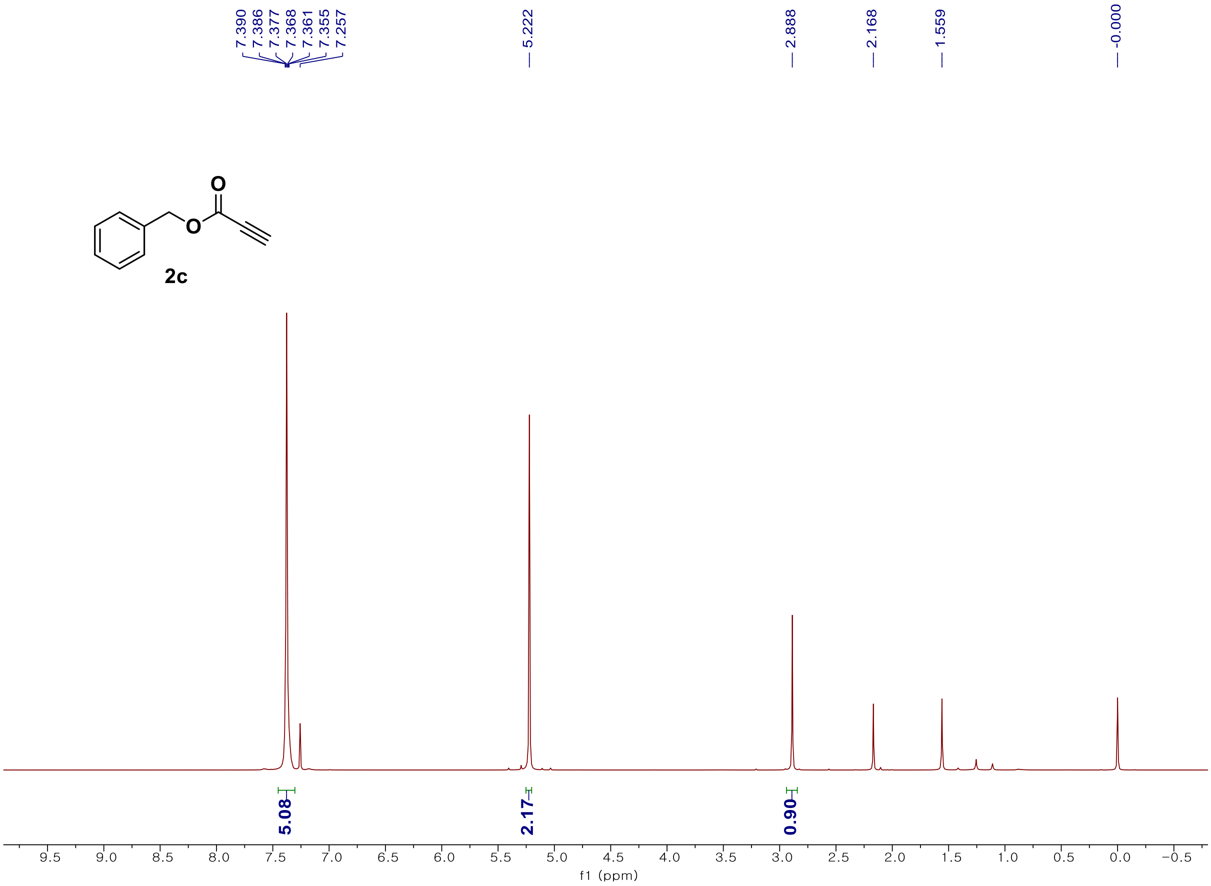
7.390
7.386
7.377
7.368
7.361
7.355
7.257
5.222
2.888
2.168
1.559
-0.000
2c
5.08
2.17
0.90
9.5 9.0 8.5 8.0 7.5 7.0 6.5 6.0 5.5 5.0 4.5 4.0 3.5 3.0 2.5 2.0 1.5 1.0 0.5 0.0 -0.5
f1 (ppm)

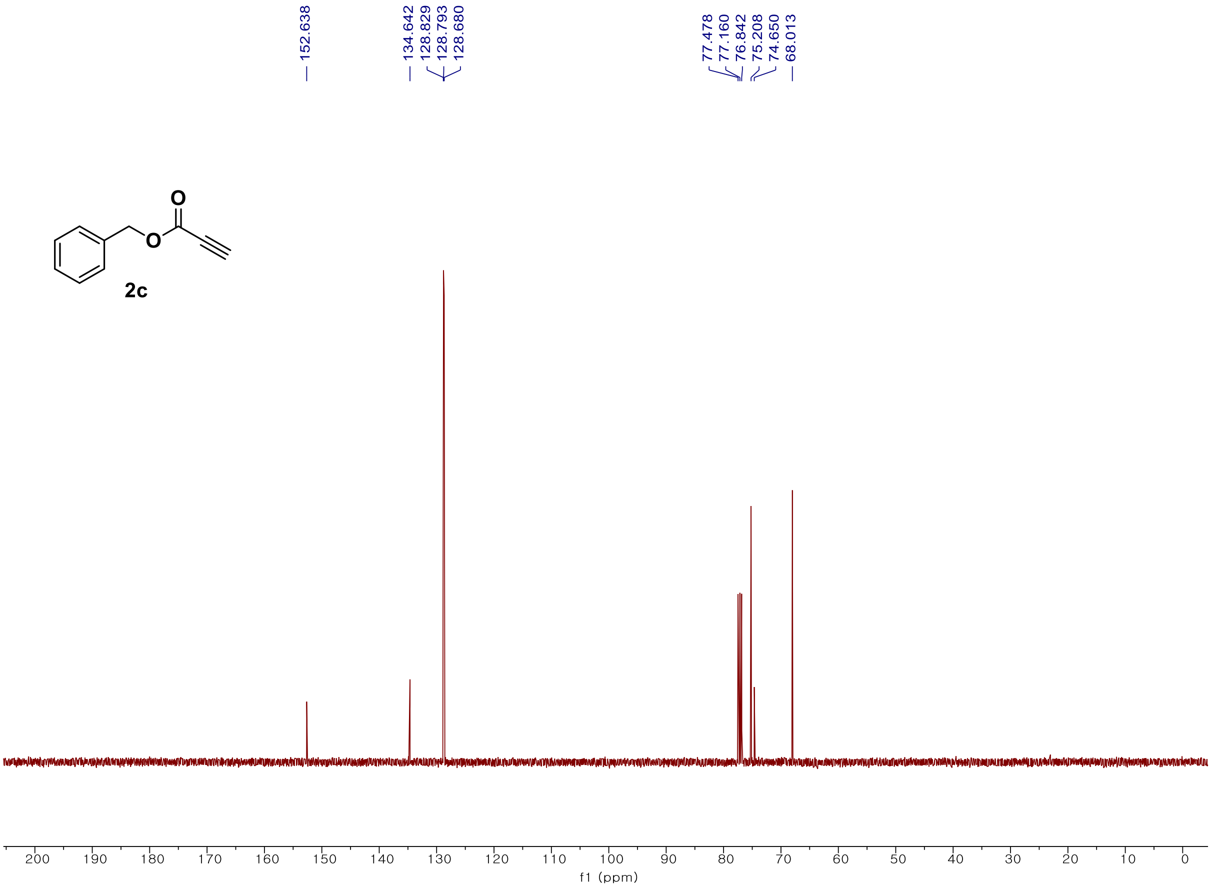
152.638
134.642
128.829
128.793
128.680
77.478
77.160
76.842
75.208
74.650
68.013
2c
200 190 180 170 160 150 140 130 120 110 100 90 80 70 60 50 40 30 20 10 0
f1 (ppm)

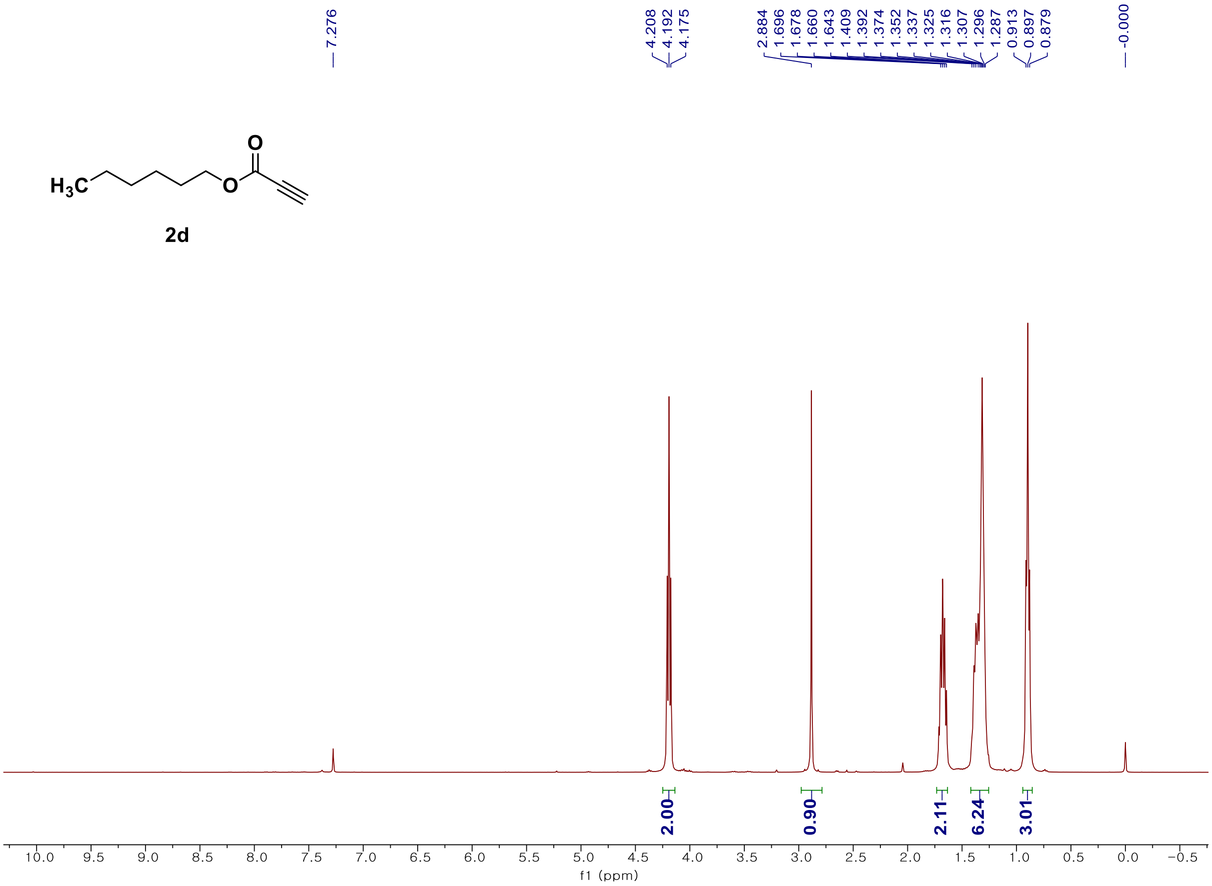

7.276
4.208
4.192
4.175
2.884
1.696
1.678
1.660
1.643
1.409
1.392
1.374
1.352
1.337
1.325
1.316
1.307
1.296
1.287
0.913
0.897
0.879
-0.000
H_3C
O
O
2.00
0.90
2.11
6.24
3.01
10.0 9.5 9.0 8.5 8.0 7.5 7.0 6.5 6.0 5.5 5.0 4.5 4.0 3.5 3.0 2.5 2.0 1.5 1.0 0.5 0.0 -0.5
f1 (ppm)

2d

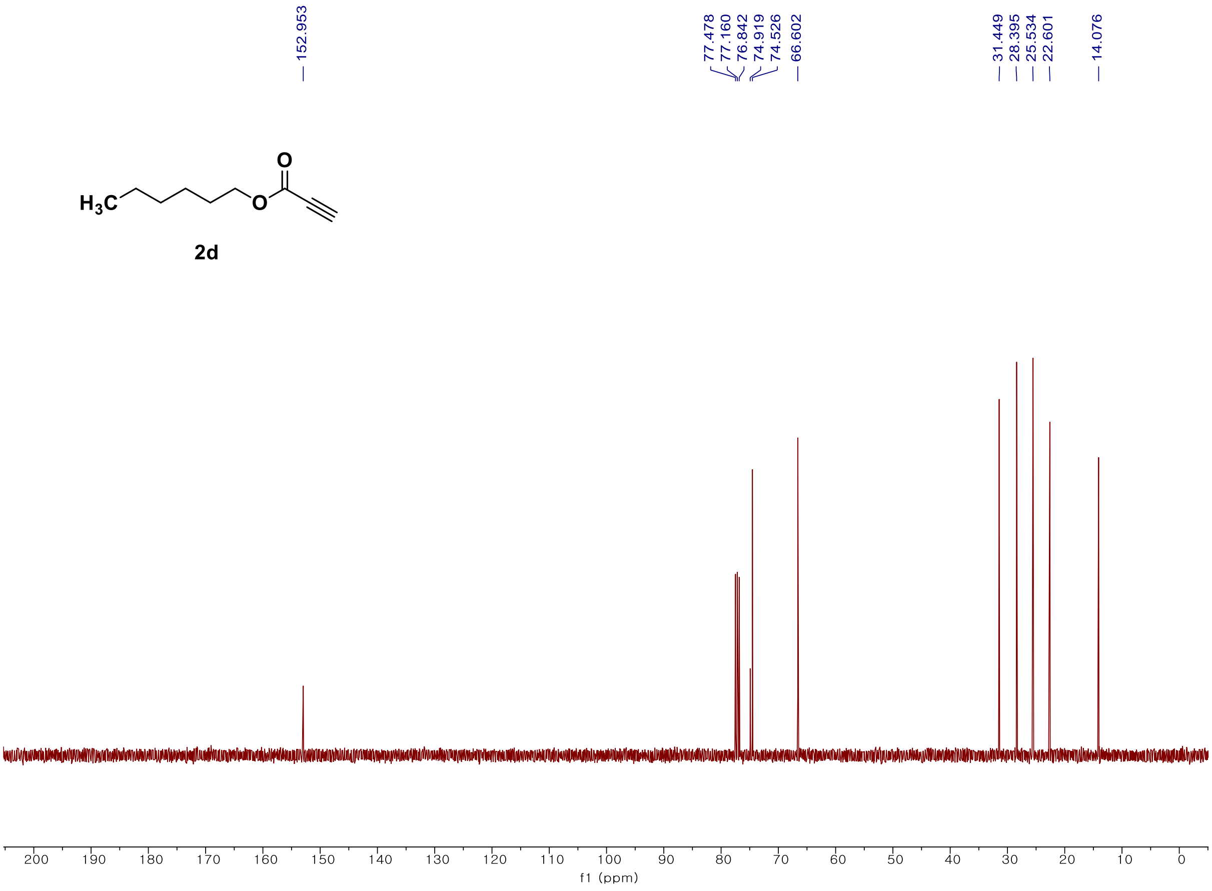

152.953
77.478
77.160
76.842
74.919
74.526
66.602
31.449
28.395
25.534
22.601
14.076
H_3C
O
O
200 190 180 170 160 150 140 130 120 110 100 90 80 70 60 50 40 30 20 10 0
f1 (ppm)

2d

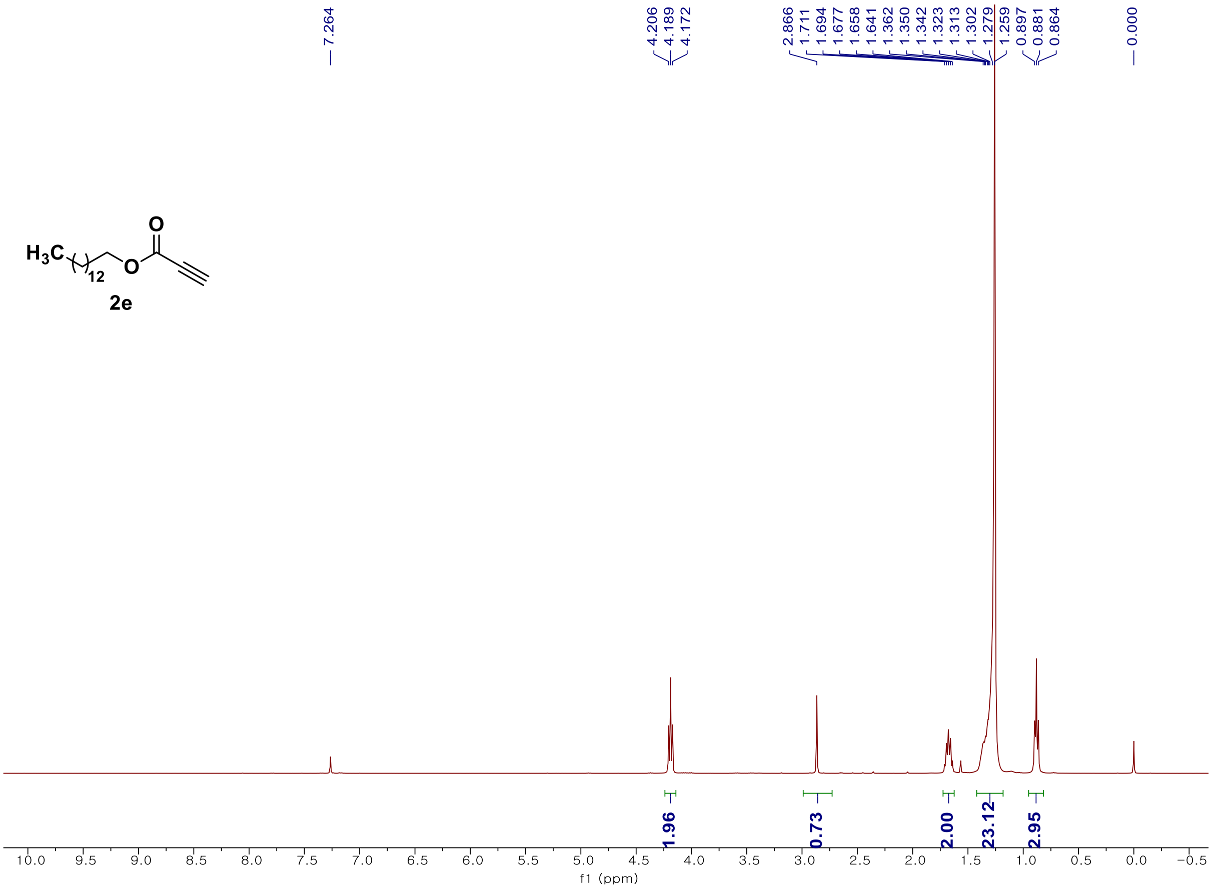
7.264
4.206
4.189
4.172
2.866
1.711
1.694
1.677
1.658
1.641
1.362
1.350
1.342
1.323
1.313
1.302
1.279
1.259
0.897
0.881
0.864
0.000
H3C
12
O
O
2e
1.96
0.73
2.00
23.12
2.95
f1 (ppm)

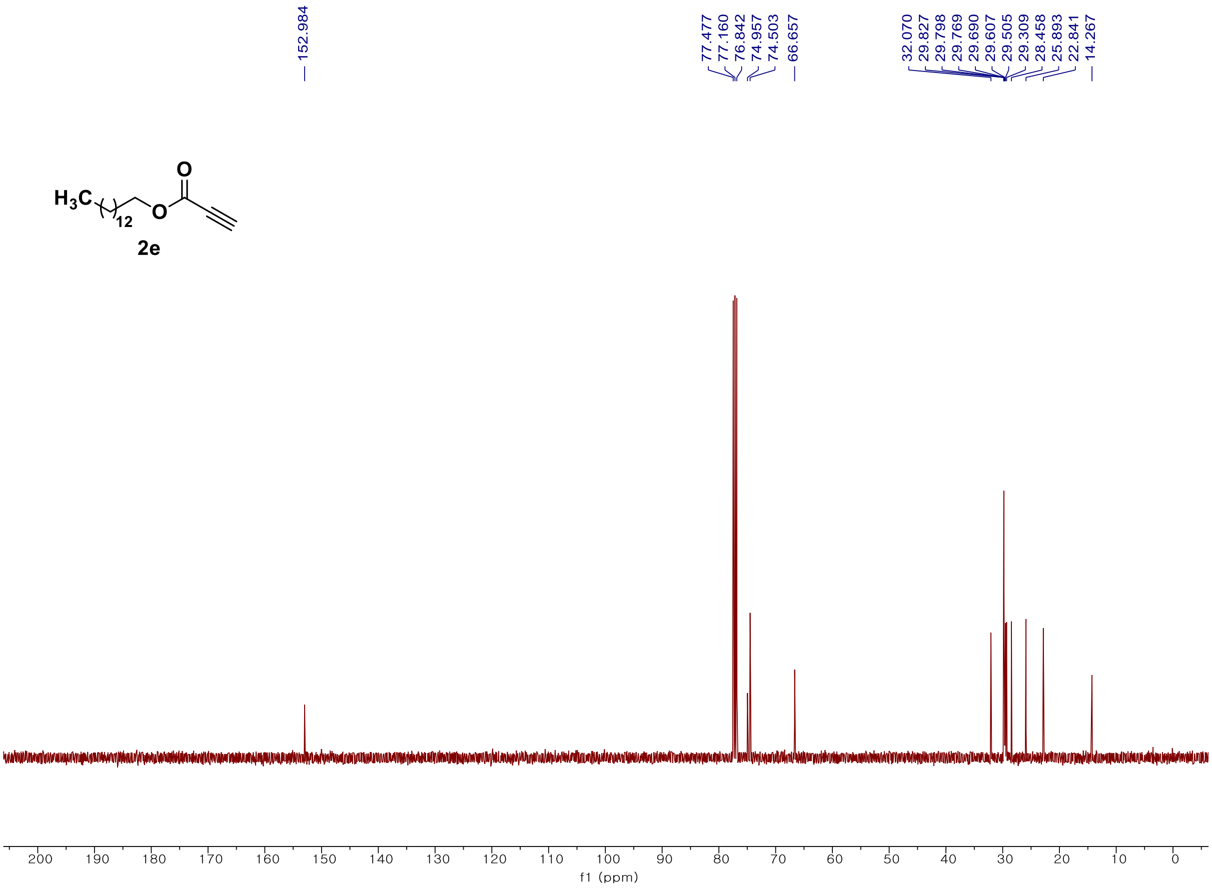
152.984
77.477
77.160
76.842
74.957
74.503
66.657
32.070
29.827
29.798
29.769
29.690
29.607
29.505
29.309
28.458
25.893
22.841
14.267
H3C
12
O
O
2e
f1 (ppm)

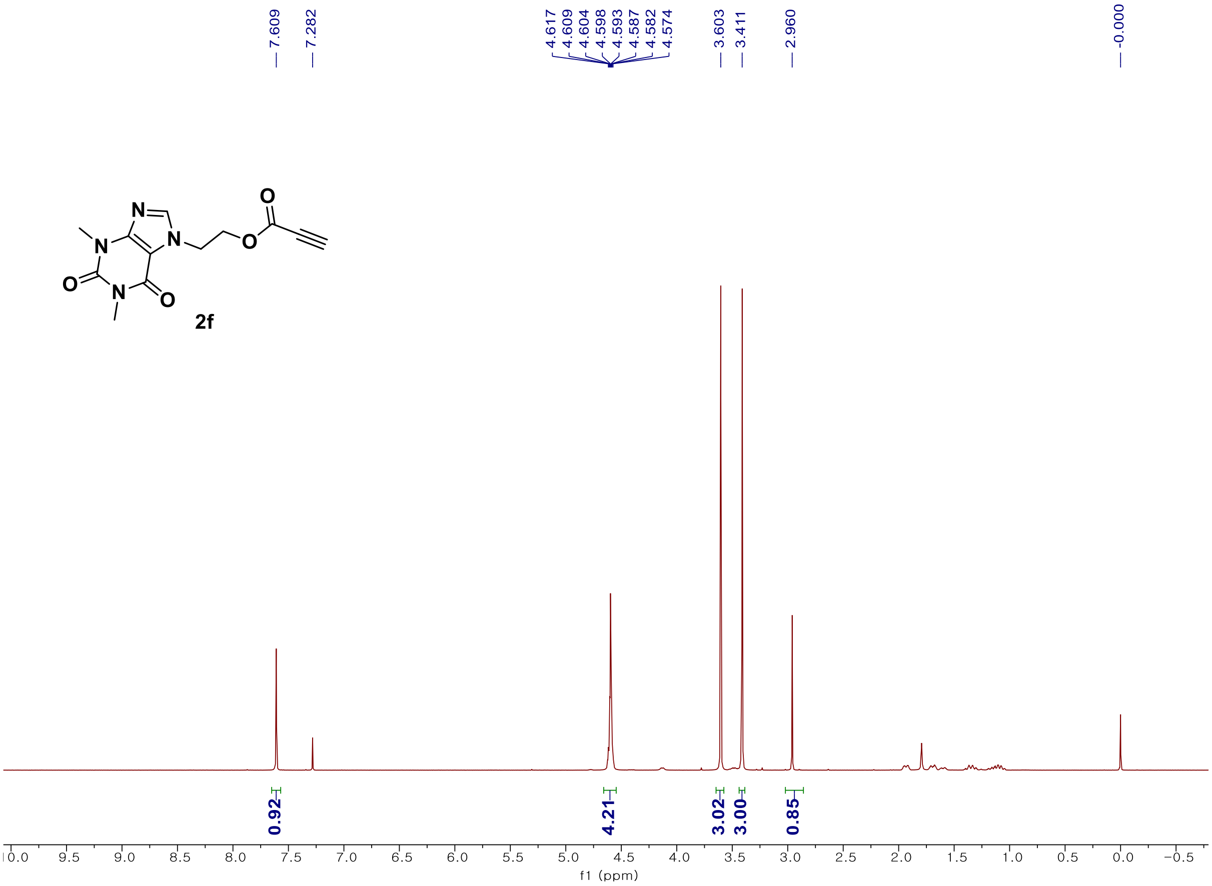
7.609
7.282
4.617
4.609
4.604
4.598
4.593
4.587
4.582
4.574
3.603
3.411
2.960
-0.000
2f
0.92
4.21
3.02
3.00
0.85
f1 (ppm)

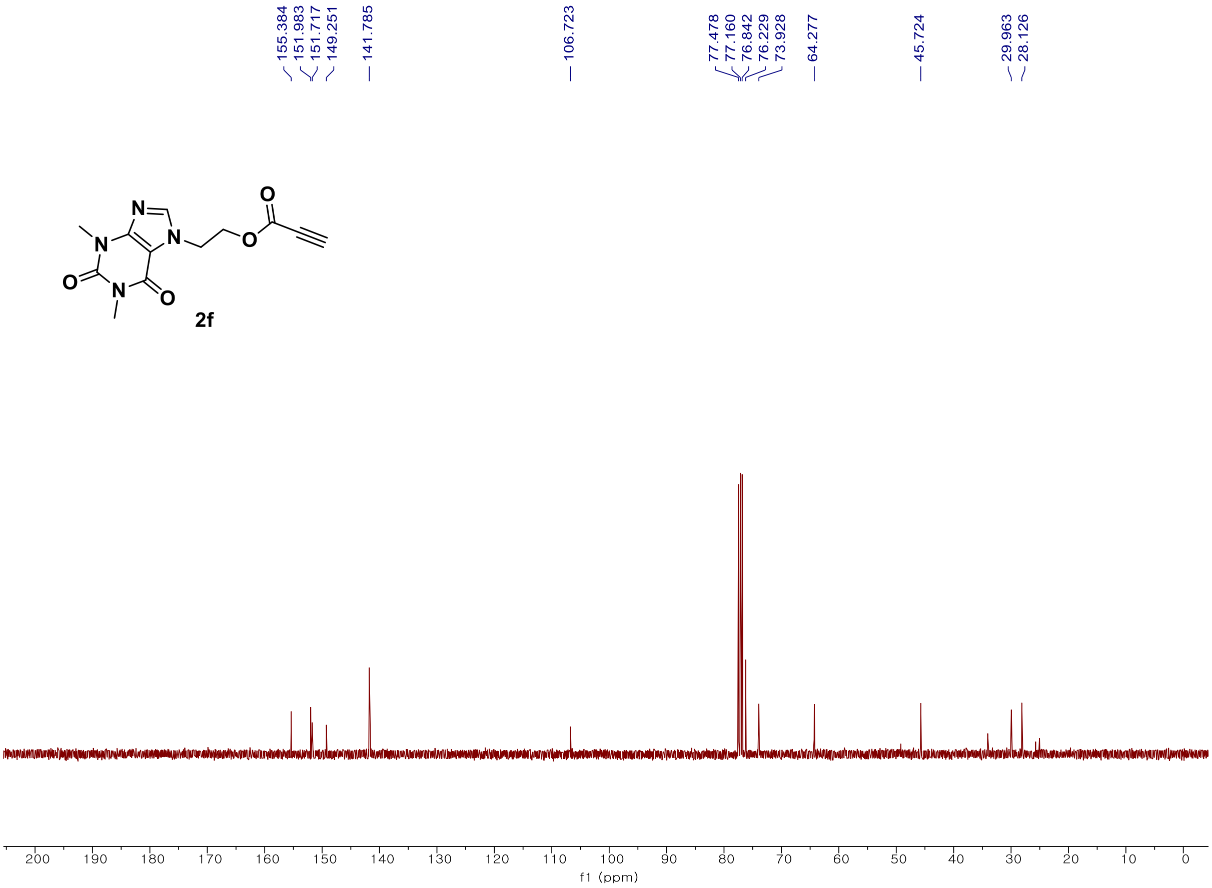
155.384
151.983
151.717
149.251
141.785
106.723
77.478
77.160
76.842
76.229
73.928
64.277
45.724
29.963
28.126
2f
f1 (ppm)

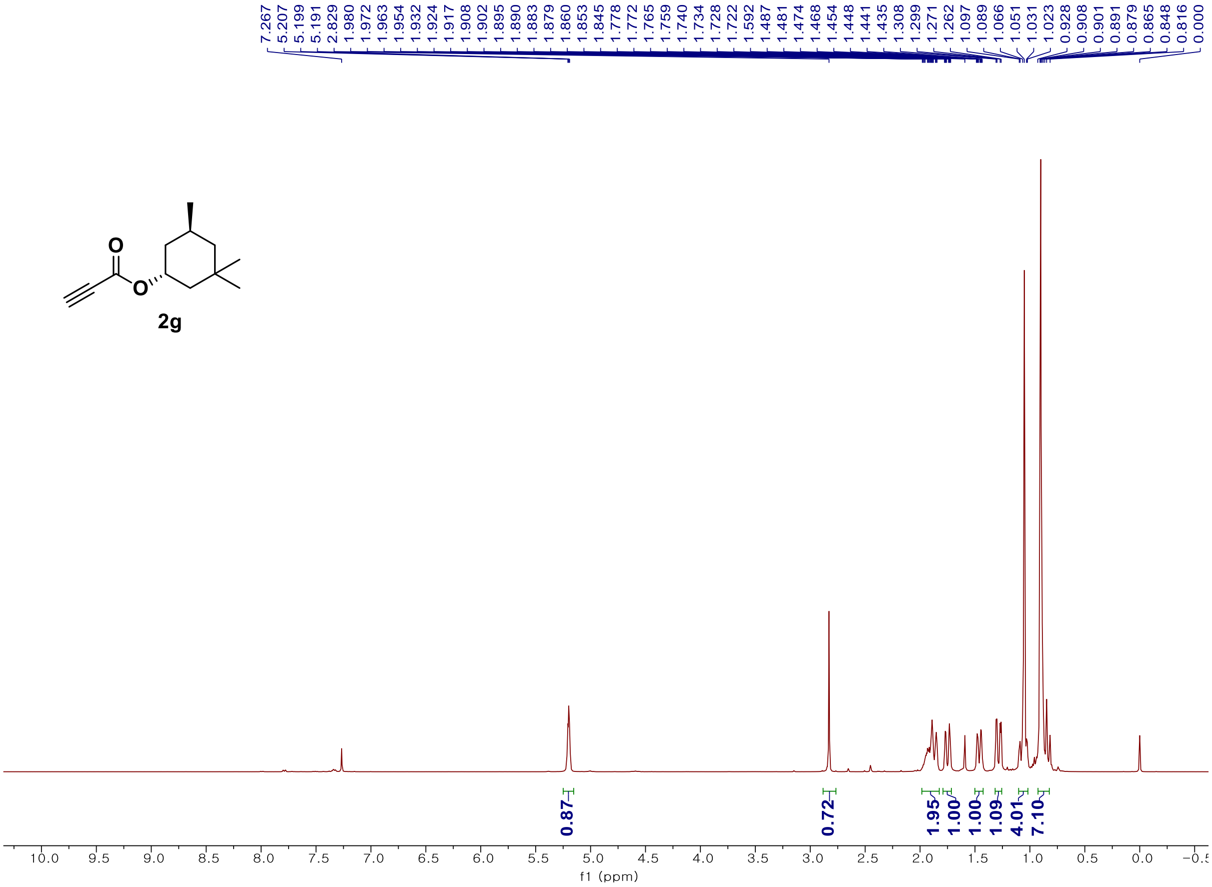
7.267
5.207
5.199
5.191
2.829
1.980
1.972
1.963
1.954
1.932
1.924
1.917
1.908
1.902
1.895
1.890
1.883
1.879
1.860
1.853
1.845
1.778
1.772
1.765
1.759
1.740
1.734
1.728
1.722
1.592
1.487
1.481
1.474
1.468
1.454
1.448
1.441
1.435
1.308
1.299
1.271
1.262
1.097
1.089
1.066
1.051
1.031
1.023
0.928
0.908
0.901
0.891
0.879
0.865
0.848
0.816
0.000
O
O
2g
0.87
0.72
1.95
1.00
1.00
1.09
4.01
7.10
10.0
9.5
9.0
8.5
8.0
7.5
7.0
6.5
6.0
5.5
5.0
4.5
4.0
3.5
3.0
2.5
2.0
1.5
1.0
0.5
0.0
f1 (ppm)

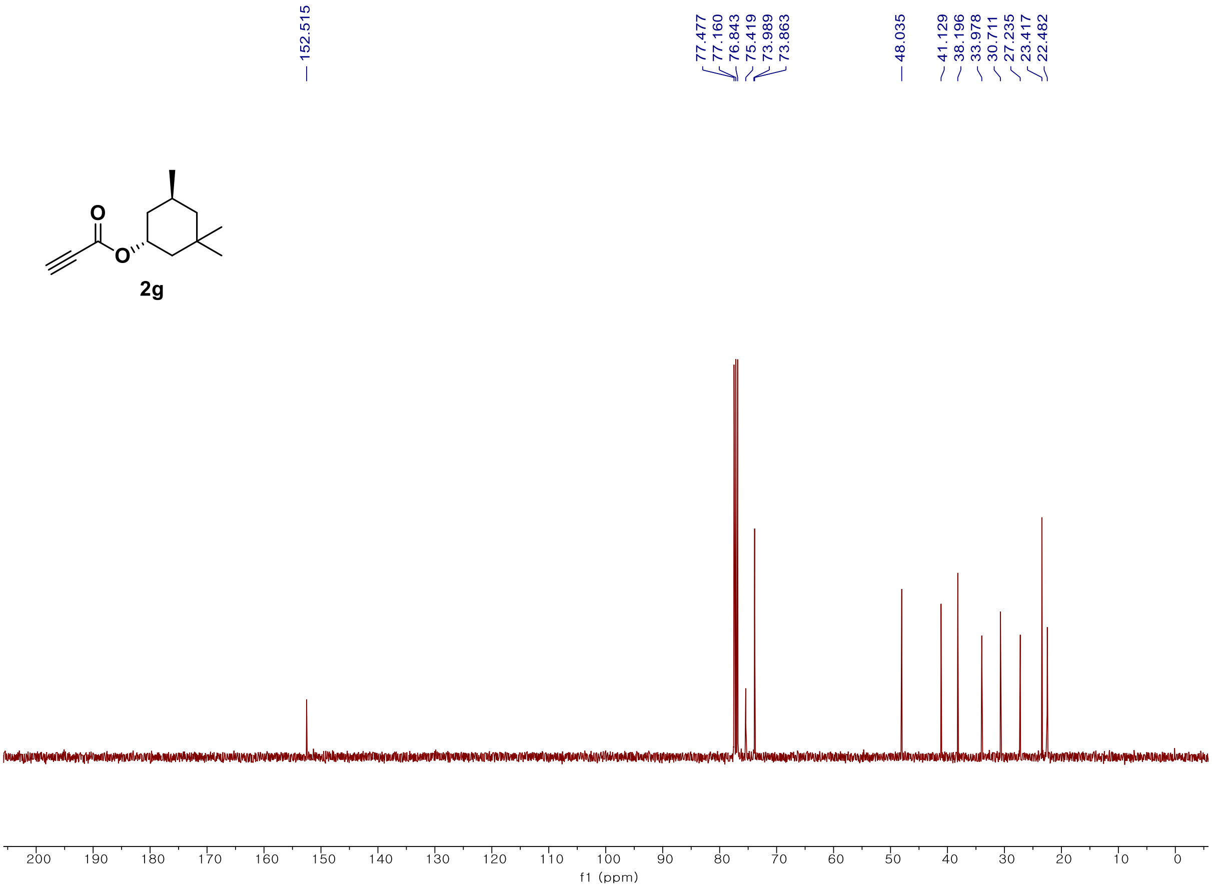
152.515
77.477
77.160
76.843
75.419
73.989
73.863
48.035
41.129
38.196
33.978
30.711
27.235
23.417
22.482
O
O
2g
200
190
180
170
160
150
140
130
120
110
100
90
80
70
60
50
40
30
20
10
0
f1 (ppm)

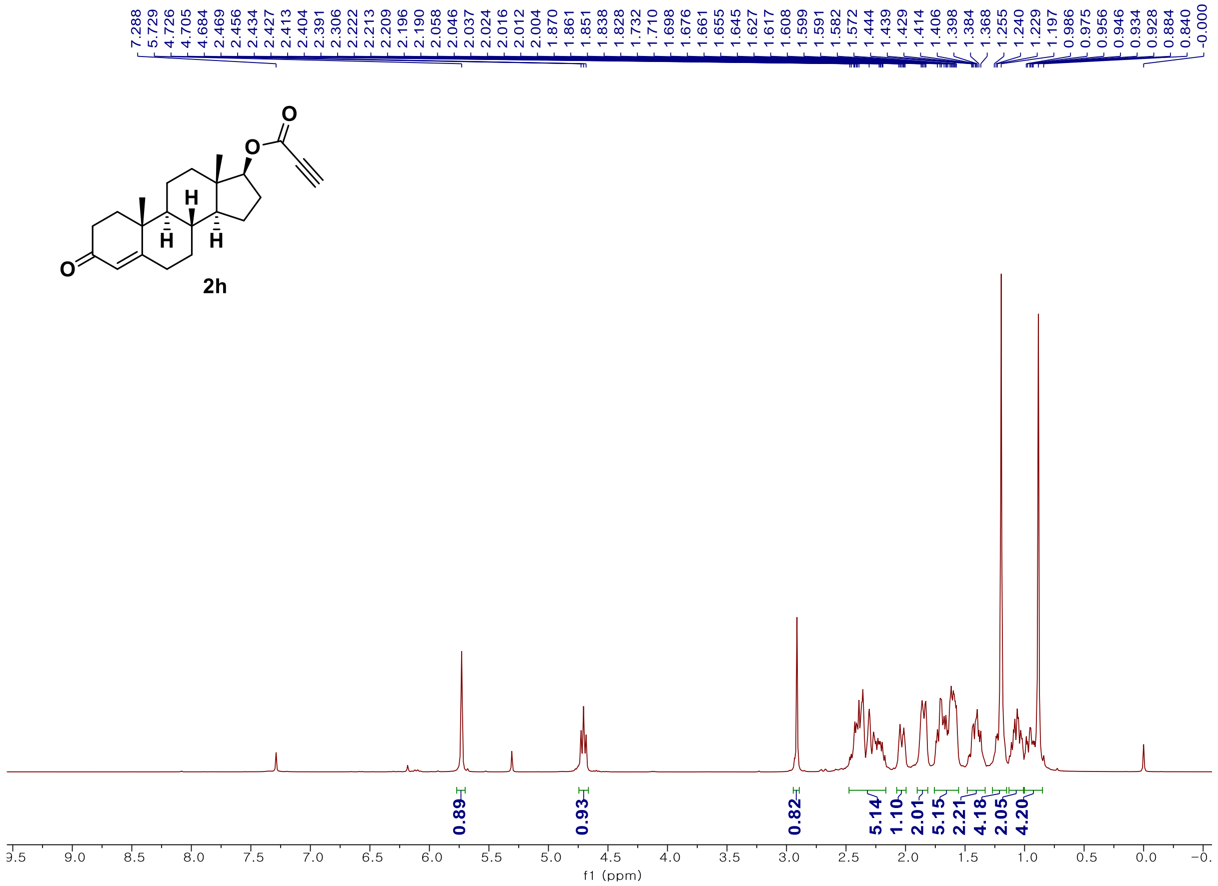

7.288
5.729
5.726
4.705
4.684
2.469
2.456
2.434
2.427
2.413
2.404
2.391
2.306
2.222
2.213
2.209
2.196
2.190
2.058
2.046
2.037
2.024
2.016
2.012
2.004
1.870
1.861
1.851
1.838
1.828
1.732
1.710
1.698
1.676
1.661
1.655
1.645
1.627
1.617
1.608
1.599
1.591
1.582
1.572
1.444
1.439
1.429
1.414
1.406
1.398
1.384
1.368
1.255
1.240
1.229
1.197
0.986
0.975
0.956
0.946
0.934
0.928
0.884
0.840
-0.000
2h
0.89
0.93
0.82
5.14
1.10
2.01
5.15
2.21
4.18
2.05
4.20
f1 (ppm)

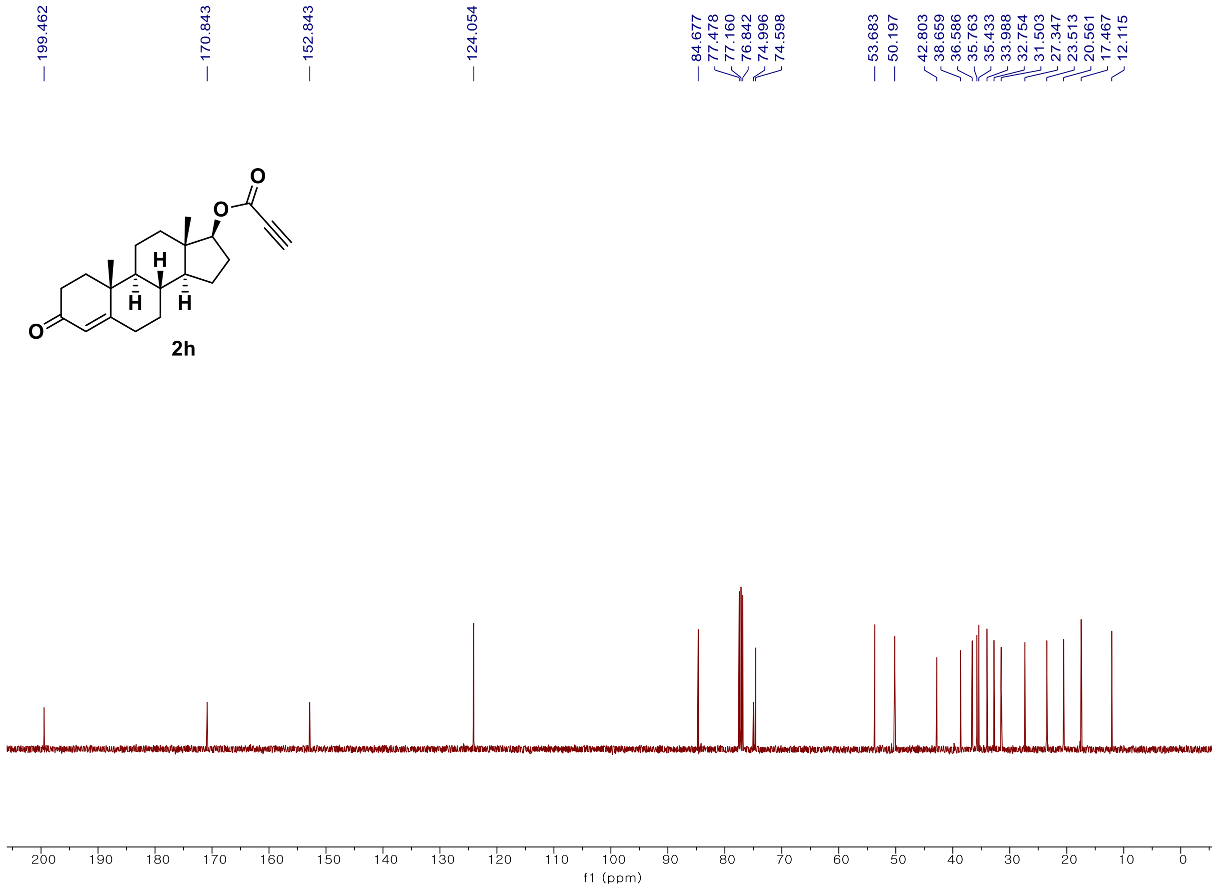

199.462
170.843
152.843
124.054
84.677
77.478
77.160
76.842
74.996
74.598
53.683
50.197
42.803
38.659
36.586
35.763
35.433
33.988
32.754
31.503
27.347
23.513
20.561
17.467
12.115
2h
f1 (ppm)

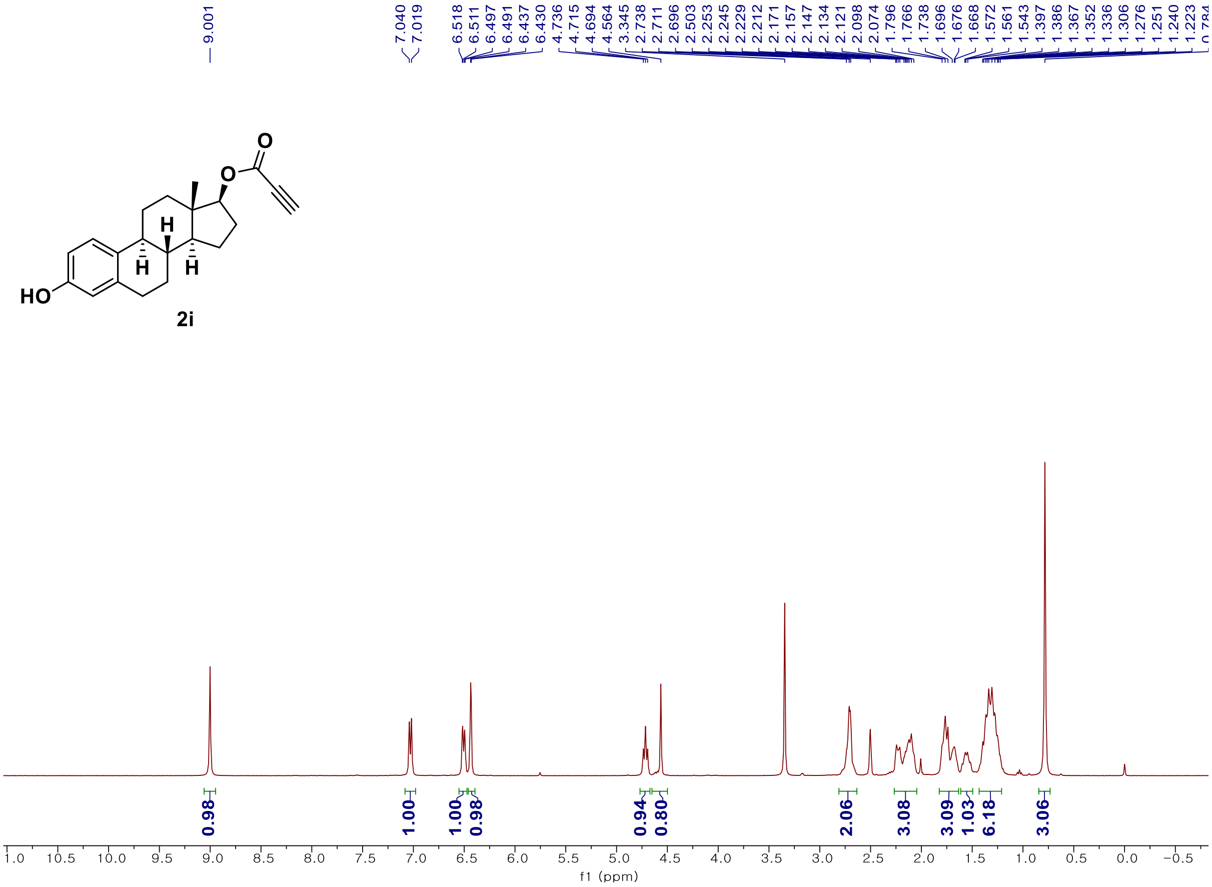
9.001
7.040
7.019
6.518
6.511
6.497
6.491
6.437
6.430
4.736
4.715
4.694
4.564
3.345
2.738
2.711
2.696
2.503
2.253
2.245
2.229
2.212
2.171
2.157
2.147
2.134
2.121
2.098
2.074
1.796
1.766
1.738
1.696
1.676
1.668
1.572
1.561
1.543
1.397
1.386
1.367
1.352
1.336
1.306
1.276
1.251
1.240
1.223
O
HO
H
H
H
2i
0.98
1.00
1.00
0.98
0.94
0.80
2.06
3.08
3.09
1.03
6.18
3.06
f1 (ppm)

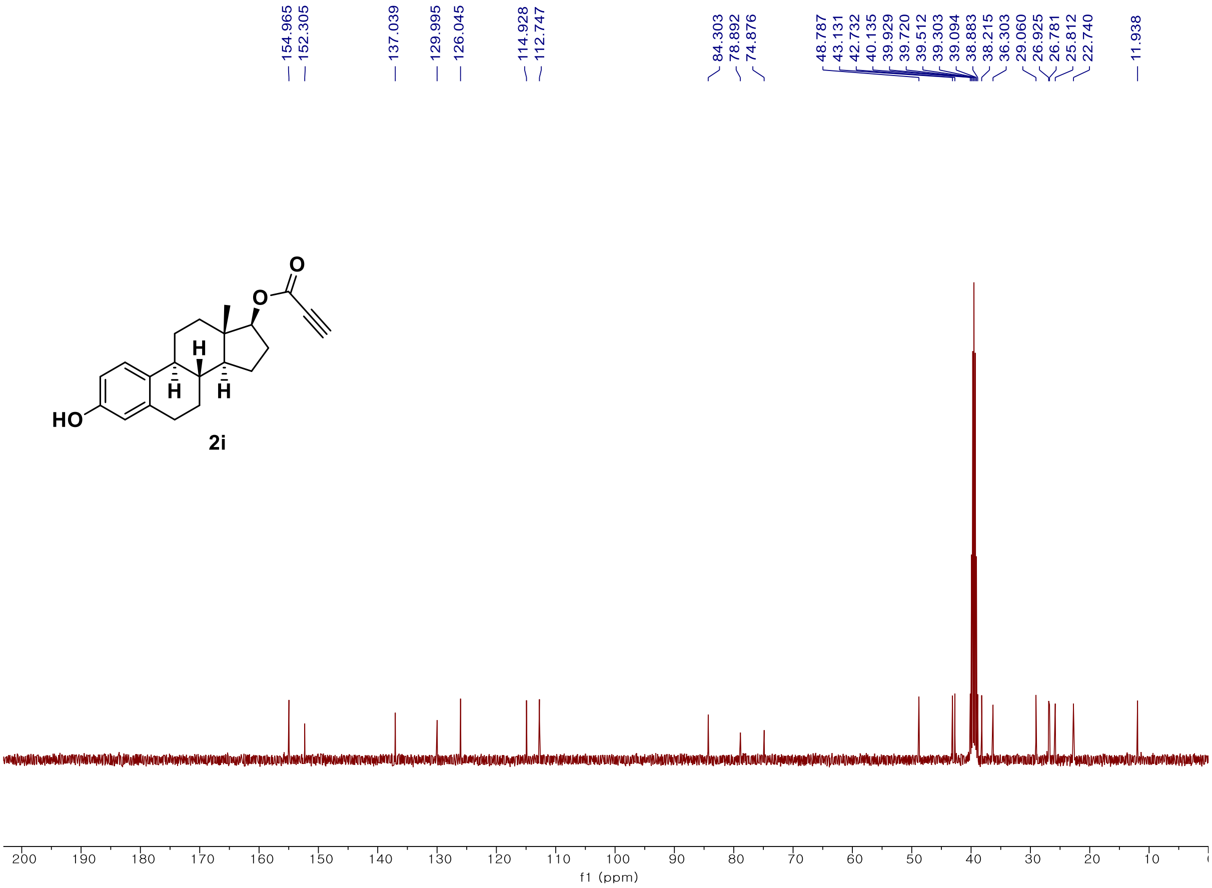
154.965
152.305
137.039
129.995
126.045
114.928
112.747
84.303
78.892
74.876
48.787
43.131
42.732
40.135
39.929
39.720
39.512
39.303
39.094
38.883
38.215
36.303
29.060
26.925
26.781
25.812
22.740
11.938
O
HO
H
H
H
2i
f1 (ppm)

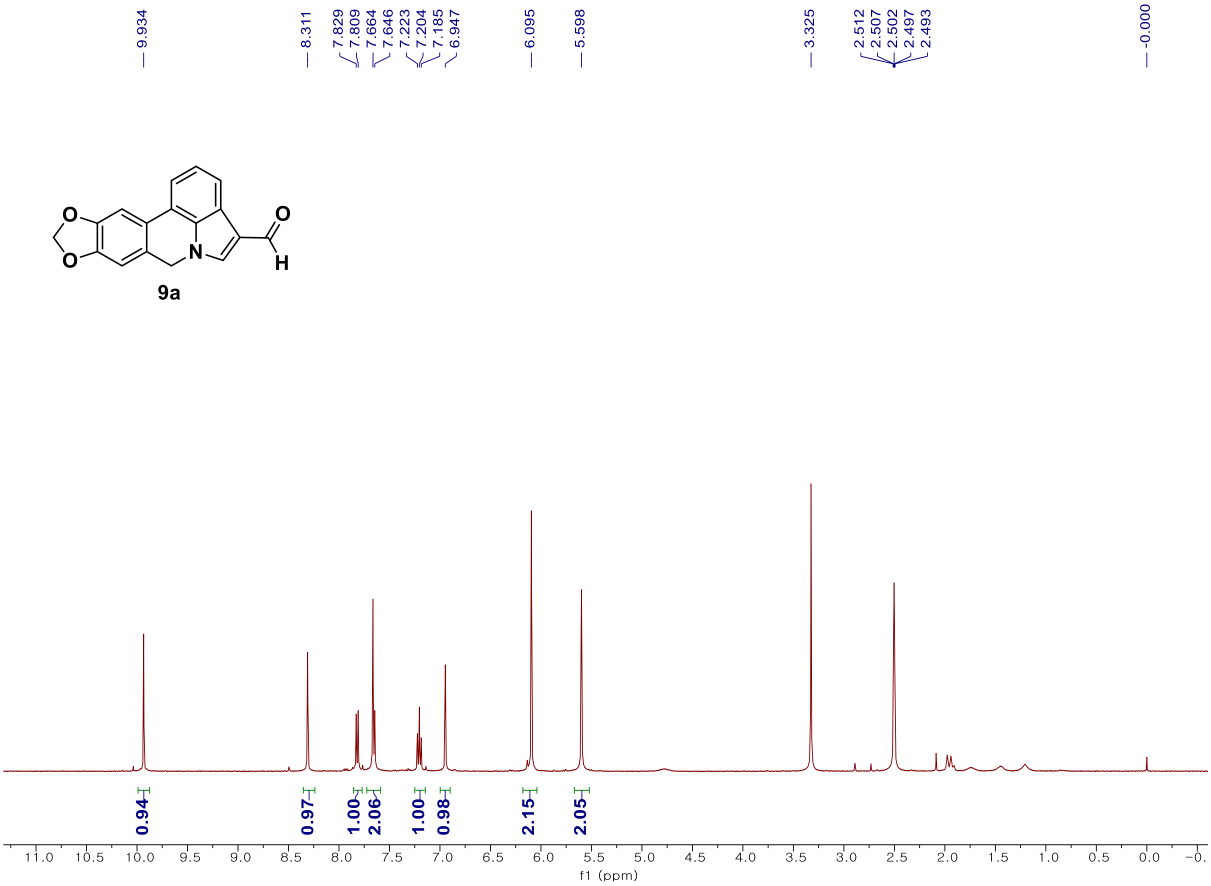

9.934
8.311
7.829
7.809
7.664
7.646
7.223
7.204
7.185
6.947
6.095
5.598
3.325
2.512
2.507
2.502
2.497
2.493
-0.000
0.94
0.97
1.00
2.06
1.00
0.98
2.15
2.05
f1 (ppm)
O
O
N
O
H

9a

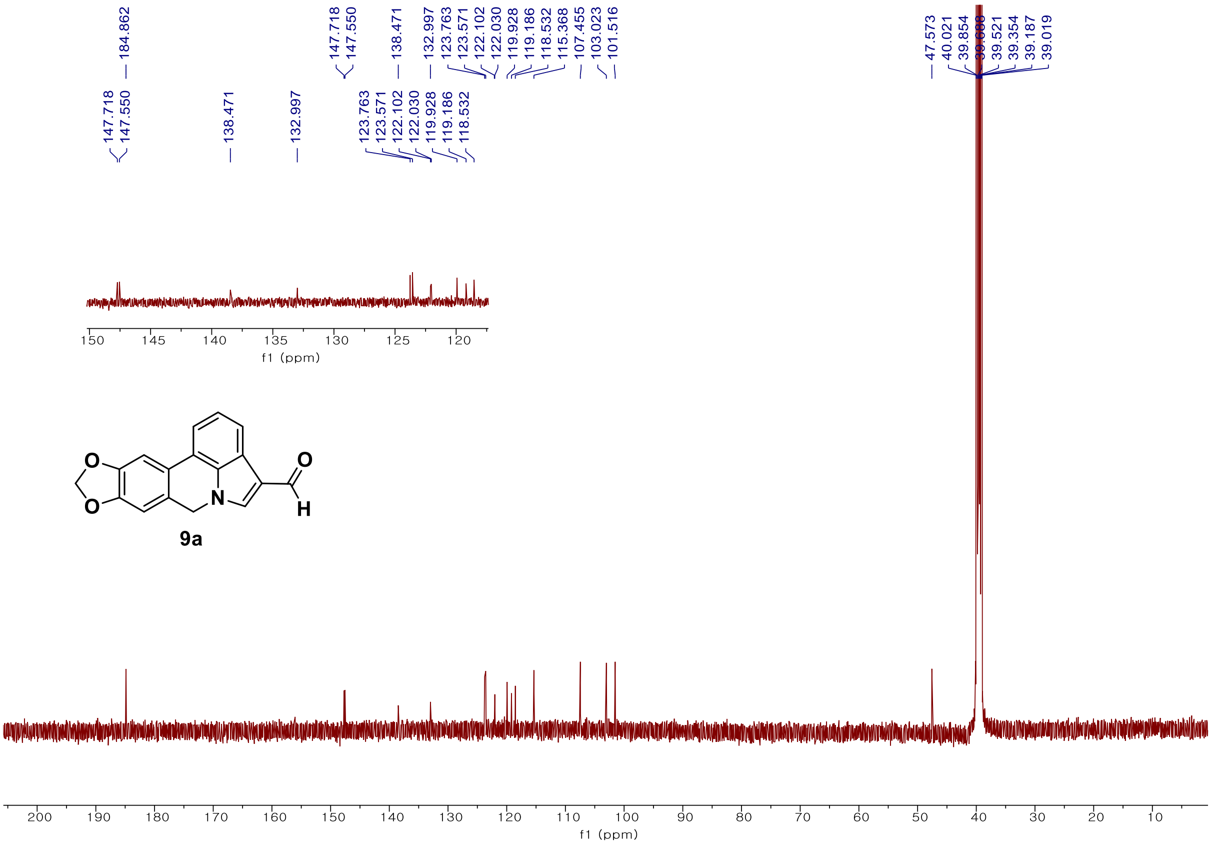

184.862
147.718
147.550
138.471
132.997
123.763
123.571
122.102
122.030
119.928
119.186
118.532
115.368
107.455
103.023
101.516
47.573
40.021
39.854
39.521
39.354
39.187
39.019
f1 (ppm)
O
O
N
O
H

9a

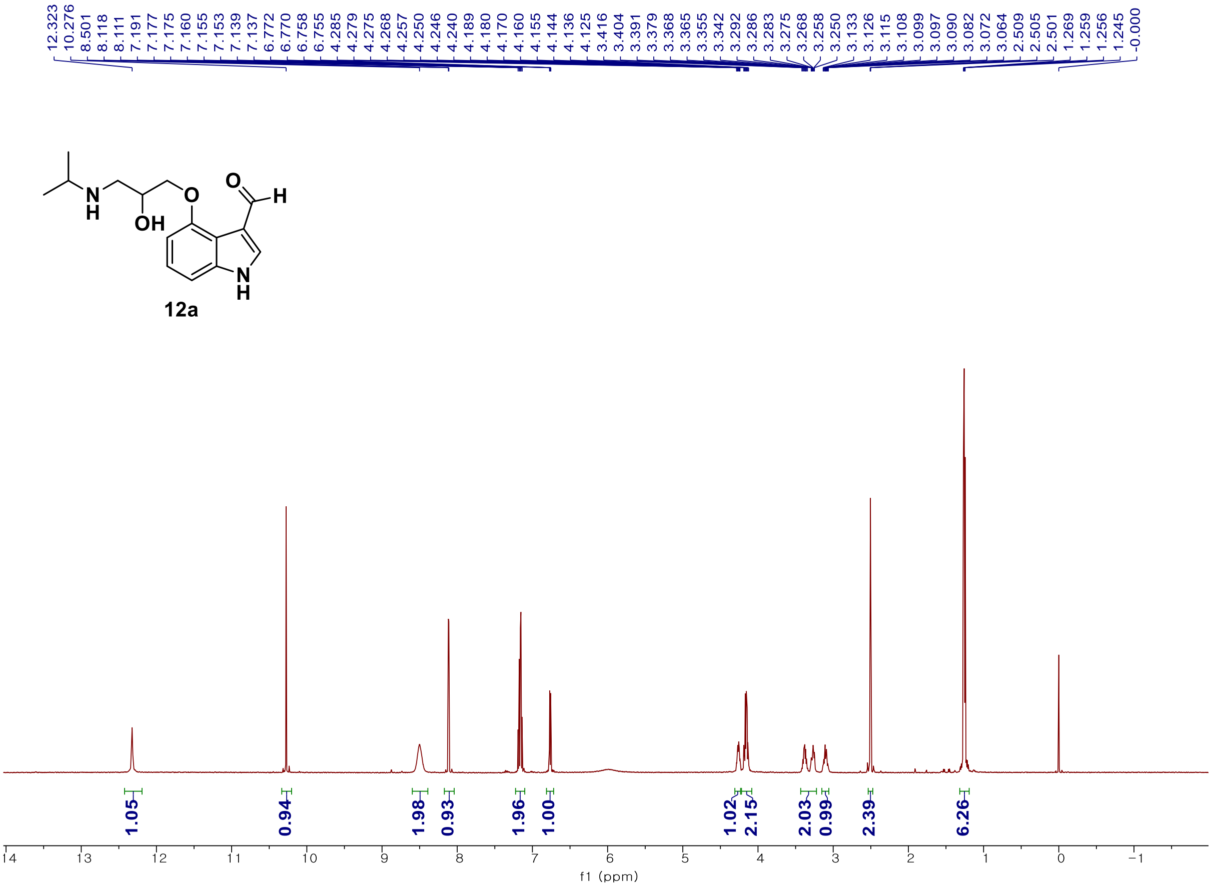
12.323
10.276
8.501
8.118
8.111
7.191
7.177
7.175
7.160
7.155
7.153
7.139
7.137
6.772
6.770
6.758
6.755
4.285
4.279
4.275
4.268
4.257
4.250
4.246
4.240
4.189
4.180
4.170
4.160
4.155
4.144
4.136
4.125
3.416
3.404
3.391
3.379
3.368
3.365
3.355
3.342
3.292
3.286
3.283
3.275
3.268
3.258
3.250
3.133
3.126
3.115
3.108
3.099
3.097
3.090
3.082
3.072
3.064
2.509
2.505
2.501
1.269
1.259
1.256
1.245
-0.000
12a
1.05
0.94
1.98
0.93
1.96
1.00
1.02
2.15
2.03
0.99
2.39
6.26
f1 (ppm)

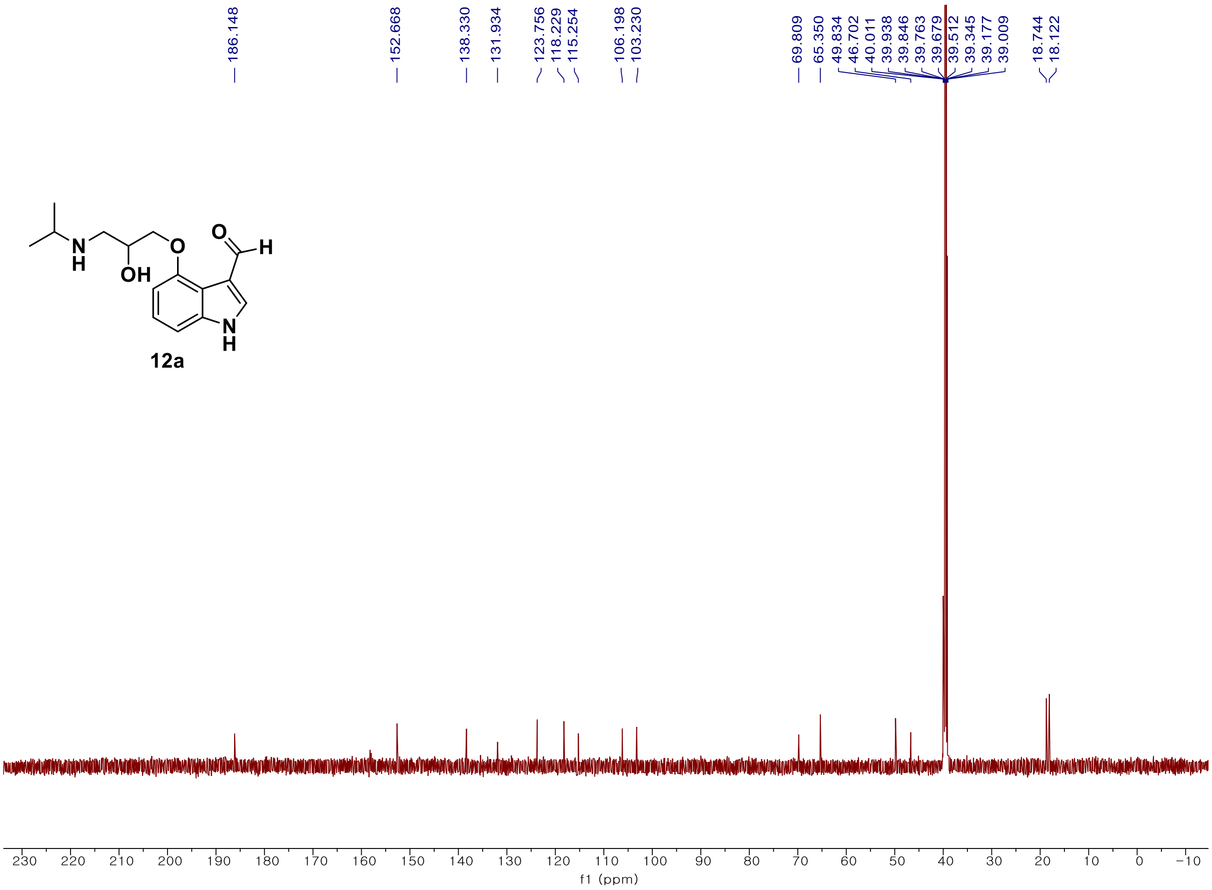
186.148
152.668
138.330
131.934
123.756
118.229
115.254
106.198
103.230
69.809
65.350
49.834
46.702
40.011
39.938
39.846
39.763
39.679
39.512
39.345
39.177
39.009
18.744
18.122
12a
f1 (ppm)

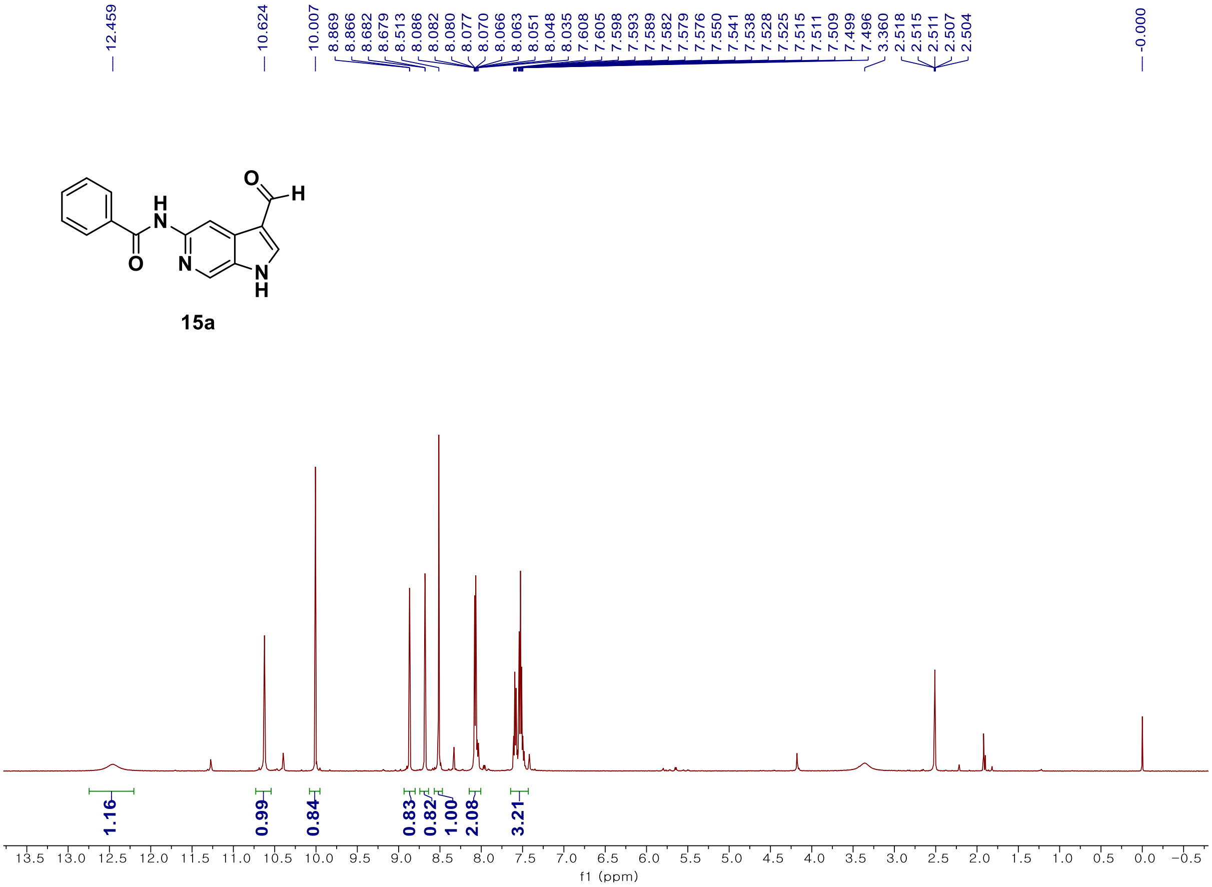
12.459
10.624
10.007
8.869
8.866
8.682
8.679
8.513
8.086
8.082
8.080
8.077
8.070
8.066
8.063
8.051
8.048
8.035
7.608
7.605
7.598
7.593
7.589
7.582
7.579
7.576
7.550
7.541
7.538
7.528
7.525
7.515
7.511
7.509
7.499
7.496
3.360
2.518
2.515
2.511
2.507
2.504
-0.000
15a
1.16
0.99
0.84
0.83
0.82
1.00
2.08
3.21
f1 (ppm)

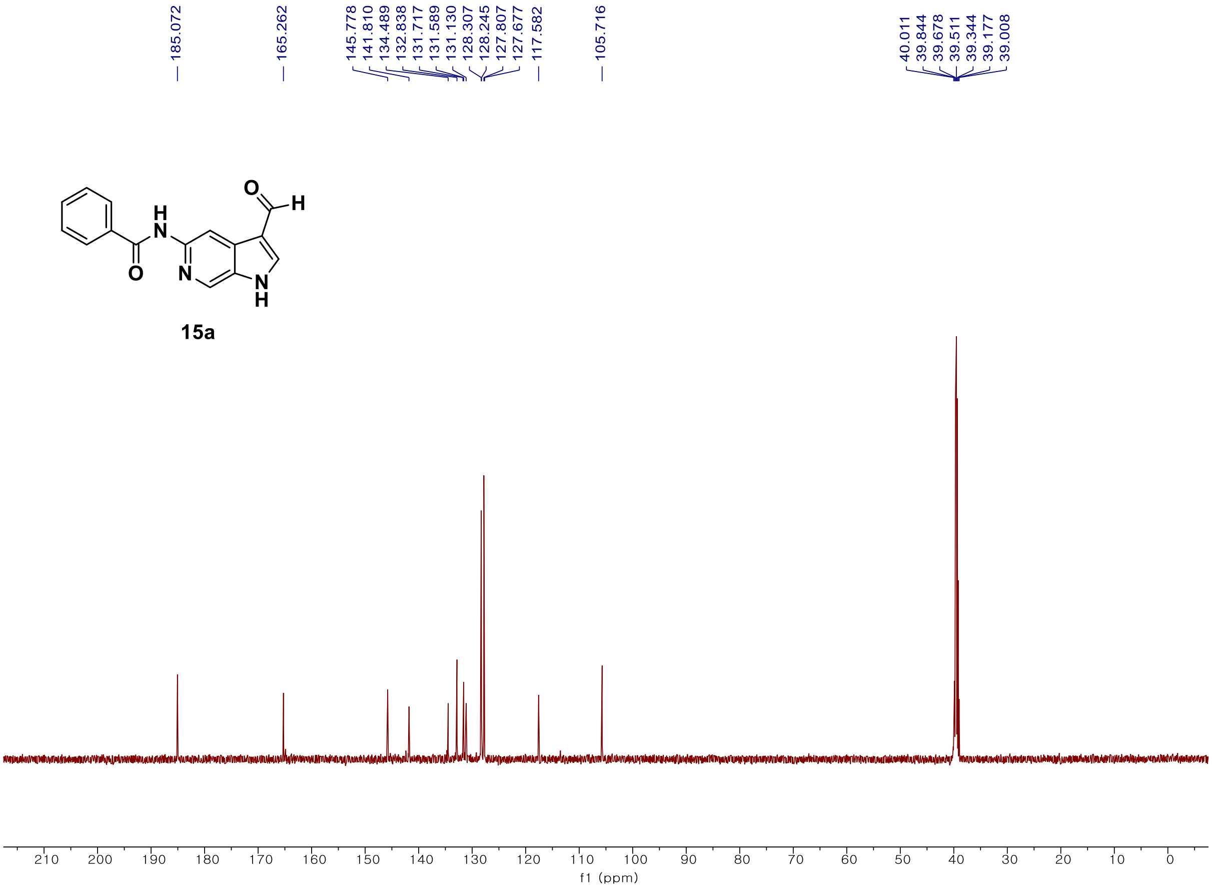
185.072
165.262
145.778
141.810
134.489
132.838
131.717
131.589
131.130
128.307
128.245
127.807
127.677
117.582
105.716
40.011
39.844
39.678
39.511
39.344
39.177
39.008
15a
f1 (ppm)

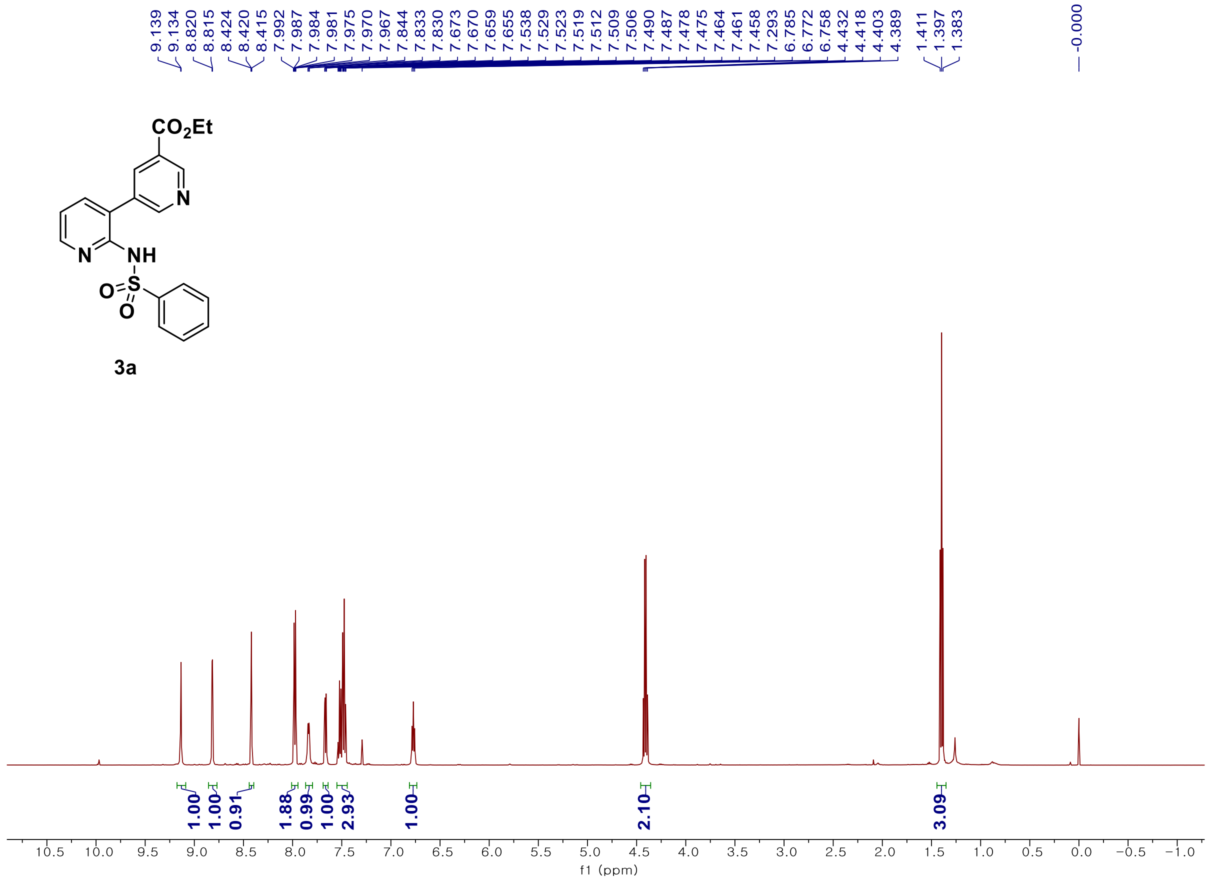
9.139
9.134
8.820
8.815
8.424
8.420
8.415
7.992
7.987
7.984
7.981
7.975
7.970
7.967
7.844
7.833
7.830
7.673
7.670
7.659
7.655
7.538
7.529
7.523
7.519
7.512
7.509
7.506
7.490
7.487
7.478
7.475
7.464
7.461
7.458
7.293
6.785
6.772
6.758
4.432
4.418
4.403
4.389
1.411
1.397
1.383
-0.000
CO_2Et
N
N
NH
O=S
O
3a
1.00
1.00
0.91
1.88
0.99
1.00
2.93
1.00
2.10
3.09
f1 (ppm)

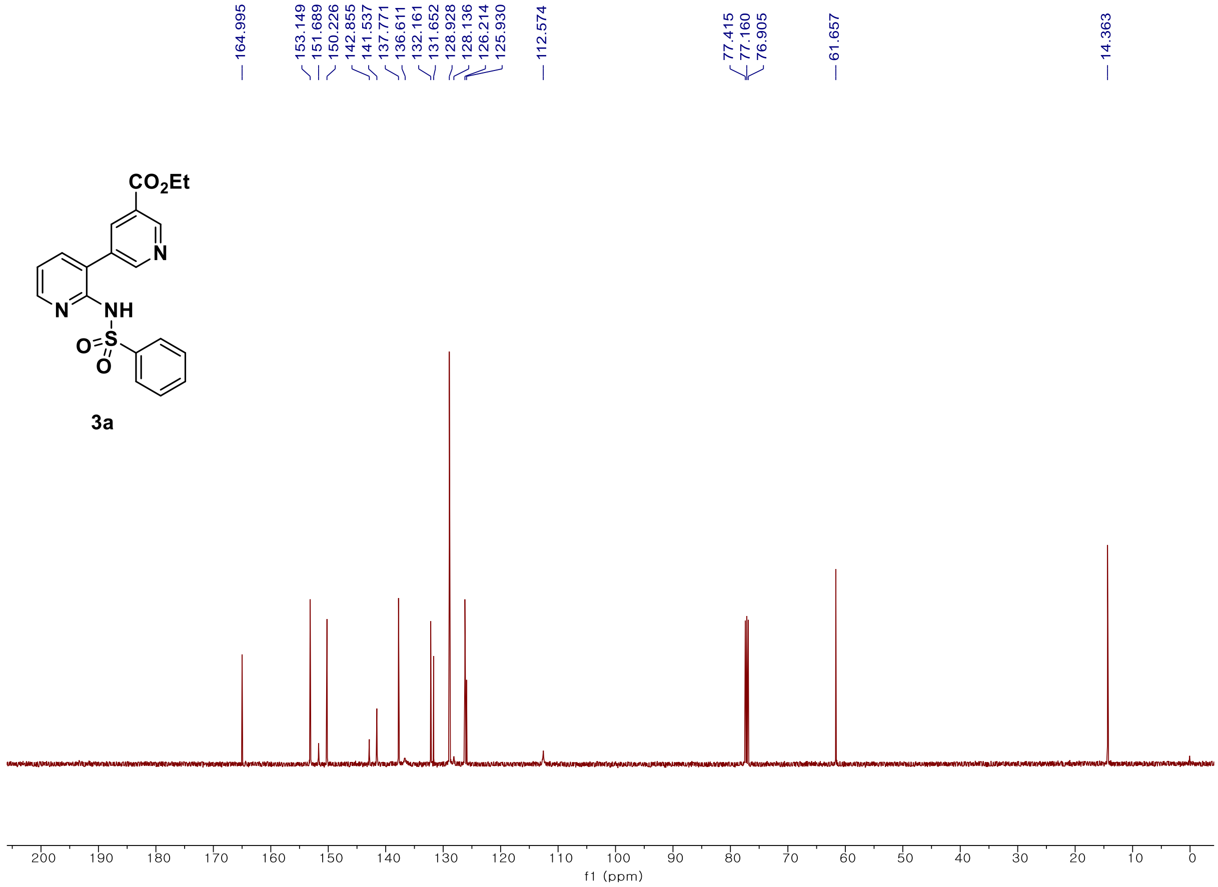
164.995
153.149
151.689
150.226
142.855
141.537
137.771
136.611
132.161
131.652
128.928
128.136
126.214
125.930
112.574
77.415
77.160
76.905
61.657
14.363
CO_2Et
N
N
NH
O=S
O
3a
f1 (ppm)

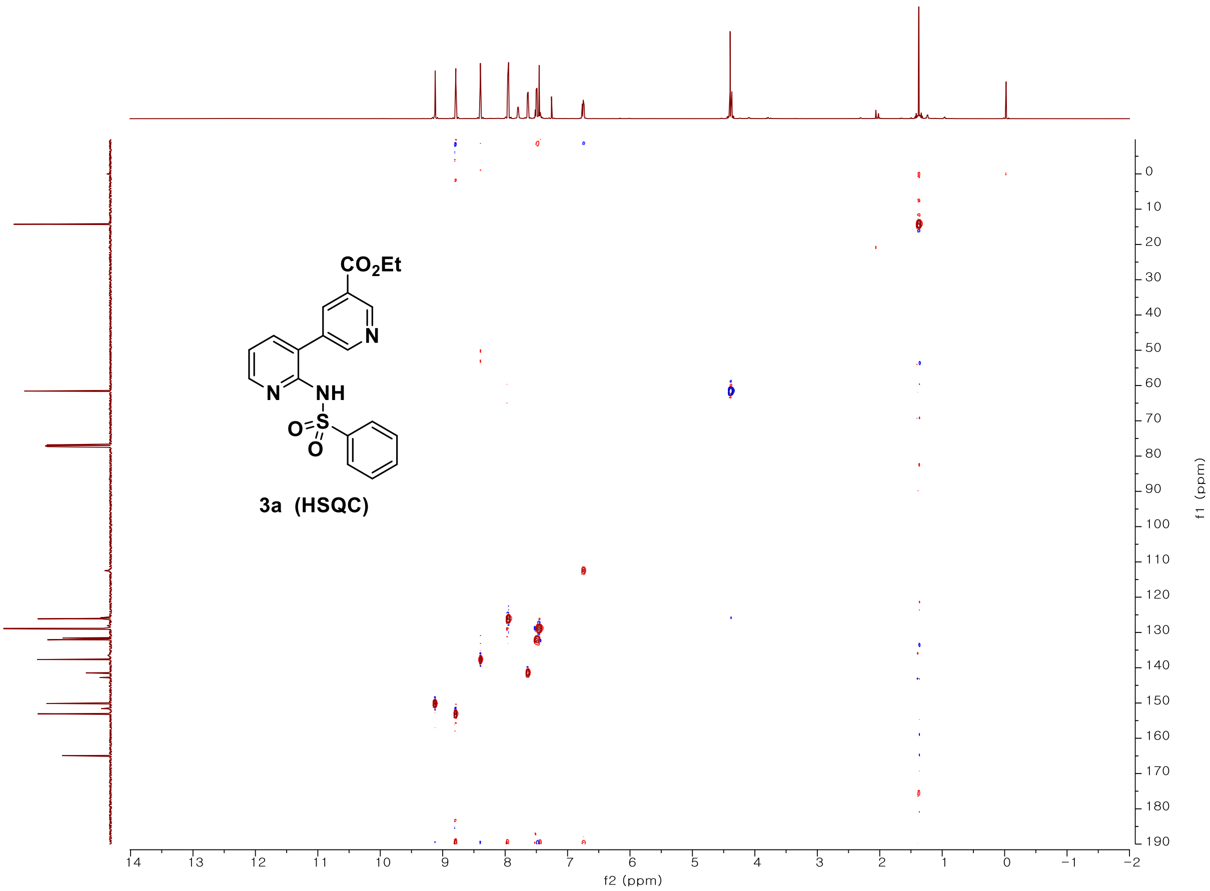
CO2Et
N
N
NH
O=S
O
3a (HSQC)
f2 (ppm)
f1 (ppm)

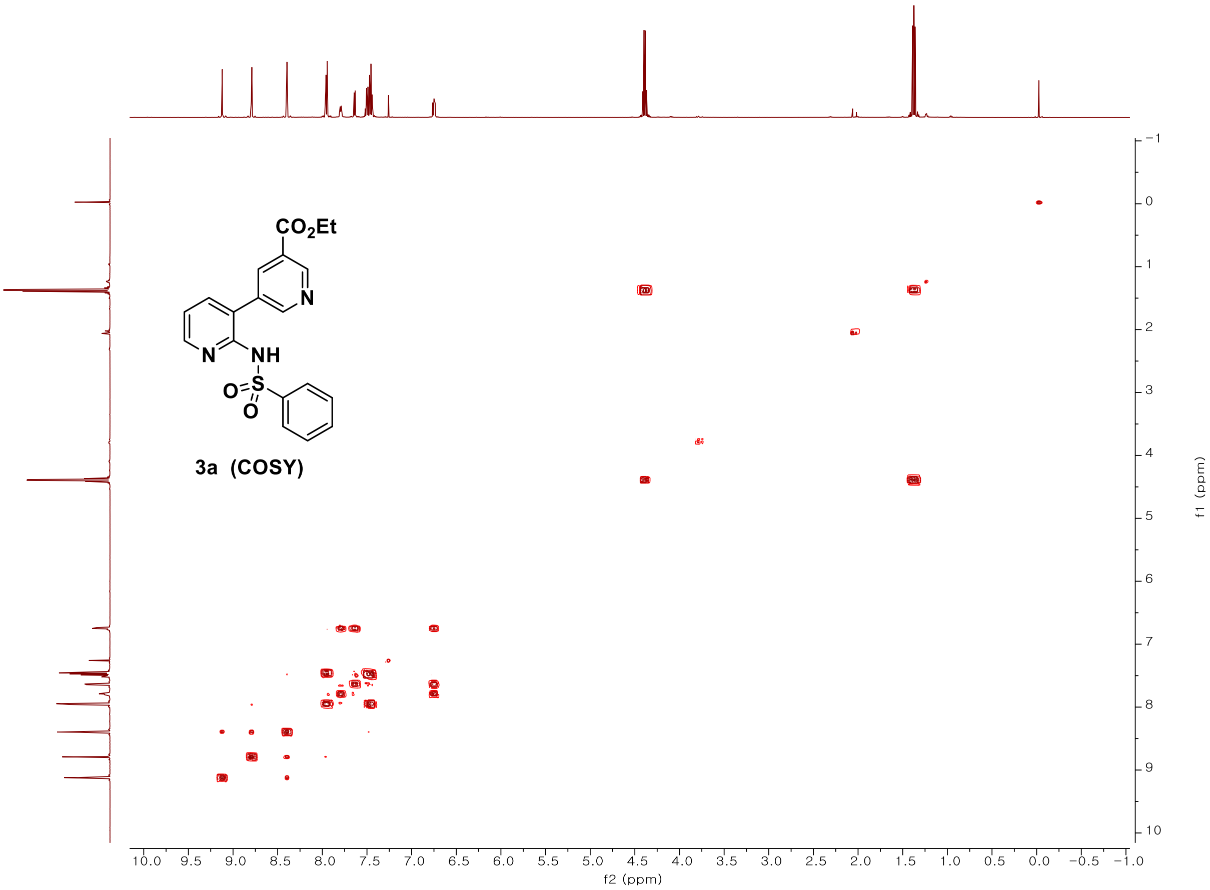
CO2Et
N
N
NH
O=S
O
3a (COSY)
f2 (ppm)
f1 (ppm)

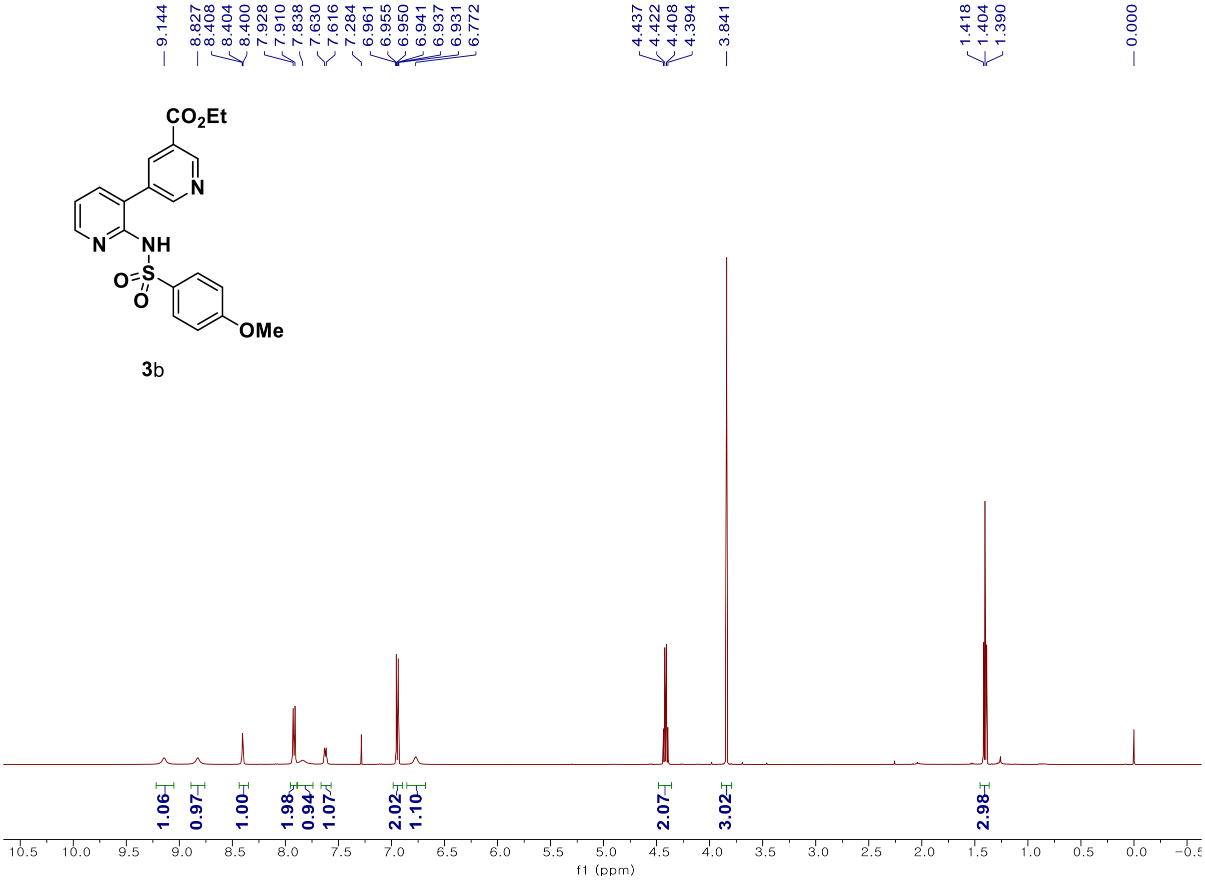
9.144
8.827
8.408
8.404
8.400
7.928
7.910
7.838
7.630
7.616
7.284
6.961
6.955
6.950
6.941
6.937
6.931
6.772
4.437
4.422
4.408
4.394
3.841
1.418
1.404
1.390
0.000
CO_2Et
N
N
NH
O=S
O
OMe
1.06
0.97
1.00
1.98
0.94
1.07
2.02
1.10
2.07
3.02
2.98
f1 (ppm)

3b

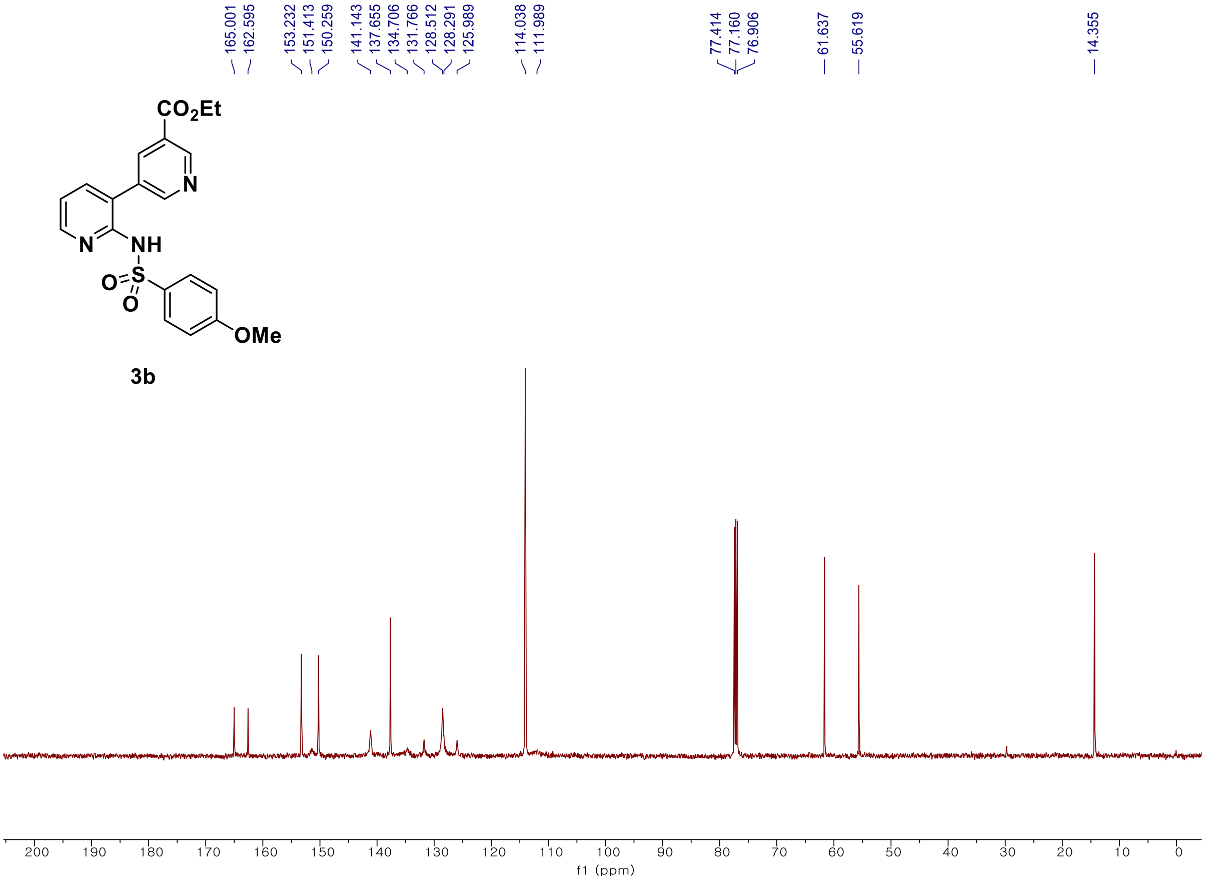
165.001
162.595
153.232
151.413
150.259
141.143
137.655
134.706
131.766
128.512
128.291
125.989
114.038
111.989
77.414
77.160
76.906
61.637
55.619
14.355
CO_2Et
N
N
NH
O=S
O
OMe
f1 (ppm)

3b

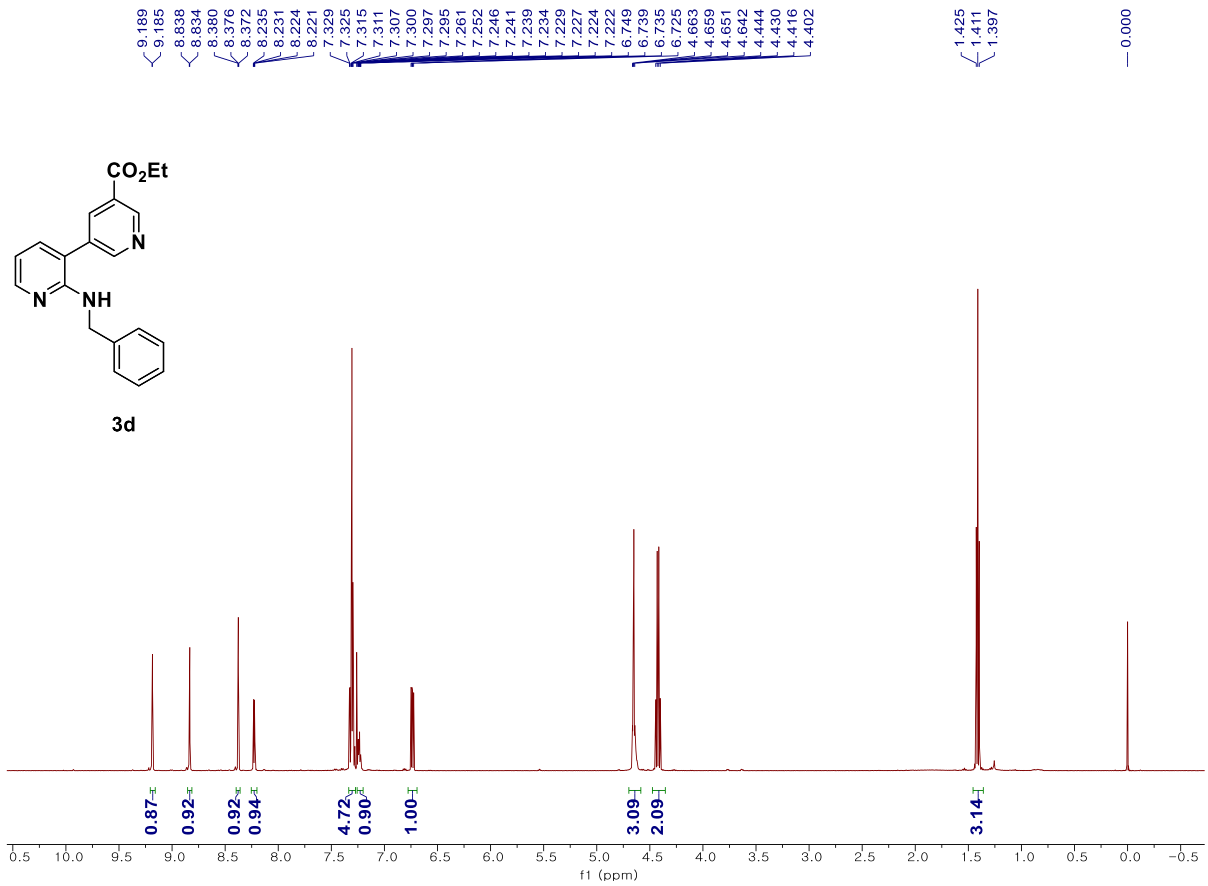
9.189
9.185
8.838
8.834
8.380
8.376
8.372
8.235
8.231
8.224
8.221
7.329
7.325
7.315
7.311
7.307
7.300
7.297
7.295
7.261
7.252
7.246
7.241
7.239
7.234
7.229
7.227
7.224
7.222
6.749
6.739
6.735
6.725
4.663
4.659
4.651
4.642
4.444
4.430
4.416
4.402
1.425
1.411
1.397
0.000
CO2Et
N
N
NH
3d
0.87
0.92
0.92
0.94
4.72
0.90
1.00
3.09
2.09
3.14
f1 (ppm)

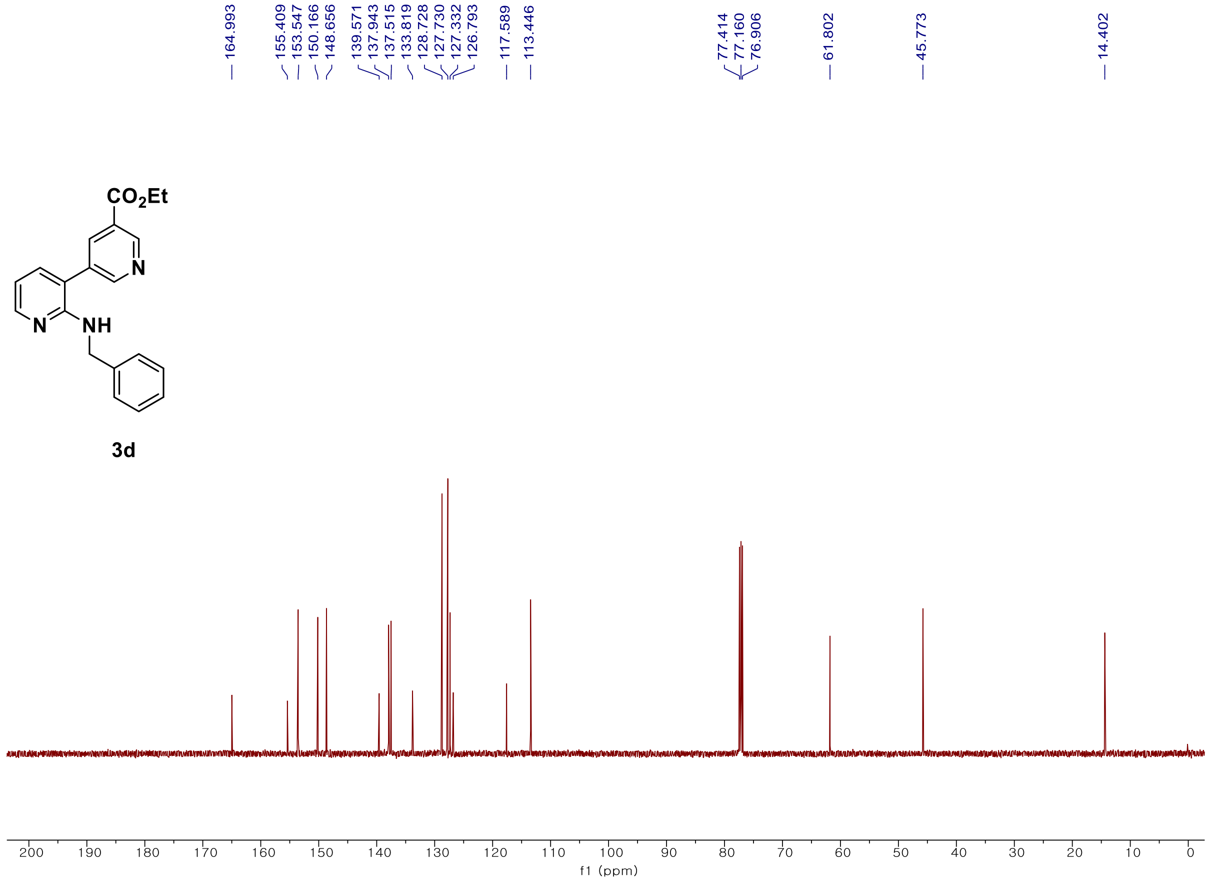
164.993
155.409
153.547
150.166
148.656
139.571
137.943
137.515
133.819
128.728
127.730
127.332
126.793
117.589
113.446
77.414
77.160
76.906
61.802
45.773
14.402
CO2Et
N
N
NH
3d
f1 (ppm)

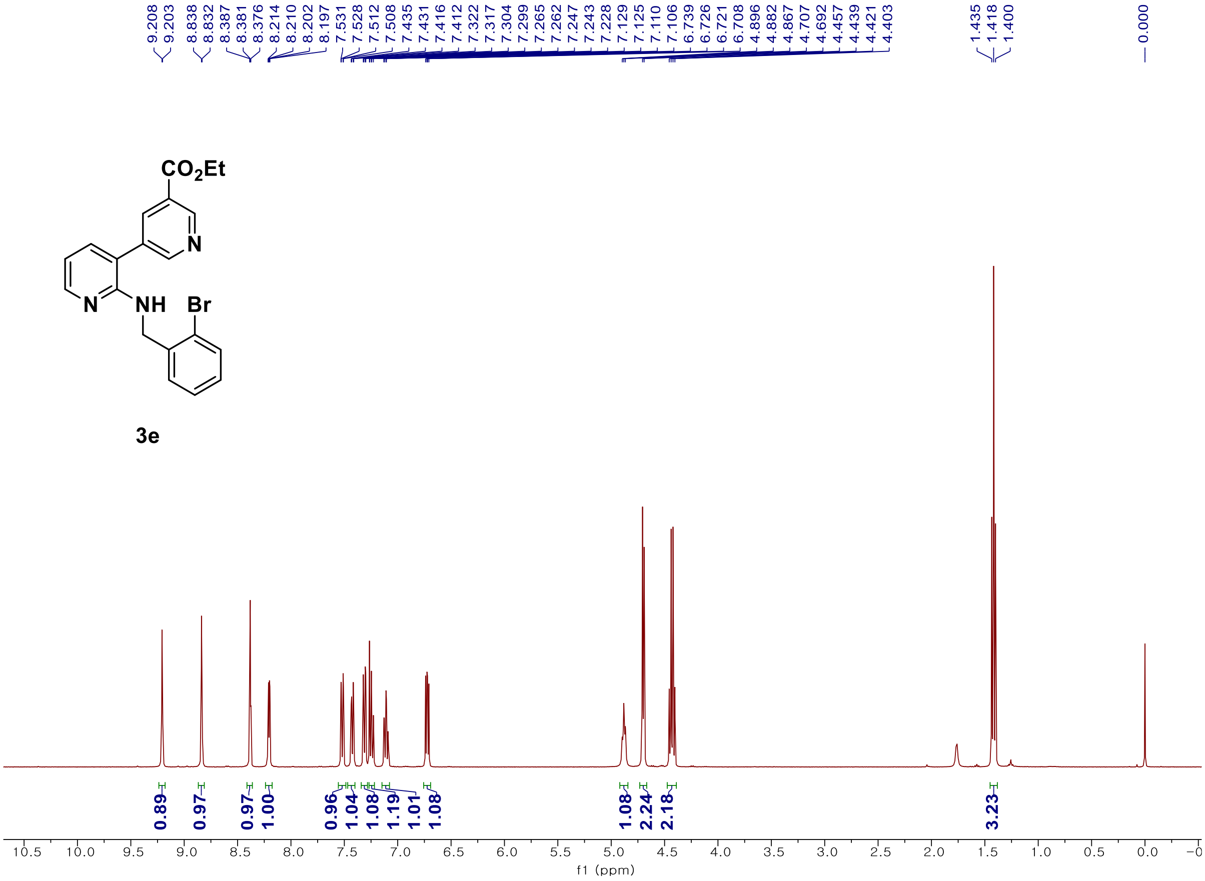
9.208
9.203
8.838
8.832
8.387
8.381
8.376
8.214
8.210
8.202
8.197
7.531
7.528
7.512
7.508
7.435
7.431
7.416
7.412
7.322
7.317
7.304
7.299
7.265
7.262
7.247
7.243
7.228
7.129
7.125
7.110
7.106
6.739
6.726
6.721
6.708
4.896
4.882
4.867
4.707
4.692
4.457
4.439
4.421
4.403
1.435
1.418
1.400
0.000
CO_2Et
N
N
NH
Br
3e
0.89
0.97
0.97
1.00
0.96
1.04
1.08
1.19
1.01
1.08
1.08
2.24
2.18
3.23
f1 (ppm)

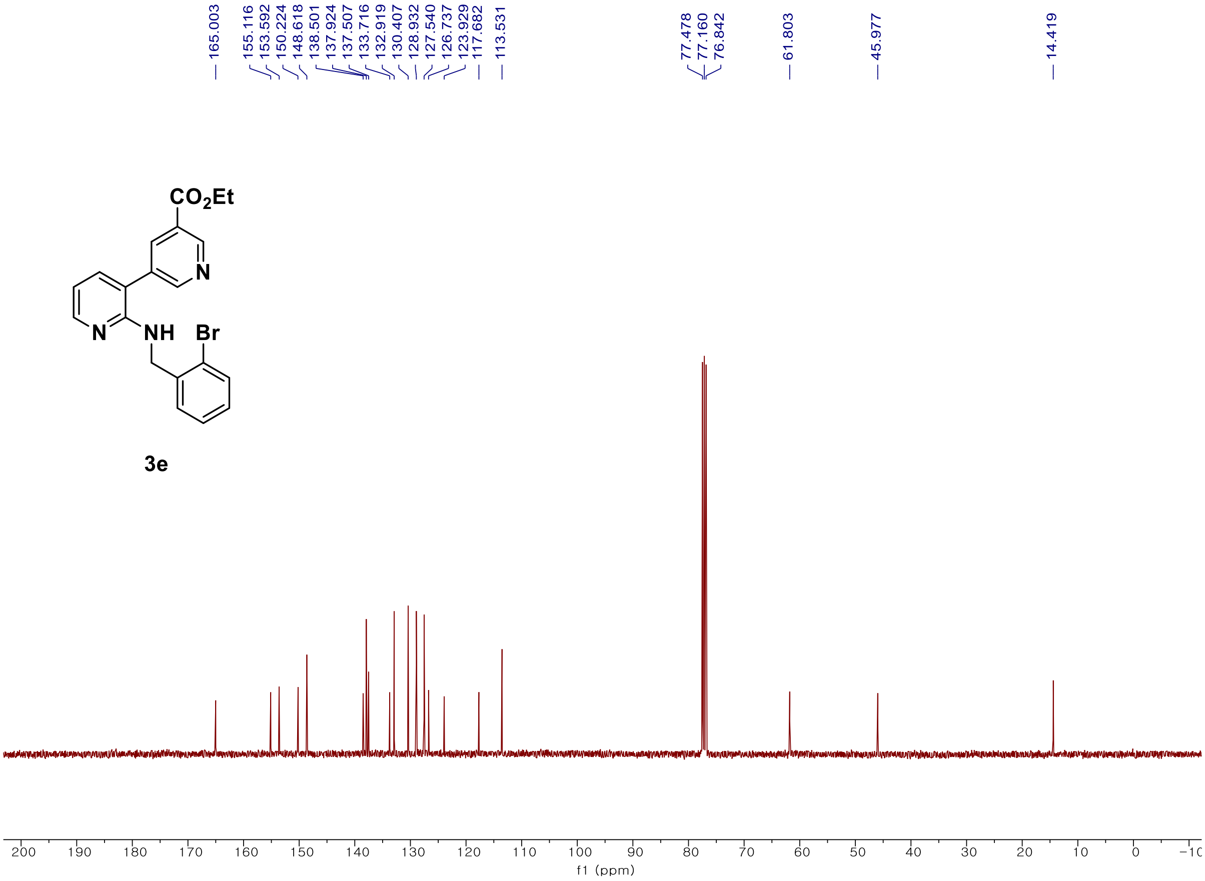
165.003
155.116
153.592
150.224
148.618
138.501
137.924
137.507
133.716
132.919
130.407
128.932
127.540
126.737
123.929
117.682
113.531
77.478
77.160
76.842
61.803
45.977
14.419
CO_2Et
N
N
NH
Br
3e
f1 (ppm)

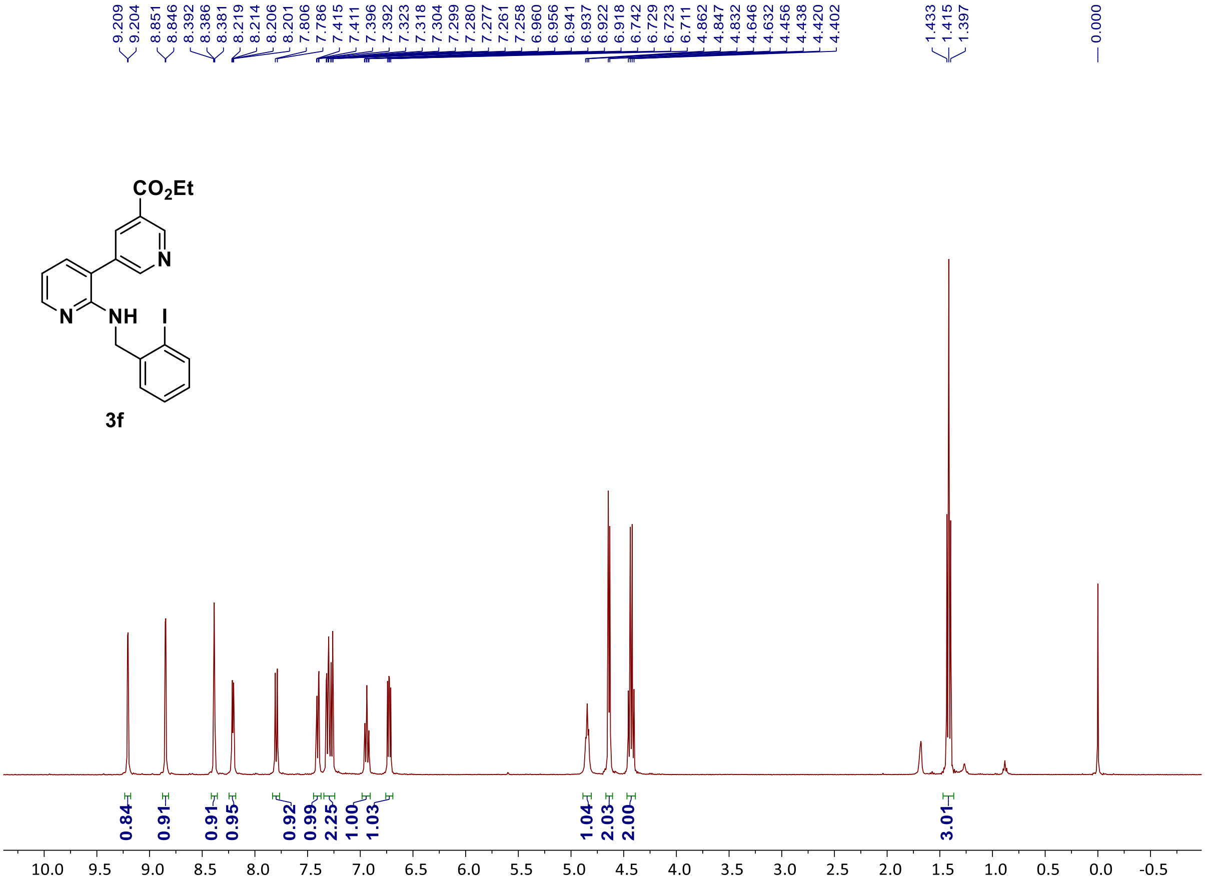
9.209
9.204
8.851
8.846
8.392
8.386
8.381
8.219
8.214
8.206
8.201
7.806
7.786
7.415
7.411
7.396
7.392
7.323
7.318
7.304
7.299
7.280
7.277
7.261
7.258
6.960
6.956
6.941
6.937
6.922
6.918
6.742
6.729
6.723
6.711
4.862
4.847
4.832
4.646
4.632
4.456
4.438
4.420
4.402
1.433
1.415
1.397
0.000
CO_2Et
N
N
NH
I
3f
0.84
0.91
0.91
0.95
0.92
0.99
2.25
1.00
1.03
1.04
2.03
2.00
3.01
10.0 9.5 9.0 8.5 8.0 7.5 7.0 6.5 6.0 5.5 5.0 4.5 4.0 3.5 3.0 2.5 2.0 1.5 1.0 0.5 0.0 -0.5

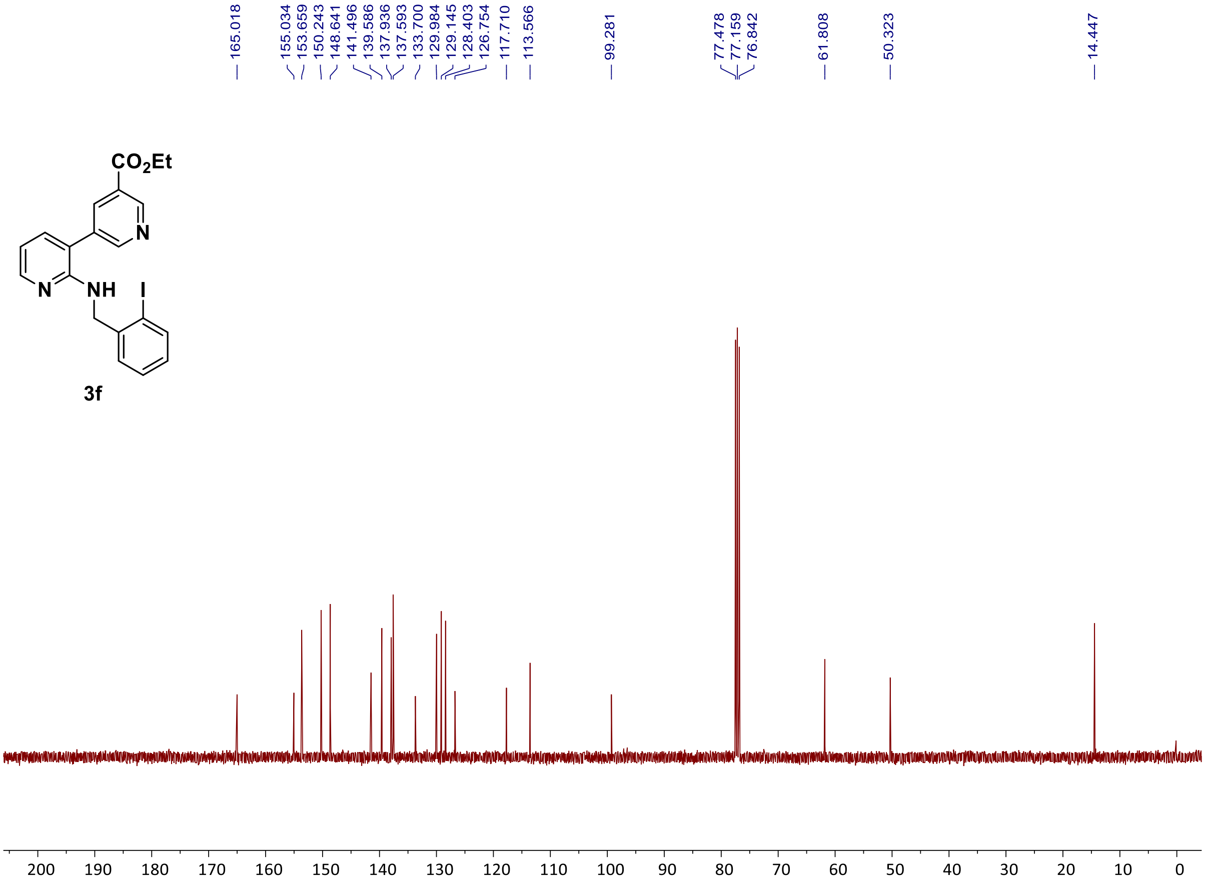
165.018
155.034
153.659
150.243
148.641
141.496
139.586
137.936
137.593
133.700
129.984
129.145
128.403
126.754
117.710
113.566
99.281
77.478
77.159
76.842
61.808
50.323
14.447
CO_2Et
N
N
NH
I
3f
200 190 180 170 160 150 140 130 120 110 100 90 80 70 60 50 40 30 20 10 0

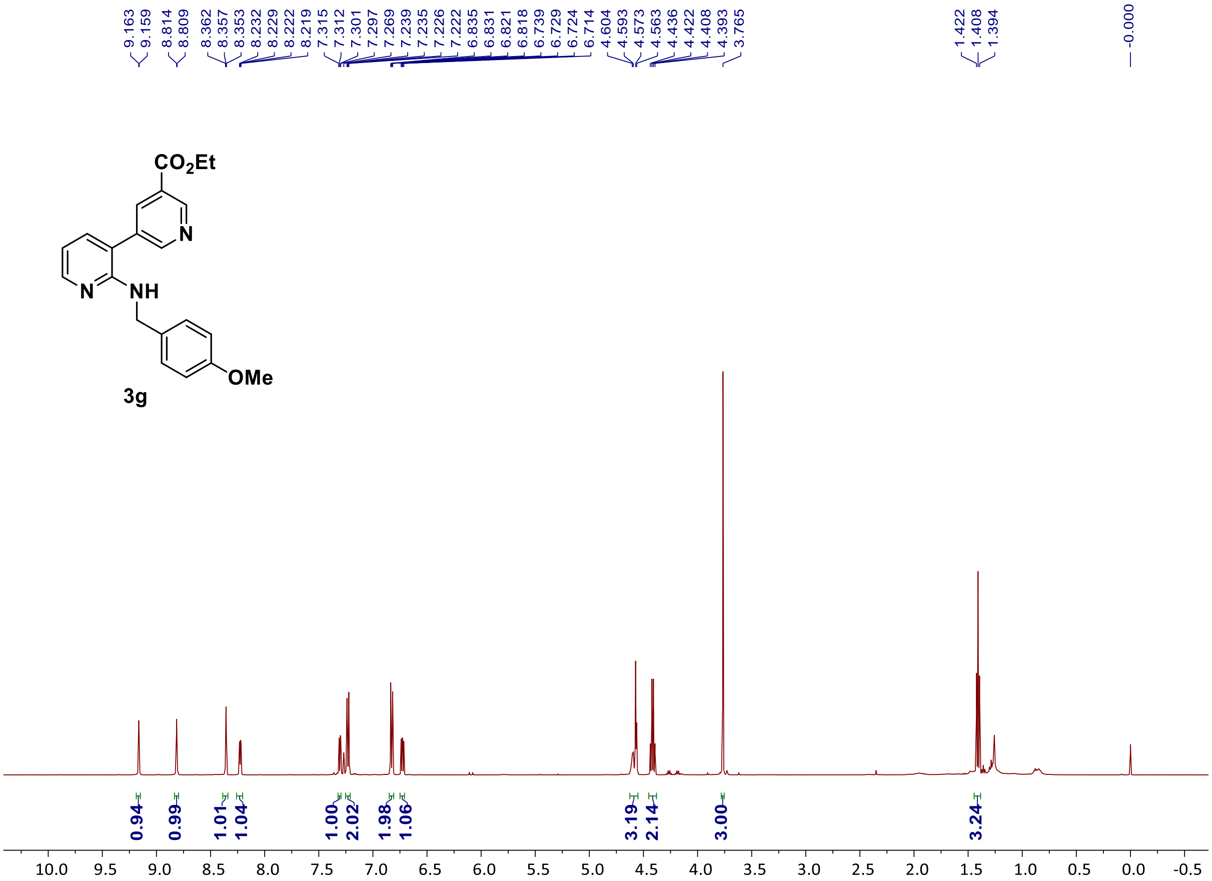
9.163 9.159 8.814 8.809 8.362 8.357 8.353 8.232 8.229 8.222 8.219 7.315 7.312 7.301 7.297 7.269 7.239 7.235 7.226 7.222 6.835 6.831 6.821 6.818 6.739 6.729 6.724 6.714 4.604 4.593 4.573 4.563 4.436 4.422 4.408 4.393 3.765
1.422 1.408 1.394
-0.000
CO2Et
N
NH
OMe
3g

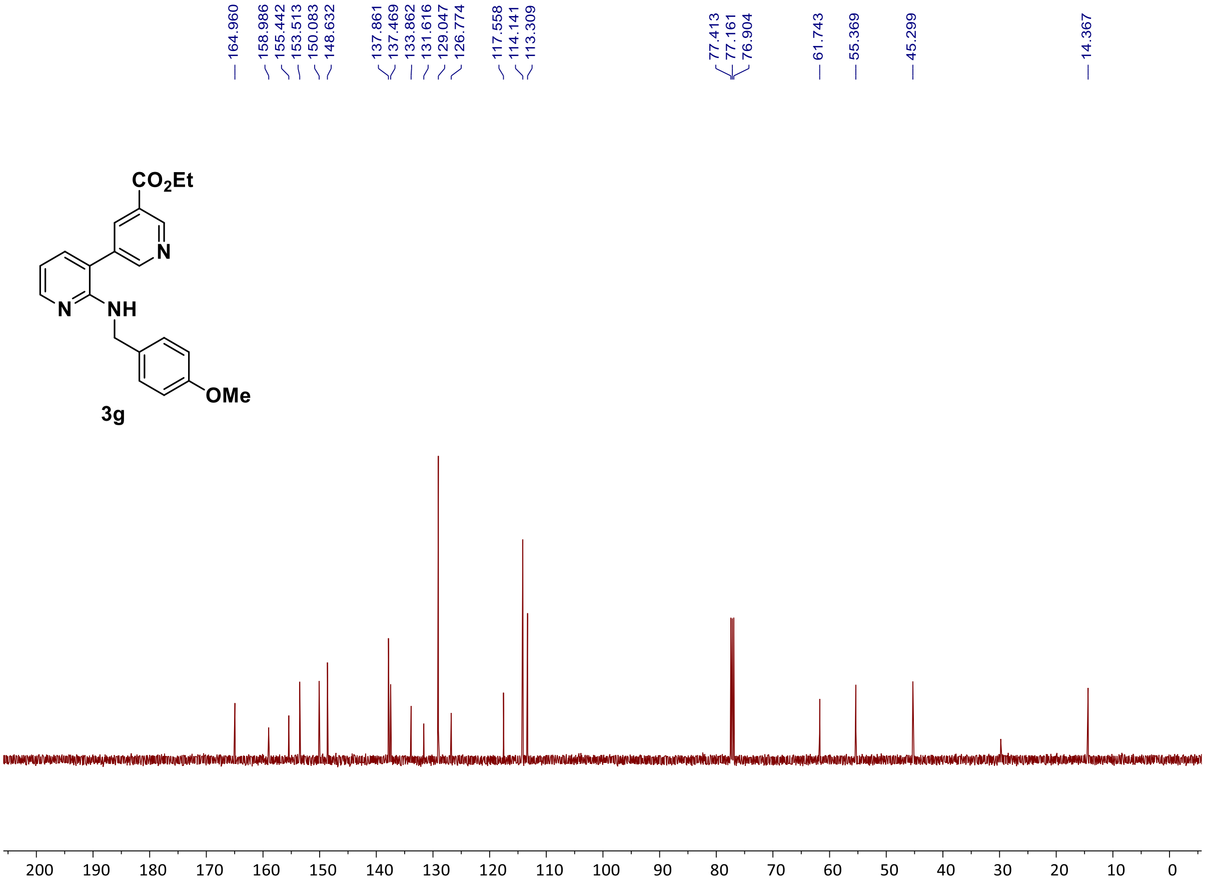
164.960 158.986 155.442 153.513 150.083 148.632 137.861 137.469 133.862 131.616 129.047 126.774 117.558 114.141 113.309
77.413 77.161 76.904
61.743
55.369
45.299
14.367
CO2Et
N
NH
OMe
3g

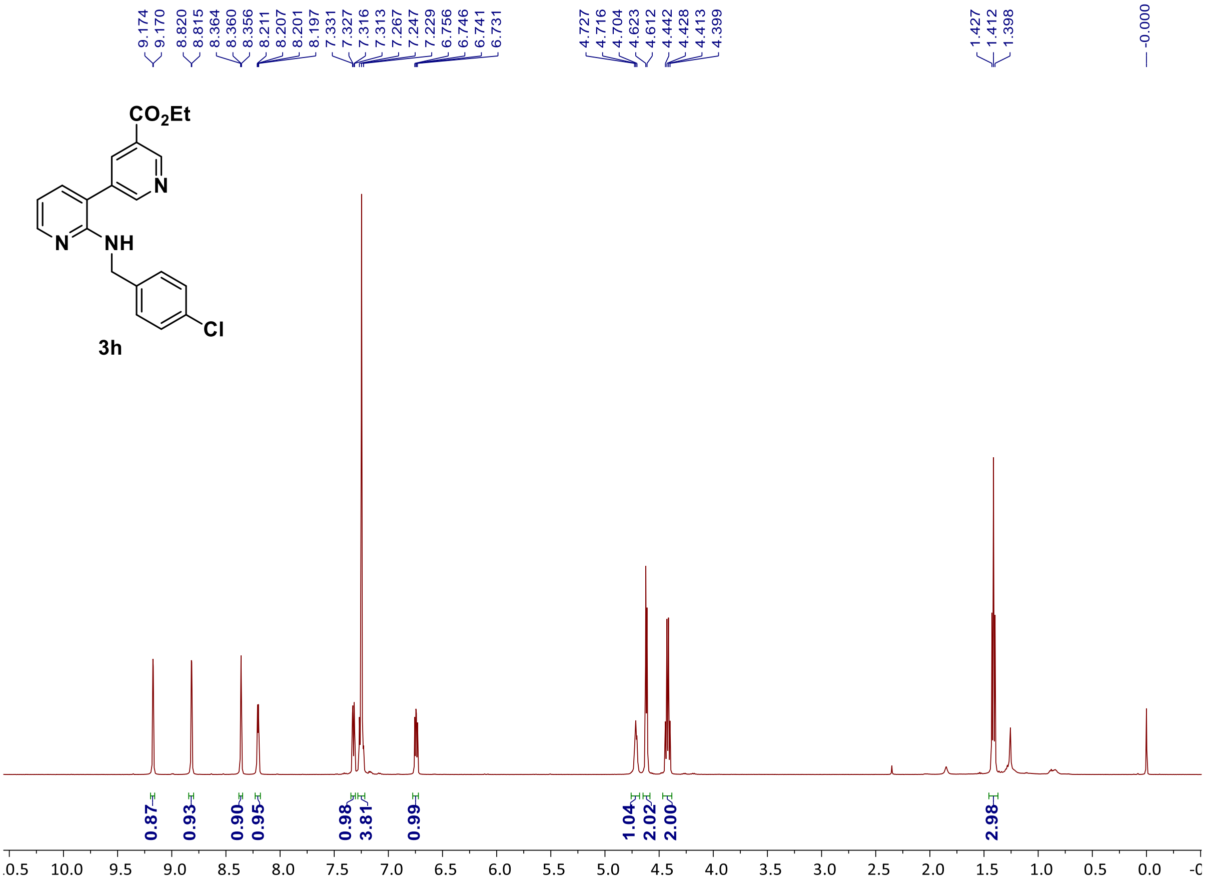
9.174
9.170
8.820
8.815
8.364
8.360
8.356
8.211
8.207
8.201
8.197
7.331
7.327
7.316
7.313
7.267
7.247
7.229
6.756
6.746
6.741
6.731
4.727
4.716
4.704
4.623
4.612
4.442
4.428
4.413
4.399
1.427
1.412
1.398
-0.000
CO2Et
N
N
NH
Cl
3h
0.87
0.93
0.90
0.95
0.98
3.81
0.99
1.04
2.02
2.00
2.98
10.5 10.0 9.5 9.0 8.5 8.0 7.5 7.0 6.5 6.0 5.5 5.0 4.5 4.0 3.5 3.0 2.5 2.0 1.5 1.0 0.5 0.0

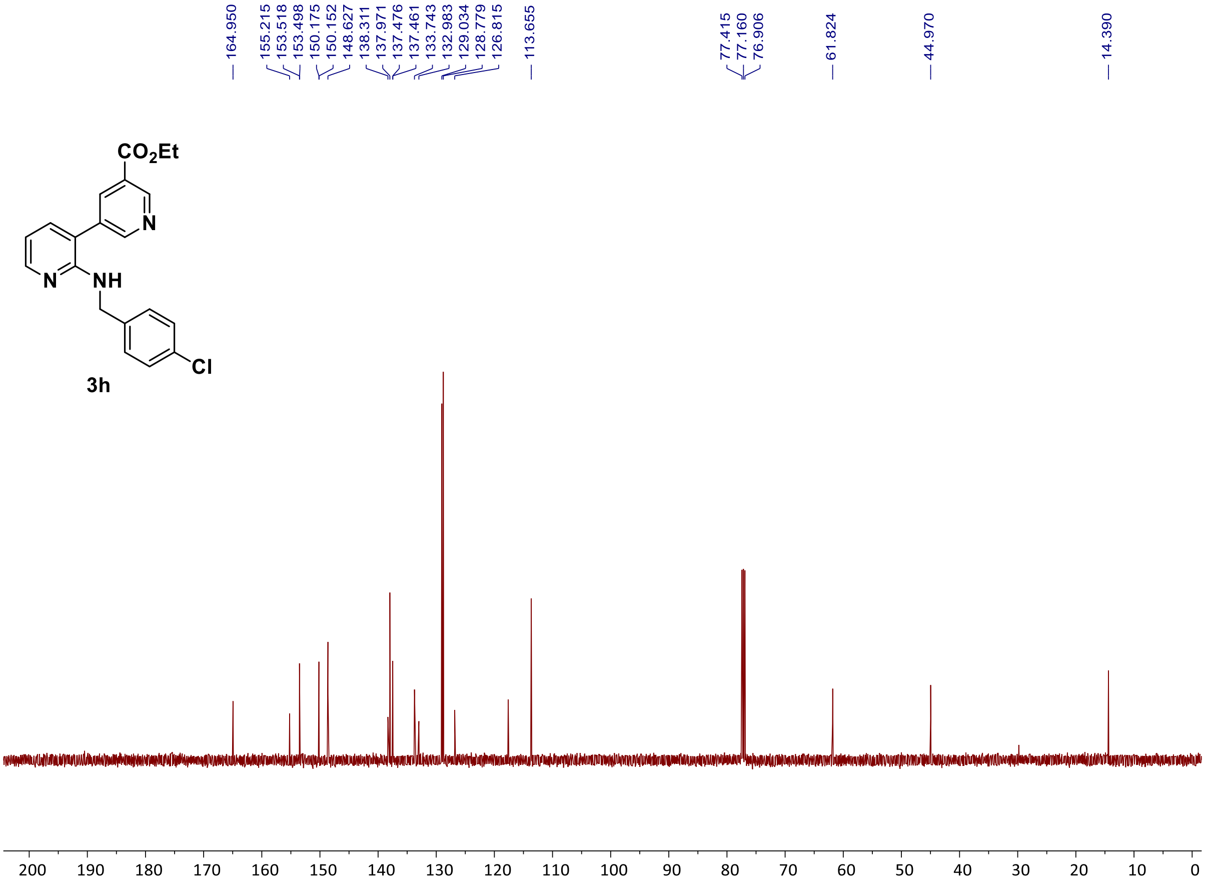
164.950
155.215
153.518
153.498
150.175
150.152
148.627
138.311
137.971
137.476
137.461
133.743
132.983
129.034
128.779
126.815
113.655
77.415
77.160
76.906
61.824
44.970
14.390
CO2Et
N
N
NH
Cl
3h
200 190 180 170 160 150 140 130 120 110 100 90 80 70 60 50 40 30 20 10 0

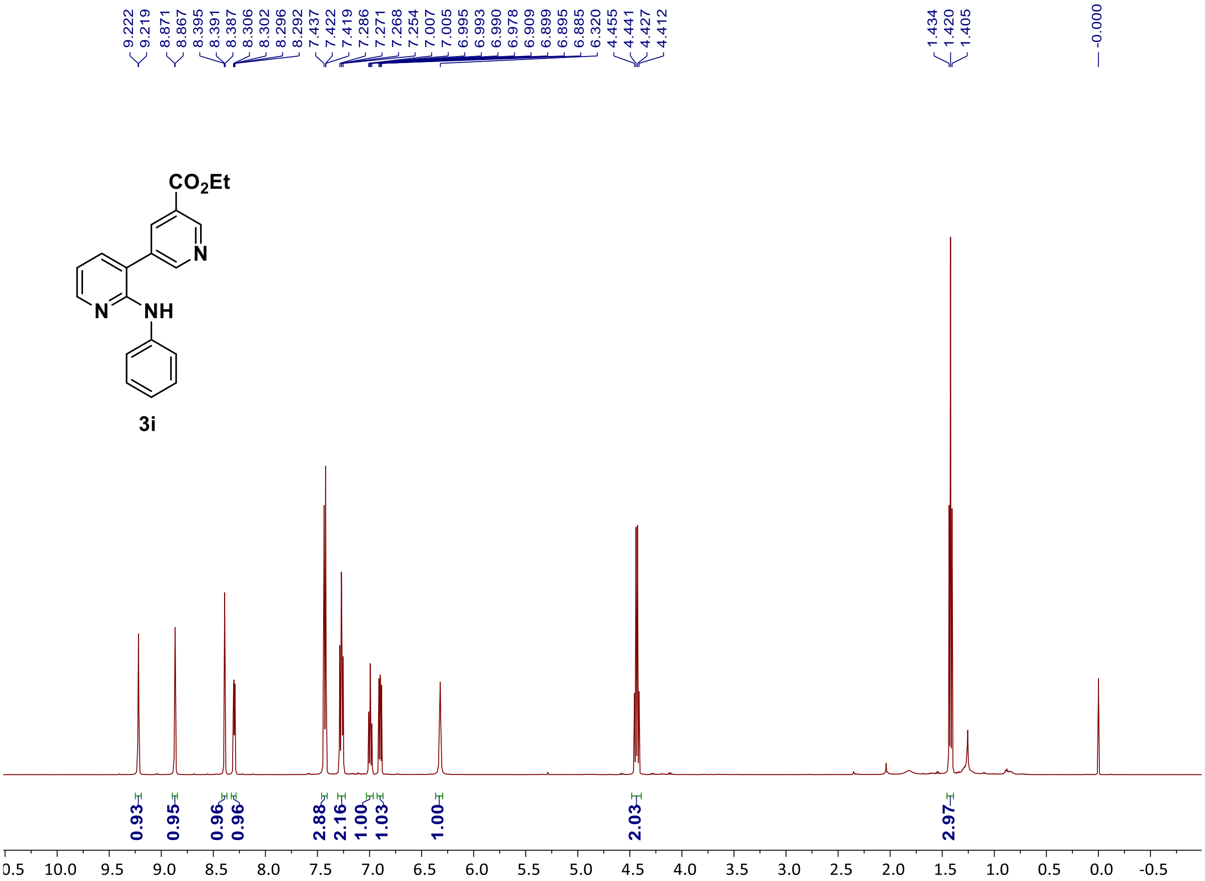
9.222
9.219
8.871
8.867
8.395
8.391
8.387
8.306
8.302
8.296
8.292
7.437
7.422
7.419
7.286
7.271
7.268
7.254
7.007
7.005
6.995
6.993
6.990
6.978
6.909
6.899
6.895
6.885
6.320
4.455
4.441
4.427
4.412
1.434
1.420
1.405
-0.000
CO_2Et
N
N
NH
3i
0.93
0.95
0.96
0.96
2.88
2.16
1.00
1.03
1.00
2.03
2.97
0.5 10.0 9.5 9.0 8.5 8.0 7.5 7.0 6.5 6.0 5.5 5.0 4.5 4.0 3.5 3.0 2.5 2.0 1.5 1.0 0.5 0.0 -0.5

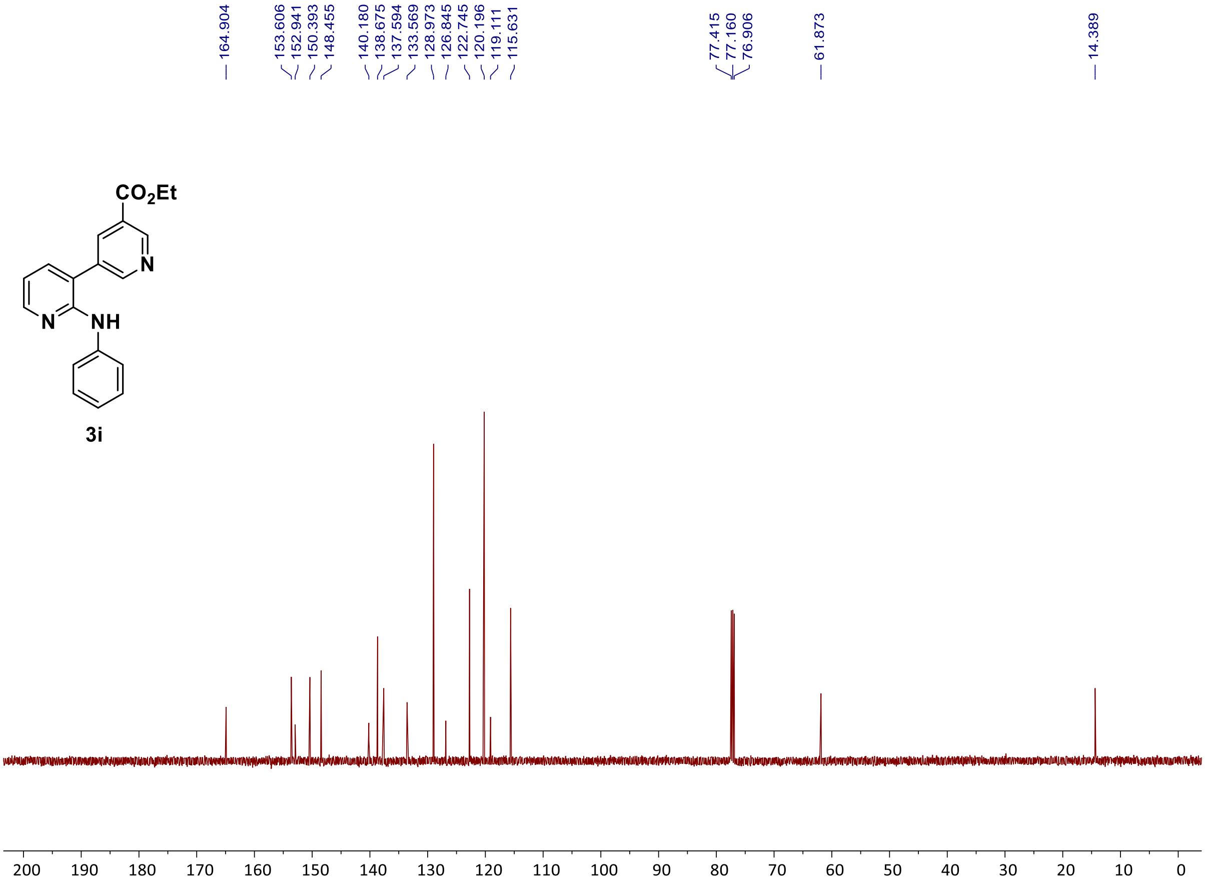
164.904
153.606
152.941
150.393
148.455
140.180
138.675
137.594
133.569
128.973
126.845
122.745
120.196
119.111
115.631
77.415
77.160
76.906
61.873
14.389
CO_2Et
N
N
NH
3i
200 190 180 170 160 150 140 130 120 110 100 90 80 70 60 50 40 30 20 10 0

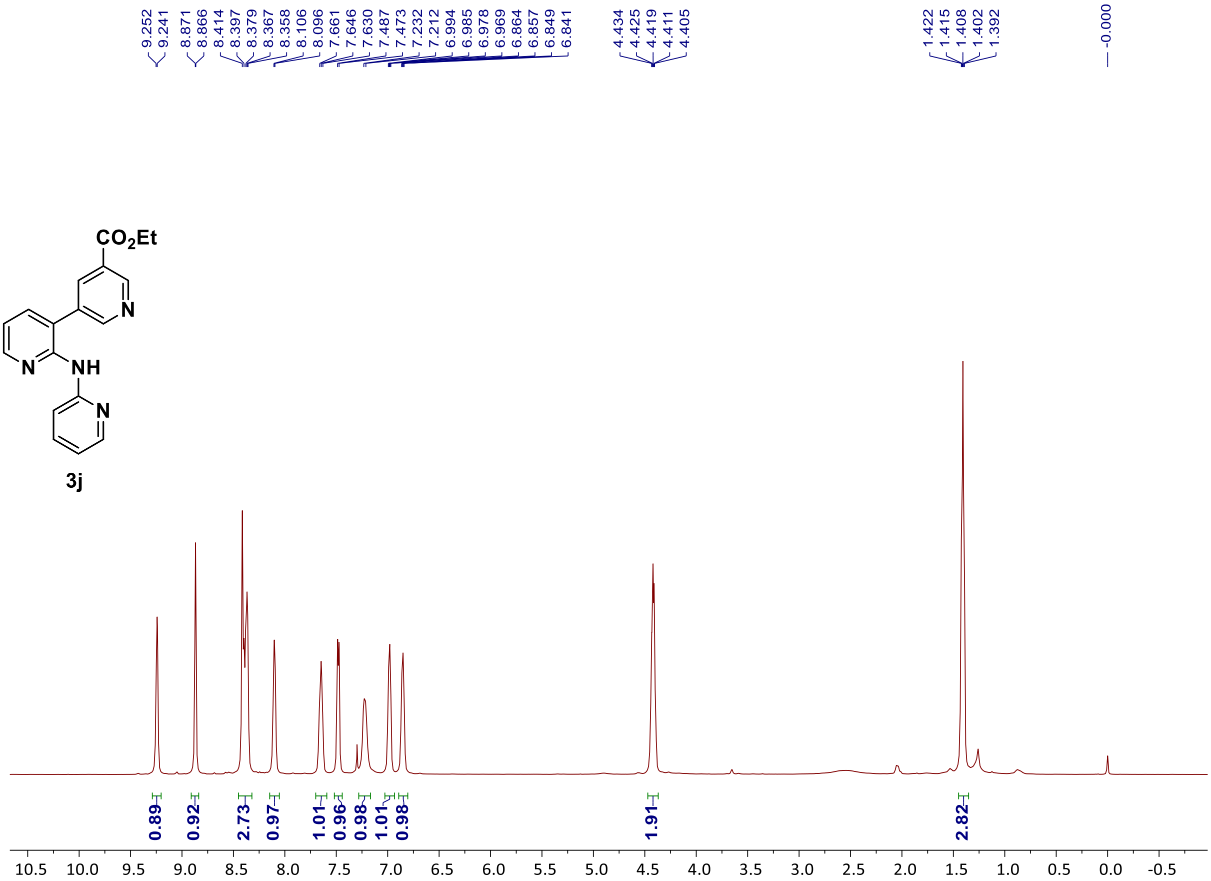
9.252
9.241
8.871
8.866
8.414
8.397
8.379
8.367
8.358
8.106
8.096
7.661
7.646
7.630
7.487
7.473
7.232
7.212
6.994
6.985
6.978
6.969
6.864
6.857
6.849
6.841
4.434
4.425
4.419
4.411
4.405
1.422
1.415
1.408
1.402
1.392
-0.000
CO2Et
N
N
NH
N
3j
0.89
0.92
2.73
0.97
1.01
0.96
0.98
1.01
0.98
1.91
2.82
10.5 10.0 9.5 9.0 8.5 8.0 7.5 7.0 6.5 6.0 5.5 5.0 4.5 4.0 3.5 3.0 2.5 2.0 1.5 1.0 0.5 0.0 -0.5

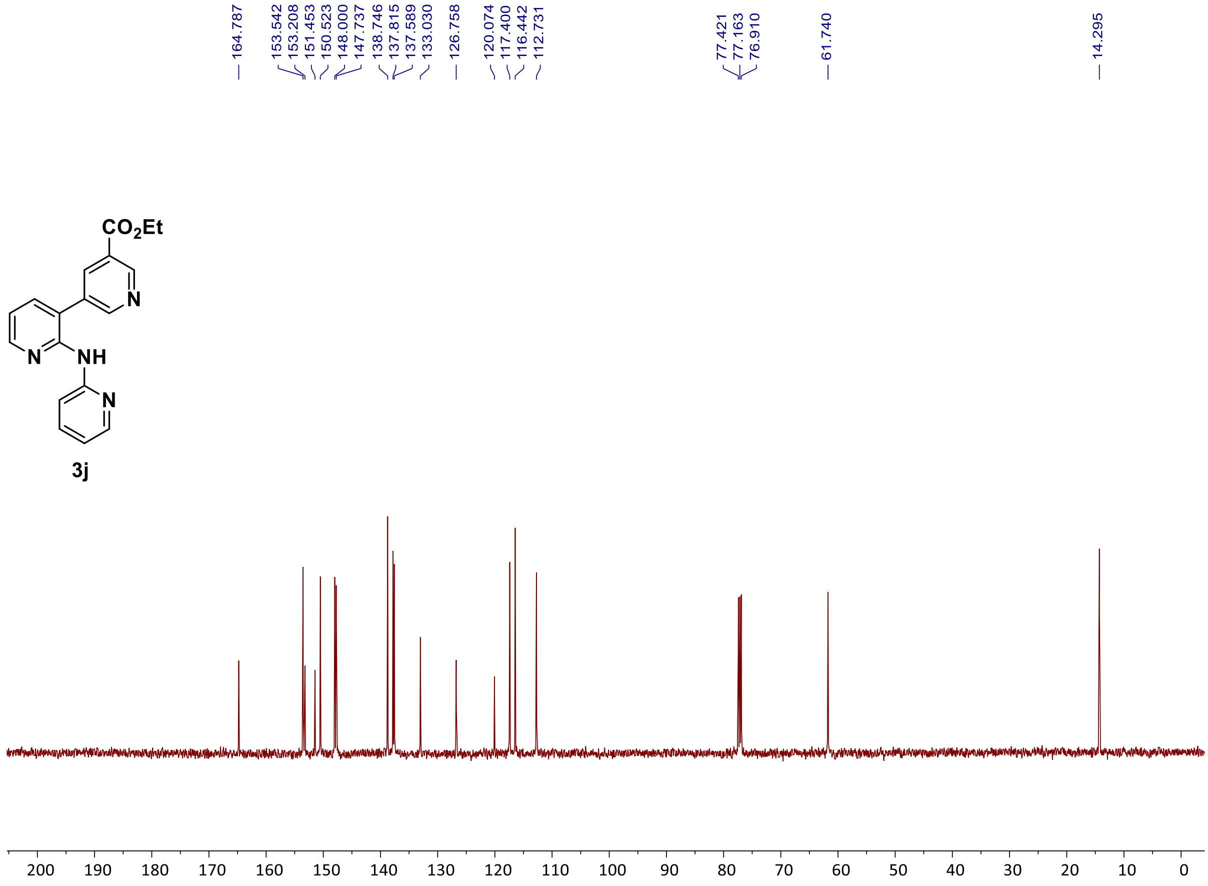
164.787
153.542
153.208
151.453
150.523
148.000
147.737
138.746
137.815
137.589
133.030
126.758
120.074
117.400
116.442
112.731
77.421
77.163
76.910
61.740
14.295
CO2Et
N
N
NH
N
3j
200 190 180 170 160 150 140 130 120 110 100 90 80 70 60 50 40 30 20 10 0

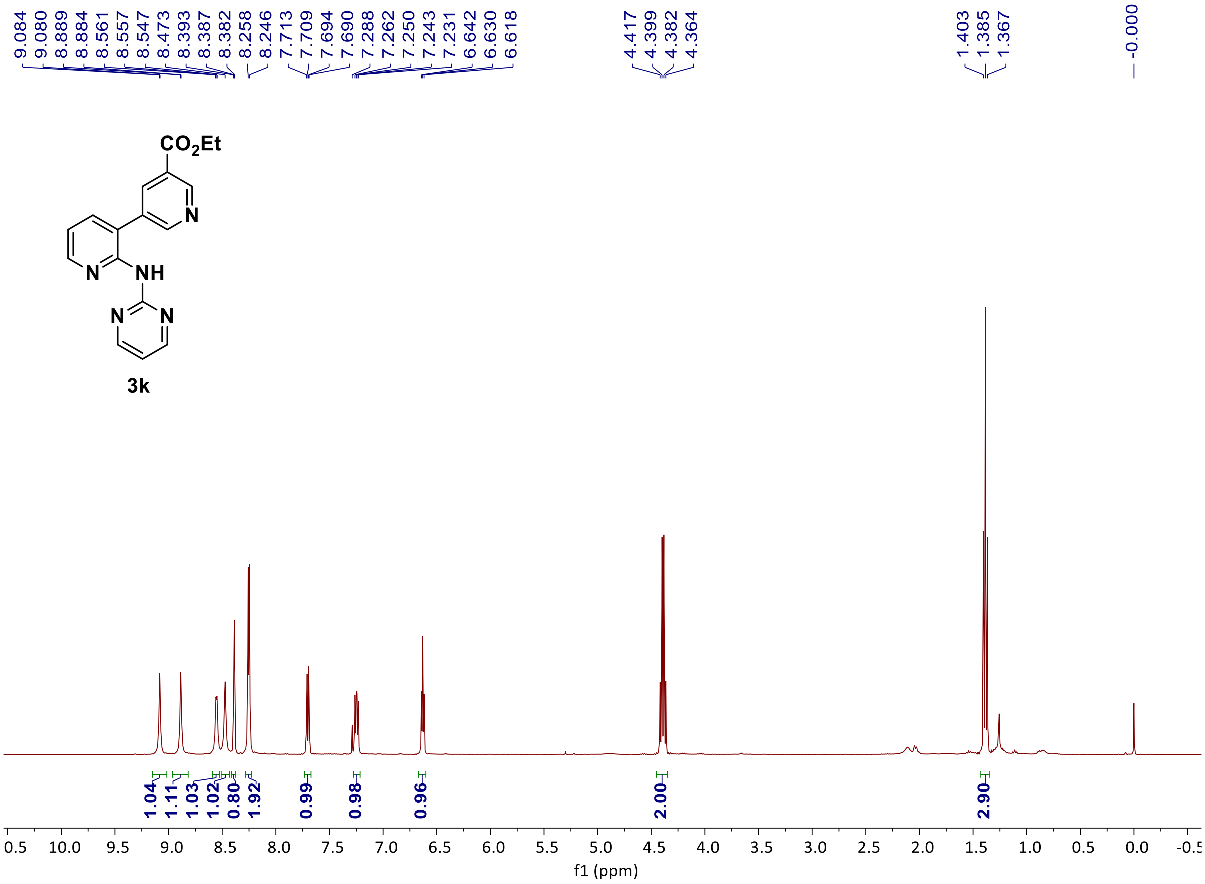
9.084 9.080 8.889 8.884 8.561 8.557 8.547 8.473 8.393 8.387 8.382 8.258 8.246 7.713 7.709 7.694 7.690 7.288 7.262 7.250 7.243 7.231 6.642 6.630 6.618
4.417 4.399 4.382 4.364
1.403 1.385 1.367
-0.000
CO2Et
N
N
NH
N
N
3k
1.04 1.11 1.03 1.02 0.80 1.92 0.99 0.98 0.96 2.00 2.90
f1 (ppm)

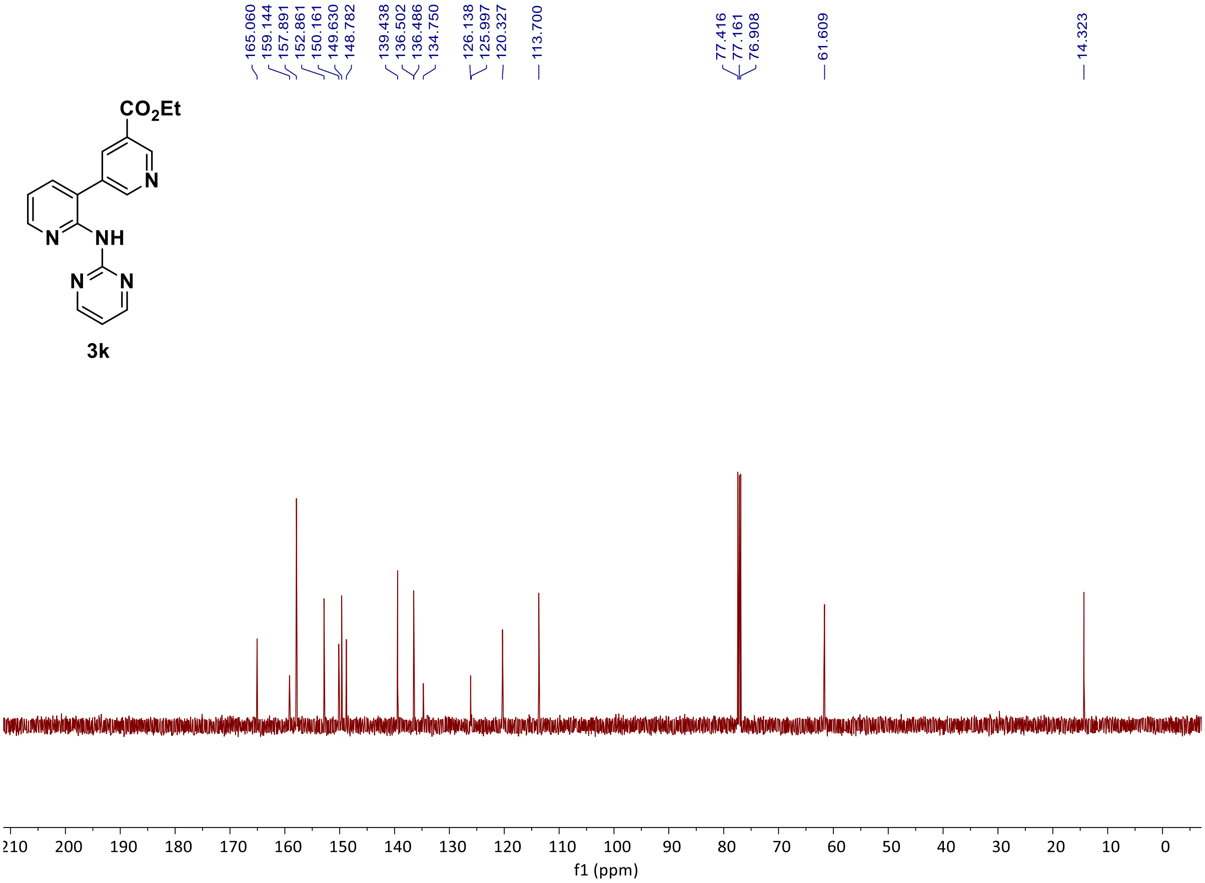
165.060 159.144 157.891 152.861 150.161 149.630 148.782 139.438 136.502 136.486 134.750 126.138 125.997 120.327 113.700
77.416 77.161 76.908
61.609
14.323
CO2Et
N
N
NH
N
N
3k
f1 (ppm)

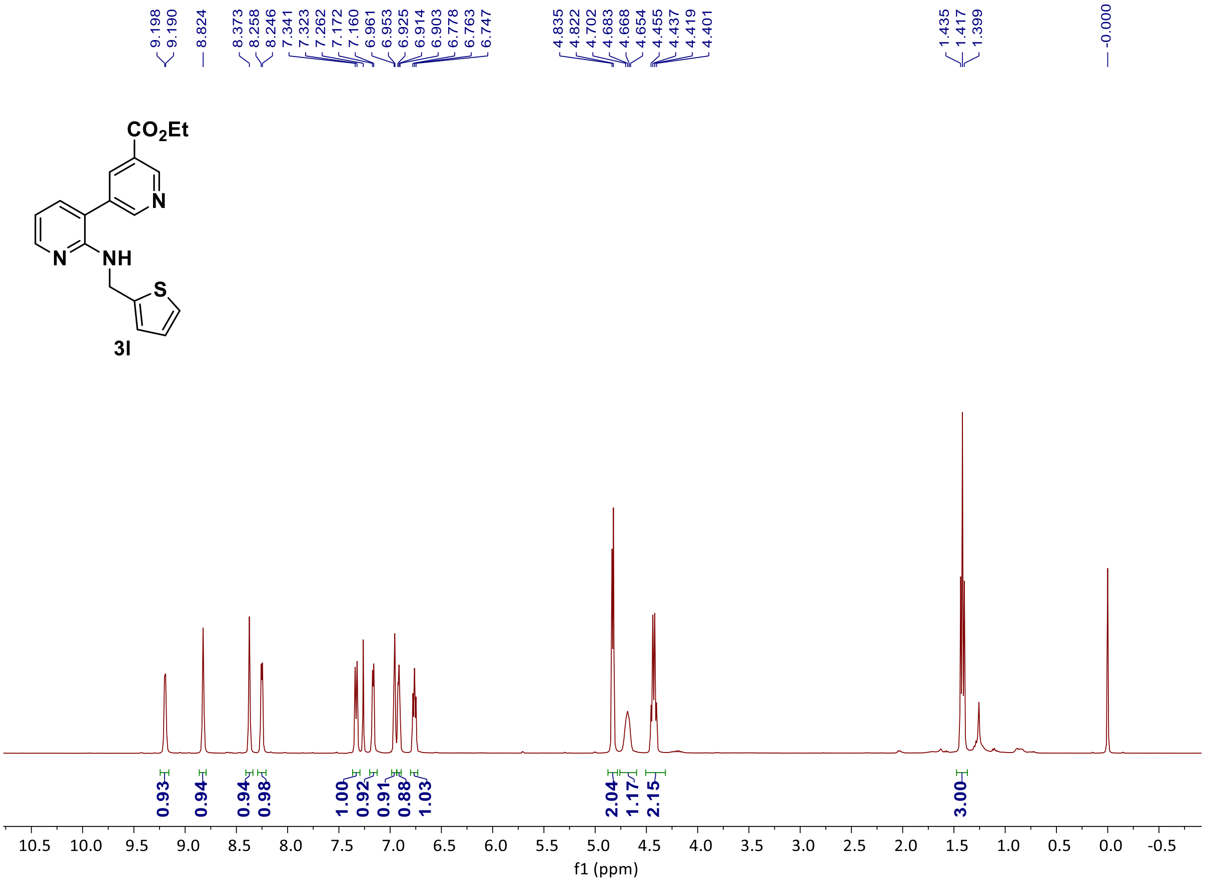
9.198
9.190
8.824
8.373
8.258
8.246
7.341
7.323
7.262
7.172
7.160
6.961
6.953
6.925
6.914
6.903
6.778
6.763
6.747
4.835
4.822
4.702
4.683
4.668
4.654
4.455
4.437
4.419
4.401
1.435
1.417
1.399
-0.000
CO_2Et
N
N
NH
S
3l
0.93
0.94
0.94
0.98
1.00
0.92
0.91
0.88
1.03
2.04
1.17
2.15
3.00
10.5 10.0 9.5 9.0 8.5 8.0 7.5 7.0 6.5 6.0 5.5 5.0 4.5 4.0 3.5 3.0 2.5 2.0 1.5 1.0 0.5 0.0 -0.5
f1 (ppm)

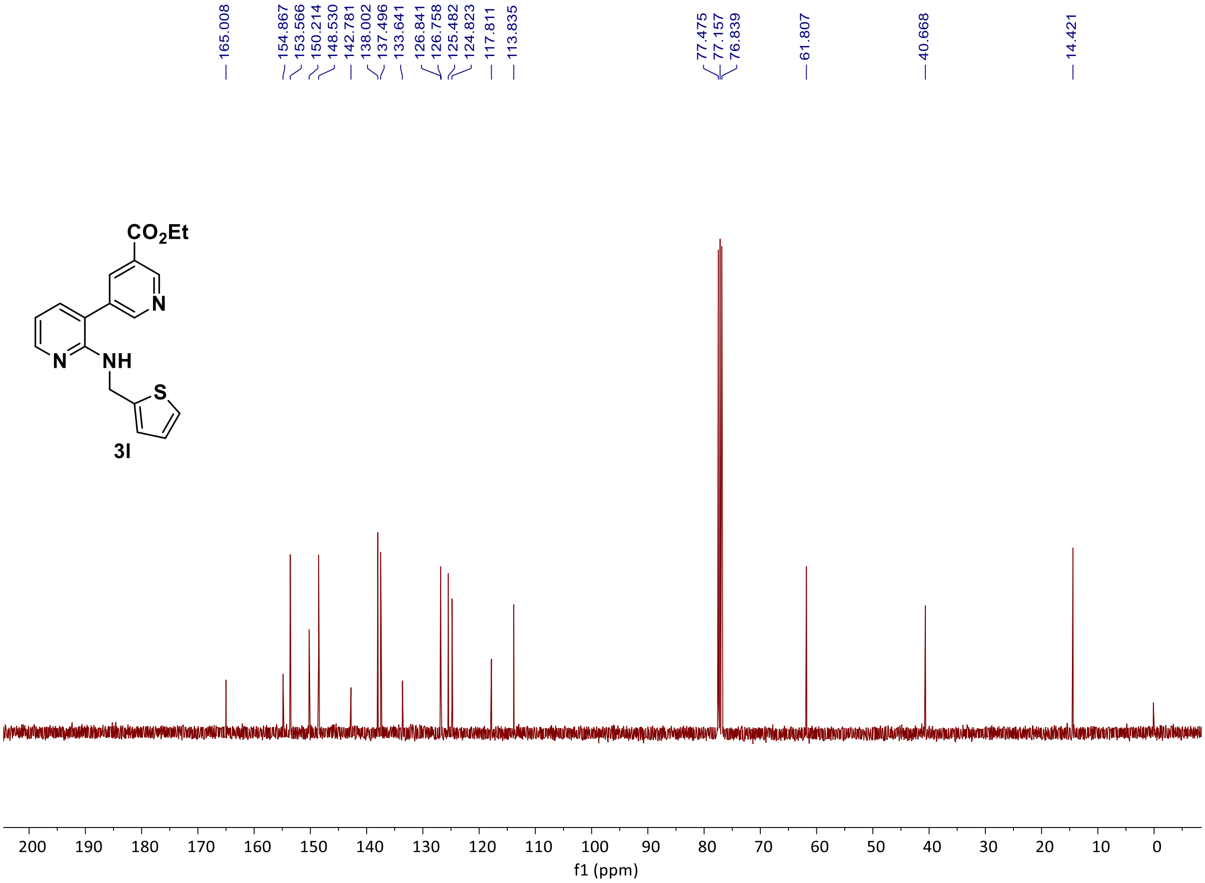
165.008
154.867
153.566
150.214
148.530
142.781
138.002
137.496
133.641
126.841
126.758
125.482
124.823
117.811
113.835
77.475
77.157
76.839
61.807
40.668
14.421
CO_2Et
N
N
NH
S
3l
200 190 180 170 160 150 140 130 120 110 100 90 80 70 60 50 40 30 20 10 0
f1 (ppm)

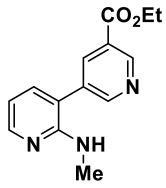

3m

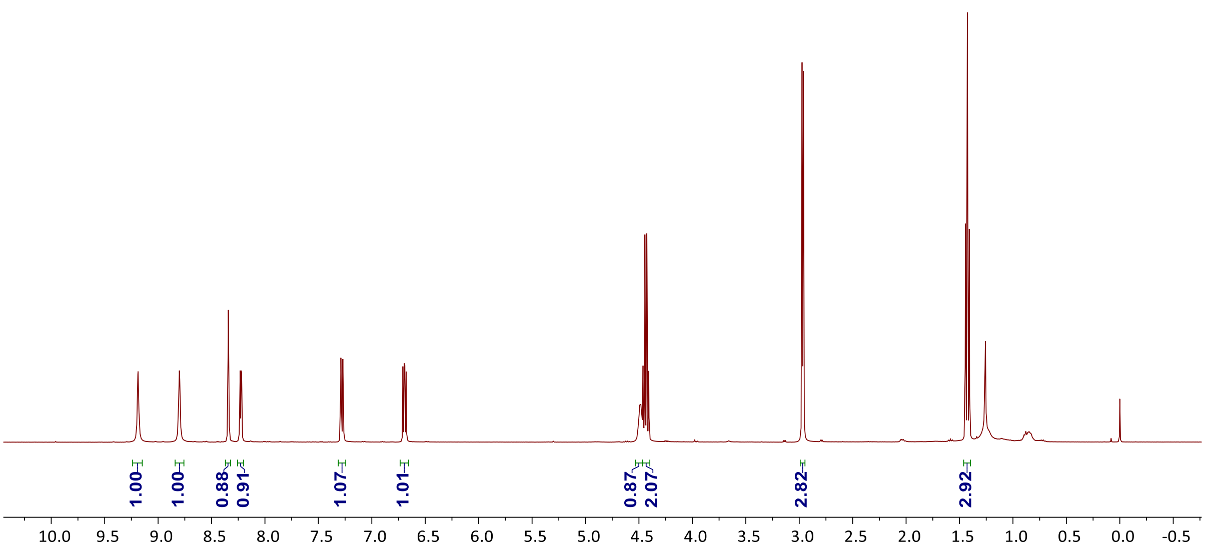
9.187
8.801
8.348
8.343
8.337
8.236
8.231
8.223
8.219
7.289
7.285
7.271
7.267
6.709
6.696
6.691
6.678
4.493
4.481
4.462
4.444
4.426
4.408
2.973
2.961
1.445
1.427
1.409
0.000
1.00
1.00
0.88
0.91
1.07
1.01
0.87
2.07
2.82
2.92
10.0 9.5 9.0 8.5 8.0 7.5 7.0 6.5 6.0 5.5 5.0 4.5 4.0 3.5 3.0 2.5 2.0 1.5 1.0 0.5 0.0 -0.5

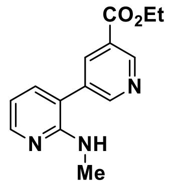

3m

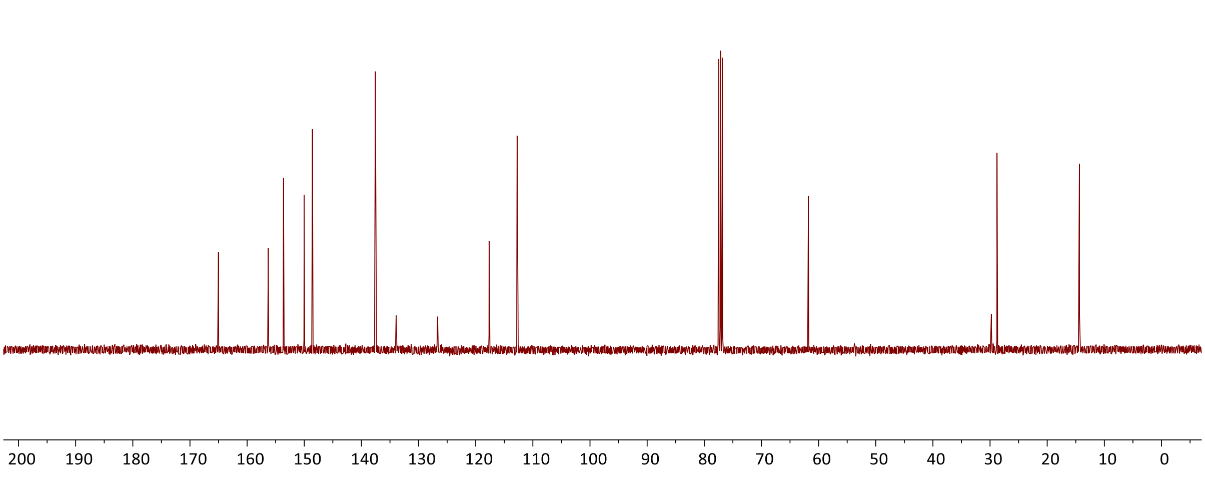
165.015
156.291
153.628
150.019
148.572
137.554
137.510
133.925
126.666
117.657
112.754
77.479
77.160
76.842
61.782
28.777
14.364
200 190 180 170 160 150 140 130 120 110 100 90 80 70 60 50 40 30 20 10 0

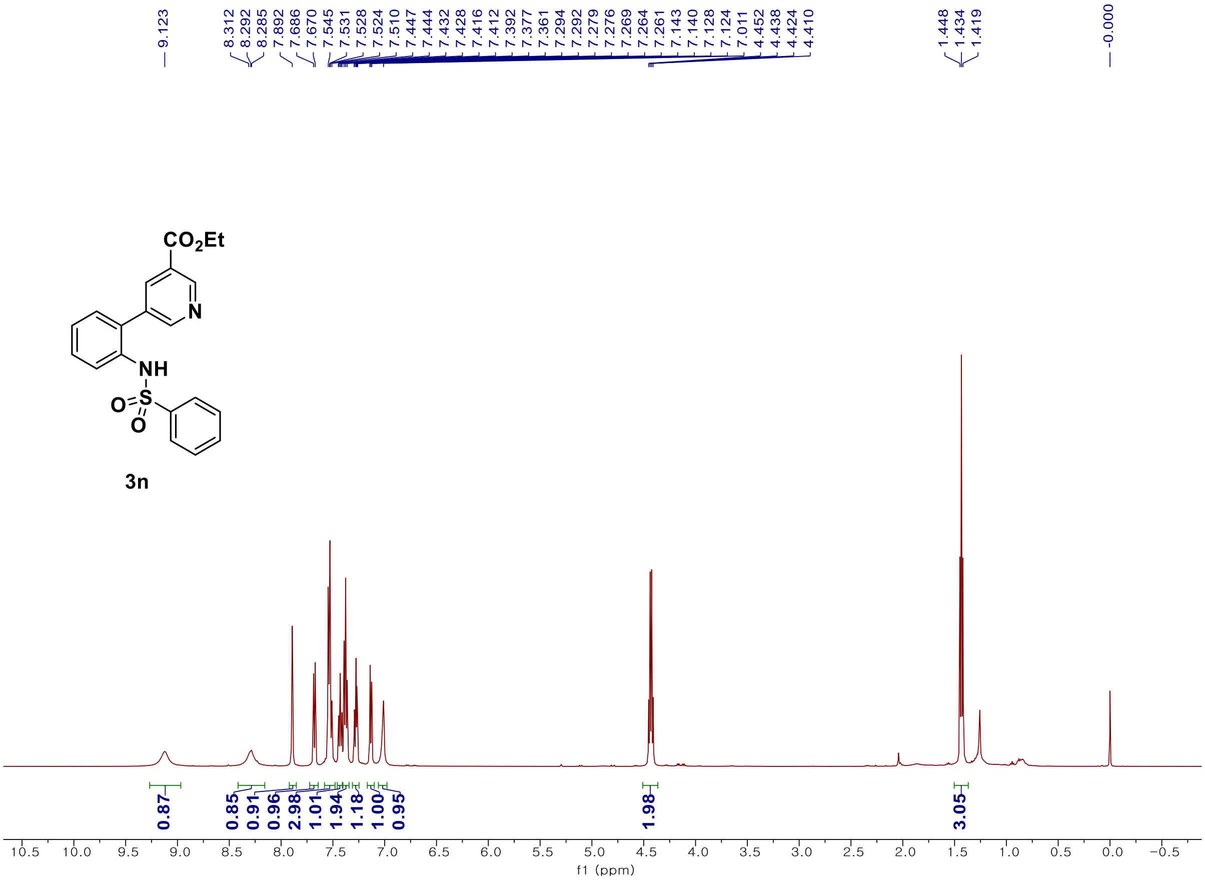
9.123
8.312
8.292
8.285
7.892
7.686
7.670
7.545
7.531
7.528
7.524
7.510
7.447
7.444
7.432
7.428
7.416
7.412
7.392
7.377
7.361
7.294
7.292
7.279
7.276
7.269
7.264
7.261
7.143
7.140
7.128
7.124
7.011
4.452
4.438
4.424
4.410
1.448
1.434
1.419
-0.000
CO2Et
N
NH
O=S
O
3n
0.87
0.85
0.91
0.96
2.98
1.01
1.94
1.18
1.00
0.95
1.98
3.05
10.5 10.0 9.5 9.0 8.5 8.0 7.5 7.0 6.5 6.0 5.5 5.0 4.5 4.0 3.5 3.0 2.5 2.0 1.5 1.0 0.5 0.0 -0.5
f1 (ppm)

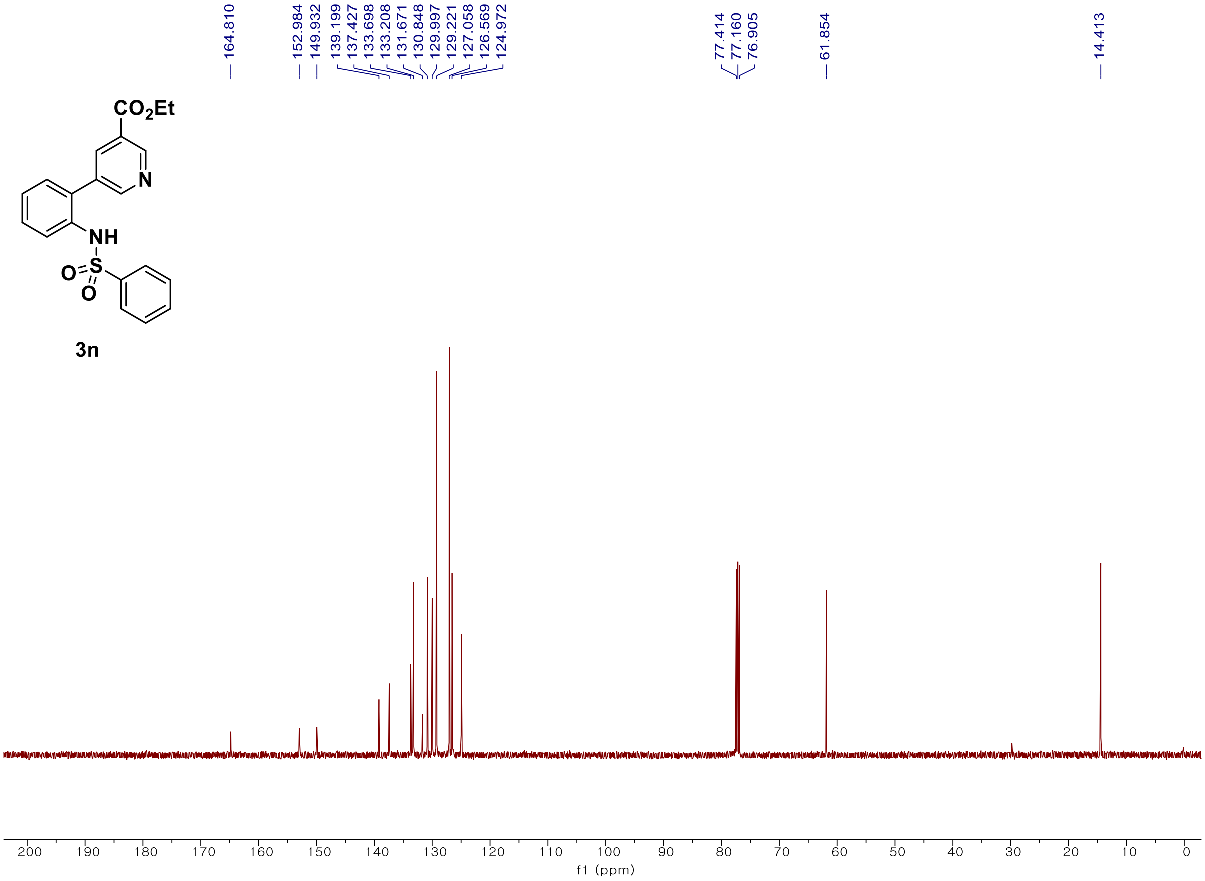
164.810
152.984
149.932
139.199
137.427
133.698
133.208
131.671
130.848
129.997
129.221
127.058
126.569
124.972
77.414
77.160
76.905
61.854
14.413
CO2Et
N
NH
O=S
O
3n
200 190 180 170 160 150 140 130 120 110 100 90 80 70 60 50 40 30 20 10 0
f1 (ppm)

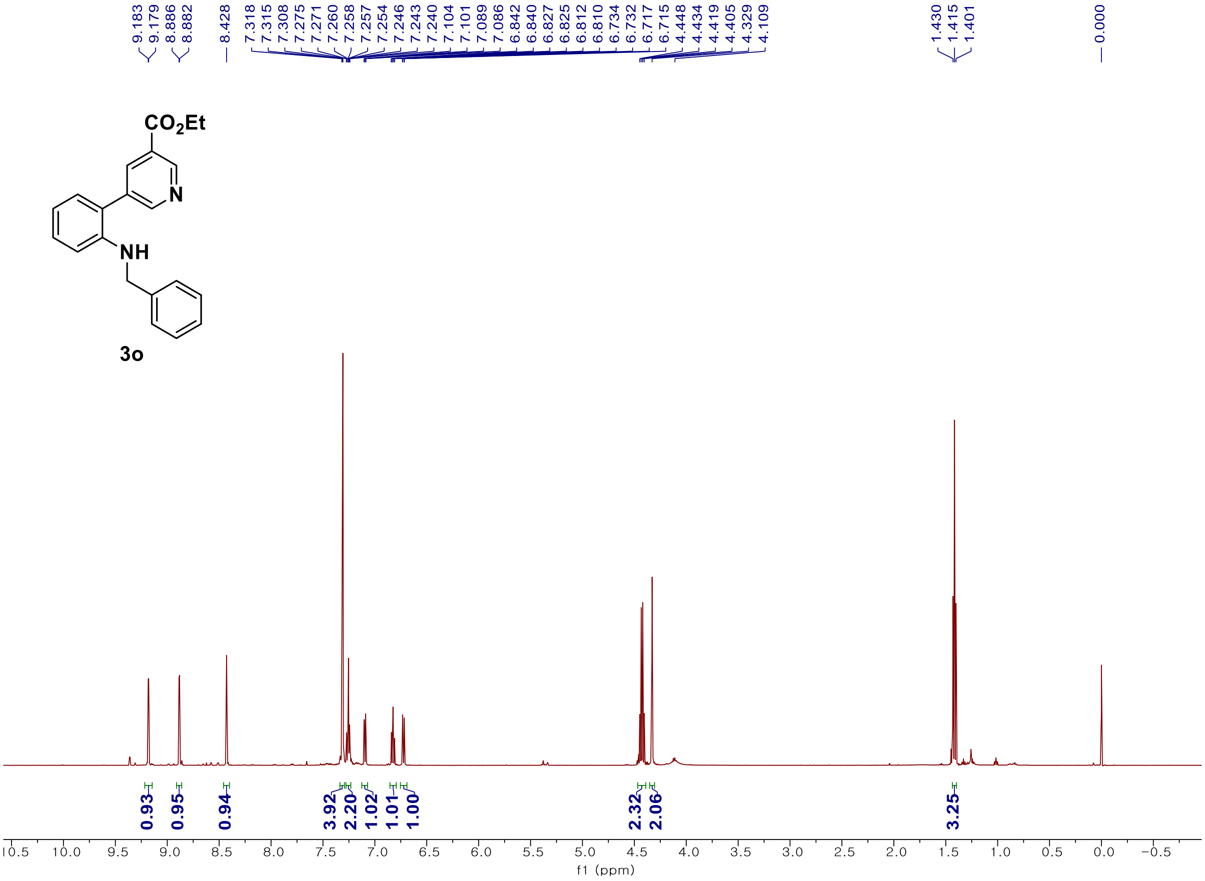
9.183
9.179
8.886
8.882
8.428
7.318
7.315
7.308
7.275
7.271
7.260
7.258
7.257
7.254
7.246
7.243
7.240
7.104
7.101
7.089
7.086
6.842
6.840
6.827
6.825
6.812
6.810
6.734
6.732
6.717
6.715
4.448
4.434
4.419
4.405
4.329
4.109
1.430
1.415
1.401
0.000
CO2Et
N
NH
3o
0.93
0.95
0.94
3.92
2.20
1.02
1.01
1.00
2.32
2.06
3.25
f1 (ppm)

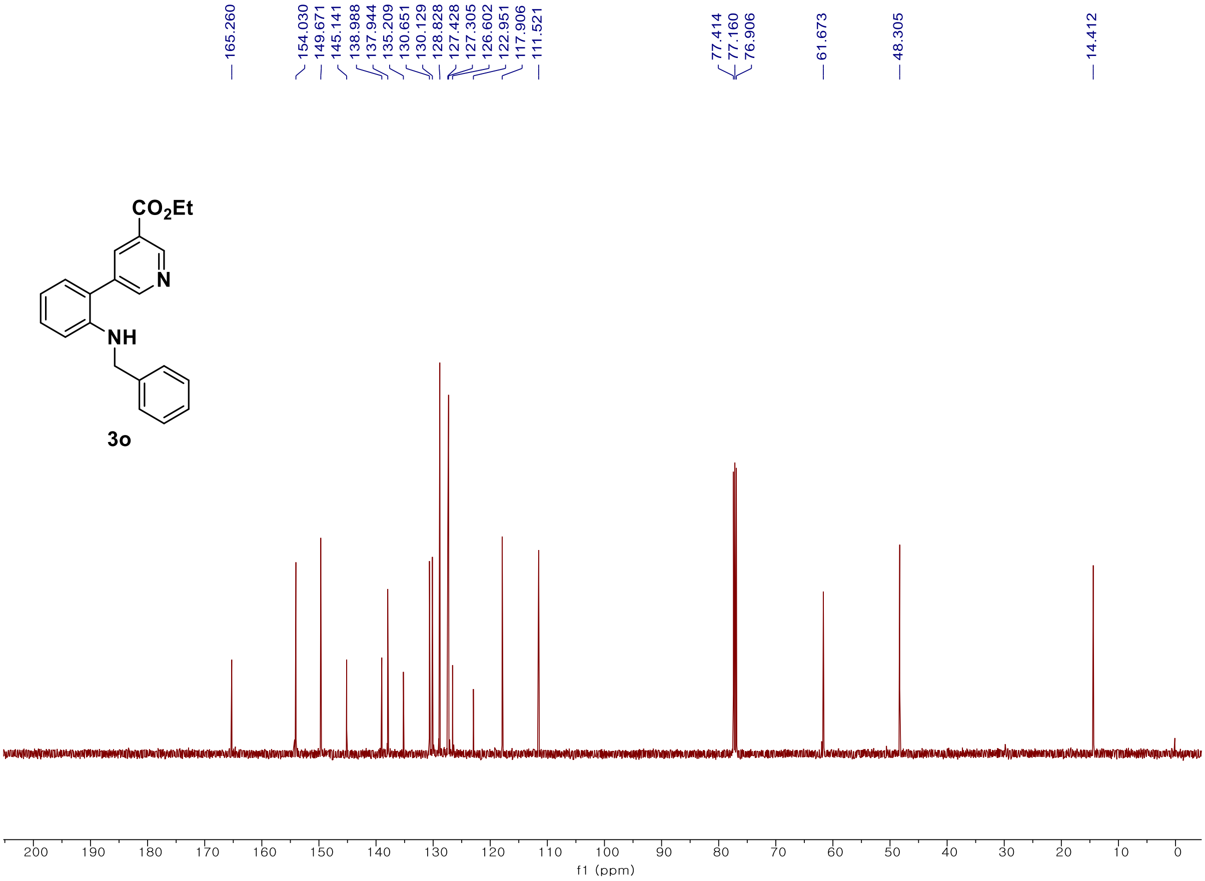
165.260
154.030
149.671
145.141
138.988
137.944
135.209
130.651
130.129
128.828
127.428
127.305
126.602
122.951
117.906
111.521
77.414
77.160
76.906
61.673
48.305
14.412
CO2Et
N
NH
3o
f1 (ppm)

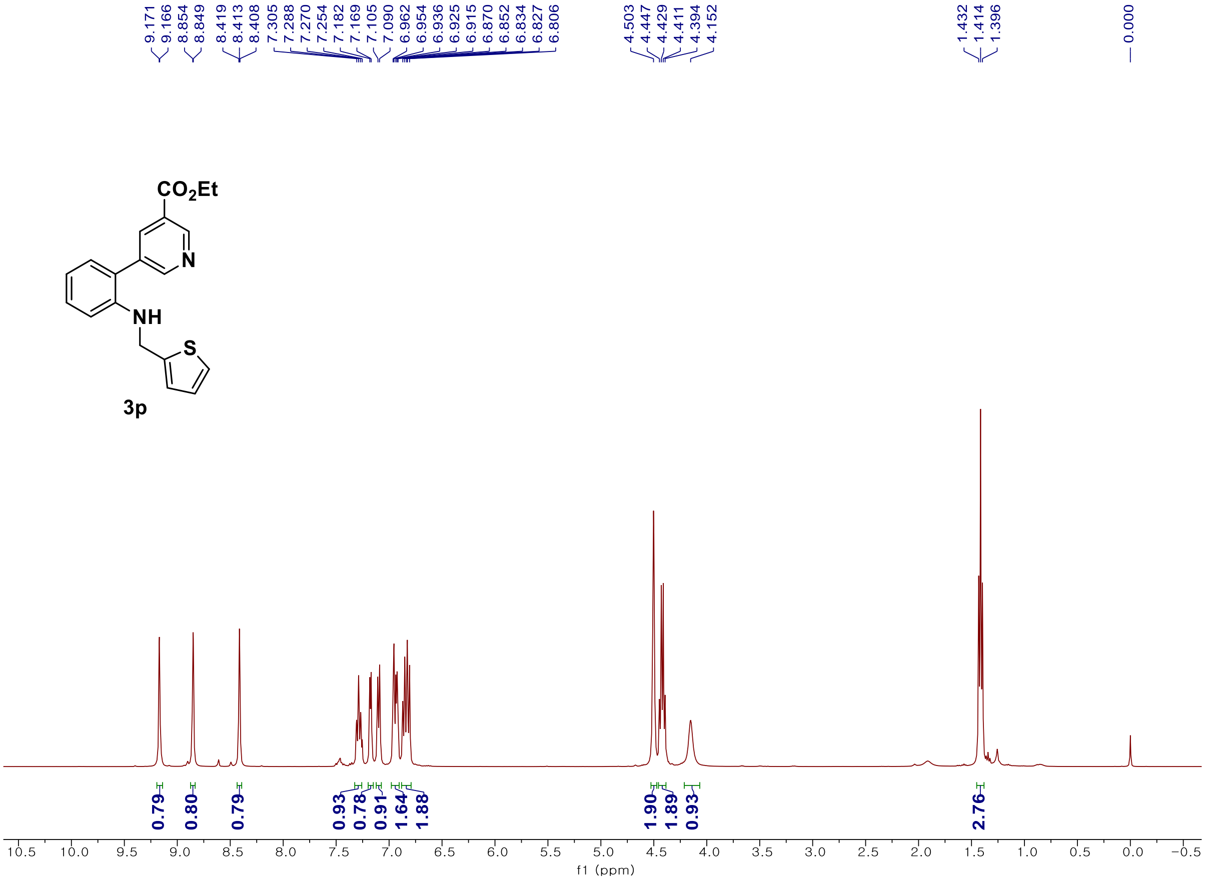
CO2Et
N
NH
S
3p
9.171
9.166
8.854
8.849
8.419
8.413
8.408
7.305
7.288
7.270
7.254
7.182
7.169
7.105
7.090
6.962
6.954
6.936
6.925
6.915
6.870
6.852
6.834
6.827
6.806
4.503
4.447
4.429
4.411
4.394
4.152
1.432
1.414
1.396
0.000
0.79
0.80
0.79
0.93
0.78
0.91
1.64
1.88
1.90
1.89
0.93
2.76
f1 (ppm)

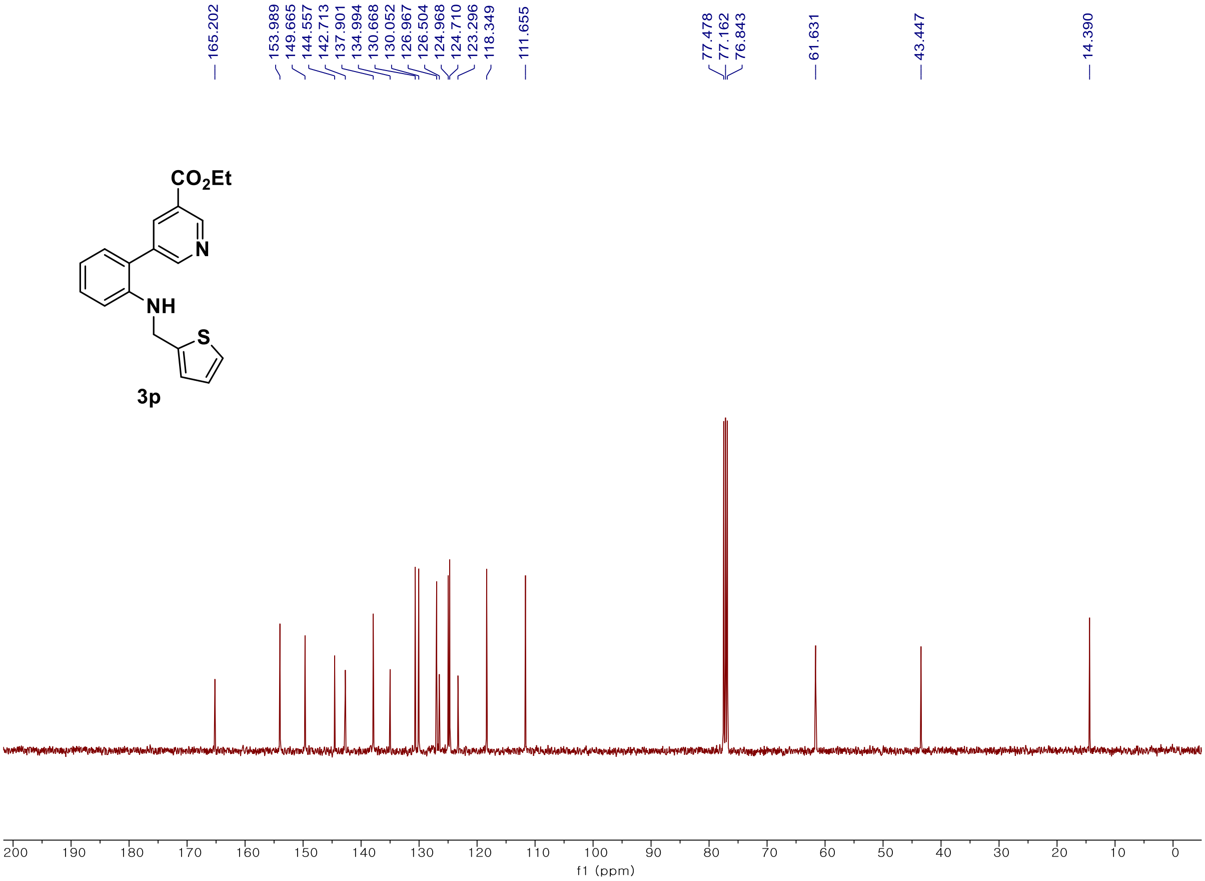
CO2Et
N
NH
S
3p
165.202
153.989
149.665
144.557
142.713
137.901
134.994
130.668
130.052
126.967
126.504
124.968
124.710
123.296
118.349
111.655
77.478
77.162
76.843
61.631
43.447
14.390
f1 (ppm)

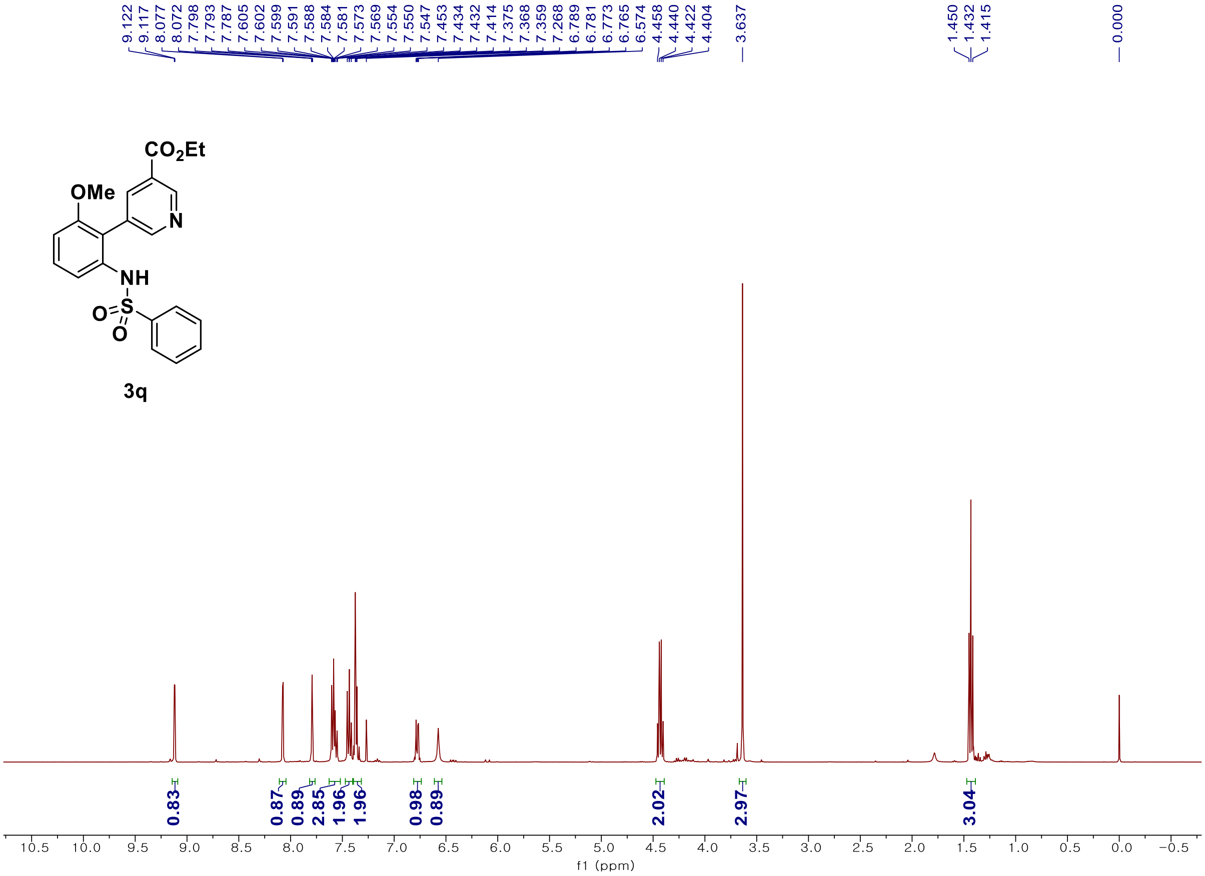

3q

9.122
9.117
8.077
8.072
7.798
7.793
7.787
7.605
7.602
7.599
7.591
7.588
7.584
7.581
7.573
7.569
7.554
7.550
7.547
7.453
7.434
7.432
7.414
7.375
7.368
7.359
7.268
6.789
6.781
6.773
6.765
6.574
4.458
4.440
4.422
4.404
3.637
1.450
1.432
1.415
0.000
0.83
0.87
0.89
2.85
1.96
1.96
0.98
0.89
2.02
2.97
3.04
f1 (ppm)

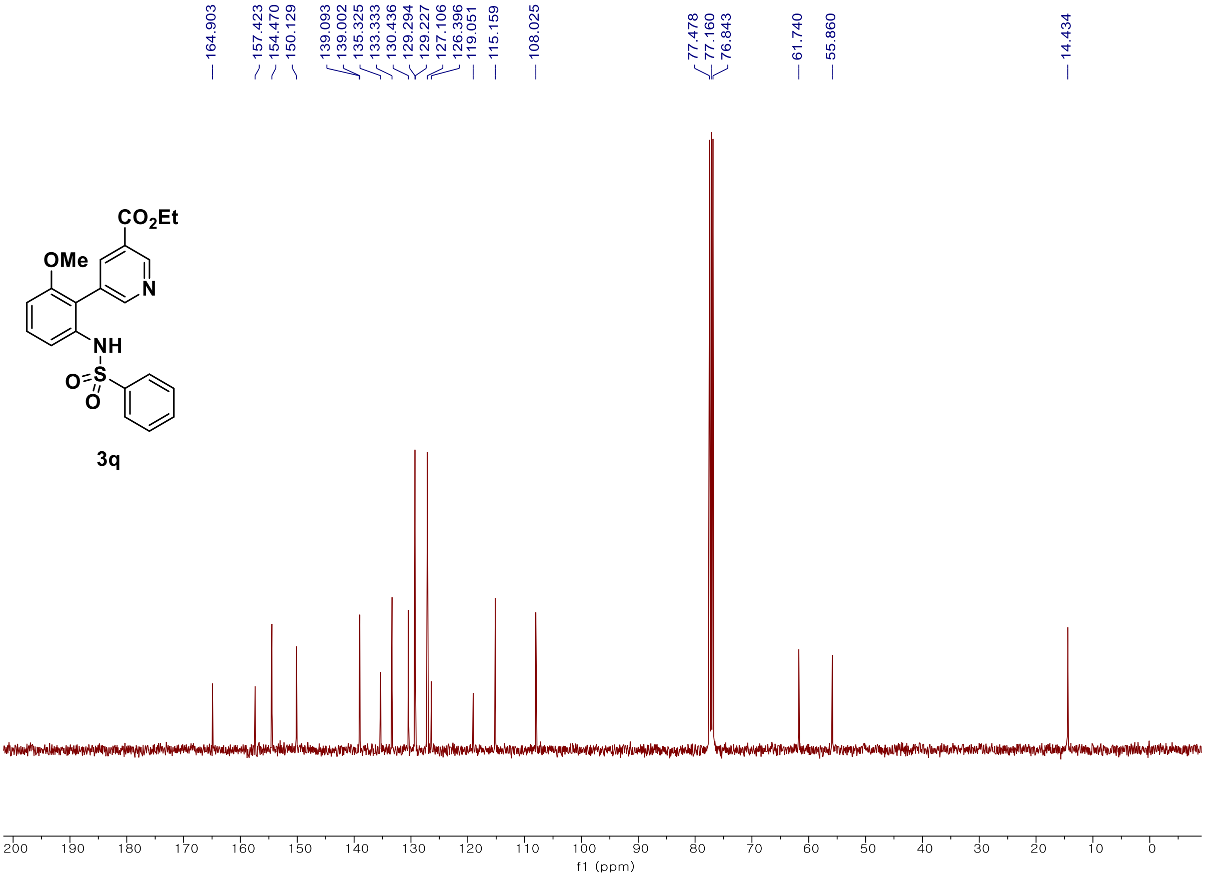

3q

164.903
157.423
154.470
150.129
139.093
139.002
135.325
133.333
130.436
129.294
129.227
127.106
126.396
119.051
115.159
108.025
77.478
77.160
76.843
61.740
55.860
14.434
f1 (ppm)

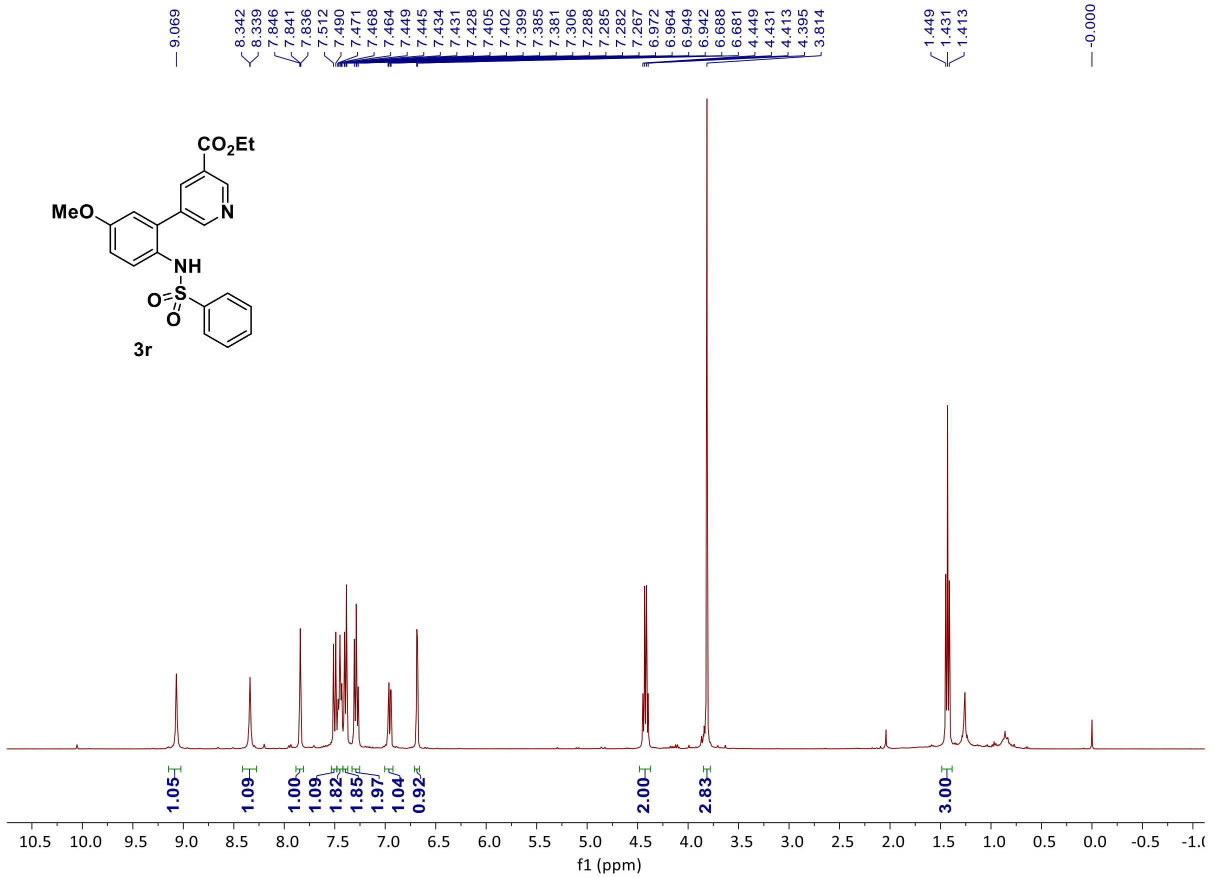
9.069
8.342
8.339
7.846
7.841
7.836
7.512
7.490
7.471
7.468
7.464
7.449
7.445
7.434
7.431
7.428
7.405
7.402
7.399
7.385
7.381
7.306
7.288
7.285
7.282
7.267
6.972
6.964
6.949
6.942
6.688
6.681
4.449
4.431
4.413
4.395
3.814
1.449
1.431
1.413
-0.000
CO2Et
MeO
N
NH
O=S
O
3r
1.05
1.09
1.00
1.09
1.82
1.85
1.97
1.04
0.92
2.00
2.83
3.00
f1 (ppm)

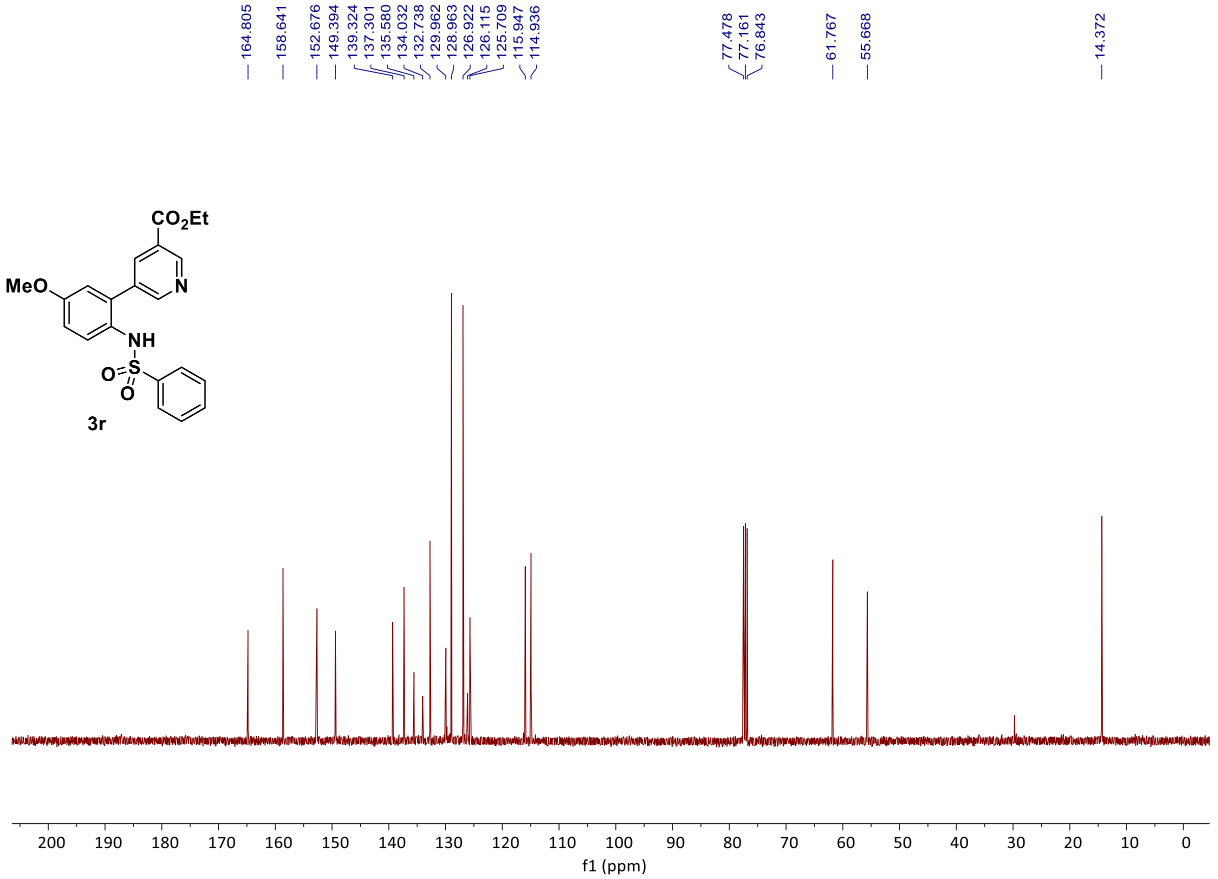
164.805
158.641
152.676
149.394
139.324
137.301
135.580
134.032
132.738
129.962
128.963
126.922
126.115
125.709
115.947
114.936
77.478
77.161
76.843
61.767
55.668
14.372
CO2Et
MeO
N
NH
O=S
O
3r
f1 (ppm)

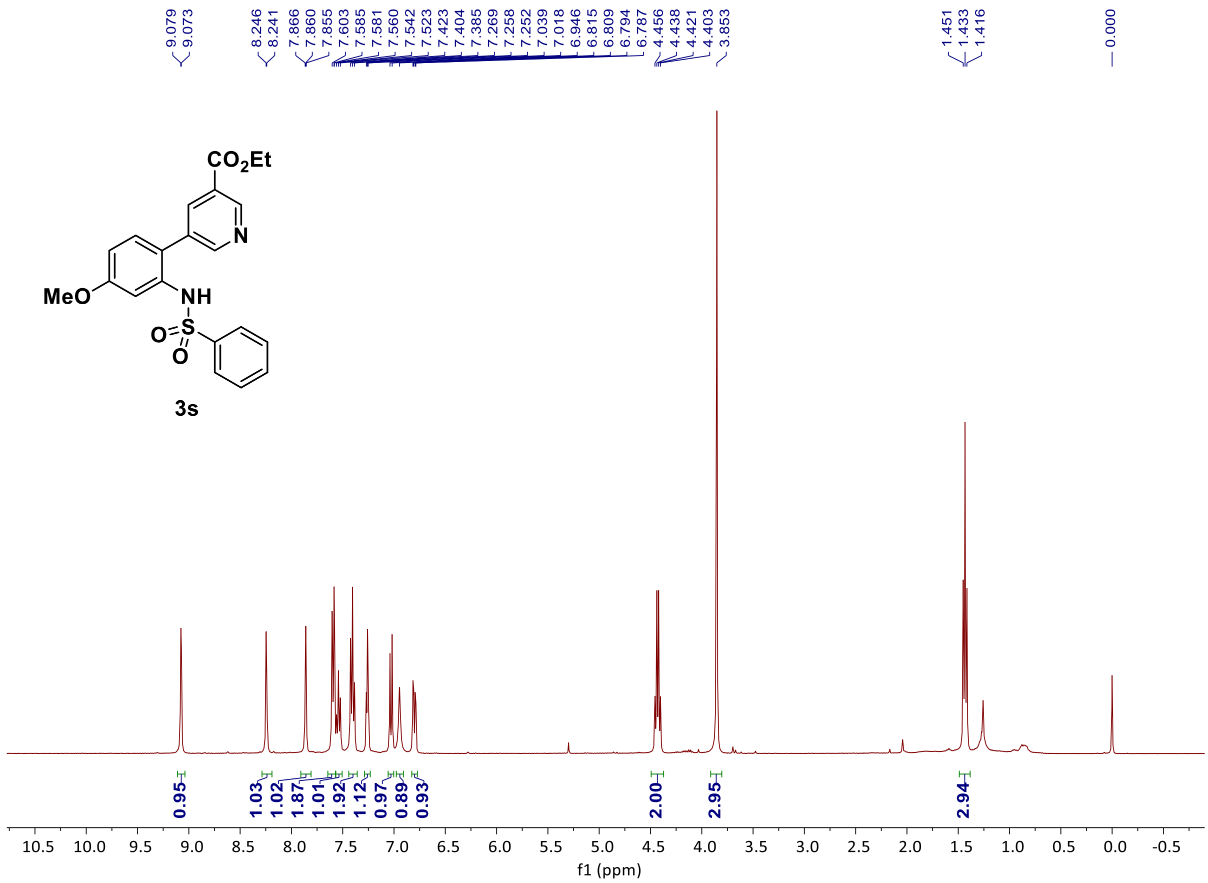
CO2Et
N
MeO
NH
O=S
O
3s
9.079
9.073
8.246
8.241
7.866
7.860
7.855
7.603
7.585
7.581
7.560
7.542
7.523
7.423
7.404
7.385
7.269
7.258
7.252
7.039
7.018
6.946
6.815
6.809
6.794
6.787
4.456
4.438
4.421
4.403
3.853
1.451
1.433
1.416
0.000
0.95
1.03
1.02
1.87
1.01
1.92
1.12
0.97
0.89
0.93
2.00
2.95
2.94
f1 (ppm)

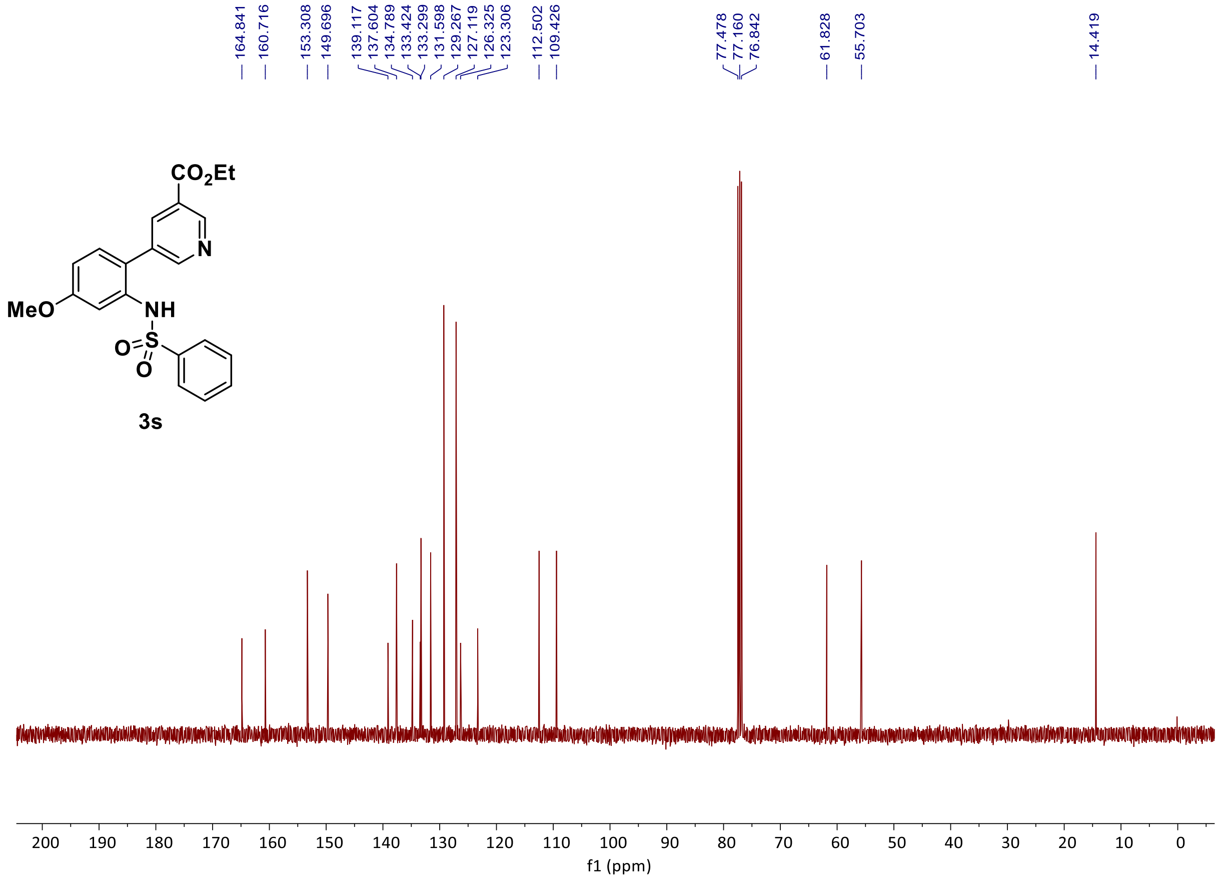
CO2Et
N
MeO
NH
O=S
O
3s
164.841
160.716
153.308
149.696
139.117
137.604
134.789
133.424
133.299
131.598
129.267
127.119
126.325
123.306
112.502
109.426
77.478
77.160
76.842
61.828
55.703
14.419
f1 (ppm)

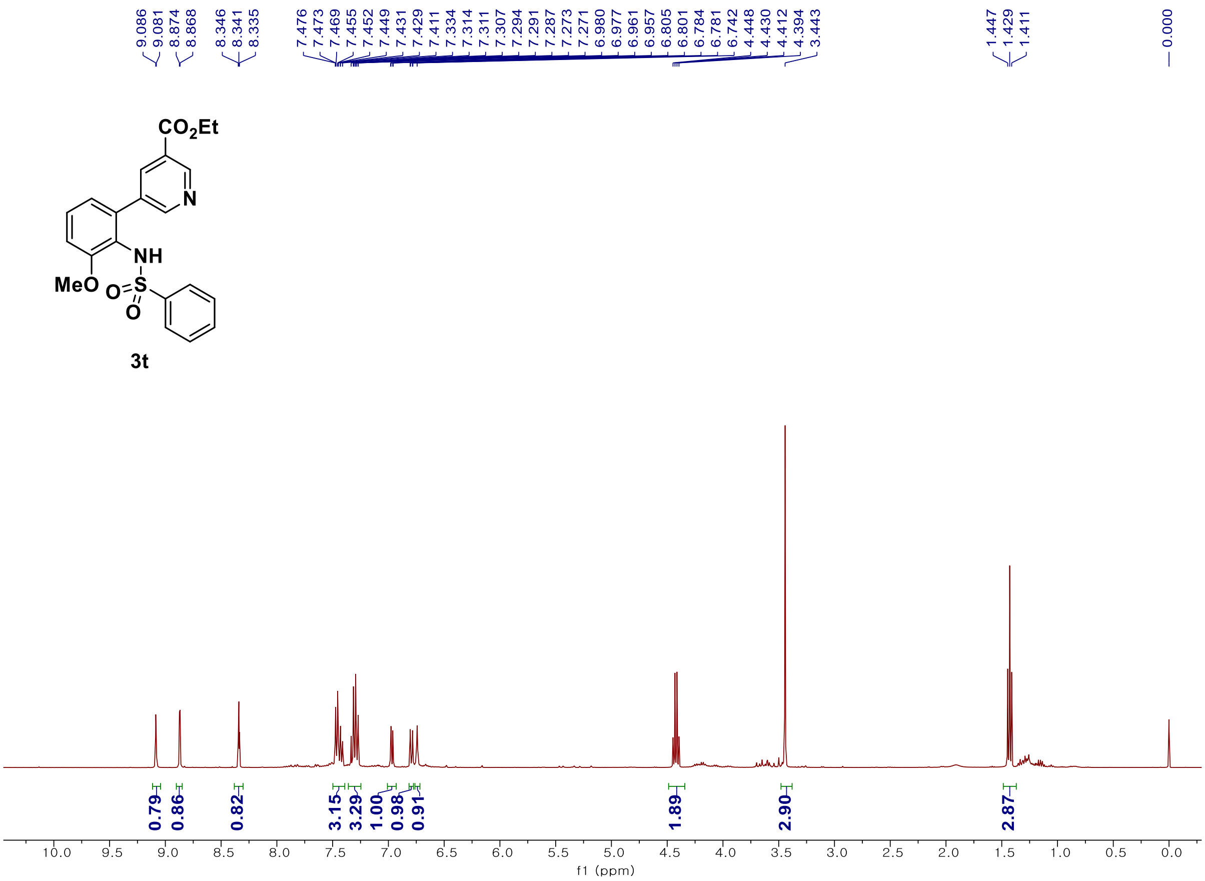

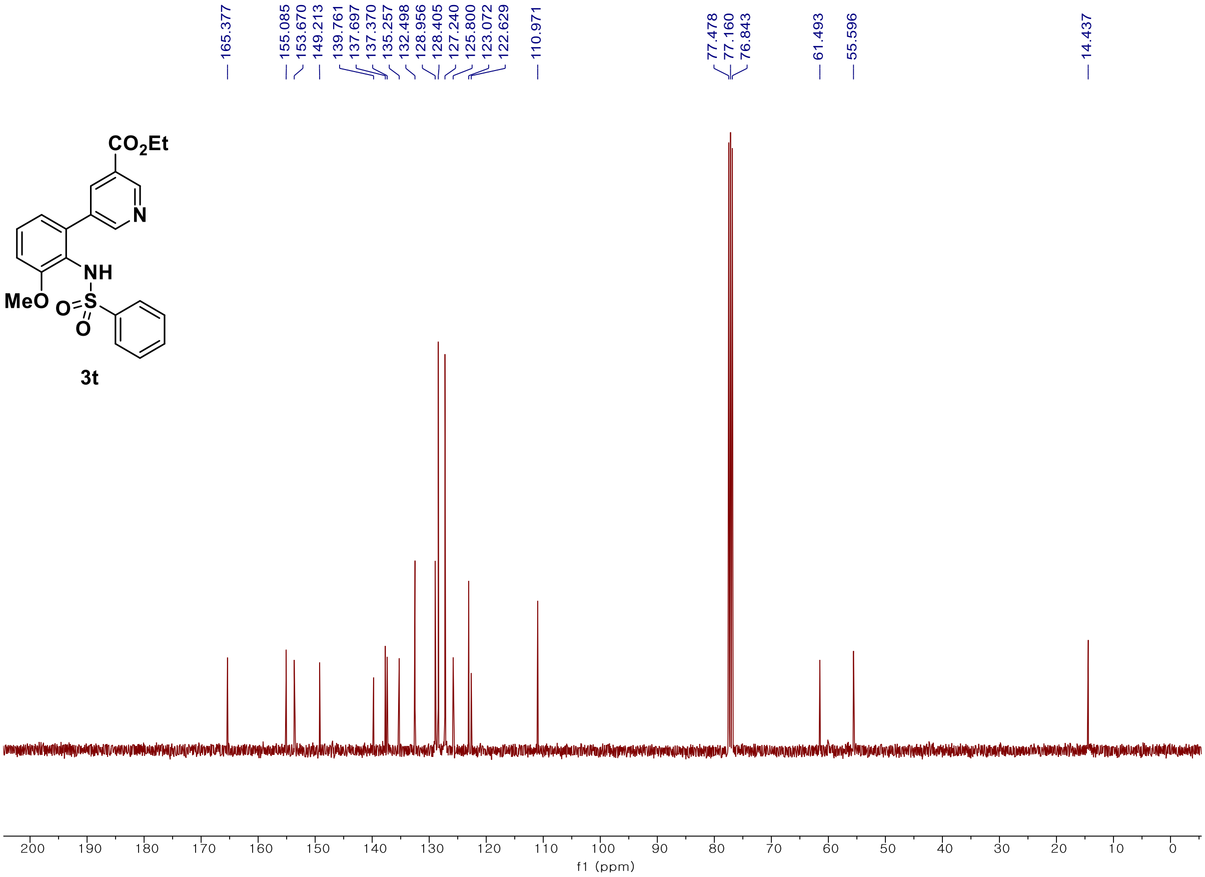

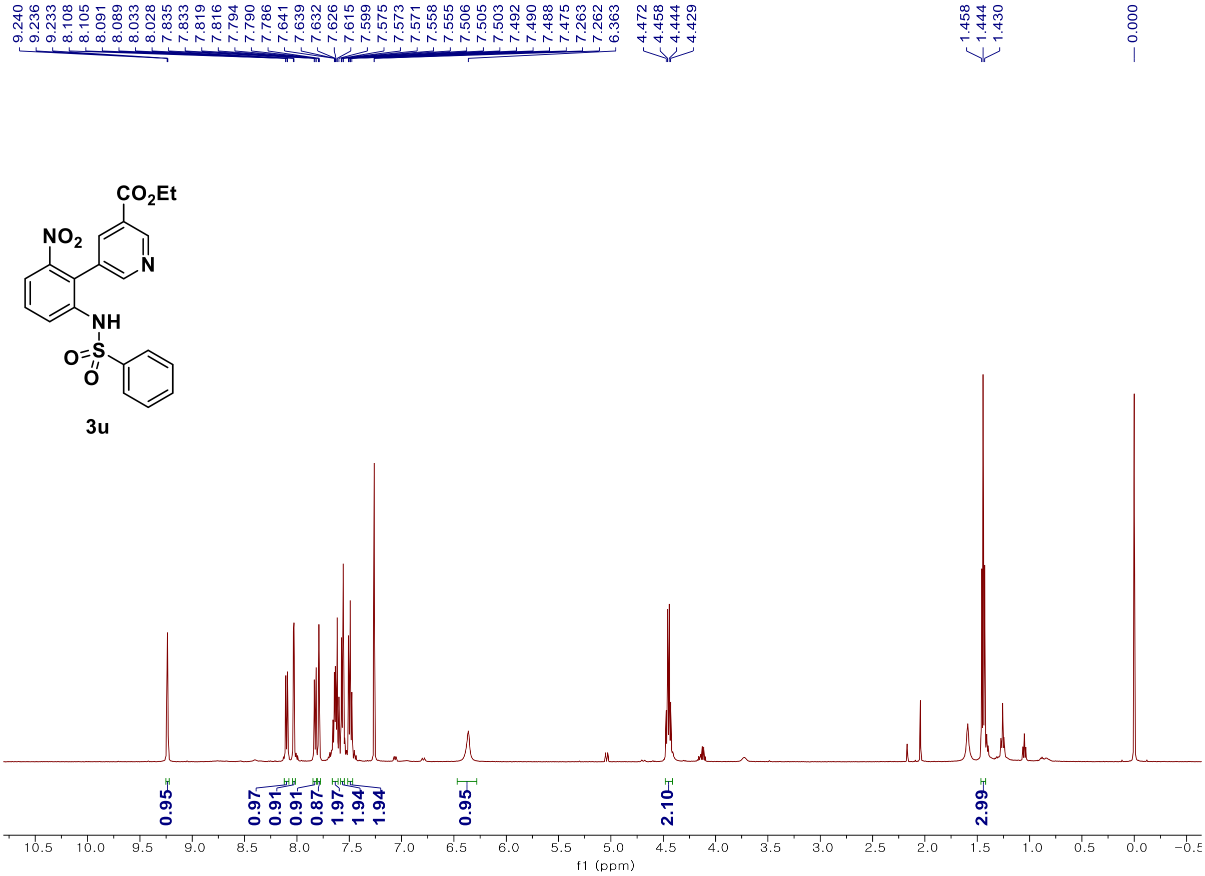
9.240
9.236
9.233
8.108
8.105
8.091
8.089
8.033
8.028
7.835
7.833
7.819
7.816
7.794
7.790
7.786
7.641
7.639
7.632
7.626
7.615
7.599
7.575
7.573
7.571
7.558
7.555
7.506
7.505
7.503
7.492
7.490
7.488
7.475
7.263
7.262
6.363
4.472
4.458
4.444
4.429
1.458
1.444
1.430
0.000
CO2Et
NO2
N
NH
O=S
O
3u
0.95
0.97
0.91
0.91
0.87
1.97
1.94
1.94
0.95
2.10
2.99
f1 (ppm)

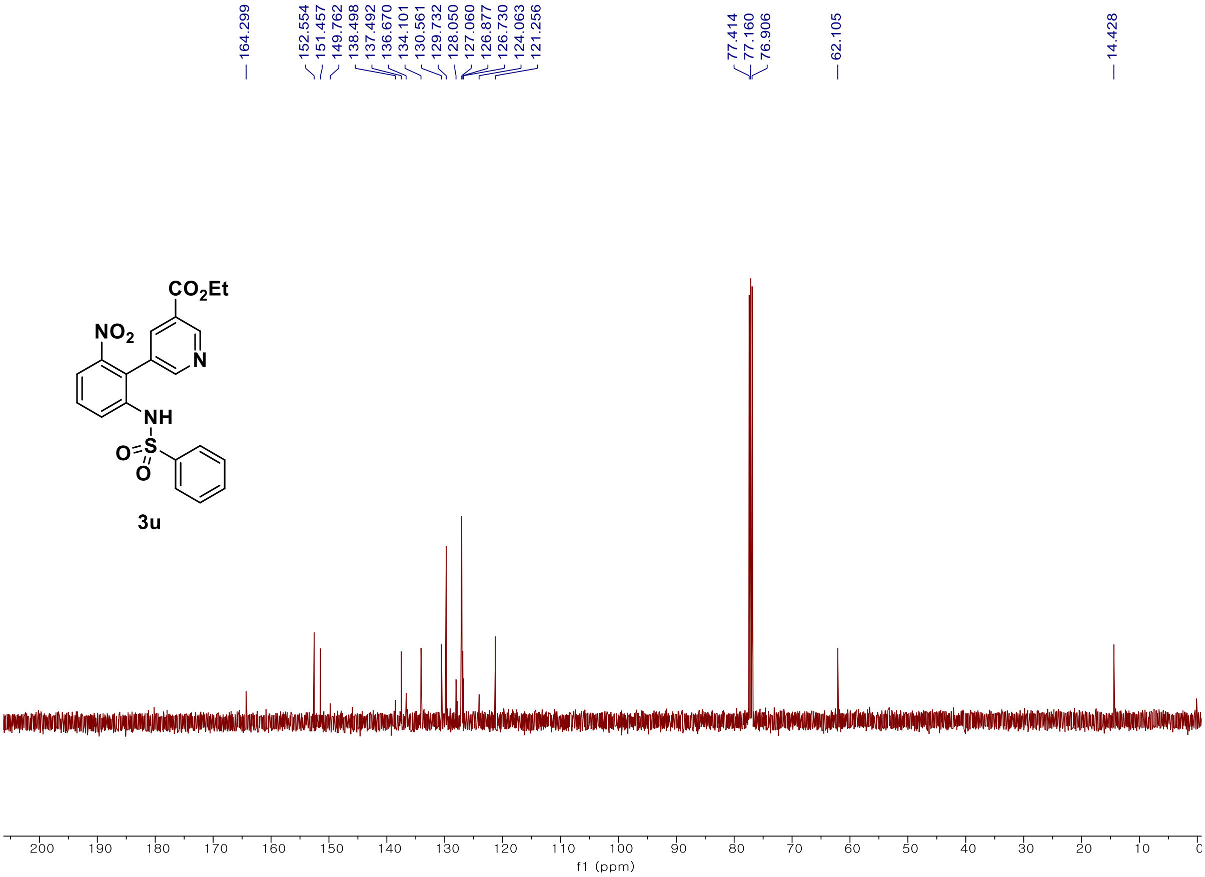
164.299
152.554
151.457
149.762
138.498
137.492
136.670
134.101
130.561
129.732
128.050
127.060
126.877
126.730
124.063
121.256
77.414
77.160
76.906
62.105
14.428
CO2Et
NO2
N
NH
O=S
O
3u
f1 (ppm)

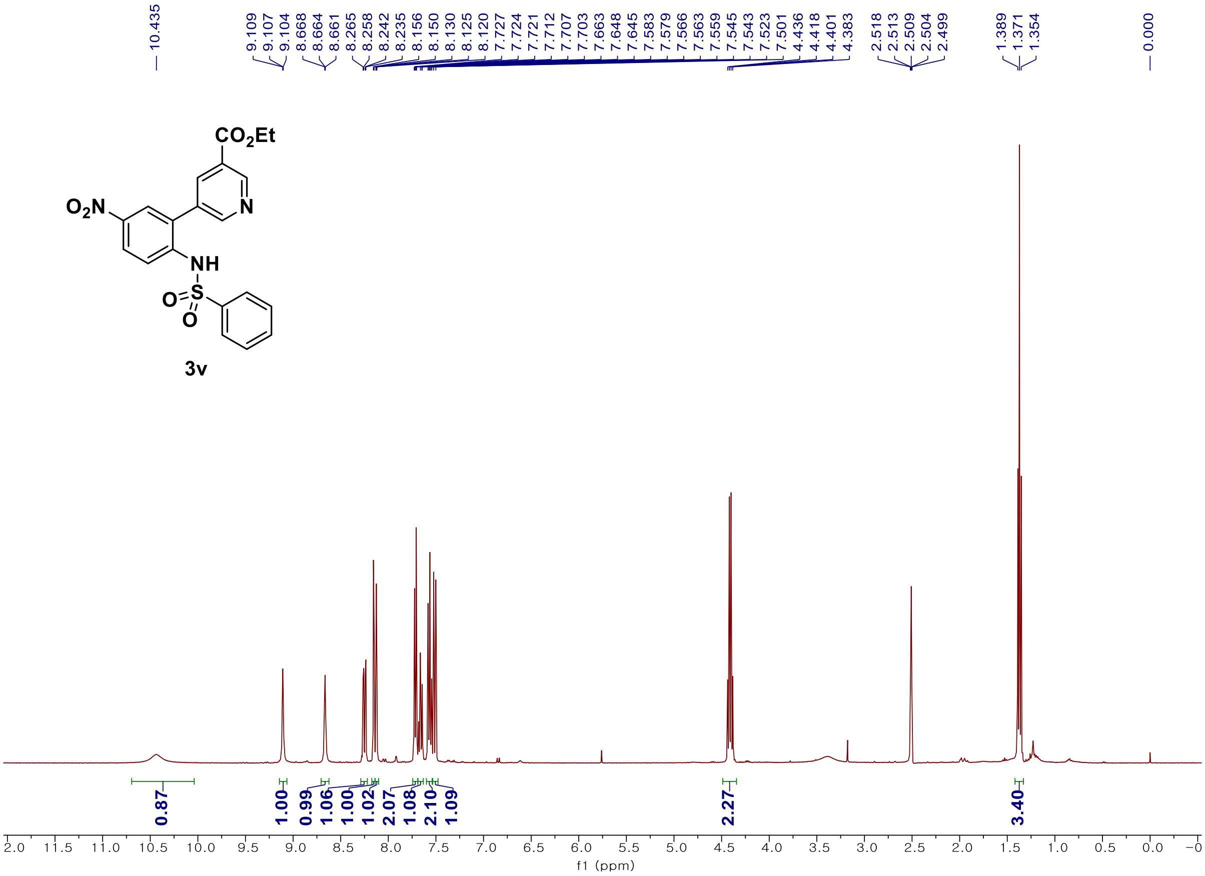

3v

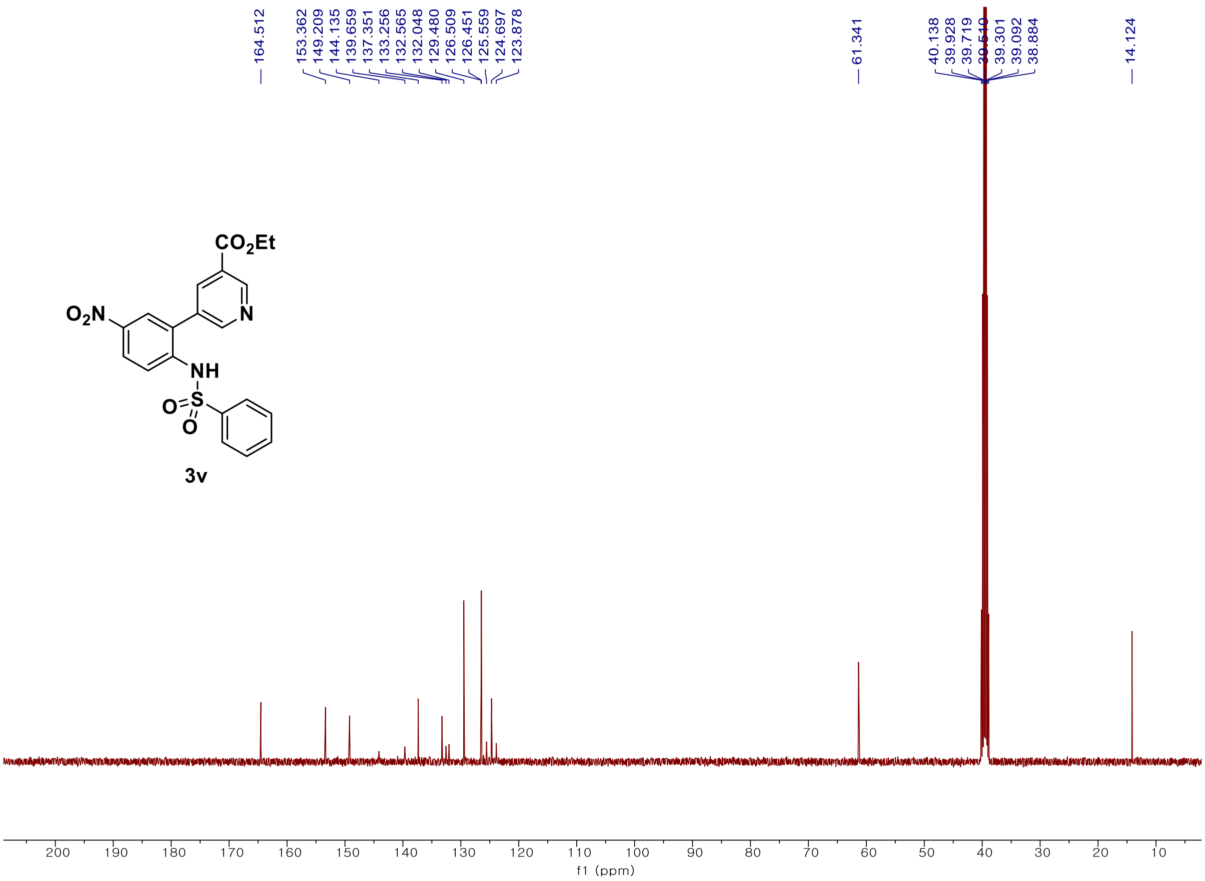

3v

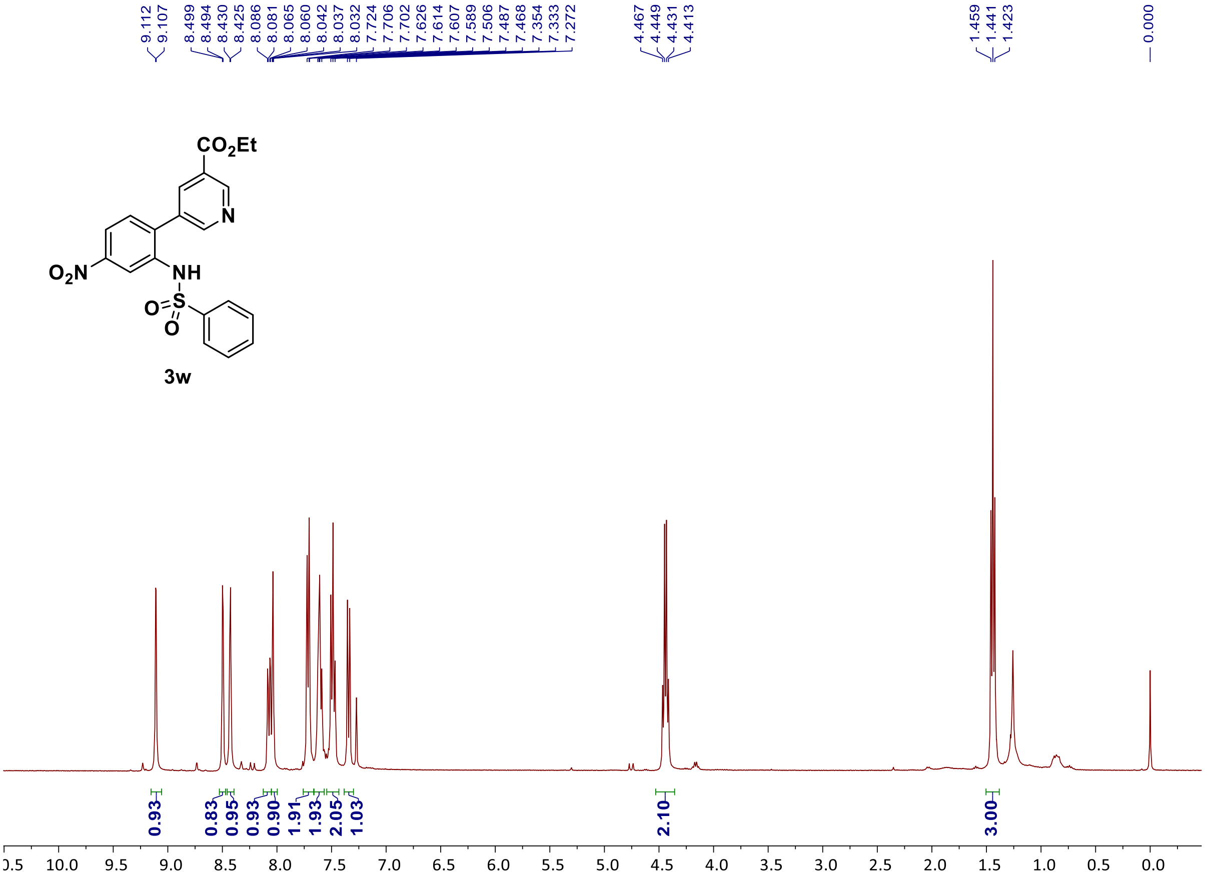
9.112
9.107
8.499
8.494
8.430
8.425
8.086
8.081
8.065
8.060
8.042
8.037
8.032
7.724
7.706
7.702
7.626
7.614
7.607
7.589
7.506
7.487
7.468
7.354
7.333
7.272
4.467
4.449
4.431
4.413
1.459
1.441
1.423
0.000
CO2Et
N
O2N
NH
S
O
O
3w
0.93
0.83
0.95
0.93
0.90
1.91
1.93
2.05
1.03
2.10
3.00

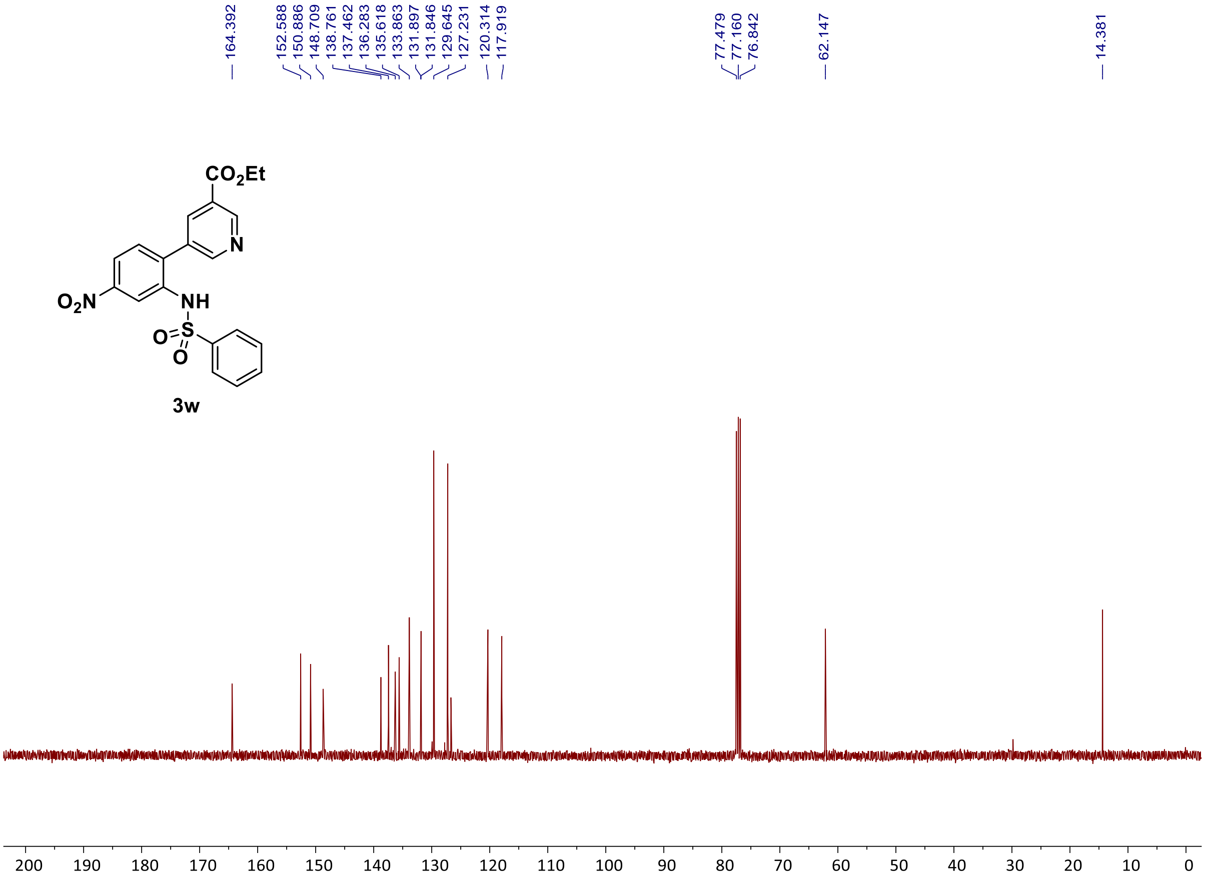
164.392
152.588
150.886
148.709
138.761
137.462
136.283
135.618
133.863
131.897
131.846
129.645
127.231
120.314
117.919
77.479
77.160
76.842
62.147
14.381
CO2Et
N
O2N
NH
S
O
O
3w

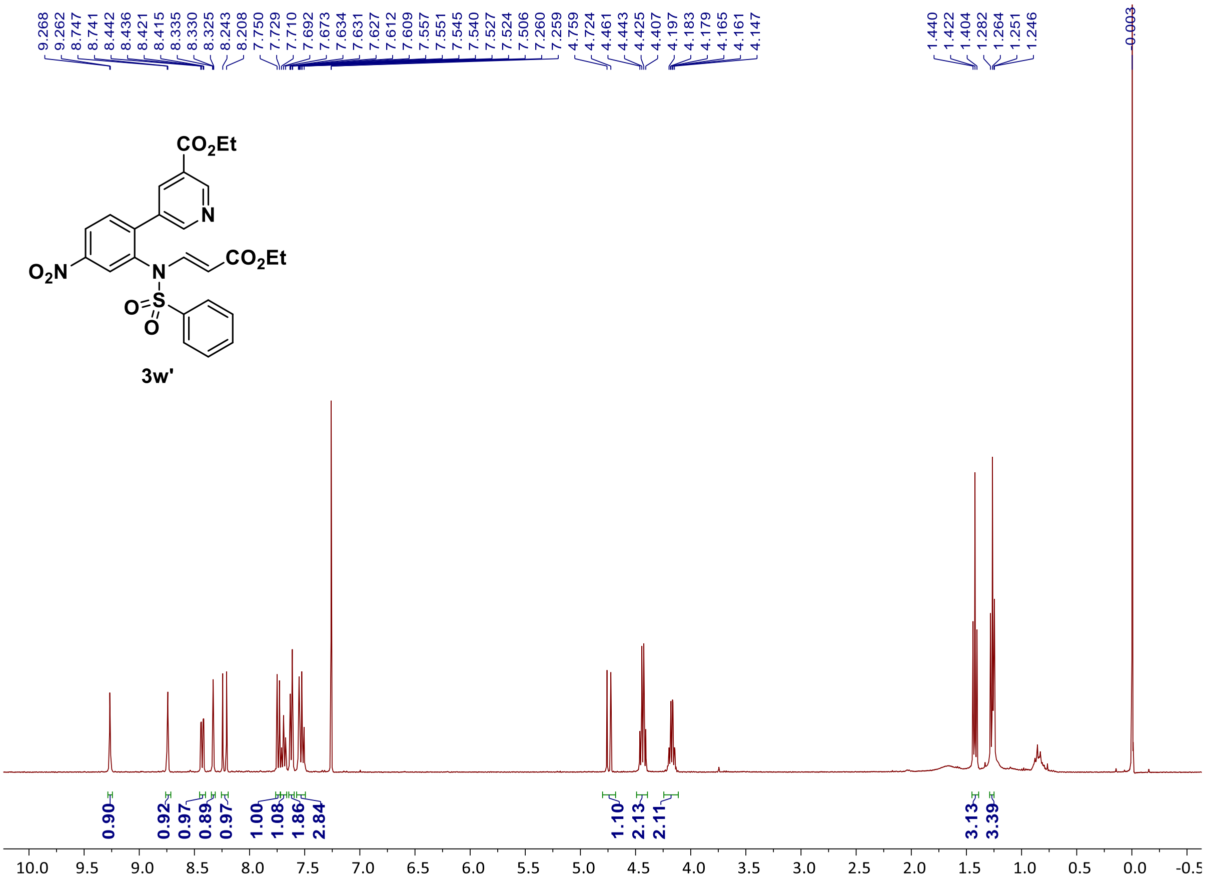
9.268
9.262
8.747
8.741
8.442
8.436
8.421
8.415
8.335
8.330
8.325
8.243
8.208
7.750
7.729
7.710
7.692
7.673
7.634
7.631
7.627
7.612
7.609
7.557
7.551
7.545
7.540
7.527
7.524
7.506
7.260
7.259
4.759
4.724
4.461
4.443
4.425
4.407
4.197
4.183
4.179
4.165
4.161
4.147
1.440
1.422
1.404
1.282
1.264
1.251
1.246
0.003
CO2Et
N
O2N
N
CO2Et
O=S=O
3w'
0.90
0.92
0.97
0.89
0.97
1.00
1.08
1.86
2.84
1.10
2.13
2.11
3.13
3.39
10.0 9.5 9.0 8.5 8.0 7.5 7.0 6.5 6.0 5.5 5.0 4.5 4.0 3.5 3.0 2.5 2.0 1.5 1.0 0.5 0.0 -0.5

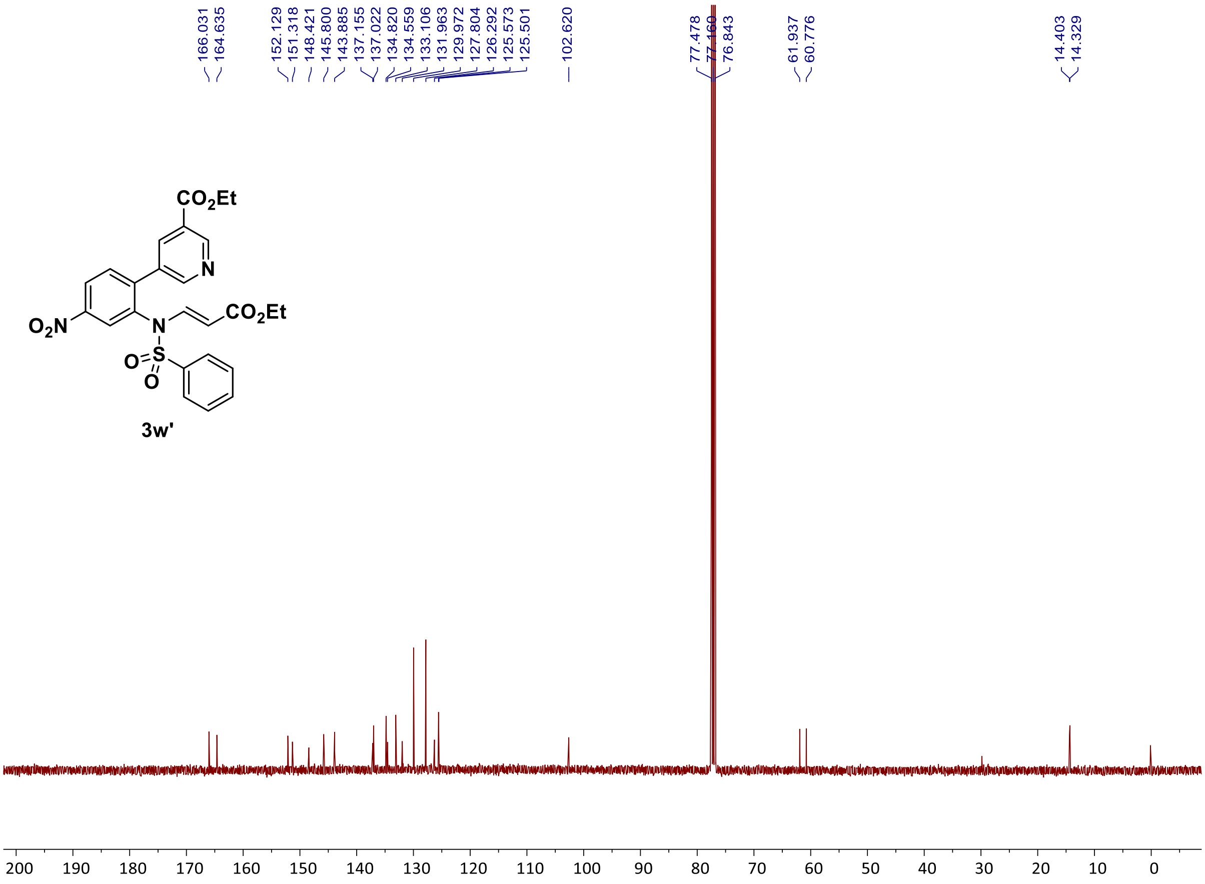
166.031
164.635
152.129
151.318
148.421
145.800
143.885
137.155
137.022
134.820
134.559
133.106
131.963
129.972
127.804
126.292
125.573
125.501
102.620
77.478
77.160
76.843
61.937
60.776
14.403
14.329
CO2Et
N
O2N
N
CO2Et
O=S=O
3w'
200 190 180 170 160 150 140 130 120 110 100 90 80 70 60 50 40 30 20 10 0

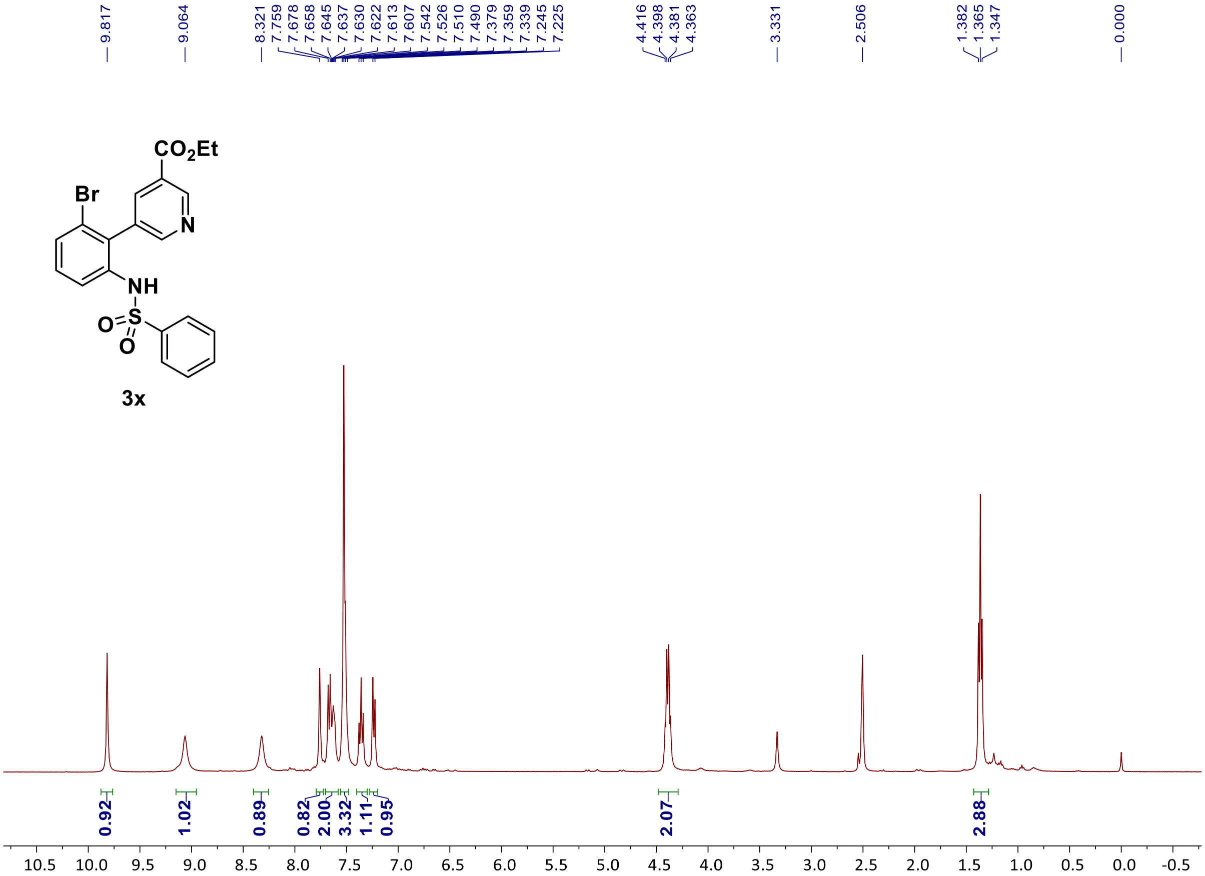
9.817
9.064
8.321
7.759
7.678
7.658
7.645
7.637
7.630
7.622
7.613
7.607
7.542
7.526
7.510
7.490
7.379
7.359
7.339
7.245
7.225
4.416
4.398
4.381
4.363
3.331
2.506
1.382
1.365
1.347
0.000
CO2Et
Br
N
NH
O=S
O
3x
0.92
1.02
0.89
0.82
2.00
3.32
1.11
0.95
2.07
2.88
10.5 10.0 9.5 9.0 8.5 8.0 7.5 7.0 6.5 6.0 5.5 5.0 4.5 4.0 3.5 3.0 2.5 2.0 1.5 1.0 0.5 0.0 -0.5

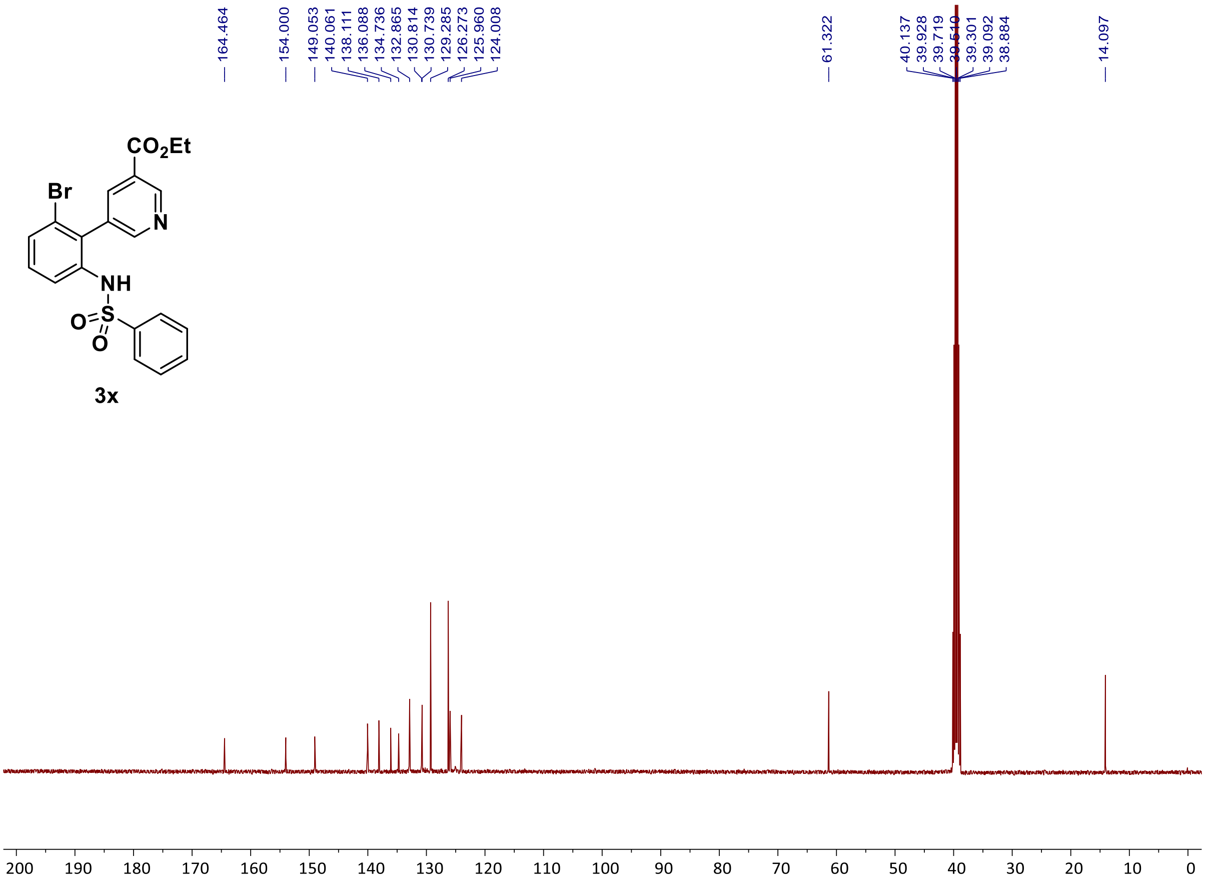
164.464
154.000
149.053
140.061
138.111
136.088
134.736
132.865
130.814
130.739
129.285
126.273
125.960
124.008
61.322
40.137
39.928
39.719
39.510
39.301
39.092
38.884
14.097
CO2Et
Br
N
NH
O=S
O
3x
200 190 180 170 160 150 140 130 120 110 100 90 80 70 60 50 40 30 20 10 0

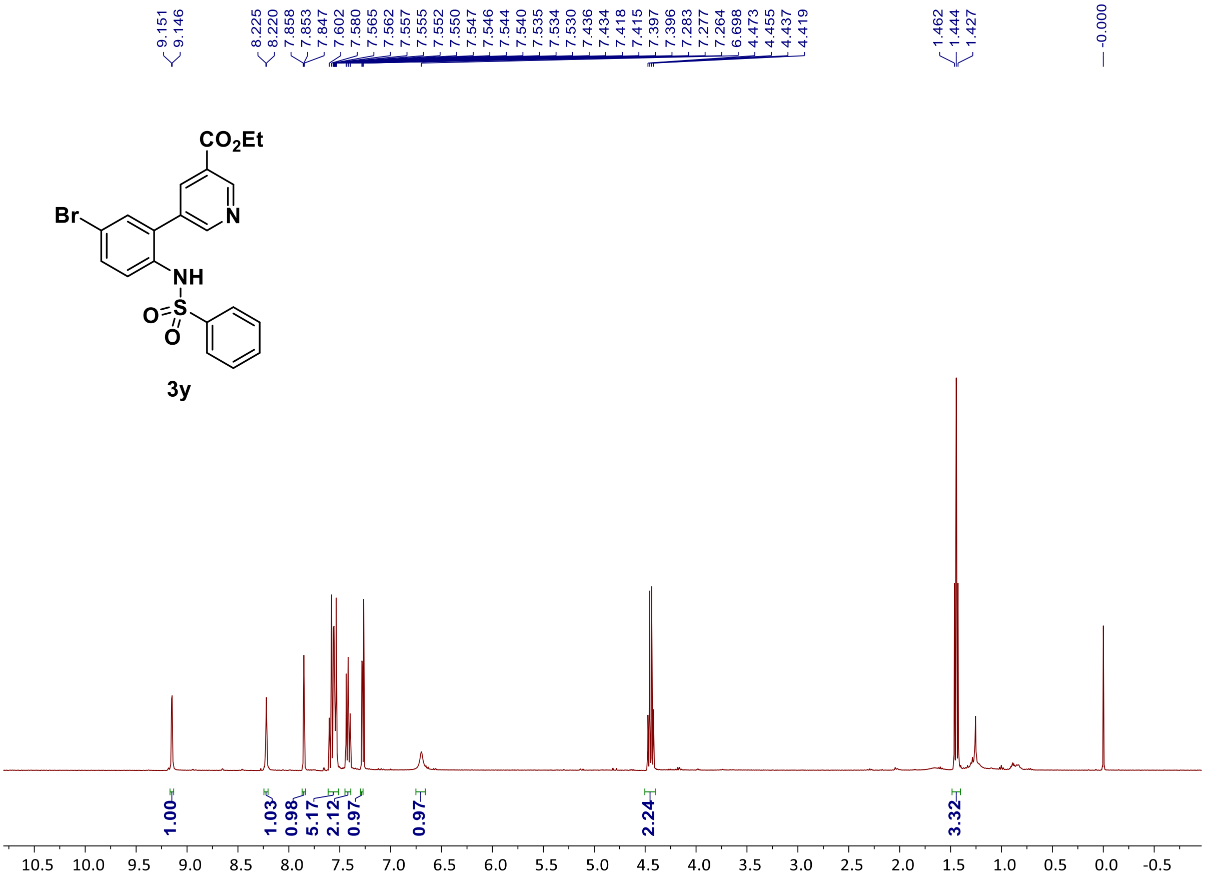
9.151
9.146
8.225
8.220
7.858
7.853
7.847
7.602
7.580
7.565
7.562
7.557
7.555
7.552
7.550
7.547
7.546
7.544
7.540
7.535
7.534
7.530
7.436
7.434
7.418
7.415
7.397
7.396
7.283
7.277
7.264
6.698
4.473
4.455
4.437
4.419
1.462
1.444
1.427
-0.000
CO2Et
Br
N
NH
O
S
O
3y
1.00
1.03
0.98
5.17
2.12
0.97
0.97
2.24
3.32
10.5 10.0 9.5 9.0 8.5 8.0 7.5 7.0 6.5 6.0 5.5 5.0 4.5 4.0 3.5 3.0 2.5 2.0 1.5 1.0 0.5 0.0 -0.5

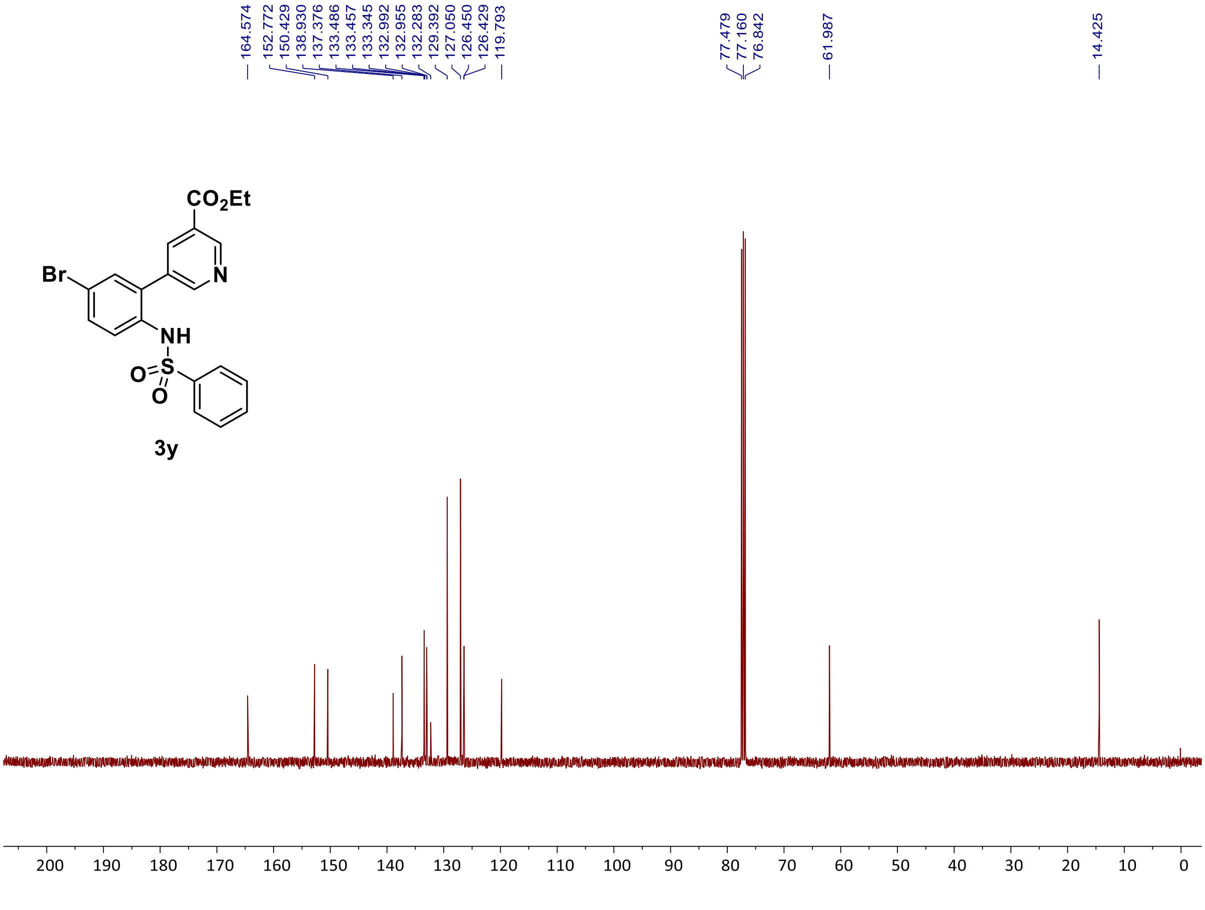
164.574
152.772
150.429
138.930
137.376
133.486
133.457
133.345
132.992
132.955
132.283
129.392
127.050
126.450
126.429
119.793
77.479
77.160
76.842
61.987
14.425
CO2Et
Br
N
NH
O
S
O
3y
200 190 180 170 160 150 140 130 120 110 100 90 80 70 60 50 40 30 20 10 0

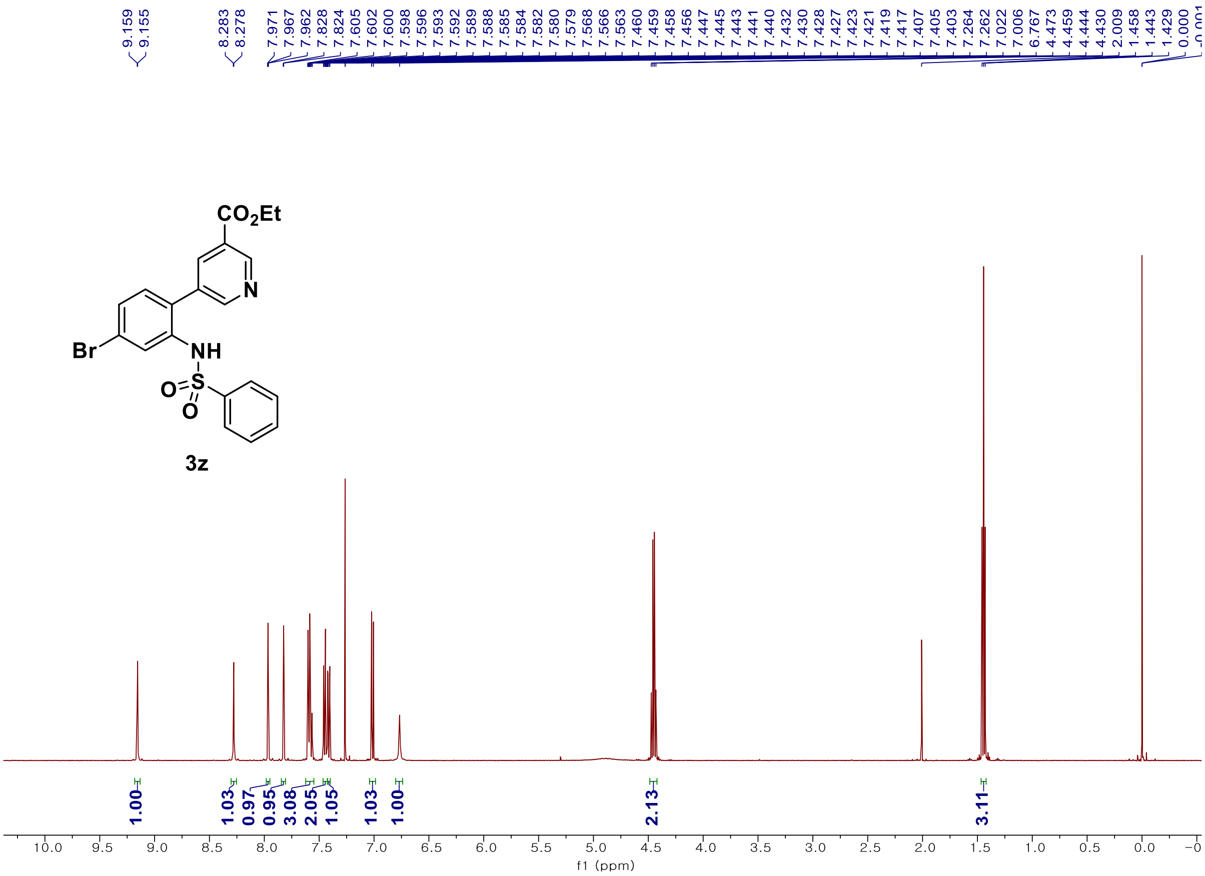
9.159
9.155
8.283
8.278
7.971
7.967
7.962
7.828
7.824
7.605
7.602
7.600
7.598
7.596
7.593
7.592
7.589
7.588
7.585
7.584
7.582
7.580
7.579
7.568
7.566
7.563
7.460
7.459
7.458
7.456
7.447
7.445
7.443
7.441
7.440
7.432
7.430
7.428
7.427
7.423
7.421
7.419
7.417
7.407
7.405
7.403
7.264
7.262
7.022
7.006
6.767
4.473
4.459
4.444
4.430
2.009
1.458
1.443
1.429
0.000
CO_2Et
N
Br
NH
O=S
O
3z
1.00
1.03
0.97
0.95
3.08
2.05
1.05
1.03
1.00
2.13
3.11
f1 (ppm)

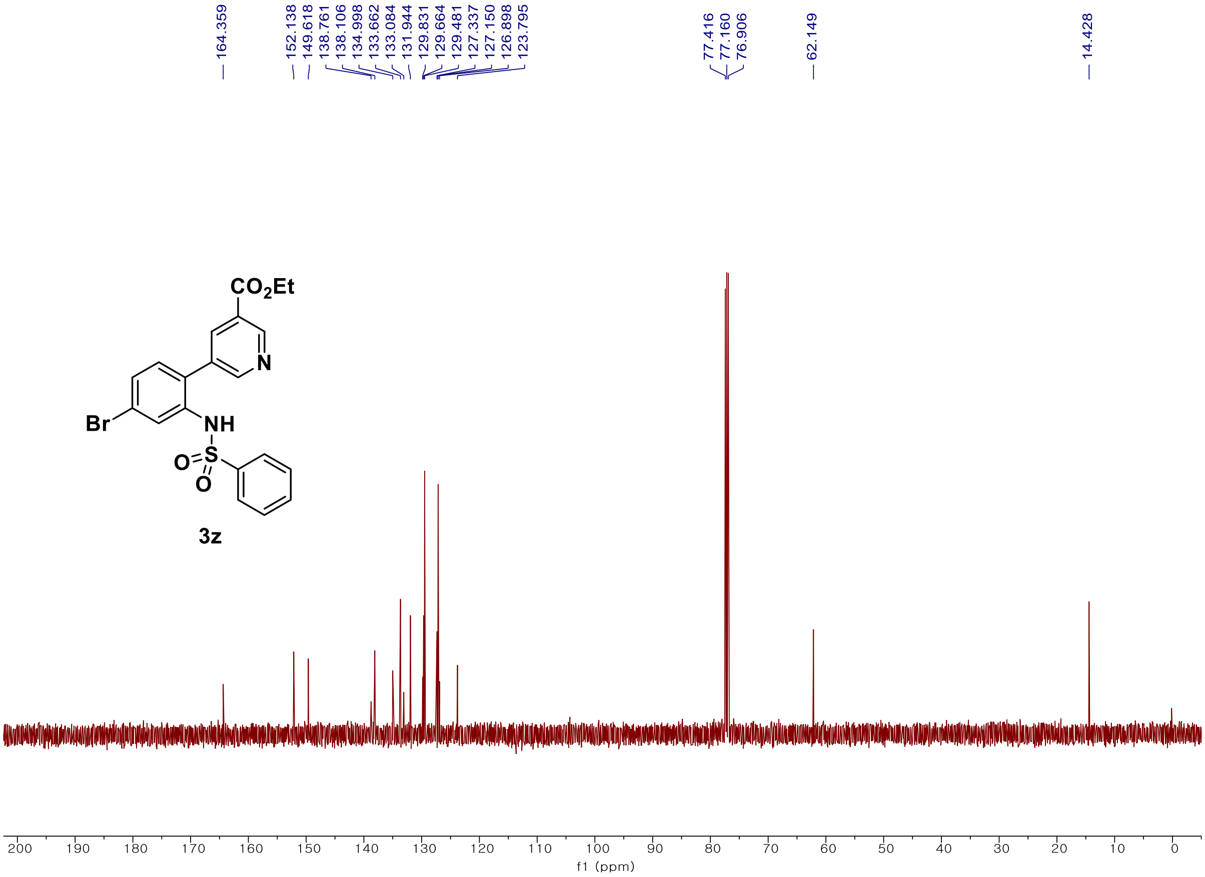
164.359
152.138
149.618
138.761
138.106
134.998
133.662
133.084
131.944
129.831
129.664
129.481
127.337
127.150
126.898
123.795
77.416
77.160
76.906
62.149
14.428
CO_2Et
N
Br
NH
O=S
O
3z
f1 (ppm)

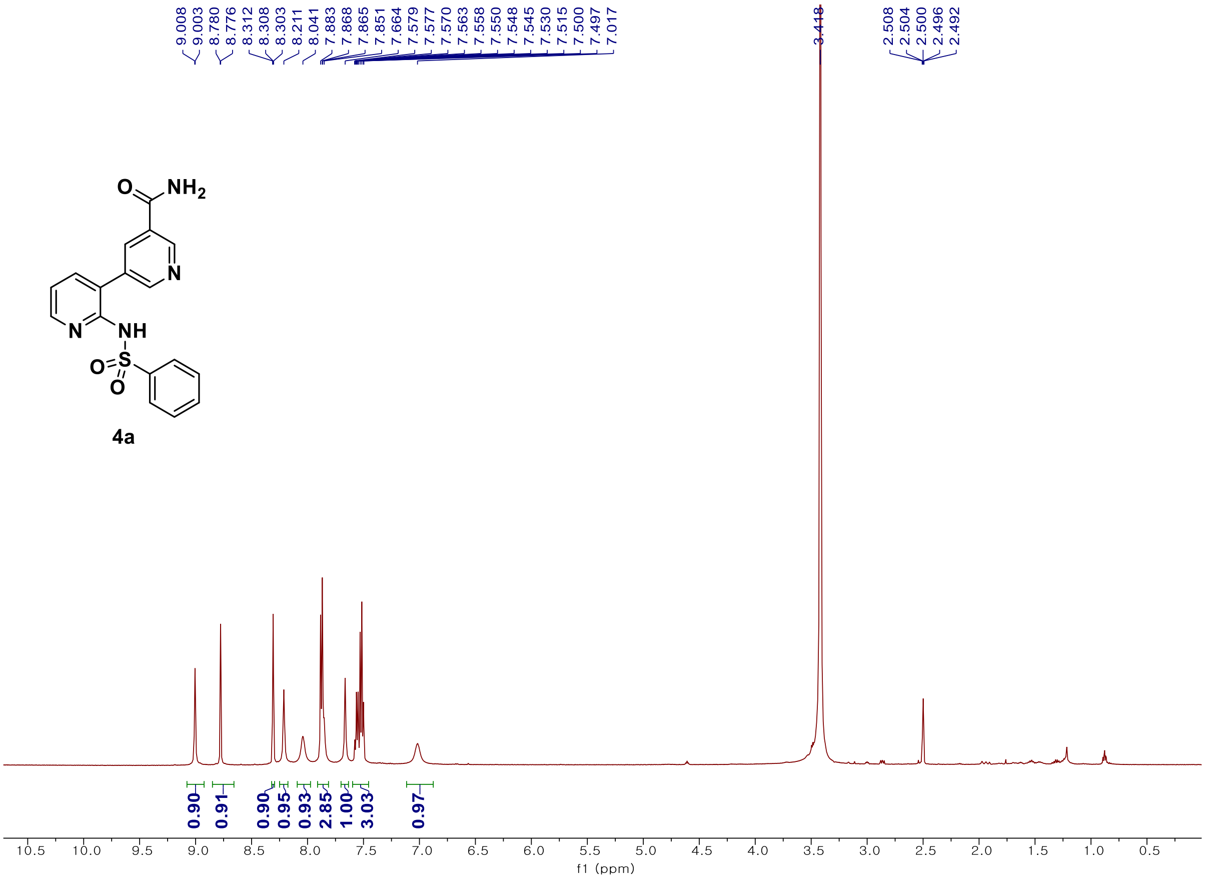

9.008
9.003
8.780
8.776
8.312
8.308
8.303
8.211
8.041
7.883
7.868
7.865
7.851
7.664
7.579
7.577
7.570
7.563
7.558
7.550
7.548
7.545
7.530
7.515
7.500
7.497
7.017
3.418
2.508
2.504
2.500
2.496
2.492
O
NH2
N
N
NH
O=S
O
4a
0.90
0.91
0.90
0.95
0.93
2.85
1.00
3.03
0.97
f1 (ppm)

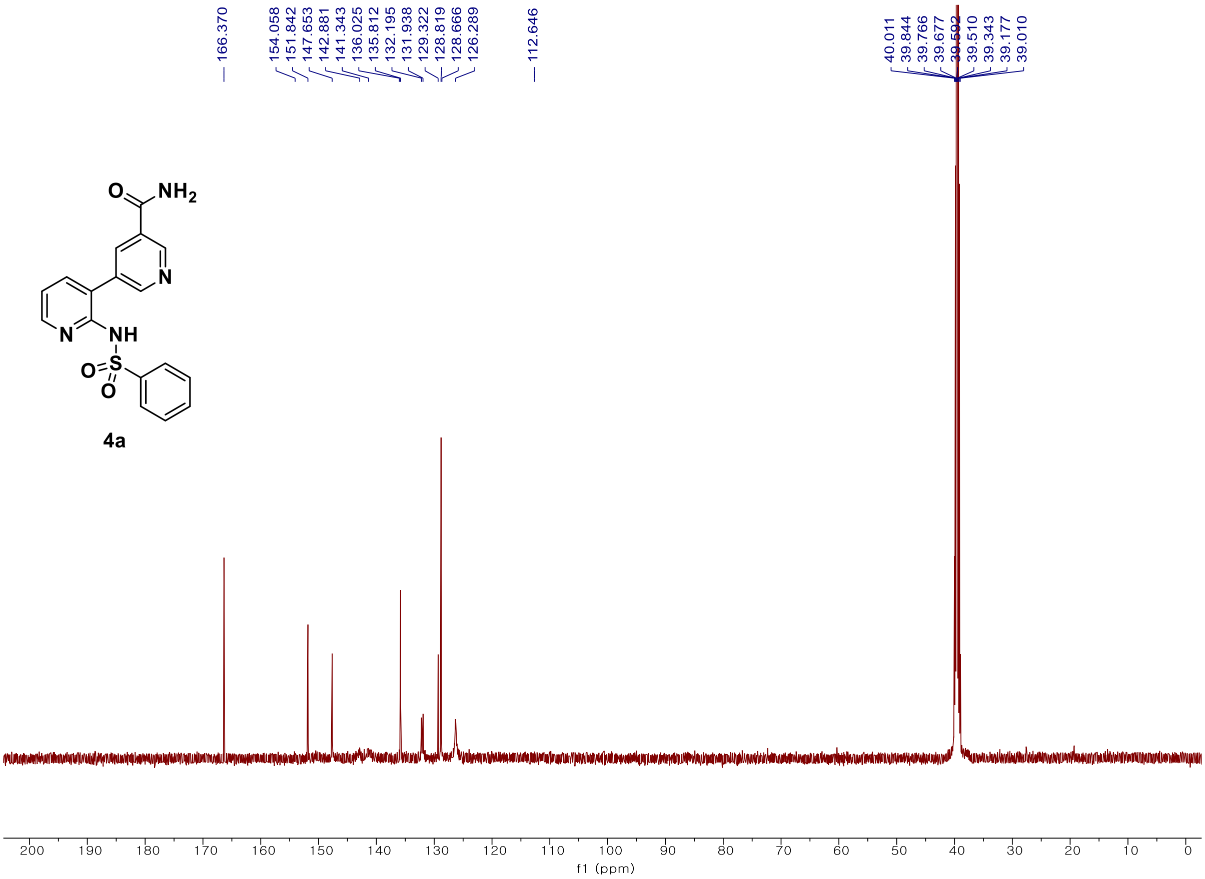

166.370
154.058
151.842
147.653
142.881
141.343
136.025
135.812
132.195
131.938
129.322
128.819
128.666
126.289
112.646
40.011
39.844
39.766
39.677
39.592
39.510
39.343
39.177
39.010
O
NH2
N
N
NH
O=S
O
4a
f1 (ppm)

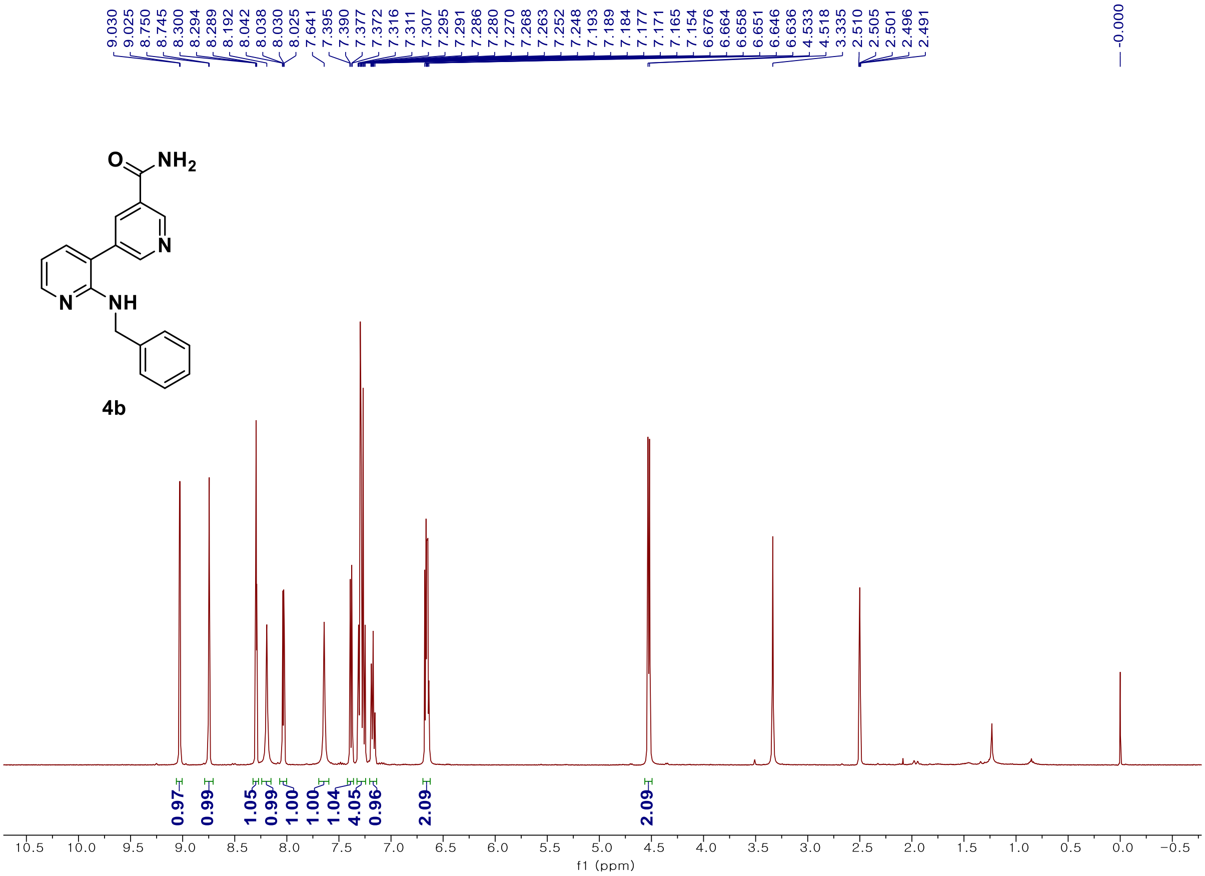

9.030 9.025 8.750 8.745 8.300 8.294 8.289 8.192 8.042 8.038 8.030 8.025 7.641 7.395 7.390 7.377 7.372 7.316 7.311 7.307 7.295 7.291 7.286 7.280 7.270 7.268 7.263 7.252 7.248 7.193 7.189 7.184 7.177 7.171 7.165 7.154 6.676 6.664 6.658 6.651 6.646 6.636 4.533 4.518 3.335 2.510 2.505 2.501 2.496 2.491
-0.000
O NH2
N
N NH
4b
0.97 0.99 1.05 0.99 1.00 1.00 1.04 4.05 0.96 2.09 2.09
10.5 10.0 9.5 9.0 8.5 8.0 7.5 7.0 6.5 6.0 5.5 5.0 4.5 4.0 3.5 3.0 2.5 2.0 1.5 1.0 0.5 0.0 -0.5
f1 (ppm)

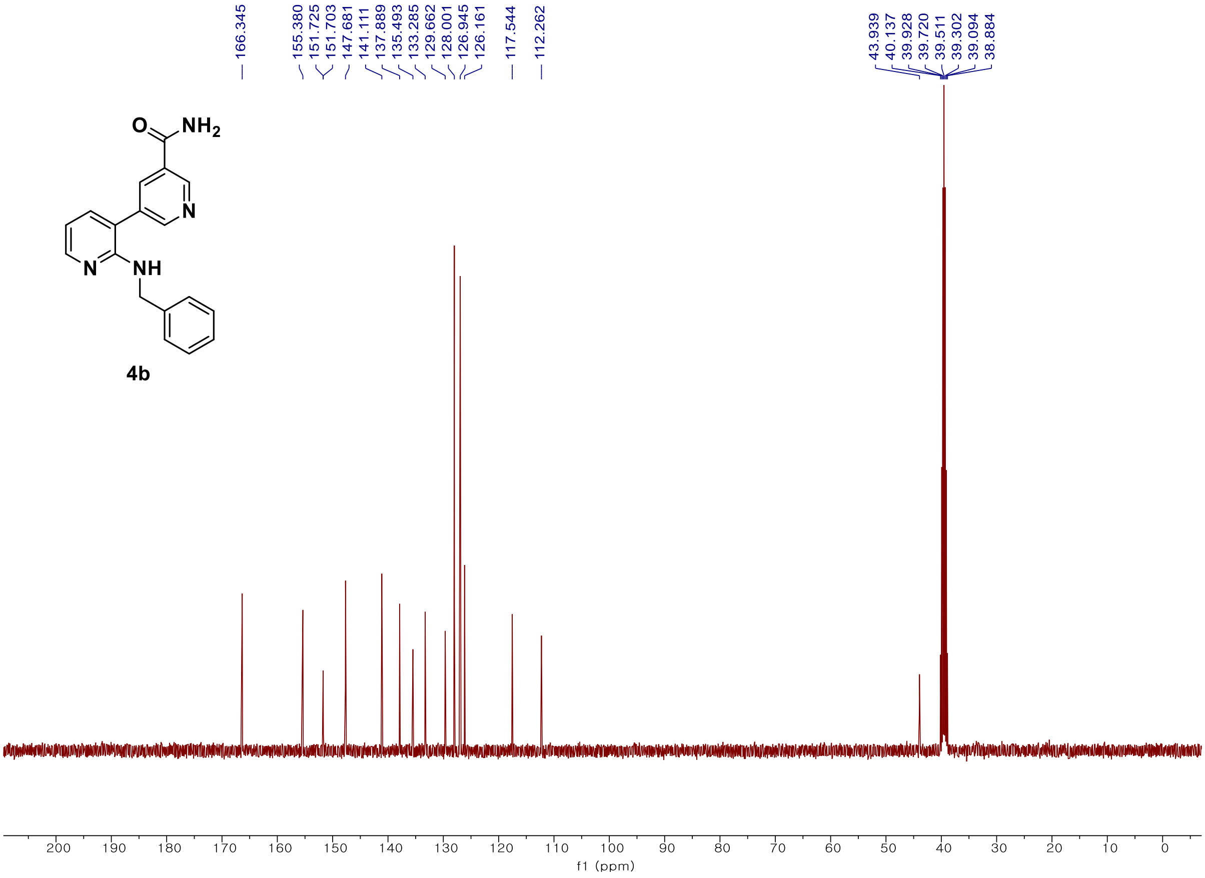

166.345
155.380 151.725 151.703 147.681 141.111 137.889 135.493 133.285 129.662 128.001 126.945 126.161
117.544
112.262
43.939 40.137 39.928 39.720 39.511 39.302 39.094 38.884
O NH2
N
N NH
4b
200 190 180 170 160 150 140 130 120 110 100 90 80 70 60 50 40 30 20 10 0
f1 (ppm)

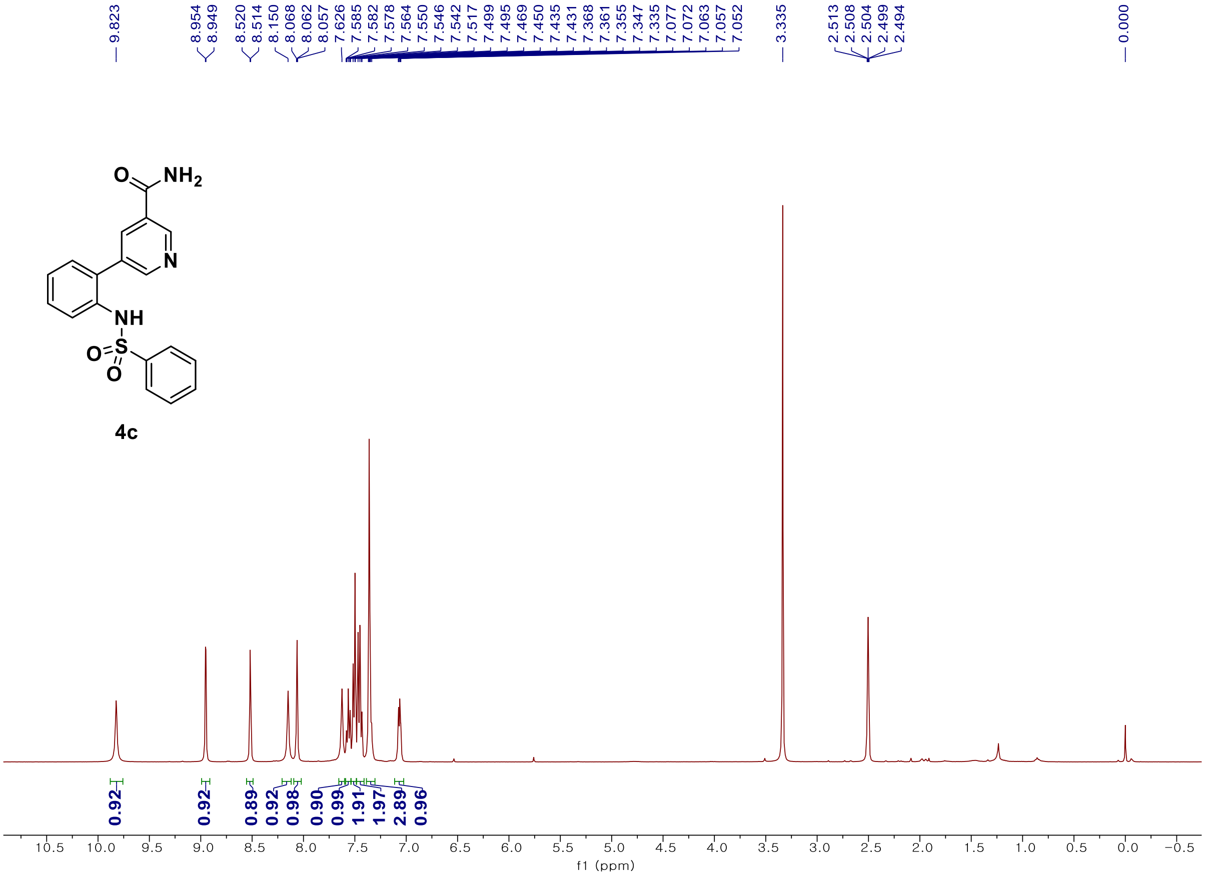
9.823
8.954
8.949
8.520
8.514
8.150
8.068
8.062
8.057
7.626
7.585
7.582
7.578
7.564
7.550
7.546
7.542
7.517
7.499
7.495
7.469
7.450
7.435
7.431
7.368
7.361
7.355
7.347
7.335
7.077
7.072
7.063
7.057
7.052
3.335
2.513
2.508
2.504
2.499
2.494
0.000
O
NH2
N
NH
O=S
O
4c
0.92
0.92
0.89
0.92
0.98
0.90
0.99
1.91
1.97
2.89
0.96
10.5
10.0
9.5
9.0
8.5
8.0
7.5
7.0
6.5
6.0
5.5
5.0
4.5
4.0
3.5
3.0
2.5
2.0
1.5
1.0
0.5
0.0
−0.5
f1 (ppm)

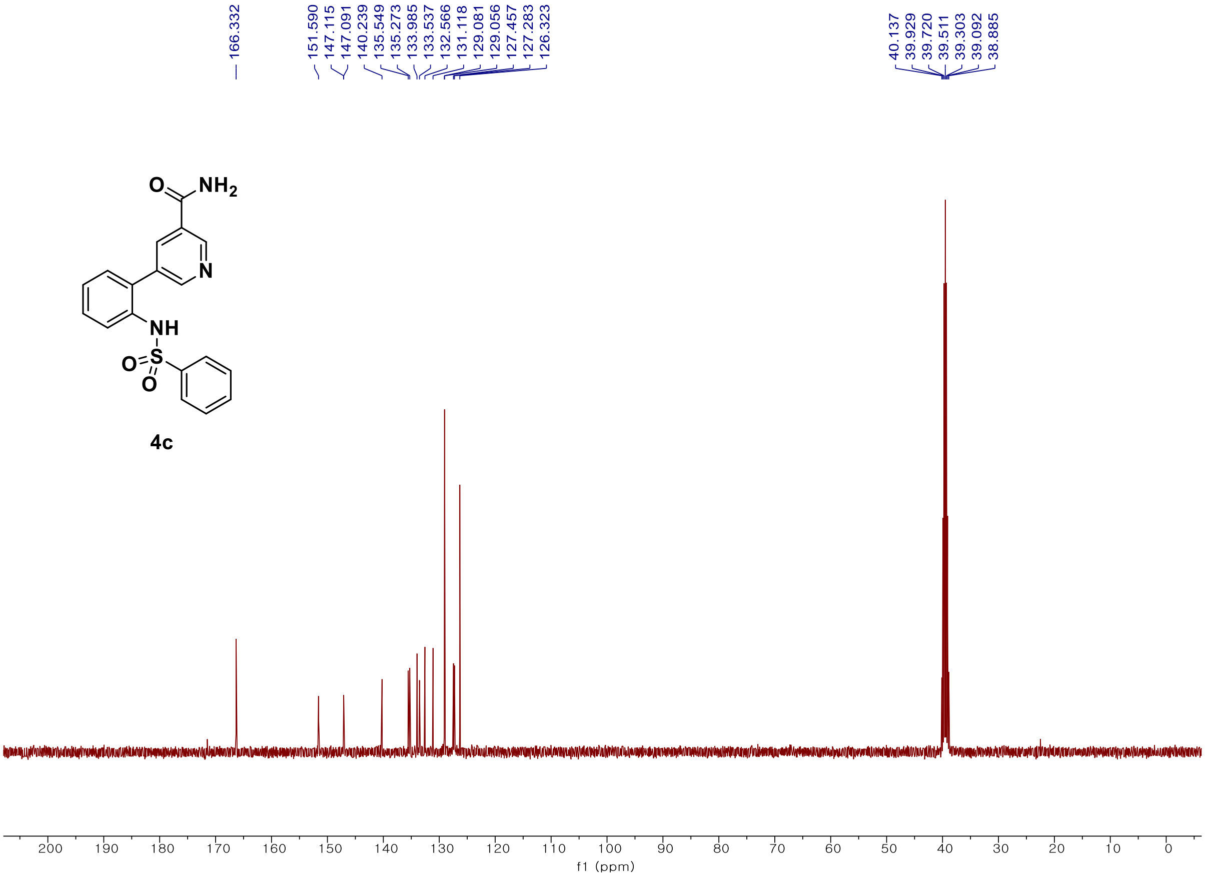
166.332
151.590
147.115
147.091
140.239
135.549
135.273
133.985
133.537
132.566
131.118
129.081
129.056
127.457
127.283
126.323
40.137
39.929
39.720
39.511
39.303
39.092
38.885
O
NH2
N
NH
O=S
O
4c
200
190
180
170
160
150
140
130
120
110
100
90
80
70
60
50
40
30
20
10
0
f1 (ppm)

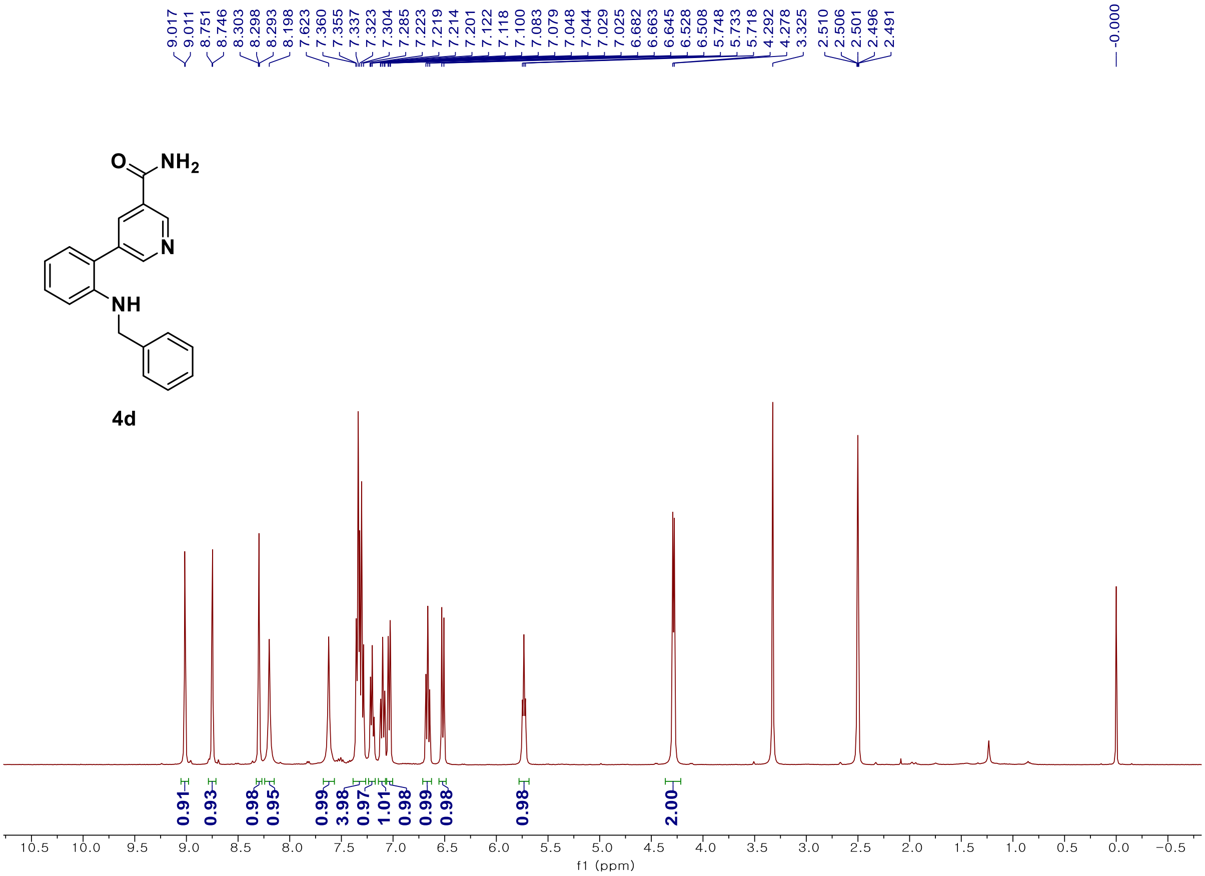
9.017
9.011
8.751
8.746
8.303
8.298
8.293
8.198
7.623
7.360
7.355
7.337
7.323
7.304
7.285
7.223
7.219
7.214
7.201
7.122
7.118
7.100
7.083
7.079
7.048
7.044
7.029
7.025
6.682
6.663
6.645
6.528
6.508
5.748
5.733
5.718
4.292
4.278
3.325
2.510
2.506
2.501
2.496
2.491
-0.000
O
NH2
N
NH
4d
0.91
0.93
0.98
0.95
0.99
3.98
0.97
1.01
0.98
0.99
0.98
0.98
2.00
f1 (ppm)

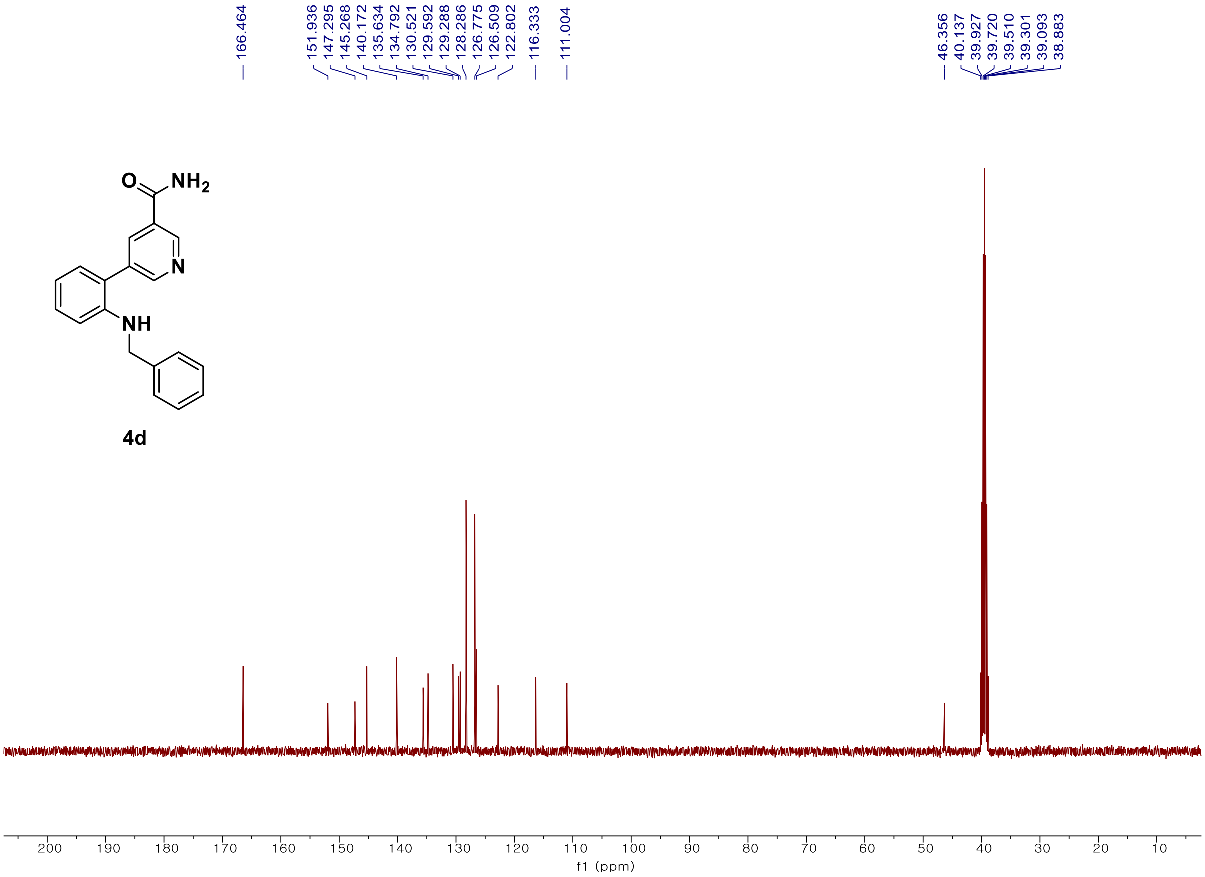
166.464
151.936
147.295
145.268
140.172
135.634
134.792
130.521
129.592
129.288
128.286
126.775
126.509
122.802
116.333
111.004
46.356
40.137
39.927
39.720
39.510
39.301
39.093
38.883
O
NH2
N
NH
4d
f1 (ppm)

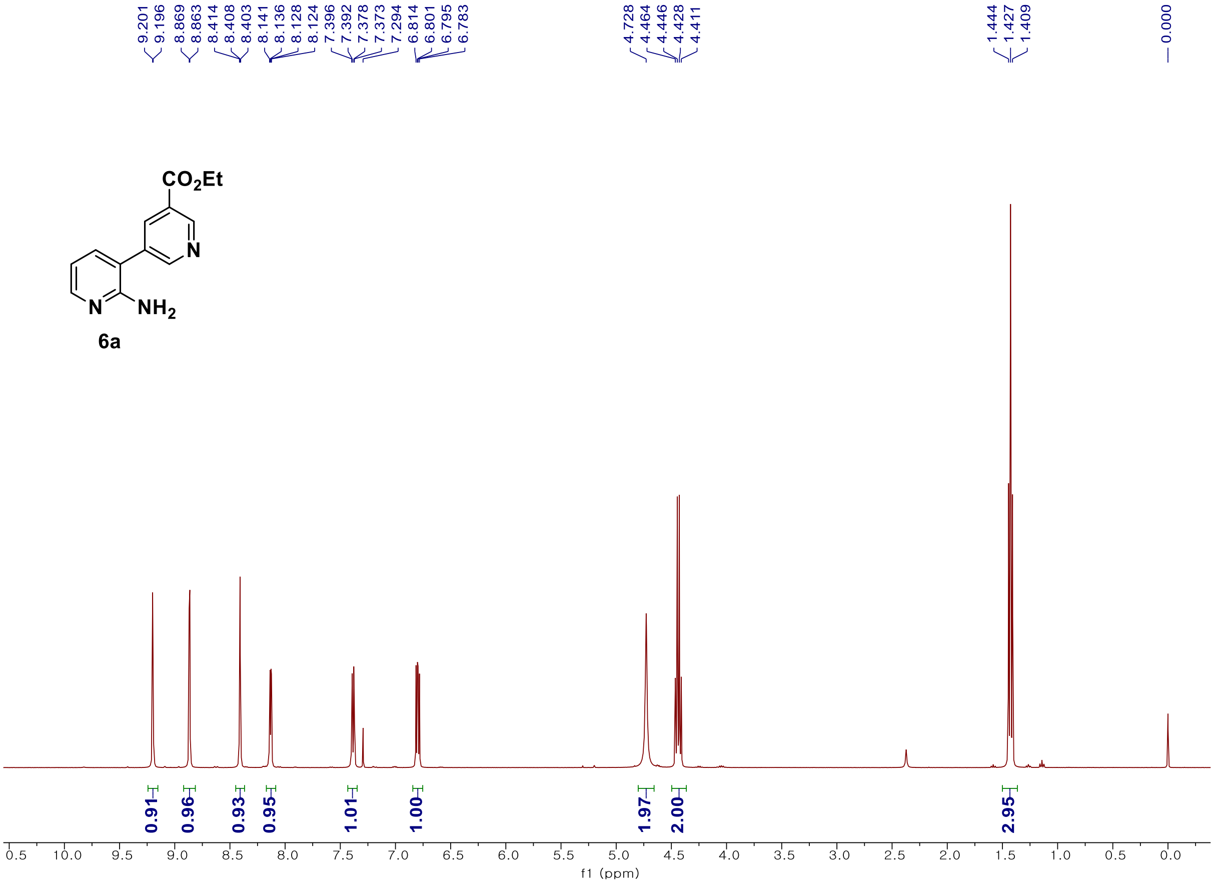
9.201
9.196
8.869
8.863
8.414
8.408
8.403
8.141
8.136
8.128
8.124
7.396
7.392
7.378
7.373
7.294
6.814
6.801
6.795
6.783
4.728
4.464
4.446
4.428
4.411
1.444
1.427
1.409
0.000
CO_2Et
N
N
NH_2
6a
0.91
0.96
0.93
0.95
1.01
1.00
1.97
2.00
2.95
0.5 10.0 9.5 9.0 8.5 8.0 7.5 7.0 6.5 6.0 5.5 5.0 4.5 4.0 3.5 3.0 2.5 2.0 1.5 1.0 0.5 0.0
f1 (ppm)

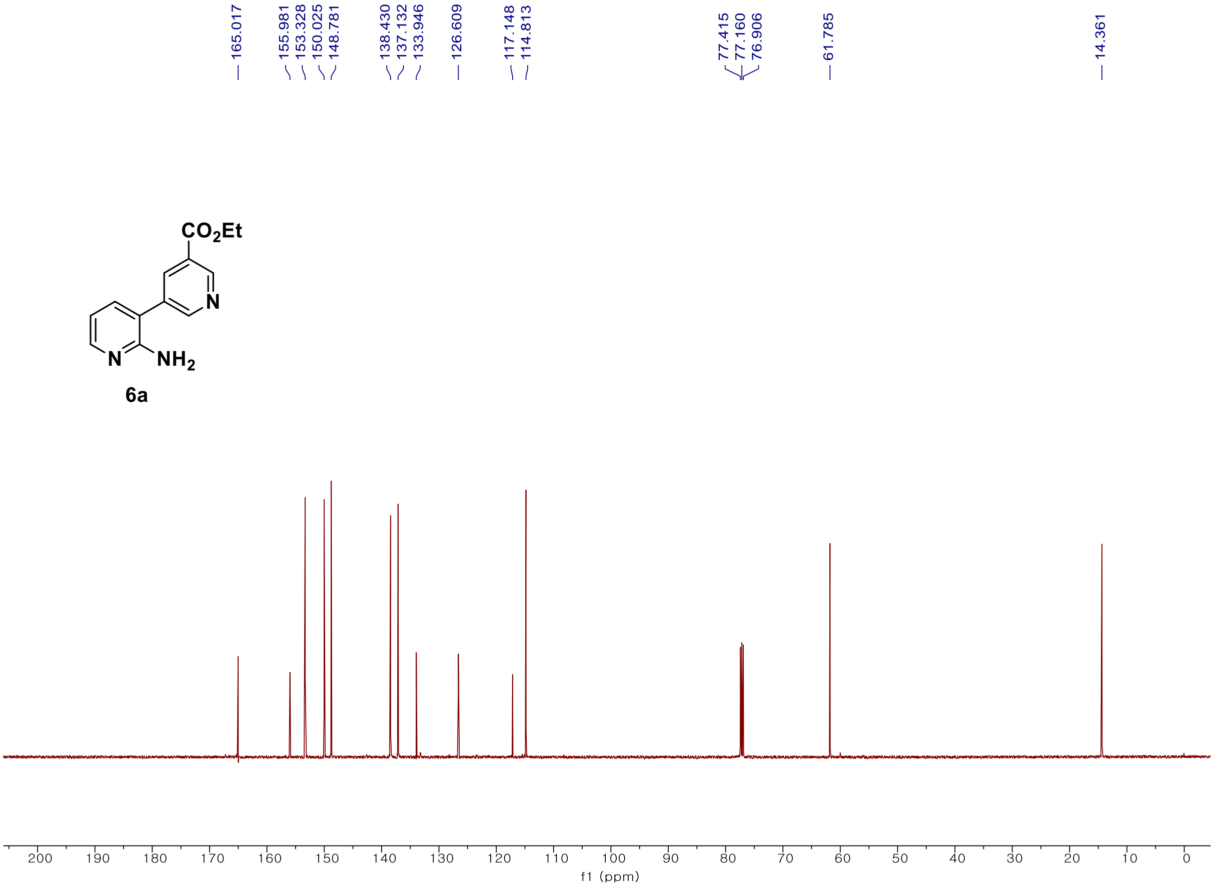
165.017
155.981
153.328
150.025
148.781
138.430
137.132
133.946
126.609
117.148
114.813
77.415
77.160
76.906
61.785
14.361
CO_2Et
N
N
NH_2
6a
200 190 180 170 160 150 140 130 120 110 100 90 80 70 60 50 40 30 20 10 0
f1 (ppm)

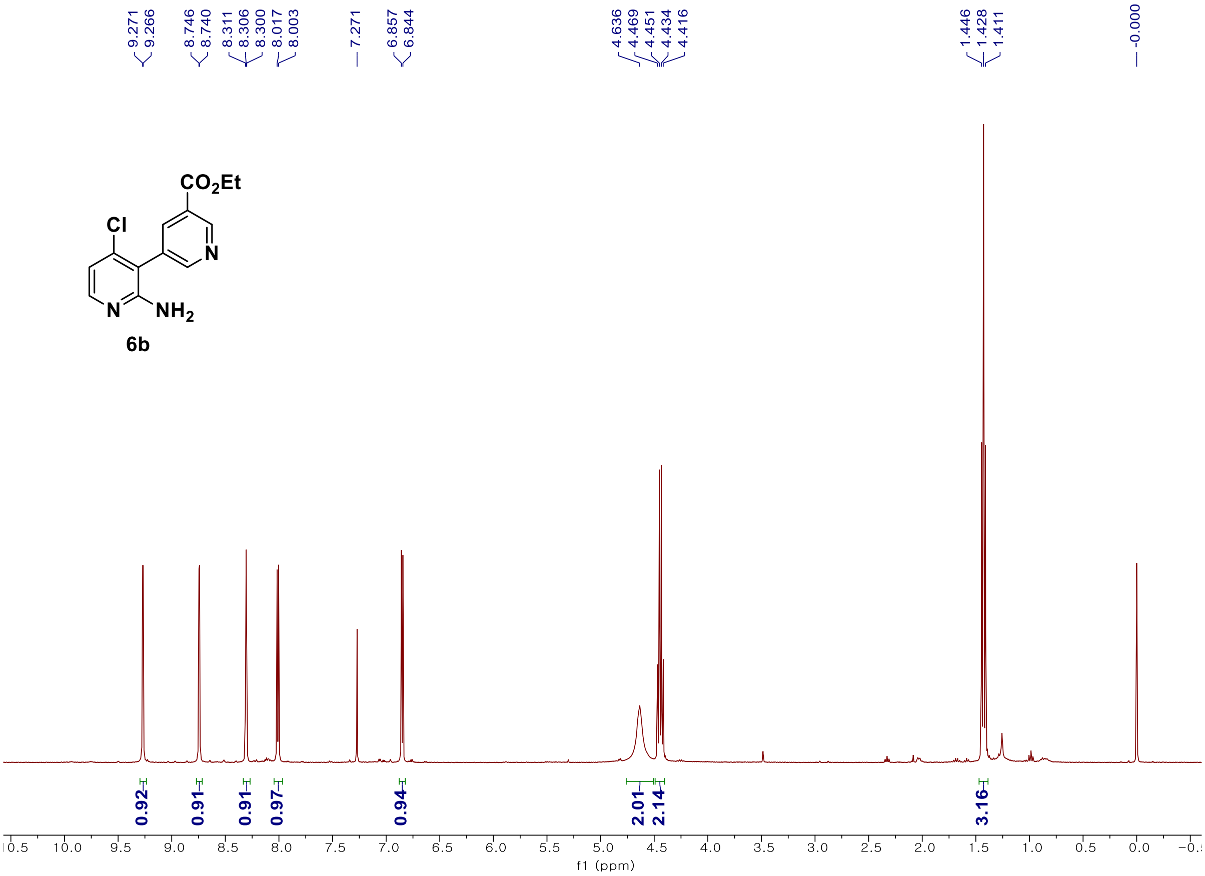
9.271
9.266
8.746
8.740
8.311
8.306
8.300
8.017
8.003
7.271
6.857
6.844
4.636
4.469
4.451
4.434
4.416
1.446
1.428
1.411
-0.000
CO2Et
Cl
N
N
NH2
6b
0.92
0.91
0.91
0.97
0.94
2.01
2.14
3.16
f1 (ppm)

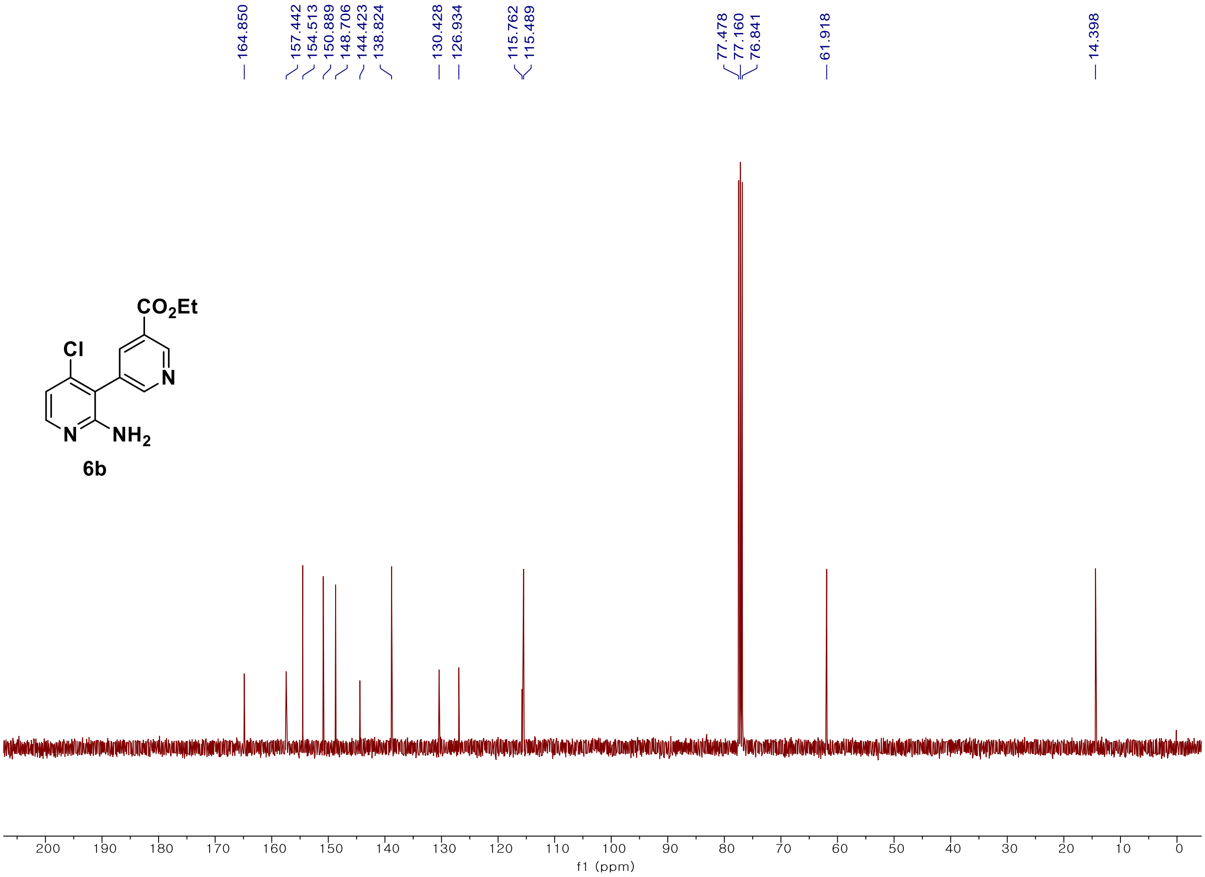
164.850
157.442
154.513
150.889
148.706
144.423
138.824
130.428
126.934
115.762
115.489
77.478
77.160
76.841
61.918
14.398
CO2Et
Cl
N
N
NH2
6b
f1 (ppm)

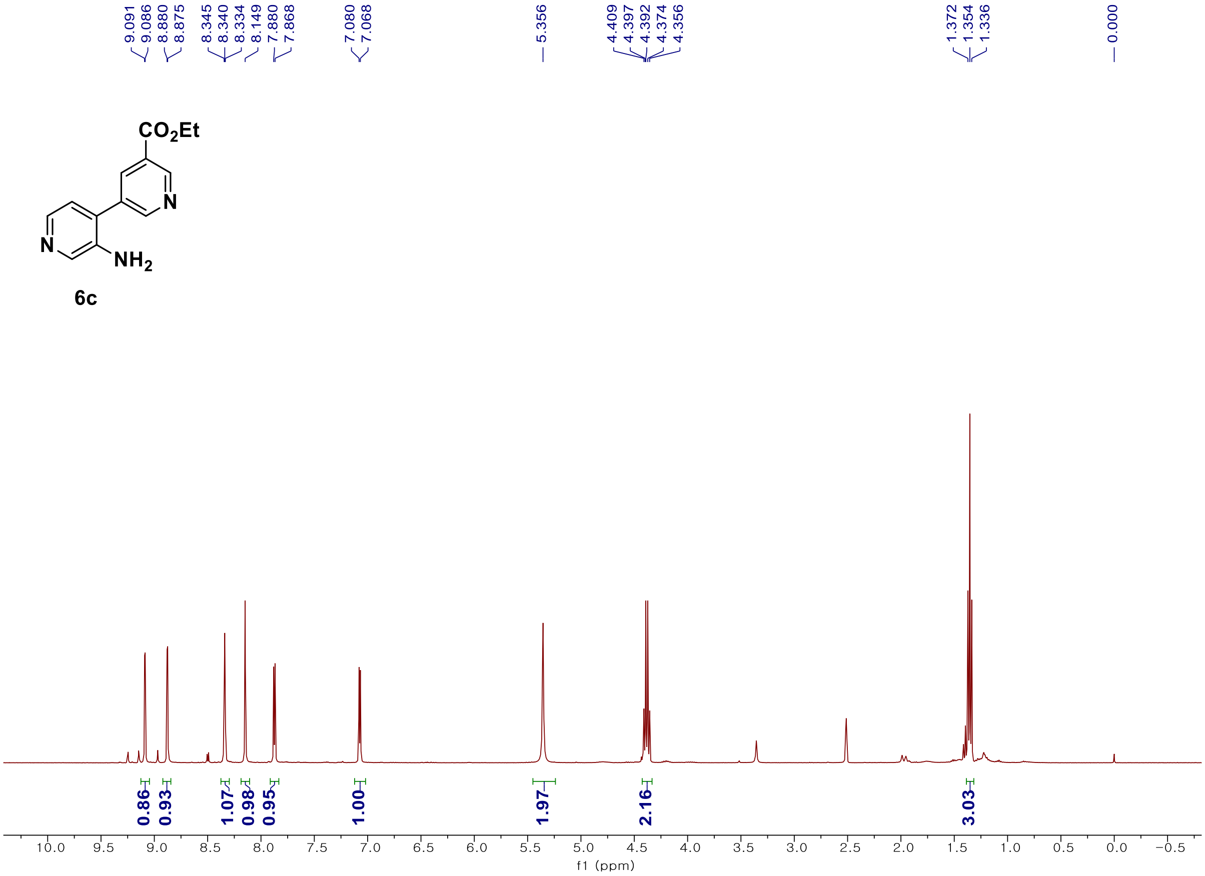
9.091
9.086
8.880
8.875
8.345
8.340
8.334
8.149
7.880
7.868
7.080
7.068
5.356
4.409
4.397
4.392
4.374
4.356
1.372
1.354
1.336
0.000
CO2Et
N
N
NH2
6c
0.86
0.93
1.07
0.98
0.95
1.00
1.97
2.16
3.03
f1 (ppm)

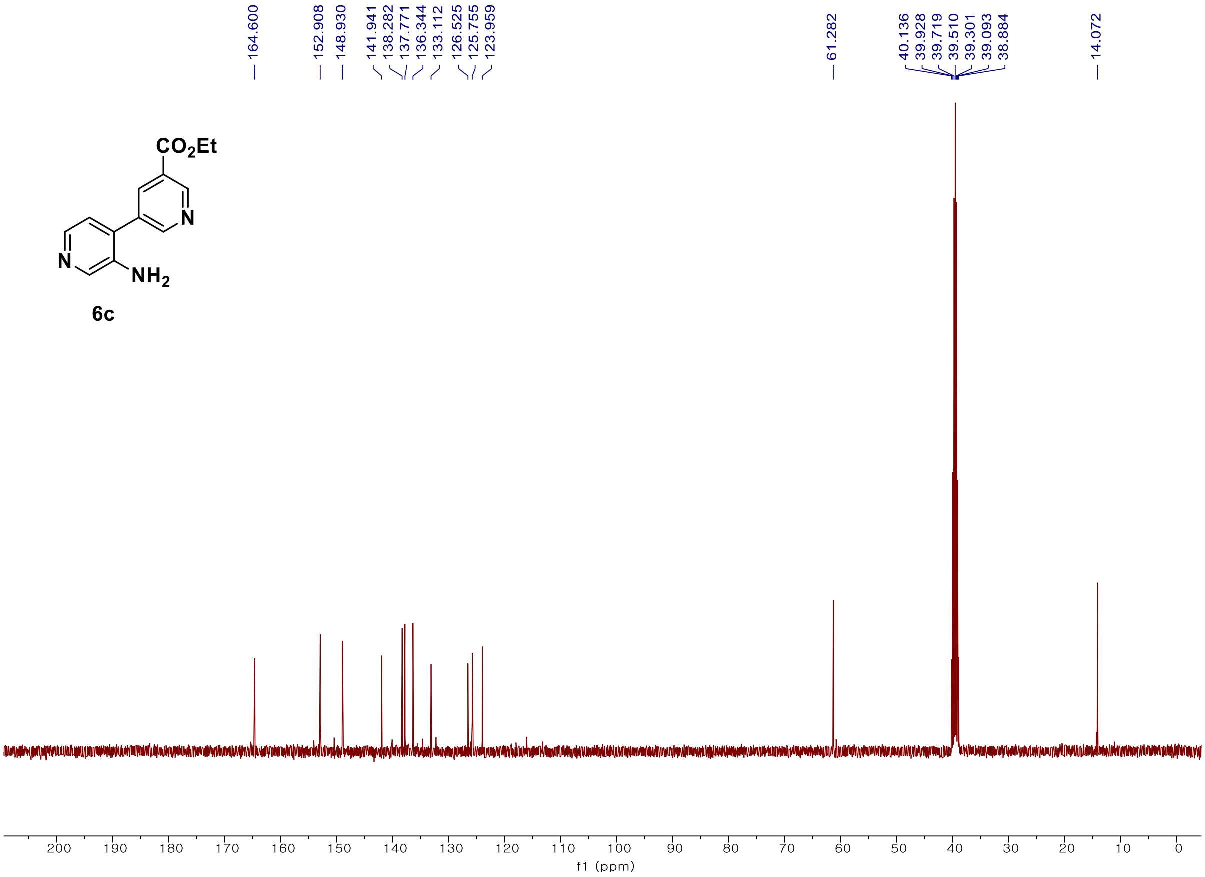
164.600
152.908
148.930
141.941
138.282
137.771
136.344
133.112
126.525
125.755
123.959
61.282
40.136
39.928
39.719
39.510
39.301
39.093
38.884
14.072
CO2Et
N
N
NH2
6c
f1 (ppm)

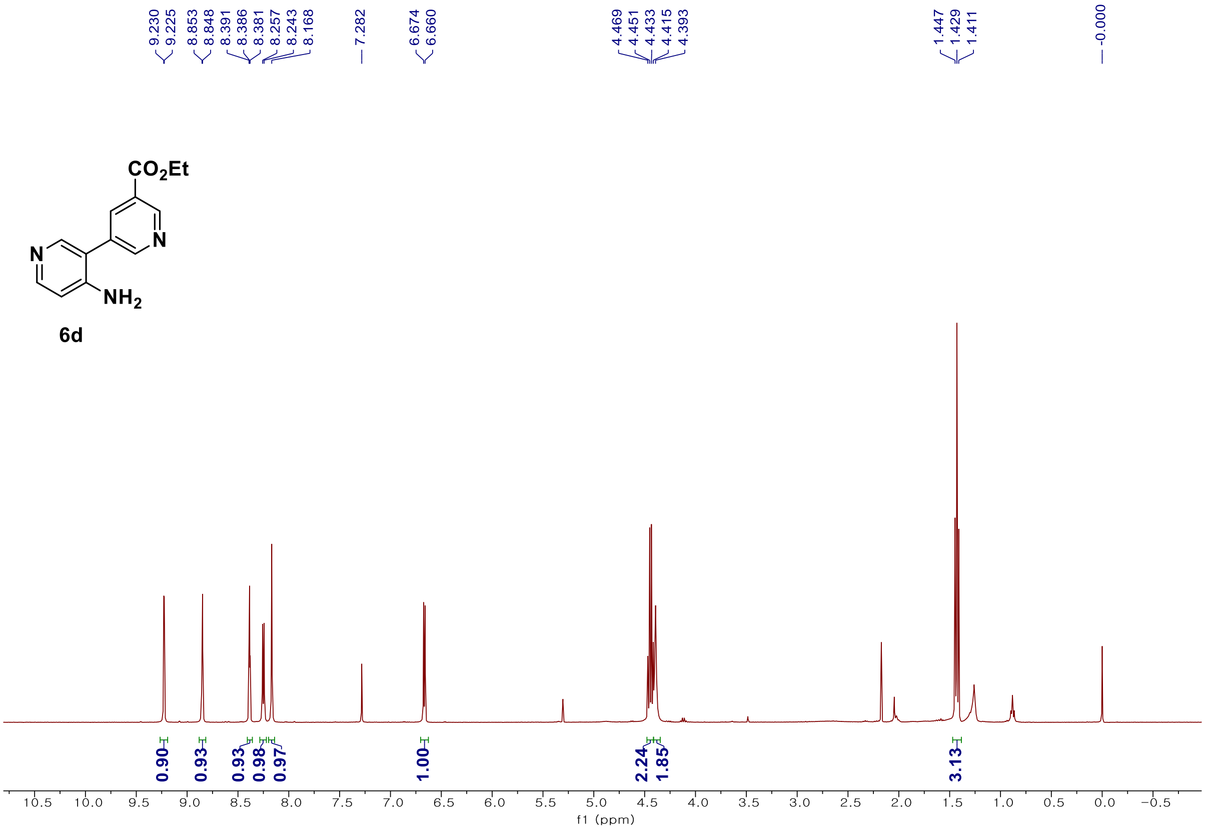

9.230
9.225
8.853
8.848
8.391
8.386
8.381
8.257
8.243
8.168
7.282
6.674
6.660
4.469
4.451
4.433
4.415
4.393
1.447
1.429
1.411
-0.000
CO2Et
N
N
NH2
6d
0.90
0.93
0.93
0.98
0.97
1.00
2.24
1.85
3.13
10.5 10.0 9.5 9.0 8.5 8.0 7.5 7.0 6.5 6.0 5.5 5.0 4.5 4.0 3.5 3.0 2.5 2.0 1.5 1.0 0.5 0.0 -0.5
f1 (ppm)

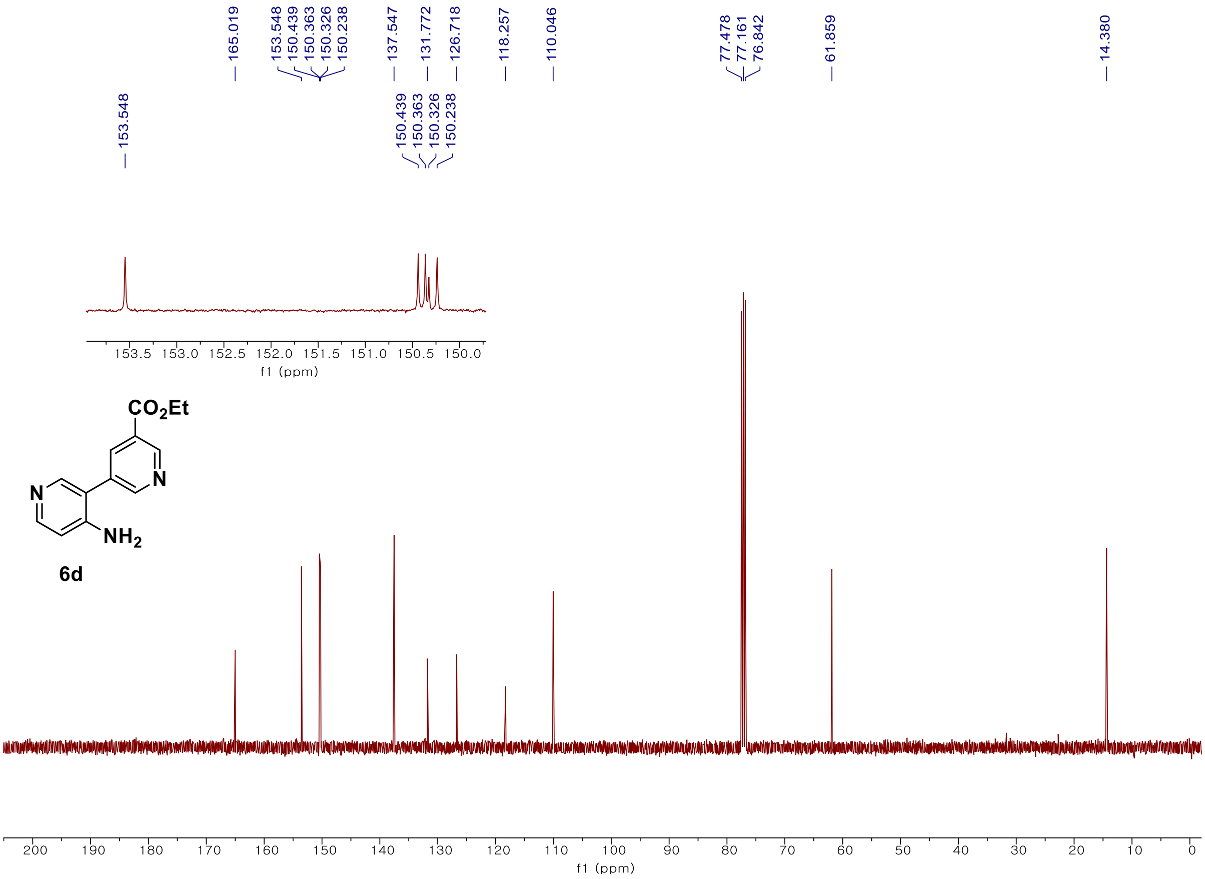

165.019
153.548
150.439
150.363
150.326
150.238
137.547
131.772
126.718
118.257
110.046
77.478
77.161
76.842
61.859
14.380
153.548
150.439
150.363
150.326
150.238
153.5 153.0 152.5 152.0 151.5 151.0 150.5 150.0
f1 (ppm)
CO2Et
N
N
NH2
6d
200 190 180 170 160 150 140 130 120 110 100 90 80 70 60 50 40 30 20 10 0
f1 (ppm)

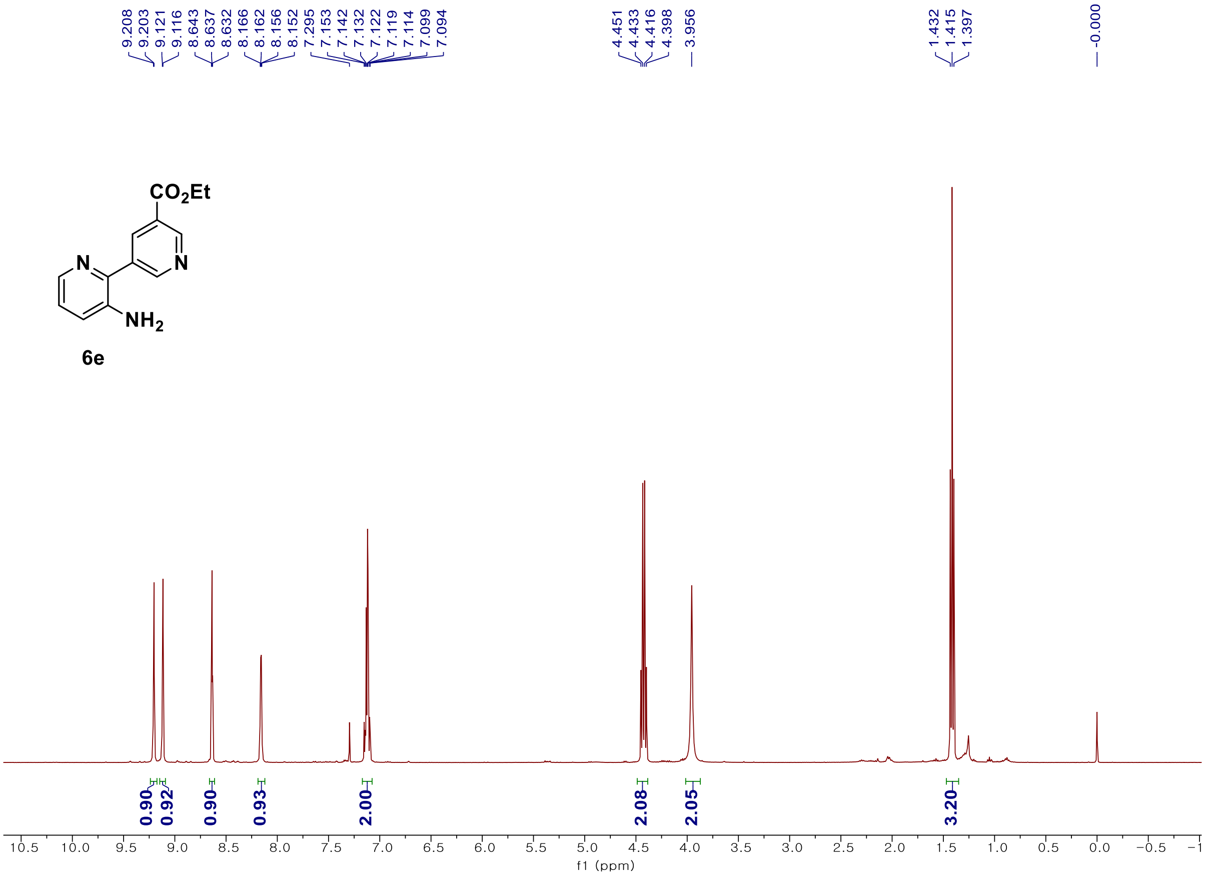
9.208
9.203
9.121
9.116
8.643
8.637
8.632
8.166
8.162
8.156
8.152
7.295
7.153
7.142
7.132
7.122
7.119
7.114
7.099
7.094
4.451
4.433
4.416
4.398
3.956
1.432
1.415
1.397
-0.000
CO2Et
N
N
NH2
6e
0.90
0.92
0.90
0.93
2.00
2.08
2.05
3.20
f1 (ppm)

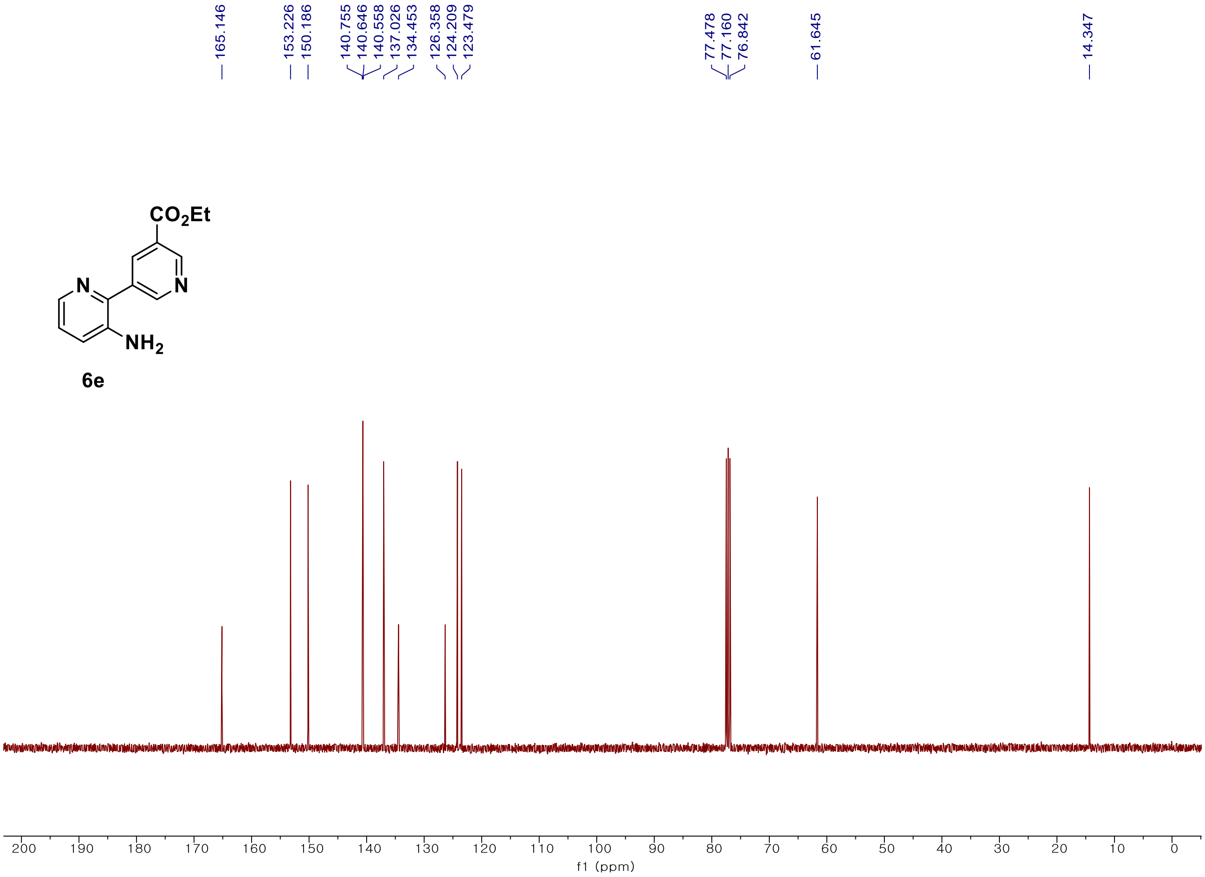
165.146
153.226
150.186
140.755
140.646
140.558
137.026
134.453
126.358
124.209
123.479
77.478
77.160
76.842
61.645
14.347
CO2Et
N
N
NH2
6e
f1 (ppm)

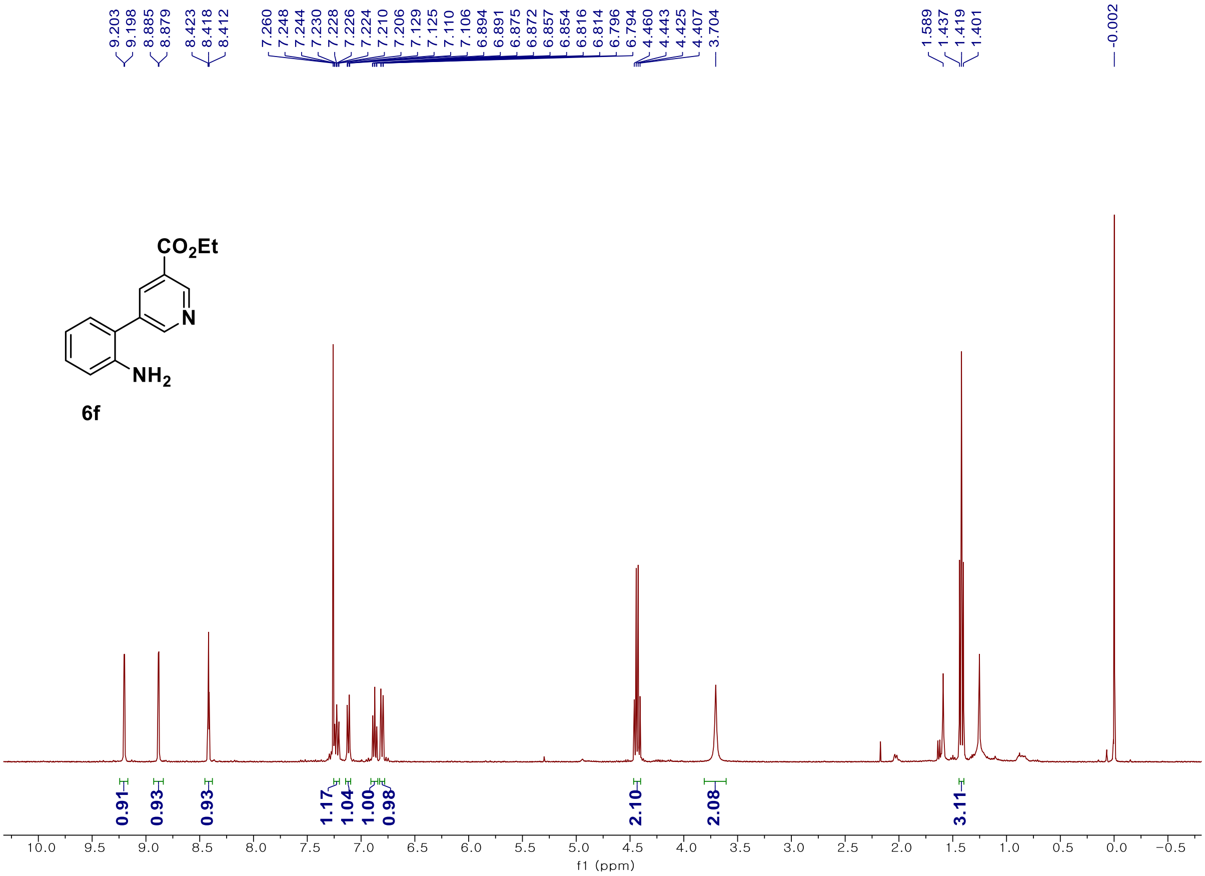
9.203
9.198
8.885
8.879
8.423
8.418
8.412
7.260
7.248
7.244
7.230
7.228
7.226
7.224
7.210
7.206
7.129
7.125
7.110
7.106
6.894
6.891
6.875
6.872
6.857
6.854
6.816
6.814
6.796
6.794
4.460
4.443
4.425
4.407
3.704
1.589
1.437
1.419
1.401
-0.002
CO2Et
N
NH2
6f
0.91
0.93
0.93
1.17
1.04
1.00
0.98
2.10
2.08
3.11
f1 (ppm)

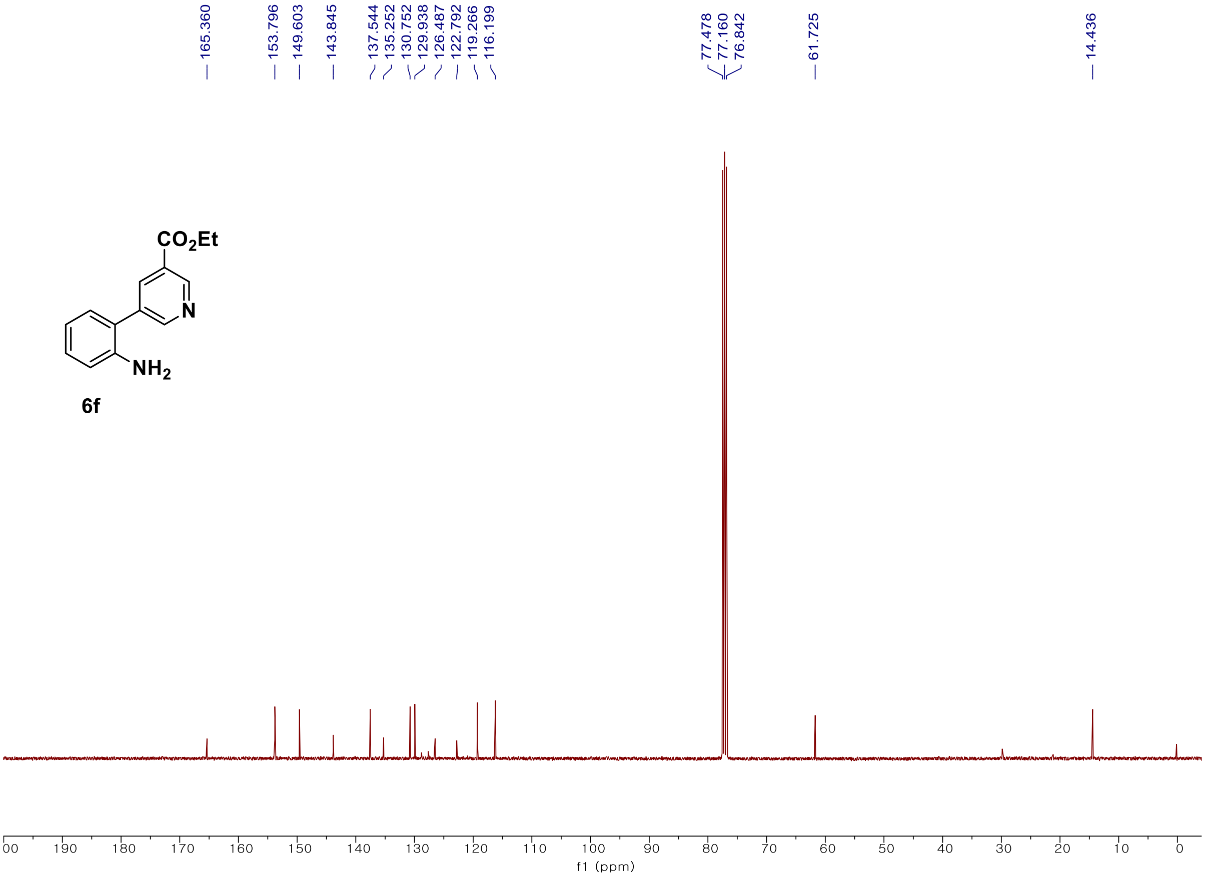
165.360
153.796
149.603
143.845
137.544
135.252
130.752
129.938
126.487
122.792
119.266
116.199
77.478
77.160
76.842
61.725
14.436
CO2Et
N
NH2
6f
f1 (ppm)

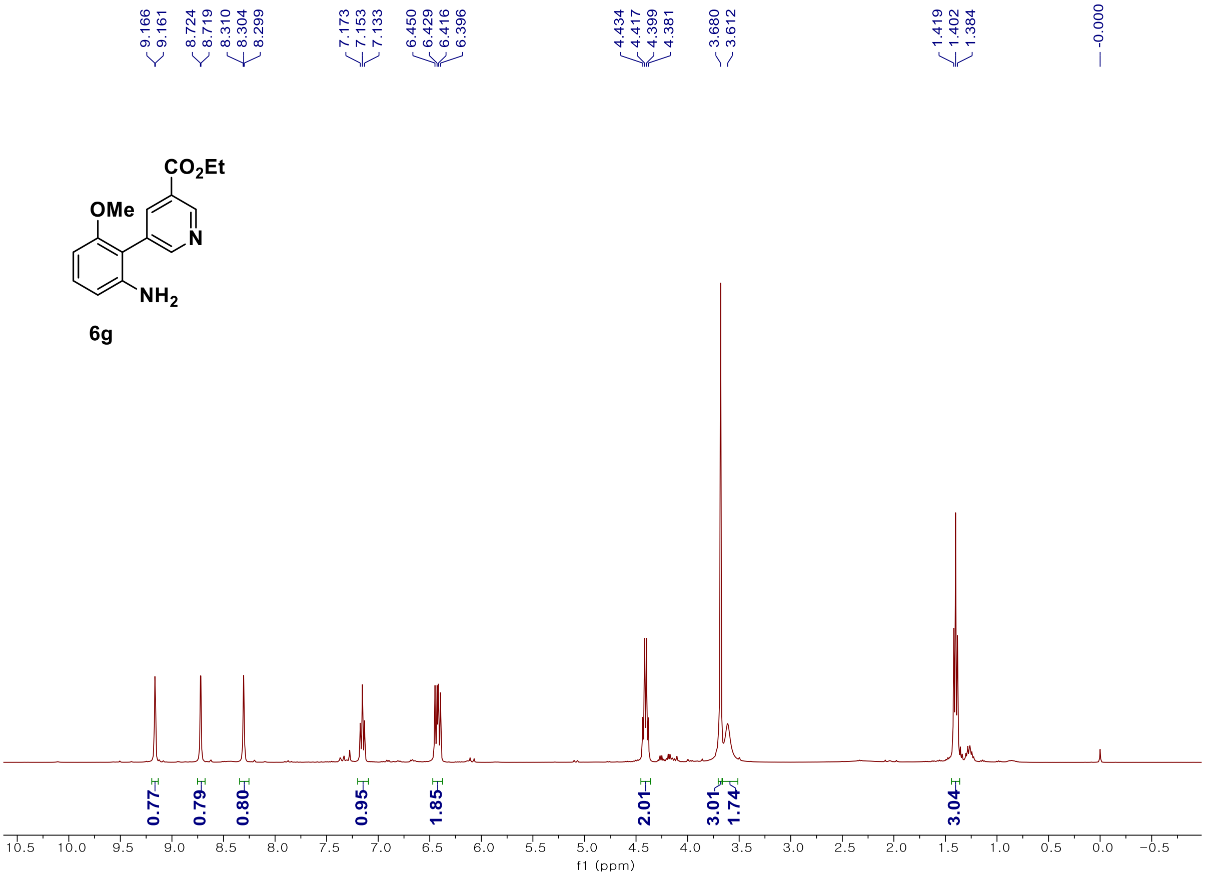
9.166
9.161
8.724
8.719
8.310
8.304
8.299
7.173
7.153
7.133
6.450
6.429
6.416
6.396
4.434
4.417
4.399
4.381
3.680
3.612
1.419
1.402
1.384
-0.000
CO2Et
OMe
N
NH2
6g
0.77
0.79
0.80
0.95
1.85
2.01
3.01
1.74
3.04
f1 (ppm)

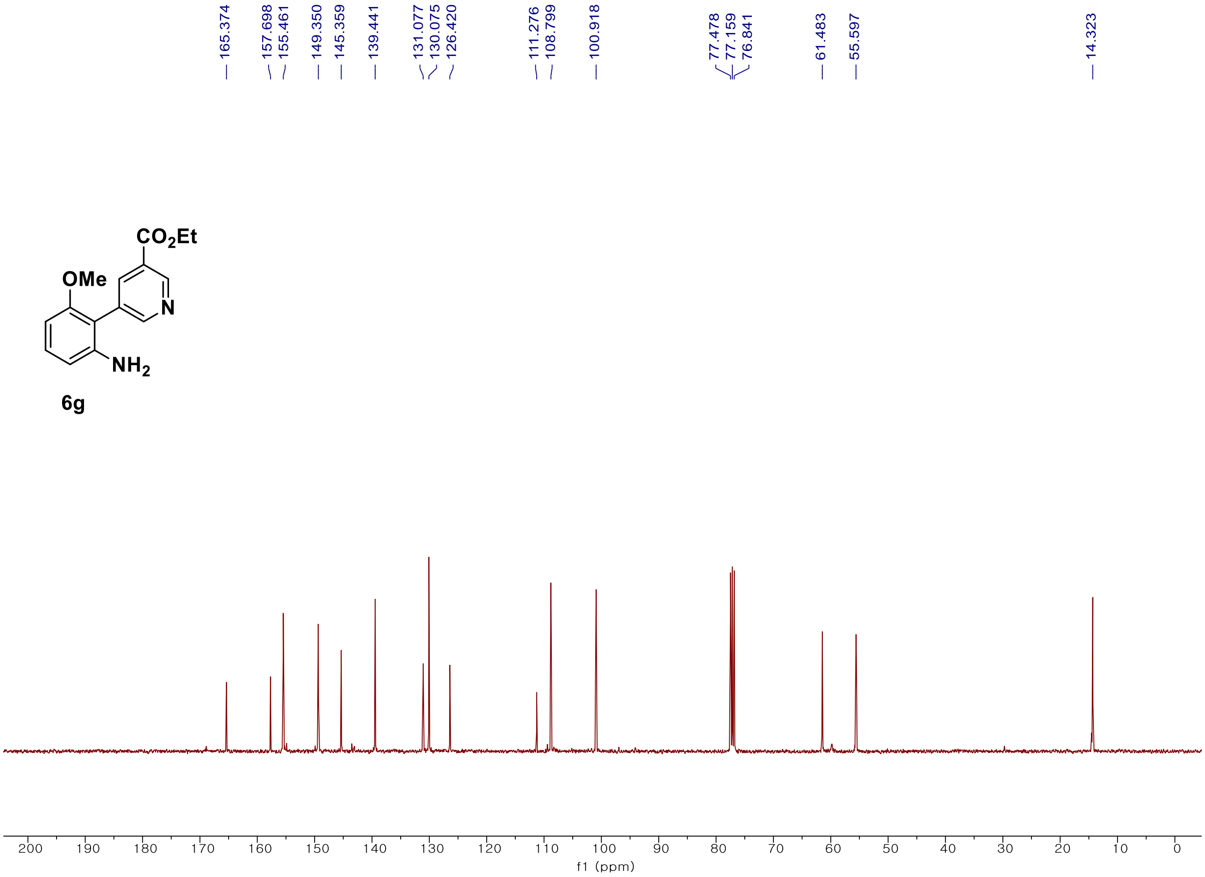
165.374
157.698
155.461
149.350
145.359
139.441
131.077
130.075
126.420
111.276
108.799
100.918
77.478
77.159
76.841
61.483
55.597
14.323
CO2Et
OMe
N
NH2
6g
f1 (ppm)

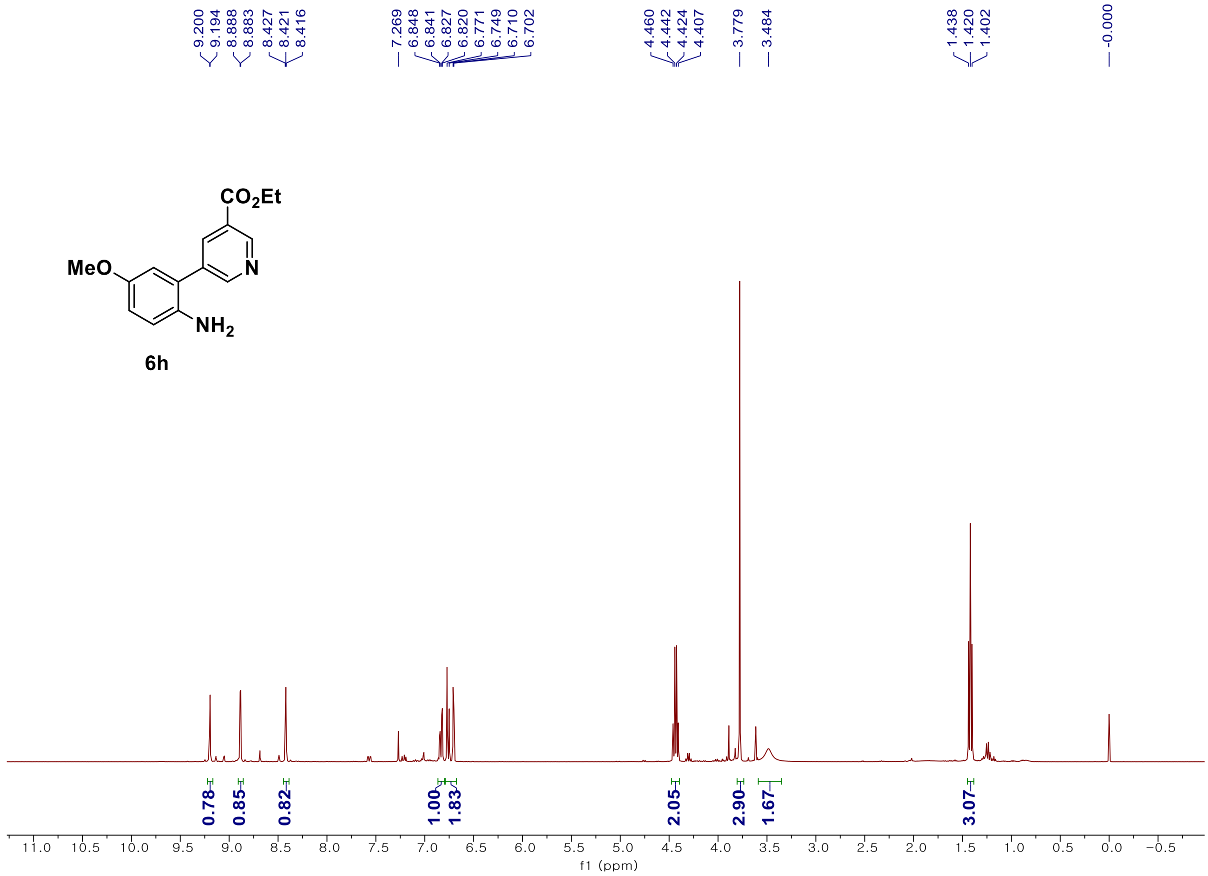
9.200
9.194
8.888
8.883
8.427
8.421
8.416
7.269
6.848
6.841
6.827
6.820
6.771
6.749
6.710
6.702
4.460
4.442
4.424
4.407
3.779
3.484
1.438
1.420
1.402
-0.000
CO2Et
MeO
N
NH2
6h
0.78
0.85
0.82
1.00
1.83
2.05
2.90
1.67
3.07
f1 (ppm)

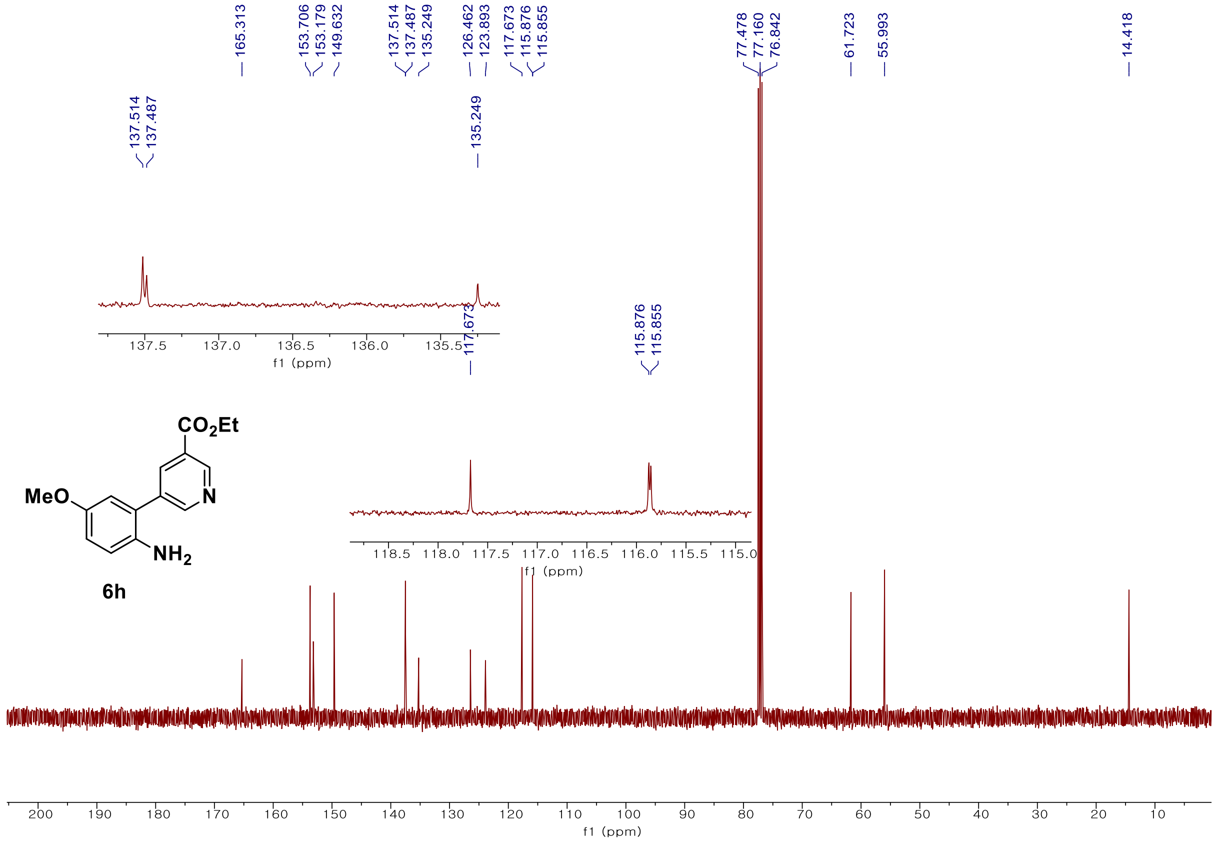
165.313
153.706
153.179
149.632
137.514
137.487
135.249
126.462
123.893
117.673
115.876
115.855
77.478
77.160
76.842
61.723
55.993
14.418
137.514
137.487
135.249
f1 (ppm)
117.673
115.876
115.855
f1 (ppm)
CO2Et
MeO
N
NH2
6h
f1 (ppm)

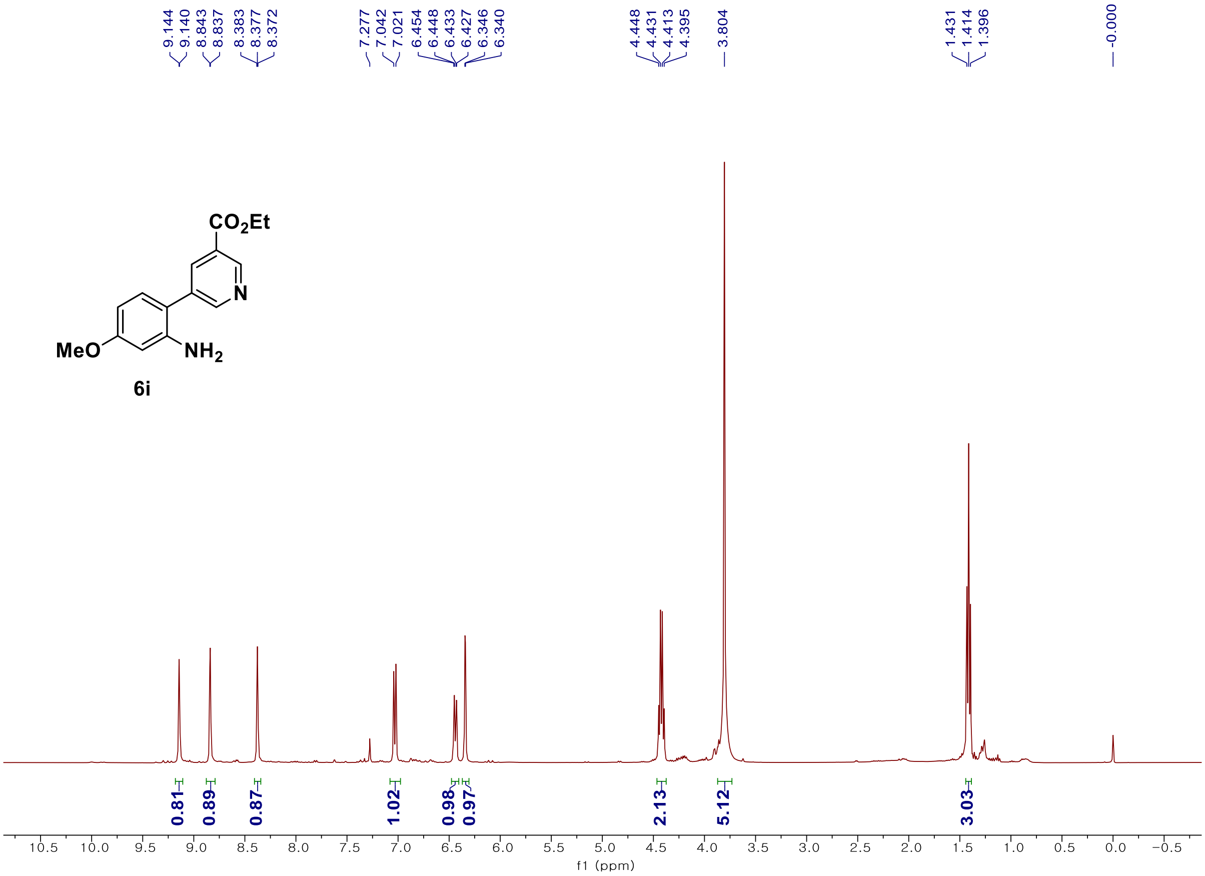
9.144
9.140
8.843
8.837
8.383
8.377
8.372
7.277
7.042
7.021
6.454
6.448
6.433
6.427
6.346
6.340
4.448
4.431
4.413
4.395
3.804
1.431
1.414
1.396
-0.000
CO2Et
N
MeO
NH2
6i
0.81
0.89
0.87
1.02
0.98
0.97
2.13
5.12
3.03
f1 (ppm)

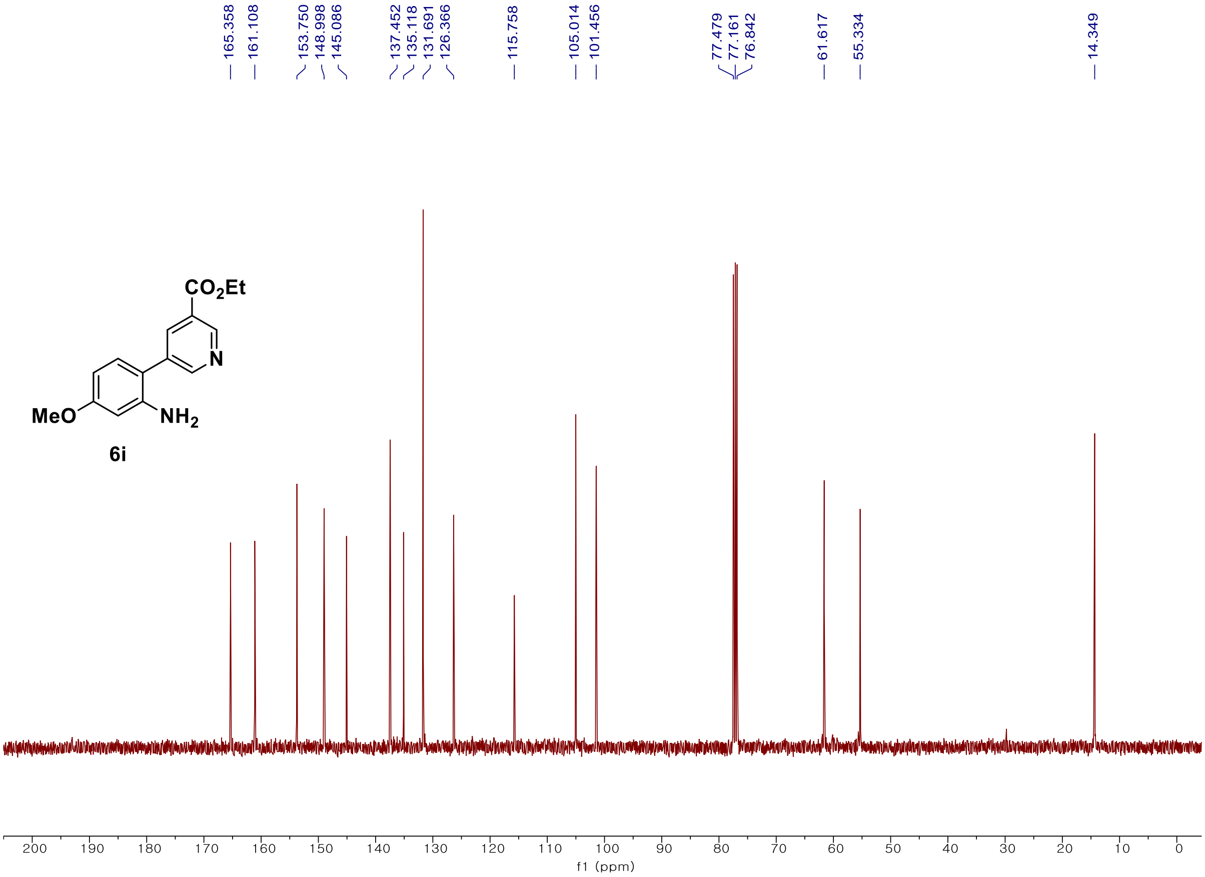
165.358
161.108
153.750
148.998
145.086
137.452
135.118
131.691
126.366
115.758
105.014
101.456
77.479
77.161
76.842
61.617
55.334
14.349
CO2Et
N
MeO
NH2
6i
f1 (ppm)

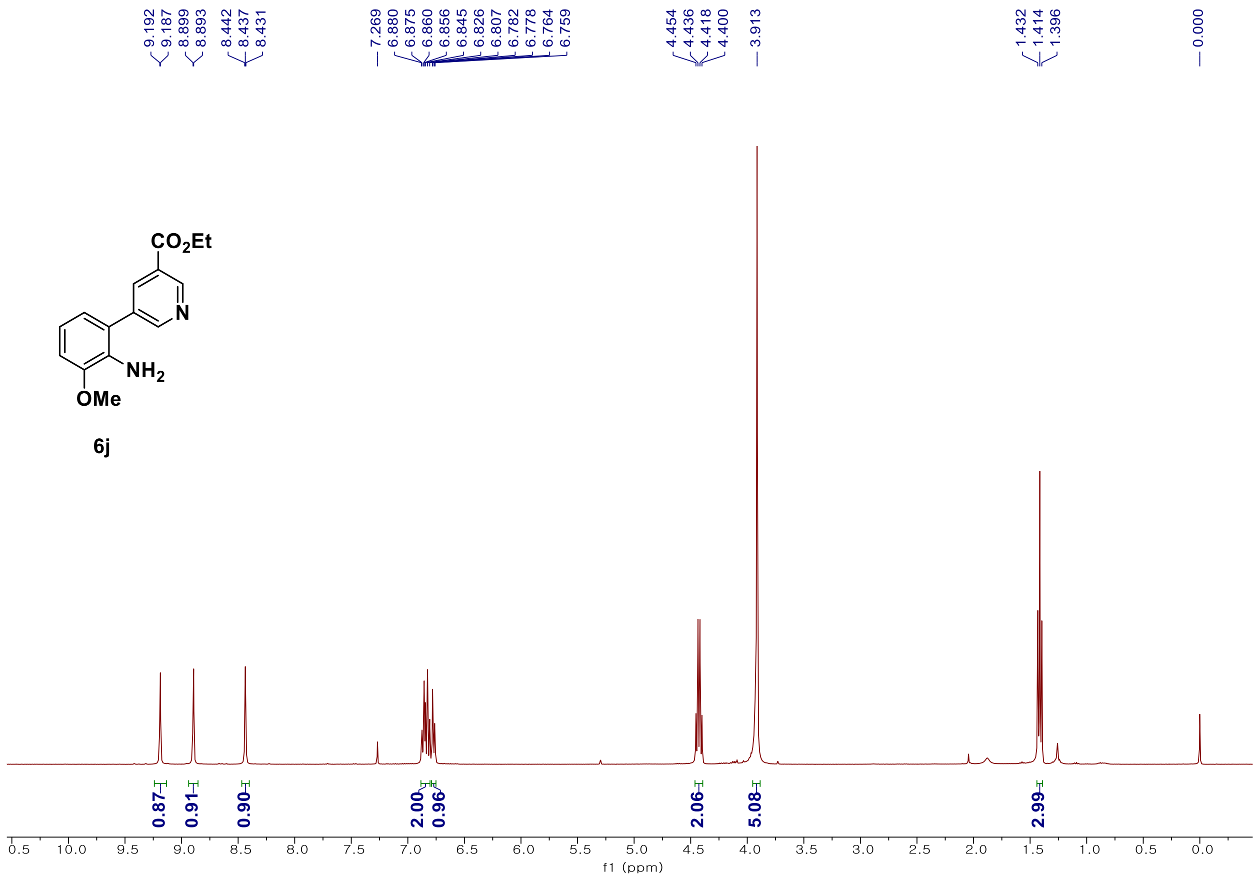
9.192
9.187
8.899
8.893
8.442
8.437
8.431
7.269
6.880
6.875
6.860
6.856
6.845
6.826
6.807
6.782
6.778
6.764
6.759
4.454
4.436
4.418
4.400
3.913
1.432
1.414
1.396
0.000
CO2Et
N
NH2
OMe
6j
0.87
0.91
0.90
2.00
0.96
2.06
5.08
2.99
f1 (ppm)

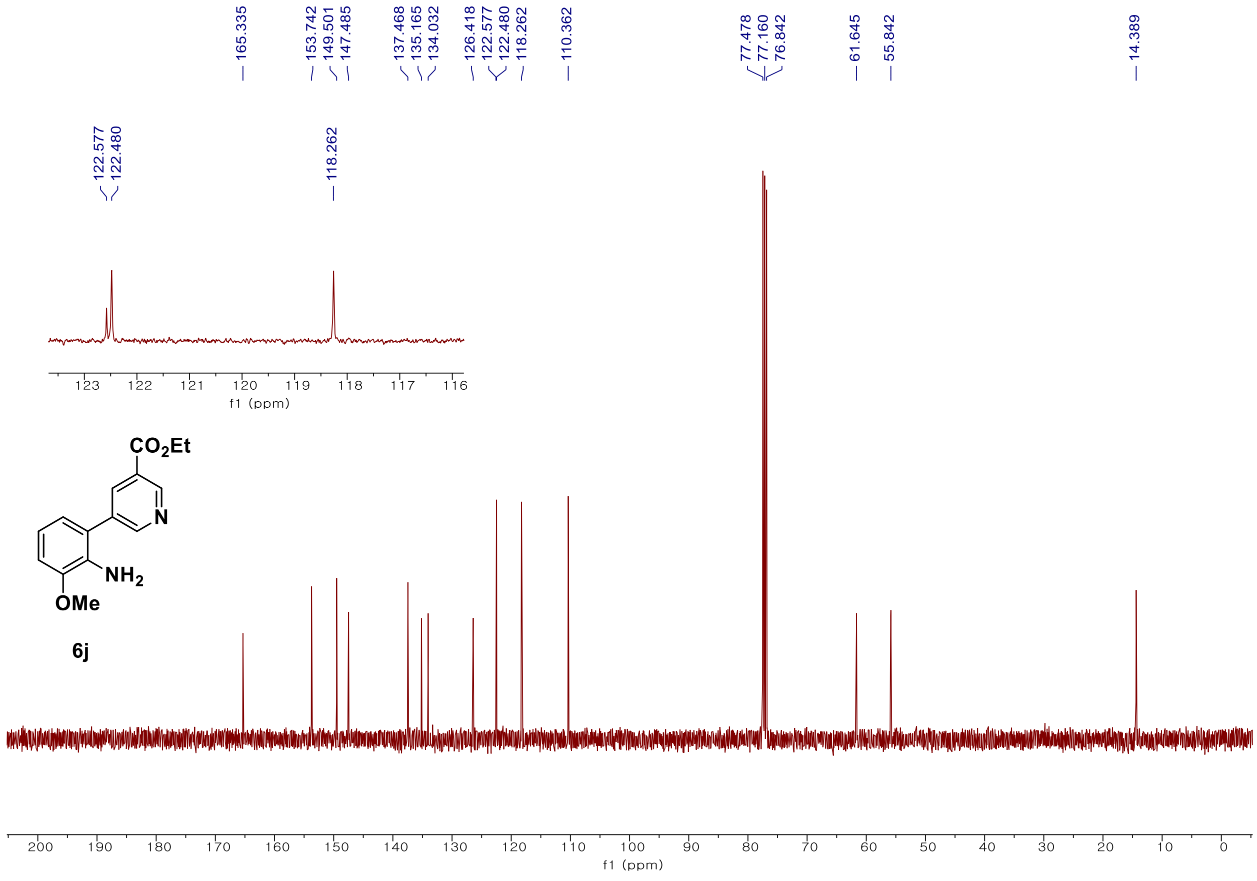
165.335
153.742
149.501
147.485
137.468
135.165
134.032
126.418
122.577
122.480
118.262
110.362
77.478
77.160
76.842
61.645
55.842
14.389
122.577
122.480
118.262
f1 (ppm)
CO2Et
N
NH2
OMe
6j
f1 (ppm)

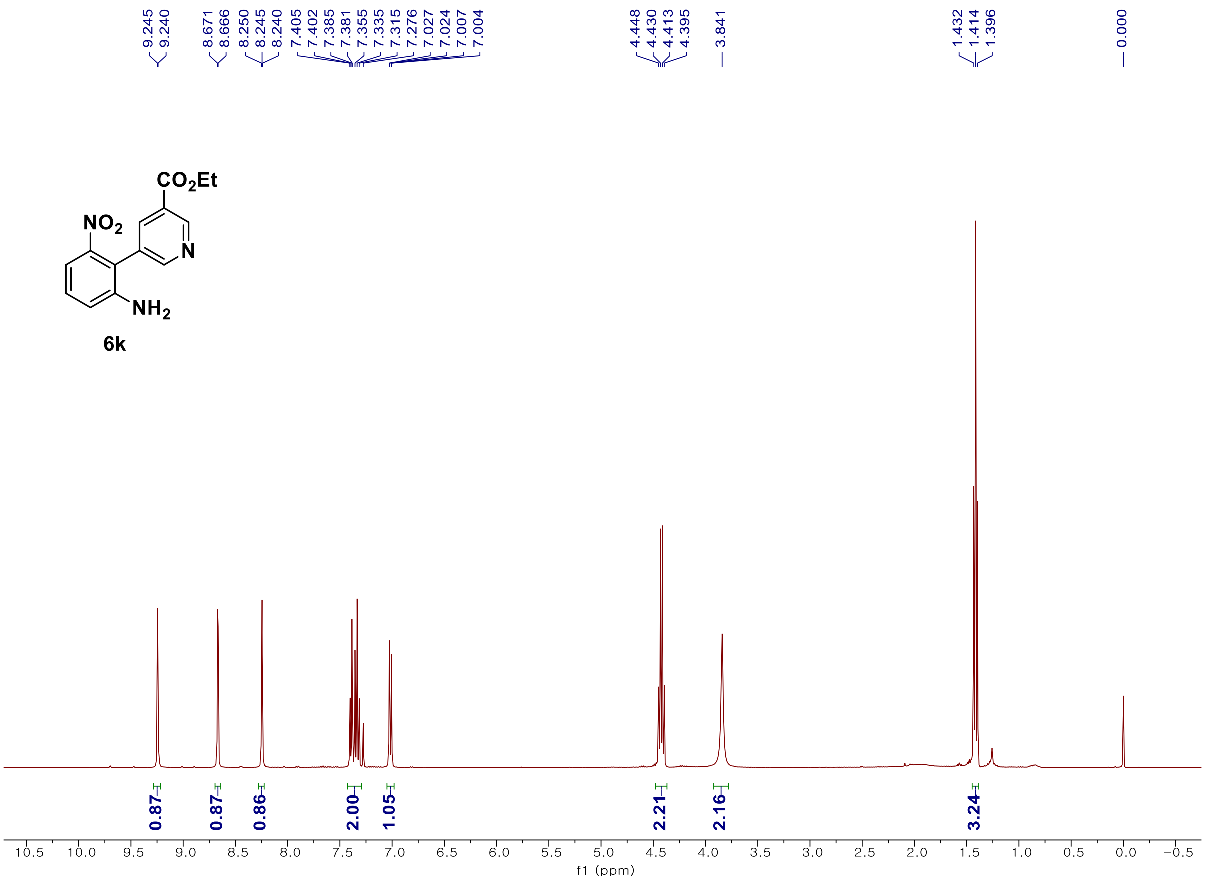
9.245
9.240
8.671
8.666
8.250
8.245
8.240
7.405
7.402
7.385
7.381
7.355
7.335
7.315
7.276
7.027
7.024
7.007
7.004
4.448
4.430
4.413
4.395
3.841
1.432
1.414
1.396
0.000
CO2Et
NO2
N
NH2
6k
0.87
0.87
0.86
2.00
1.05
2.21
2.16
3.24
10.5 10.0 9.5 9.0 8.5 8.0 7.5 7.0 6.5 6.0 5.5 5.0 4.5 4.0 3.5 3.0 2.5 2.0 1.5 1.0 0.5 0.0 −0.5
f1 (ppm)

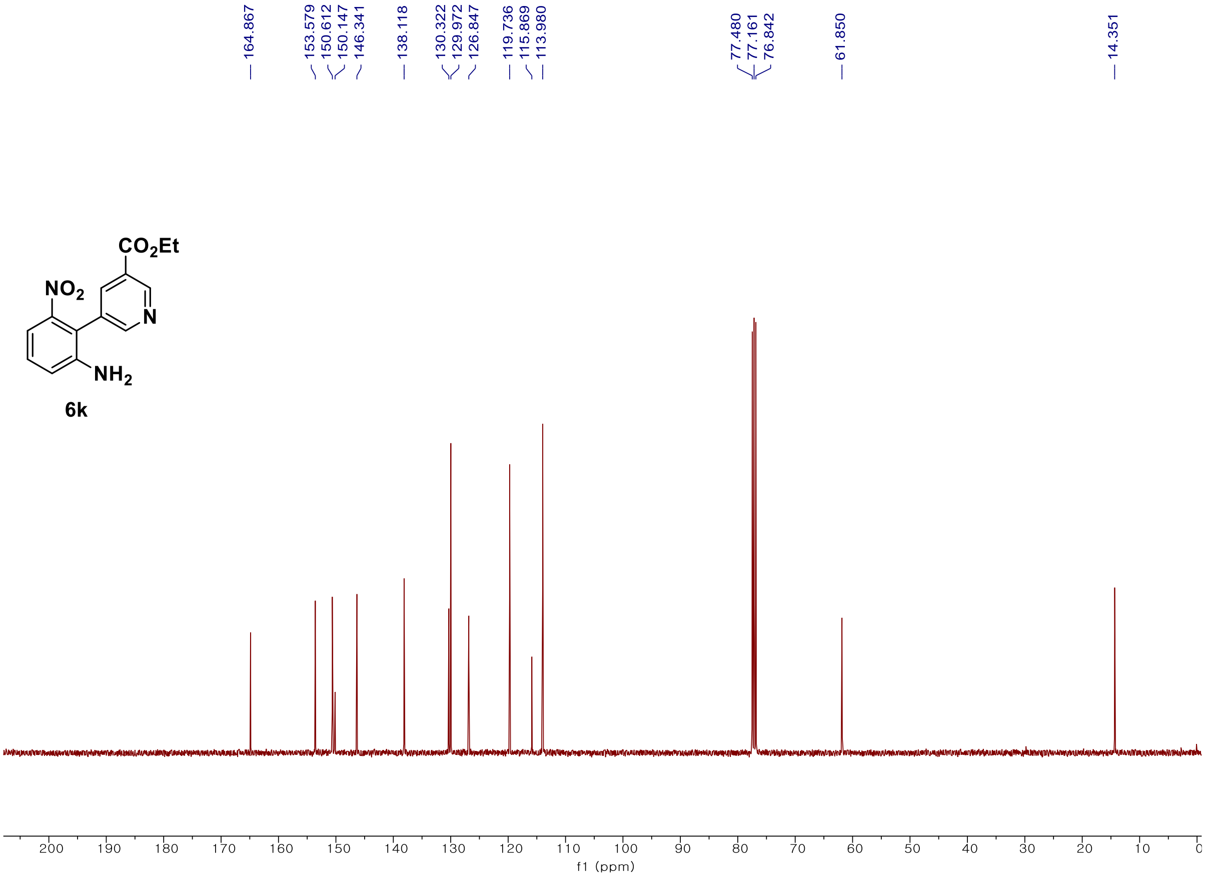
164.867
153.579
150.612
150.147
146.341
138.118
130.322
129.972
126.847
119.736
115.869
113.980
77.480
77.161
76.842
61.850
14.351
CO2Et
NO2
N
NH2
6k
200 190 180 170 160 150 140 130 120 110 100 90 80 70 60 50 40 30 20 10 0
f1 (ppm)

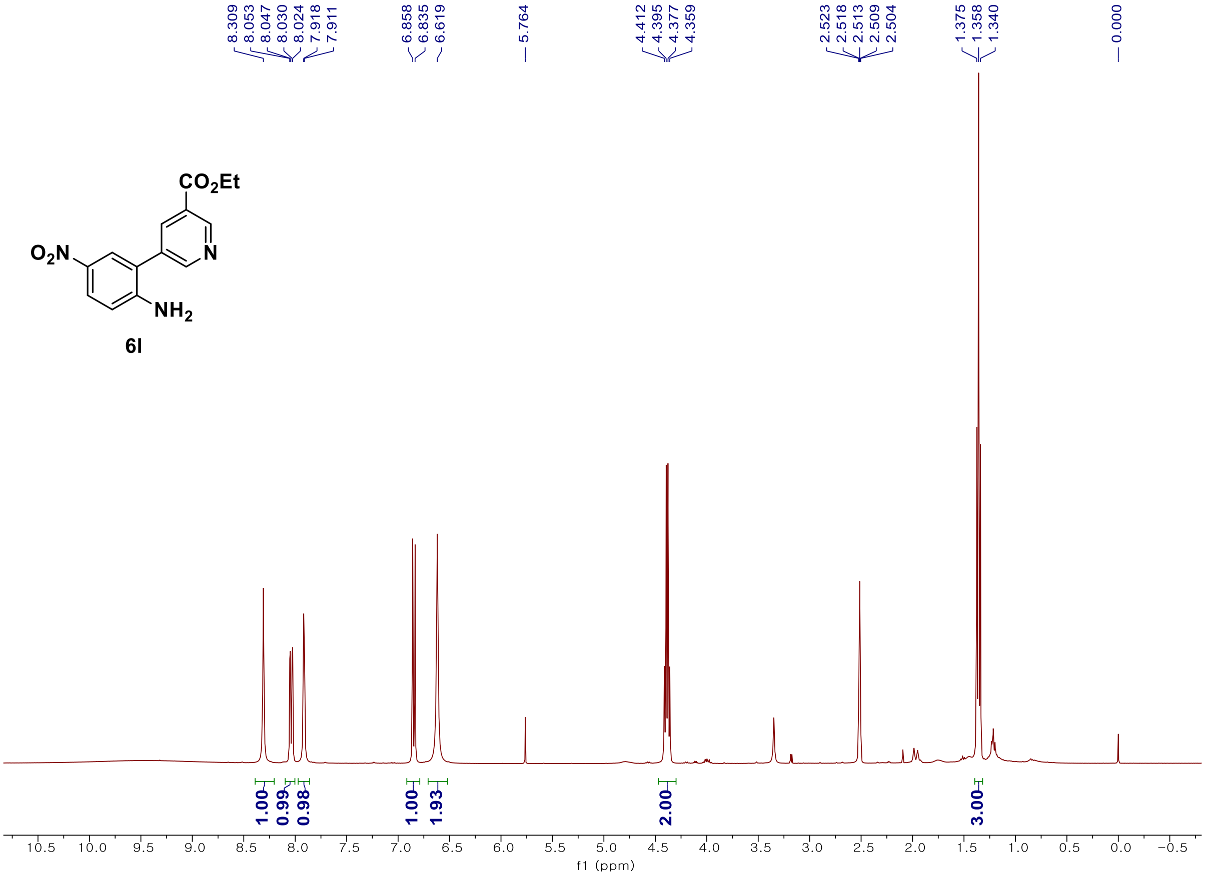
8.309
8.053
8.047
8.030
8.024
7.918
7.911
6.858
6.835
6.619
5.764
4.412
4.395
4.377
4.359
2.523
2.518
2.513
2.509
2.504
1.375
1.358
1.340
0.000
CO2Et
O2N
N
NH2
6l
1.00
0.99
0.98
1.00
1.93
2.00
3.00
f1 (ppm)

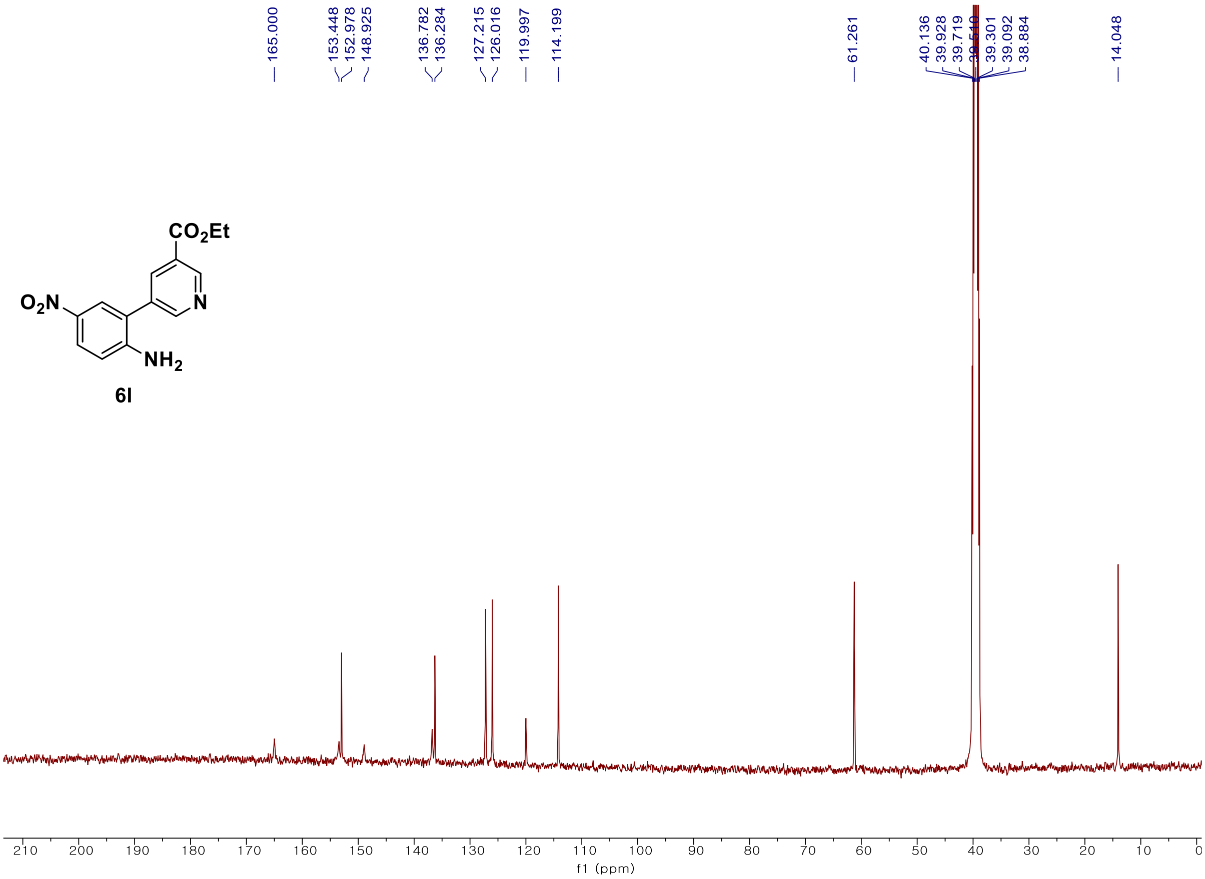
165.000
153.448
152.978
148.925
136.782
136.284
127.215
126.016
119.997
114.199
61.261
40.136
39.928
39.719
39.301
39.092
38.884
14.048
CO2Et
O2N
N
NH2
6l
f1 (ppm)

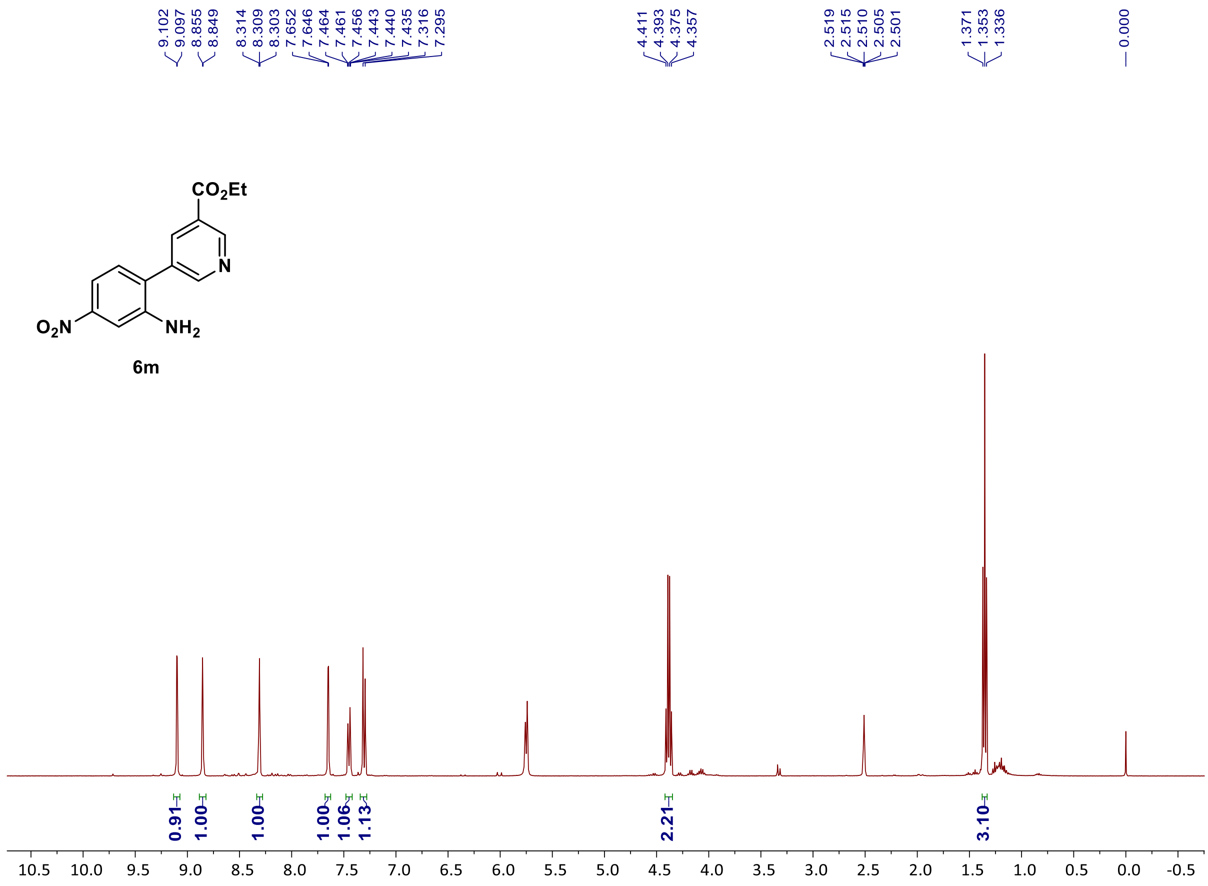
9.102
9.097
8.855
8.849
8.314
8.309
8.303
7.652
7.646
7.464
7.461
7.456
7.443
7.440
7.435
7.316
7.295
4.411
4.393
4.375
4.357
2.519
2.515
2.510
2.505
2.501
1.371
1.353
1.336
0.000
CO2Et
N
O2N
NH2
6m
0.91
1.00
1.00
1.00
1.06
1.13
2.21
3.10
10.5 10.0 9.5 9.0 8.5 8.0 7.5 7.0 6.5 6.0 5.5 5.0 4.5 4.0 3.5 3.0 2.5 2.0 1.5 1.0 0.5 0.0 -0.5

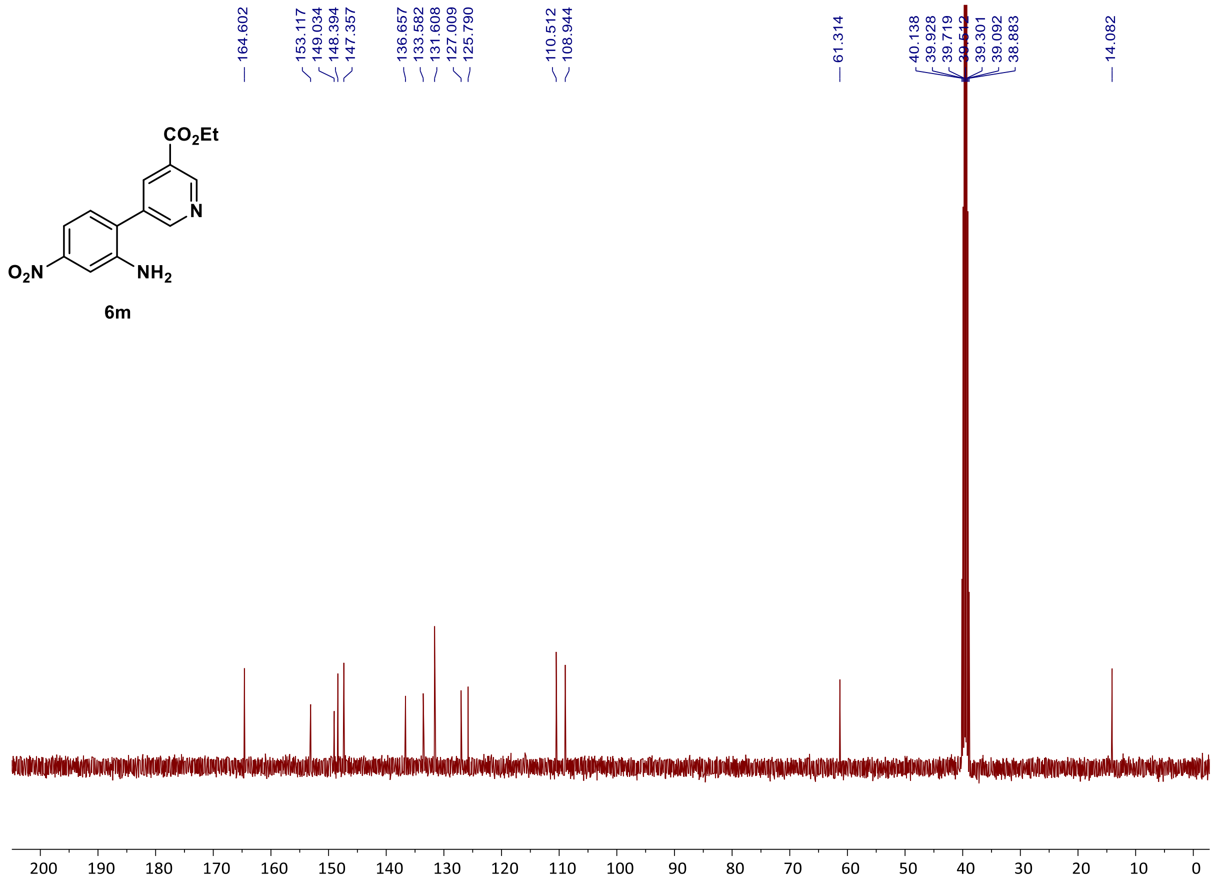
164.602
153.117
149.034
148.394
147.357
136.657
133.582
131.608
127.009
125.790
110.512
108.944
61.314
40.138
39.928
39.719
39.512
39.301
39.092
38.883
14.082
CO2Et
N
O2N
NH2
6m
200 190 180 170 160 150 140 130 120 110 100 90 80 70 60 50 40 30 20 10 0

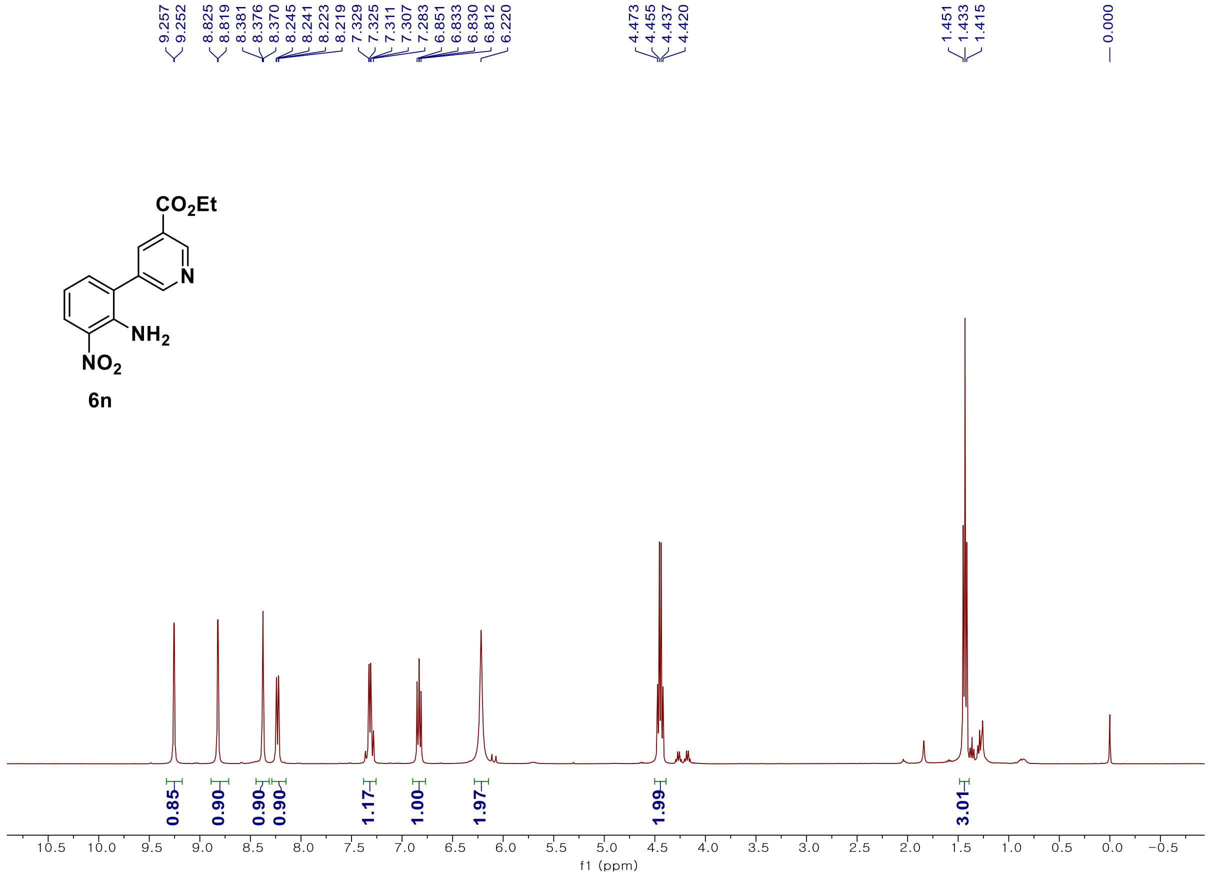
9.257
9.252
8.825
8.819
8.381
8.376
8.370
8.245
8.241
8.223
8.219
7.329
7.325
7.311
7.307
7.283
6.851
6.833
6.830
6.812
6.220
4.473
4.455
4.437
4.420
1.451
1.433
1.415
0.000
CO2Et
N
NH2
NO2
6n
0.85
0.90
0.90
0.90
1.17
1.00
1.97
1.99
3.01
f1 (ppm)

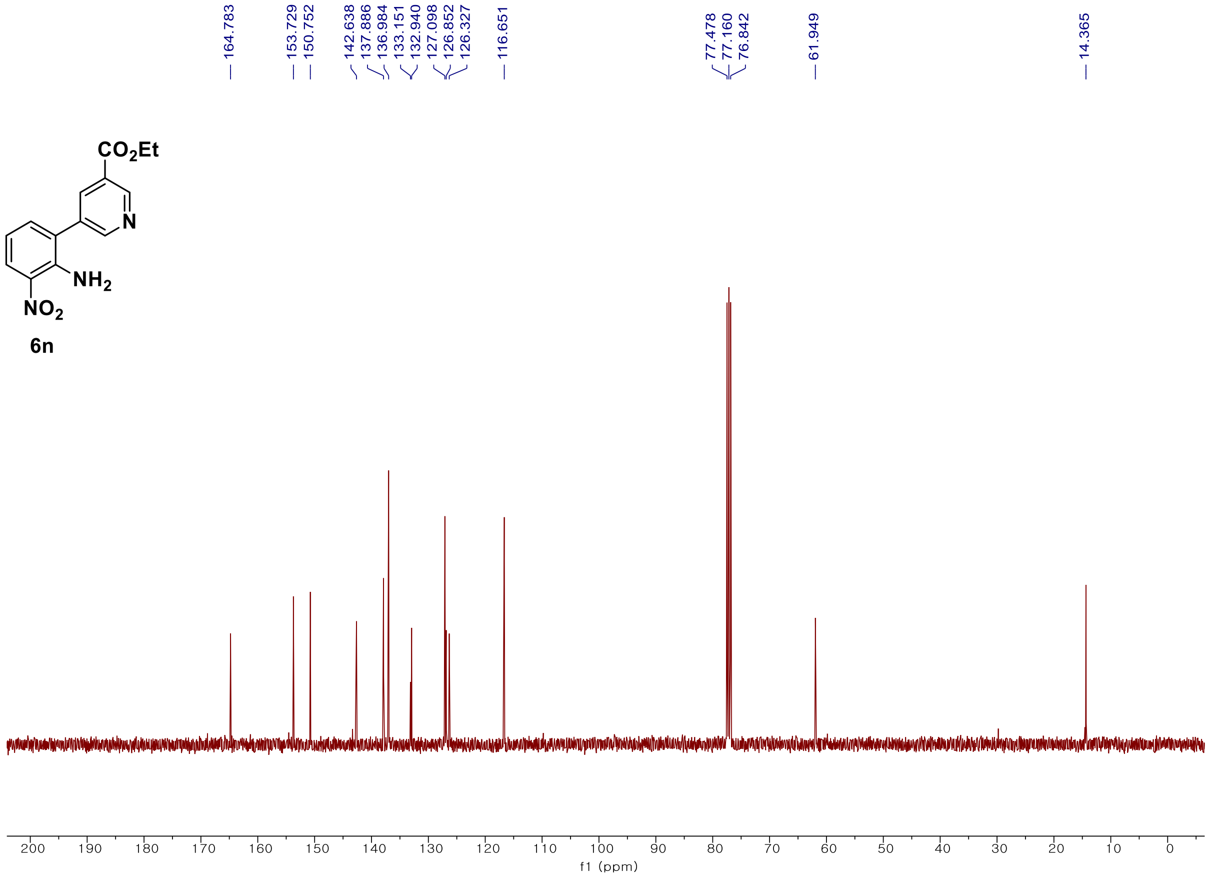
164.783
153.729
150.752
142.638
137.886
136.984
133.151
132.940
127.098
126.852
126.327
116.651
77.478
77.160
76.842
61.949
14.365
CO2Et
N
NH2
NO2
6n
f1 (ppm)

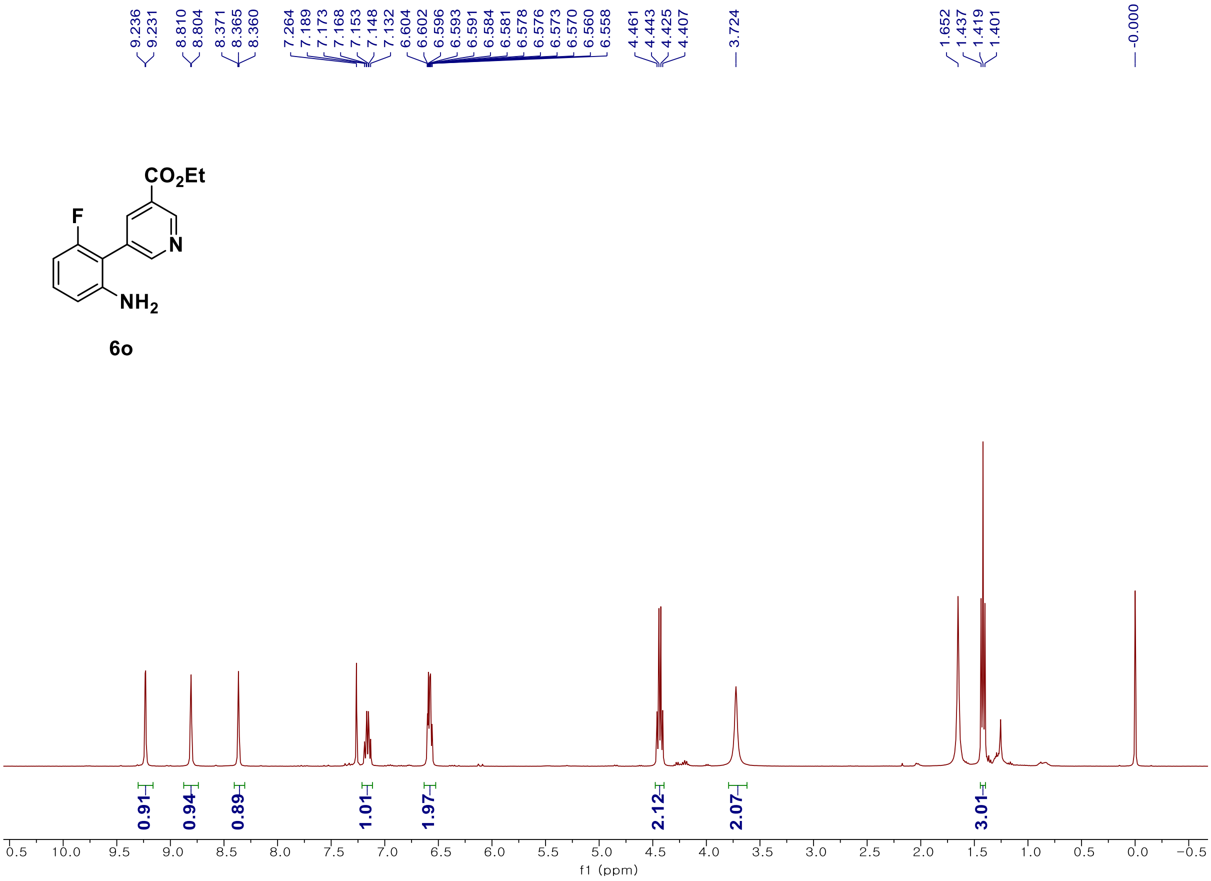
9.236
9.231
8.810
8.804
8.371
8.365
8.360
7.264
7.189
7.173
7.168
7.153
7.148
7.132
6.604
6.602
6.596
6.593
6.591
6.584
6.581
6.578
6.576
6.573
6.570
6.560
6.558
4.461
4.443
4.425
4.407
3.724
1.652
1.437
1.419
1.401
-0.000
CO2Et
F
N
NH2
6o
0.91
0.94
0.89
1.01
1.97
2.12
2.07
3.01
f1 (ppm)

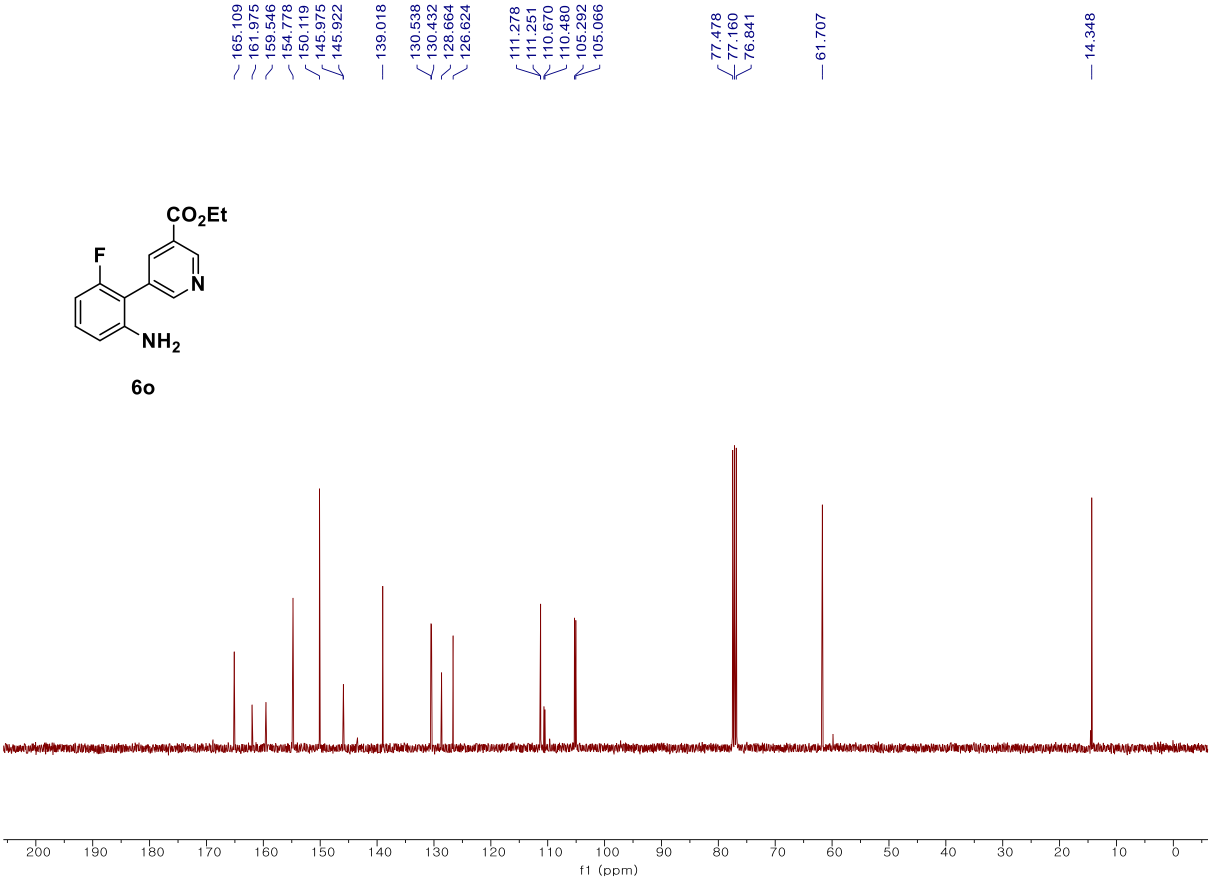
165.109
161.975
159.546
154.778
150.119
145.975
145.922
139.018
130.538
130.432
128.664
126.624
111.278
111.251
110.670
110.480
105.292
105.066
77.478
77.160
76.841
61.707
14.348
CO2Et
F
N
NH2
6o
f1 (ppm)

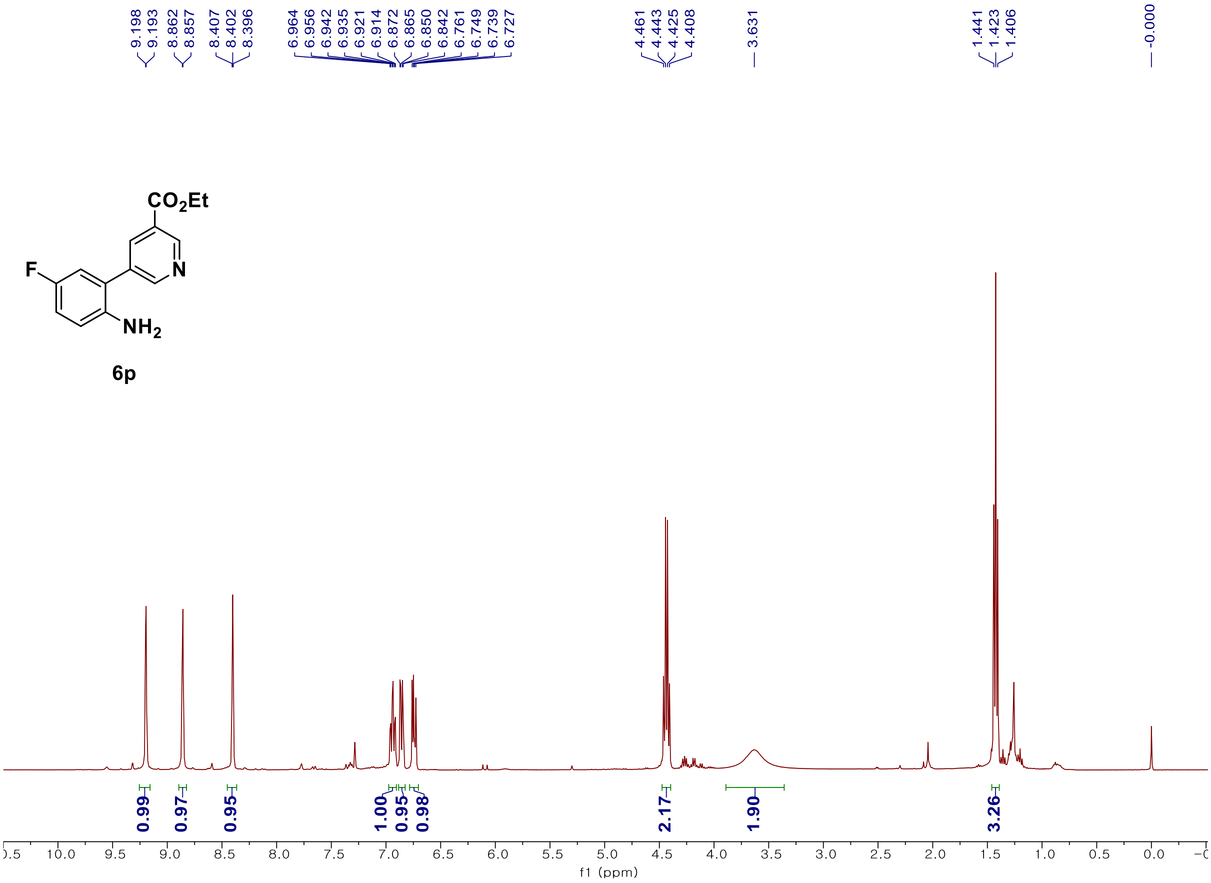
9.198
9.193
8.862
8.857
8.407
8.402
8.396
6.964
6.956
6.942
6.935
6.921
6.914
6.872
6.865
6.850
6.842
6.761
6.749
6.739
6.727
4.461
4.443
4.425
4.408
3.631
1.441
1.423
1.406
-0.000
CO2Et
F
N
NH2
6p
0.99
0.97
0.95
1.00
0.95
0.98
2.17
1.90
3.26
f1 (ppm)

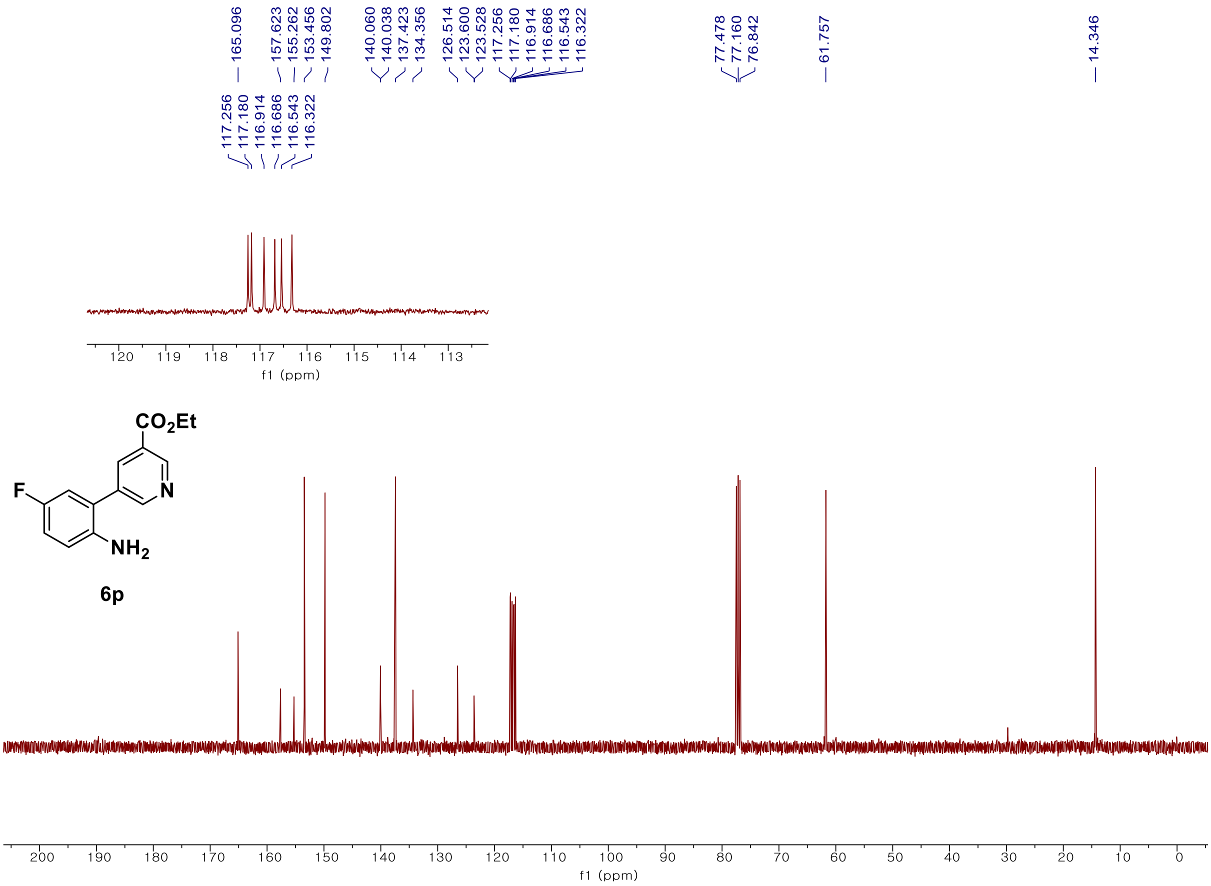
165.096
157.623
155.262
153.456
149.802
140.060
140.038
137.423
134.356
126.514
123.600
123.528
117.256
117.180
116.914
116.686
116.543
116.322
77.478
77.160
76.842
61.757
14.346
f1 (ppm)
CO2Et
F
N
NH2
6p

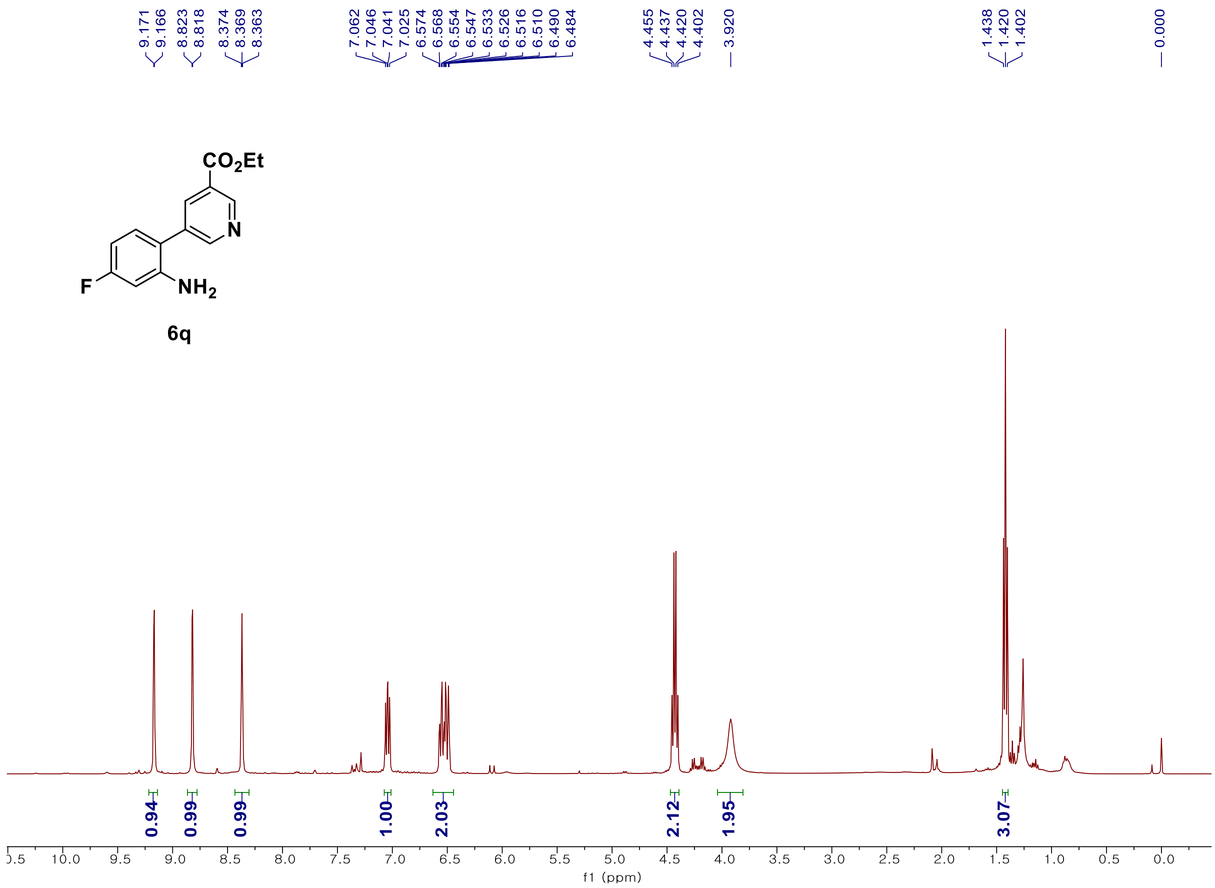

6q
f1 (ppm)

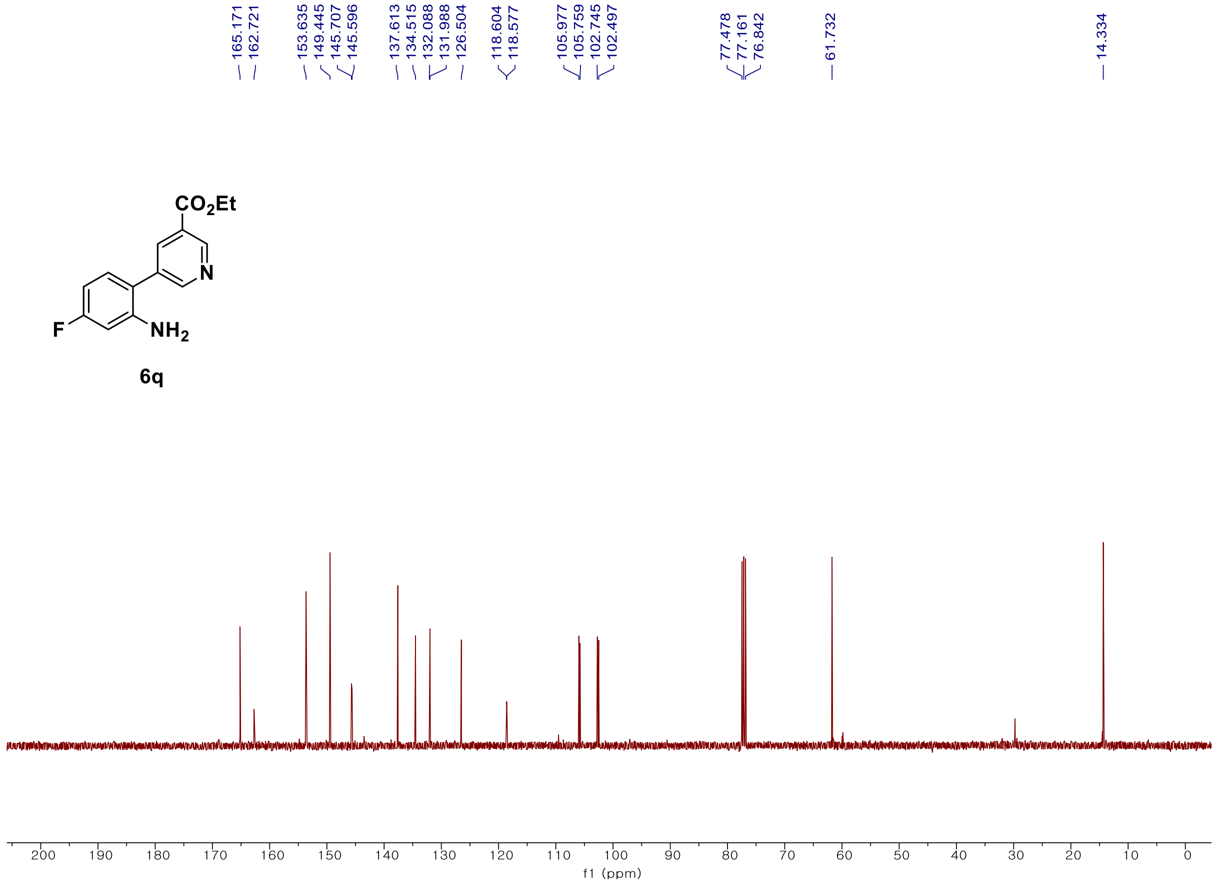

6q
f1 (ppm)

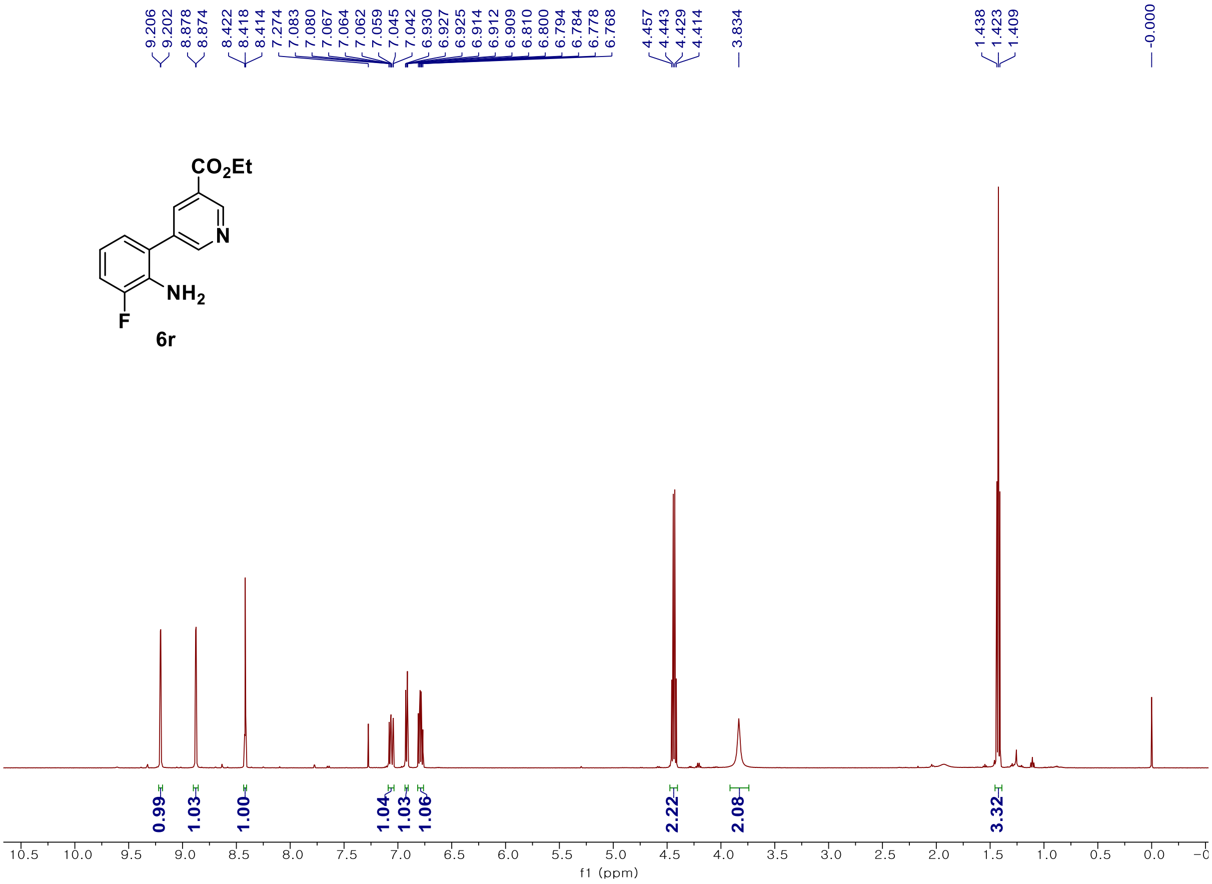
9.206
9.202
8.878
8.874
8.422
8.418
8.414
7.274
7.083
7.080
7.067
7.064
7.062
7.059
7.045
7.042
6.930
6.927
6.925
6.914
6.912
6.909
6.810
6.800
6.794
6.784
6.778
6.768
4.457
4.443
4.429
4.414
3.834
1.438
1.423
1.409
-0.000
CO2Et
N
NH2
F
6r
0.99
1.03
1.00
1.04
1.03
1.06
2.22
2.08
3.32
10.5 10.0 9.5 9.0 8.5 8.0 7.5 7.0 6.5 6.0 5.5 5.0 4.5 4.0 3.5 3.0 2.5 2.0 1.5 1.0 0.5 0.0
f1 (ppm)

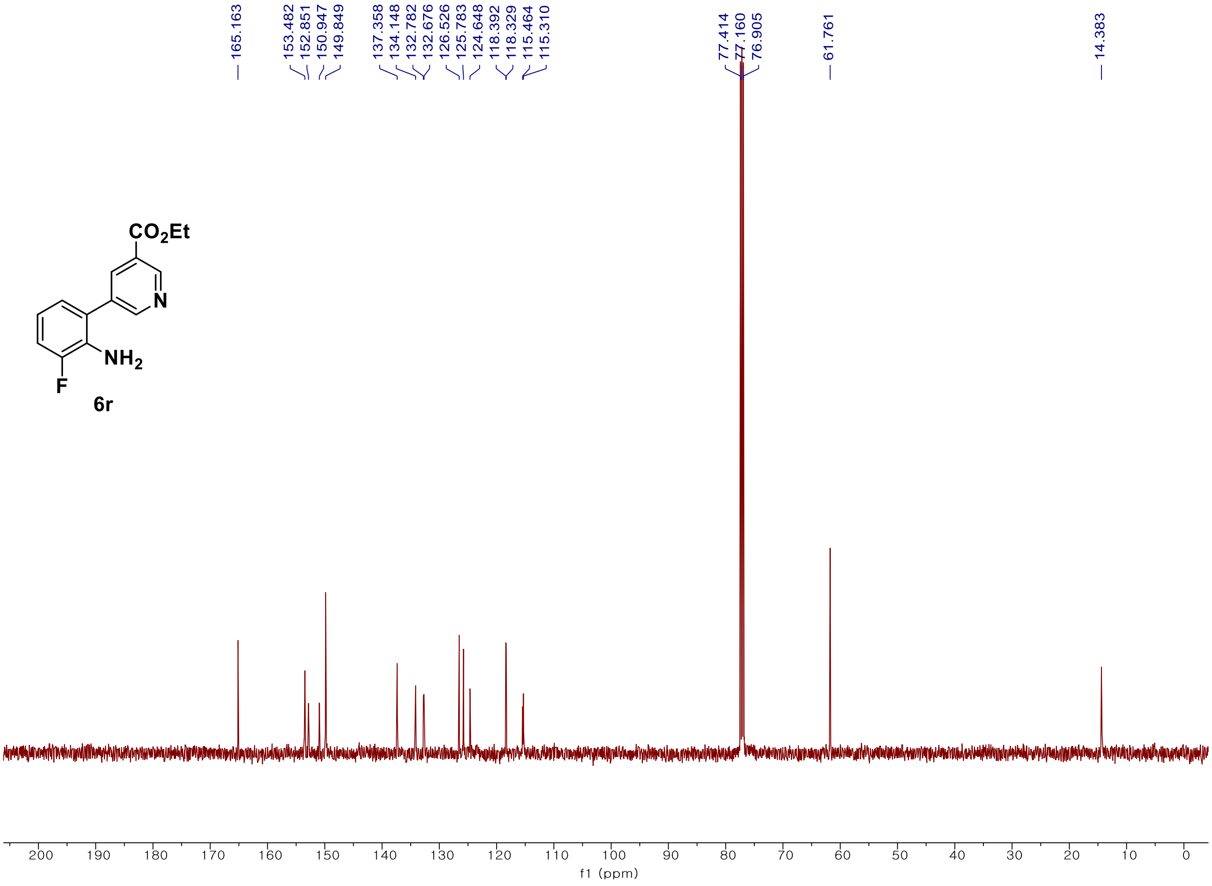
165.163
153.482
152.851
150.947
149.849
137.358
134.148
132.782
132.676
126.526
125.783
124.648
118.392
118.329
115.464
115.310
77.414
77.160
76.905
61.761
14.383
CO2Et
N
NH2
F
6r
200 190 180 170 160 150 140 130 120 110 100 90 80 70 60 50 40 30 20 10 0
f1 (ppm)

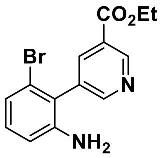

CO2Et
Br
N
NH2

6s

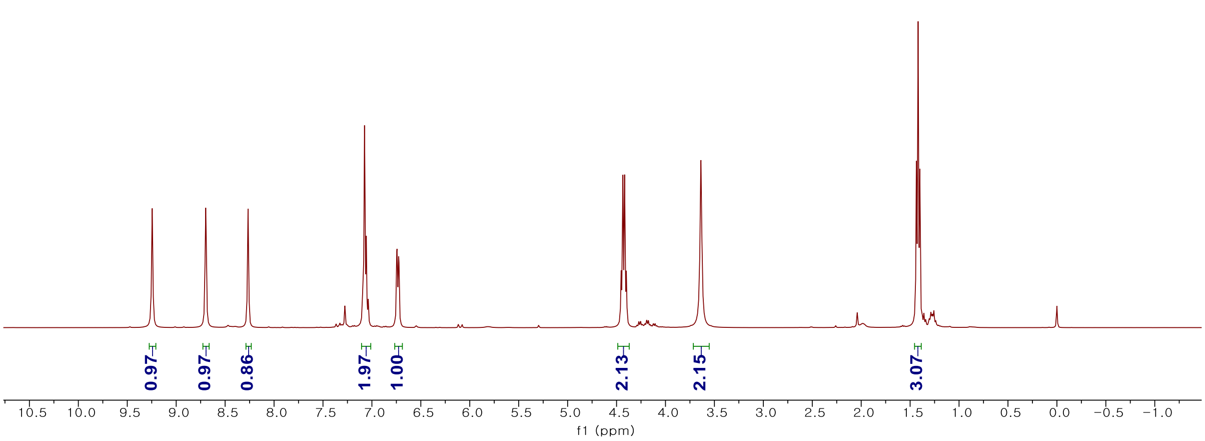

9.247
9.241
8.701
8.694
8.271
8.266
8.260
7.276
7.094
7.075
7.057
7.037
6.746
6.741
6.729
6.724
4.453
4.436
4.418
4.400
3.637
1.436
1.418
1.400
-0.000
0.97
0.97
0.86
1.97
1.00
2.13
2.15
3.07
10.5 10.0 9.5 9.0 8.5 8.0 7.5 7.0 6.5 6.0 5.5 5.0 4.5 4.0 3.5 3.0 2.5 2.0 1.5 1.0 0.5 0.0 −0.5 −1.0
f1 (ppm)

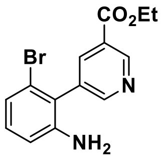

CO2Et
Br
N
NH2

6s

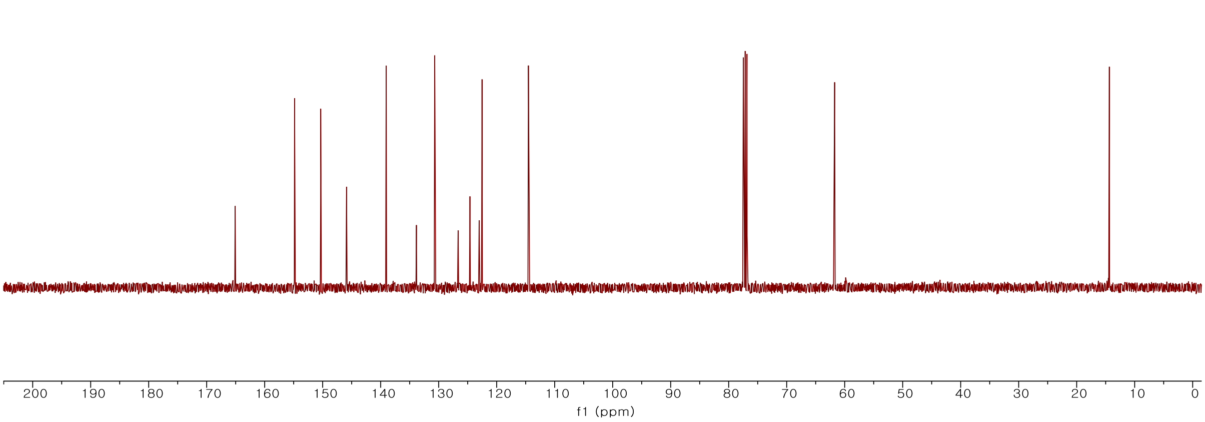

165.096
154.850
150.331
145.884
139.054
133.840
130.686
126.652
124.610
123.023
122.526
114.528
77.479
77.160
76.842
61.735
14.372
200 190 180 170 160 150 140 130 120 110 100 90 80 70 60 50 40 30 20 10 0
f1 (ppm)

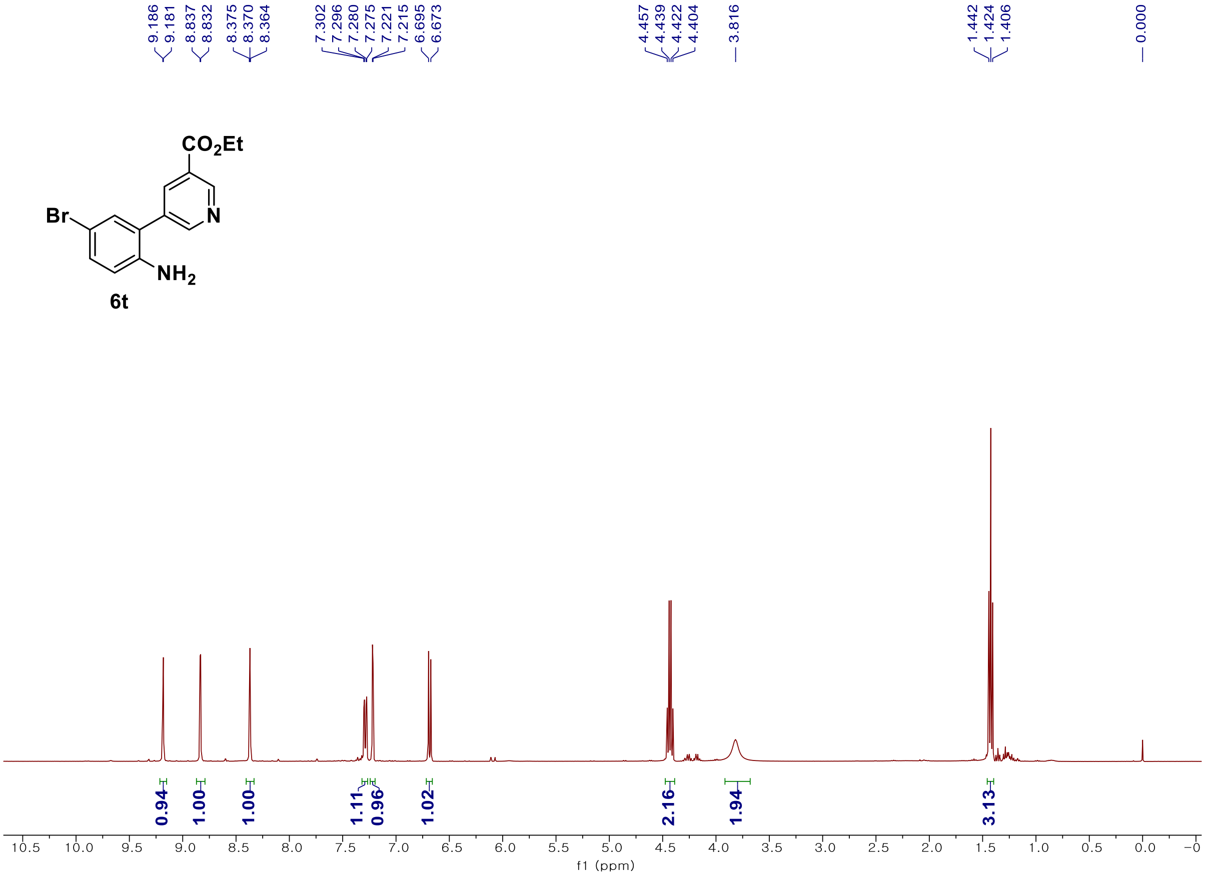
9.186
9.181
8.837
8.832
8.375
8.370
8.364
7.302
7.296
7.280
7.275
7.221
7.215
6.695
6.673
4.457
4.439
4.422
4.404
3.816
1.442
1.424
1.406
0.000
CO2Et
Br
N
NH2
6t
0.94
1.00
1.00
1.11
0.96
1.02
2.16
1.94
3.13
f1 (ppm)

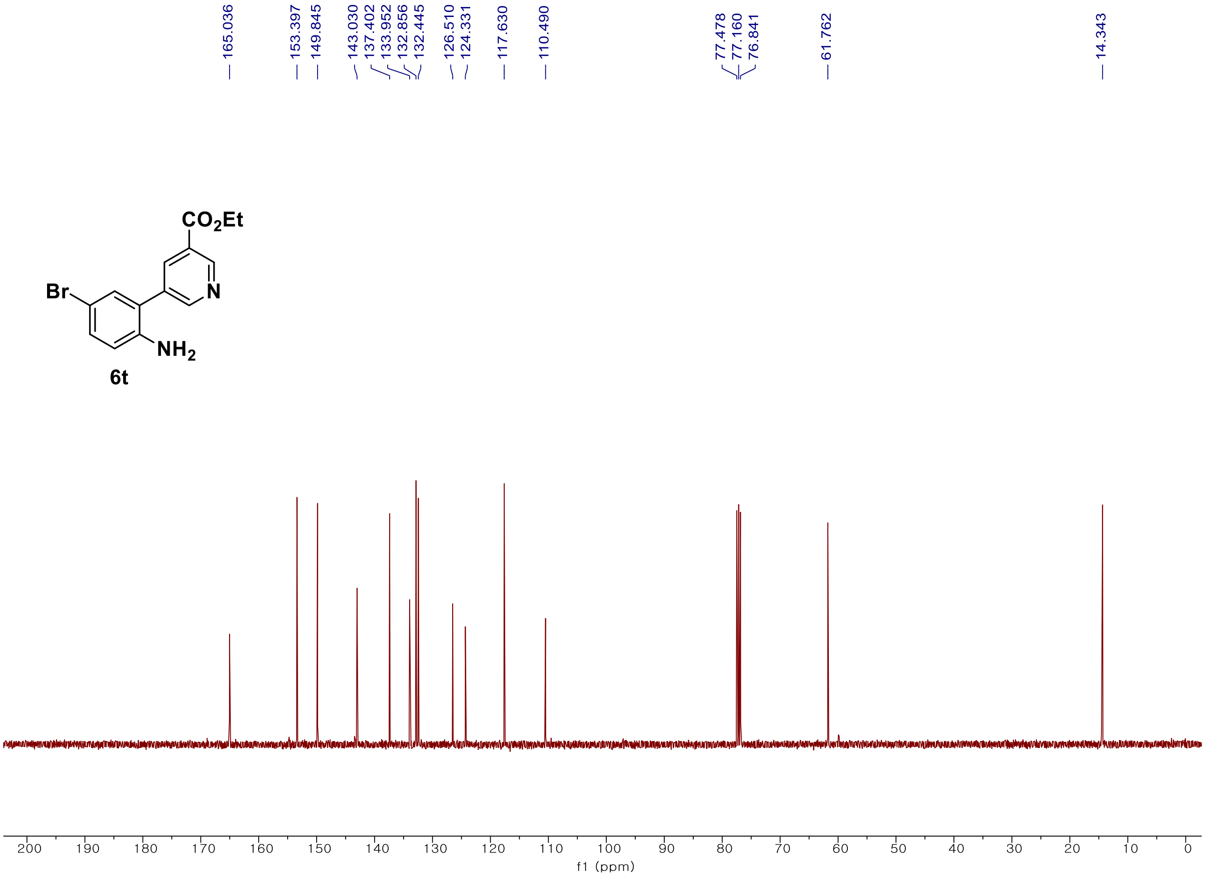
165.036
153.397
149.845
143.030
137.402
133.952
132.856
132.445
126.510
124.331
117.630
110.490
77.478
77.160
76.841
61.762
14.343
CO2Et
Br
N
NH2
6t
f1 (ppm)

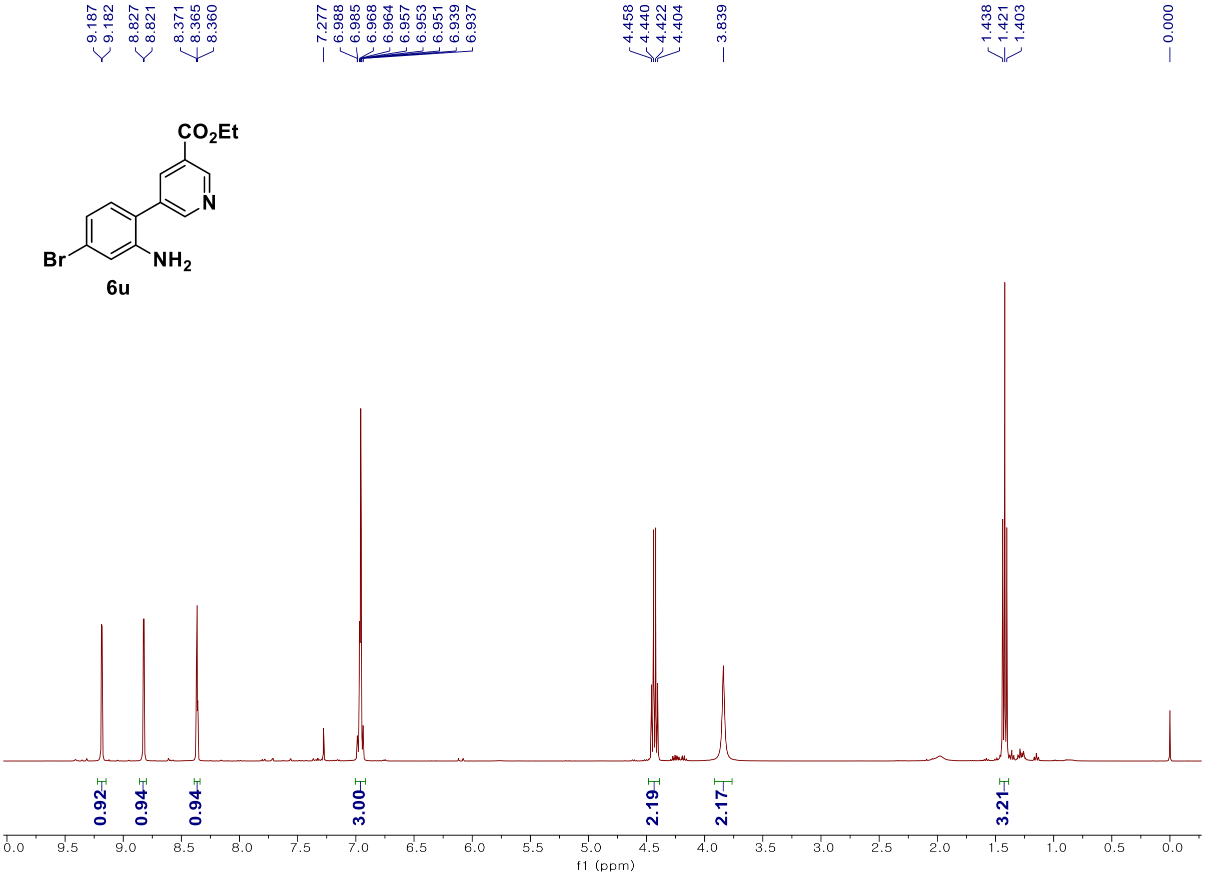
9.187
9.182
8.827
8.821
8.371
8.365
8.360
7.277
6.988
6.985
6.968
6.964
6.957
6.953
6.951
6.939
6.937
4.458
4.440
4.422
4.404
3.839
1.438
1.421
1.403
0.000
CO2Et
N
Br
NH2
6u
0.92
0.94
0.94
3.00
2.19
2.17
3.21
f1 (ppm)

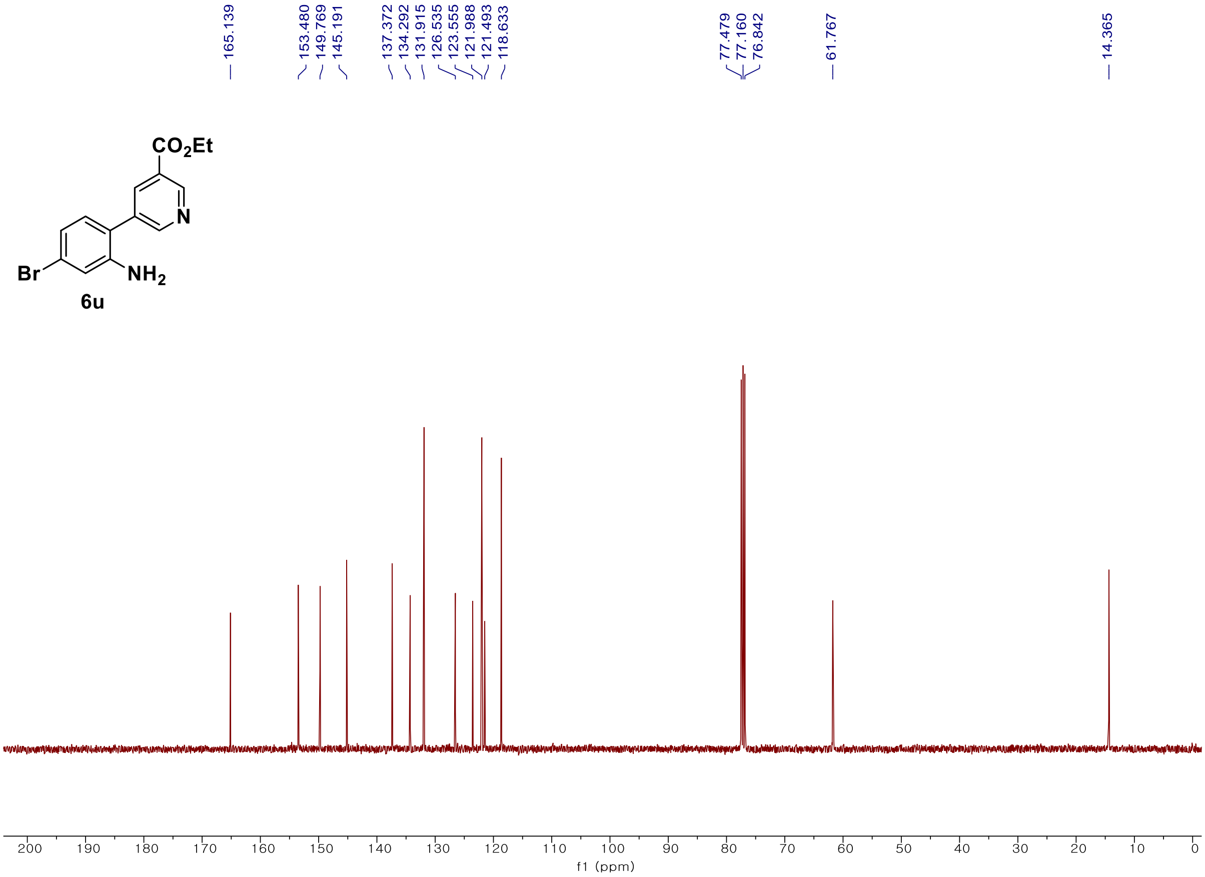
165.139
153.480
149.769
145.191
137.372
134.292
131.915
126.535
123.555
121.988
121.493
118.633
77.479
77.160
76.842
61.767
14.365
CO2Et
N
Br
NH2
6u
f1 (ppm)

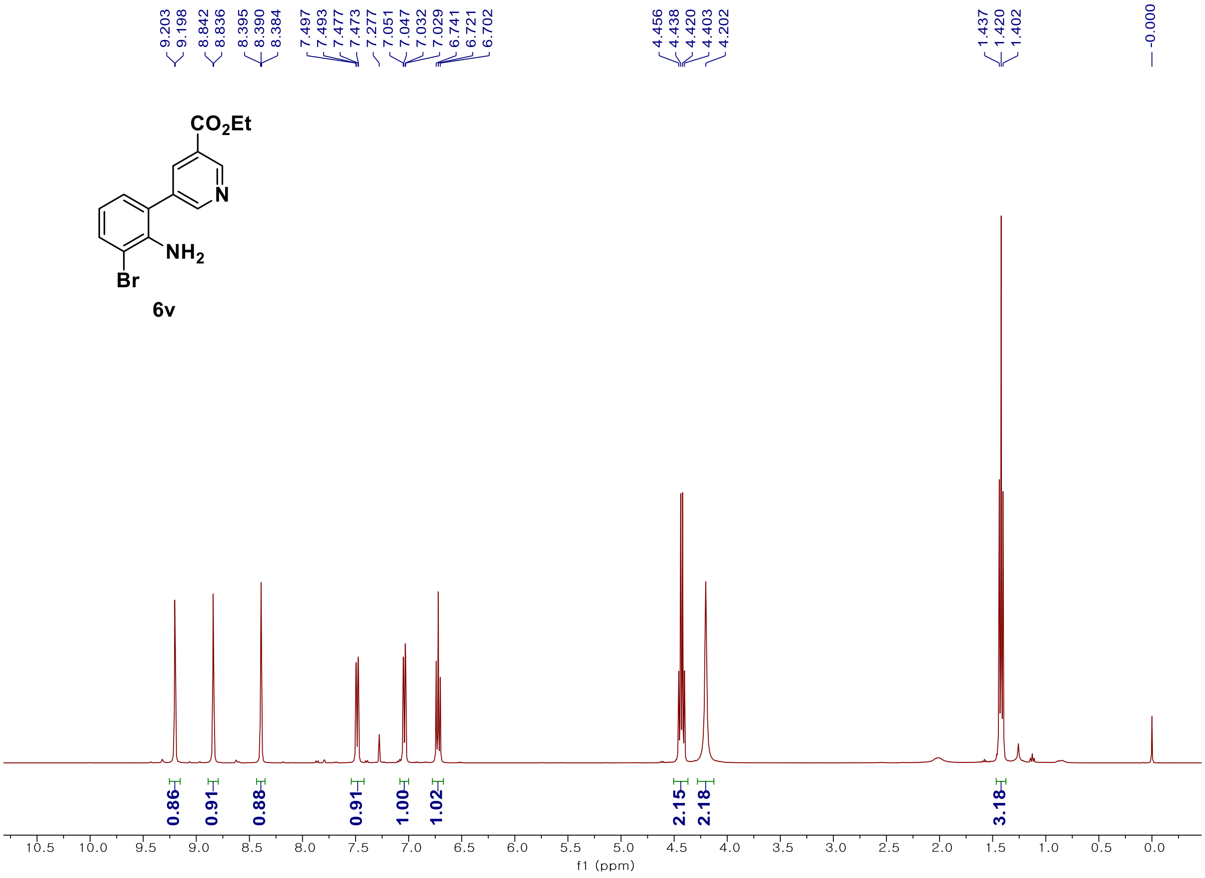
9.203
9.198
8.842
8.836
8.395
8.390
8.384
7.497
7.493
7.477
7.473
7.277
7.051
7.047
7.032
7.029
6.741
6.721
6.702
4.456
4.438
4.420
4.403
4.202
1.437
1.420
1.402
-0.000
CO2Et
N
NH2
Br
6v
0.86
0.91
0.88
0.91
1.00
1.02
2.15
2.18
3.18
f1 (ppm)

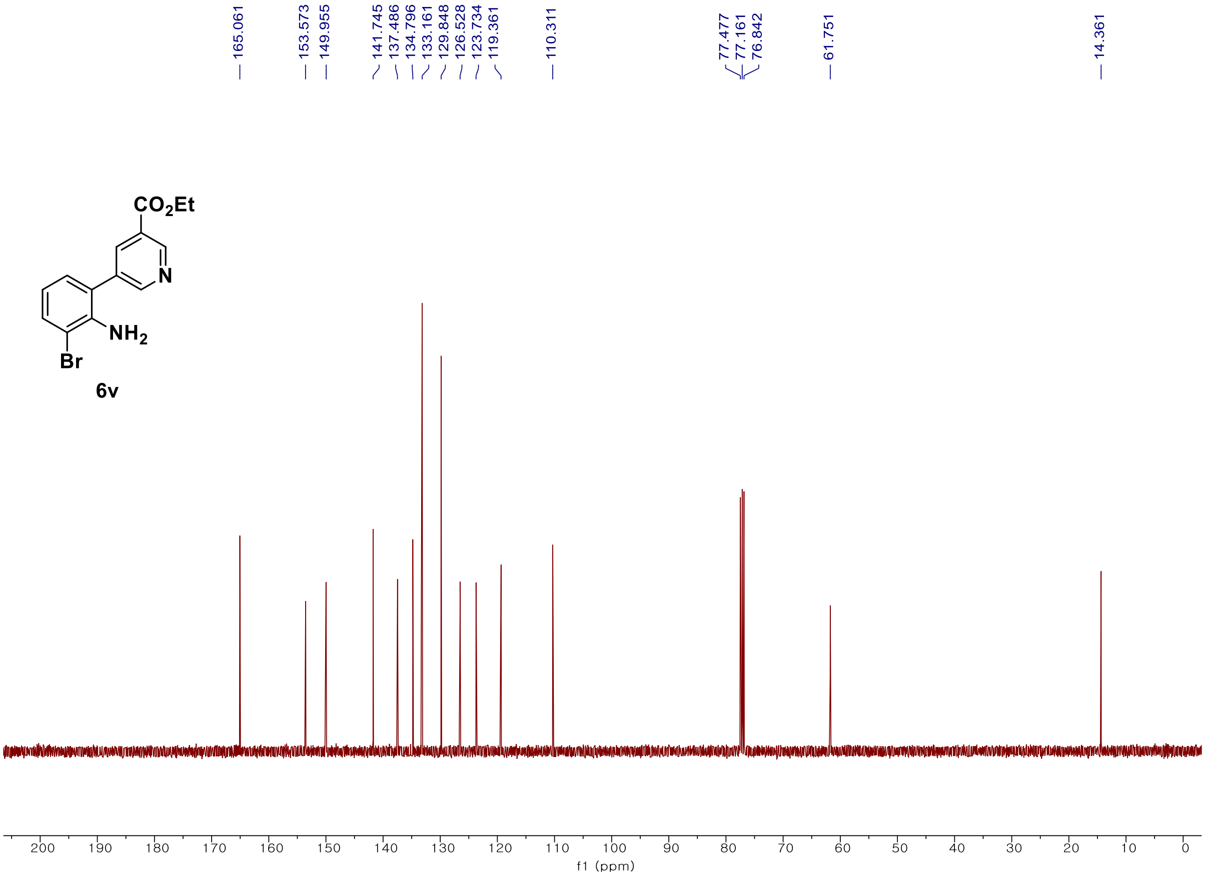
165.061
153.573
149.955
141.745
137.486
134.796
133.161
129.848
126.528
123.734
119.361
110.311
77.477
77.161
76.842
61.751
14.361
CO2Et
N
NH2
Br
6v
f1 (ppm)

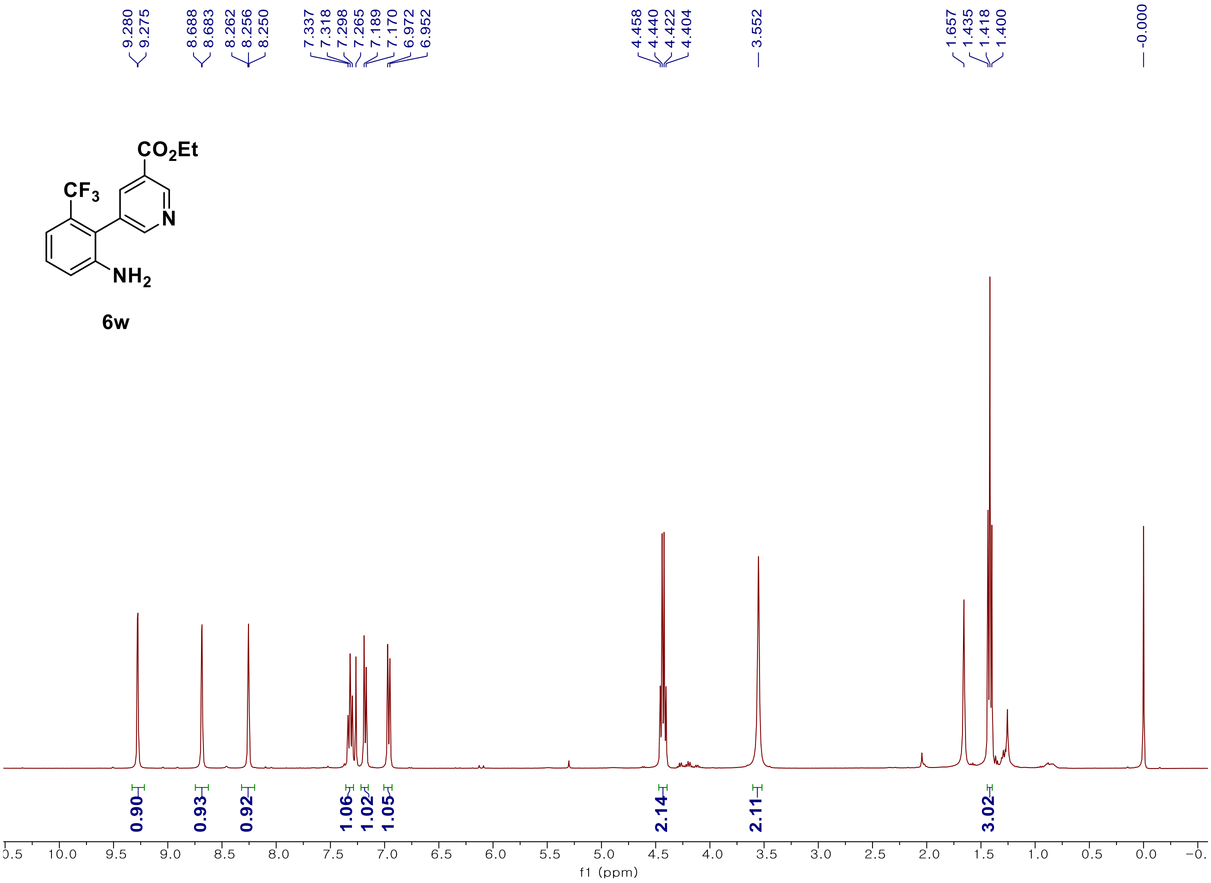
9.280
9.275
8.688
8.683
8.262
8.256
8.250
7.337
7.318
7.298
7.265
7.189
7.170
6.972
6.952
4.458
4.440
4.422
4.404
3.552
1.657
1.435
1.418
1.400
-0.000
CO2Et
CF3
N
NH2
6w
0.90
0.93
0.92
1.06
1.02
1.05
2.14
2.11
3.02
f1 (ppm)

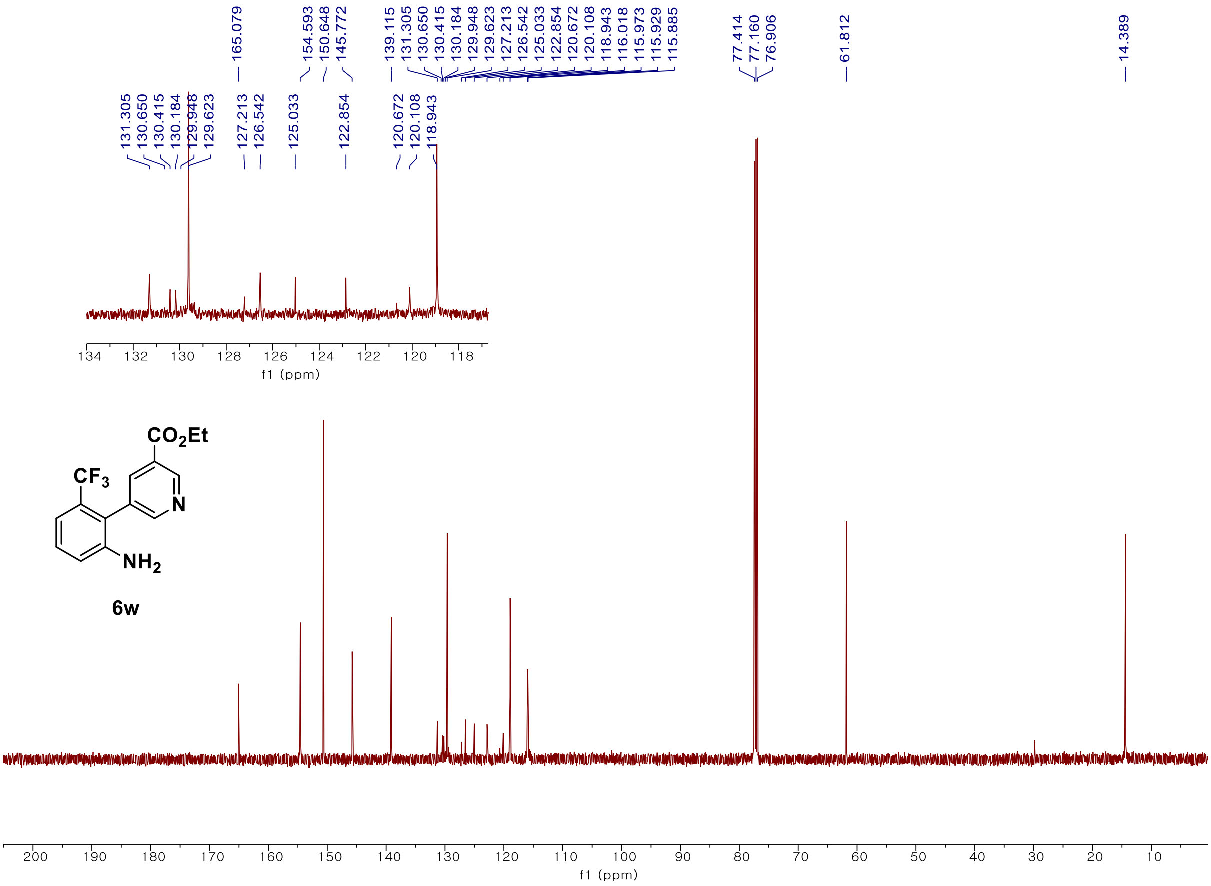
165.079
154.593
150.648
145.772
139.115
131.305
130.650
130.415
130.184
129.948
129.623
127.213
126.542
125.033
122.854
120.672
120.108
118.943
116.018
115.973
115.929
115.885
77.414
77.160
76.906
61.812
14.389
f1 (ppm)
CO2Et
CF3
N
NH2
6w

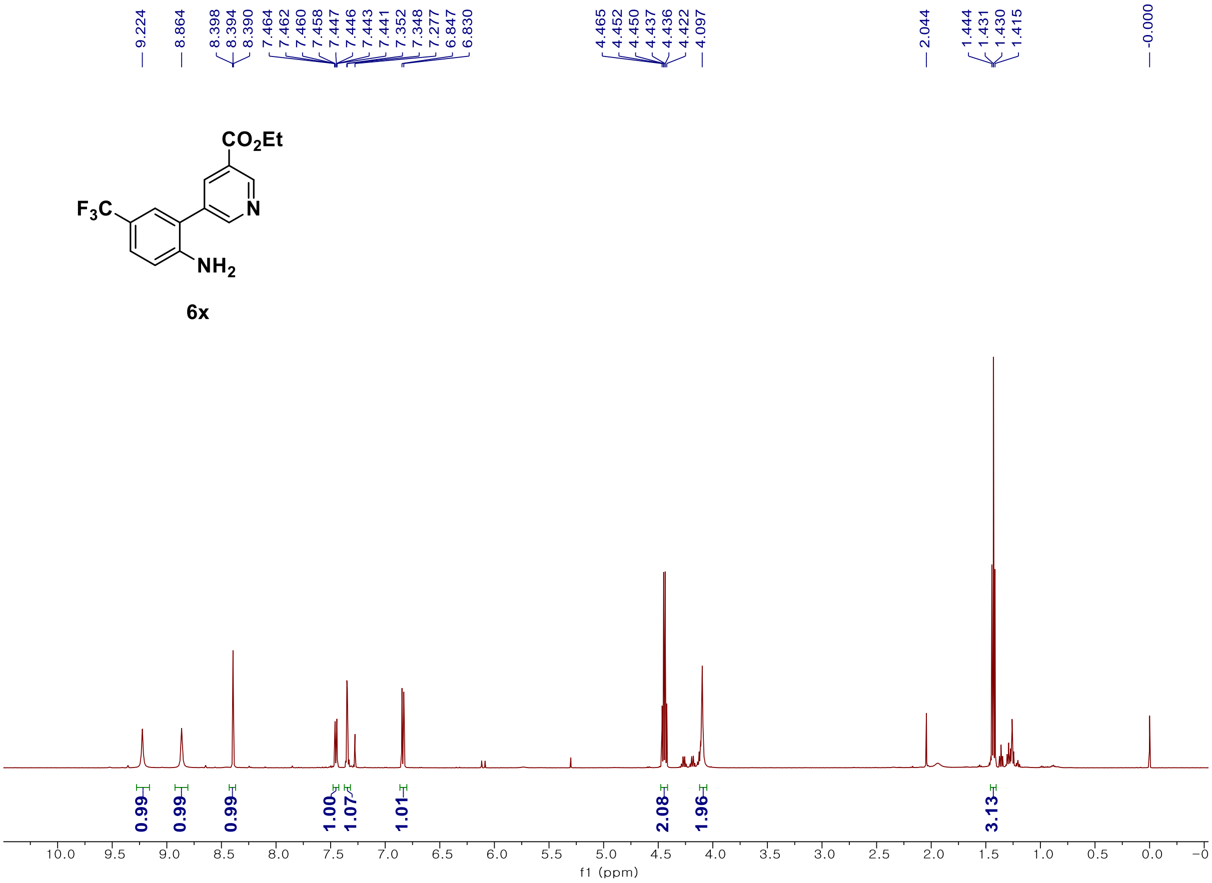
9.224
8.864
8.398
8.394
8.390
7.464
7.462
7.460
7.458
7.447
7.446
7.443
7.441
7.352
7.348
7.277
6.847
6.830
4.465
4.452
4.450
4.437
4.436
4.422
4.097
2.044
1.444
1.431
1.430
1.415
-0.000
CO2Et
F3C
N
NH2
6x
0.99
0.99
0.99
1.00
1.07
1.01
2.08
1.96
3.13
f1 (ppm)

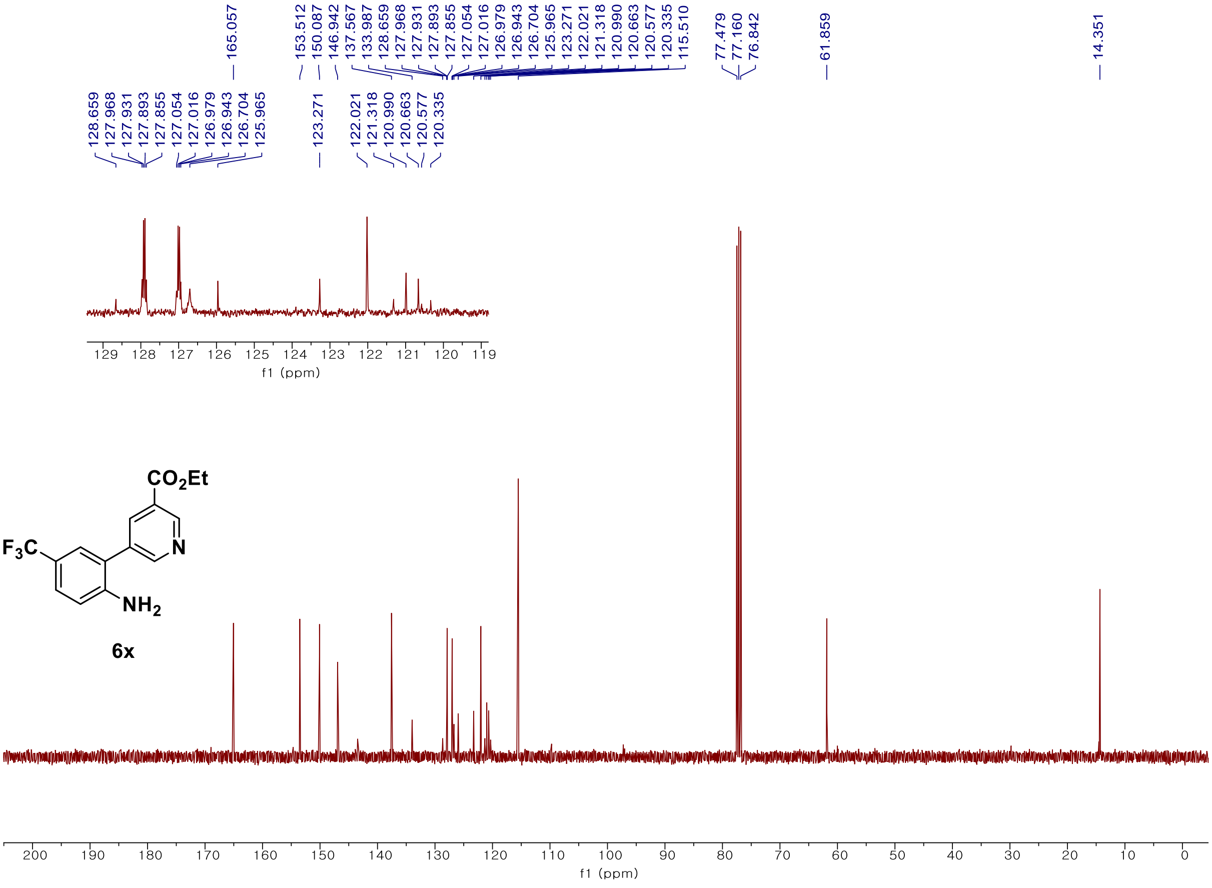
165.057
153.512
150.087
146.942
137.567
133.987
128.659
127.968
127.931
127.893
127.855
127.054
127.016
126.979
126.943
126.704
125.965
123.271
122.021
121.318
120.990
120.663
120.577
120.335
115.510
77.479
77.160
76.842
61.859
14.351
f1 (ppm)
CO2Et
F3C
N
NH2
6x
f1 (ppm)

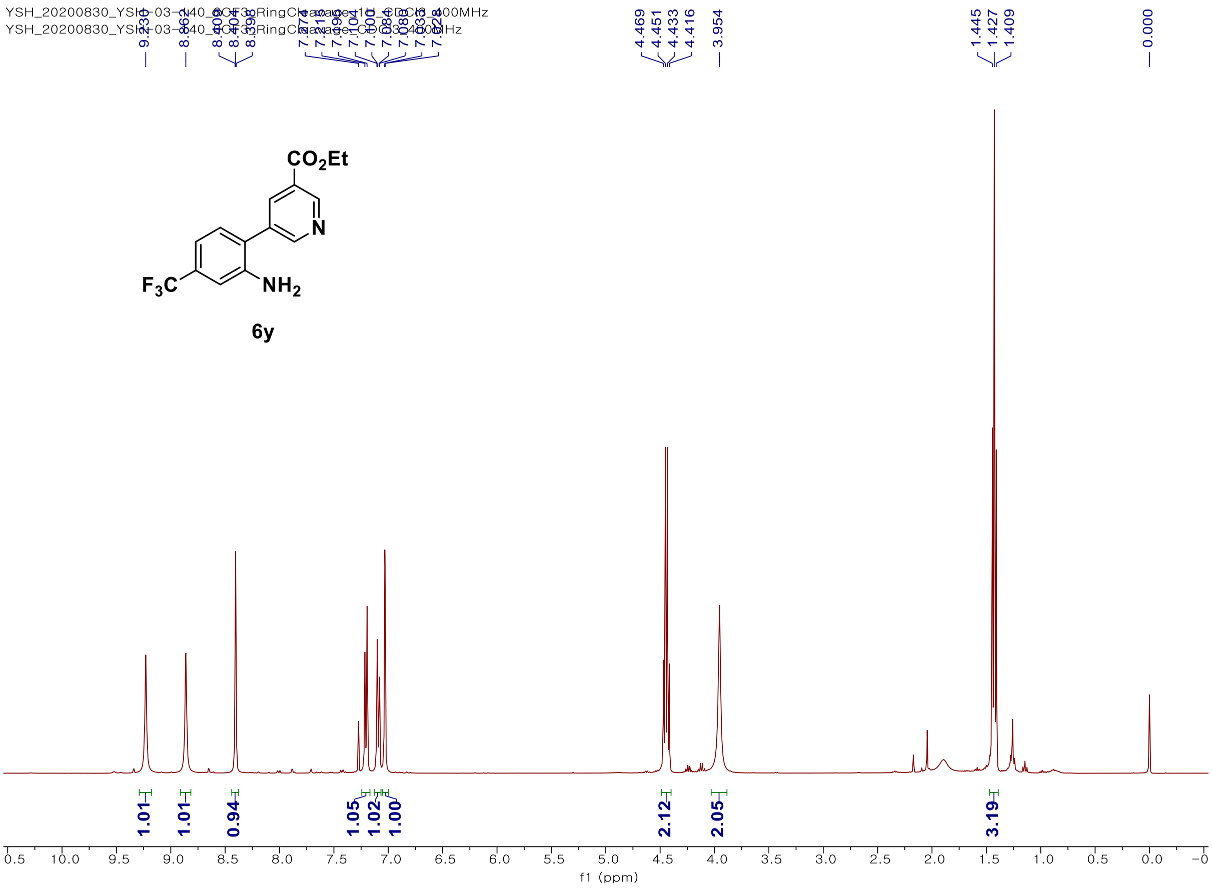
9.230
8.861
8.405
8.401
8.397
8.393
7.274
7.215
7.196
7.104
7.100
7.084
7.080
7.033
7.028
4.469
4.451
4.433
4.416
3.954
1.445
1.427
1.409
0.000
CO2Et
N
F3C
NH2
6y
1.01
1.01
0.94
1.05
1.02
1.00
2.12
2.05
3.19
f1 (ppm)

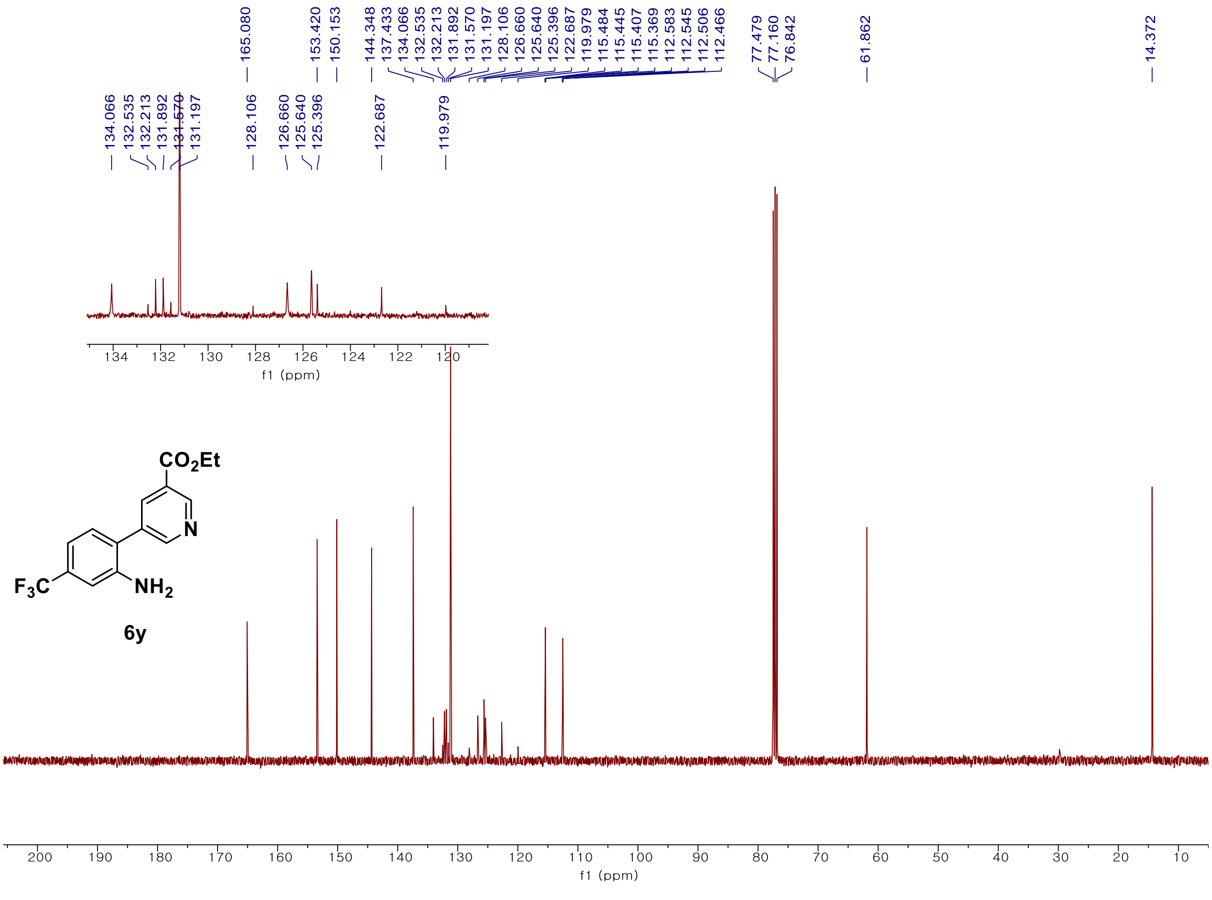
165.080
153.420
150.153
144.348
137.433
134.066
132.535
132.213
131.892
131.570
131.197
128.106
126.660
125.640
125.396
122.687
119.979
115.484
115.445
115.407
115.369
112.583
112.545
112.506
112.466
77.479
77.160
76.842
61.862
14.372
CO2Et
N
F3C
NH2
6y
f1 (ppm)

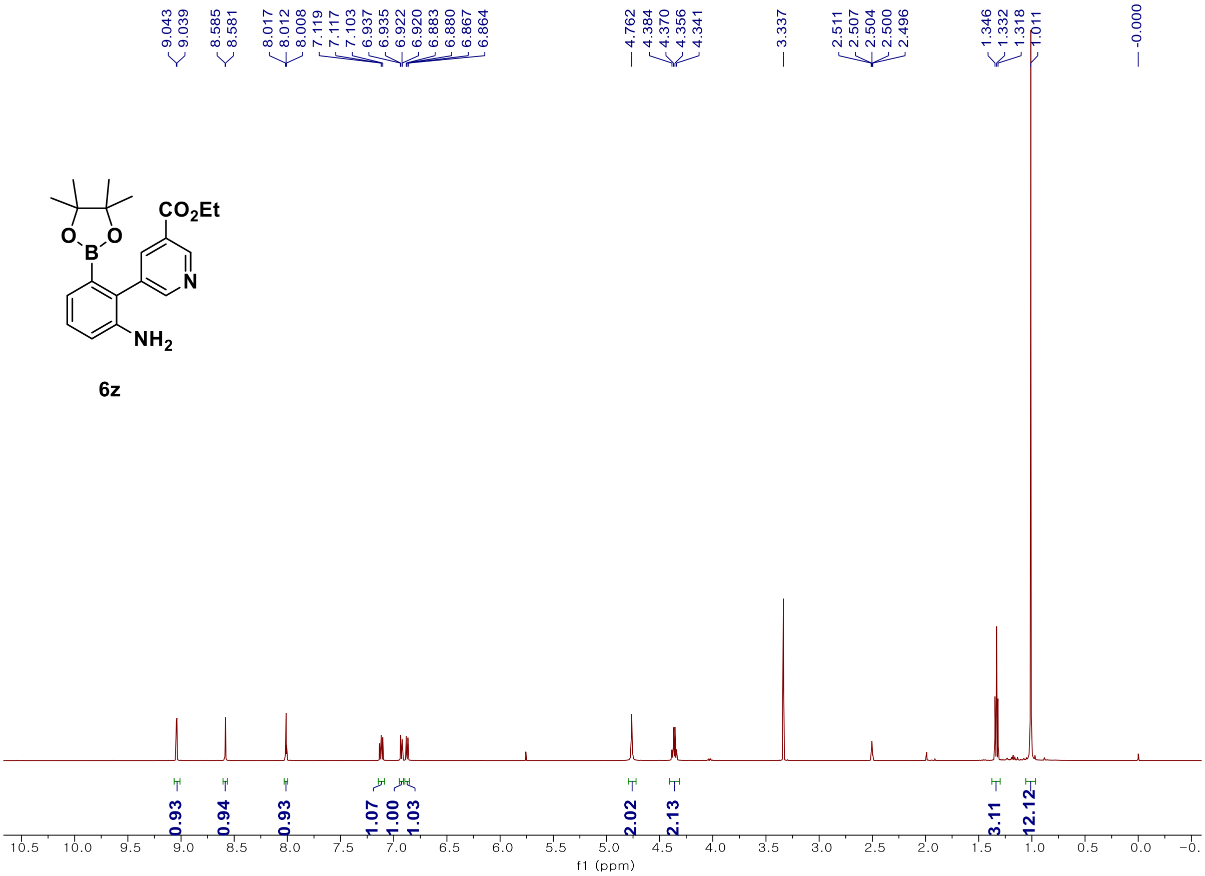
9.043
9.039
8.585
8.581
8.017
8.012
8.008
7.119
7.117
7.103
6.937
6.935
6.922
6.920
6.883
6.880
6.867
6.864
4.762
4.384
4.370
4.356
4.341
3.337
2.511
2.507
2.504
2.500
2.496
1.346
1.332
1.318
1.011
-0.000
CO2Et
O
B
O
N
NH2
6z
0.93
0.94
0.93
1.07
1.00
1.03
2.02
2.13
3.11
12.12
f1 (ppm)

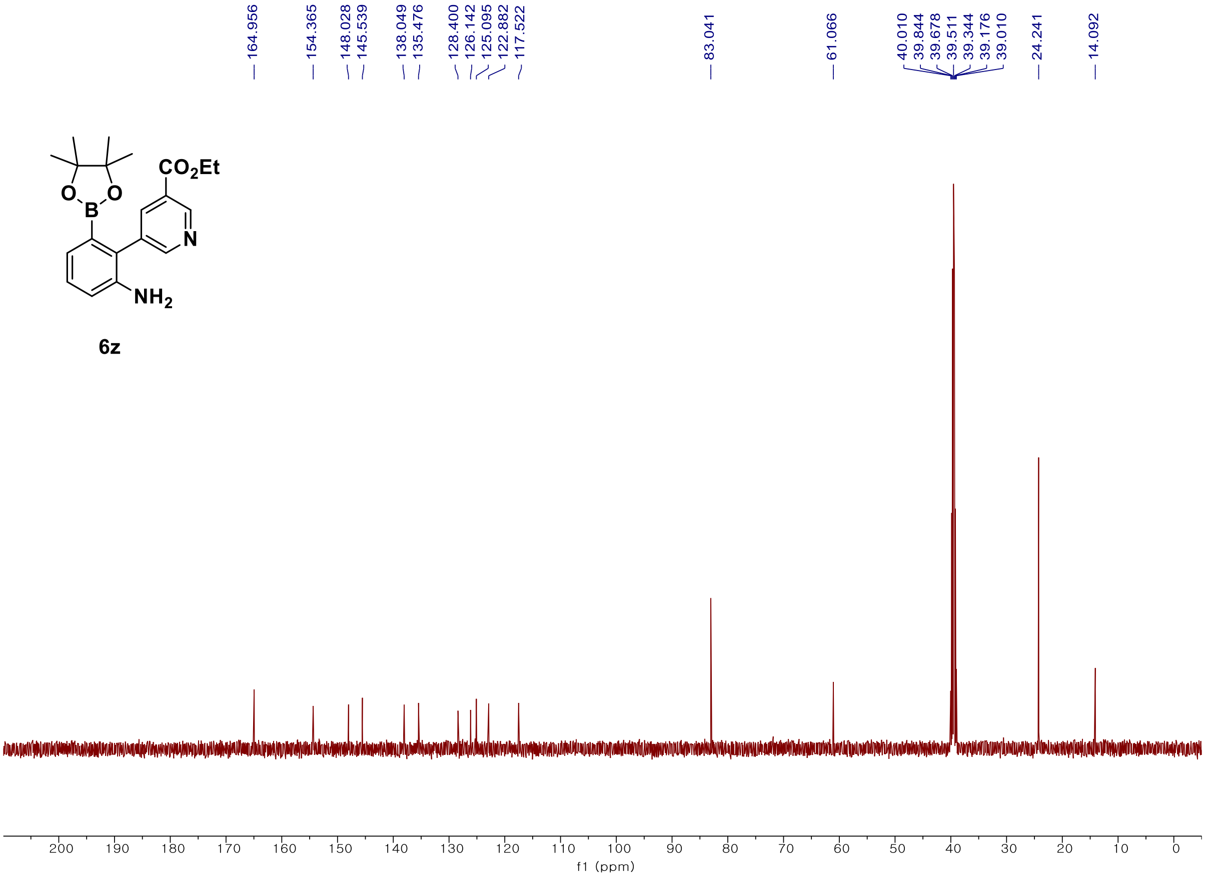
164.956
154.365
148.028
145.539
138.049
135.476
128.400
126.142
125.095
122.882
117.522
83.041
61.066
40.010
39.844
39.678
39.511
39.344
39.176
39.010
24.241
14.092
CO2Et
O
B
O
N
NH2
6z
f1 (ppm)

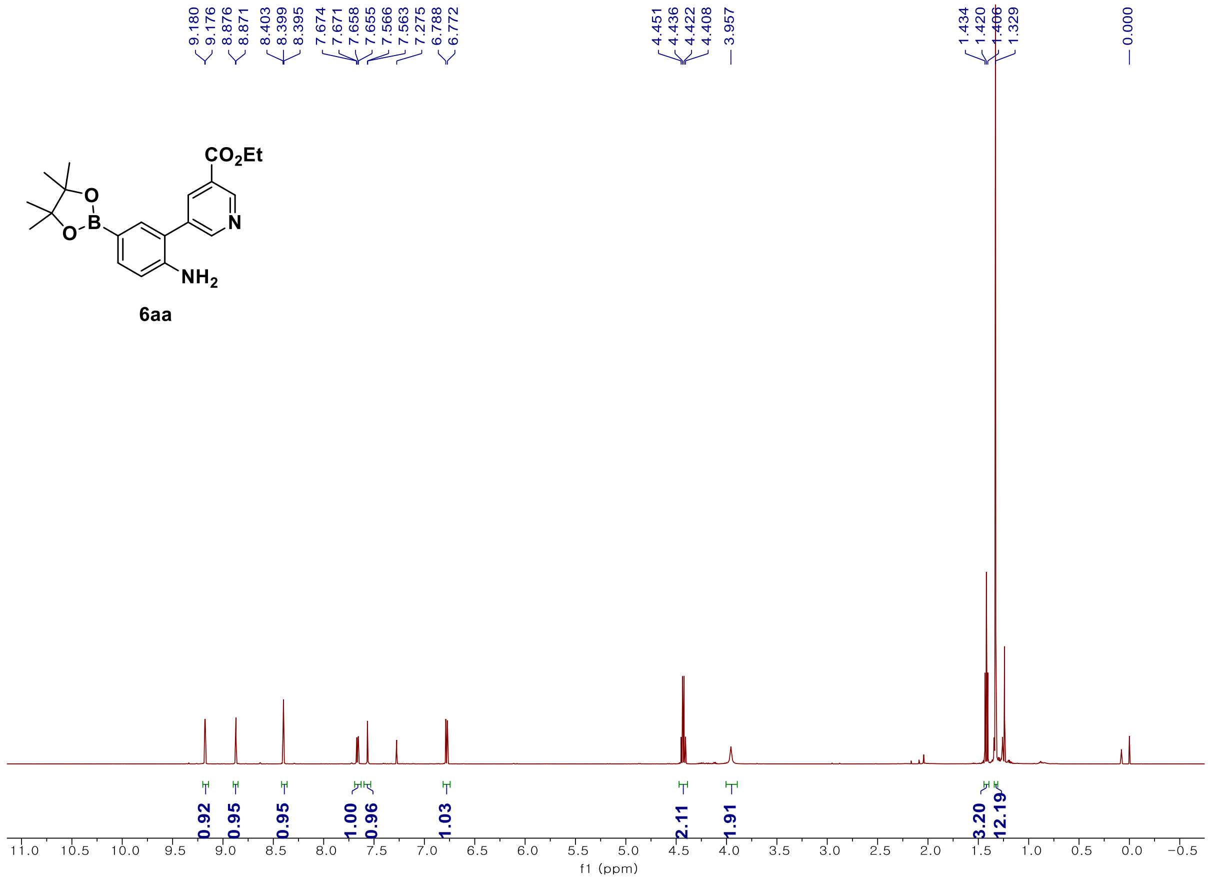
9.180
9.176
8.876
8.871
8.403
8.399
8.395
7.674
7.671
7.658
7.655
7.566
7.563
7.275
6.788
6.772
4.451
4.436
4.422
4.408
3.957
1.434
1.420
1.406
1.329
0.000
CO_2Et
O
B
O
N
NH_2
6aa
0.92
0.95
0.95
1.00
0.96
1.03
2.11
1.91
3.20
12.19
f1 (ppm)

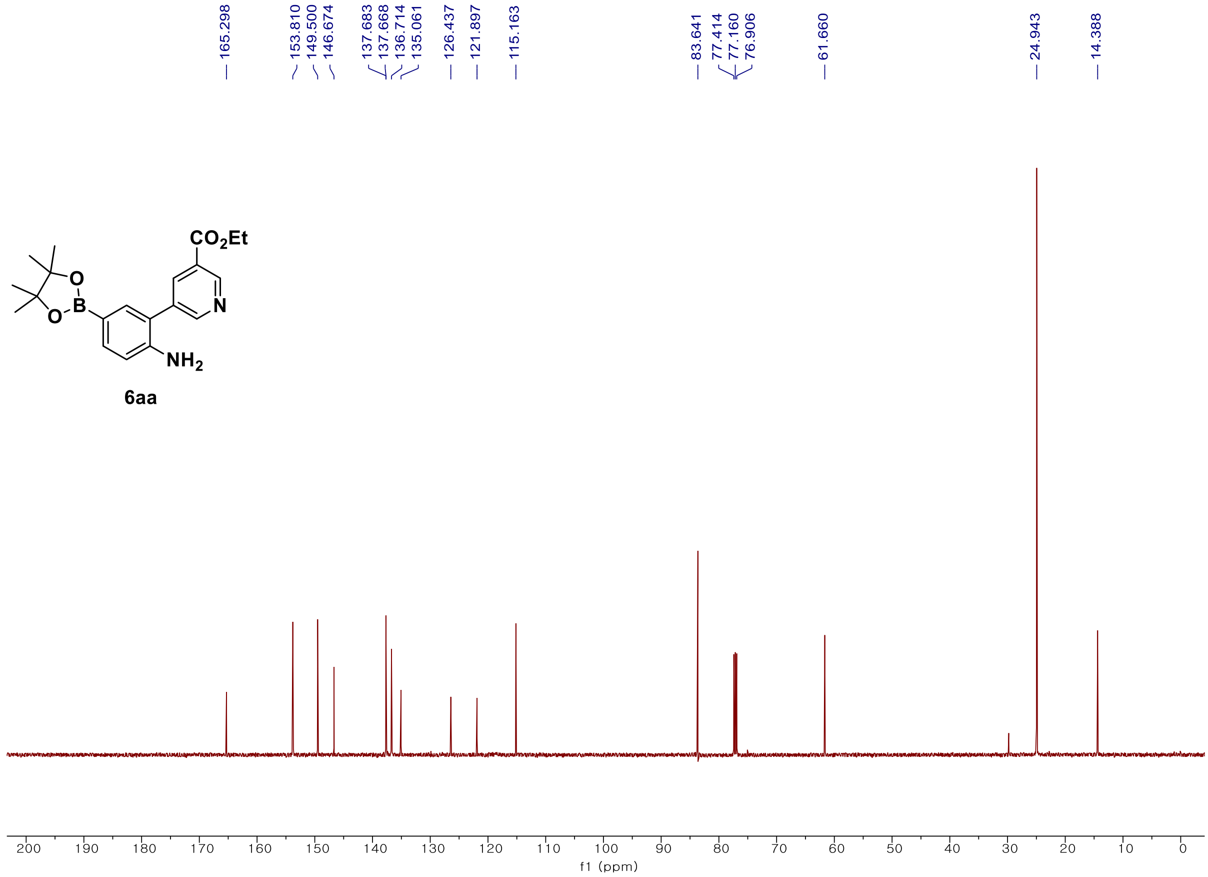
165.298
153.810
149.500
146.674
137.683
137.668
136.714
135.061
126.437
121.897
115.163
83.641
77.414
77.160
76.906
61.660
24.943
14.388
CO_2Et
O
B
O
N
NH_2
6aa
f1 (ppm)

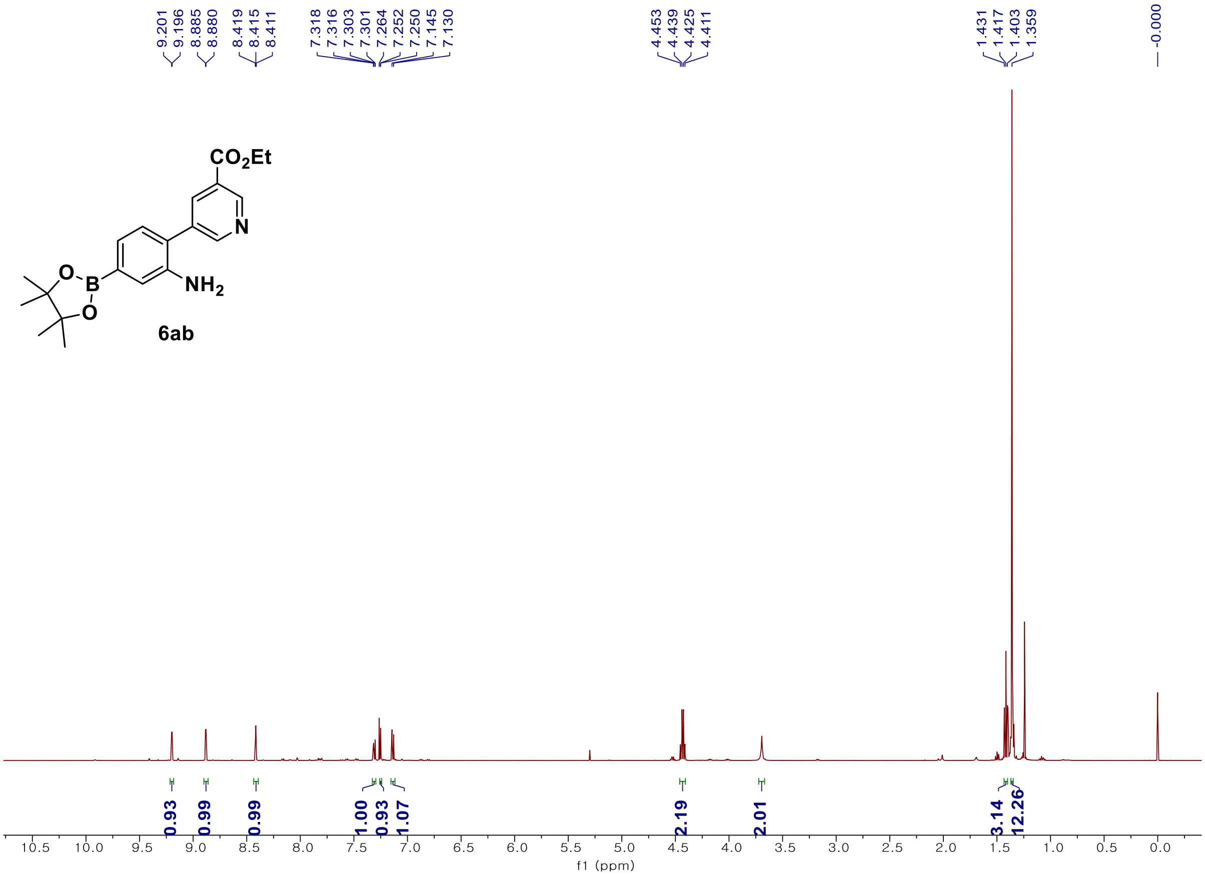

9.201
9.196
8.885
8.880
8.419
8.415
8.411
7.318
7.316
7.303
7.301
7.264
7.252
7.250
7.145
7.130
4.453
4.439
4.425
4.411
1.431
1.417
1.403
1.359
-0.000
CO2Et
N
O
B
O
NH2
0.93
0.99
0.99
1.00
0.93
1.07
2.19
2.01
3.14
12.26
f1 (ppm)

6ab

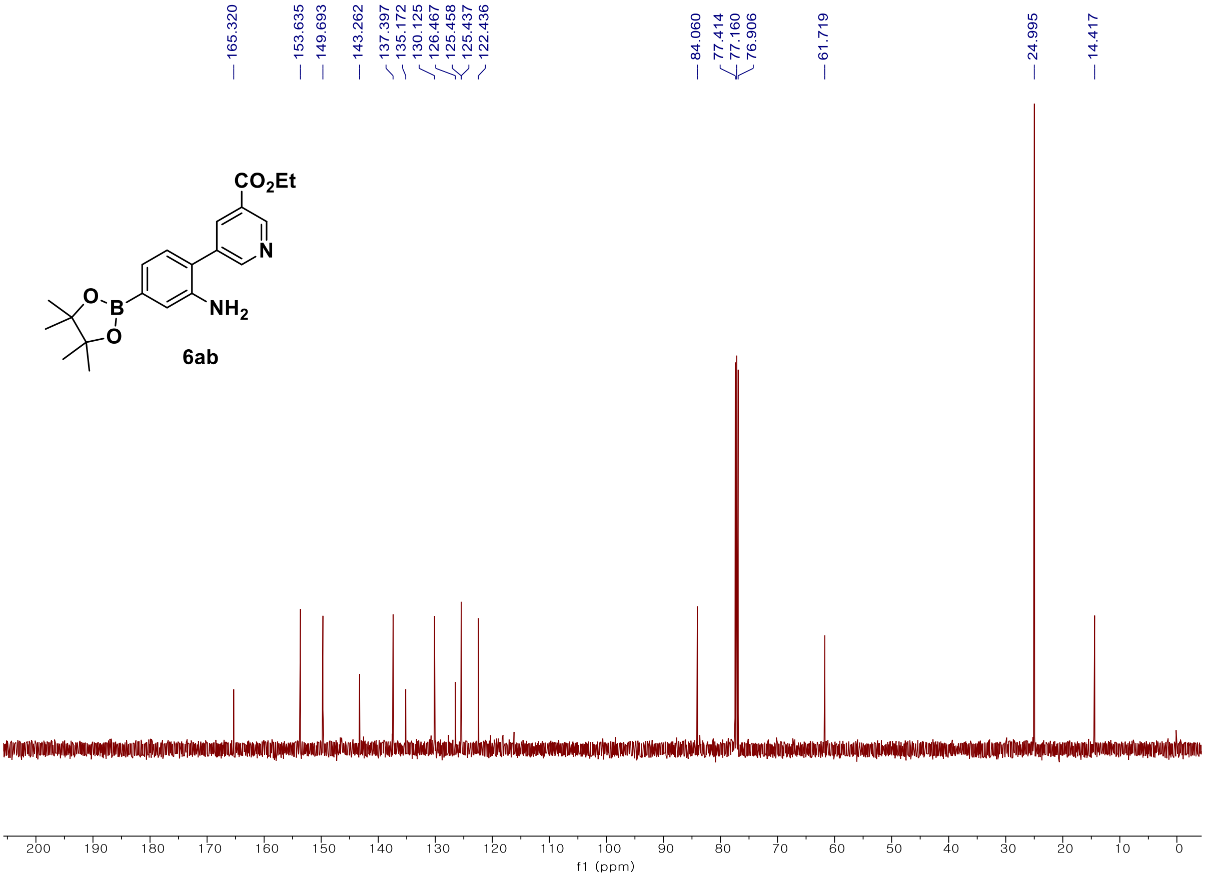

165.320
153.635
149.693
143.262
137.397
135.172
130.125
126.467
125.458
125.437
122.436
84.060
77.414
77.160
76.906
61.719
24.995
14.417
CO2Et
N
O
B
O
NH2
f1 (ppm)

6ab

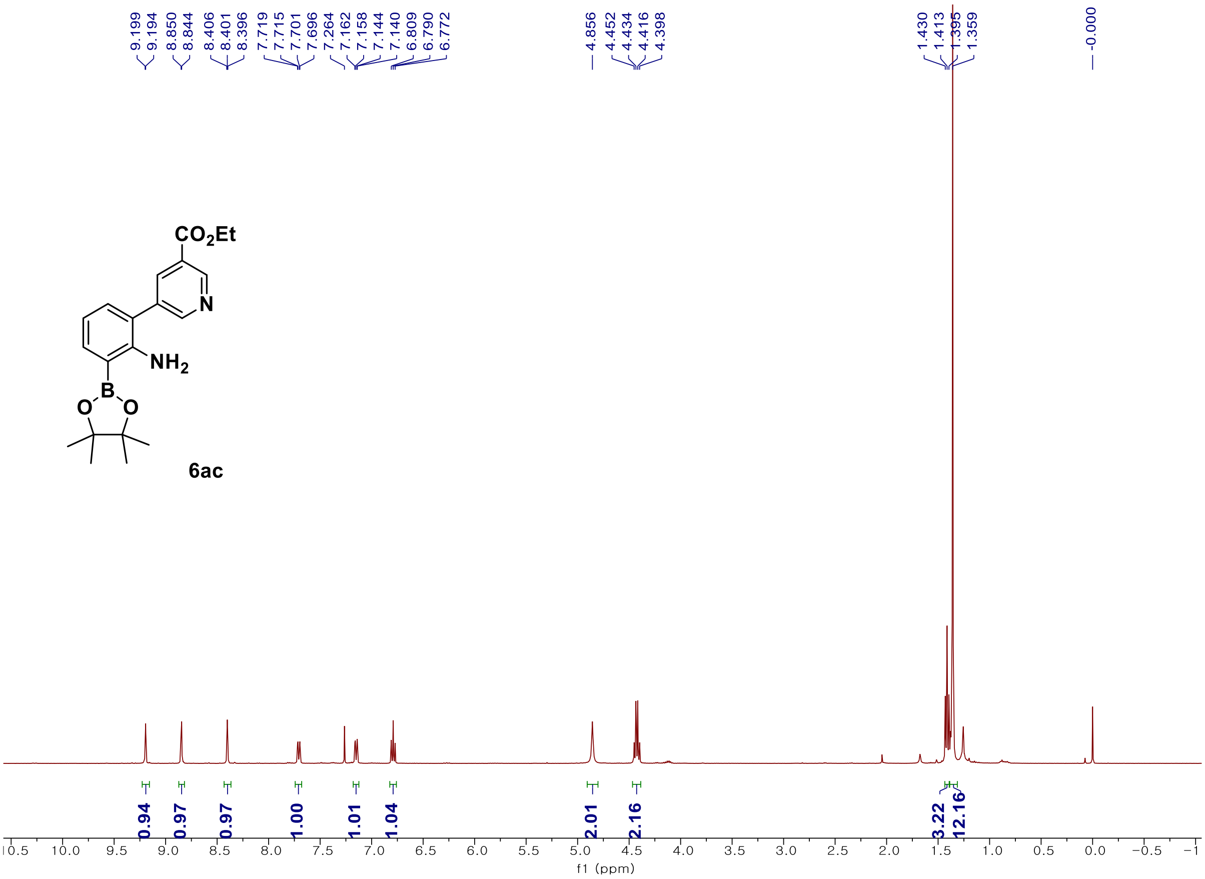

9.199
9.194
8.850
8.844
8.406
8.401
8.396
7.719
7.715
7.701
7.696
7.264
7.162
7.158
7.144
7.140
6.809
6.790
6.772
4.856
4.452
4.434
4.416
4.398
1.430
1.413
1.395
1.359
-0.000
CO_2Et
N
NH_2
B
O
O
6ac
0.94
0.97
0.97
1.00
1.01
1.04
2.01
2.16
3.22
12.16
f1 (ppm)

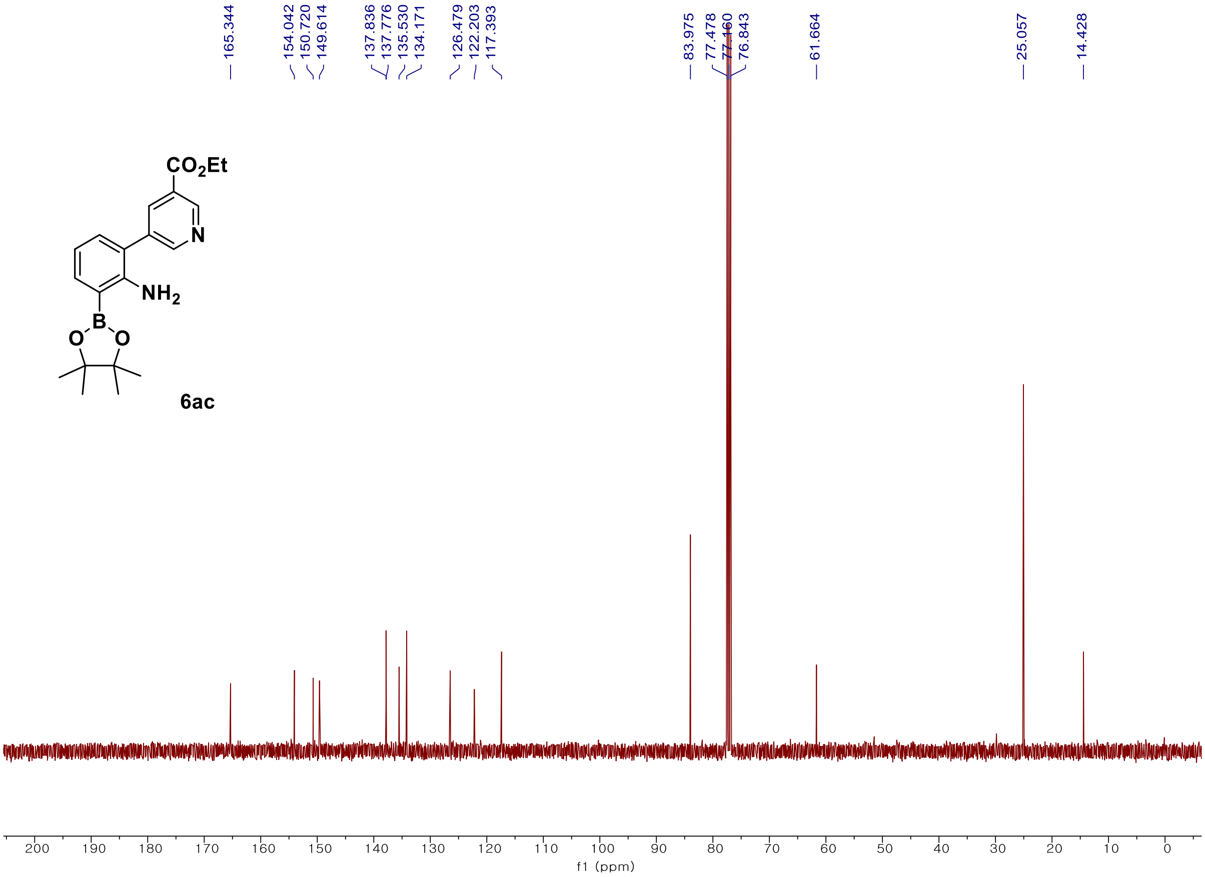

165.344
154.042
150.720
149.614
137.836
137.776
135.530
134.171
126.479
122.203
117.393
83.975
77.478
77.160
76.843
61.664
25.057
14.428
CO_2Et
N
NH_2
B
O
O
6ac
f1 (ppm)

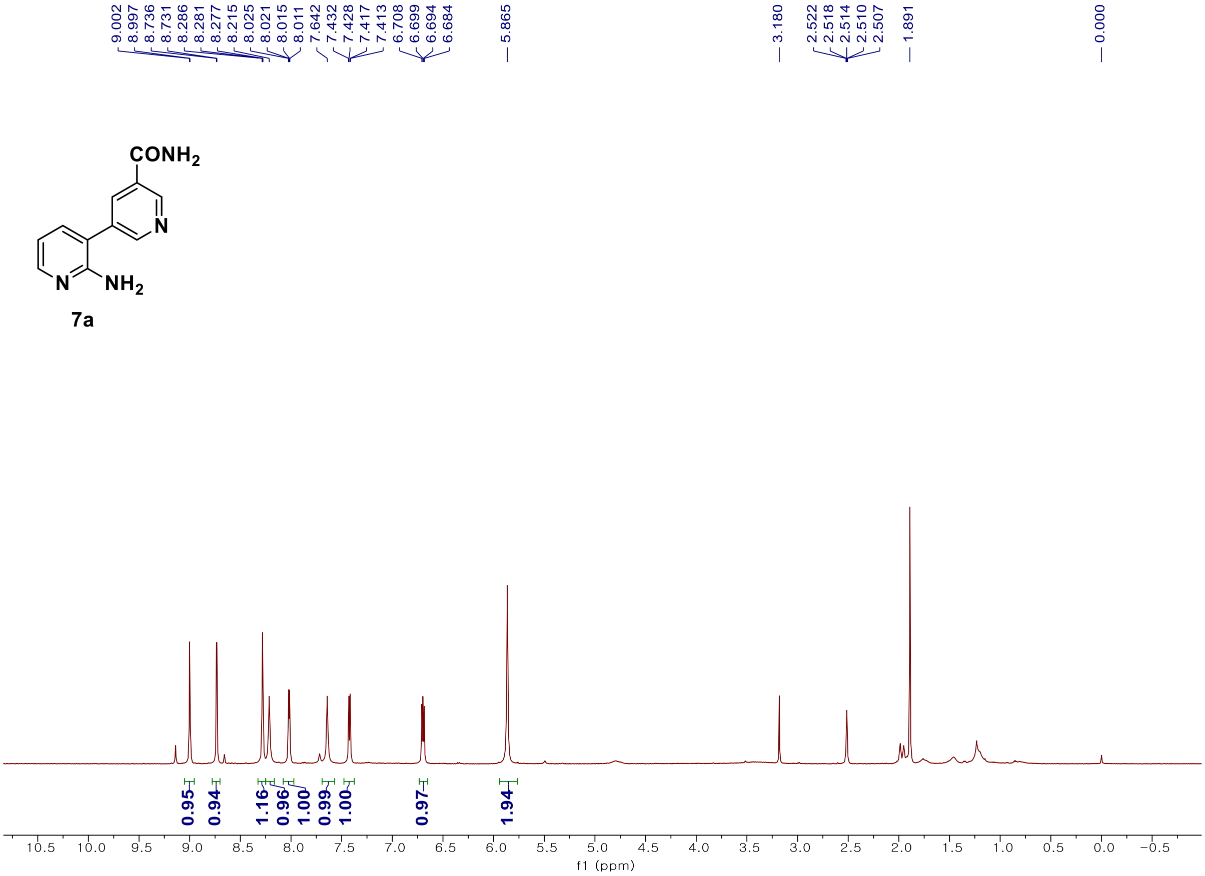
9.002
8.997
8.736
8.731
8.286
8.281
8.277
8.215
8.025
8.021
8.015
8.011
7.642
7.432
7.428
7.417
7.413
6.708
6.699
6.694
6.684
5.865
3.180
2.522
2.518
2.514
2.510
2.507
1.891
0.000
CONH2
N
N
NH2
7a
0.95
0.94
1.16
0.96
1.00
0.99
1.00
0.97
1.94
f1 (ppm)

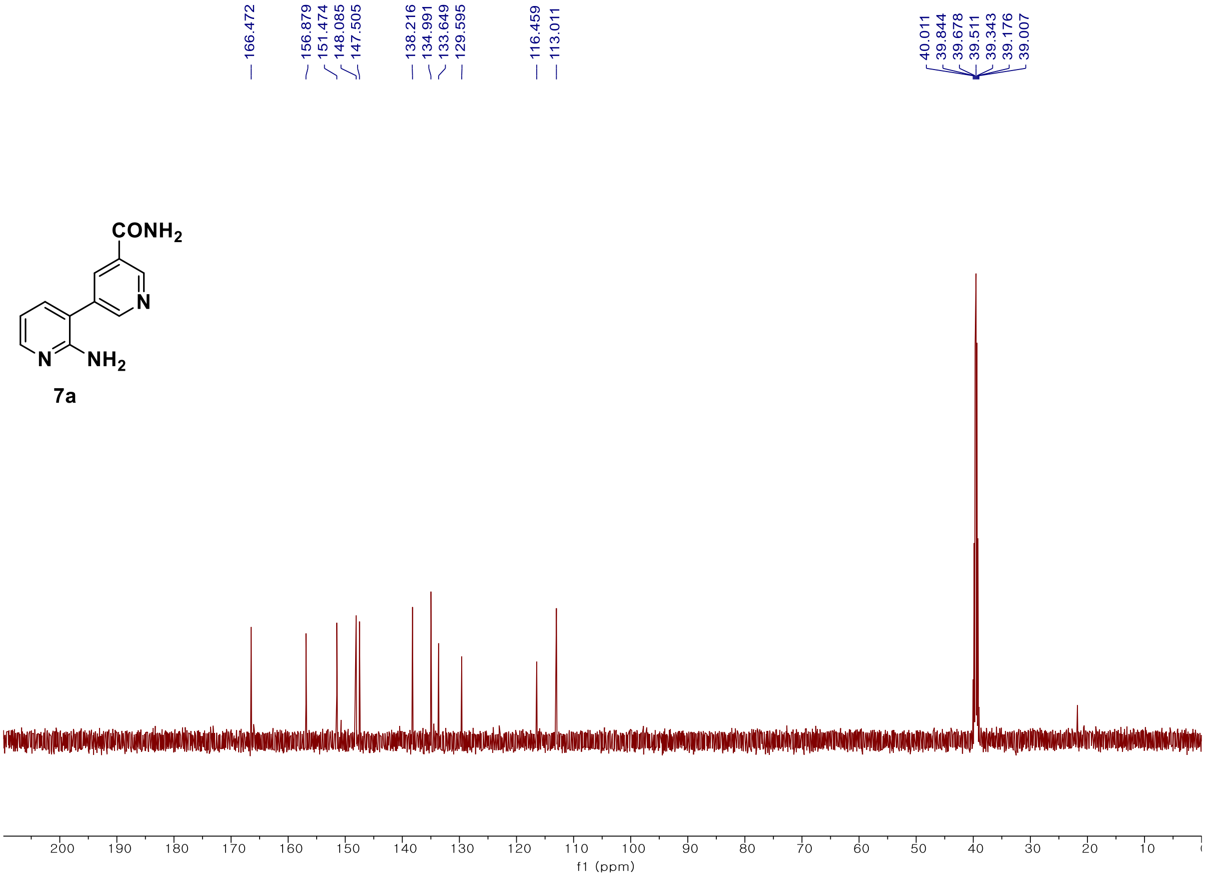
166.472
156.879
151.474
148.085
147.505
138.216
134.991
133.649
129.595
116.459
113.011
40.011
39.844
39.678
39.511
39.343
39.176
39.007
CONH2
N
N
NH2
7a
f1 (ppm)

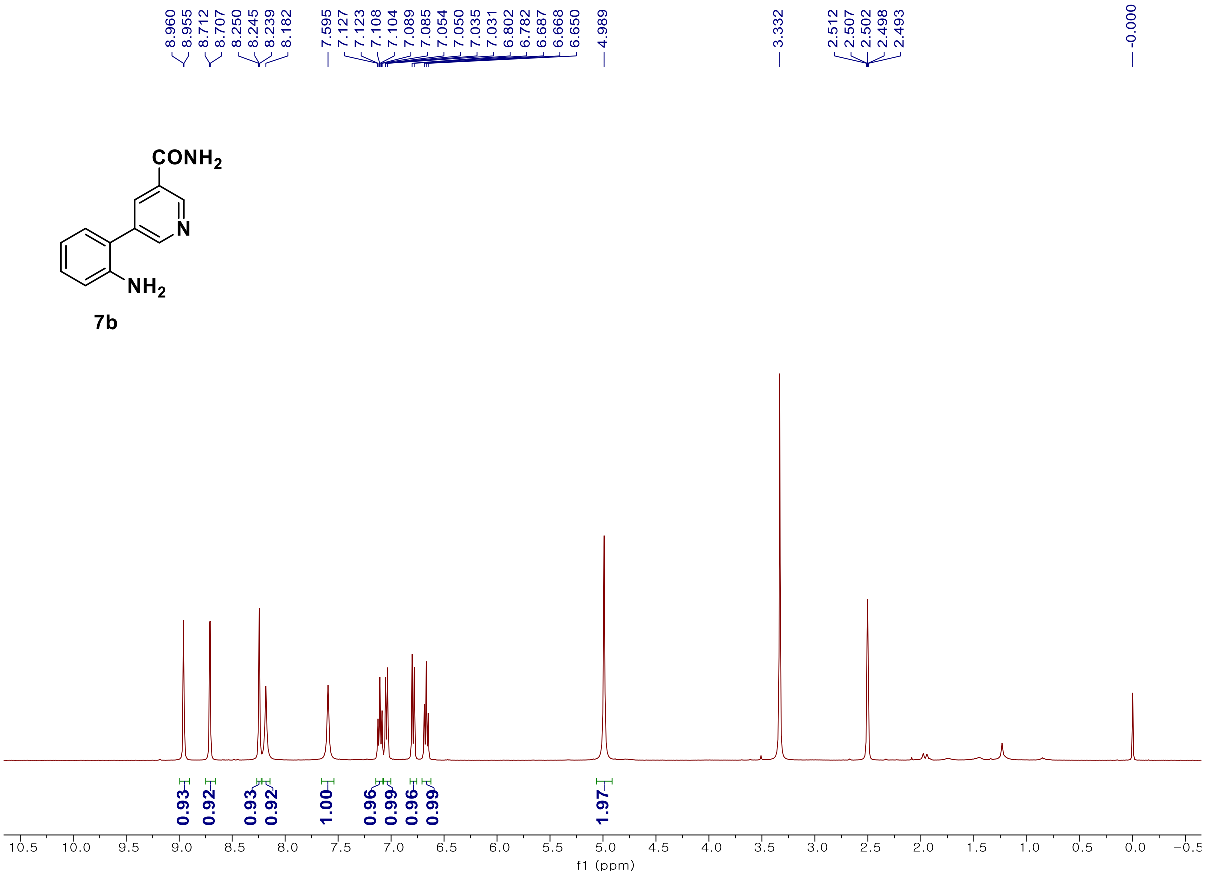
8.960
8.955
8.712
8.707
8.250
8.245
8.239
8.182
7.595
7.127
7.123
7.108
7.104
7.089
7.085
7.054
7.050
7.035
7.031
6.802
6.782
6.687
6.668
6.650
4.989
3.332
2.512
2.507
2.502
2.498
2.493
-0.000
CONH2
N
NH2
7b
0.93
0.92
0.93
0.92
1.00
0.96
0.99
0.96
0.99
1.97
f1 (ppm)

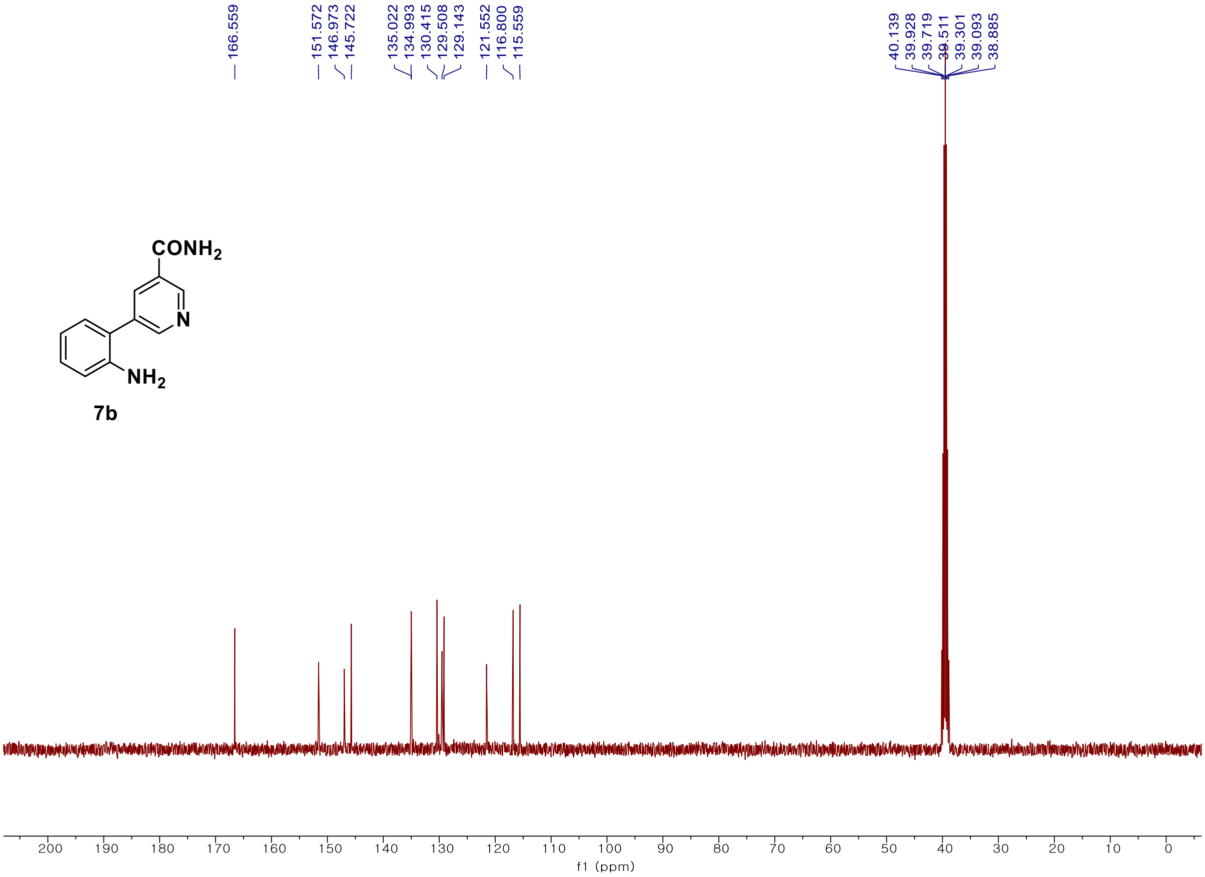
166.559
151.572
146.973
145.722
135.022
134.993
130.415
129.508
129.143
121.552
116.800
115.559
40.139
39.928
39.719
39.511
39.301
39.093
38.885
CONH2
N
NH2
7b
f1 (ppm)

5o

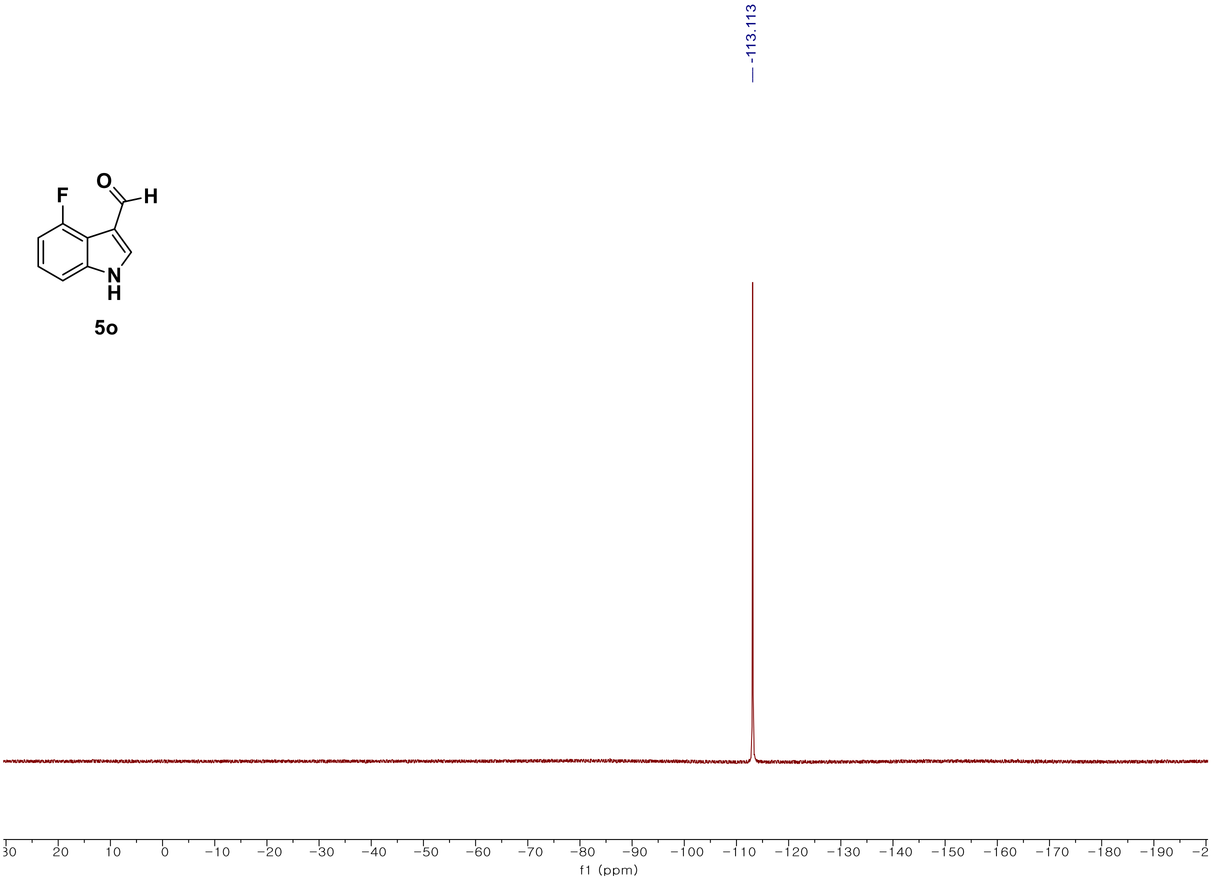

-113.113
30 20 10 0 −10 −20 −30 −40 −50 −60 −70 −80 −90 −100 −110 −120 −130 −140 −150 −160 −170 −180 −190 −2
f1 (ppm)

6o

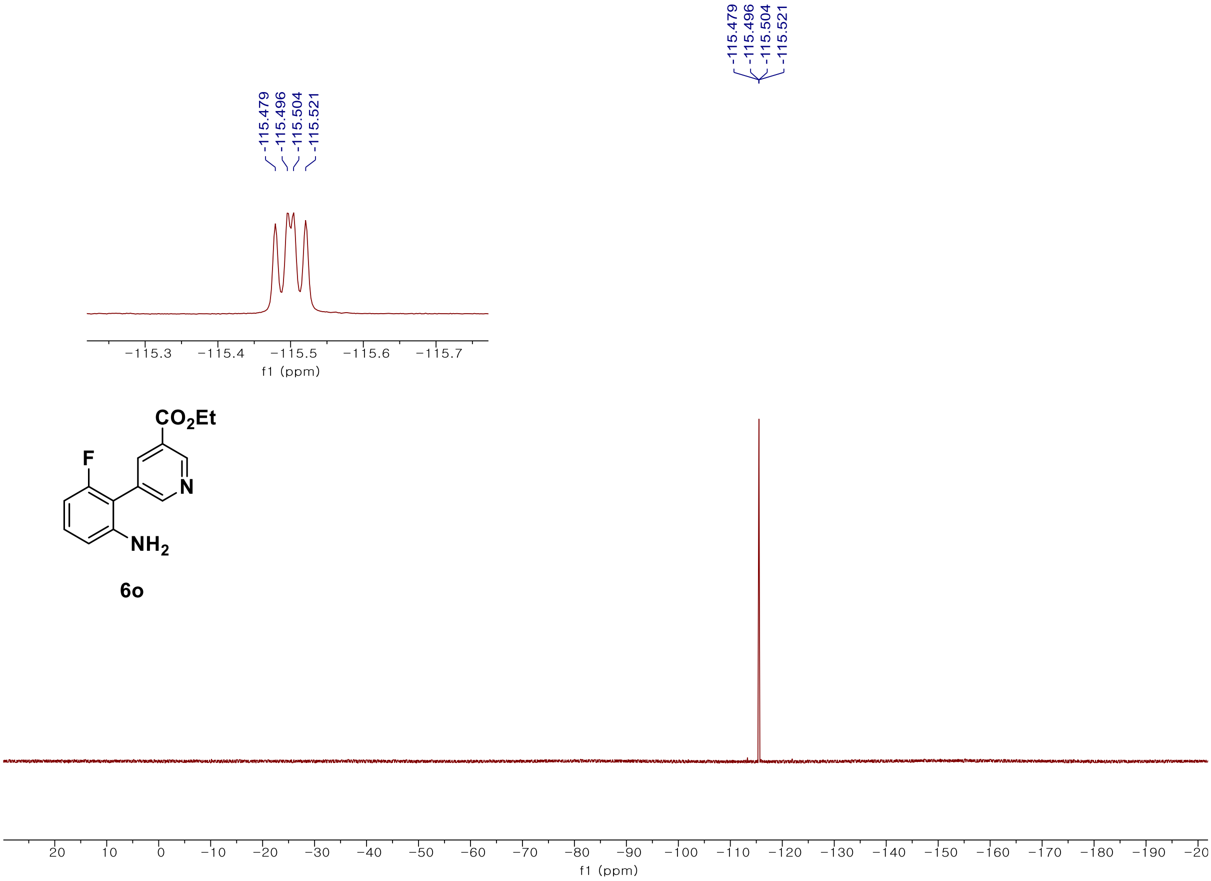

-115.479
-115.496
-115.504
-115.521
−115.3 −115.4 −115.5 −115.6 −115.7
f1 (ppm)
20 10 0 −10 −20 −30 −40 −50 −60 −70 −80 −90 −100 −110 −120 −130 −140 −150 −160 −170 −180 −190 −20
f1 (ppm)

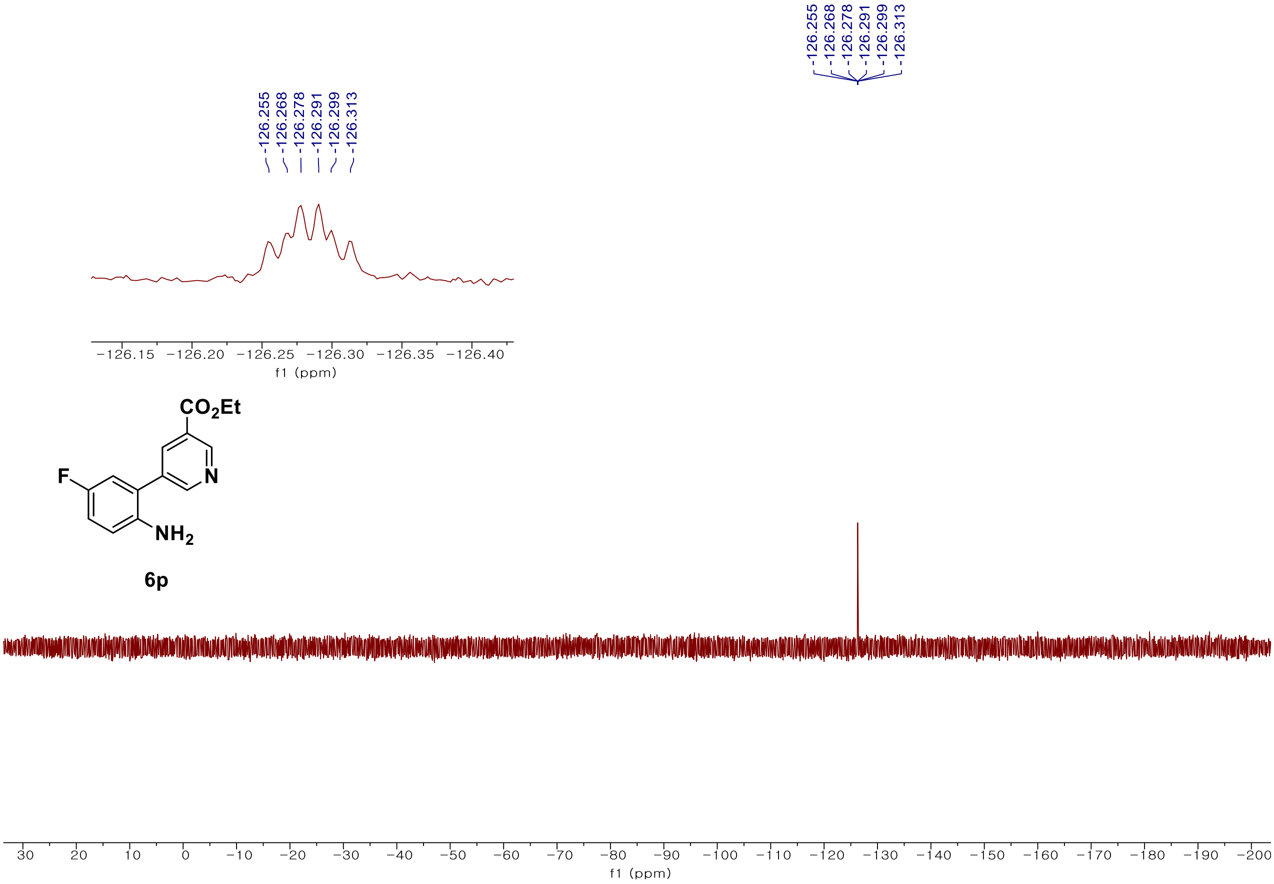

-126.255
-126.268
-126.278
-126.291
-126.299
-126.313
−126.15 −126.20 −126.25 −126.30 −126.35 −126.40
f1 (ppm)
CO2Et
F
N
NH2
6p
30 20 10 0 −10 −20 −30 −40 −50 −60 −70 −80 −90 −100 −110 −120 −130 −140 −150 −160 −170 −180 −190 −200
f1 (ppm)

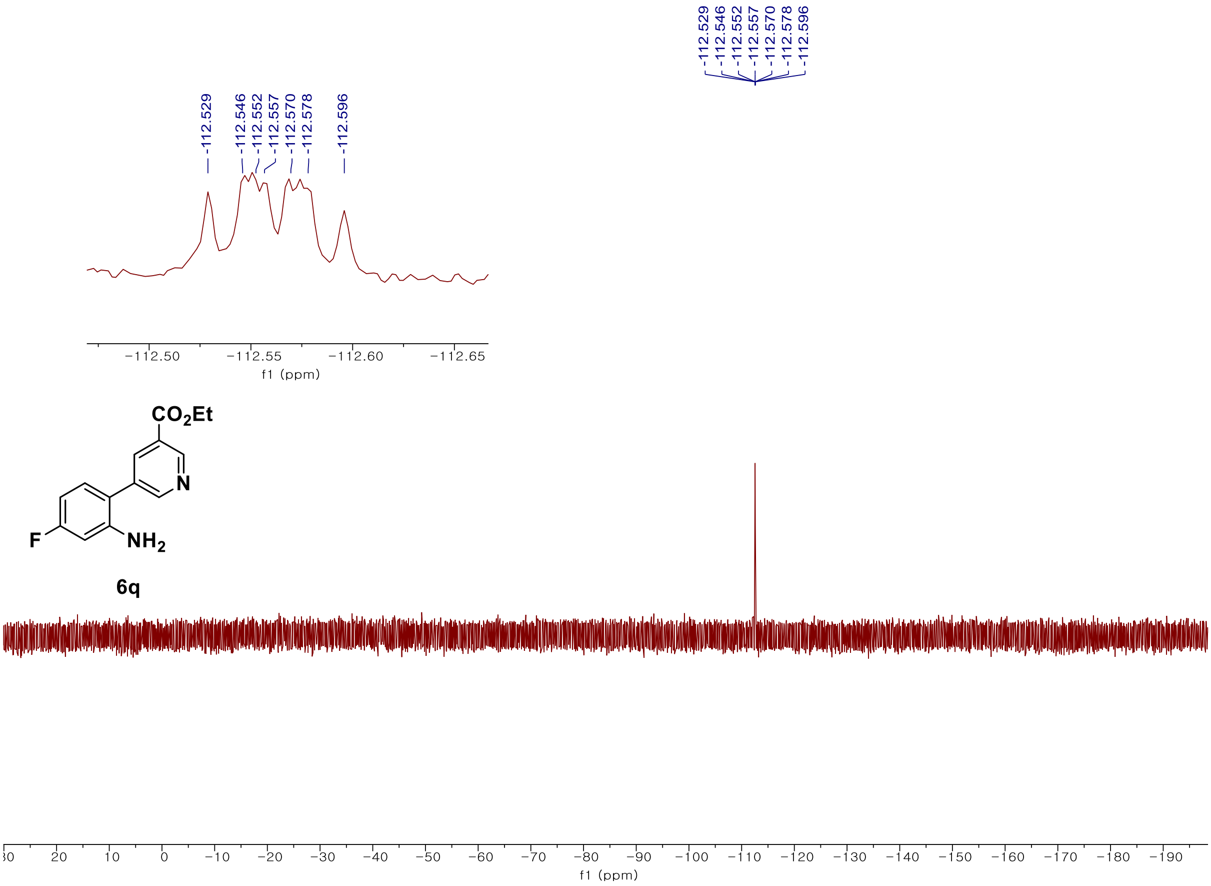

-112.529
-112.546
-112.552
-112.557
-112.570
-112.578
-112.596
−112.50 −112.55 −112.60 −112.65
f1 (ppm)
CO2Et
N
F
NH2
6q
20 10 0 −10 −20 −30 −40 −50 −60 −70 −80 −90 −100 −110 −120 −130 −140 −150 −160 −170 −180 −190
f1 (ppm)

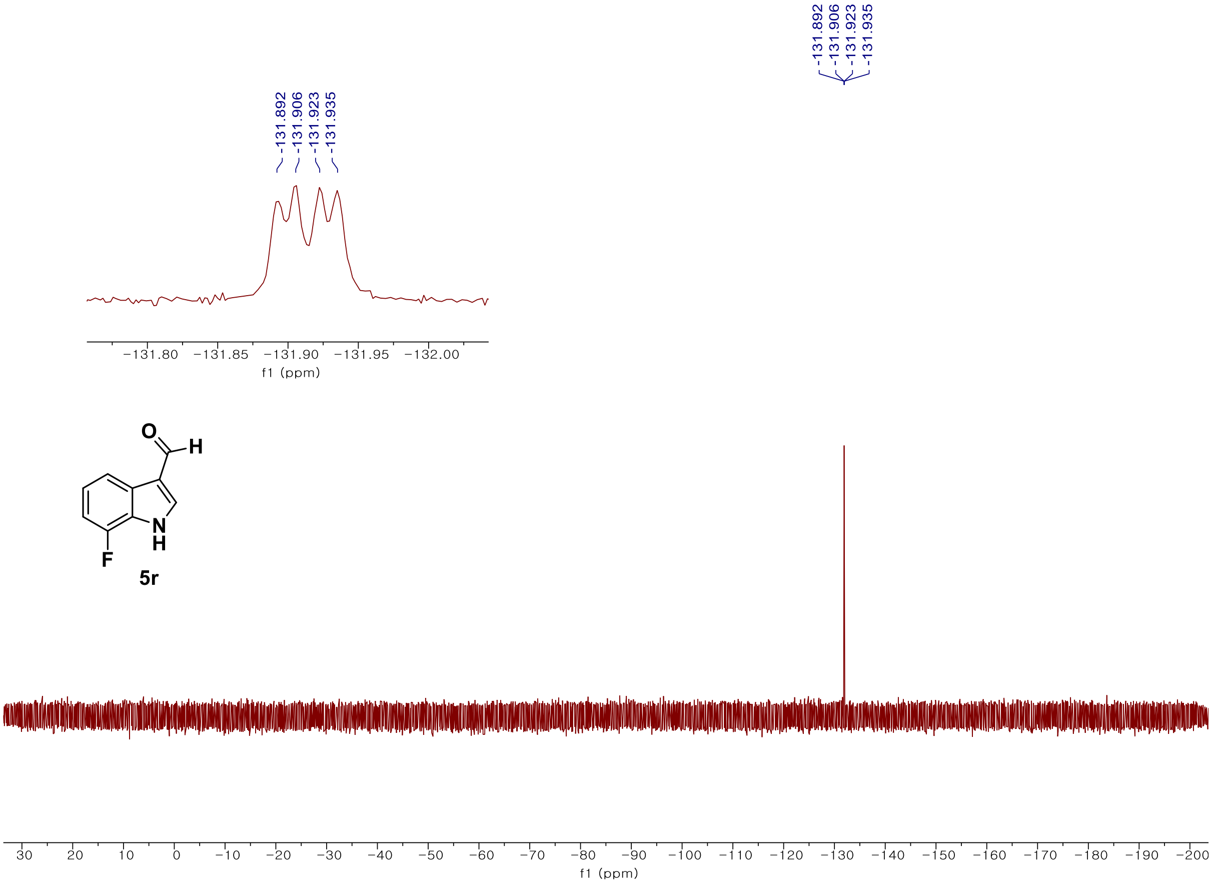
-131.892
-131.906
-131.923
-131.935
−131.80 −131.85 −131.90 −131.95 −132.00
f1 (ppm)
O
H
N
H
F
5r
30 20 10 0 −10 −20 −30 −40 −50 −60 −70 −80 −90 −100 −110 −120 −130 −140 −150 −160 −170 −180 −190 −200
f1 (ppm)

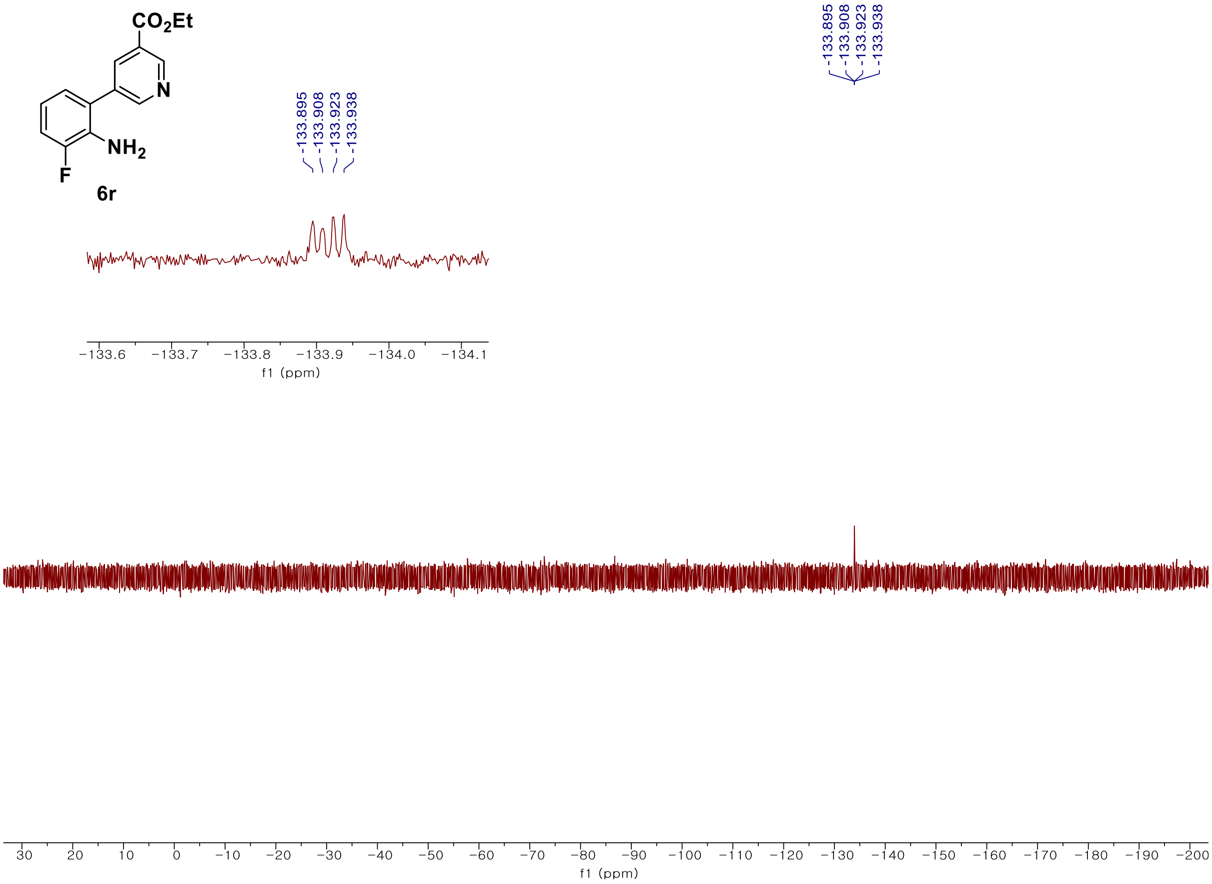
-133.895
-133.908
-133.923
-133.938
CO2Et
N
NH2
F
6r
−133.6 −133.7 −133.8 −133.9 −134.0 −134.1
f1 (ppm)
30 20 10 0 −10 −20 −30 −40 −50 −60 −70 −80 −90 −100 −110 −120 −130 −140 −150 −160 −170 −180 −190 −200
f1 (ppm)

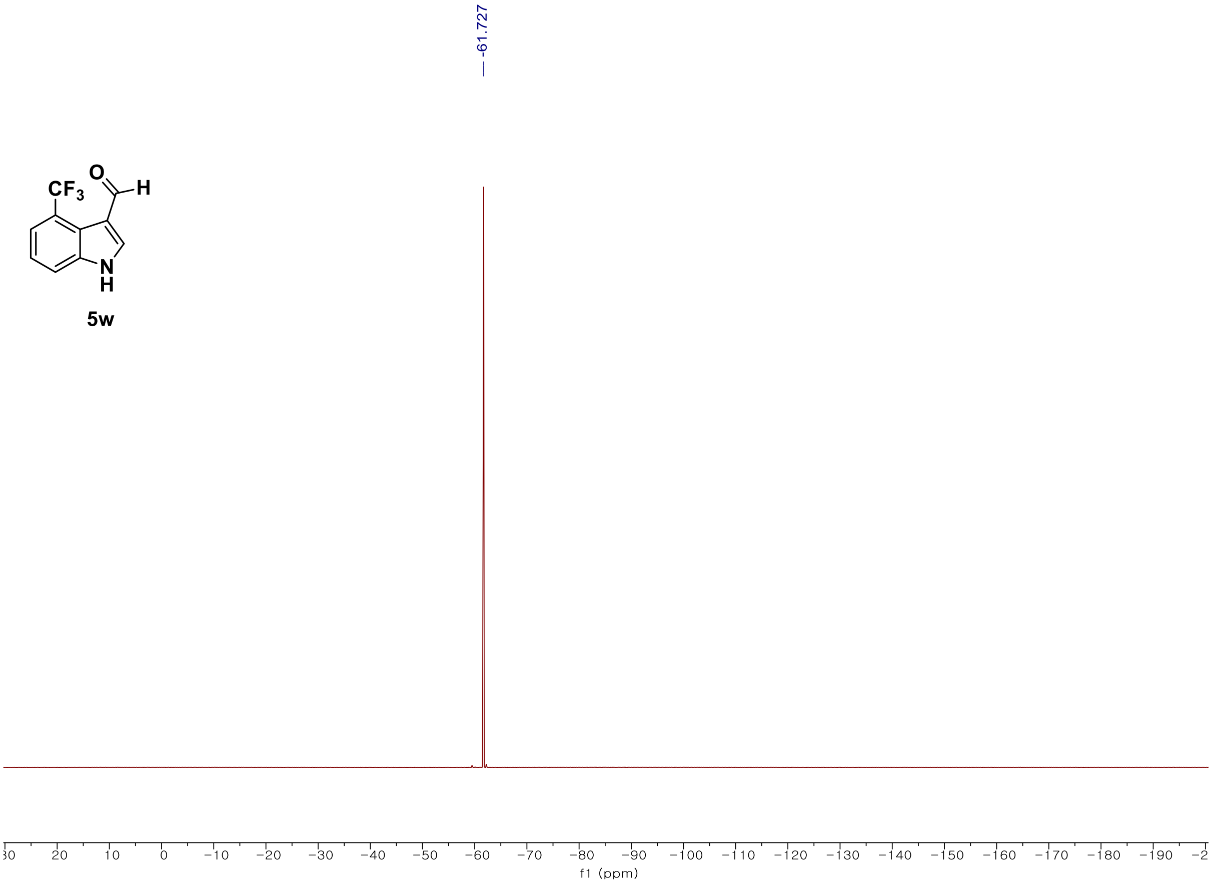
-61.727
CF3
O
H
N
H
5w
30 20 10 0 −10 −20 −30 −40 −50 −60 −70 −80 −90 −100 −110 −120 −130 −140 −150 −160 −170 −180 −190 −200
f1 (ppm)

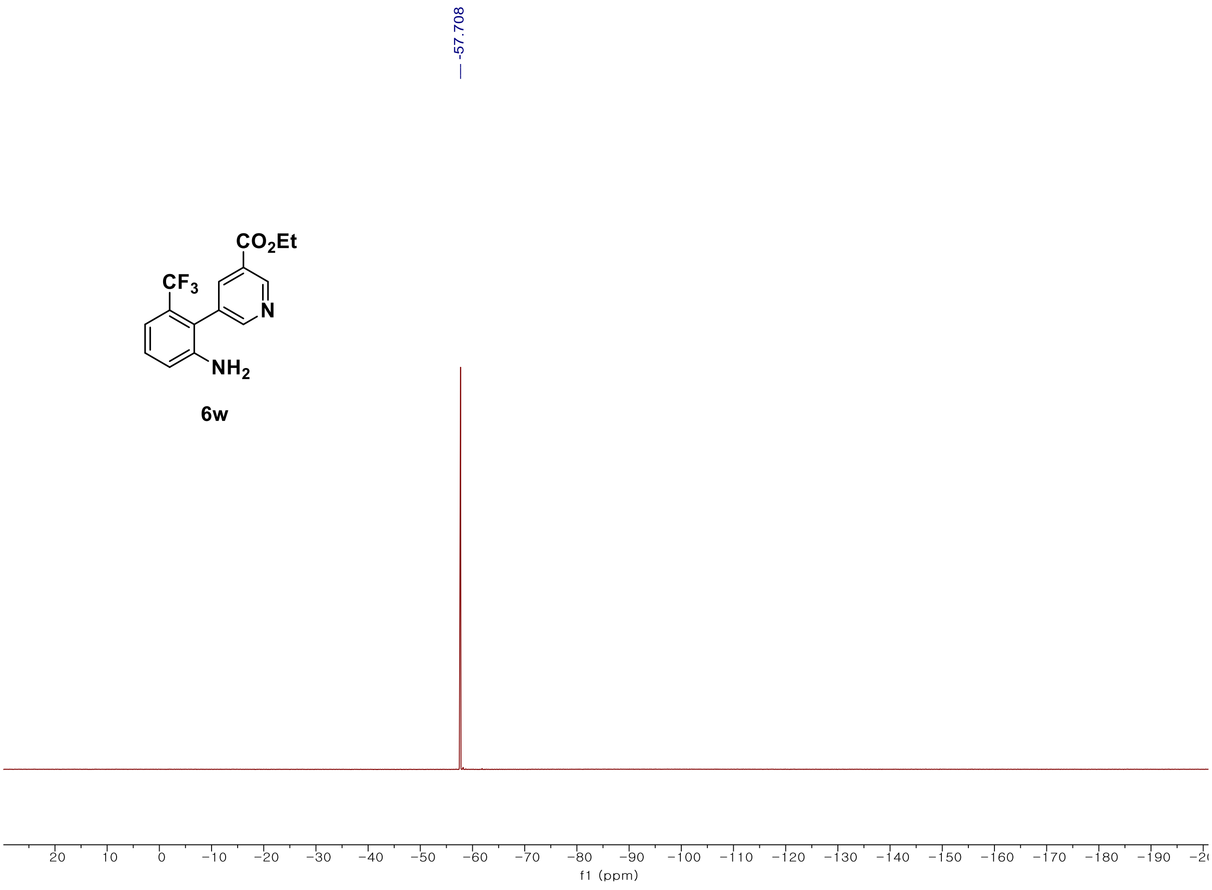
-57.708
CO2Et
CF3
N
NH2
6w
20 10 0 −10 −20 −30 −40 −50 −60 −70 −80 −90 −100 −110 −120 −130 −140 −150 −160 −170 −180 −190 −200
f1 (ppm)

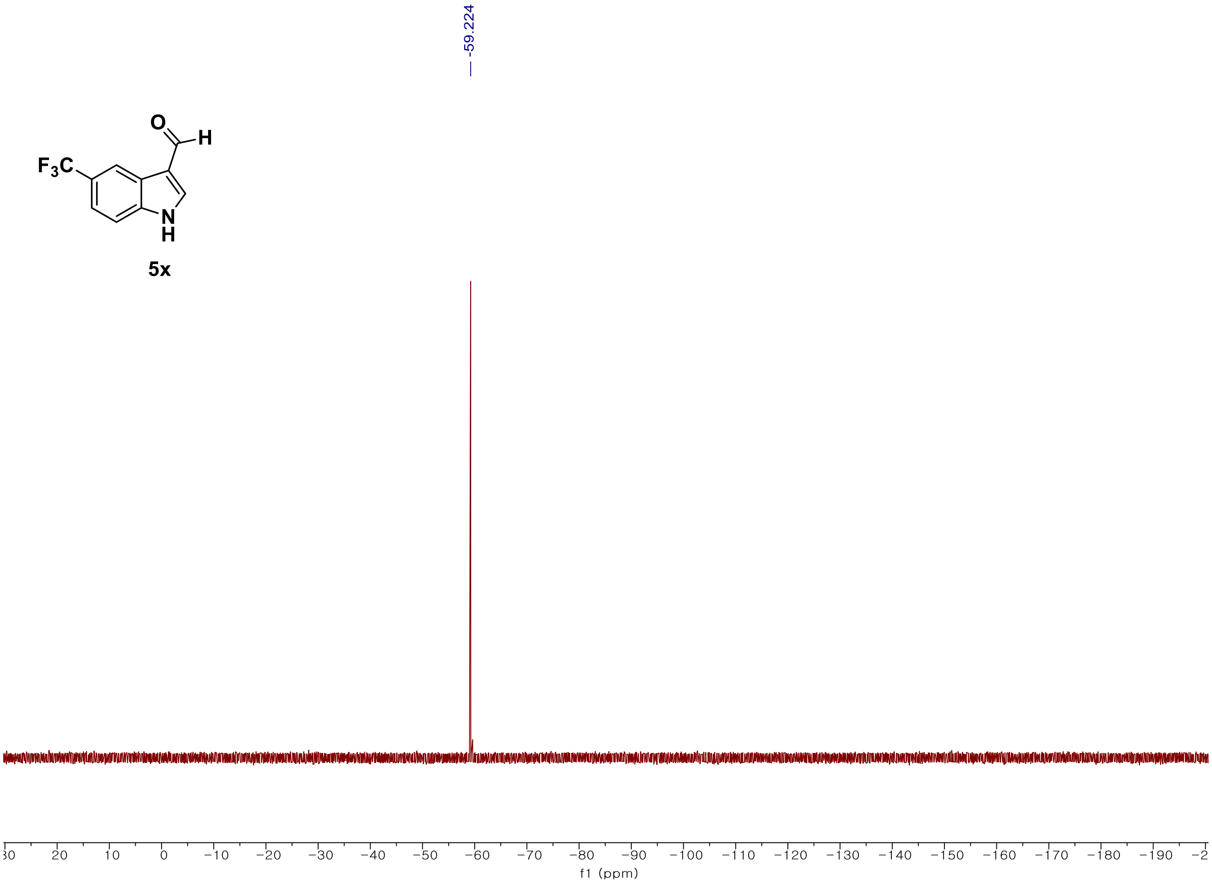
-59.224
O
H
F3C
N
H
5x
30 20 10 0 −10 −20 −30 −40 −50 −60 −70 −80 −90 −100 −110 −120 −130 −140 −150 −160 −170 −180 −190 −2
f1 (ppm)

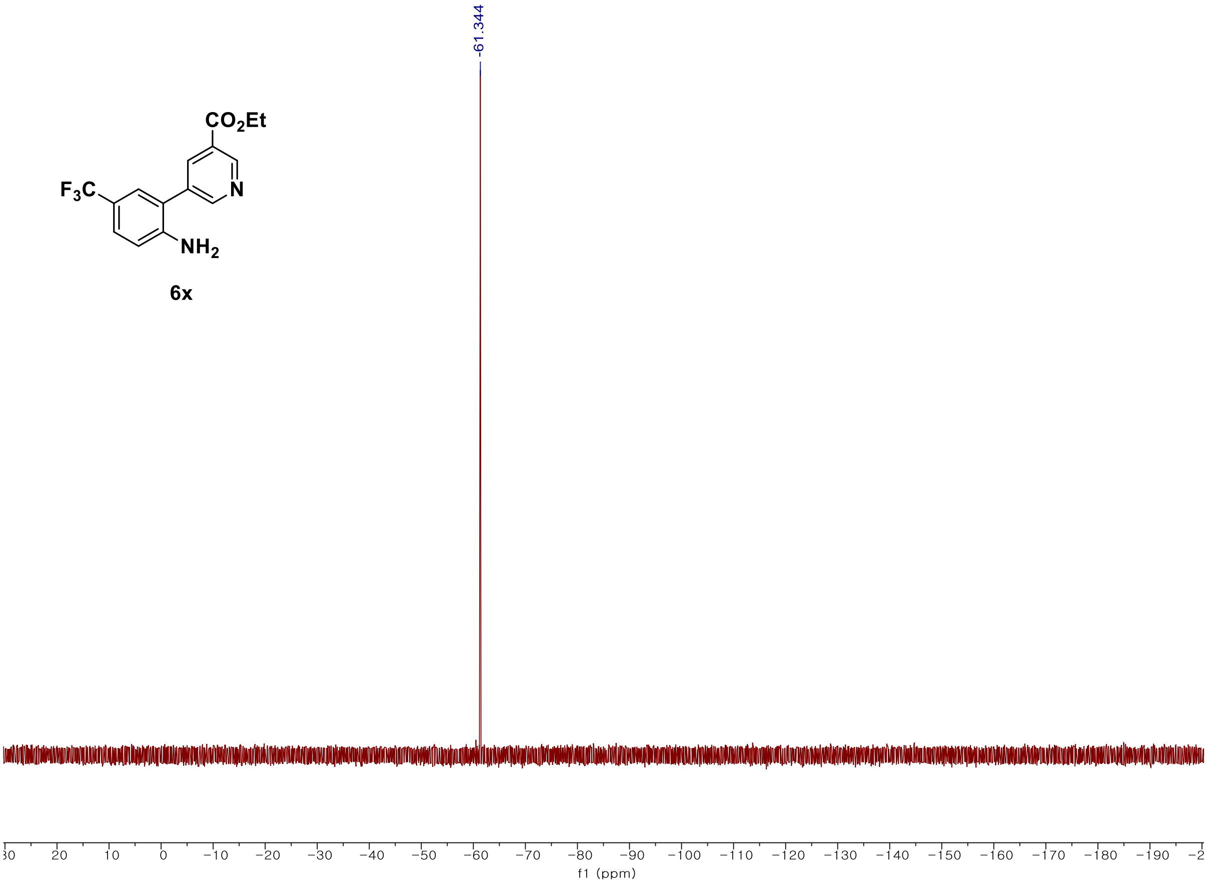
-61.344
CO2Et
F3C
N
NH2
6x
30 20 10 0 −10 −20 −30 −40 −50 −60 −70 −80 −90 −100 −110 −120 −130 −140 −150 −160 −170 −180 −190 −2
f1 (ppm)

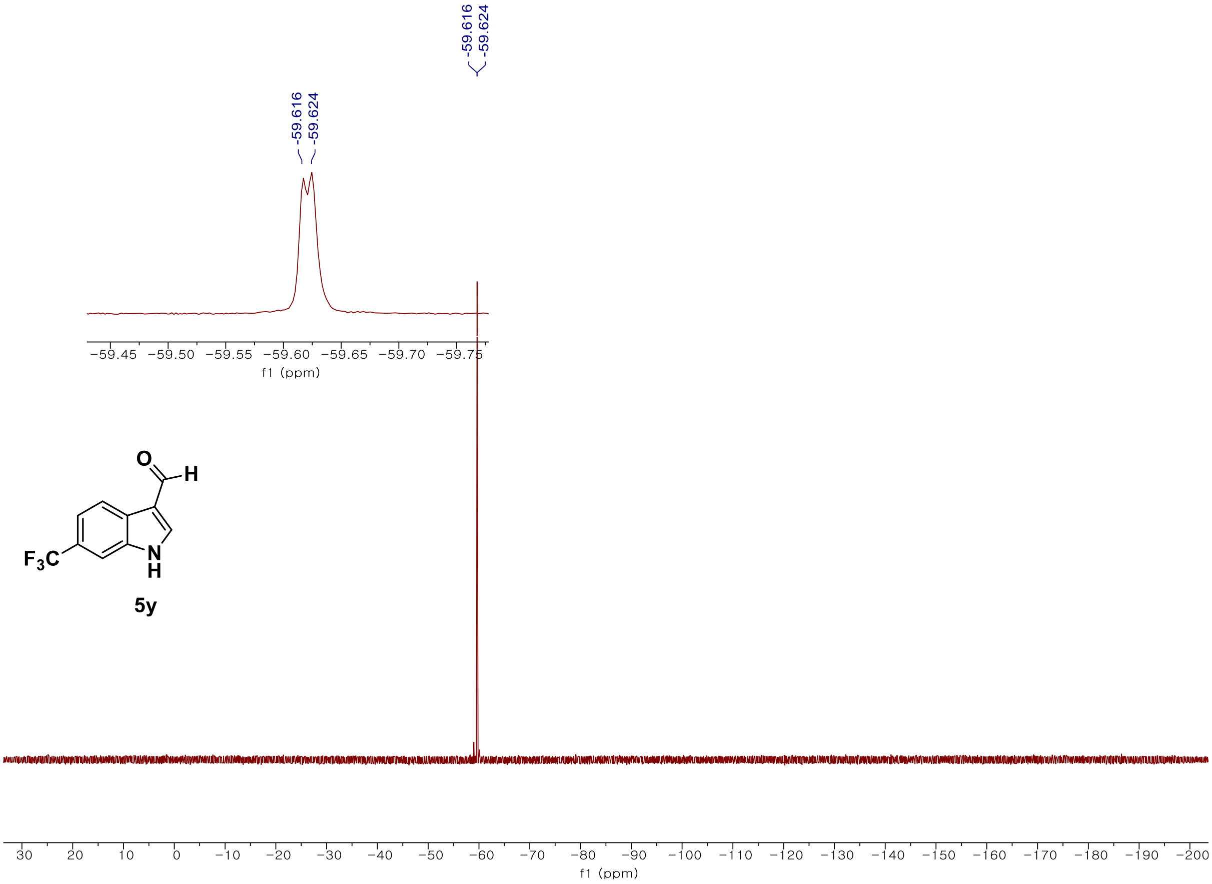

-59.616
-59.624
−59.45 −59.50 −59.55 −59.60 −59.65 −59.70 −59.75
f1 (ppm)
F3C
H
N
O
H
30 20 10 0 −10 −20 −30 −40 −50 −60 −70 −80 −90 −100 −110 −120 −130 −140 −150 −160 −170 −180 −190 −200
f1 (ppm)

5y

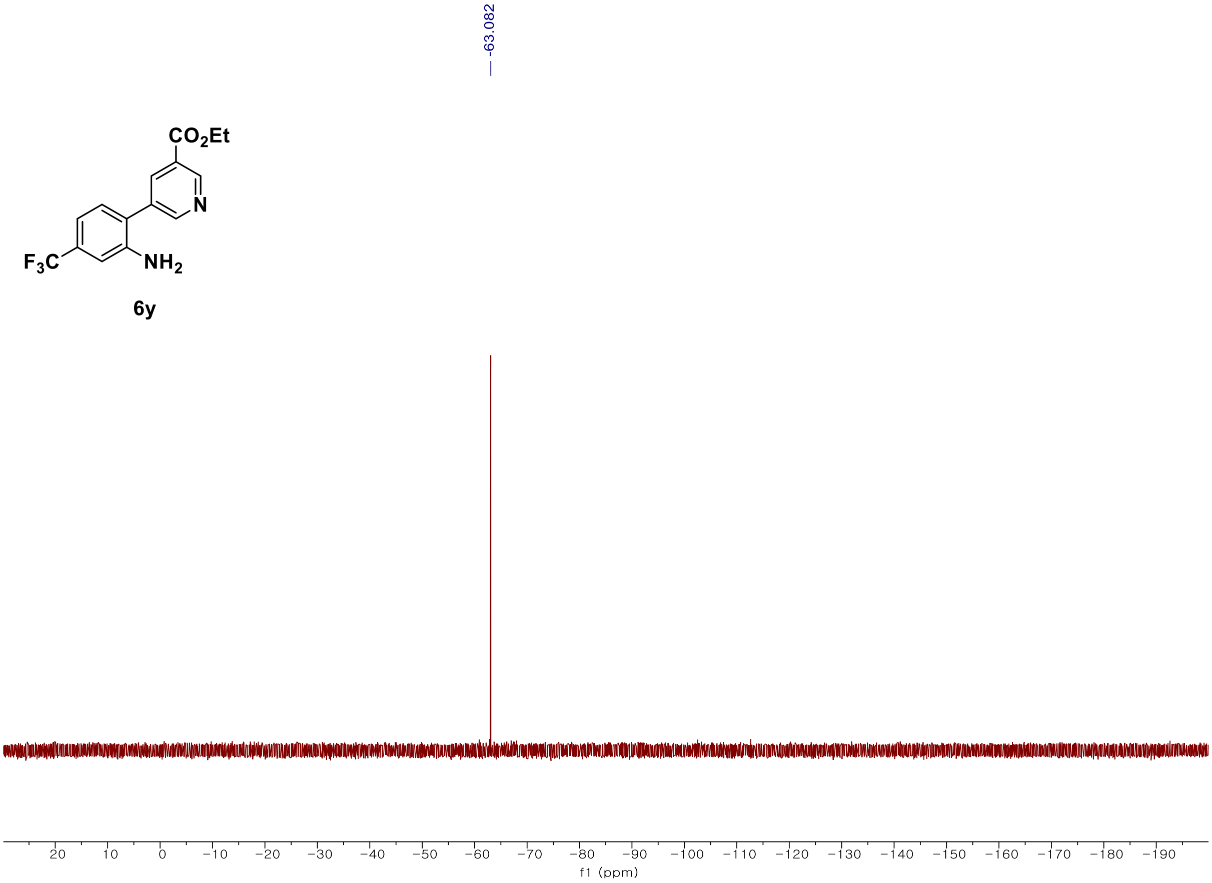

-63.082
CO2Et
N
F3C
NH2
20 10 0 −10 −20 −30 −40 −50 −60 −70 −80 −90 −100 −110 −120 −130 −140 −150 −160 −170 −180 −190
f1 (ppm)

6y

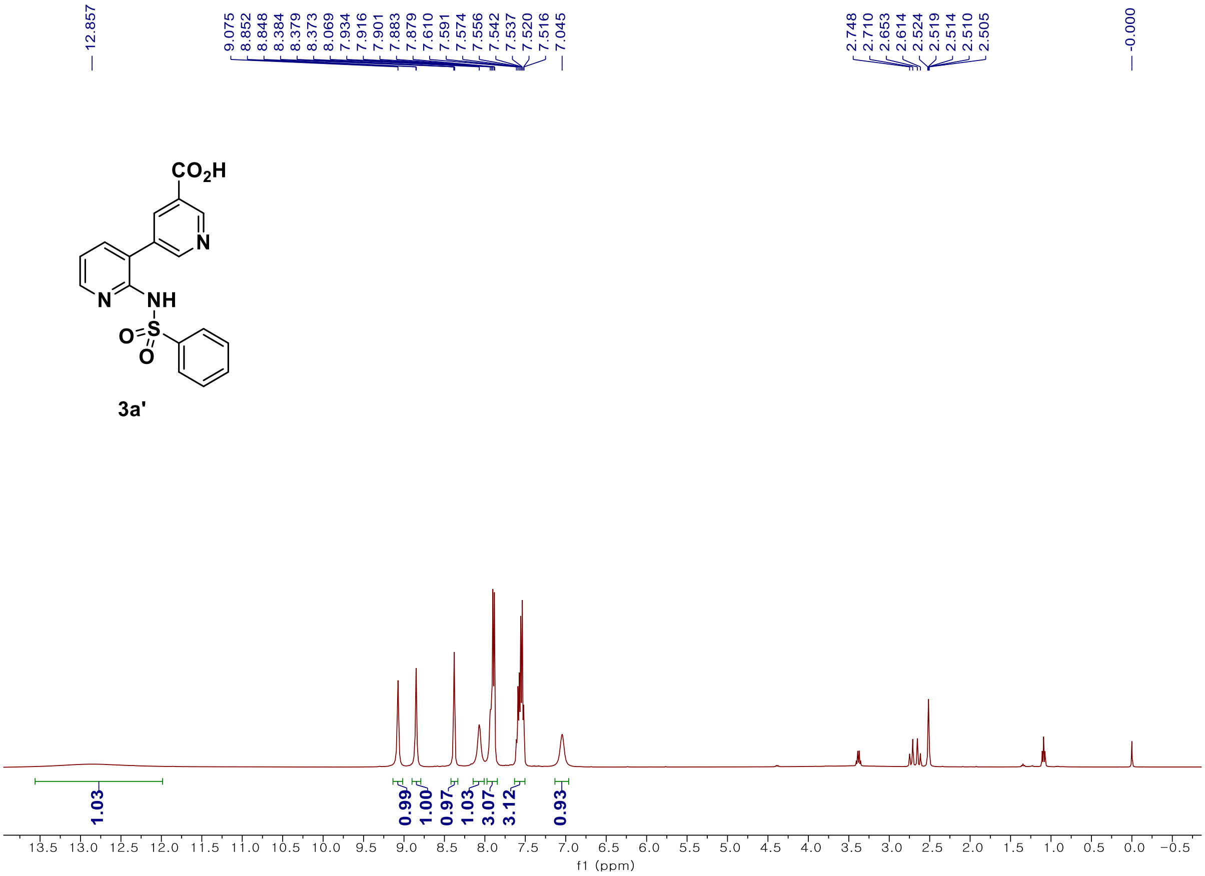
12.857
9.075 8.852 8.848 8.384 8.379 8.373 8.069 7.934 7.916 7.901 7.883 7.879 7.610 7.591 7.574 7.556 7.542 7.537 7.520 7.516 7.045
2.748 2.710 2.653 2.614 2.524 2.519 2.514 2.510 2.505
-0.000
CO2H
N
N
NH
O=S
O
3a'
1.03
0.99 1.00 0.97 1.03 3.07 3.12 0.93
13.5 13.0 12.5 12.0 11.5 11.0 10.5 10.0 9.5 9.0 8.5 8.0 7.5 7.0 6.5 6.0 5.5 5.0 4.5 4.0 3.5 3.0 2.5 2.0 1.5 1.0 0.5 0.0 -0.5
f1 (ppm)

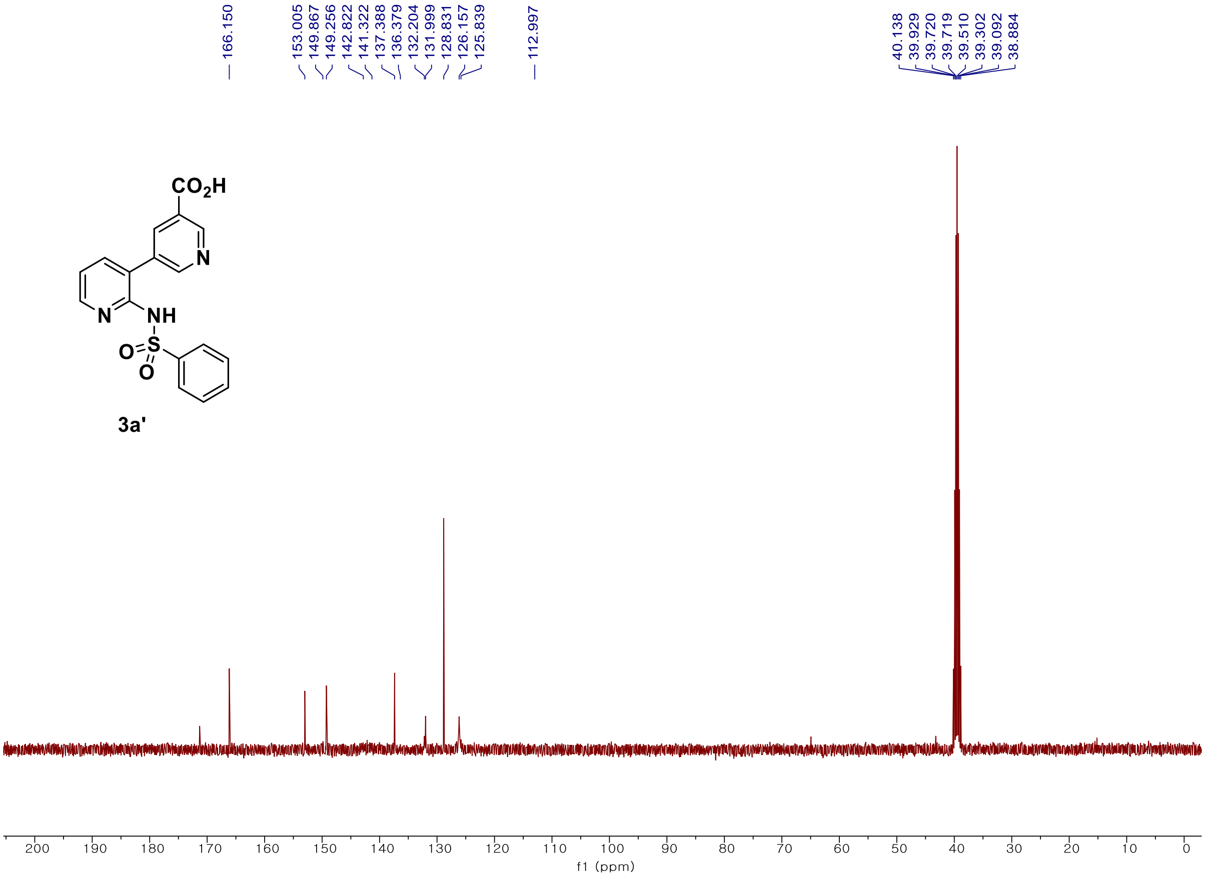
166.150
153.005 149.867 149.256 142.822 141.322 137.388 136.379 132.204 131.999 128.831 126.157 125.839
112.997
40.138 39.929 39.720 39.719 39.510 39.302 39.092 38.884
CO2H
N
N
NH
O=S
O
3a'
200 190 180 170 160 150 140 130 120 110 100 90 80 70 60 50 40 30 20 10 0
f1 (ppm)

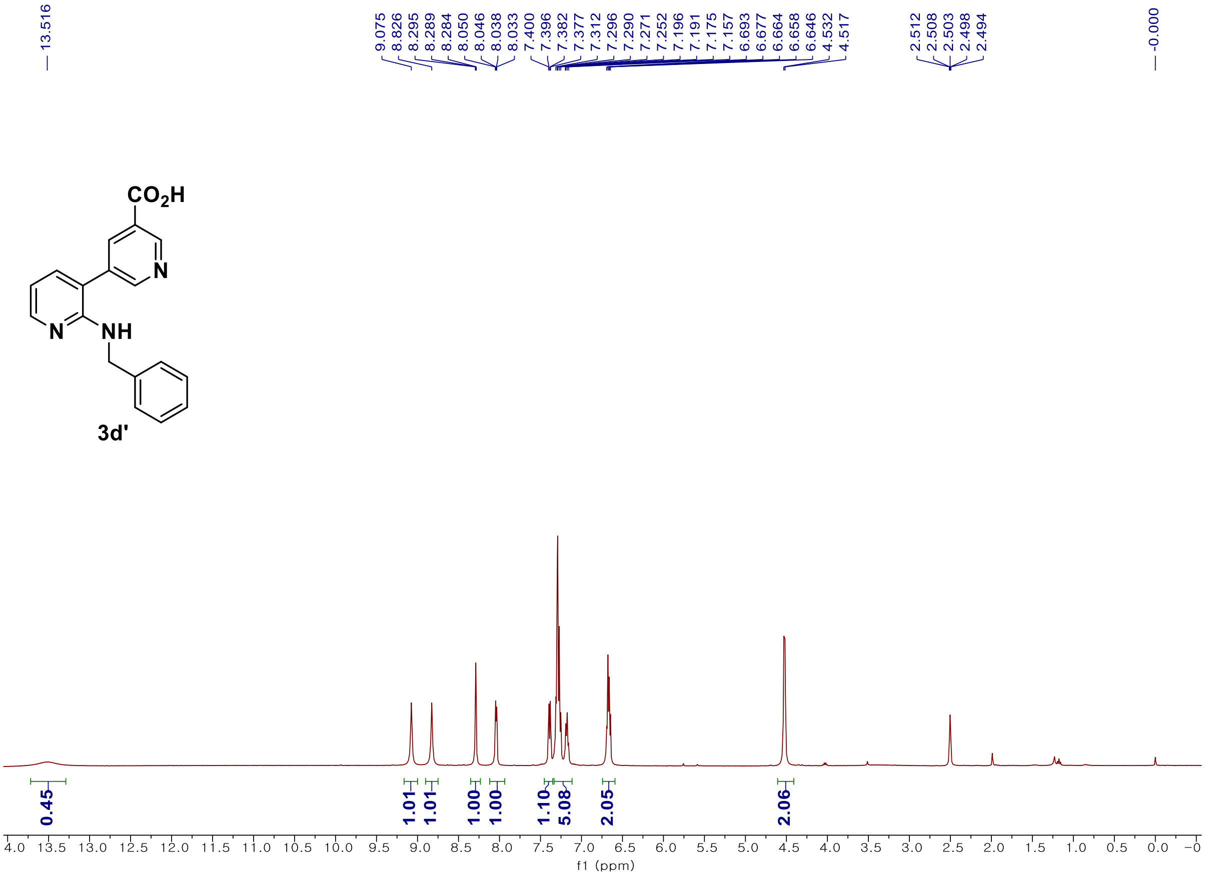
13.516
9.075 8.826 8.295 8.289 8.284 8.050 8.046 8.038 8.033 7.400 7.396 7.382 7.377 7.312 7.296 7.290 7.271 7.252 7.196 7.191 7.175 7.157 6.693 6.677 6.664 6.658 6.646 4.532 4.517
2.512 2.508 2.503 2.498 2.494
-0.000
CO2H
N
N
NH
3d'
0.45
1.01
1.01
1.00
1.00
1.10
5.08
2.05
2.06
f1 (ppm)

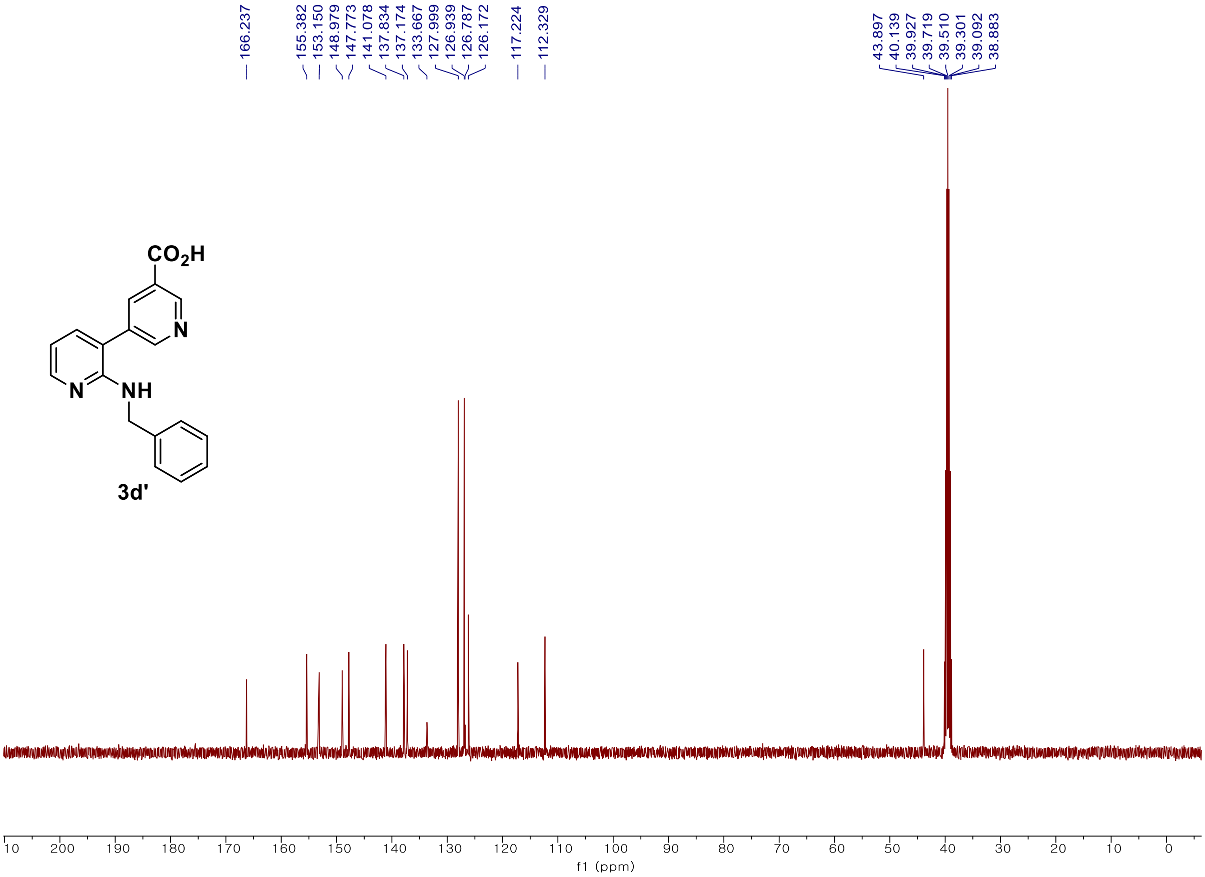
166.237
155.382 153.150 148.979 147.773 141.078 137.834 137.174 133.667 127.999 126.939 126.787 126.172
117.224
112.329
43.897 40.139 39.927 39.719 39.510 39.301 39.092 38.883
CO2H
N
N
NH
3d'
f1 (ppm)

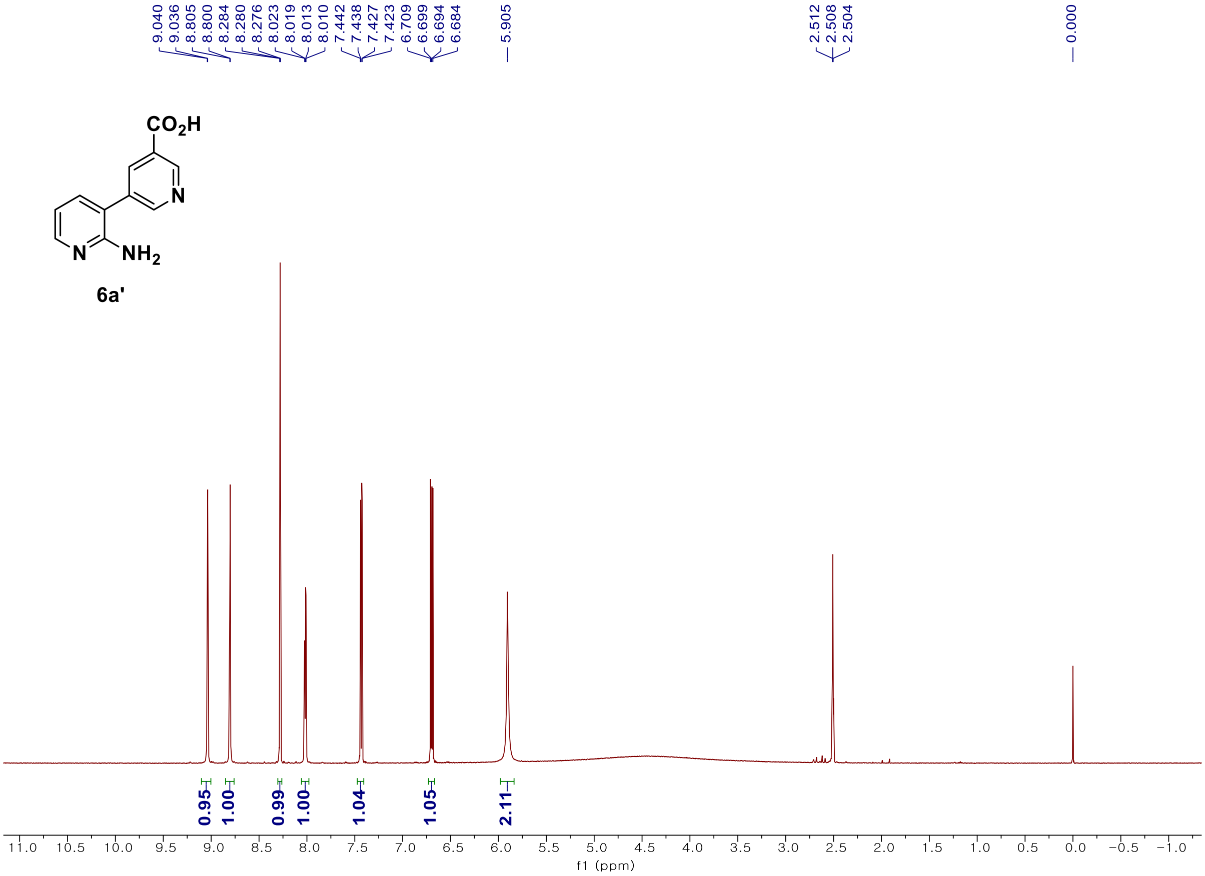

9.040
9.036
8.805
8.800
8.284
8.280
8.276
8.023
8.019
8.013
8.010
7.442
7.438
7.427
7.423
6.709
6.699
6.694
6.684
5.905
2.512
2.508
2.504
0.000
CO2H
N
N
NH2
6a'
0.95
1.00
0.99
1.00
1.04
1.05
2.11
f1 (ppm)

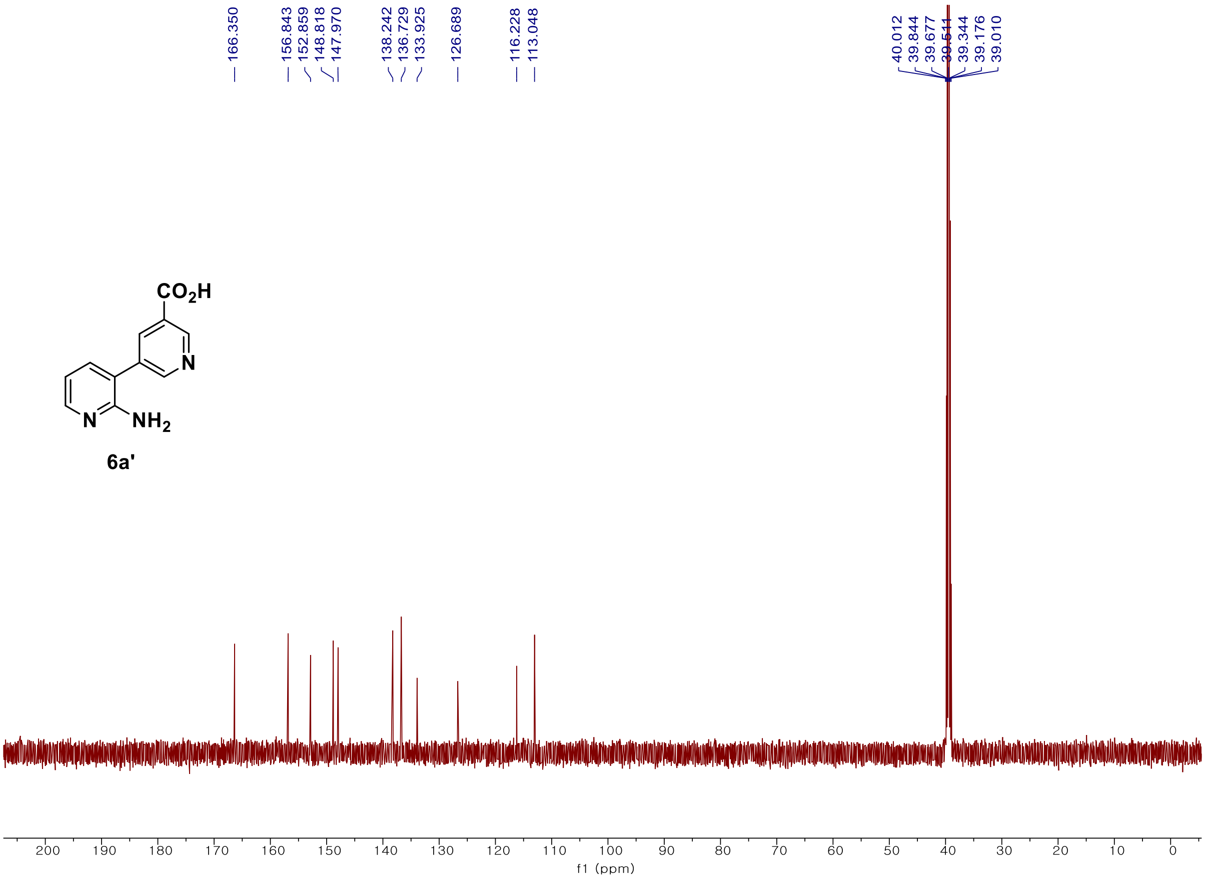

166.350
156.843
152.859
148.818
147.970
138.242
136.729
133.925
126.689
116.228
113.048
40.012
39.844
39.677
39.511
39.344
39.176
39.010
CO2H
N
N
NH2
6a'
f1 (ppm)

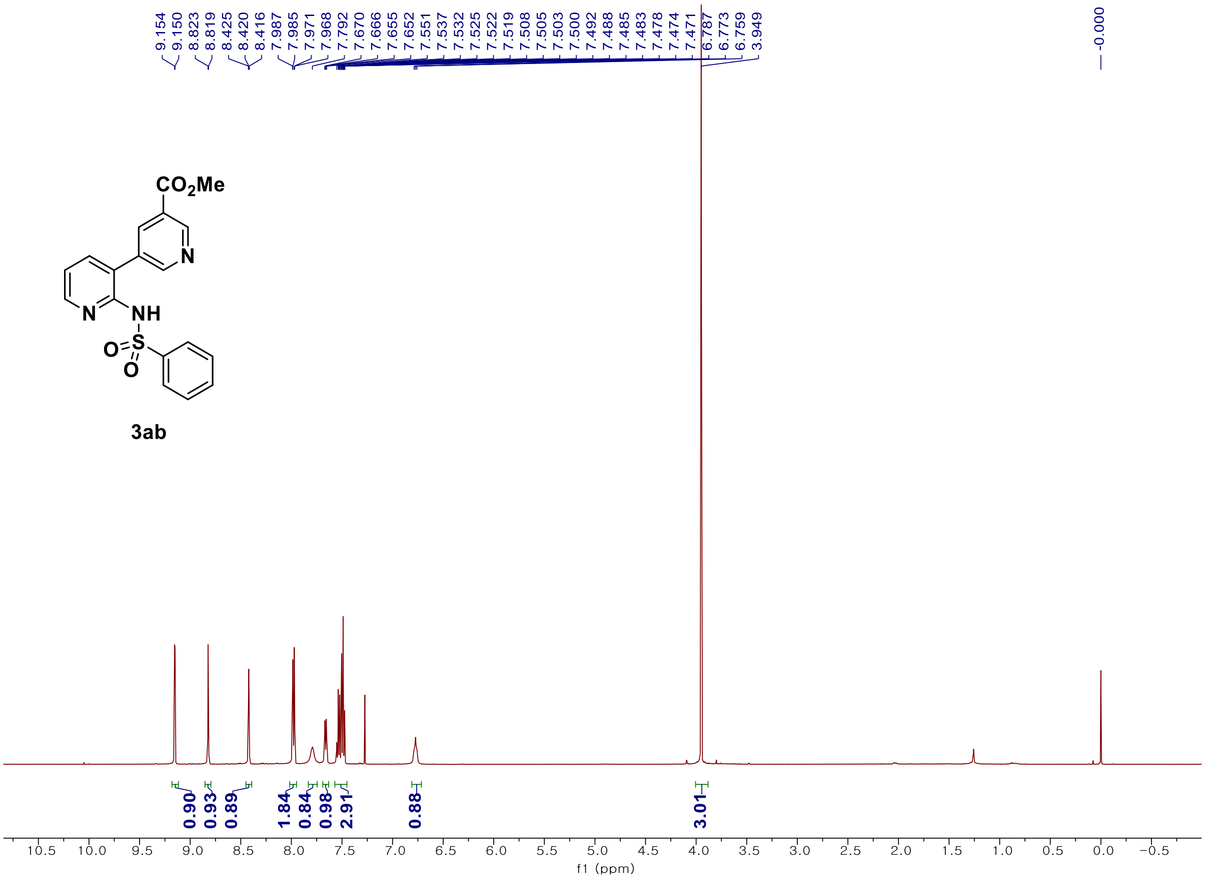
9.154
9.150
8.823
8.819
8.425
8.420
8.416
7.987
7.985
7.971
7.968
7.792
7.670
7.666
7.655
7.652
7.551
7.537
7.532
7.525
7.522
7.519
7.508
7.505
7.503
7.500
7.492
7.488
7.485
7.483
7.478
7.474
7.471
6.787
6.773
6.759
3.949
-0.000
CO2Me
N
N
NH
O=S
O
3ab
0.90
0.93
0.89
1.84
0.84
0.98
2.91
0.88
3.01
f1 (ppm)

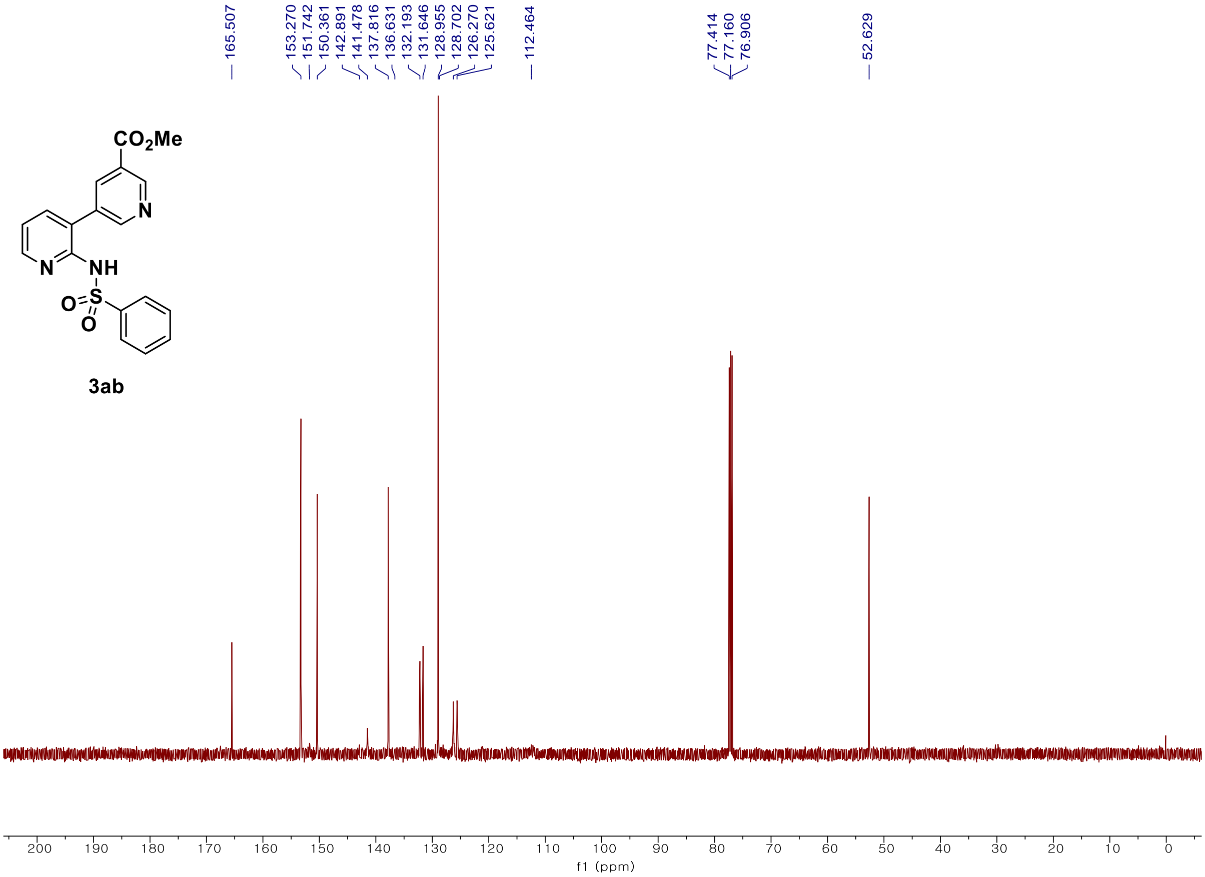
165.507
153.270
151.742
150.361
142.891
141.478
137.816
136.631
132.193
131.646
128.955
128.702
126.270
125.621
112.464
77.414
77.160
76.906
52.629
CO2Me
N
N
NH
O=S
O
3ab
f1 (ppm)

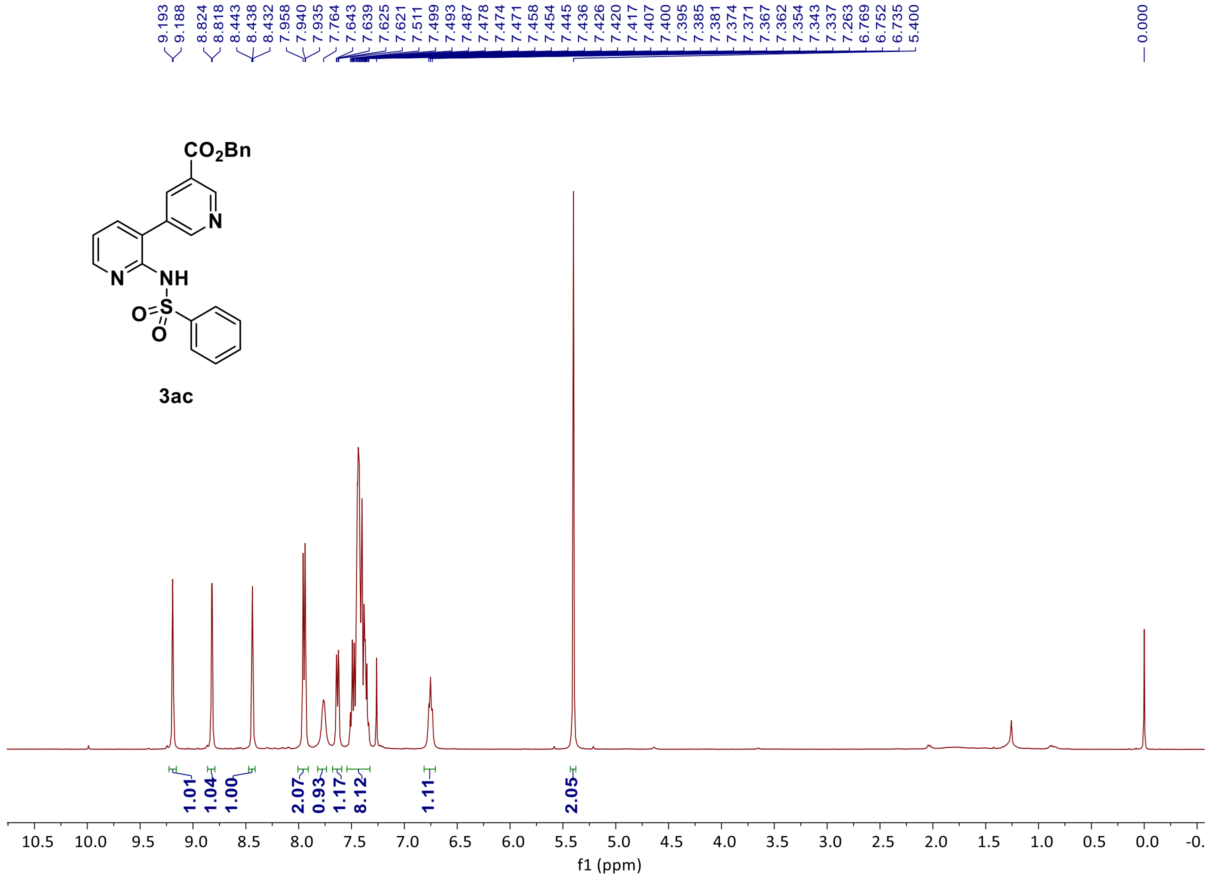
9.193
9.188
8.824
8.818
8.443
8.438
8.432
7.958
7.940
7.935
7.764
7.643
7.639
7.625
7.621
7.511
7.499
7.493
7.487
7.478
7.474
7.471
7.458
7.454
7.445
7.436
7.426
7.420
7.417
7.407
7.400
7.395
7.385
7.381
7.374
7.371
7.367
7.362
7.354
7.343
7.337
7.263
6.769
6.752
6.735
5.400
0.000
CO_2Bn
N
NH
O=S
O
3ac
1.01
1.04
1.00
2.07
0.93
1.17
8.12
1.11
2.05
10.5 10.0 9.5 9.0 8.5 8.0 7.5 7.0 6.5 6.0 5.5 5.0 4.5 4.0 3.5 3.0 2.5 2.0 1.5 1.0 0.5 0.0 -0.
f1 (ppm)

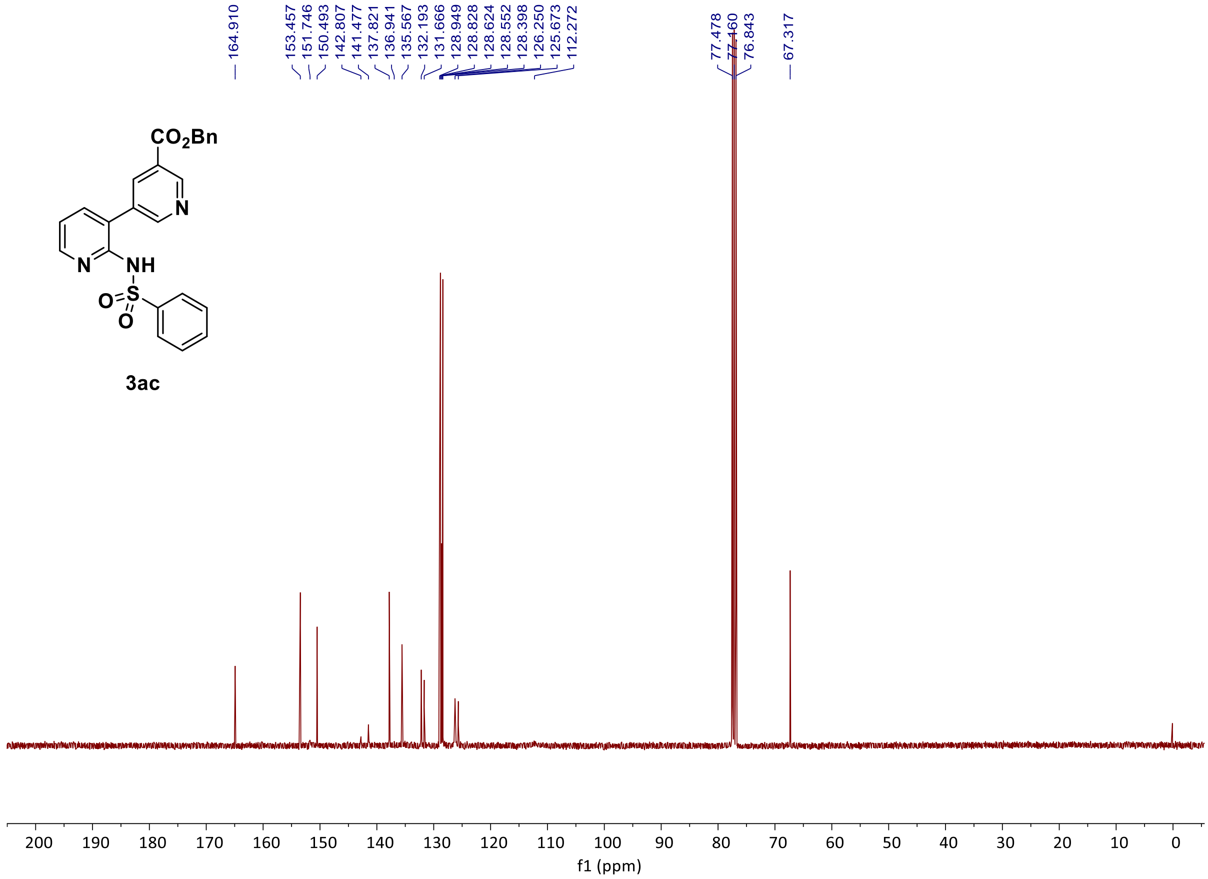
164.910
153.457
151.746
150.493
142.807
141.477
137.821
136.941
135.567
132.193
131.666
128.949
128.828
128.624
128.552
128.398
126.250
125.673
112.272
77.478
77.160
76.843
67.317
CO_2Bn
N
NH
O=S
O
3ac
200 190 180 170 160 150 140 130 120 110 100 90 80 70 60 50 40 30 20 10 0
f1 (ppm)

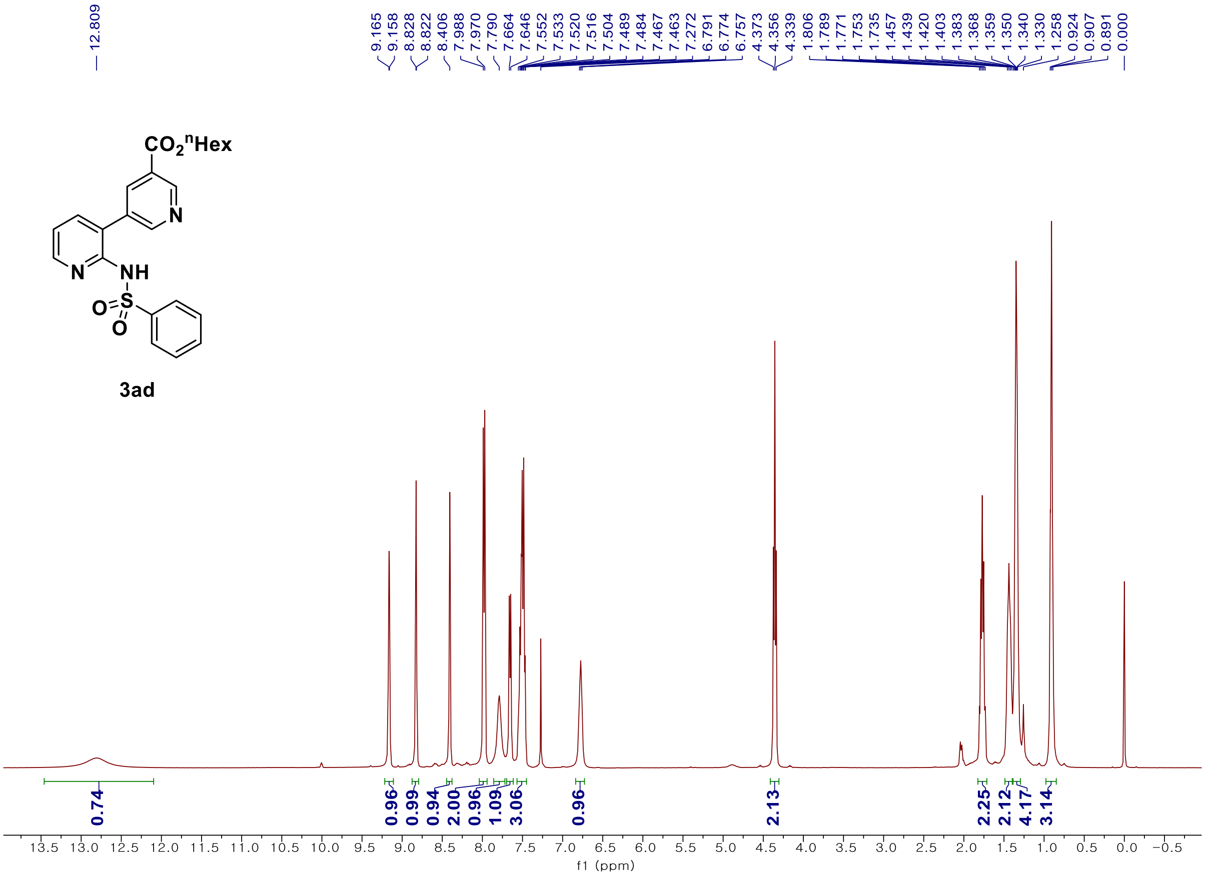

12.809
9.165
9.158
8.828
8.822
8.406
7.988
7.970
7.790
7.664
7.646
7.552
7.533
7.520
7.516
7.504
7.489
7.484
7.467
7.463
7.272
6.791
6.774
6.757
4.373
4.356
4.339
1.806
1.789
1.771
1.753
1.735
1.457
1.439
1.420
1.403
1.383
1.368
1.359
1.350
1.340
1.330
1.258
0.924
0.907
0.891
0.000
$CO_2{}^nHex$
N
NH
O=S
O
3ad
0.74
0.96
0.99
0.94
2.00
0.96
1.09
3.06
0.96
2.13
2.25
2.12
4.17
3.14
f1 (ppm)

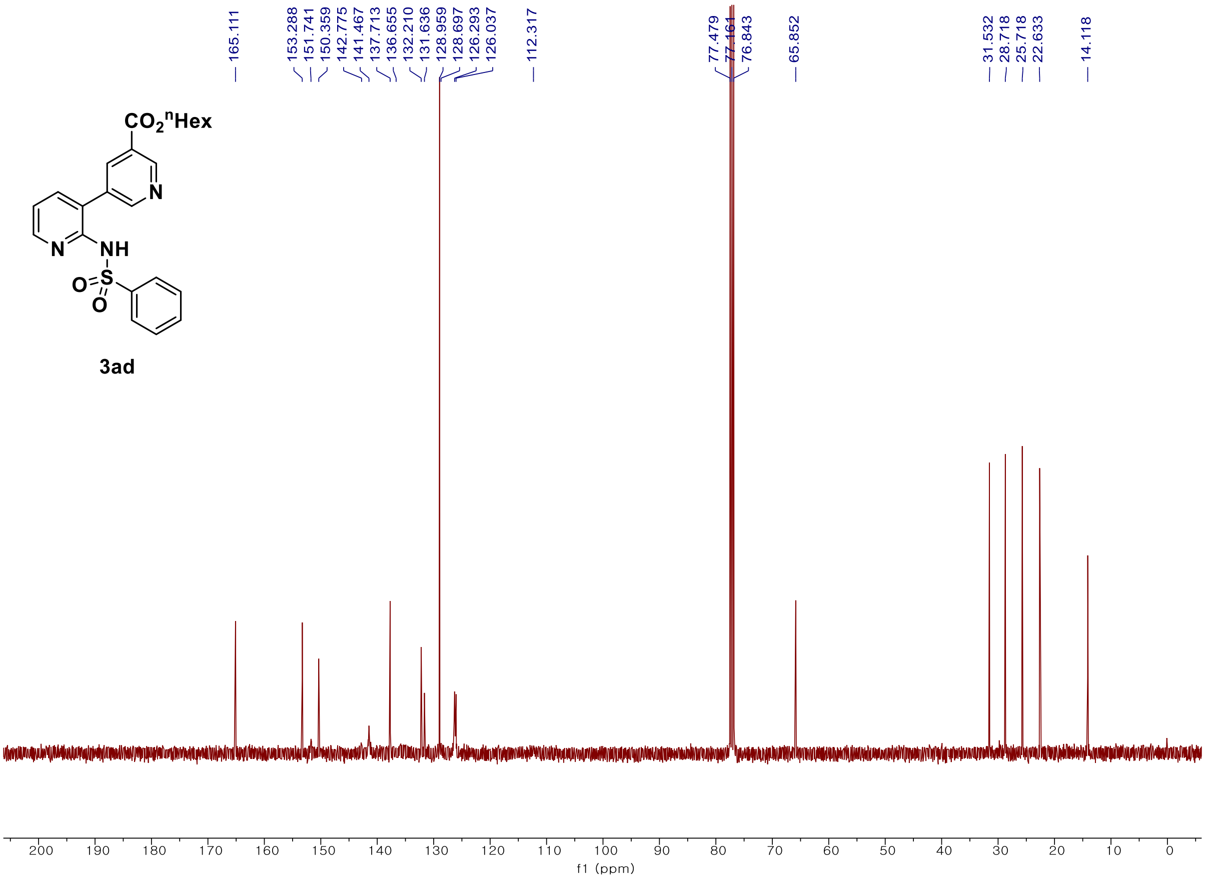

165.111
153.288
151.741
150.359
142.775
141.467
137.713
136.655
132.210
131.636
128.959
128.697
126.293
126.037
112.317
77.479
77.161
76.843
65.852
31.532
28.718
25.718
22.633
14.118
$CO_2{}^nHex$
N
NH
O=S
O
3ad
f1 (ppm)

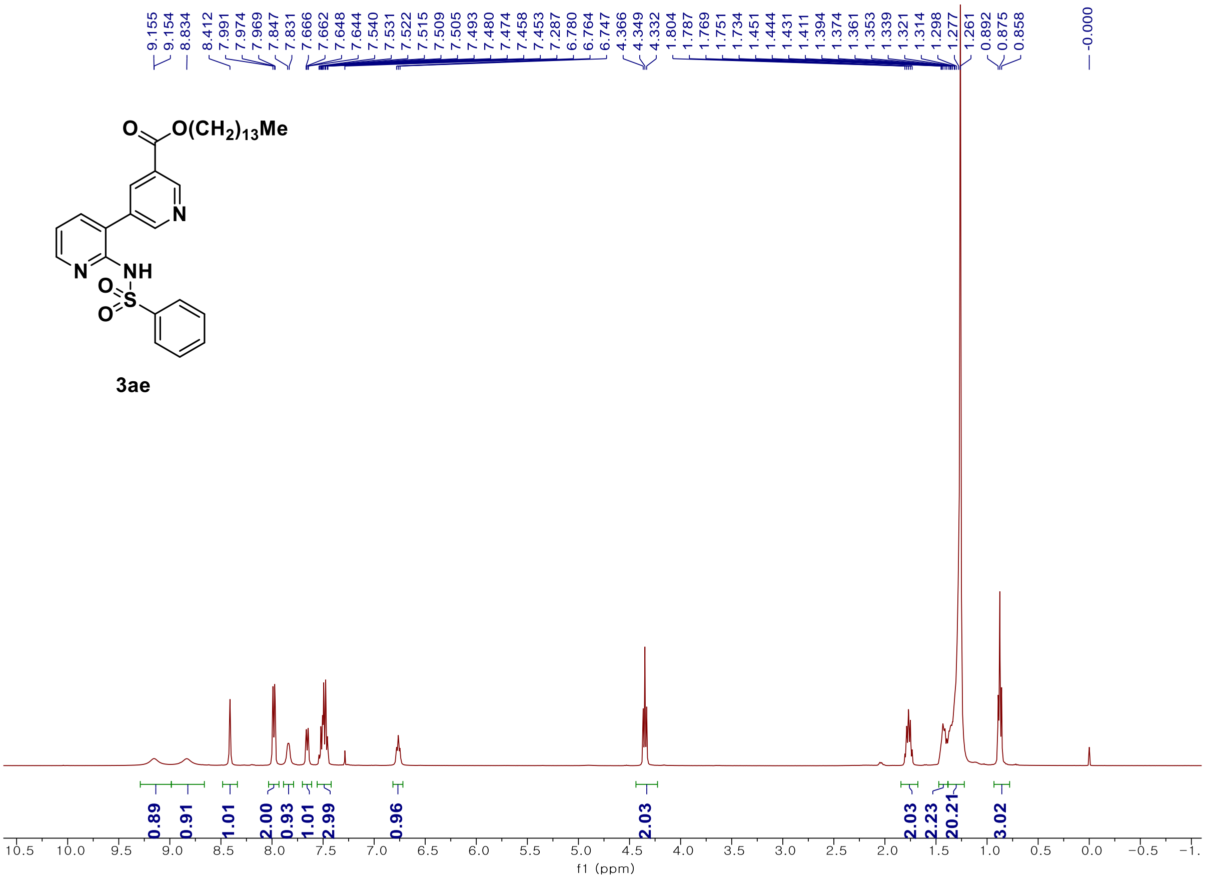
9.155
9.154
8.834
8.412
7.991
7.974
7.969
7.847
7.831
7.666
7.662
7.648
7.644
7.540
7.531
7.522
7.515
7.509
7.505
7.493
7.480
7.474
7.458
7.453
7.287
6.780
6.764
6.747
4.366
4.349
4.332
1.804
1.787
1.769
1.751
1.734
1.451
1.444
1.431
1.411
1.394
1.374
1.361
1.353
1.339
1.321
1.314
1.298
1.277
1.261
0.892
0.875
0.858
-0.000
O(CH2)13Me
3ae
0.89
0.91
1.01
2.00
0.93
1.01
2.99
0.96
2.03
2.03
2.23
20.21
3.02
f1 (ppm)

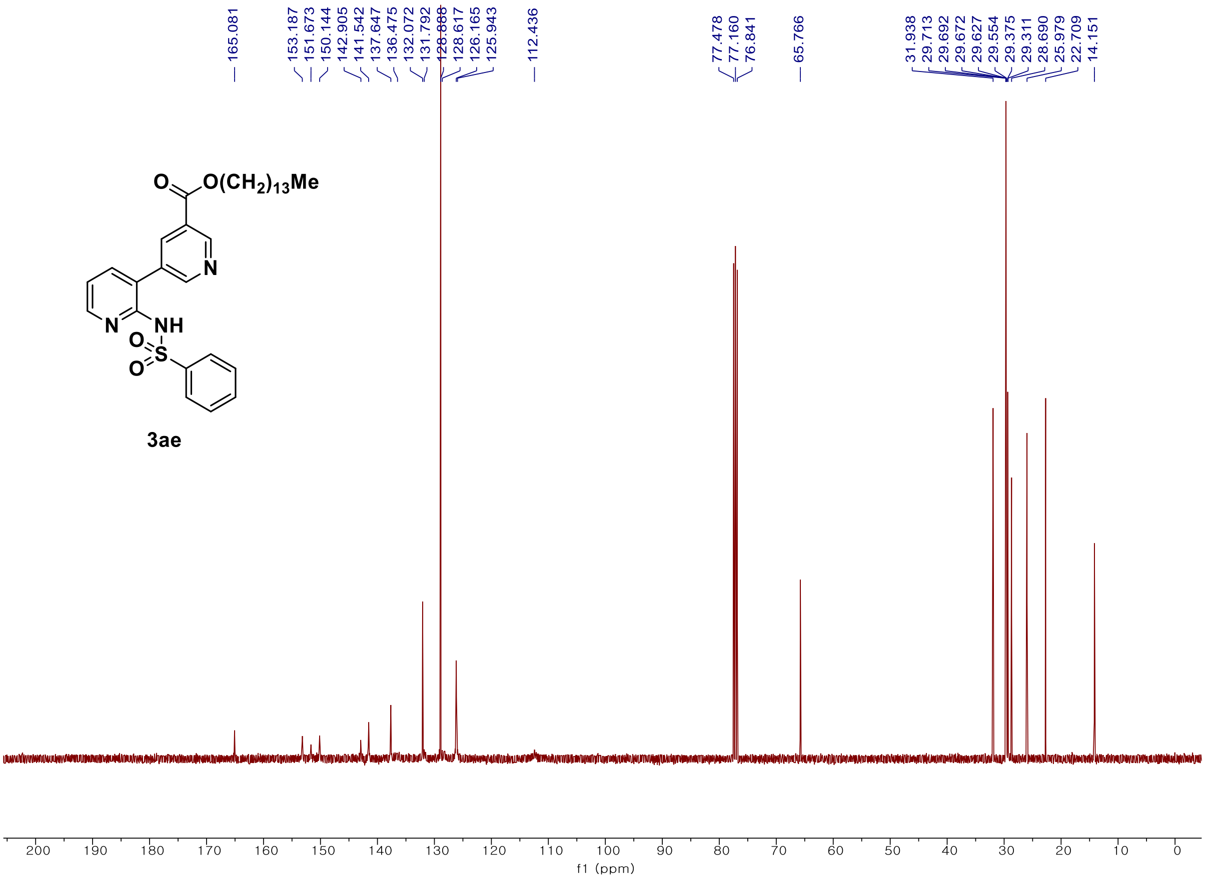
165.081
153.187
151.673
150.144
142.905
141.542
137.647
136.475
132.072
131.792
128.886
128.617
126.165
125.943
112.436
77.478
77.160
76.841
65.766
31.938
29.713
29.692
29.672
29.627
29.554
29.375
29.311
28.690
25.979
22.709
14.151
O(CH2)13Me
3ae
f1 (ppm)

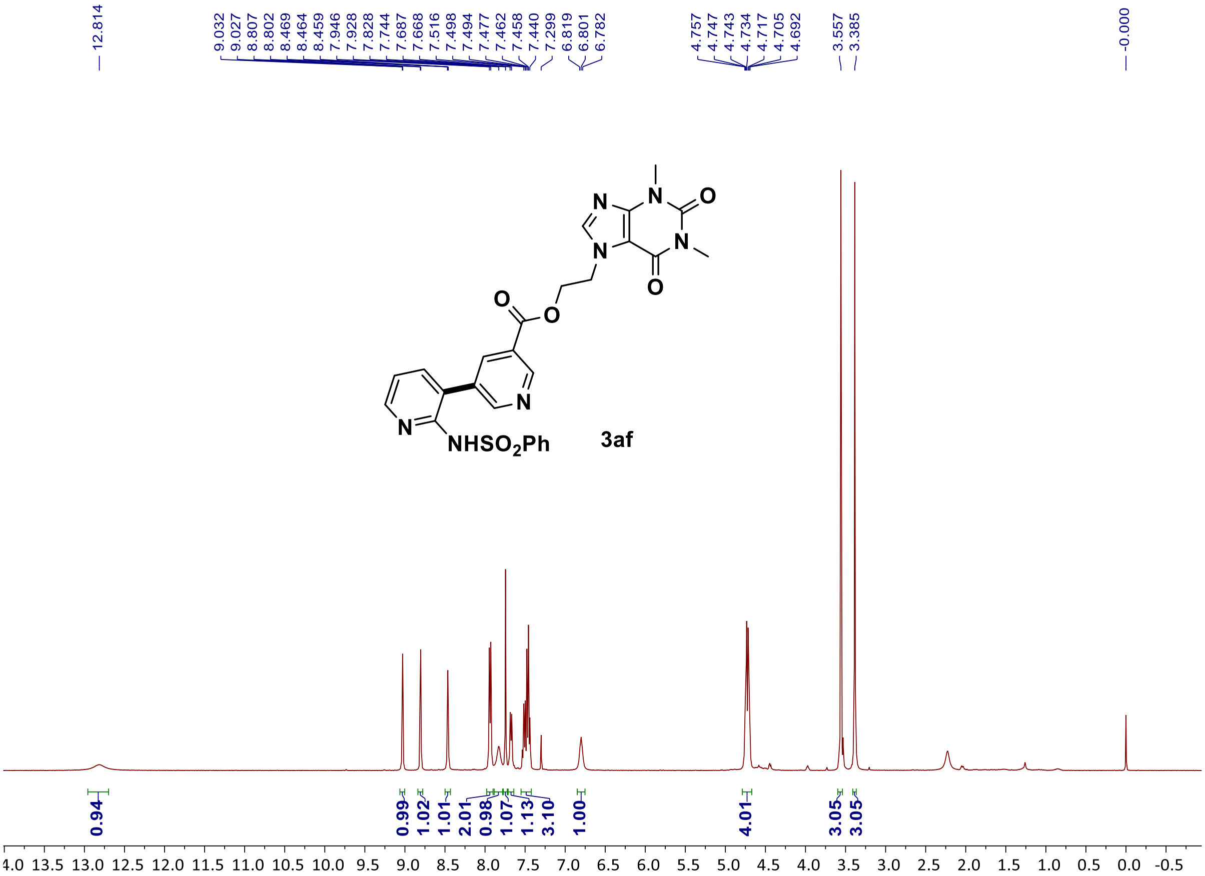
12.814
9.032
9.027
8.807
8.802
8.469
8.464
8.459
7.946
7.928
7.828
7.744
7.687
7.668
7.516
7.498
7.494
7.477
7.462
7.458
7.440
7.299
6.819
6.801
6.782
4.757
4.747
4.743
4.734
4.717
4.705
4.692
3.557
3.385
-0.000
N
N
N
N
O
O
O
O
N
N
NHSO2Ph
3af
0.94
0.99
1.02
1.01
2.01
0.98
1.07
1.13
3.10
1.00
4.01
3.05
3.05
4.0 13.5 13.0 12.5 12.0 11.5 11.0 10.5 10.0 9.5 9.0 8.5 8.0 7.5 7.0 6.5 6.0 5.5 5.0 4.5 4.0 3.5 3.0 2.5 2.0 1.5 1.0 0.5 0.0 -0.5

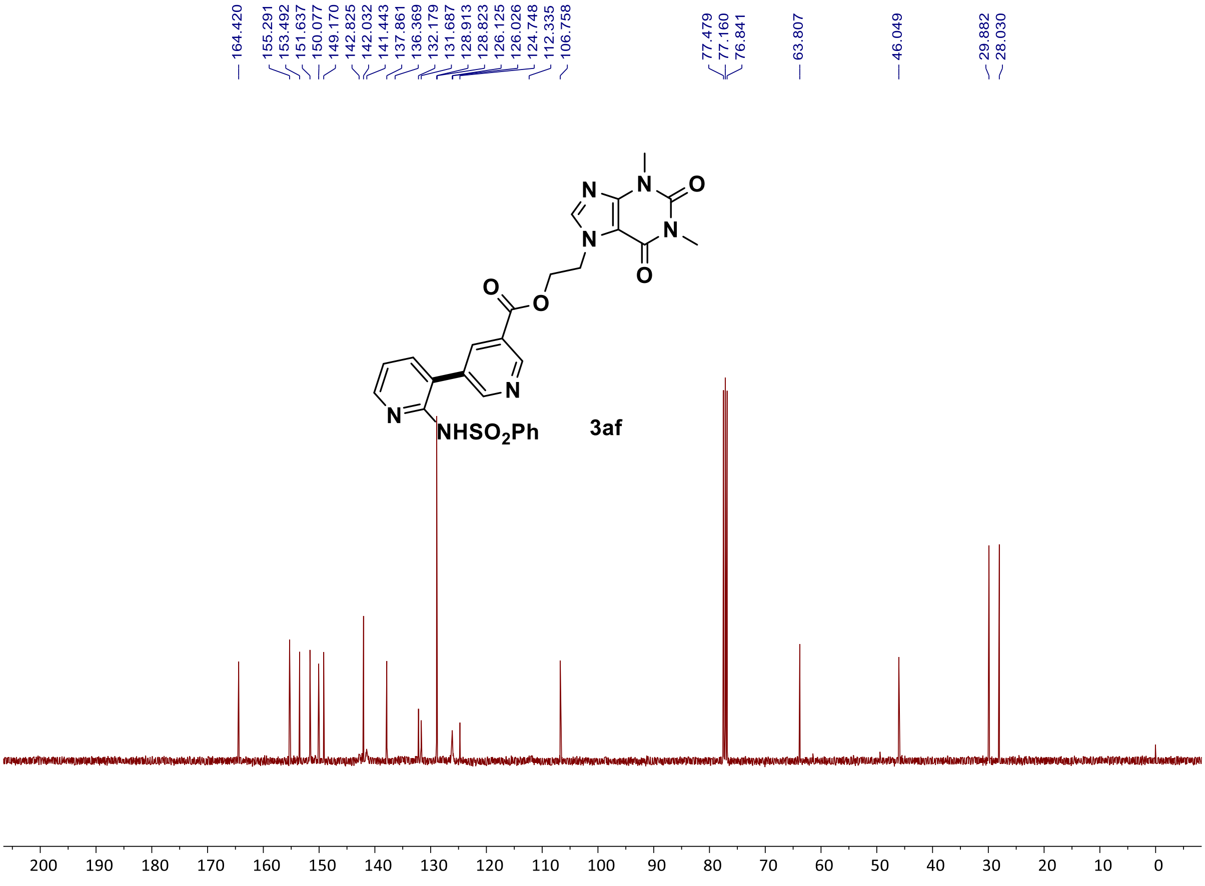
164.420
155.291
153.492
151.637
150.077
149.170
142.825
142.032
141.443
137.861
136.369
132.179
131.687
128.913
128.823
126.125
126.026
124.748
112.335
106.758
77.479
77.160
76.841
63.807
46.049
29.882
28.030
N
N
N
N
O
O
O
O
N
N
NHSO2Ph
3af
200 190 180 170 160 150 140 130 120 110 100 90 80 70 60 50 40 30 20 10 0

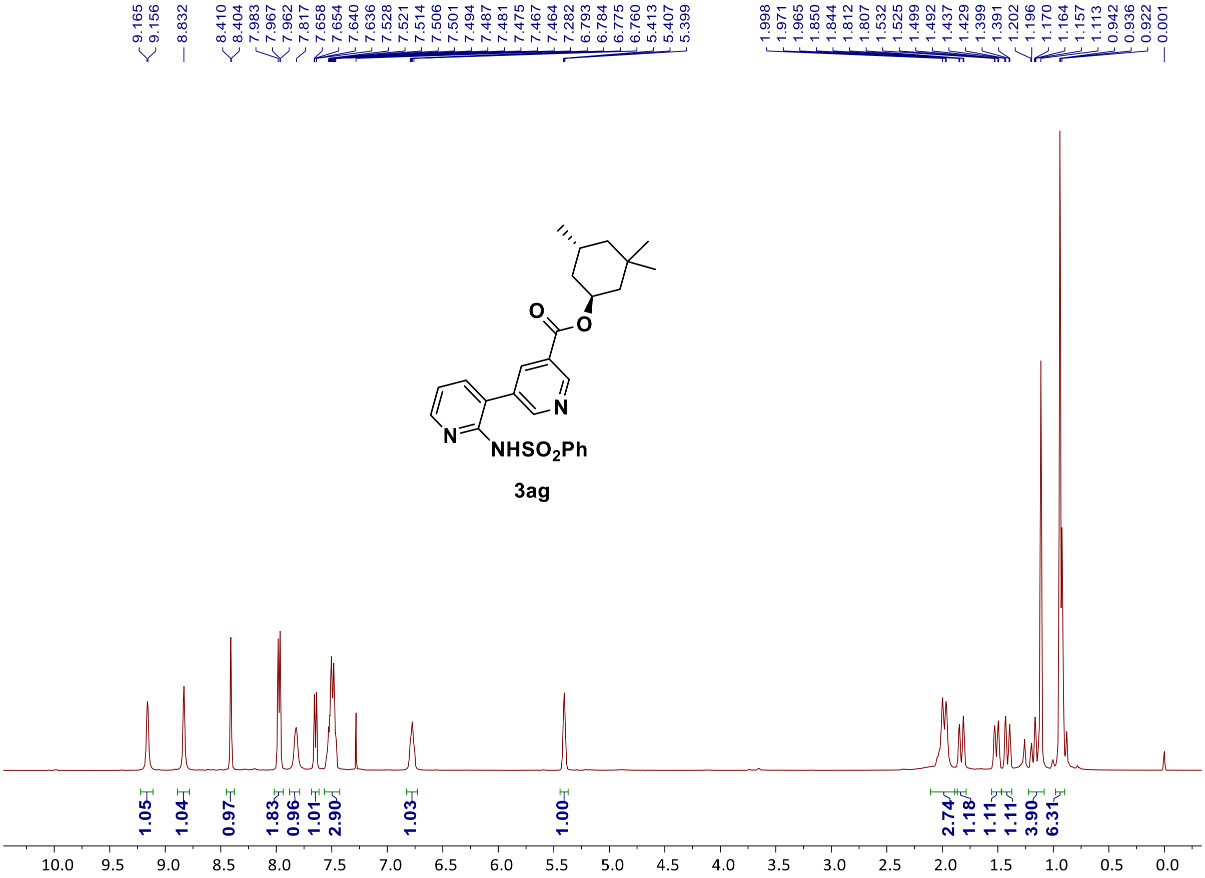
9.165
9.156
8.832
8.410
8.404
7.983
7.967
7.962
7.817
7.658
7.654
7.640
7.636
7.528
7.521
7.514
7.506
7.501
7.494
7.487
7.481
7.475
7.467
7.464
7.282
6.793
6.784
6.775
6.760
5.413
5.407
5.399
1.998
1.971
1.965
1.850
1.844
1.812
1.807
1.532
1.525
1.499
1.492
1.437
1.429
1.399
1.391
1.202
1.196
1.170
1.164
1.157
1.113
0.942
0.936
0.922
0.001
O
O
N
N
NHSO2Ph
3ag
1.05
1.04
0.97
1.83
0.96
1.01
2.90
1.03
1.00
2.74
1.18
1.11
1.11
3.90
6.31
10.0 9.5 9.0 8.5 8.0 7.5 7.0 6.5 6.0 5.5 5.0 4.5 4.0 3.5 3.0 2.5 2.0 1.5 1.0 0.5 0.0

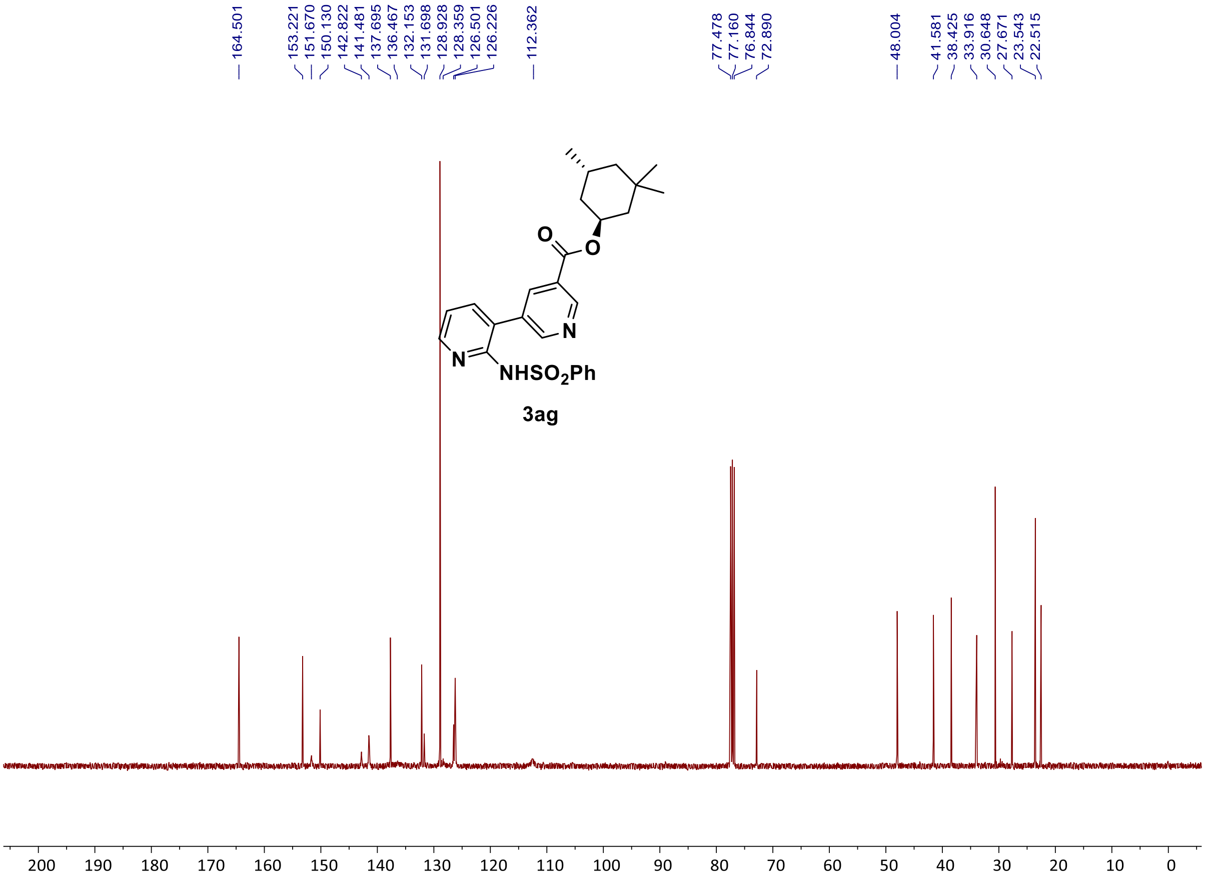
164.501
153.221
151.670
150.130
142.822
141.481
137.695
136.467
132.153
131.698
128.928
128.359
126.501
126.226
112.362
77.478
77.160
76.844
72.890
48.004
41.581
38.425
33.916
30.648
27.671
23.543
22.515
O
O
N
N
NHSO2Ph
3ag
200 190 180 170 160 150 140 130 120 110 100 90 80 70 60 50 40 30 20 10 0

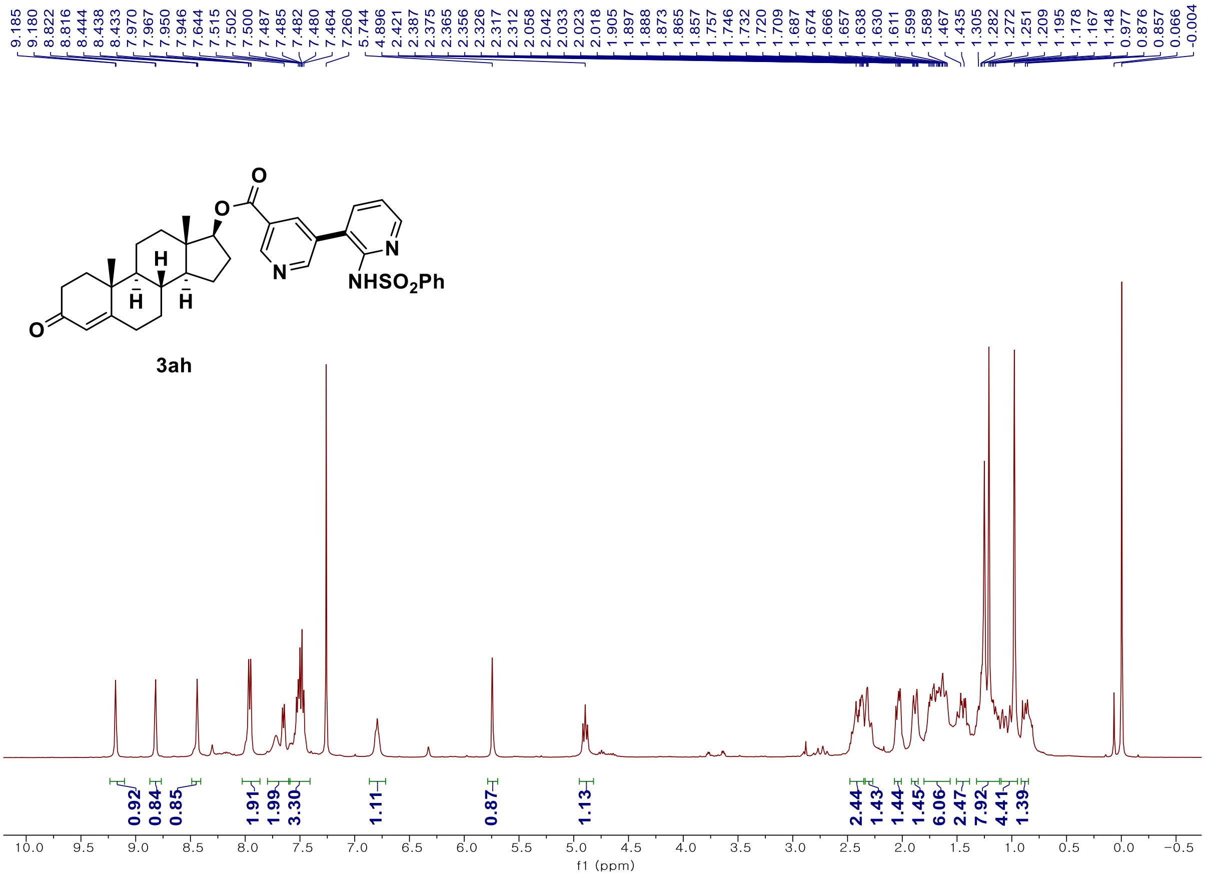
9.185 9.180 8.822 8.816 8.444 8.438 8.433 7.970 7.967 7.950 7.946 7.644 7.515 7.502 7.500 7.487 7.485 7.482 7.480 7.464 7.260 5.744 4.896 2.421 2.387 2.375 2.365 2.356 2.326 2.317 2.312 2.058 2.042 2.033 2.023 2.018 1.905 1.897 1.888 1.873 1.865 1.857 1.757 1.746 1.732 1.720 1.709 1.687 1.674 1.666 1.657 1.638 1.630 1.611 1.599 1.589 1.467 1.435 1.305 1.282 1.272 1.251 1.209 1.195 1.178 1.167 1.148 0.977 0.876 0.857 0.066 -0.004
NHSO2Ph
3ah
0.92 0.84 0.85 1.91 1.99 3.30 1.11 0.87 1.13 2.44 1.43 1.44 1.45 6.06 2.47 7.92 4.41 1.39
f1 (ppm)

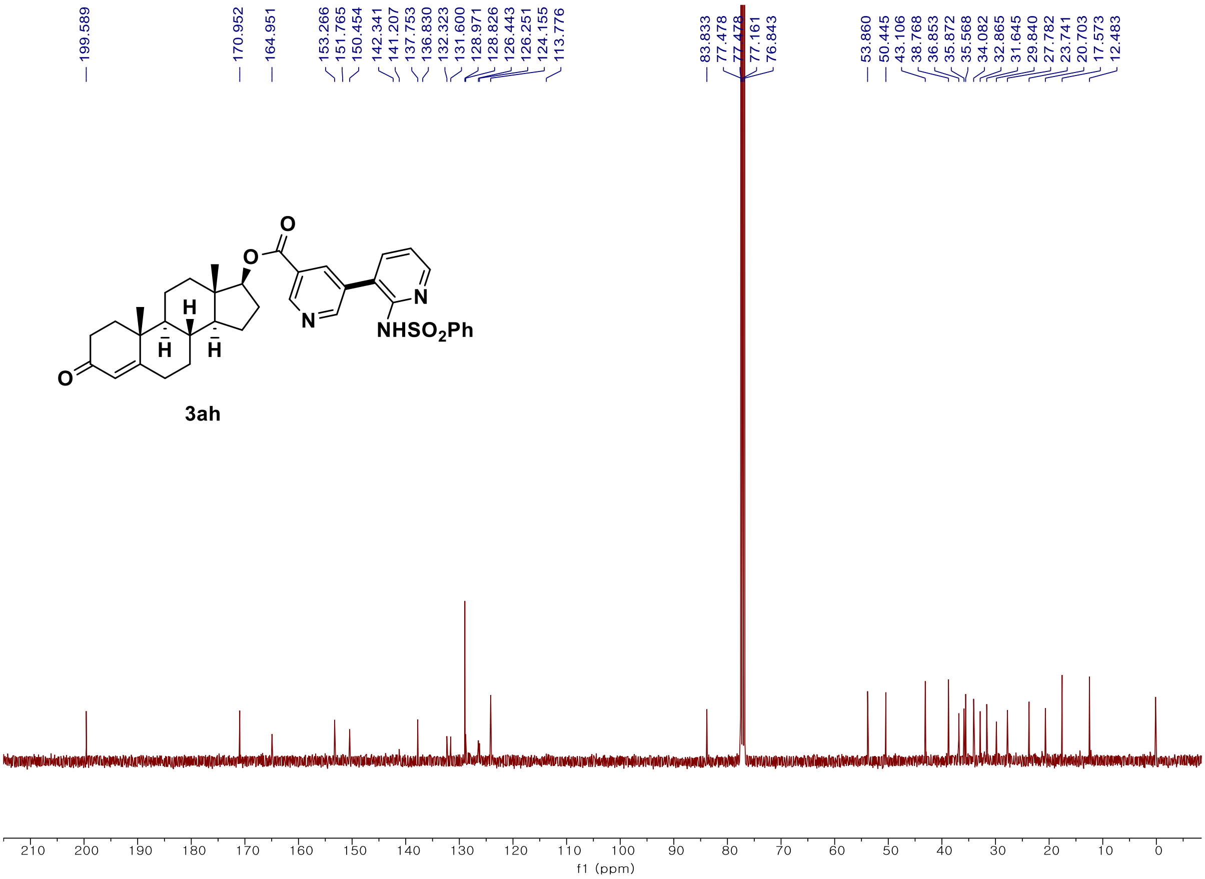
199.589 170.952 164.951 153.266 151.765 150.454 142.341 141.207 137.753 136.830 132.323 131.600 128.971 128.826 126.443 126.251 124.155 113.776 83.833 77.478 77.478 77.161 76.843 53.860 50.445 43.106 38.768 36.853 35.872 35.568 34.082 32.865 31.645 29.840 27.782 23.741 20.703 17.573 12.483
NHSO2Ph
3ah
f1 (ppm)

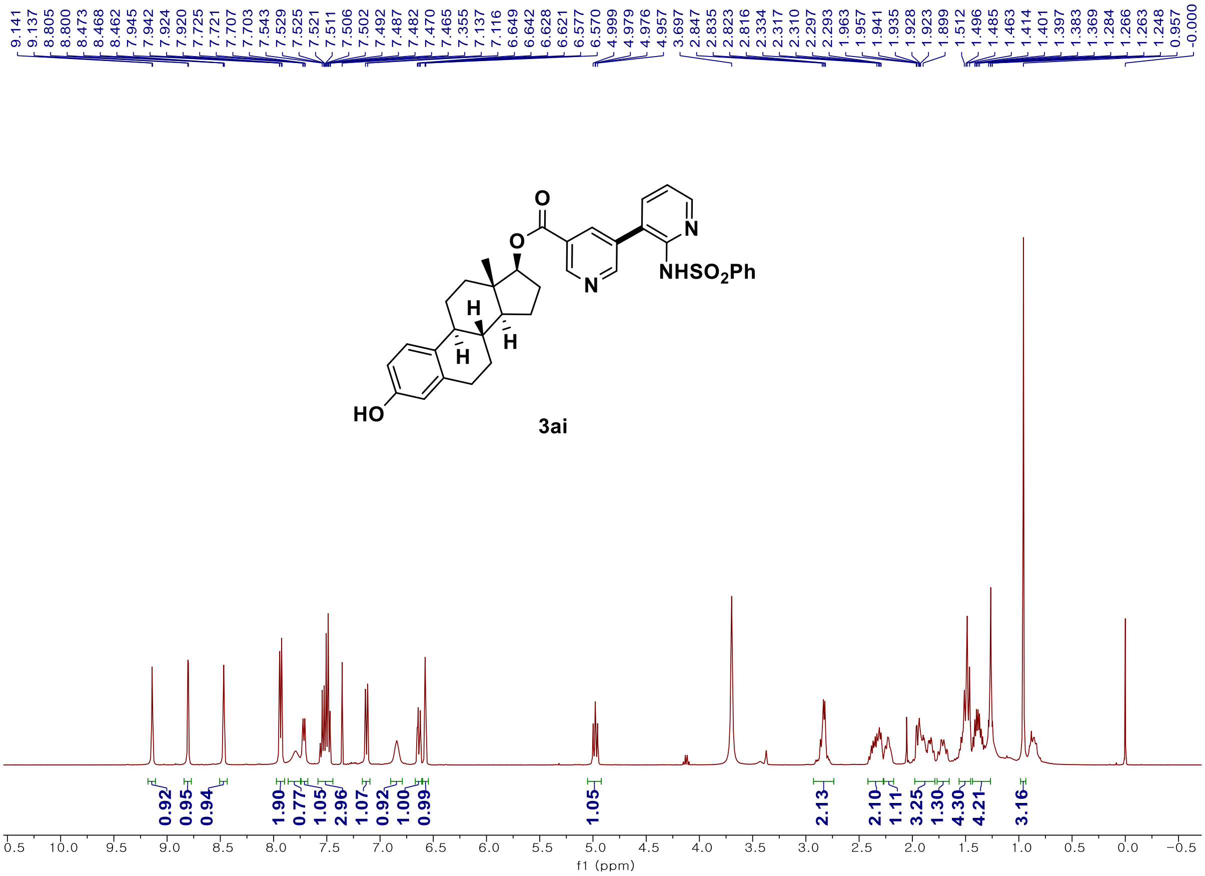
9.141 9.137 8.805 8.800 8.473 8.468 8.462 7.945 7.942 7.924 7.920 7.725 7.721 7.707 7.703 7.543 7.529 7.525 7.521 7.511 7.506 7.502 7.492 7.487 7.482 7.470 7.465 7.355 7.137 7.116 6.649 6.642 6.628 6.621 6.577 6.570 4.999 4.979 4.976 4.957 3.697 2.847 2.835 2.823 2.816 2.334 2.317 2.310 2.297 2.293 1.963 1.957 1.941 1.935 1.928 1.923 1.899 1.512 1.496 1.485 1.463 1.414 1.401 1.397 1.383 1.369 1.284 1.266 1.263 1.248 0.957 -0.000
O
O
N
N
NHSO2Ph
H
H
H
HO
3ai
0.92 0.95 0.94 1.90 0.77 1.05 2.96 1.07 0.92 1.00 0.99 1.05 2.13 2.10 1.11 3.25 1.30 4.30 4.21 3.16
f1 (ppm)

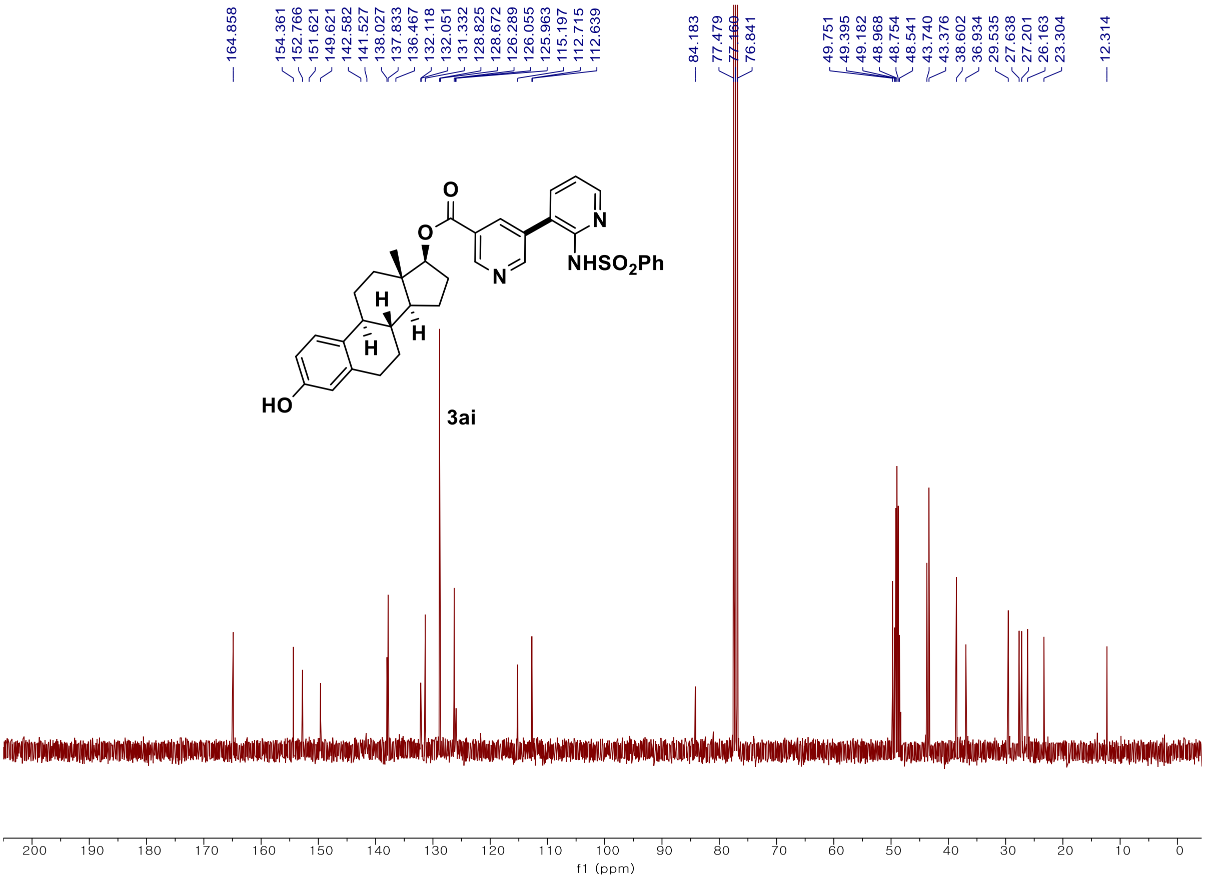
164.858 154.361 152.766 151.621 149.621 142.582 141.527 138.027 137.833 136.467 132.118 132.051 131.332 128.825 128.672 126.289 126.055 125.963 115.197 112.715 112.639 84.183 77.479 77.160 76.841 49.751 49.395 49.182 48.968 48.754 48.541 43.740 43.376 38.602 36.934 29.535 27.638 27.201 26.163 23.304 12.314
O
O
N
N
NHSO2Ph
H
H
H
HO
3ai
f1 (ppm)

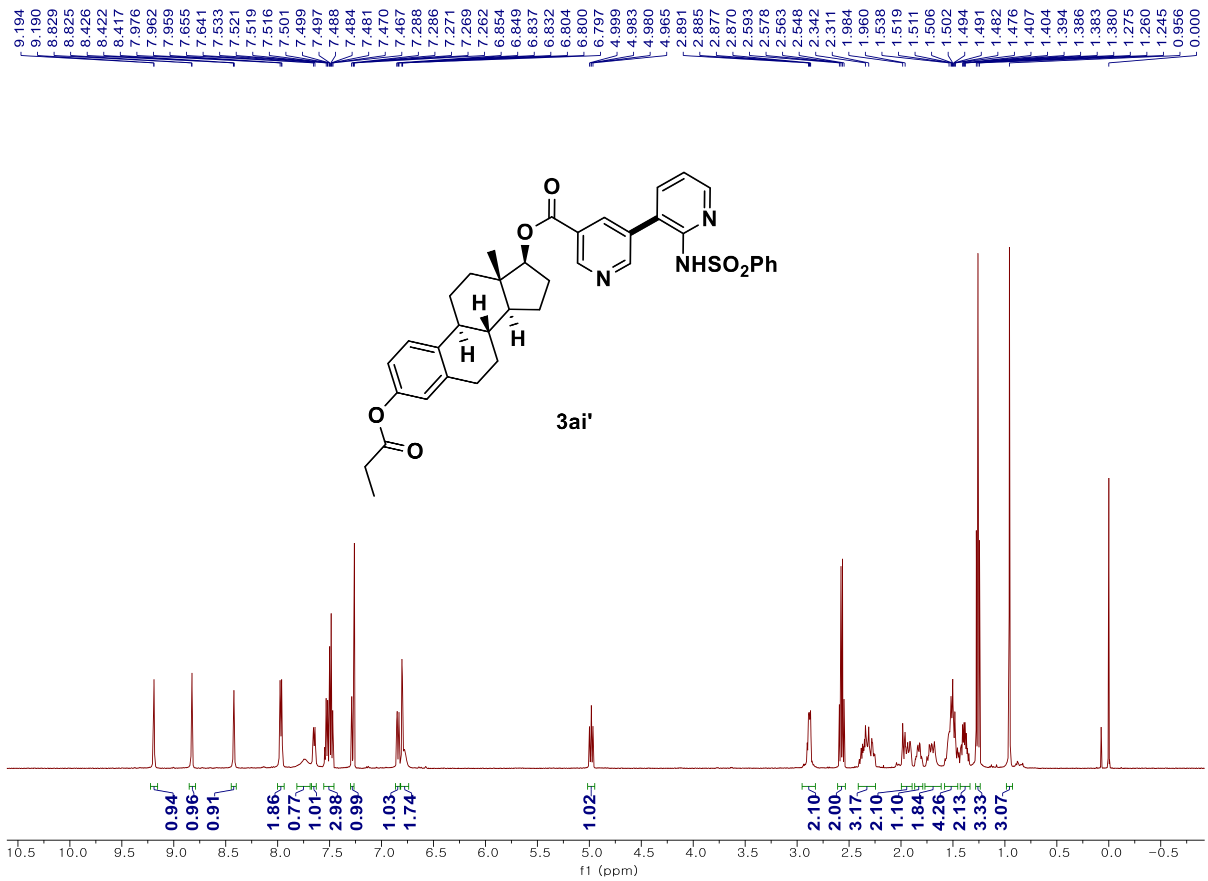
9.194 9.190 8.829 8.825 8.426 8.422 8.417 7.976 7.962 7.959 7.655 7.641 7.533 7.521 7.519 7.516 7.501 7.499 7.497 7.488 7.484 7.481 7.470 7.467 7.288 7.286 7.271 7.269 7.262 6.854 6.849 6.837 6.832 6.804 6.800 6.797 4.999 4.983 4.980 4.965 2.891 2.885 2.877 2.870 2.593 2.578 2.563 2.548 2.342 2.311 1.984 1.960 1.538 1.519 1.511 1.506 1.502 1.494 1.491 1.482 1.476 1.407 1.404 1.394 1.386 1.383 1.380 1.275 1.260 1.245 0.956 0.000
O
O
N
N
NHSO2Ph
H
H
H
O
O
3ai'
0.94 0.96 0.91 1.86 0.77 1.01 2.98 0.99 1.03 1.74 1.02 2.10 2.00 3.17 2.10 1.10 1.84 4.26 2.13 3.33 3.07
10.5 10.0 9.5 9.0 8.5 8.0 7.5 7.0 6.5 6.0 5.5 5.0 4.5 4.0 3.5 3.0 2.5 2.0 1.5 1.0 0.5 0.0 -0.5
f1 (ppm)

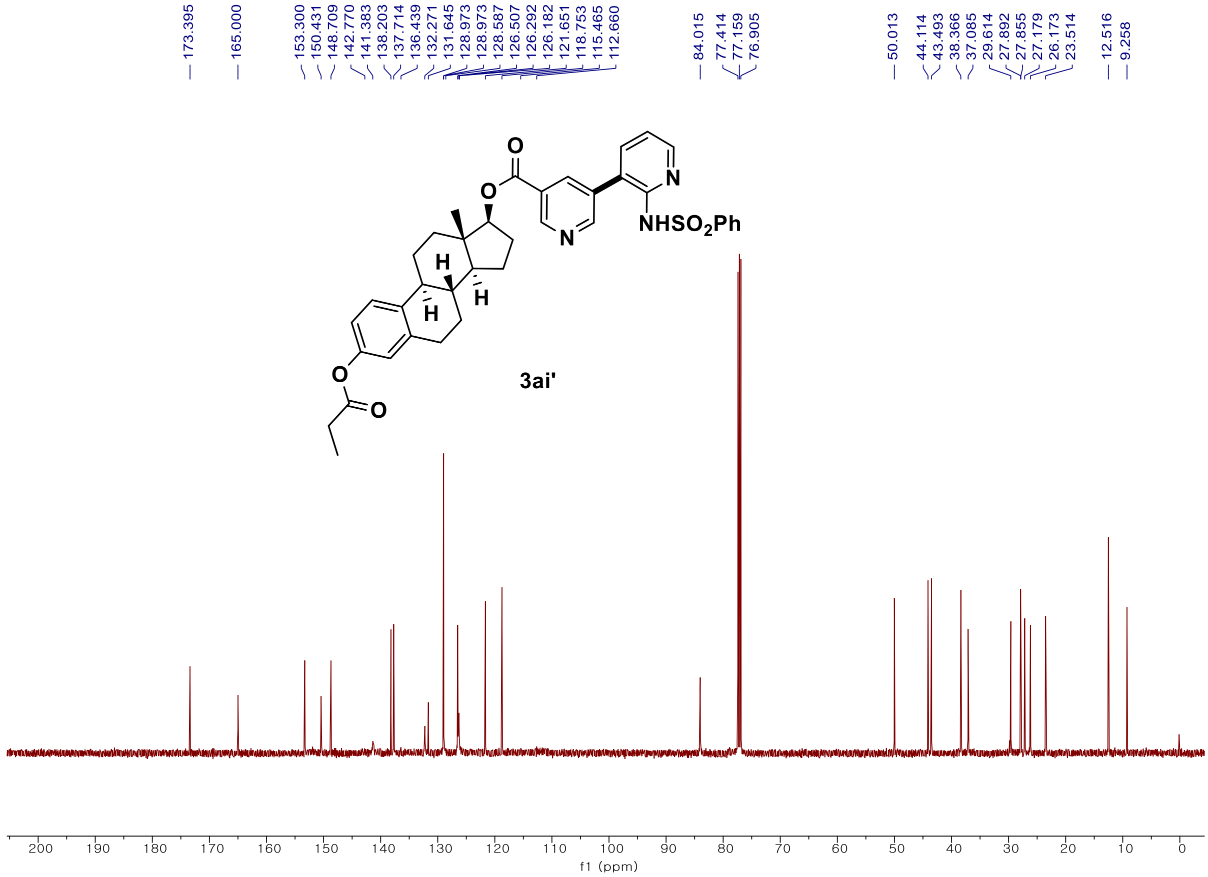
173.395
165.000
153.300 150.431 148.709 142.770 141.383 138.203 137.714 136.439 132.271 131.645 128.973 128.973 128.587 126.507 126.292 126.182 121.651 118.753 115.465 112.660
84.015 77.414 77.159 76.905
50.013 44.114 43.493 38.366 37.085 29.614 27.892 27.855 27.179 26.173 23.514
12.516 9.258
O
O
N
N
NHSO2Ph
H
H
H
O
O
3ai'
200 190 180 170 160 150 140 130 120 110 100 90 80 70 60 50 40 30 20 10 0
f1 (ppm)

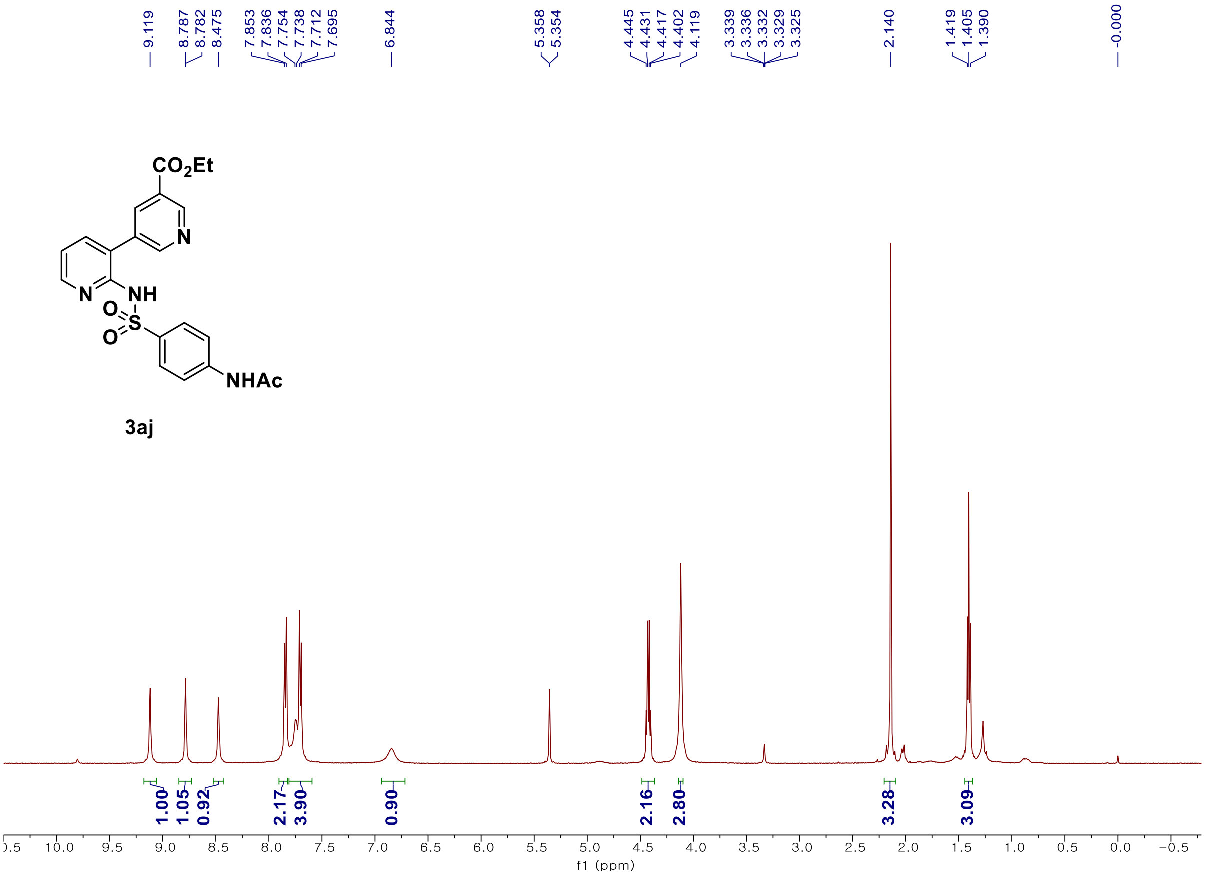

9.119
8.787
8.782
8.475
7.853
7.836
7.754
7.738
7.712
7.695
6.844
5.358
5.354
4.445
4.431
4.417
4.402
4.119
3.339
3.336
3.332
3.329
3.325
2.140
1.419
1.405
1.390
-0.000
CO2Et
N
N
NH
O
S
O
NHAc
3aj
1.00
1.05
0.92
2.17
3.90
0.90
2.16
2.80
3.28
3.09
f1 (ppm)

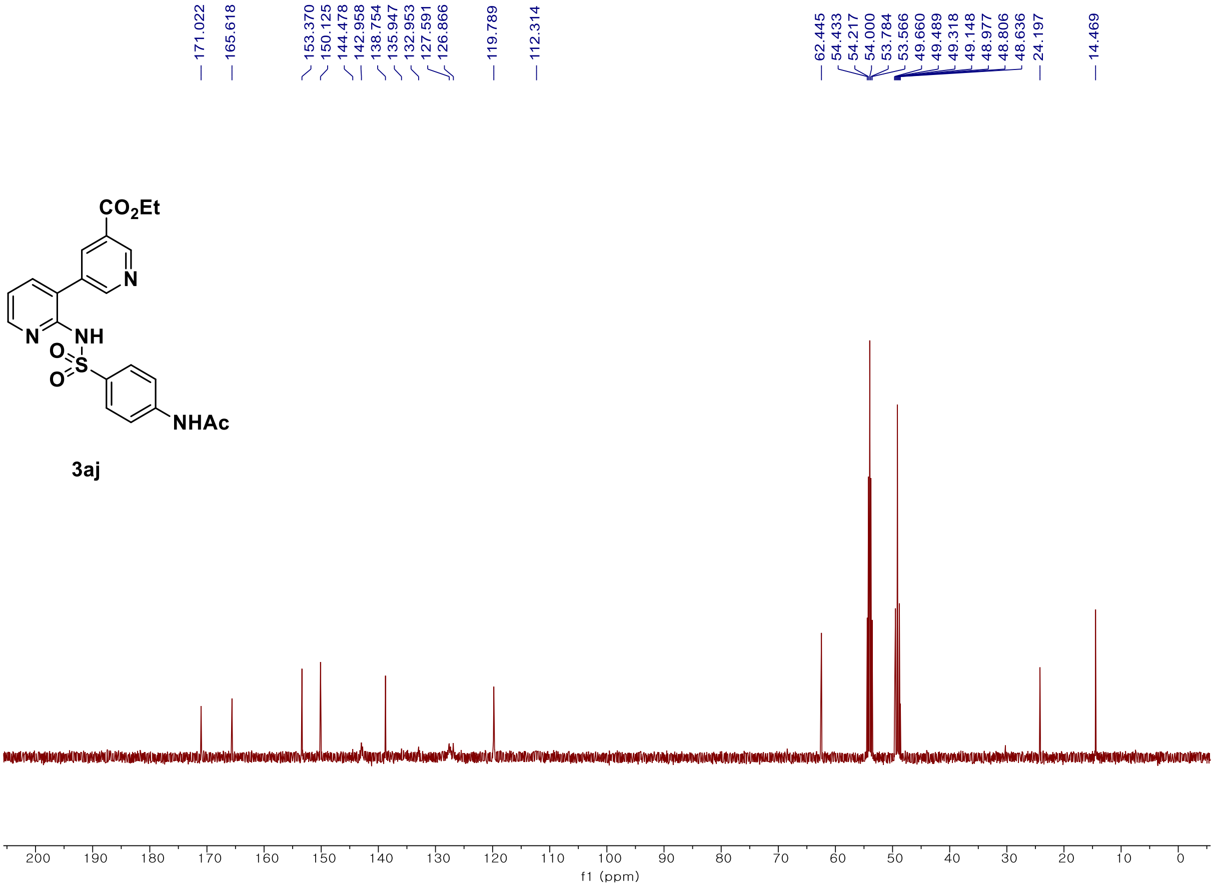

171.022
165.618
153.370
150.125
144.478
142.958
138.754
135.947
132.953
127.591
126.866
119.789
112.314
62.445
54.433
54.217
54.000
53.784
53.566
49.660
49.489
49.318
49.148
48.977
48.806
48.636
24.197
14.469
CO2Et
N
N
NH
O
S
O
NHAc
3aj
f1 (ppm)

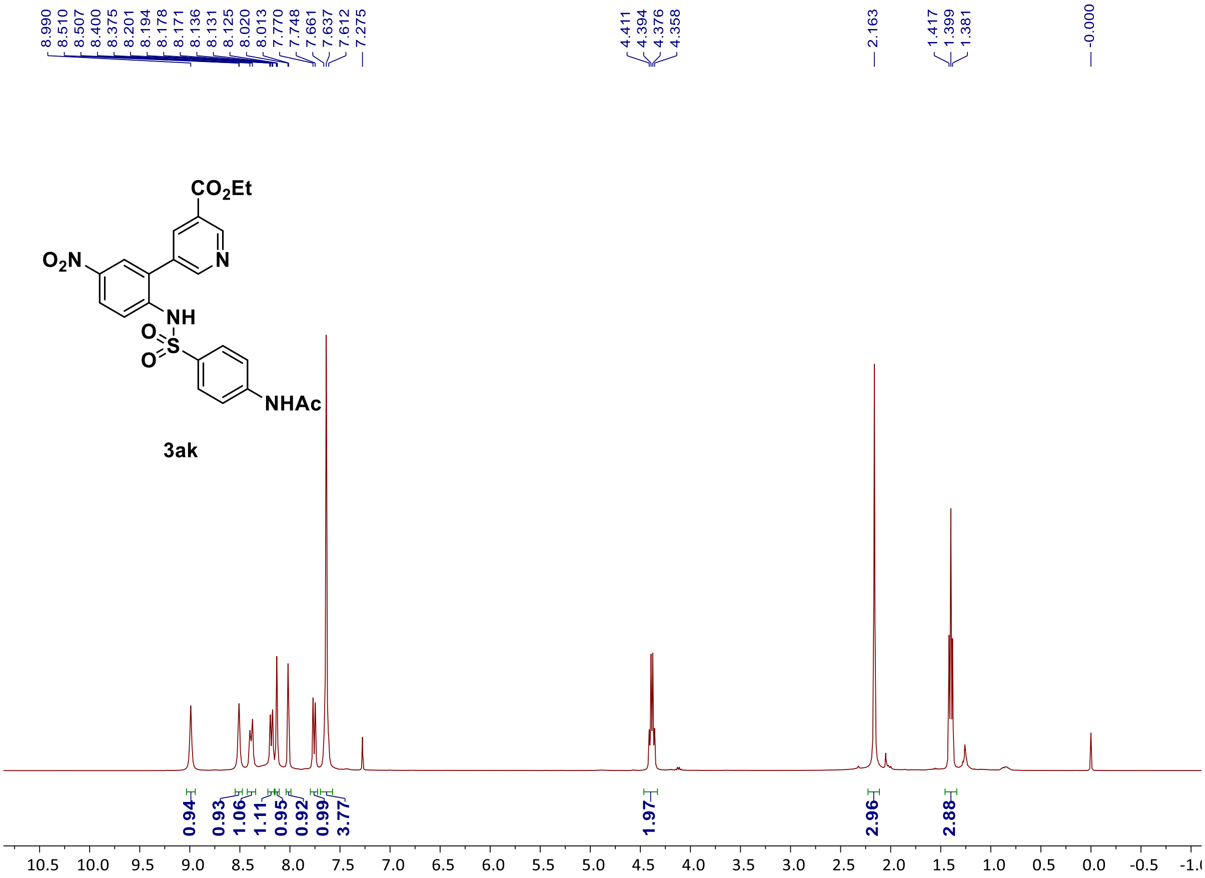
8.990
8.510
8.507
8.400
8.375
8.201
8.194
8.178
8.171
8.136
8.131
8.125
8.020
8.013
7.770
7.748
7.661
7.637
7.612
7.275
4.411
4.394
4.376
4.358
2.163
1.417
1.399
1.381
-0.000
CO2Et
O2N
N
NH
O
S
O
NHAc
3ak
0.94
0.93
1.06
1.11
0.95
0.92
0.99
3.77
1.97
2.96
2.88
10.5
10.0
9.5
9.0
8.5
8.0
7.5
7.0
6.5
6.0
5.5
5.0
4.5
4.0
3.5
3.0
2.5
2.0
1.5
1.0
0.5
0.0
-0.5

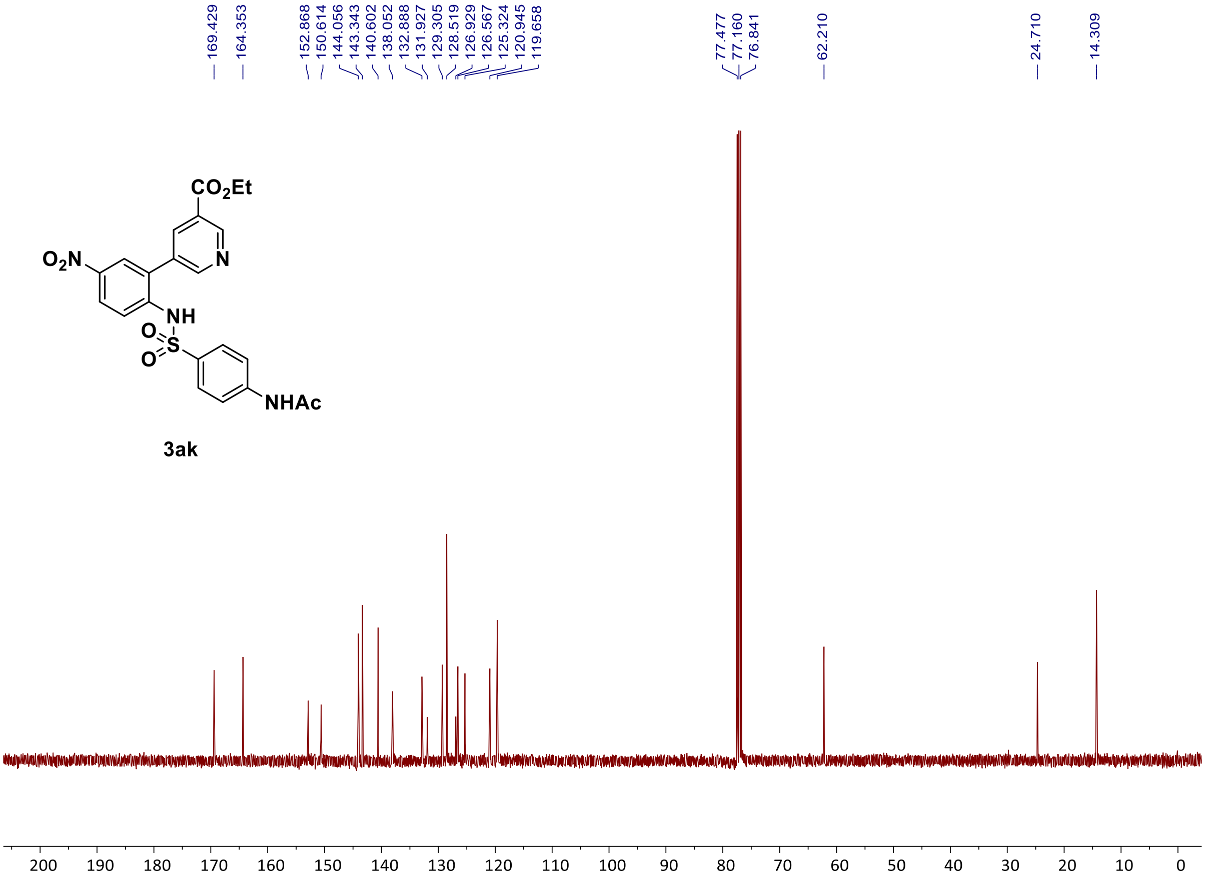
169.429
164.353
152.868
150.614
144.056
143.343
140.602
138.052
132.888
131.927
129.305
128.519
126.929
126.567
125.324
120.945
119.658
77.477
77.160
76.841
62.210
24.710
14.309
CO2Et
O2N
N
NH
O
S
O
NHAc
3ak
200
190
180
170
160
150
140
130
120
110
100
90
80
70
60
50
40
30
20
10
0

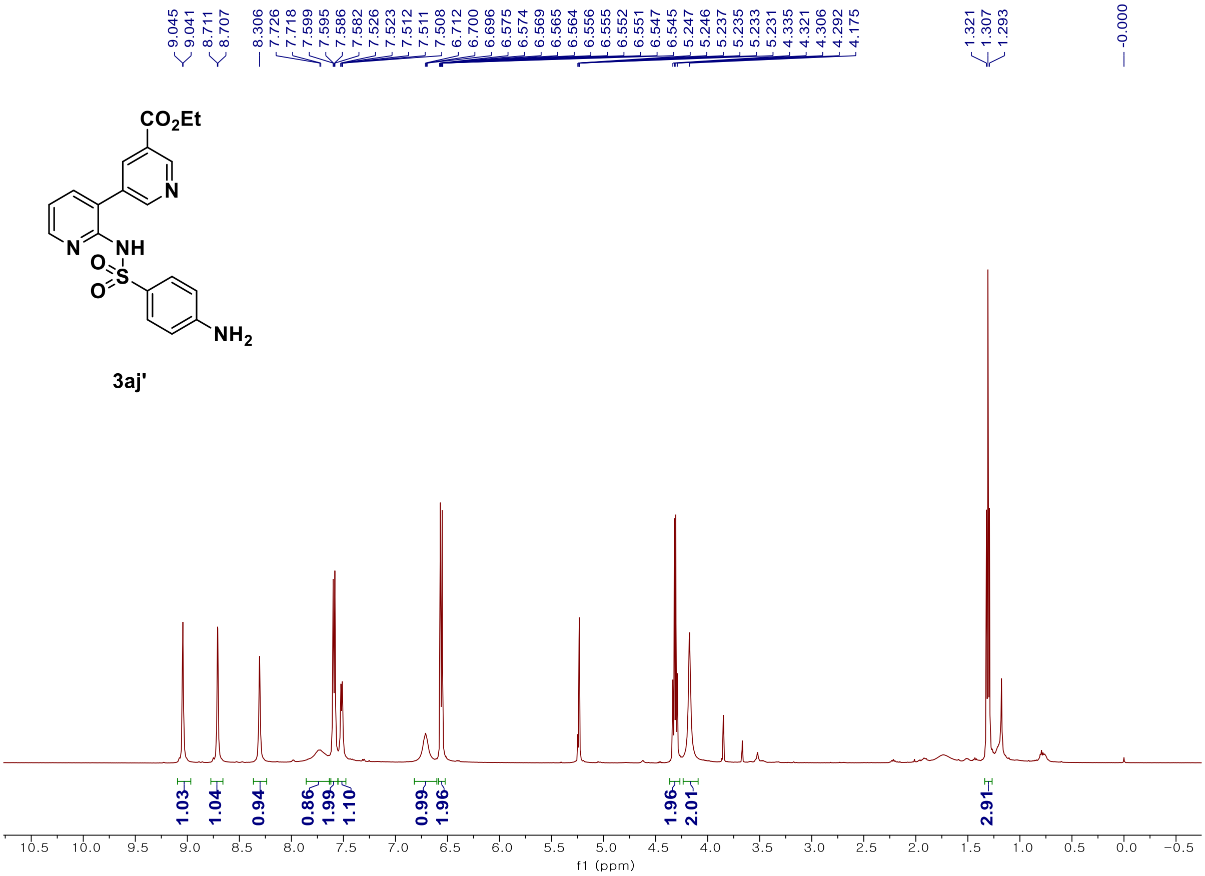
CO2Et
N
NH
O
S
O
NH2
3aj'
f1 (ppm)

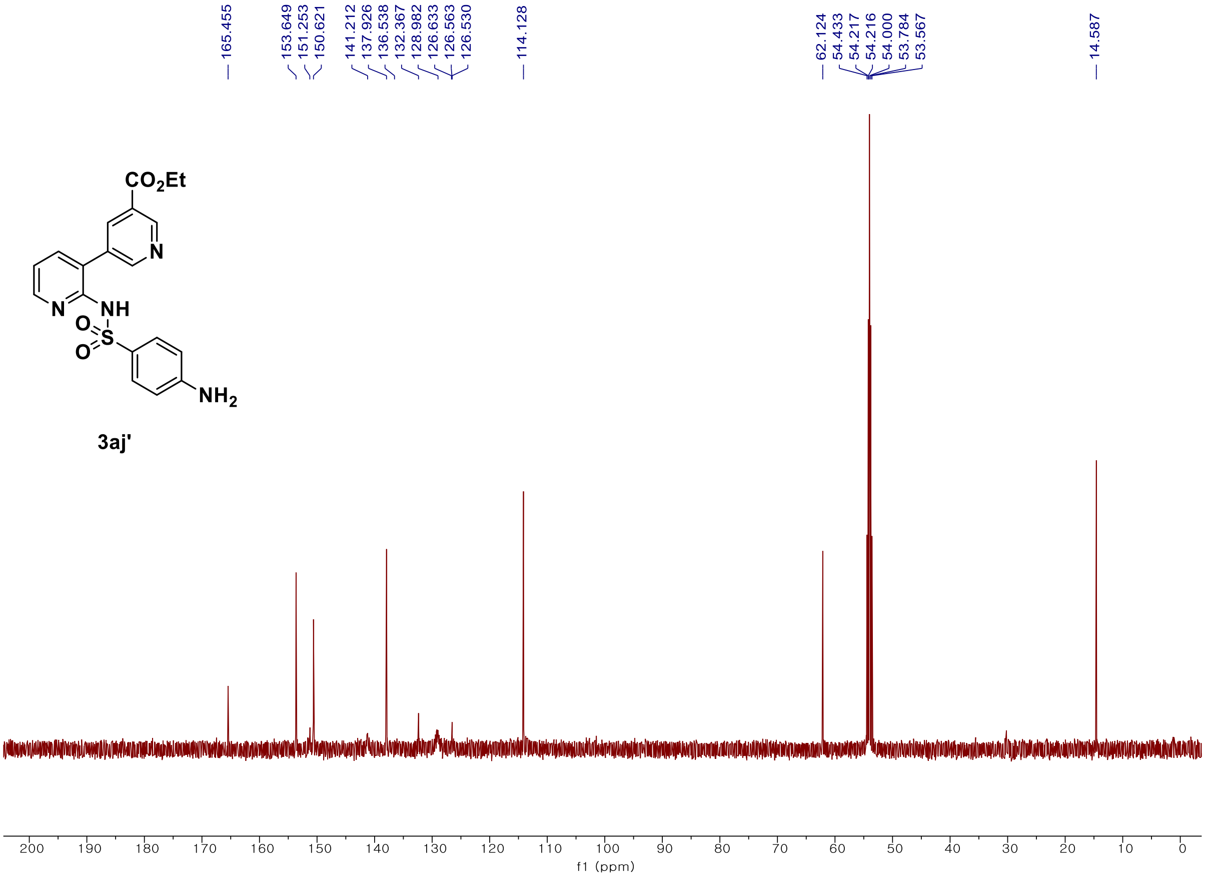
CO2Et
N
NH
O
S
O
NH2
3aj'
f1 (ppm)

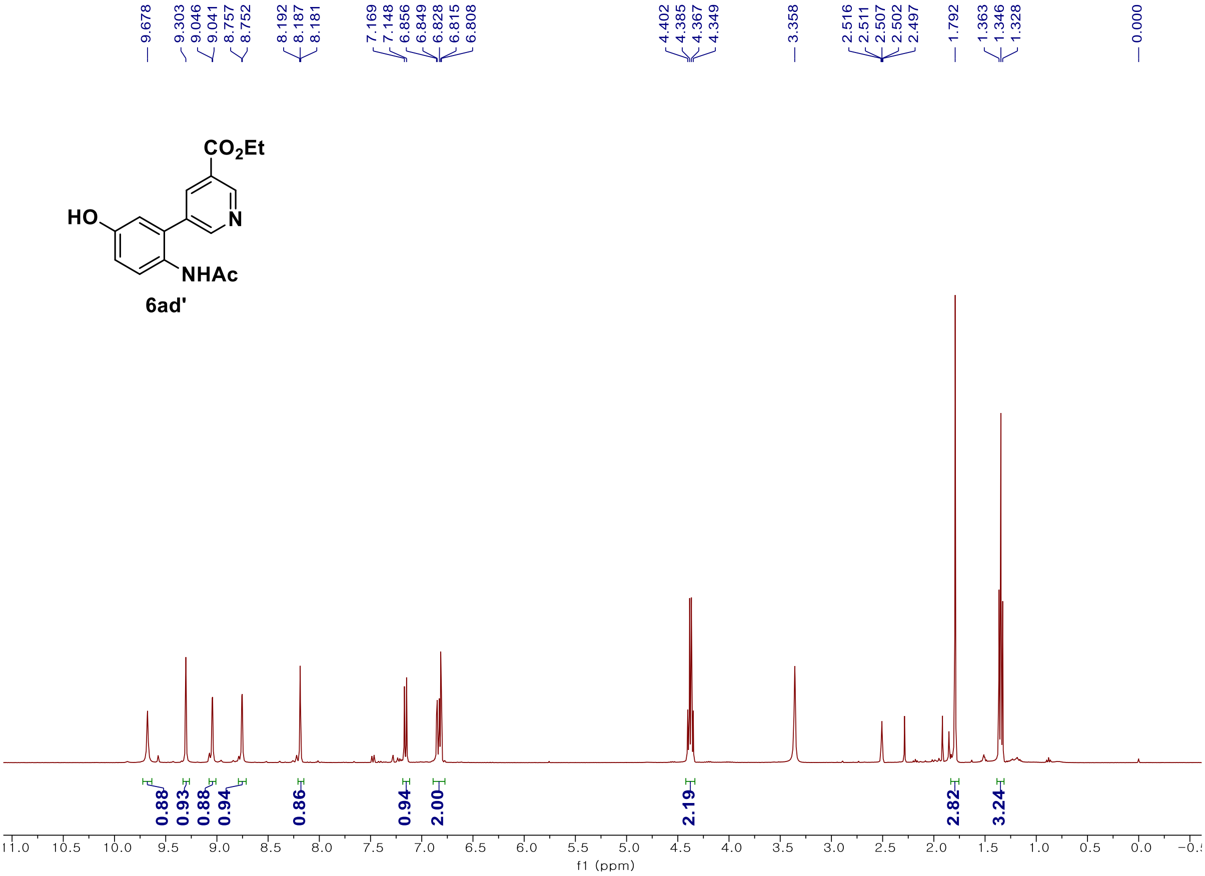

9.678
9.303
9.046
9.041
8.757
8.752
8.192
8.187
8.181
7.169
7.148
6.856
6.849
6.828
6.815
6.808
4.402
4.385
4.367
4.349
3.358
2.516
2.511
2.507
2.502
2.497
1.792
1.363
1.346
1.328
0.000
CO2Et
HO
N
NHAc
6ad'
0.88
0.93
0.88
0.94
0.86
0.94
2.00
2.19
2.82
3.24
f1 (ppm)

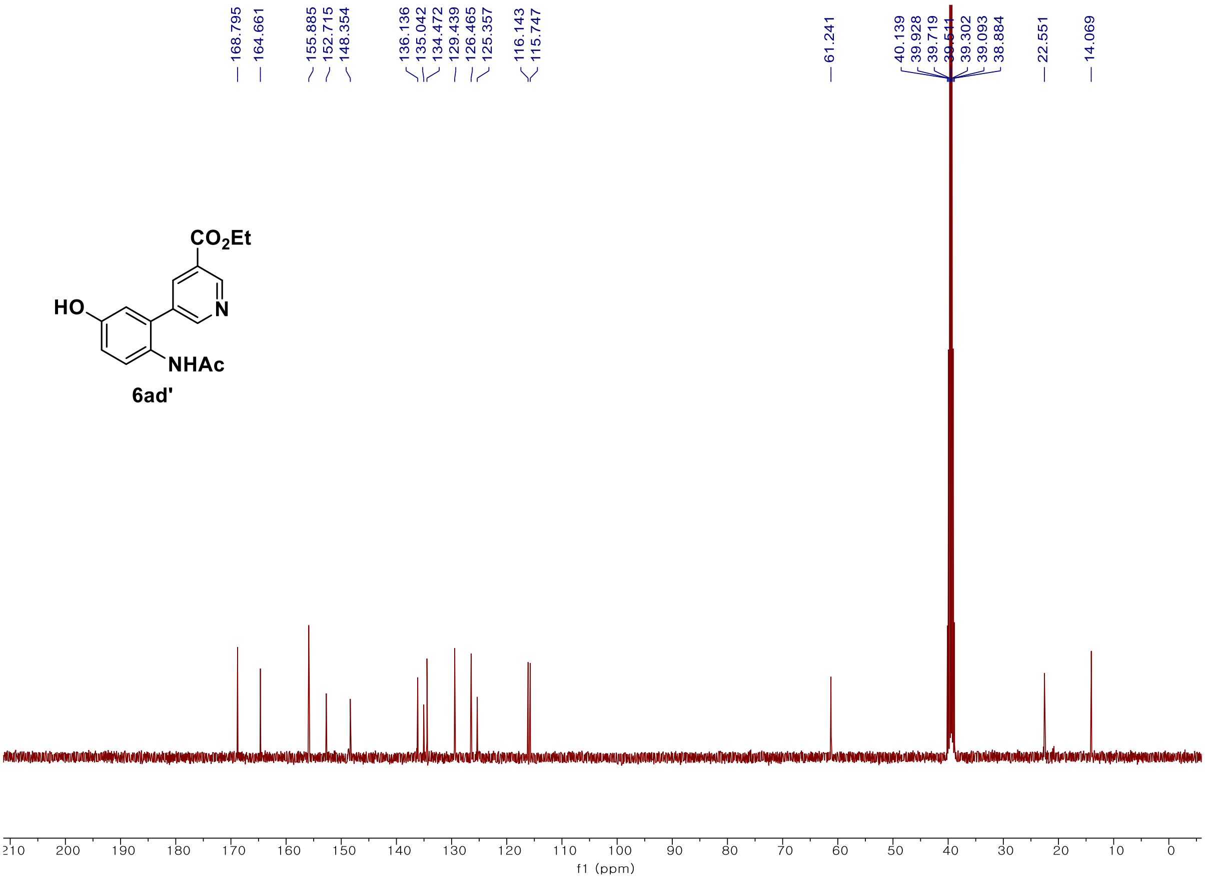

168.795
164.661
155.885
152.715
148.354
136.136
135.042
134.472
129.439
126.465
125.357
116.143
115.747
61.241
40.139
39.928
39.719
39.511
39.302
39.093
38.884
22.551
14.069
CO2Et
HO
N
NHAc
6ad'
f1 (ppm)

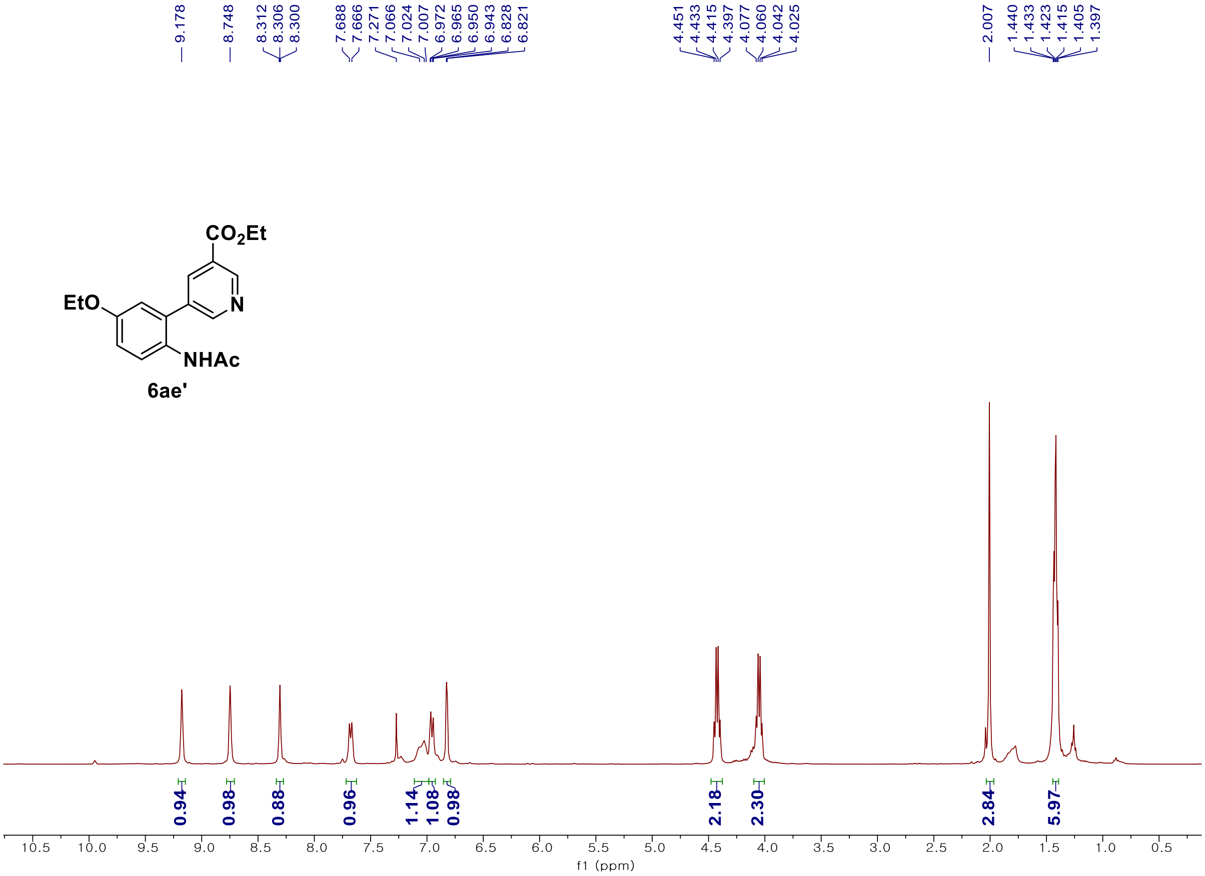
9.178
8.748
8.312
8.306
8.300
7.688
7.666
7.271
7.066
7.024
7.007
6.972
6.965
6.950
6.943
6.828
6.821
4.451
4.433
4.415
4.397
4.077
4.060
4.042
4.025
2.007
1.440
1.433
1.423
1.415
1.405
1.397
CO2Et
EtO
N
NHAc
6ae'
0.94
0.98
0.88
0.96
1.14
1.08
0.98
2.18
2.30
2.84
5.97
f1 (ppm)

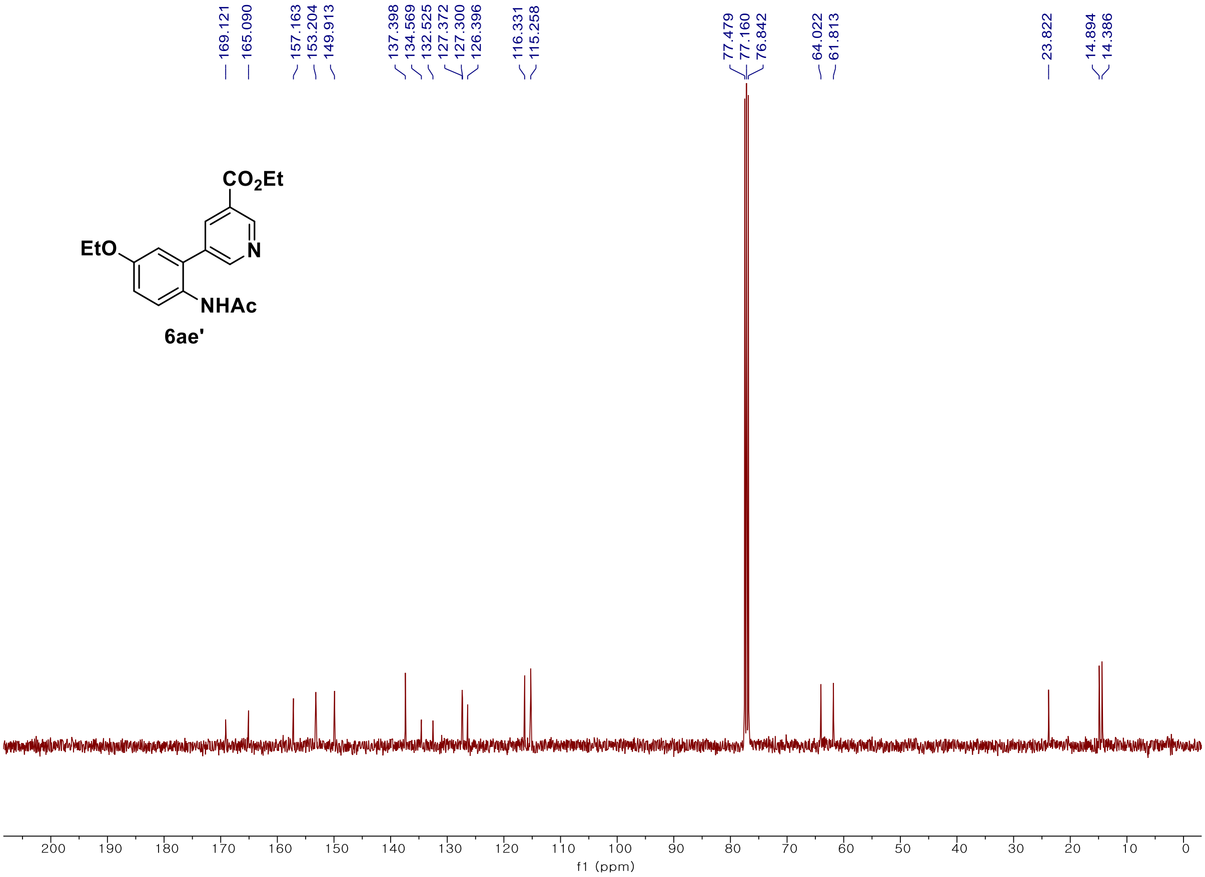
169.121
165.090
157.163
153.204
149.913
137.398
134.569
132.525
127.372
127.300
126.396
116.331
115.258
77.479
77.160
76.842
64.022
61.813
23.822
14.894
14.386
CO2Et
EtO
N
NHAc
6ae'
f1 (ppm)

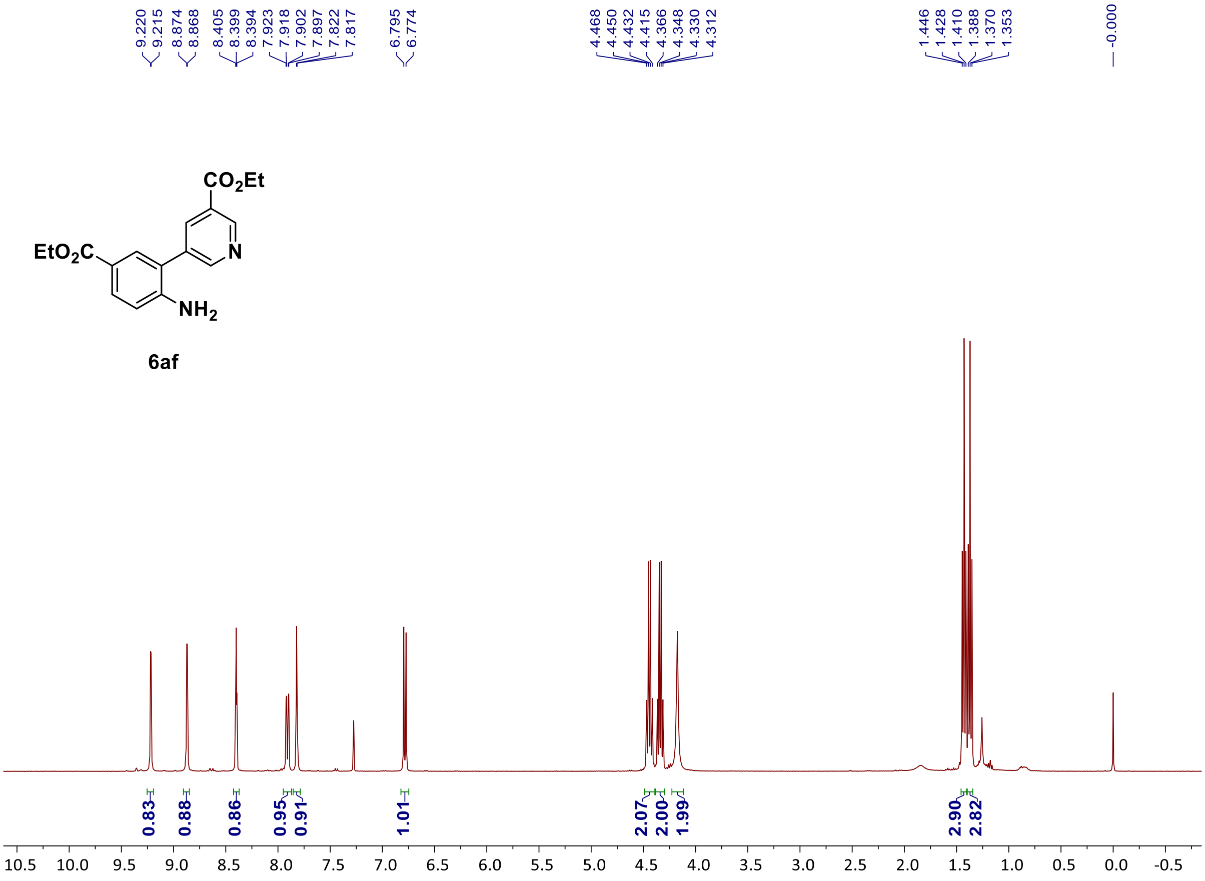
9.220
9.215
8.874
8.868
8.405
8.399
8.394
7.923
7.918
7.902
7.897
7.822
7.817
6.795
6.774
4.468
4.450
4.432
4.415
4.366
4.348
4.330
4.312
1.446
1.428
1.410
1.388
1.370
1.353
-0.000
CO_2Et
EtO_2C
N
NH_2
6af
0.83
0.88
0.86
0.95
0.91
1.01
2.07
2.00
1.99
2.90
2.82
10.5 10.0 9.5 9.0 8.5 8.0 7.5 7.0 6.5 6.0 5.5 5.0 4.5 4.0 3.5 3.0 2.5 2.0 1.5 1.0 0.5 0.0 -0.5

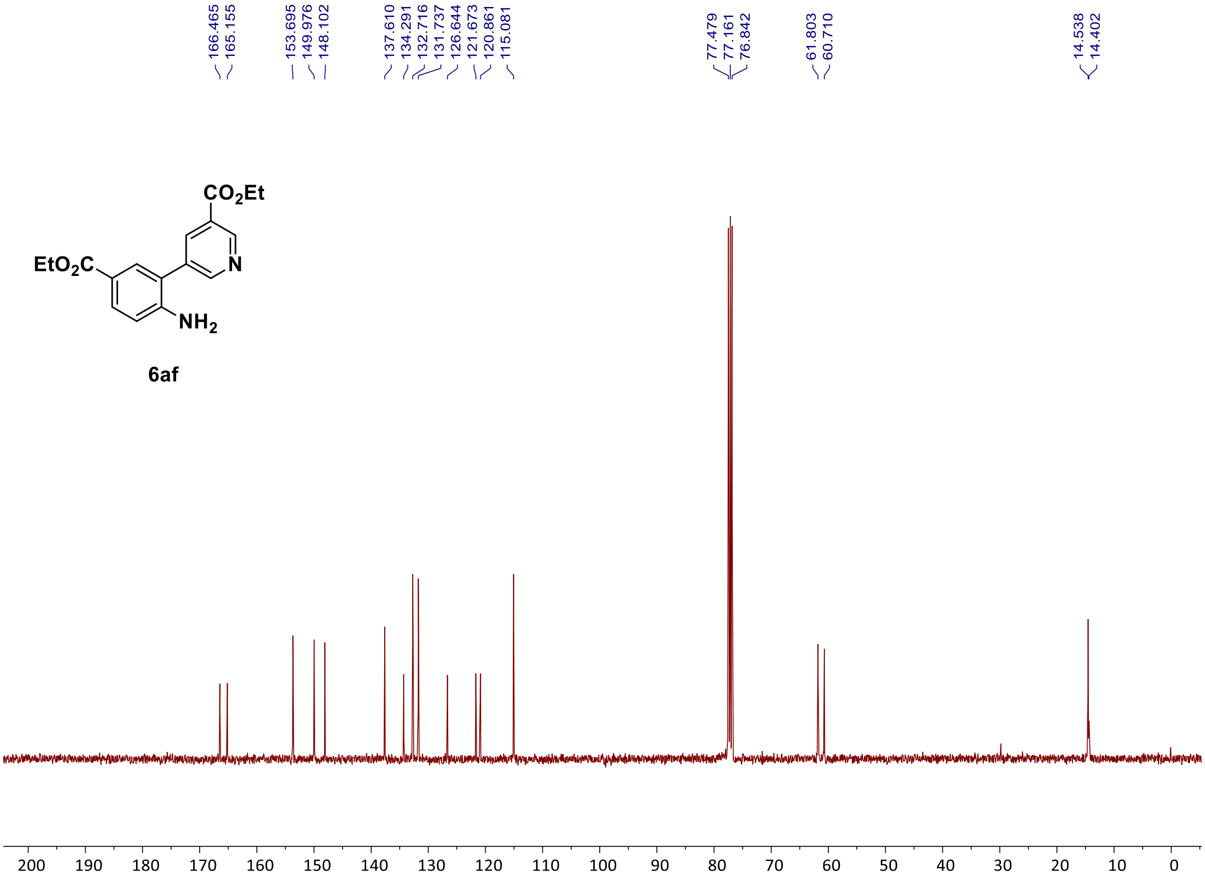
166.465
165.155
153.695
149.976
148.102
137.610
134.291
132.716
131.737
126.644
121.673
120.861
115.081
77.479
77.161
76.842
61.803
60.710
14.538
14.402
CO_2Et
EtO_2C
N
NH_2
6af
200 190 180 170 160 150 140 130 120 110 100 90 80 70 60 50 40 30 20 10 0

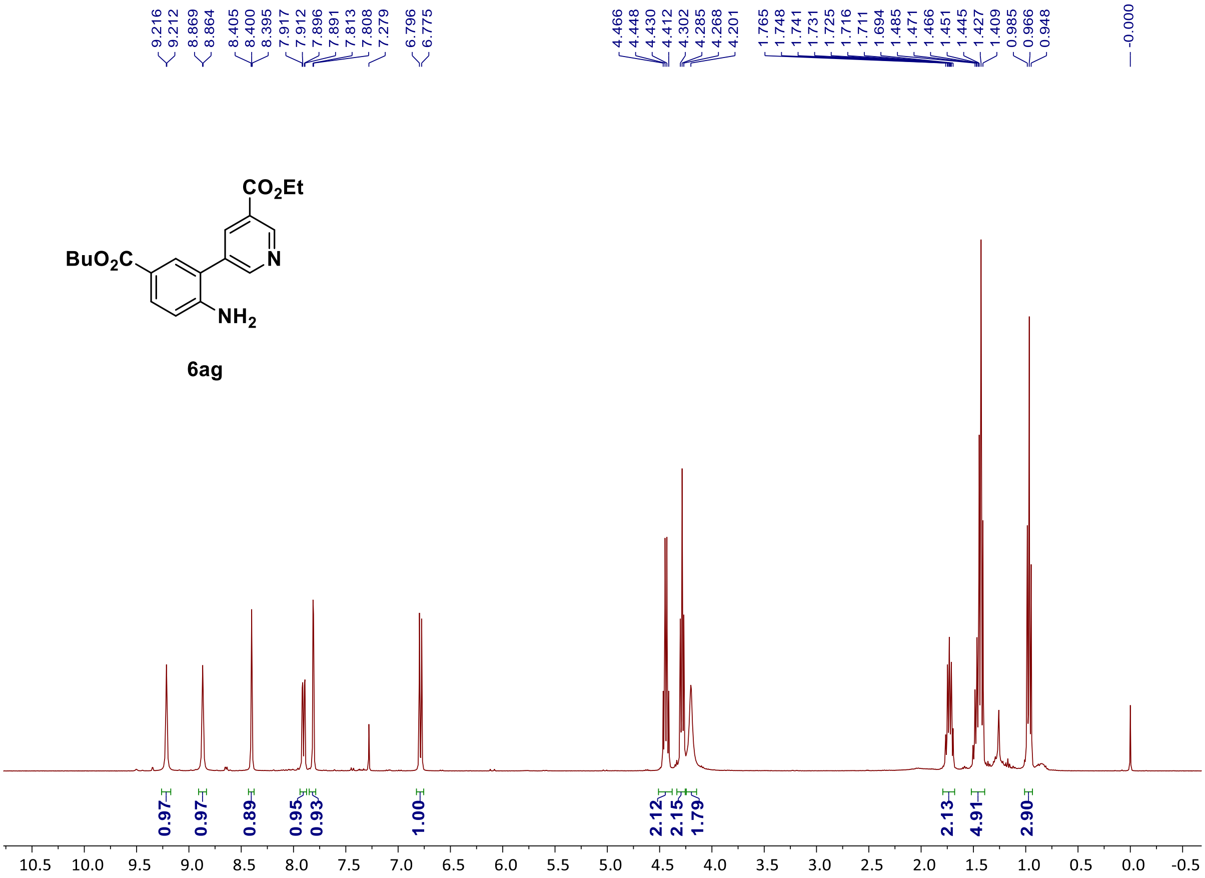
9.216
9.212
8.869
8.864
8.405
8.400
8.395
7.917
7.912
7.896
7.891
7.813
7.808
7.279
6.796
6.775
4.466
4.448
4.430
4.412
4.302
4.285
4.268
4.201
1.765
1.748
1.741
1.731
1.725
1.716
1.711
1.694
1.485
1.471
1.466
1.451
1.445
1.427
1.409
0.985
0.966
0.948
-0.000
CO2Et
BuO2C
N
NH2
6ag
0.97
0.97
0.89
0.95
0.93
1.00
2.12
2.15
1.79
2.13
4.91
2.90
10.5 10.0 9.5 9.0 8.5 8.0 7.5 7.0 6.5 6.0 5.5 5.0 4.5 4.0 3.5 3.0 2.5 2.0 1.5 1.0 0.5 0.0 -0.5

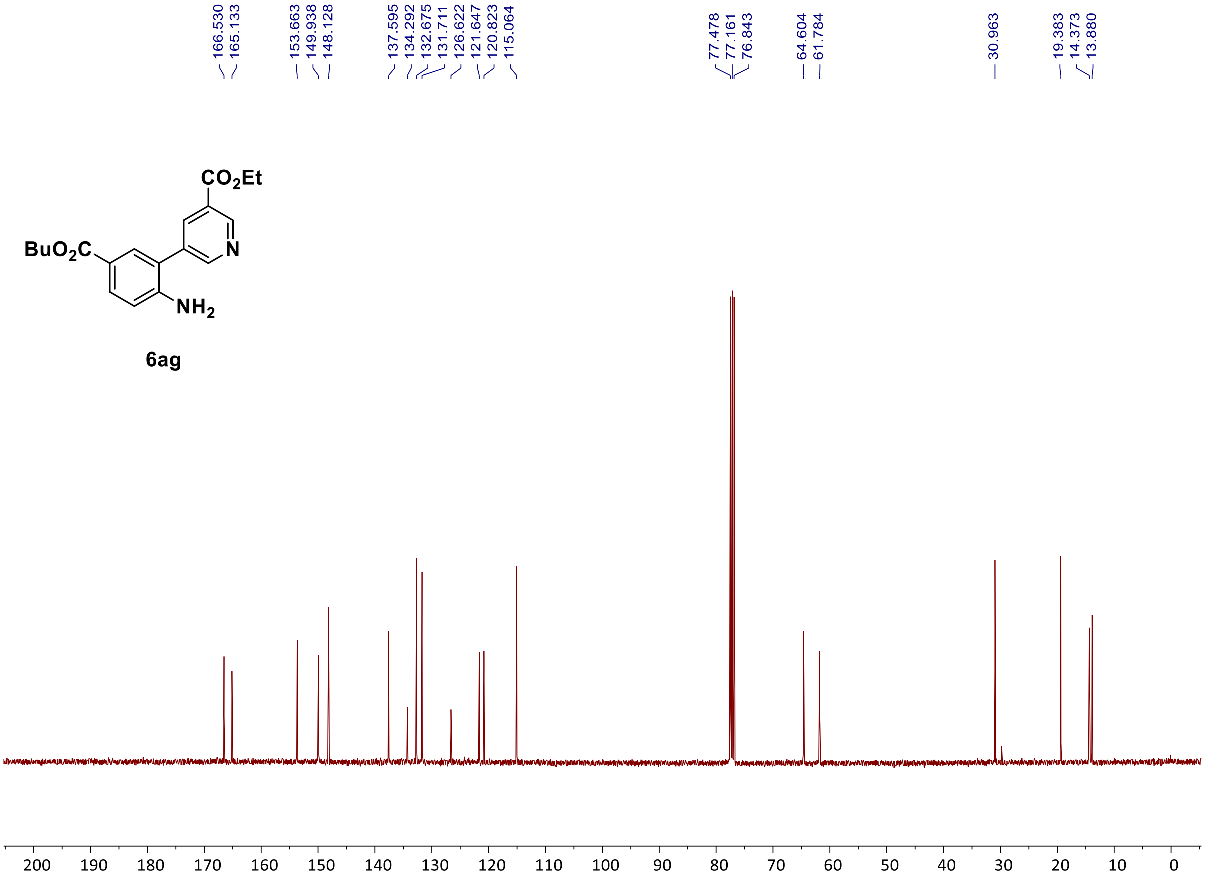
166.530
165.133
153.663
149.938
148.128
137.595
134.292
132.675
131.711
126.622
121.647
120.823
115.064
77.478
77.161
76.843
64.604
61.784
30.963
19.383
14.373
13.880
CO2Et
BuO2C
N
NH2
6ag
200 190 180 170 160 150 140 130 120 110 100 90 80 70 60 50 40 30 20 10 0

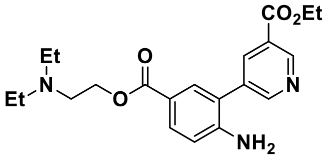

6ah

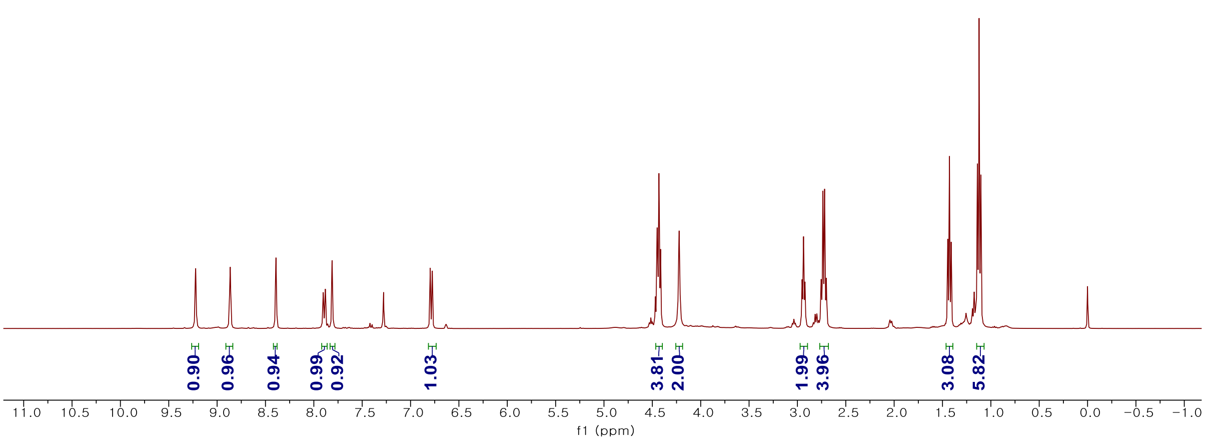
9.224
9.221
8.868
8.864
8.391
8.386
7.906
7.901
7.885
7.880
7.813
7.808
7.279
6.797
6.775
4.451
4.431
4.415
4.223
2.952
2.937
2.921
2.755
2.737
2.719
2.701
1.446
1.428
1.410
1.139
1.122
1.104
0.000
0.90
0.96
0.94
0.99
0.92
1.03
3.81
2.00
1.99
3.96
3.08
5.82
f1 (ppm)

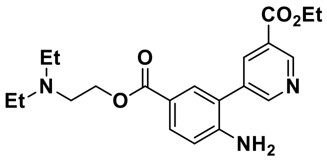

6ah

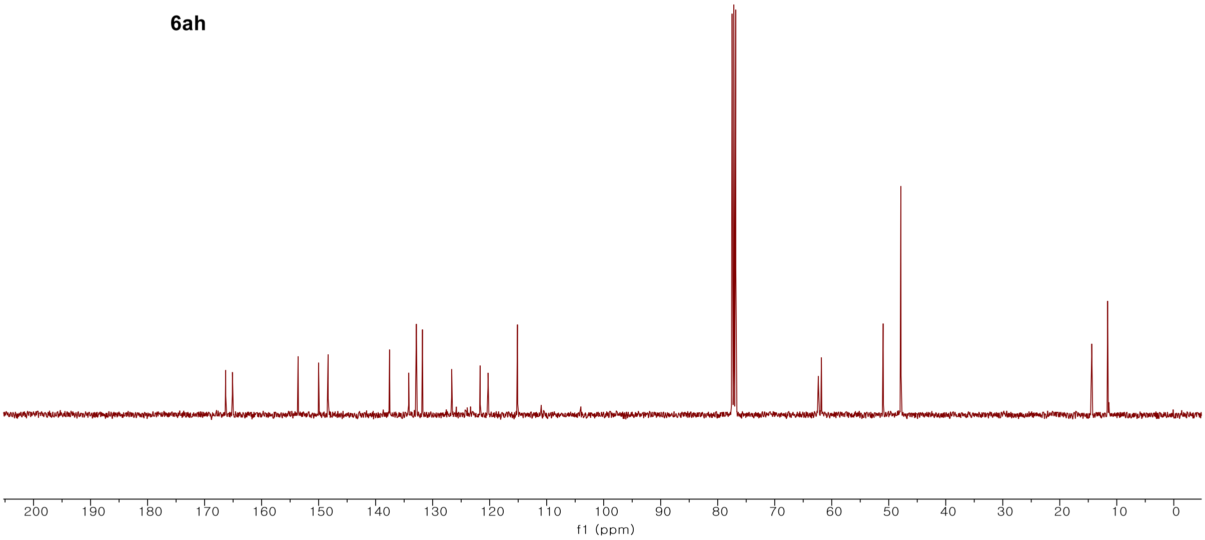
166.301
165.121
153.606
149.998
148.338
137.555
134.186
132.856
131.791
126.659
121.675
120.289
115.123
77.478
77.161
76.842
62.348
61.811
50.979
47.870
14.393
11.598
f1 (ppm)

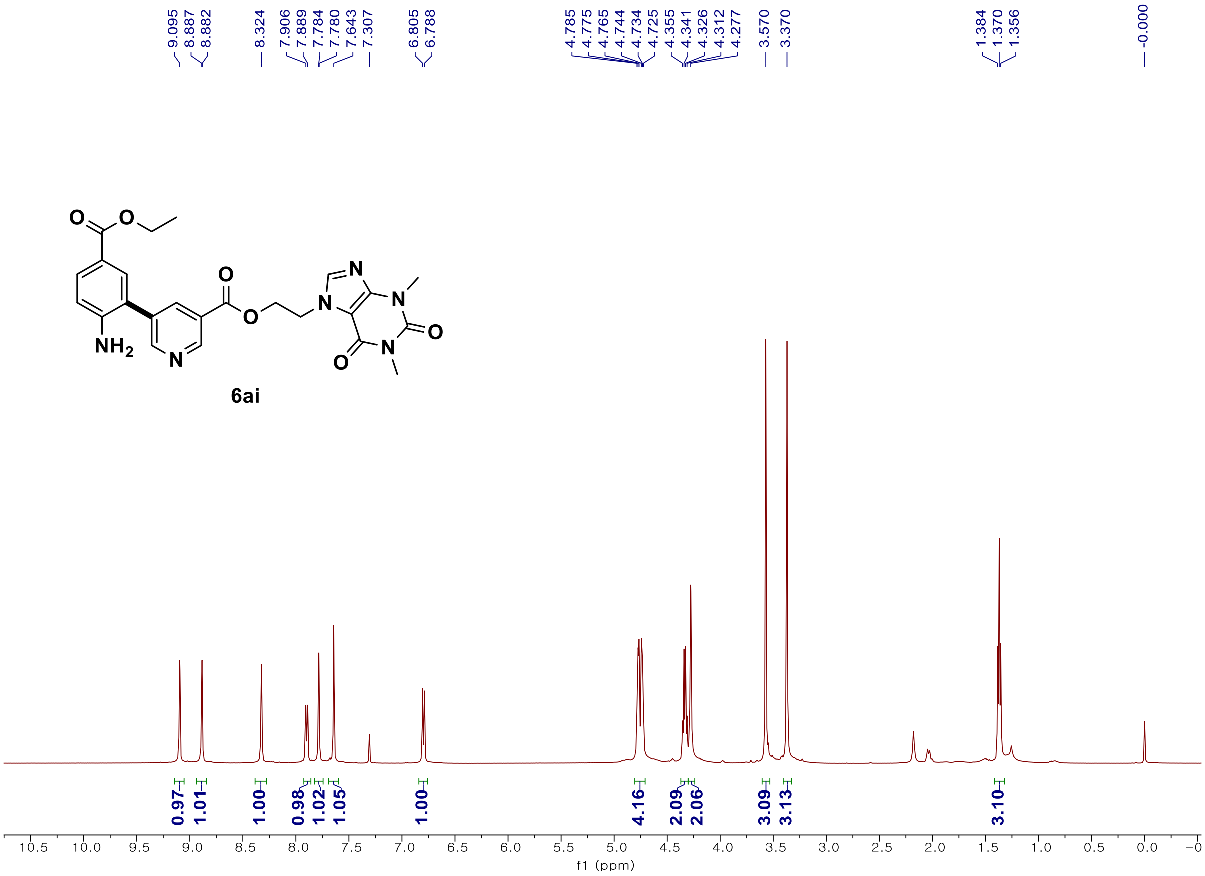
9.095
8.887
8.882
8.324
7.906
7.889
7.784
7.780
7.643
7.307
6.805
6.788
4.785
4.775
4.765
4.744
4.734
4.725
4.355
4.341
4.326
4.312
4.277
3.570
3.370
1.384
1.370
1.356
-0.000
NH2
6ai
0.97
1.01
1.00
0.98
1.02
1.05
1.00
4.16
2.09
2.06
3.09
3.13
3.10
f1 (ppm)

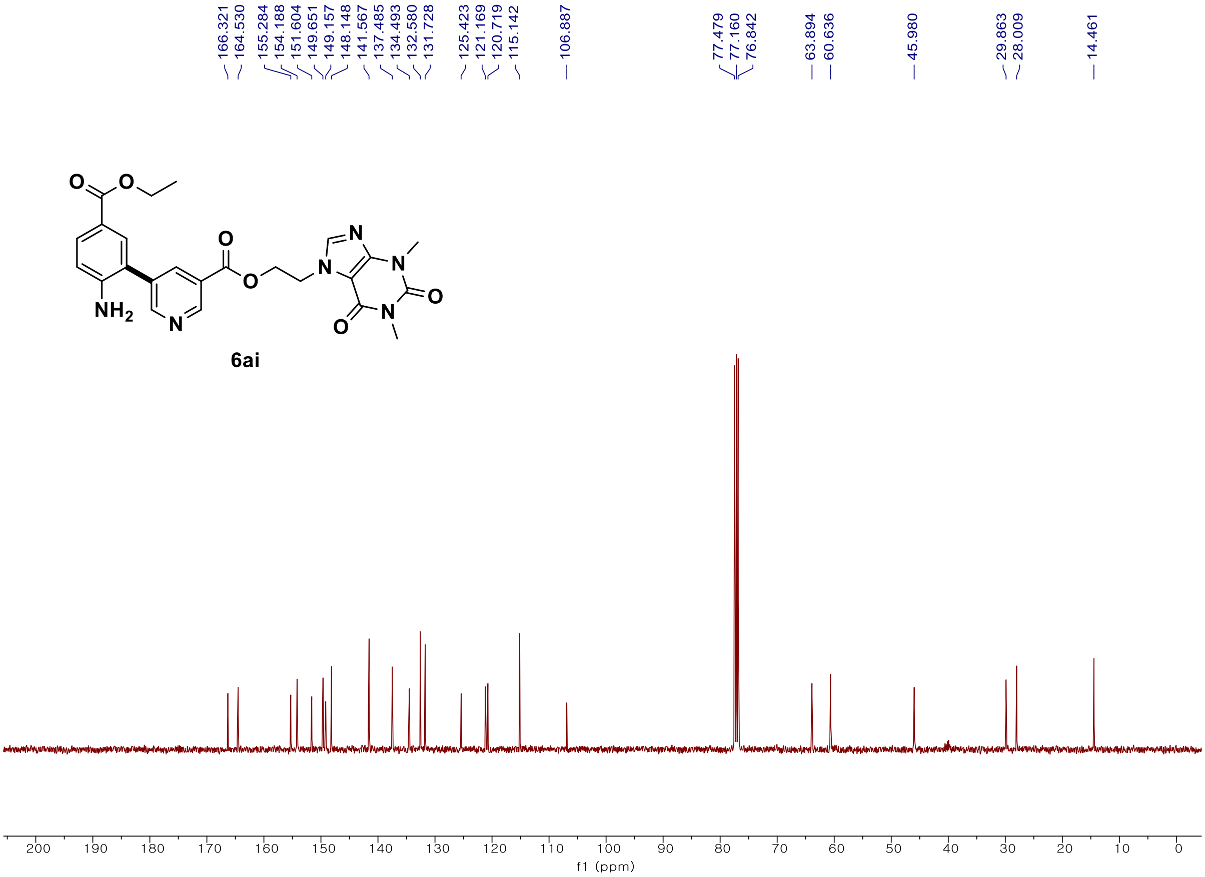
166.321
164.530
155.284
154.188
151.604
149.651
149.157
148.148
141.567
137.485
134.493
132.580
131.728
125.423
121.169
120.719
115.142
106.887
77.479
77.160
76.842
63.894
60.636
45.980
29.863
28.009
14.461
NH2
6ai
f1 (ppm)

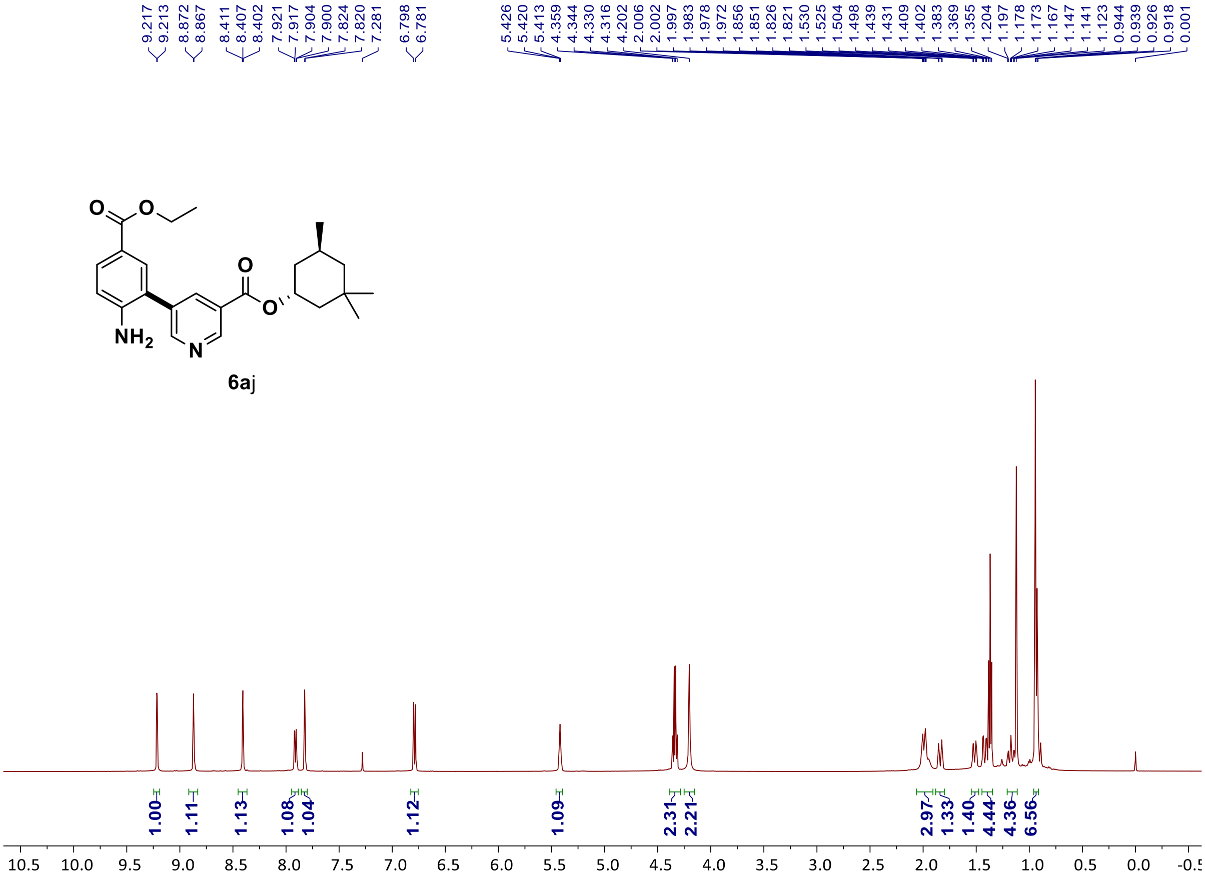
9.217
9.213
8.872
8.867
8.411
8.407
8.402
7.921
7.917
7.904
7.900
7.824
7.820
7.281
6.798
6.781
5.426
5.420
5.413
4.359
4.344
4.330
4.316
4.202
2.006
2.002
1.997
1.983
1.978
1.972
1.856
1.851
1.826
1.821
1.530
1.525
1.504
1.498
1.439
1.431
1.409
1.402
1.383
1.369
1.355
1.204
1.197
1.178
1.173
1.167
1.147
1.141
1.123
0.944
0.939
0.926
0.918
0.001
NH_2
N
6aj
1.00
1.11
1.13
1.08
1.04
1.12
1.09
2.31
2.21
2.97
1.33
1.40
4.44
4.36
6.56
10.5 10.0 9.5 9.0 8.5 8.0 7.5 7.0 6.5 6.0 5.5 5.0 4.5 4.0 3.5 3.0 2.5 2.0 1.5 1.0 0.5 0.0 -0.5

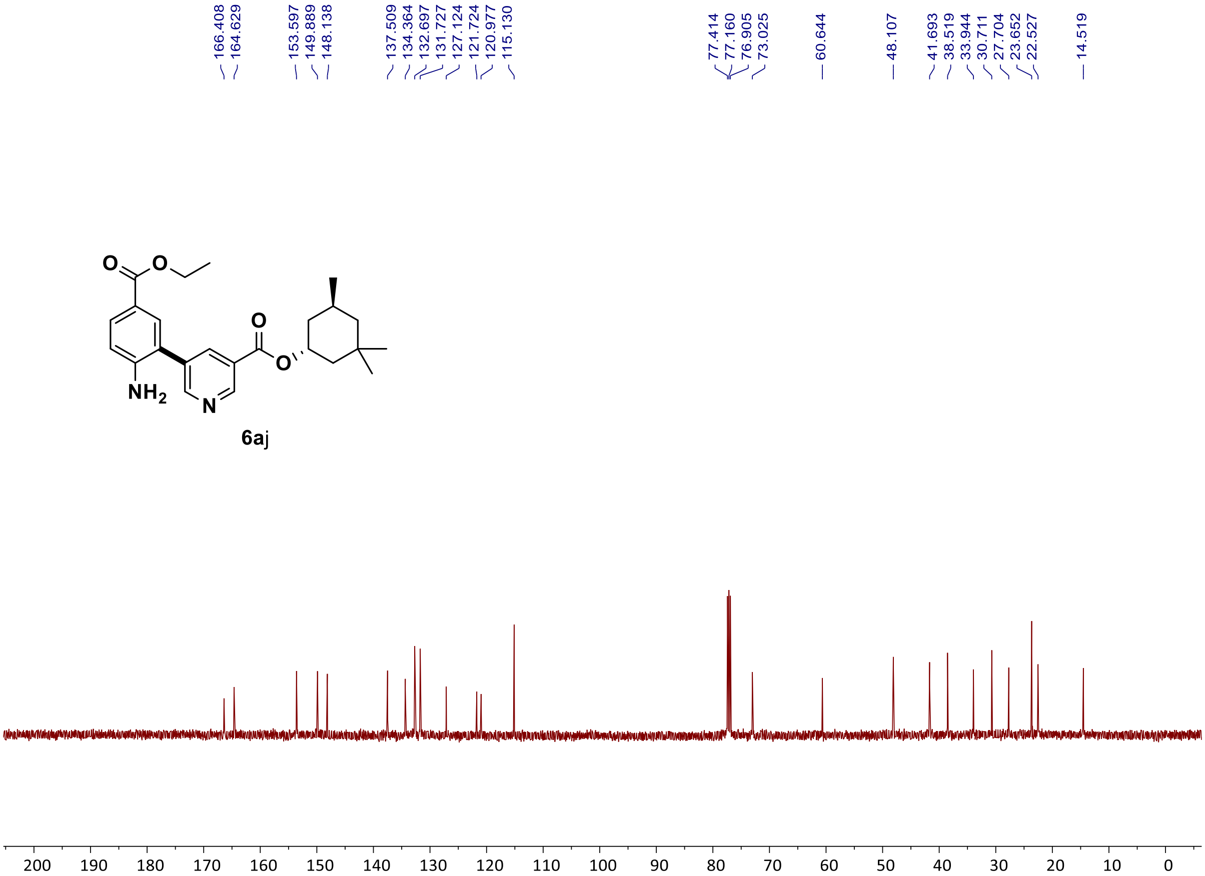
166.408
164.629
153.597
149.889
148.138
137.509
134.364
132.697
131.727
127.124
121.724
120.977
115.130
77.414
77.160
76.905
73.025
60.644
48.107
41.693
38.519
33.944
30.711
27.704
23.652
22.527
14.519
NH_2
N
6aj
200 190 180 170 160 150 140 130 120 110 100 90 80 70 60 50 40 30 20 10 0

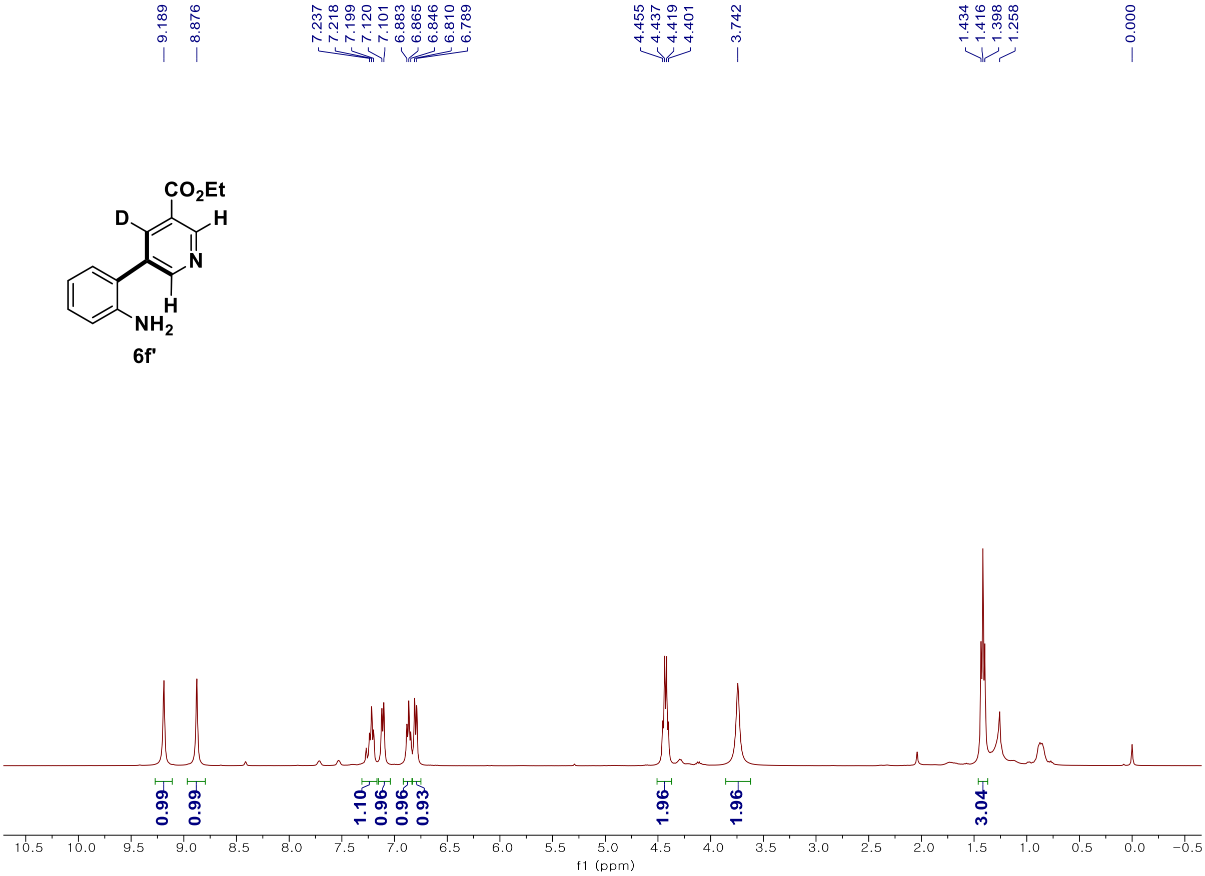

9.189
8.876
7.237
7.218
7.199
7.120
7.101
6.883
6.865
6.846
6.810
6.789
4.455
4.437
4.419
4.401
3.742
1.434
1.416
1.398
1.258
0.000
CO2Et
D
H
N
H
NH2
6f'
0.99
0.99
1.10
0.96
0.96
0.93
1.96
1.96
3.04
f1 (ppm)

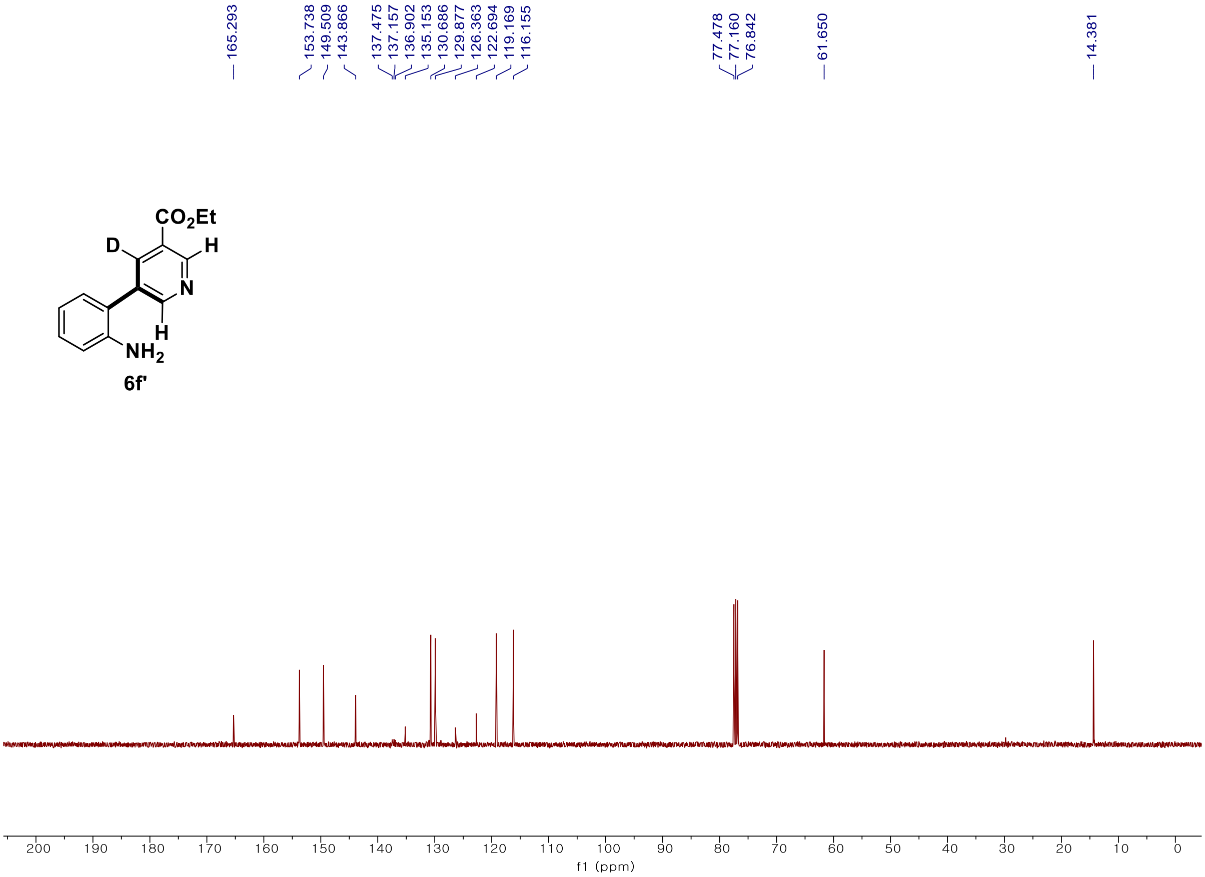

165.293
153.738
149.509
143.866
137.475
137.157
136.902
135.153
130.686
129.877
126.363
122.694
119.169
116.155
77.478
77.160
76.842
61.650
14.381
CO2Et
D
H
N
H
NH2
6f'
f1 (ppm)

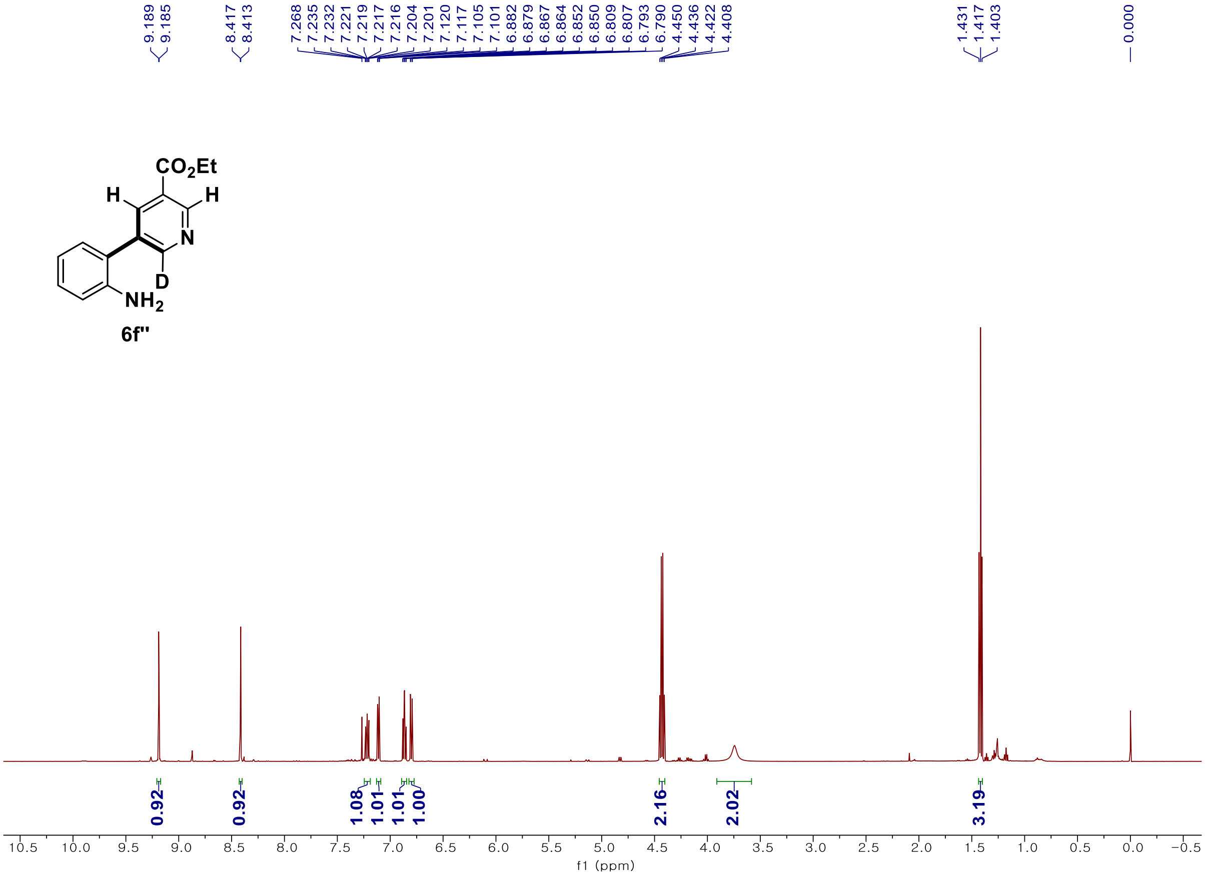
9.189
9.185
8.417
8.413
7.268
7.235
7.232
7.221
7.219
7.217
7.216
7.204
7.201
7.120
7.117
7.105
7.101
6.882
6.879
6.867
6.864
6.852
6.850
6.809
6.807
6.793
6.790
4.450
4.436
4.422
4.408
1.431
1.417
1.403
0.000
CO2Et
H
H
N
D
NH2
6f''
0.92
0.92
1.08
1.01
1.01
1.00
2.16
2.02
3.19
f1 (ppm)

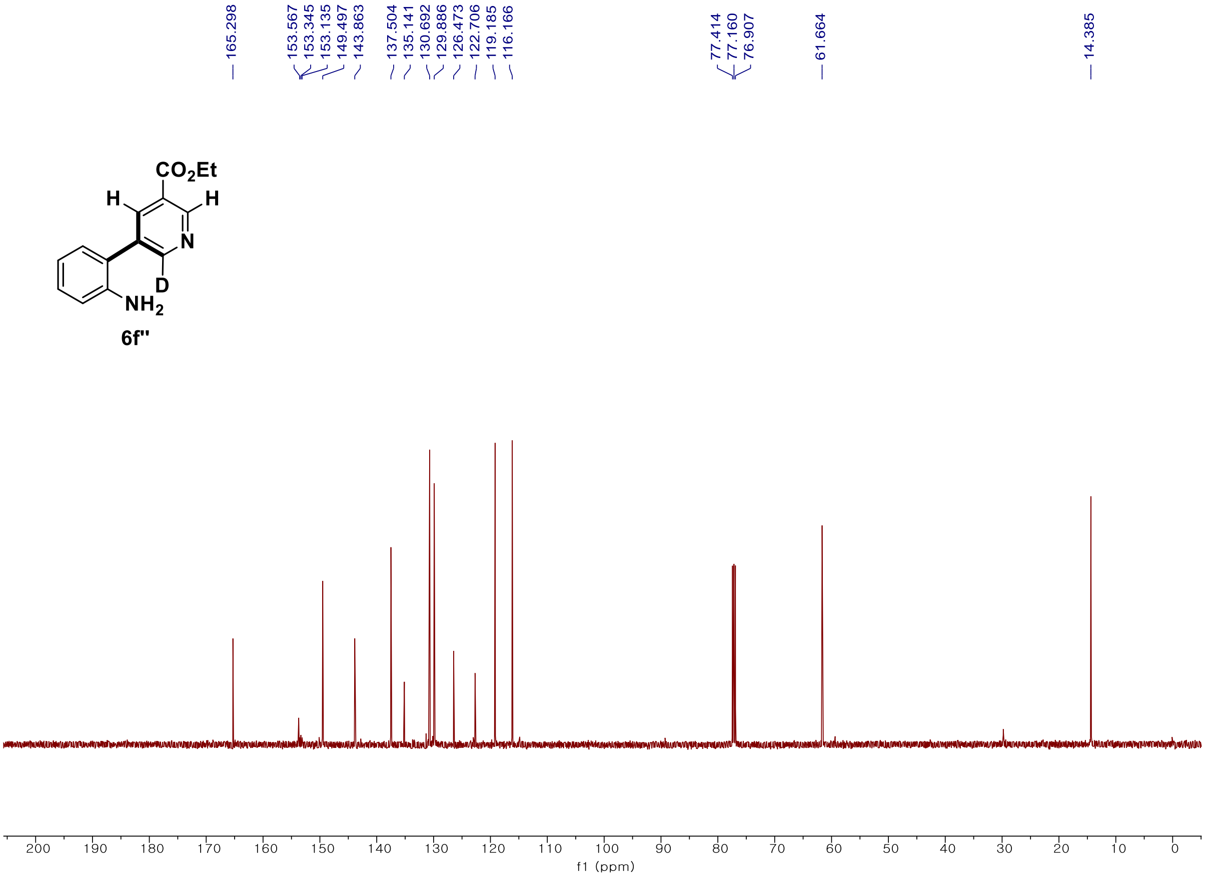
165.298
153.567
153.345
153.135
149.497
143.863
137.504
135.141
130.692
129.886
126.473
122.706
119.185
116.166
77.414
77.160
76.907
61.664
14.385
CO2Et
H
H
N
D
NH2
6f''
f1 (ppm)

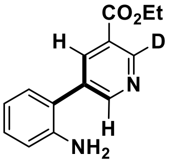
CO2Et
H
D
N
H
NH2

6f'''

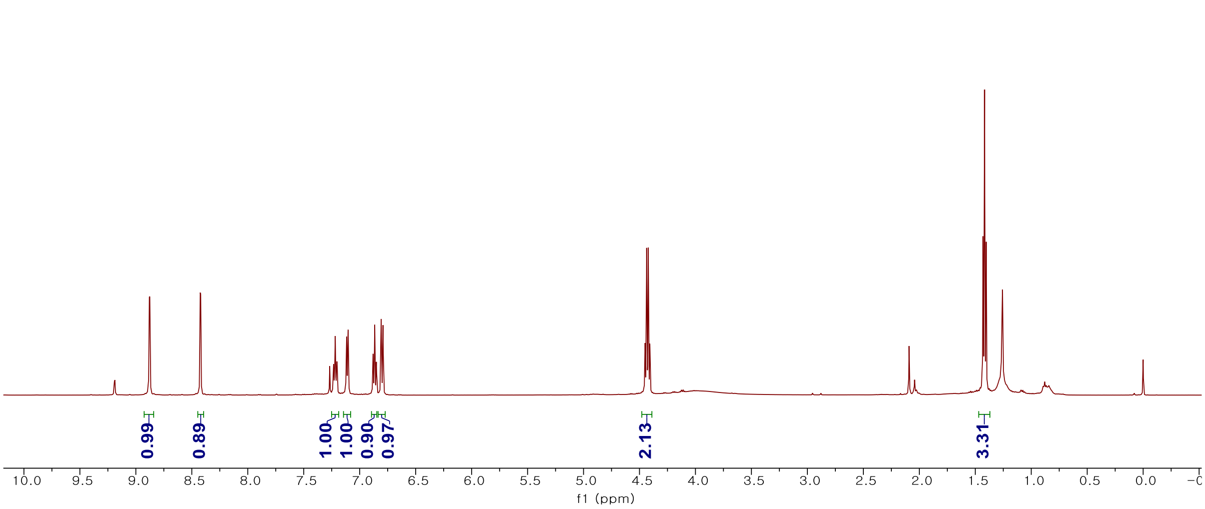
8.879
8.875
8.425
8.420
7.268
7.236
7.233
7.221
7.217
7.205
7.202
7.119
7.116
7.104
7.101
6.882
6.880
6.867
6.865
6.852
6.850
6.809
6.807
6.793
6.790
4.450
4.436
4.422
4.408
1.431
1.417
1.403
-0.000
0.99
0.89
1.00
1.00
0.90
0.97
2.13
3.31
f1 (ppm)

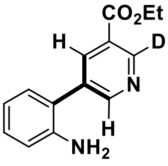
CO2Et
H
D
N
H
NH2

6f'''

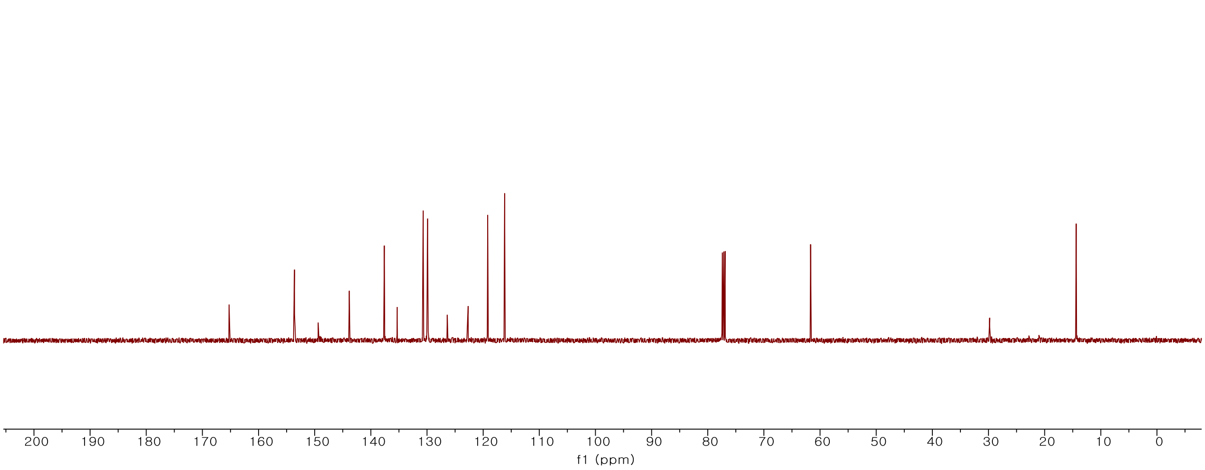
165.255
153.603
149.268
149.047
148.835
143.843
137.597
135.327
130.689
129.900
126.392
122.678
119.187
116.174
77.414
77.160
76.905
61.683
14.380
f1 (ppm)

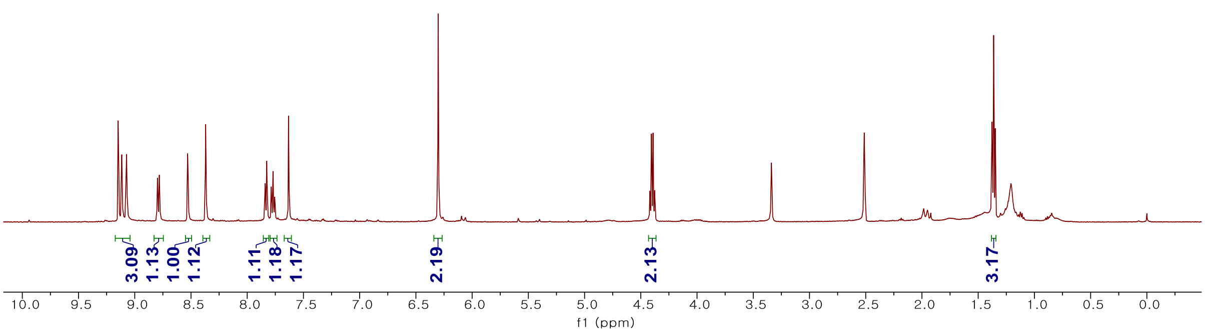
9.147
9.119
9.115
9.077
9.073
8.801
8.798
8.781
8.534
8.530
8.526
8.523
8.369
7.843
7.840
7.829
7.826
7.786
7.770
7.755
7.632
6.301
4.419
4.405
4.391
4.377
3.338
2.513
2.510
1.376
1.362
1.348
-0.000
3.09
1.13
1.00
1.12
1.11
1.18
1.17
2.19
2.13
3.17
10.0 9.5 9.0 8.5 8.0 7.5 7.0 6.5 6.0 5.5 5.0 4.5 4.0 3.5 3.0 2.5 2.0 1.5 1.0 0.5 0.0
f1 (ppm)

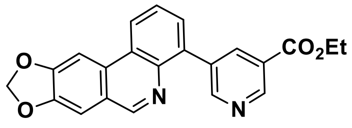
CO2Et
O
O
N
N

10a'

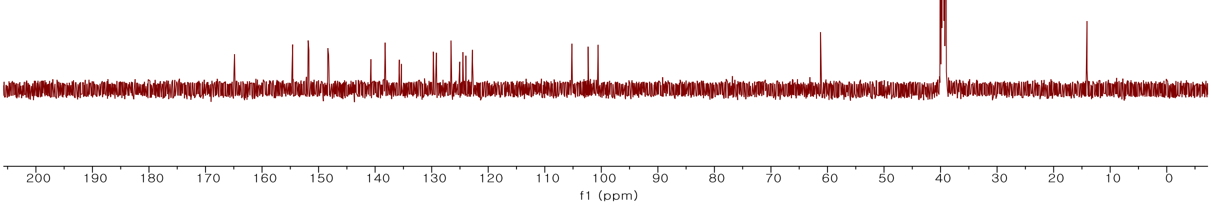
164.869
154.587
151.840
151.715
148.369
148.229
140.759
138.225
135.725
135.355
129.711
129.171
126.574
125.037
124.487
123.967
122.799
105.207
102.327
100.563
61.227
40.021
39.854
39.687
39.520
39.353
39.186
39.019
14.100
200 190 180 170 160 150 140 130 120 110 100 90 80 70 60 50 40 30 20 10 0
f1 (ppm)

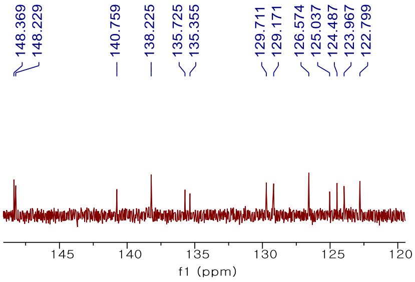
148.369
148.229
140.759
138.225
135.725
135.355
129.711
129.171
126.574
125.037
124.487
123.967
122.799
145 140 135 130 125 120
f1 (ppm)

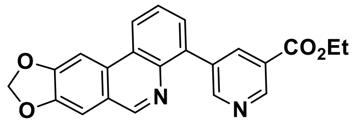
CO2Et
O
O
N
N

10a'

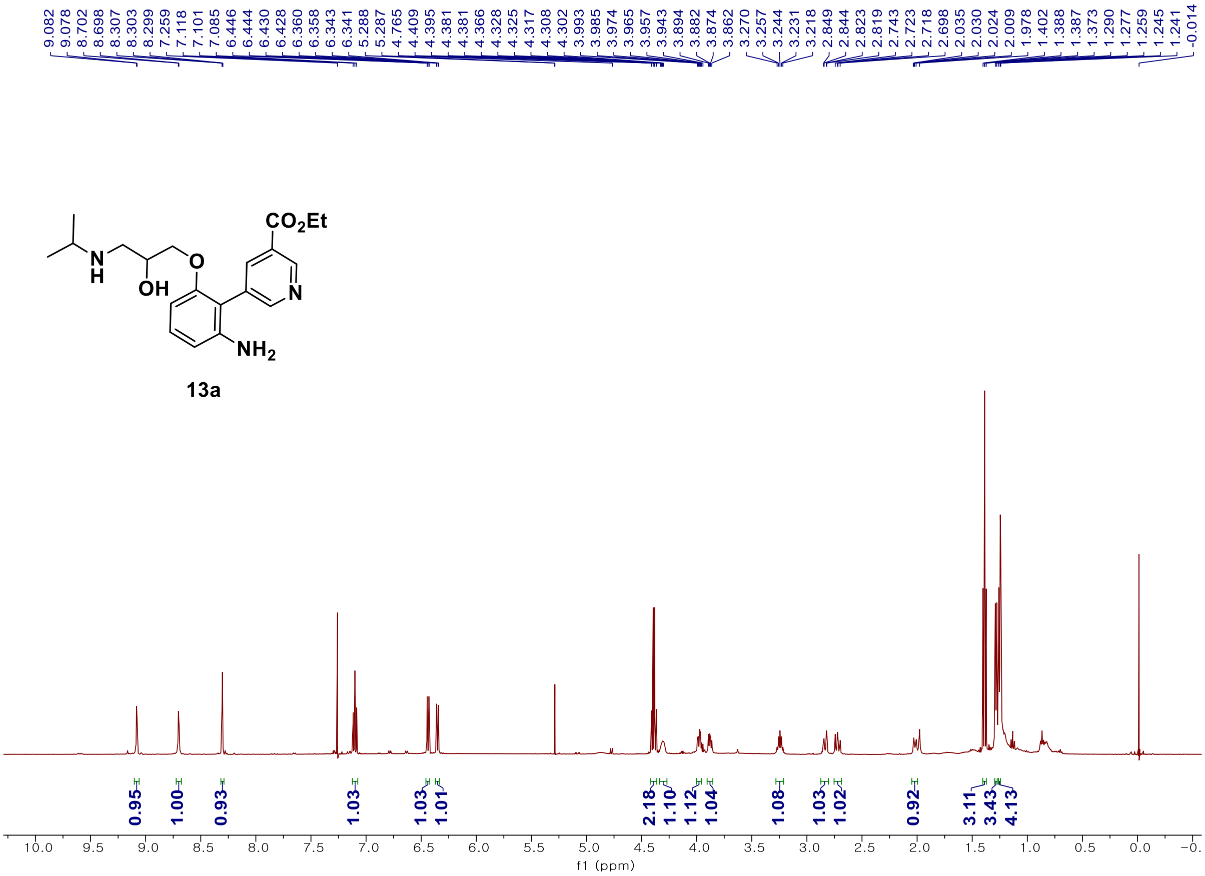
9.082 9.078 8.702 8.698 8.307 8.303 8.299 7.259 7.118 7.101 7.085 6.446 6.444 6.430 6.428 6.360 6.358 6.343 6.341 5.288 5.287 4.765 4.409 4.395 4.381 4.366 4.328 4.325 4.317 4.308 4.302 3.993 3.985 3.974 3.965 3.957 3.943 3.894 3.882 3.874 3.862 3.270 3.257 3.244 3.231 3.218 2.849 2.844 2.823 2.819 2.743 2.723 2.718 2.698 2.035 2.030 2.024 2.009 1.978 1.402 1.388 1.387 1.373 1.290 1.277 1.259 1.245 1.241 -0.014
CO2Et
N
H
OH
O
N
NH2
13a
0.95
1.00
0.93
1.03
1.03
1.01
2.18
1.10
1.12
1.04
1.08
1.03
1.02
0.92
3.11
3.43
4.13
f1 (ppm)

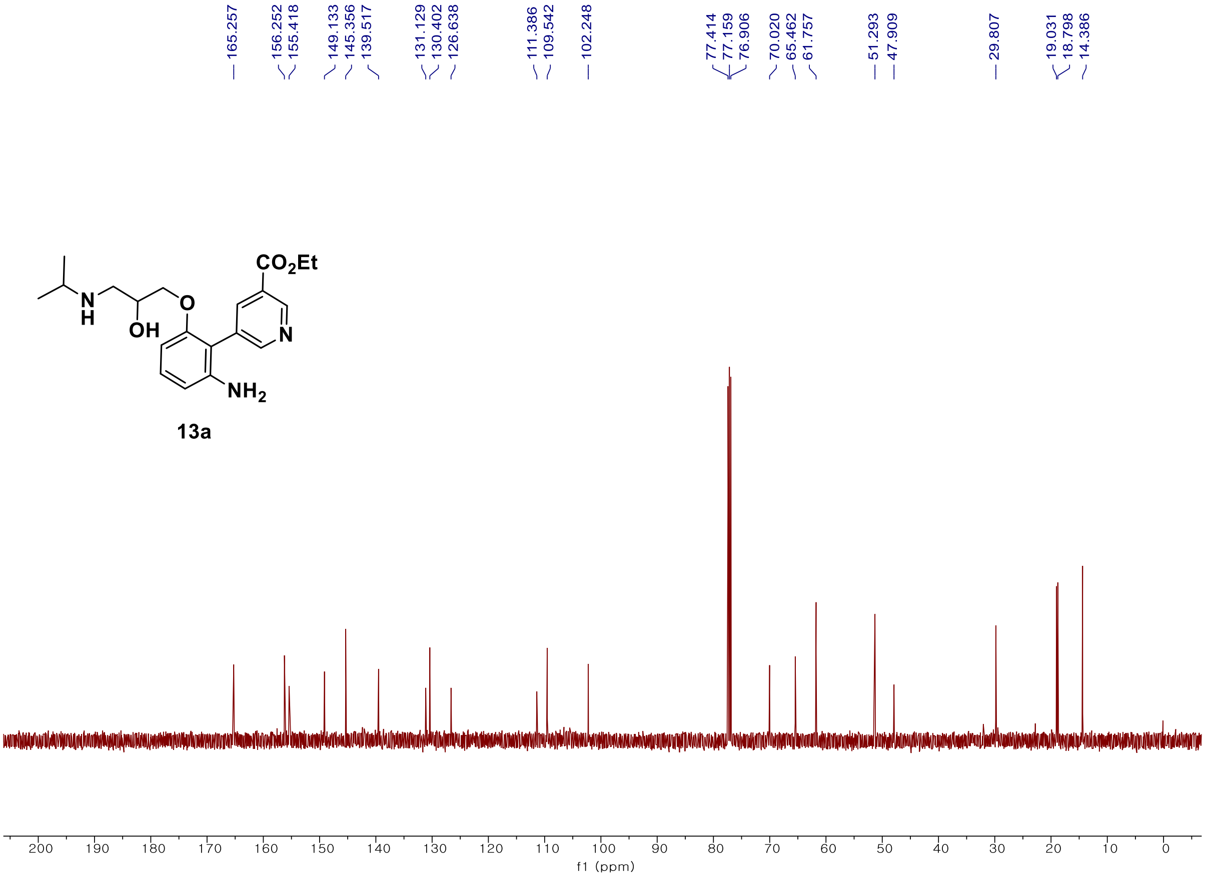
165.257 156.252 155.418 149.133 145.356 139.517 131.129 130.402 126.638 111.386 109.542 102.248 77.414 77.159 76.906 70.020 65.462 61.757 51.293 47.909 29.807 19.031 18.798 14.386
CO2Et
N
H
OH
O
N
NH2
13a
f1 (ppm)

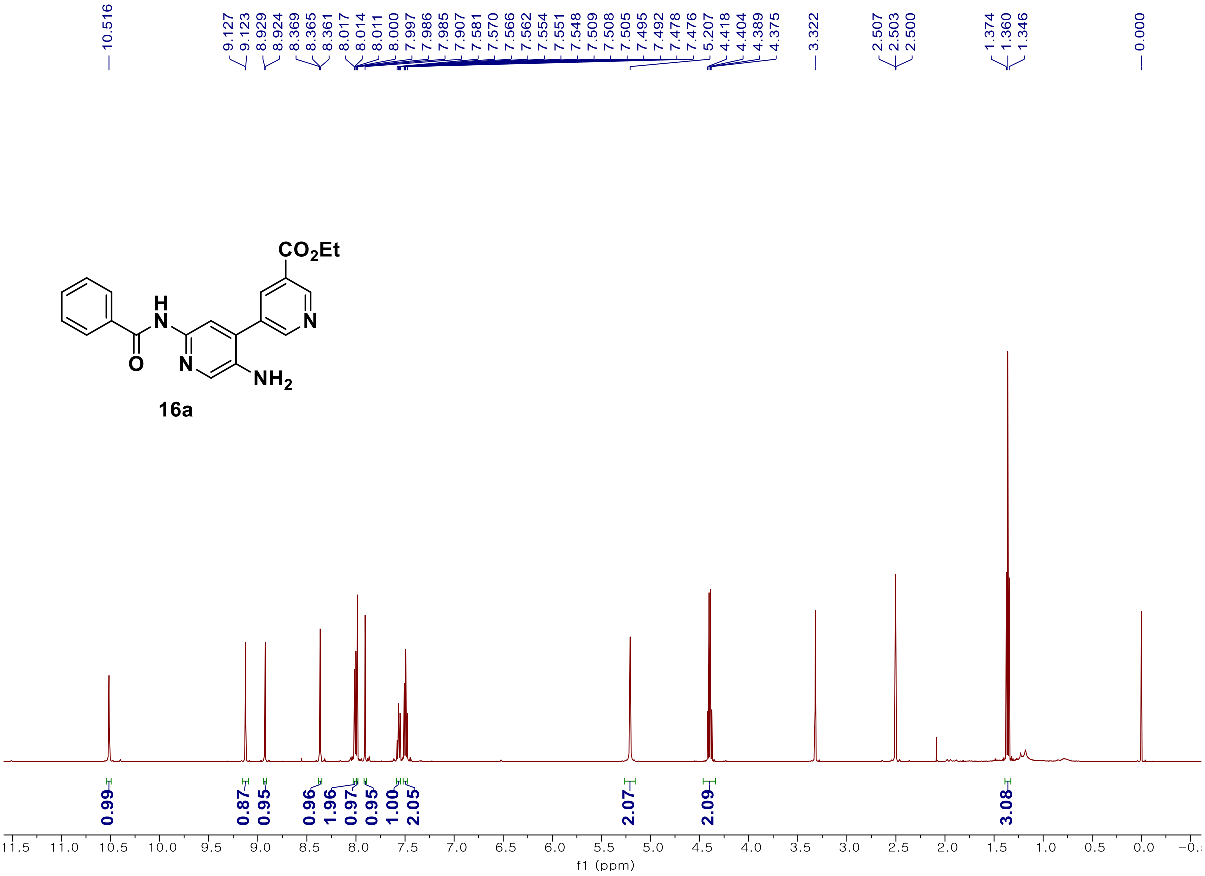
10.516
9.127
9.123
8.929
8.924
8.369
8.365
8.361
8.017
8.014
8.011
8.000
7.997
7.986
7.985
7.907
7.581
7.570
7.566
7.562
7.554
7.551
7.548
7.509
7.508
7.505
7.495
7.492
7.478
7.476
5.207
4.418
4.404
4.389
4.375
3.322
2.507
2.503
2.500
1.374
1.360
1.346
0.000
CO2Et
H
N
N
N
NH2
O
16a
0.99
0.87
0.95
0.96
1.96
0.97
0.95
1.00
2.05
2.07
2.09
3.08
f1 (ppm)

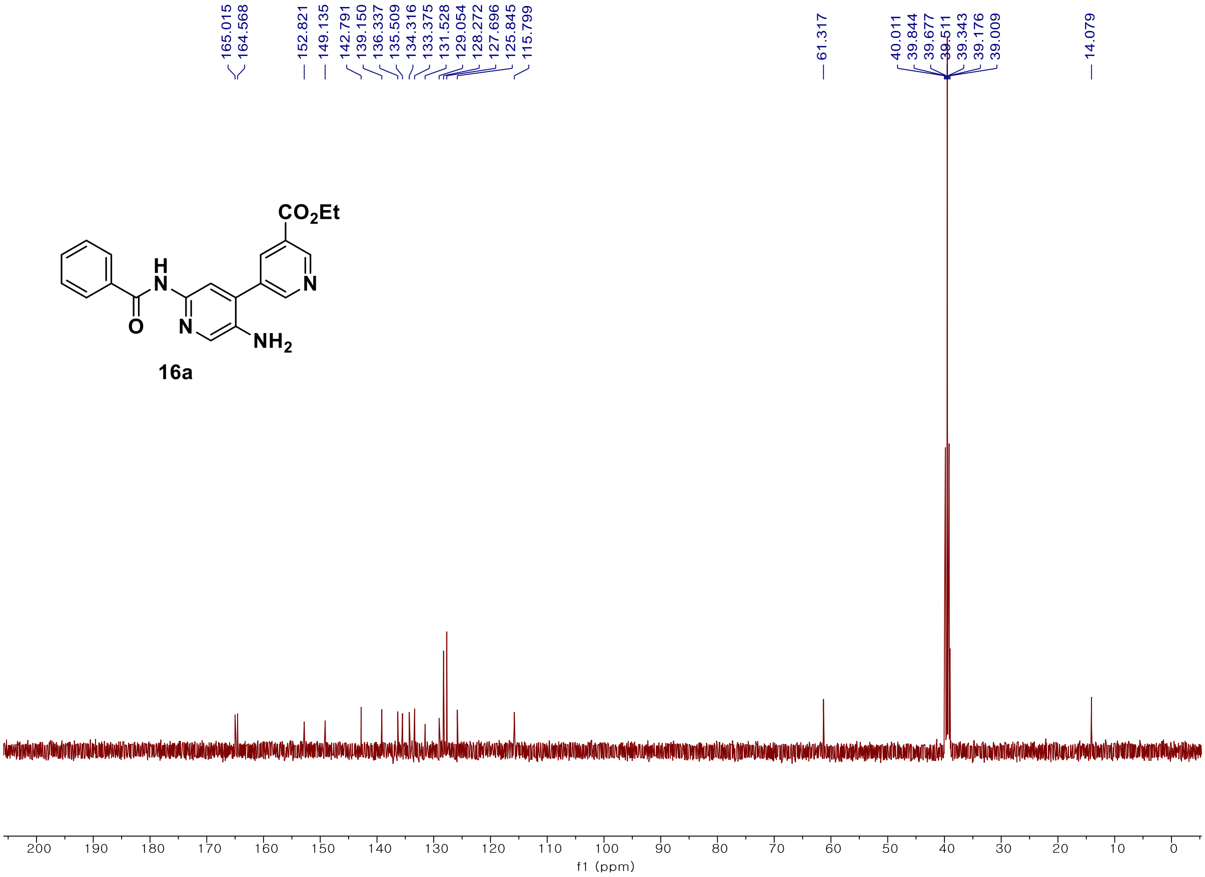
165.015
164.568
152.821
149.135
142.791
139.150
136.337
135.509
134.316
133.375
131.528
129.054
128.272
127.696
125.845
115.799
61.317
40.011
39.844
39.677
39.511
39.343
39.176
39.009
14.079
CO2Et
H
N
N
N
NH2
O
16a
f1 (ppm)

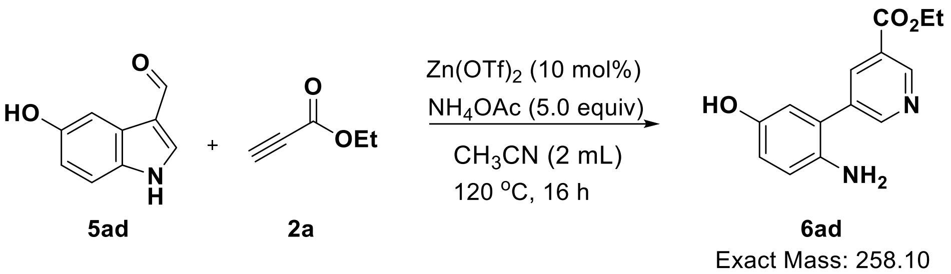
HO
O
N
H
5ad
+
O
OEt
2a
Zn(OTf)2 (10 mol%)
NH4OAc (5.0 equiv)
CH3CN (2 mL)
120 oC, 16 h
CO2Et
HO
N
NH2
6ad
Exact Mass: 258.10

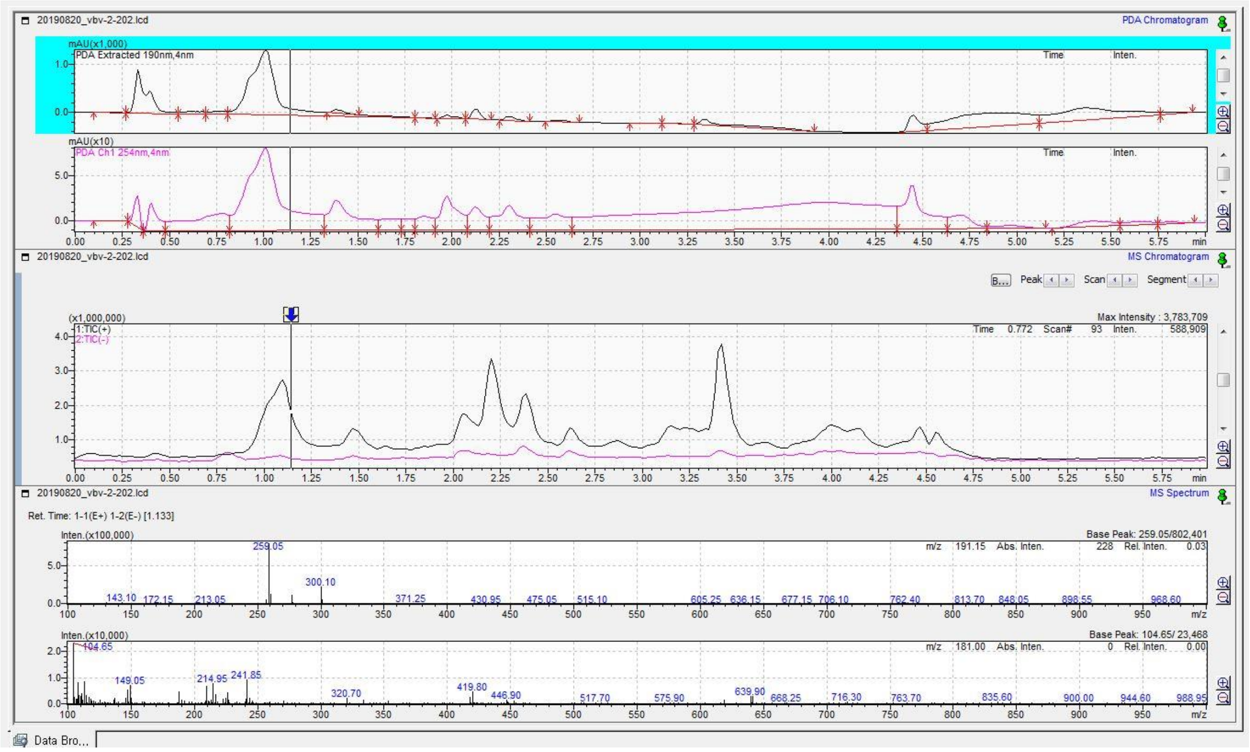
20190820_vbv-2-202.lcd
PDA Chromatogram
PDA Extracted 190nm,4nm
PDA Ch1 254nm,4nm
MS Chromatogram
Max Intensity : 3,783,709
1:TIC(+)
2:TIC(-)
MS Spectrum
Ret. Time: 1-1(E+) 1-2(E-) [1.133]
Base Peak: 259.05/802,401
259.05
300.10
Base Peak: 104.65/ 23,468
104.65
149.05
214.95
241.85

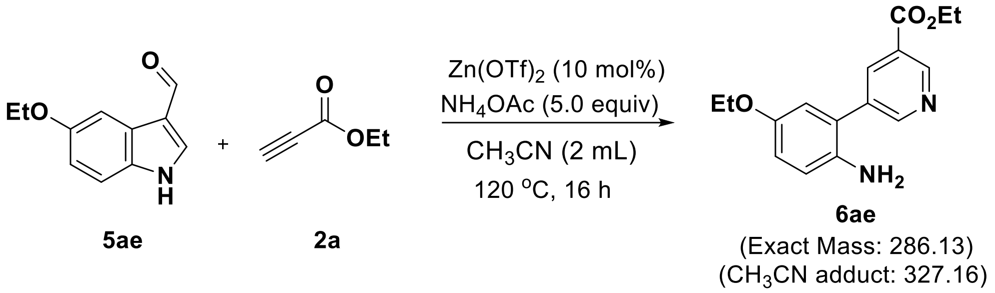
EtO
O
N
H
5ae
+
O
OEt
2a
Zn(OTf)2 (10 mol%)
NH4OAc (5.0 equiv)
CH3CN (2 mL)
120 °C, 16 h
CO2Et
EtO
N
NH2
6ae
(Exact Mass: 286.13)
(CH3CN adduct: 327.16)

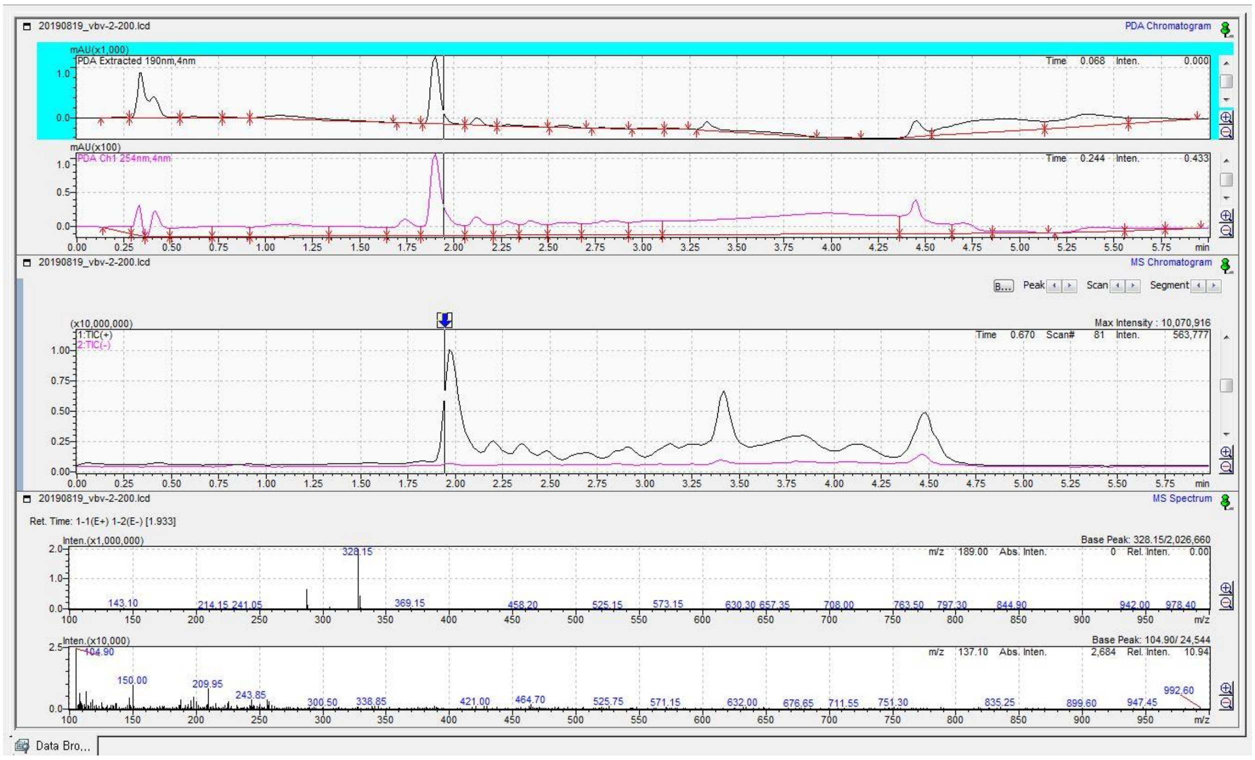
20190819_vbv-2-200.lcd
PDA Chromatogram
PDA Extracted 190nm,4nm
PDA Ch1 254nm,4nm
MS Chromatogram
Max Intensity : 10,070,916
1:TIC(+)
2:TIC(-)
MS Spectrum
Ret. Time: 1-1(E+) 1-2(E-) [1.933]
Base Peak: 328.15/2,026,660
328.15
Base Peak: 104.90/ 24,544
104.90
Data Bro...

VII. Supplementary References

1) Zhang, M. *et al.* Synthesis and antifungal activity of novel streptochlorin analogues. *Eur. J. Med. Chem.* **92**, 776−783 (2015).
2) Allais, C., Grassot, J. M., Rodriguez, J. & Constantieux, T. Metal-free multicomponent syntheses of pyridines. *Chem. Rev.* **114**, 10829–10868 (2014).
3) Yamamoto, Y., Gridnev, I. D., Patil, N. T. & Jin, T. Alkyne activation with Brønsted acids, iodine, or gold complexes, and its fate leading to synthetic application. *Chem. Commun.* 5075–5087 (2009).
4) Chen, Shaoqing, C. & Achyutharao, S. Azaindole thiazolinones, US2006/84674 A1 (2006).
5) Lee, S. & Park, S. B. An efficient one-step synthesis of heterobiaryl pyrazolo[3,4-ù] pyridines via indole ring opening. *Org. Lett.* **11**, 5214–5217 (2009).
6) Hoque, M. E. *et al.* Non-covalent Interactions in Ir-Catalyzed C–H Activation: L-Shaped Ligand for Para- Selective Borylation of Aromatic Esters. *J. Am. Chem. Soc*. **139**, 7745−7748 (2017).
7) Štarha, P., Trávníček, Z., Crlíková, H., Vančo, J., Kašpárková, J. & Dvořák, Z. Half-Sandwich Ir(III) Complex of *N*1-Pyridyl-7-azaindole Exceeds Cytotoxicity of Cisplatin at Various Human Cancer Cells and 3D Multicellular Tumor Spheroids. *Organometallics* **37**, 2749−2759 (2018).
8) Archer, W., J. Cook, R. & Taylor, R. Electrophilic Aromatic Substitution. Part 34. Partial Rate Factors for Detritiation of Dithieno[l,2-*b*:4,3-*b*'] benzene, Dithieno[l,2-*b*:3,4- *b*'] benzene, and Dithieno[2,1-*b*:3,4-*b*'] benzene. *J. Chem. Soc. Perkin Trans.* **II**, 813−819 (1983).
9) Amir-Heidari, B., Thirlway, J. & Micklefield, J. Stereochemical Course of Tryptophan Dehydrogenation during Biosynthesis of the Calcium-Dependent Lipopeptide Antibiotics. *Org. Lett.* **9**, 1513–1516 (2007).
10) Maresh, J. J. *et al.* Strictosidine Synthase: Mechanism of a Pictet-Spengler Catalyzing Enzyme. *J. Am. Chem. Soc.* **130**, 710–723 (2008).
11) Harrowven, D. C., Lai, D. & Lucas, M. C. A Short Synthesis of Hippadine. *Synthesis.* 1300–1302 (1999).
12) Ollis, W., D. Sutherland, I., O. & Thebtaranonth, Y. Base Catalysed Rearrangements Involving Ylide Intermediates. The Preparation and Reactions of 2-Oxidoanilinium Ylides. *J. Chem. Soc. Perkin Trans.* **I**, 1981−1993 (1981).
